# A comparative analysis of serum and tissue proteomic profiles in non-small cell lung cancer patients with or without brain metastasis

**DOI:** 10.1038/s41420-026-03109-8

**Published:** 2026-04-13

**Authors:** Yongtao Zheng, Yueting Xiong, Yuxiao Ma, Yijie Qiu, Qingfang Bu, Zhenxi Wang, Qingfang Sun, Yuhao Sun, Xiaohui Liu, Quan Yuan, Yuping Li, Liuguan Bian, Baofeng Wang

**Affiliations:** 1 Department of Neurosurgery, Ruijin Hospital, Shanghai Jiao Tong University School of Medicine, Shanghai, China; 2 State Key Laboratory of Vaccines for Infectious Diseases, Xiang An Biomedicine Laboratory, Xiamen University, Xiamen, Fujian China; 3 Zhongshan Hospital, Fudan University, Shanghai, China; 4 Institute of Translational Medicine, Shanghai Jiao Tong University, Shanghai, China; 5 Department of Thoracic Surgery, Shanghai Pulmonary Hospital, School of Medicine, Tongji University, Shanghai, China

**Keywords:** CNS cancer, Biochemistry

## Abstract

To identify specific, sensitive, and non-invasive circulating protein biomarkers that could facilitate the diagnosis of brain metastasis (BrM) and improve risk prediction for BrM among patients with non-small cell lung cancer (NSCLC). We performed data-independent acquisition mass spectrometry (DIA-MS)-based proteomic profiling of 14 tissue specimens obtained from 7 patients, together with 89 serum samples from NSCLC and NSCLC-BrM cohorts, to identify candidate biomarkers associated with BrM. A total of 12,808 proteins were identified in the tissue proteome and 6041 proteins in the serum proteome, representing an extensive proteomic analysis of lung cancer with BrM reported to date. Using integrated analyses, we identified a four-protein classifier that served as biomarkers for predicting the risk of NSCLC metastasis to the brain. Notably, PSMA4, LAP3, and LZIC were consistently downregulated in both the sera and tissues of patients with NSCLC-BrM compared with those with NSCLC without BrM. These biomarkers were subsequently validated by ELISA in an additional cohort, demonstrating high concordance with the PRM results. Immunohistochemical analyses further supported the utility of these proteins in distinguishing BrM from primary brain tumors. The integrated analysis of tissue and serum proteomics across the cohorts supports the potential value of proteomics-guided, biomarker-assisted diagnosis and risk prediction in BrM and may help enable more accurate stratification and more targeted treatment strategies.

## Introduction

Lung cancer (LC) is among the most common malignant tumors worldwide and causes more than one million deaths each year. Among all LC cases, approximately 85% are classified as non-small cell LC (NSCLC) [1, 2], and this subtype has a strong tendency to spread to the brain, contributing to an estimated 24–44% of all brain metastasis (BrM) cases [3]. Once BrM develops, progression is often rapid and the prognosis is poor, with overall survival typically limited to about 3–6 months [4, 5]. Although surgery combined with radiotherapy and chemotherapy can provide clinical benefit for selected patients, outcomes remain unsatisfactory for most individuals with BrM [6, 7]. Therefore, if BrM risk can be identified early, preventive or earlier therapeutic strategies could be initiated promptly, which may translate into improved survival. At present, BrM is mainly diagnosed using routine contrast-enhanced magnetic resonance imaging (MRI) of the brain; however, MRI may miss lesions at an early stage because detectable findings often appear only after blood-brain barrier disruption has occurred to a certain extent, which limits its value for early diagnosis [8, 9]. Furthermore, some patients do not seek hospital care specifically for MRI screening when neurological symptoms are mild or absent, further contributing to delayed detection.

LC cells metastasizing to the brain represent a complex process characterized by a unique immune environment, intricate cell-cell interactions, and the involvement of multiple molecular factors. When LC cells invade adjacent small veins or capillaries, they can readily enter the circulation, travel to distant organs, and eventually establish metastases. Although the blood-brain barrier serves as a protective mechanism that limits the entry of cancer cells into the central nervous system, tumor cells in the metastatic microenvironment can undergo cellular reprogramming and trigger inflammatory responses with cytokine release, which in turn disrupts the integrity of the blood-brain barrier [10, 11]. Therefore, early diagnosis and intervention before cancer cells induce substantial blood-brain barrier disruption are important for reducing both the incidence and progression of BrM. Changes in human plasma or serum proteins have been widely used as molecular biomarkers for early tumor screening because they are minimally invasive and broadly accessible, and, in this context, mass spectrometry (MS)-based proteomics approaches provide valuable insights into unbiased biomarker discovery followed by targeted validation associated with tumor progression [12–16].

However, the serum proteome presents a major challenge because high-abundance proteins, such as albumin and immunoglobulins, constitute most of the total protein content and can suppress the detection of low-abundance candidates [17]. Depletion of high-abundance proteins is therefore advantageous, as it compresses the dynamic range of serum samples, reduces spectral interference, and can markedly increase the depth of proteome coverage. Data-independent acquisition (DIA) MS provides a robust platform for large-scale clinical proteomics by systematically fragmenting all detectable peptides, and, because this approach is less dependent on stochastic precursor selection, it supports high data integrity and reproducibility across batches. In addition, its cyclic acquisition mode can improve the detection of low-abundance proteins by reducing signal interference [18], which makes high-abundance protein depletion-based DIA particularly suitable for biomarker discovery in complex biofluids. Here, we employed DIA-MS-based quantitative proteomics to analyze paired BrM tissue samples (*n* = 14) and serum specimens (*n* = 89) from patients with NSCLC-BrM and from those with NSCLC only, together with age- and sex-matched healthy volunteers, to identify circulating biomarkers that could predict the risk of NSCLC metastasizing to the brain. We observed that both the BrM tissue proteome and the serum proteome undergo substantial remodeling during NSCLC BrM, and the integrated tissue-serum proteomics strategy enabled the identification of signature proteins. Building on these results, we applied a machine-learning strategy to define serum-based proteomic biomarker panels capable of distinguishing patients with BrM from those with NSCLC alone, and this finding supports the potential value of liquid biopsy as an alternative to more invasive procedures. Collectively, this study provides an overview of tissue and serum proteomic changes in patients with NSCLC-BrM and offers a resource for further biological research, diagnostic development, and drug discovery.

## Results

### Experimental design for tissue and serum proteome profiling in NSCLC-BrM diseases

**Fig. 1:**
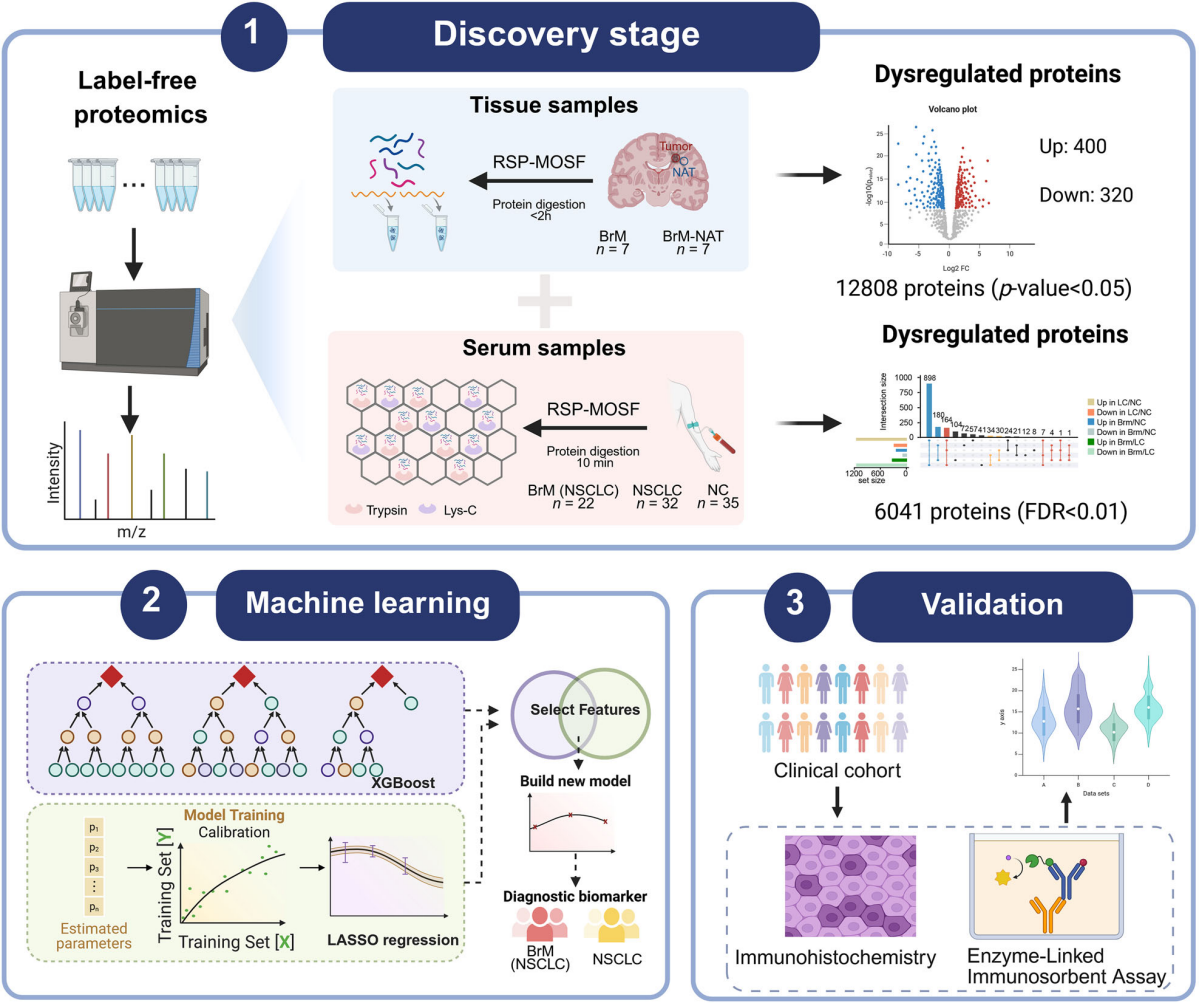
Workflow outlining the generation of the proteomic landscape for BrM and NSCLC. The RSP-MOSF method refers to rapid sample preparation (RSP) incorporating macroporous ordered siliceous foams (MOSF) to enhance proteome coverage.

The comprehensive experimental pipeline is illustrated in Fig. 1. To improve our understanding of the mechanisms underlying NSCLC metastasis to the brain, we first analyzed NSCLC-BrM tumor tissues together with paired non-cancerous adjacent tissues from seven Chinese patients using DIA quantitative proteomics, with the aim of identifying dysregulated proteins associated with the local disease microenvironment. In parallel, serum proteomics was performed on 89 serum samples (BrM, *n* = 22; NSCLC, *n* = 32; NC, *n* = 35) using our previously developed serum preparation strategy [19] to uncover altered proteins reflecting systemic changes and to support the identification of potential circulating biomarkers. By integrating differentially expressed proteins (DEPs) identified in tumor tissues versus non-cancerous adjacent tissues (NAT) with dysregulated proteins detected in serum, we constructed a multidimensional proteomic landscape to characterize NSCLC-BrM. Furthermore, machine learning models were developed using the serum proteomics data to identify significantly altered molecules that may reflect molecular changes associated with the progression from NSCLC to BrM. The detailed clinicopathological features are summarized in Supplementary Fig. 1.

### Proteome of BrM and non-cancerous adjacent tissues

**Fig. 2:**
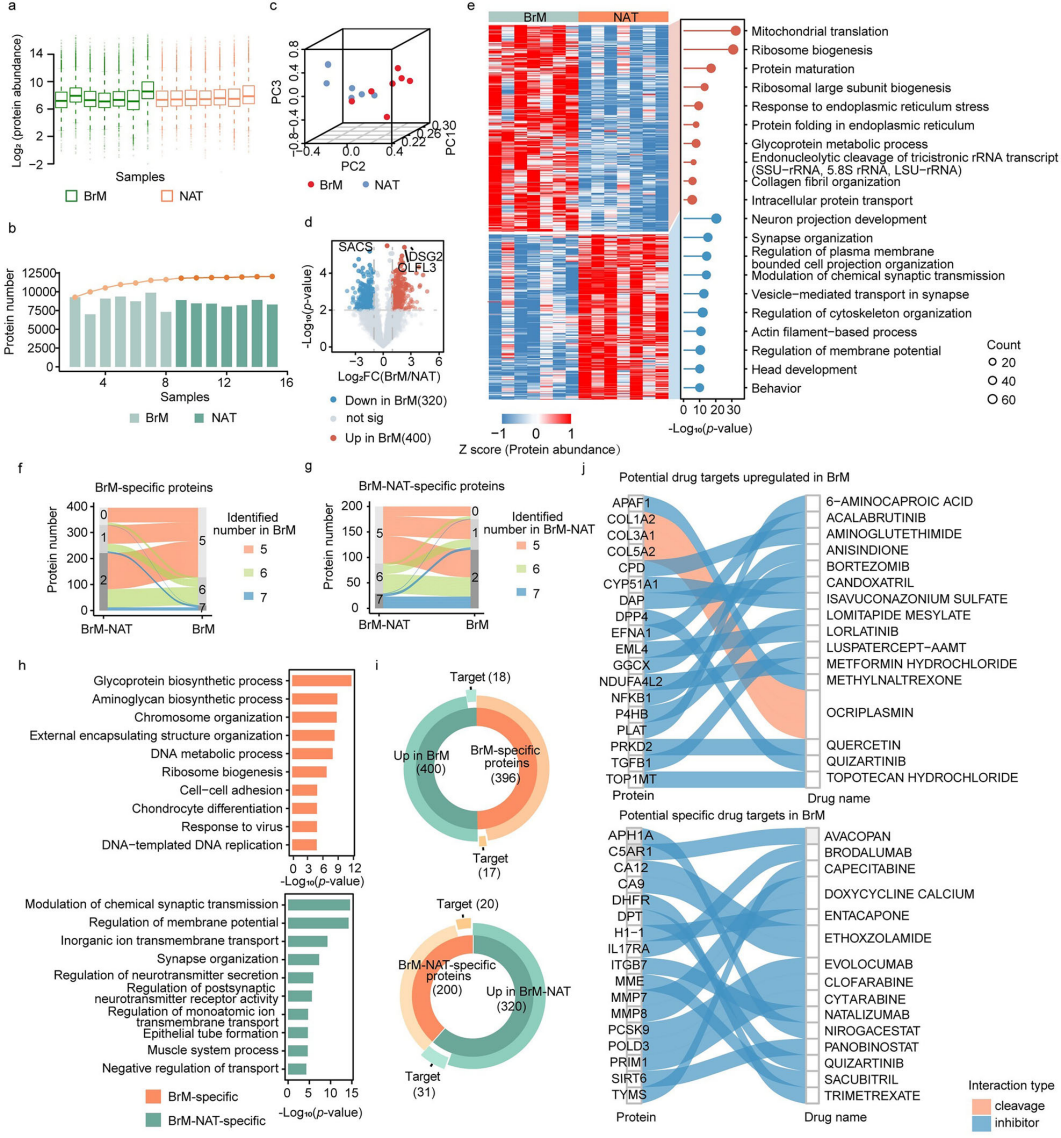
Tissue proteome profiles differ between BrM and normal adjacent tissue (NAT) samples. **a** Box plot showing the median protein abundance in BrM and NAT samples. **b** Bar chart showing the number of proteins identified in each sample, with a line plot showing the cumulative total number of identified proteins. **c** 3D PCA scatter plots showing the first three principal components of the protein level proteomic data for the BrM and NAT groups. **d** Volcano plots showing protein abundance differences between BrM and NAT samples. Differentially expressed proteins (DEPs) were identified using Student’s *t*-test (*p* < 0.05 and log2 (foldchange) > 1 or < -1); significant proteins are highlighted in red (upregulated) or blue (downregulated). **e** Differential protein patterns and functional enrichment analysis in BrM and NAT groups. The left heatmap shows DEPs between BrM and NAT, and the right panel shows the top 10 GO terms ranked by *p-*value for proteins enriched in the BrM and NAT groups. **f** Sankey diagram showing the number of BrM-specific proteins. **g** Sankey diagram showing the number of BrM-NAT-specific proteins. **h** GO analysis of BrM- and BrM-NAT-specific proteins. **i** Putative drug targets among proteins upregulated in BrM (or BrM-NAT) and among BrM (or BrM-NAT)-specific proteins. **j** Sankey diagram showing putative BrM drug targets and the corresponding drug-target interaction types.

To explore disease-related molecular features, we firstly analyzed protein expression profiles from brain tumor tissues and paired NATs using DIA, and all 14 tissue samples met the quality-control criteria (Fig. 2a). In total, we identified 12,808 proteins with high confidence (FDR < 0.01). On average, we detected 70,108 and 68,922 peptides in tumors and NATs, respectively, with counts ranging from 42,606 to 88,357 across cases (Fig. 2b). In addition, the tumor and NAT groups showed comparable distributions of protein intensity with closely matched identifications, supporting good reproducibility across replicates and stable MS performance (Fig. 2a, b). Notably, unsupervised clustering of the global proteome clearly separated normal tissues from tumor tissues (Fig. 2c), indicating that the dataset captures tumor-associated functional changes and can support subsequent analysis.

Using our predefined criteria for differential expression, we identified 720 proteins that were significantly dysregulated between BrM tissues and NAT, including 400 upregulated and 320 downregulated proteins (Fig. 2d; Supplementary Table 2; *p*-value < 0.05). Collectively, these DEPs represented approximately 5.6% of the total quantified proteome. Among them, desmoglein-2 (DSG2) showed marked upregulation in BrM in our dataset, and it has also been reported as a biomarker in an independent cervical cancer study, in which it promotes cancer cell proliferation and metastasis and is associated with poor prognosis in early-stage disease [20].

Furthermore, biological processes analysis demonstrated that the upregulated proteins in BrM were predominantly associated with pathways involved in ribosomal function, protein synthesis and folding, and the endoplasmic reticulum stress response (Fig. 2e, Supplementary Table 3). These pathways likely play a pivotal role in supporting the energy demands and protein maturation required for cancer cell progression in BrM. In contrast, the downregulated proteins in BrM were mainly linked to pathways related to neuronal development and synaptic structure pathways, highlighting the impact of brain tumors on central nervous system (CNS) development (Fig. 2e). Our data indicated that these biological processes might contribute to the formation of brain tumors in patients with NSCLC.

### Identification and characterization of tissue-specific enriched proteins

Cellular protein expression shows substantial variation across tissues and organs, and it also changes under different physiological and pathological conditions [21]. Therefore, a clear understanding of baseline human tissue transcriptome profiles is important for distinguishing disease-related changes within tissues and for identifying potential disease markers. To investigate tissue-specific proteins in our study, we focused on proteins uniquely detected in either BrM or BrM-NAT, and we compared the protein profiles of each tissue type with those obtained from the other tissue groups. Proteins detected in more than 60% of BrM or BrM-NAT samples but in fewer than 40% of samples from the other groups were defined as tissue-specific proteins.

A total of 396 BrM-specific proteins and 200 BrM-NAT-specific proteins were identified (Fig. 2f–g; Supplementary Table 4). We then analyzed the biological processes associated with these tissue-specific proteins using GO annotations. BrM-specific proteins were found to be involved in glycoprotein biosynthetic process and aminoglycan biosynthetic process, indicating distinct energy mediation mechanisms present in patients with BrM (Fig. 2h, Supplementary Table 3). In contrast, most BrM-NAT-specific proteins were primarily involved in complex neurological functions, including signal transduction, ion transport, synaptic function, and the regulation of the muscular system, thereby reflecting normal brain function (Fig. 2h). Taken together, these results indicate that profiling tissue-enriched proteins can provide useful information on their roles in maintaining tissue-specific biological functions.

Targeted cancer therapies and immunotherapies, which act on specific proteins in dysregulated pathways to inhibit tumor growth and spread, have supported the development of precision medicine and may offer additional treatment options for patients with BrM through therapies directed at relevant molecular targets [21–23]. As of 2015, among 1578 drug molecules approved by the United States Food and Drug Administration (FDA), 667 were human proteins, accounting for more than 75% of total drug targets, which suggests that there remains a broad range of therapeutic targets to explore (Supplementary Table 5). To identify potential therapeutic targets related to BrM-NAT, we performed a systematic search using DGIdb (https://www.dgidb.org/).

Our analysis focused mainly on proteins that were specific to BrM, as well as those significantly upregulated in this context, and we applied the same approach to BrM-NAT. In the BrM group, we identified 35 potential targets associated with 78 drugs (Fig. 2i, j), of which 38 were classified as antineoplastic agents. For drug-target interactions in this group, there were 2 cleavage agents and 76 inhibitors. Notably, CLOFARABINE exhibited inhibitory effects on the growth of mesothelioma cell lines (MSTO-211H and NCI-H2052) in vitro. CYTARABINE, an antimetabolite chemotherapy drug, functions by disrupting DNA synthesis and repair processes that are required for cancer cell growth and proliferation [24, 25]. In contrast, in the BrM-NAT group, we identified 51 potential targets corresponding to 160 drugs (Fig. 2i), with 36 targets categorized as antineoplastic agents. Among the drug-target interactions, there were 56 blockers, 102 inhibitors, and 6 negative modulators. These targets may represent candidates for targeted therapy; however, their therapeutic value still requires further validation through experimental studies and clinical trials to clarify their role in disease biology and their potential as treatment targets.

### Proteomic profiles among BrM, NSCLC, and normal control serum samples

**Fig. 3:**
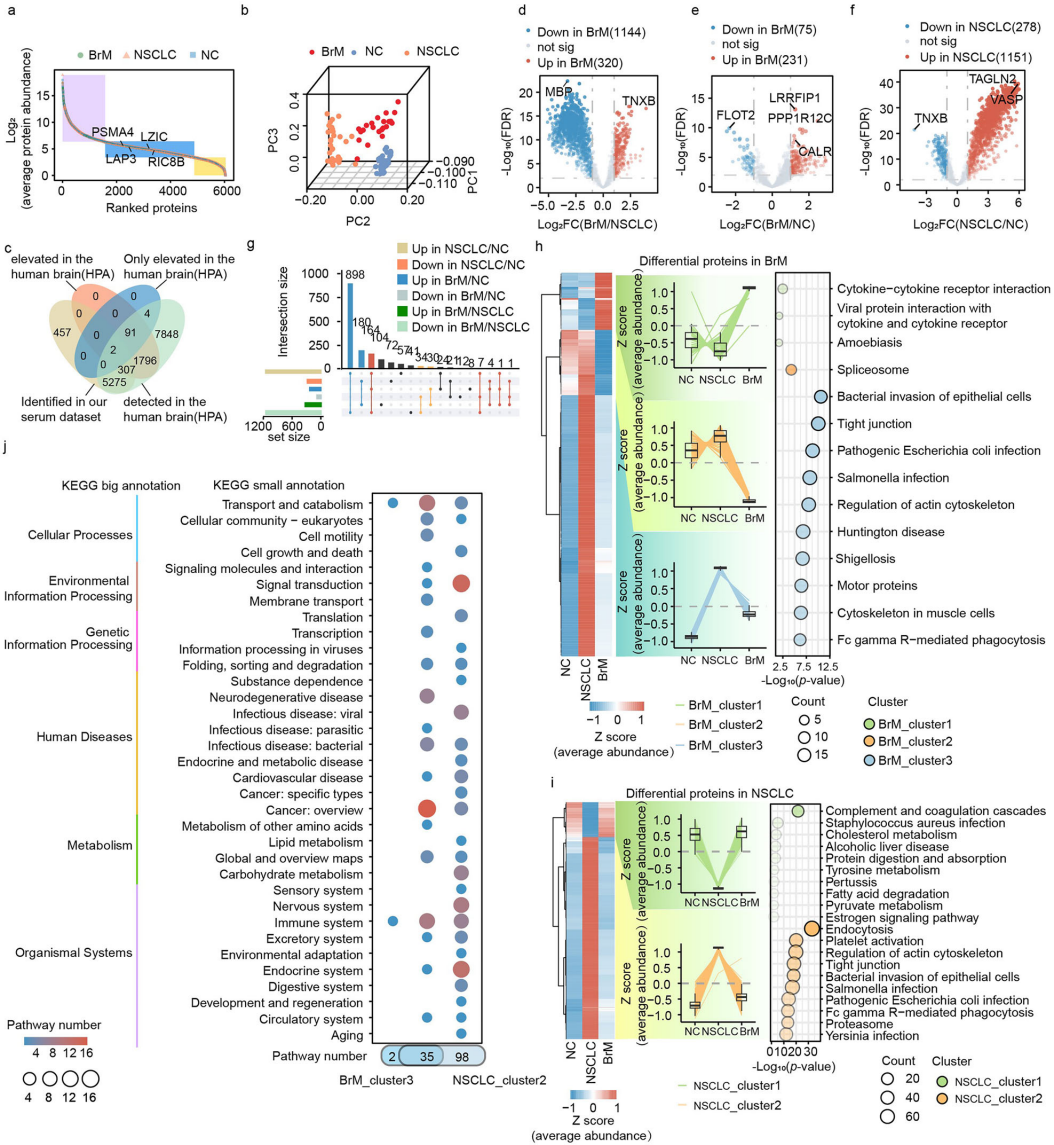
Serum proteomic profiling of BrM, NSCLC, and normal control (NC) samples. **a** Distribution of protein abundance across 6041 proteins in serum samples from BrM, NSCLC, and NC groups. The abundance range is stratified into high-, medium-, and low-abundance categories, as indicated by the shaded rectangles. **b** 3D PCA scatter plots showing the first three principal components derived from the serum proteomic data for the BrM, NSCLC, and NC groups. **c** Brain-related proteins identified in serum proteomics, annotated using the Human Protein Atlas (HPA). **d**–**f** Volcano plots showing differentially expressed proteins (DEPs) for each pairwise comparison (BrM vs. NSCLC, BrM vs. NC, and NSCLC vs. NC). DEPs were identified using Student’s *t*-test with FDR < 0.01 and log2 (foldchange) > 1 or < −1; significant proteins are highlighted in red (upregulated) or blue (downregulated). **g** UpSet plot showing shared and unique DEPs among the BrM vs. NSCLC, BrM vs. NC, and NSCLC vs. NC comparisons. K-means clustering and KEGG enrichment analysis of DEPs in BrM (**h**) and NSCLC (**i**). The left heatmap shows the mean abundance of DEPs across the three groups with K-means clustering, the middle box plot shows the average abundance of DEPs across groups, and the right panel shows the top 10 KEGG pathways ranked by *p-*value for each cluster. **j** KEGG pathway classification for proteins in BrM_cluster3 and NSCLC_cluster2.

We hypothesize that certain disease-associated proteins identified in tissue biopsies can also be detected in serum samples; however, the extremely wide dynamic range of protein abundance in serum, across approximately 10 orders of magnitude, with high-abundance proteins accounting for ~95%, creates substantial challenges for detecting low-abundance proteins. In practice, advanced non-targeted methods can directly identify only about 300–700 serum proteins without depletion of high-abundance proteins [26, 27], and as most of these proteins are either high-abundance species or intrinsic blood components, accurate quantification of disease-related proteins becomes difficult. Therefore, we applied our previously developed RSP-MOSF method [19] to increase the depth of identification in the serum proteome. Through rigorous QC procedures, we identified a total of 6041 serum proteins (Supplementary Fig. 2a, b), which represents, to our knowledge, the most comprehensive characterization of the serum proteome in NSCLC with BrM reported to date. Across all samples, the identified serum proteins spanned more than six orders of magnitude in signal intensity (Fig. 3a), and PCA based on all quantified proteins clearly separated the NSCLC, BrM, and NC groups (Fig. 3b). According to the Human Protein Atlas (HPA; https://www.proteinatlas.org/), among these proteins, 309 were elevated in the brain, 2 were exclusively elevated in the brain, and 5584 proteins were annotated as detected (Fig. 3c).

Differential abundance analysis of the serum proteome identified 1464 proteins with significant dysregulation (FDR-adjusted *p* < 0.05) when comparing the BrM and NSCLC groups. In addition, 1429 altered proteins were identified in the comparison between NSCLC and NC, and 306 DEPs were identified in the BrM versus NC comparison, resulting in 1658 unique proteins overall, which accounted for 27.45% of the quantified serum proteome (Fig. 3d-g; Supplementary Table 2).

To further examine how these DEPs behaved across groups, we performed K-means clustering. In the BrM group, we identified three distinct clusters of DEPs, whereas in the NSCLC group, we identified two clusters of altered proteins (Fig. 3h-i). Notably, BrM-associated proteins in BrM_cluster1 were mainly enriched in pathways involving cytokines (Fig. 3h, Supplementary Table 3). Previous studies demonstrated that cytokines and chemokines can promote transmigration across the blood-brain barrier, contribute to immunosuppression within the tumor microenvironment, and support colonization of metastatic cells in the brain parenchyma [28–30]. Altered proteins within BrM_cluster2 were involved in the spliceosome process. Studies have reported that STIP is a component of the nuclear spliceosome that may participate in intron removal from pre-mRNA and thereby influence gene expression regulation; importantly, STIP is also overexpressed in NSCLC tissues and has been reported to promote NSCLC cell proliferation [31]. The proteins in BrM_cluster3 showed the highest expression levels in NSCLC tissues, followed by BrM tissues, and the lowest levels in normal tissues, and they were mainly involved in bacterial invasion of epithelial cells and tight junction regulation. In NSCLC, these proteins may be induced as part of an antibacterial defense response to potential bacterial infection within the tumor microenvironment. Furthermore, they play a role in regulating tight junctions, which subsequently influences the invasive and metastatic capabilities of tumor cells. During the process of brain metastasizing, although there is a decrease in protein expression levels, these proteins may still retain some functional significance. These findings support that the identified DEPs play diverse and context-dependent roles in the initiation, progression, and metastatic spread of NSCLC.

In contrast, within the NSCLC group, proteins in NSCLC_cluster1 were mainly associated with cholesterol metabolism, tyrosine metabolism, pyruvate metabolism, and glycolysis/gluconeogenesis pathways, which reflects the metabolic adaptability of cancer cells across different environtments (Fig. 3i, Supplementary Table 3). Cancer cells can reprogram metabolic pathways to meet demands for energy, biosynthetic precursors, and redox power necessary for rapid proliferation, and targeting key metabolic pathways can inhibit tumor growth [32]; in BrM, given the distinct microenvironment, these metabolic pathways may be further enhanced to meet higher energy and biosynthetic requirements. Proteins in NSCLC_cluster2 were primarily involved in endocytosis, which may relate to the need for tumor cells to traverse the blood-brain barrier and adapt to a new microenvironment during NSCLC BrM, because endocytosis may support this process by facilitating uptake of essential nutrients and growth factors [33].

Intriguingly, BrM_cluster3 and NSCLC_cluster2 both showed a significant increase in protein levels during NSCLC progression, with higher levels in NSCLC, lower levels in BrM, and minimal levels in NC (Fig. 3j). This pattern suggests that the biological changes driving NSCLC development and metastatic spread may affect multiple pathways and signaling cascades, and that such changes may be more prominent in primary tumors, for example, because NSCLC patients often have a larger tumor burden and higher tumor metabolic activity. As a result, tumor cells may release substantial amounts of proteins and metabolic products into the bloodstream, leading to a significant increase in circulating protein levels. In addition, because NSCLC-BrM commonly occurs at an advanced stage, the patient’s overall metabolic state may already be altered, which could contribute to less pronounced protein level changes than those seen in early- or mid-stage NSCLC. Consistent with these observations, proteins in these two clusters were enriched in pathways related to cancer, immunity, and transport, while, in NSCLC specifically, there was also notable enrichment in pathways related to signal transduction.

### Integration of BrM tissue and serum proteomics

**Fig. 4:**
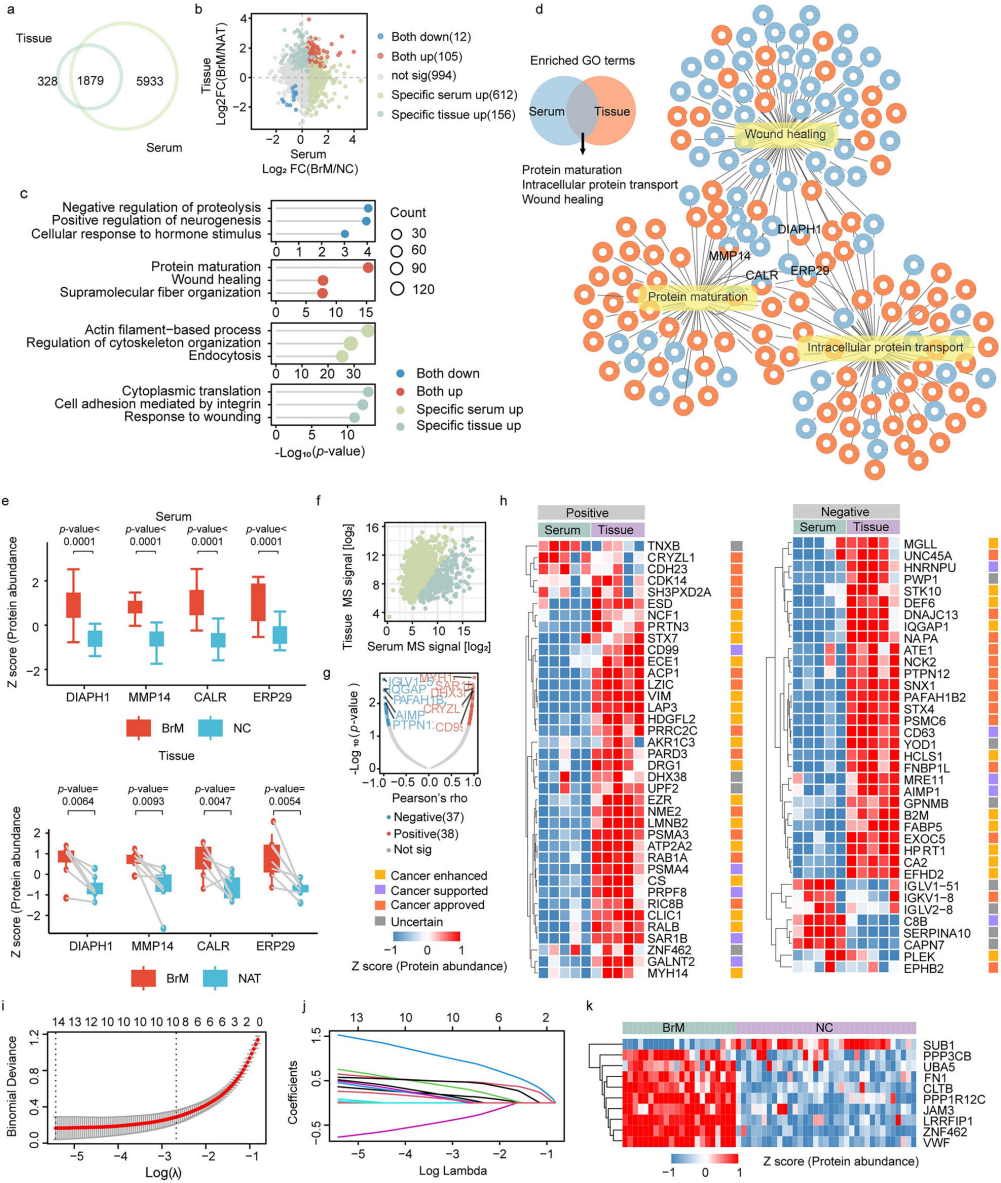
Integration of BrM-associated tissue and serum proteomes. **a** Venn diagram showing the overlap between serum and tissue proteomes in BrM. **b** Fold changes of proteins in serum and tissue for BrM versus NC. **c** Top 3 GO terms enriched among proteins specifically altered in BrM tissue and in BrM serum. **d** Venn diagram showing the overlap of GO gene sets enriched by differentially expressed proteins in serum and tissue. **e** Box plots showing the abundance of DIAPH1, MMP14, CALR, and ERP29 in serum (upper panel, *n* = 5) and tissue (lower panel, *n* = 5), comparing BrM versus NC (serum) and BrM versus NAT (tissue), respectively. **f** Pearson correlation between serum and tissue proteomes. **g** Correlation analysis of co-quantified proteins between tissue and serum. **h** Heatmap of proteins showing positive (left) and negative (right) correlations between serum and tissue, with cancer-related annotations from the Human Protein Atlas (HPA). Variable selection using LASSO regression. LASSO coefficient profiles of candidate variables (**i**) and cross-validation plot for selection of the optimal regularization parameter (**j**). **k** Heatmap showing the abundance of selected protein variables in BrM and NC groups.

Given that leakage of cancer-associated proteins into serum may influence its composition, we further compared the proteomic profiles of BrM tissue with those of serum. A total of 1879 proteins were co-quantified in both BrMtissue and BrMserum (Fig. 4a). Among these, 105 proteins were upregulated in both tissue and serum proteomes, whereas 12 proteins were downregulated in both (Fig. 4b). The upregulated proteins were mainly involved in protein maturation, wound healing, and supramolecular fiber organization (Fig. 4c, Supplementary Table 3). In addition, 612 proteins were specifically upregulated in serum samples, primarily involved in actin filament-based process, regulation of cytoskeleton organization and endocytosis. In comparison, 156 proteins showed specific upregulation in tissue samples, mainly associated with cytoplasmic translation, cell adhesion mediated by integrin and response to wounding (Fig. 4c). These proteins showed both consistent and inconsistent expression patterns between tissue and serum, highlighting that their biological roles may differ across compartments. We also examined the biological processes that may contribute to BrM progression by analyzing gene sets corresponding to dysregulated proteins in tissues and serum, and three. Three pathwaysprotein maturation, intracellular protein transport, and wound healing, were commonly enriched (Fig. 4d).

Notably, several key regulatory proteins, including matrix metalloproteinase-14 (MMP14), protein diaphanous homolog 1 (DIAPH1), endoplasmic reticulum resident protein 29 (ERP29), and calreticulin (CALR), were upregulated in both tissues and serum (Fig. 4e). As co-regulated proteins, they collectively participate in core processes driving BrM: MMP14 mediates extracellular matrix (ECM) degradation and cell invasion; DIAPH1 regulates cytoskeletal remodeling to facilitate cell migration; CALR and ERP29 coordinate protein folding and calcium homeostasis, which support tumor cell survival under stress.

More specifically, MMP14 is closely linked to a mesenchymal phenotype and is highly expressed in many sarcomas, where it regulates extracellular and plasma membrane proteins to influence cell-cell and cell-ECM interactions, thereby promoting ECM degradation, remodeling, cell invasion, and metastasis [34]. CALR knockdown has been reported to deplete breast cancer stem cells, which impaired tumor initiation and metastasis while increasing chemosensitivity in vivo [35]. In addition, ERp29 has been reported to function as an oncoprotein in colorectal cancer (CRC) by promoting CRC cell proliferation and metastasis while inhibiting apoptosis [36]. These results suggested that our identified proteins are consistent with previously reported data.

To prioritize the most informative candidate signals from our limited but valuable paired tissue and serum sample sets, we performed a hypothesis-generating correlation analysis between tissue and serum proteomes within the BrM group. We found that the distribution of co-quantified protein levels in two-dimensional space showed a bimodal pattern, in which one set of proteins displayed correlated relative abundance rankings in both tissue and serum (the “diagonal cluster”), whereas the other set did not show this correlation (the “vertical cluster”; each point represents an individual protein; Fig. 4f). In total, 75 proteins were identified as differentially abundant in both tissue and serum (Pearson’s rho, *p* < 0.05; Fig. 4g, Supplementary Table 6), and for clarity, we refer to these as co-regulated proteins; among them, 38 were positively correlated and 37 were negatively correlated. Notably, most of these DEPs showed the same direction of change in tissue and serum (i.e., they were both upregulated or both downregulated). Among the 38 positively correlated proteins, we further observed that several cancer-related proteins, including myosin-14 (MYH14), polypeptide N-acetylgalactosaminyl transferase 2 (GALNT2), and Ras-related protein Ral-B (RALB), showed significantly higher expression levels in tissue than in serum (Fig. 4h).

Overall, we aimed to identify molecules that can differentiate BrM from normal individuals by integrating tissue and serum proteomic data, and we focused on proteins showing significant positive correlations in both compartments as well as marked differences between BrM and NAT or NC. In addition, we applied LASSO regression to select ten key protein features (Fig. 4i-k), and this integrated strategy provides a complementary view for exploring molecular changes related to BrM from both tissue and circulating proteomes.

### Machine learning models to detect BrM patients from NSCLC patients

In clinical practice, plasma or serum is the most commonly utilized specimen for biomarker discovery, because circulating proteins may reflect the pathophysiology of different diseases. Our dataset, therefore, provides an opportunity to distinguish BrM from NSCLC. We performed a comparative analysis of serum proteomic profiles associated with BrM and those derived from BrM tissue samples, and, overall, we observed a significant positive correlation between the proteomic profiles of BrM tissues and serum (Fig. 4f-g).

**Fig. 5:**
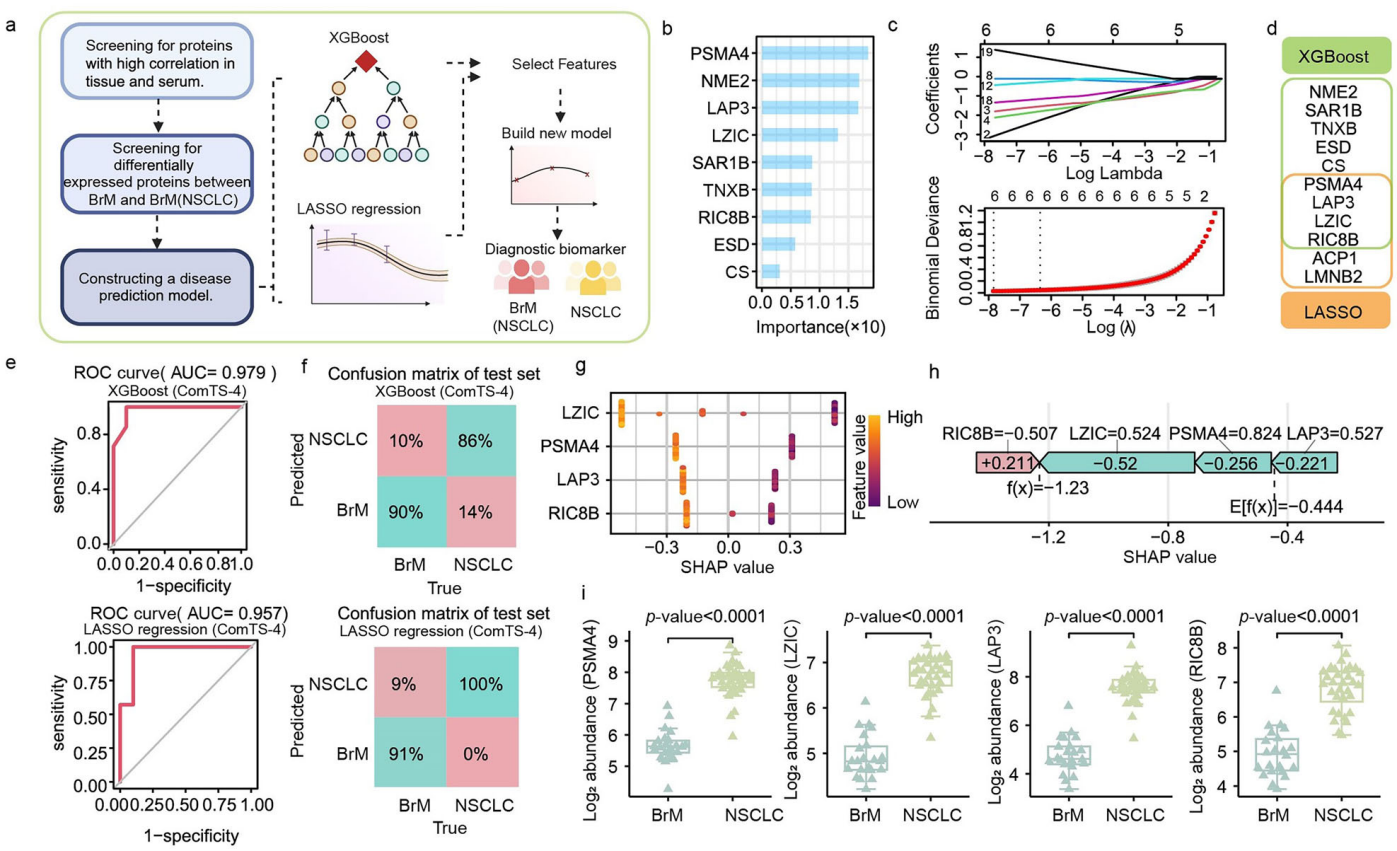
Predictive models for distinguishing BrM from NSCLC. **a** Schematic overview of the protein screening workflow used to develop predictive models. **b** Bar plot showing the importance scores of proteins identified by XGBoost when comparing BrM and NSCLC in the serum proteome. **c** Variable selection using LASSO regression. Top panel: LASSO coefficient profile for candidate variables. Bottom panel: cross-validation plot used to determine the optimal regularization parameter. **d** Venn diagram showing the intersection of variables selected by XGBoost and LASSO regression. **e** ROC curves showing the performance of the XGBoost model ([PSMA4, LAP3, LZIC, RIC8B], upper panel) and the LASSO model ([PSMA4, LAP3, LZIC, RIC8B], lower panel) in distinguishing NSCLC from BrM. **f** Confusion matrices showing classification accuracy of the XGBoost model (upper panel) and LASSO model (lower panel) for BrM versus NSCLC in the test set. **g** SHAP summary plot for the four key proteins (PSMA4, LAP3, LZIC, RIC8B). Each point represents the effect of a feature for each sample, ordered by feature importance and colored by feature value; higher SHAP values indicate stronger contribution to the prediction. **h** SHAP force plot showing the contribution of each feature to the model’s prediction, with features increasing prediction values shown in pink and those decreasing prediction values shown in green. **i** Box plots showing the abundance of PSMA4, LAP3, LZIC, and RIC8B in BrM patients (*n* = 22) versus NSCLC patients (*n* = 32).

Based on these findings, we developed a screening workflow to build models and identify biomarker combinations that can effectively classify BrM and NSCLC (Fig. 5a). First, to ensure that serum protein levels reflect tissue conditions, we screened proteins showing a significant positive correlation between their abundance in tissue and serum (Pearson’s rho > 0.85; *p*-value < 0.05). Among these proteins, 27 were differentially expressed in the serum proteomics of BrM compared with NSCLC and were taken forward for model development. Using XGBoost to construct the diagnostic model identified nine important variables (Fig. 5b), whereas the LASSO model selected six key variables (Fig. 5c). By taking the intersection of these two variable sets, we obtained a compact biomarker combination of four proteins (ComTS-4): PSMA4, LAP3, LZIC, and RIC8B (Fig. 5d). To strengthen machine-learning-based classification of BrM, we evaluated models combining four proteins using both XGBoost and LASSO, and, in the validation cohort, XGBoost (AUC = 0.979) performed better than LASSO (AUC = 0.957) (Fig. 5e).

To further evaluate model performance in distinguishing BrM from NSCLC, we added additional validation metrics. ROC curve analysis of the optimal model showed a very high AUC (0.996, 95% CI: 0.987–1.000) (Supplementary Fig. 3a), supporting strong discriminative ability, while the individual prediction error distribution yielded a mean Brier score of 0.1389 (Supplementary Fig. 3b), indicating good calibration. The model also performed well across key metrics, including sensitivity (Se = 0.955, 95% CI: 0.85–1), specificity (Sp = 0.969, 95% CI: 0.893–1), positive predictive value (PPV = 0.955, 95% CI: 0.846–1), and negative predictive value (NPV = 0.969, 95% CI: 0.903–1) (Supplementary Fig. 3c). To assess model reliability and rule out random performance, we conducted a permutation test, and the shuffled labels produced an average AUC of 0.575 (Supplementary Fig. 3d), supporting that the model’s performance was not due to random chance. Taken together with the validation-cohort results for XGBoost (AUC = 0.979) and LASSO (AUC = 0.957) (Fig. 5e), these findings support the high accuracy of our model for classifying BrM versus NSCLC. We also generated confusion matrices to assess the reliability of the machine-learning strategy, and these results showed that samples could be classified with high precision (Fig. 5f). SHapley Additive exPlanations (SHAP) was then used to examine the contributions of the four variables in the predictive model. Among these variables, LZIC had the greatest impact on prediction, followed by PSMA4, LAP3, and RIC8B (Fig. 5g), and RIC8B showed a positive SHAP value that increased the predicted probability of BrM (Fig. 5h). In addition, the abundances of these four variables were lower in BrM than in NSCLC (Fig. 5i).

Overall, our data support a four-protein classifier as a promising approach to distinguish NSCLC from BrM; however, further validation in an independent prospective cohort will be necessary to confirm the reliability and generalizability of these classifiers.

### PSMA4, LAP3, and LZIC were identified as diagnostic biomarkers for NSCLC BrM

**Fig. 6:**
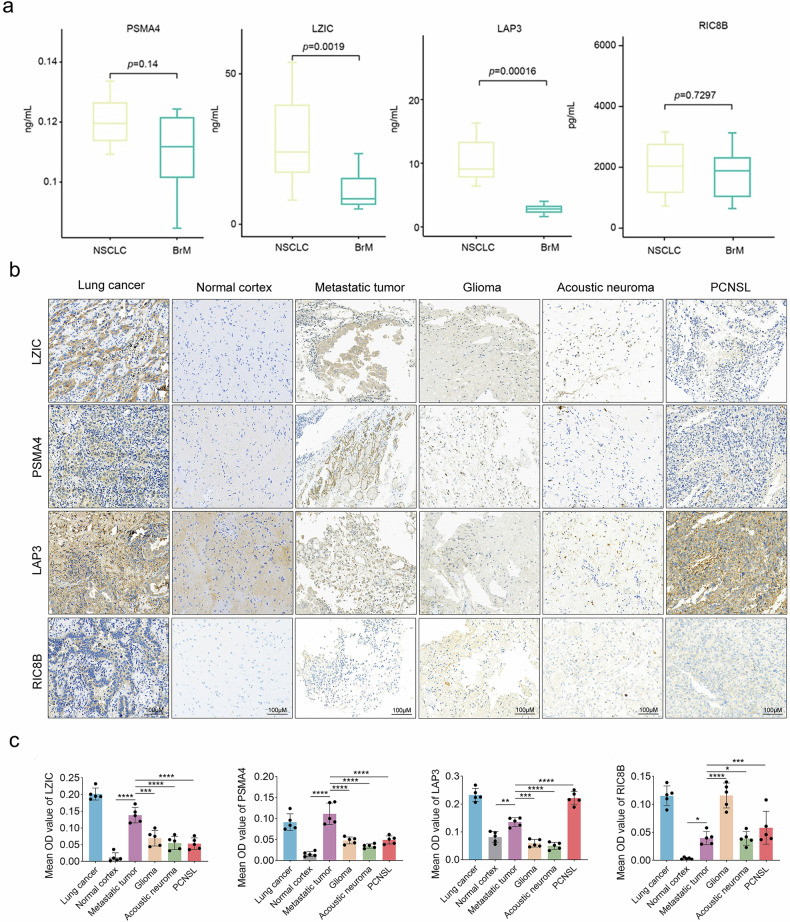
PSMA4, LAP3, and LZIC identified as diagnostic biomarkers for NSCLC-BrM. **a** ELISA validation showing the serum concentrations of PSMA4, LZIC, LAP3, and RIC8B in the study cohorts. **b** Immunohistochemical staining of LZIC, PSMA4, LAP3, and RIC8B in NSCLC-BrM, primary lung cancer, and primary brain tumor tissues. **c** Mean OD values of IHC of LZIC, PSMA4, LAP3, and RIC8B in NSCLC-BrM, primary lung cancer, and primary brain tumor tissues. (*n* = 5). All data are shown as mean ± SD. Statistics of (**a**) were calculated using Student’s *t* test and (**c**) were calculated using one-way ANOVA tests. NS not significant, *p < 0.05, **p < 0.01, ***p <0. 0.001, ****p < 0.0001. Scale bars represent 100 μm in (**b**).

To assess the specificity of PSMA4, LAP3, LZIC, and RIC8B for NSCLC-BrM, we measured their plasma concentrations by ELISA in independent cohorts. Both LZIC (*p* = 0.0019) and LAP3 (*p* = 0.00016) were significantly decreased in the plasma of NSCLC-BrM patients. Although PSMA4 was not markedly reduced overall, its level was still lower in NSCLC-BrM than in NSCLC. In contrast, no significant difference in RIC8B expression was observed between NSCLC and NSCLC-BrM patients (Fig. 6a). These results suggest that circulating LZIC, PSMA4 and LAP3 may serve as potential biomarkers to distinguish NSCLC-BrM from LC patients without BrM.

Further, we examined the expression of PSMA4, LAP3, LZIC, and RIC8B by IHC in BrM tissues from five NSCLC-BrM patients and in primary LC tissues from five patients. LZIC, PSMA4 and RIC8B staining were mainly localized to the cytoplasm of tumor cells, whereas LAP3 staining was observed not only in the cytoplasm of tumor cells but also in interstitial cells. In a relatively larger proportion of cases, LZIC, LAP3, and RIC8B showed lower expression in brain metastases than in the corresponding primary lung lesions (Fig. 6b).

Next, we examined tissue specimens of 5 patients without NSCLC-BrM and 5 glioma tissues, schwannoma tissues and primary central nervous system lymphoma (PCNSL) tissues as controls. In general, those patients showed higher PSMA4, LAP3 and LZIC expression in brain metastases than that in the other lesion. The expression in BrM tissue was significantly higher than that in the controls (*p* < 0.01), and LZIC was almost absent in normal cortex, while it was weakly expressed in glioma tissue, schwannoma, and PCNSL tissues. PSMA4 was also expressed in a proportion of BrM tissues but was not commonly expressed in other primary brain tumors (*p* < 0.01). In comparison, LAP3 was not exclusively expressed in BrM tissue, because its expression in BrM was higher than that in glioma and schwannoma tissues but lower than that in PCNSL tissue. By contrast, RIC8B showed strong expression in glioma tissue but weak expression in BrM, schwannoma, and PCNSL tissues (Fig. 6b, c). Collectively, these findings indicate that LZIC and PSMA4 may serve as potential histological markers for NSCLC BrM and may help distinguish BrM from primary intracranial tumors.

## Discussion

Approximately 25–40% of patients with NSCLC develop BrM during disease progression, and this complication has a major adverse impact on both quality of life and survival. For this reason, reliable tools that can identify patients at increased risk of BrM are needed, because earlier risk recognition may allow timely prophylactic or early therapeutic strategies that reduce morbidity and mortality. However, non-invasive biomarkers that can support early diagnosis of NSCLC-BrM remain insufficiently validated for routine clinical use. At present, the diagnosis of LC brain metastases relies mainly on contrast-enhanced brain MRI together with postoperative histopathological confirmation when tissue is available. In patients with advanced LC, periodic cranial MRI surveillance is important for detecting intracranial metastatic lesions at an earlier stage; nevertheless, the cost of MRI limits its practicality for long-term routine monitoring in many settings, and contrast-enhanced MRI may not be suitable for patients with contrast-agent allergy, renal insufficiency, or critical illness. In addition, MRI commonly identifies metastases once lesions have become established and radiographically apparent, which means that, for some patients, detection may still occur after the most favorable window for intervention has narrowed. Although histopathological examination remains the diagnostic gold standard, its invasive nature and its inability to provide dynamic, longitudinal information restrict its broader clinical applicability.

Therefore, there is a clear need for diagnostic approaches for LC brain metastases that are highly sensitive, non-invasive, reproducible, and suitable for repeated assessment over time. In recent years, liquid biopsy technologies have emerged as an important option for monitoring tumor progression, largely because they are minimally invasive and can be performed serially with good analytical reproducibility. In this study, we aimed to characterize tissue and serum proteome changes associated with NSCLC-BrM pathophysiology and, based on these data, to identify circulating proteins with potential diagnostic value for distinguishing BrM from NSCLC without BrM.

This study reports several promising findings. First, our study provides a high-depth tissue and serum proteomic dataset in NSCLC with BrM, with 12,808 proteins identified in tissue proteomics and 6041 proteins identified in serum proteomics. Second, the proteomic patterns observed in both BrM tissue and serum highlight key biological processes involved in BrM, including protein maturation, intracellular protein transport, and wound healing, and proteins within these pathways showed pronounced changes in abundance.

Moreover, tissue proteomics showed that proteins downregulated in BrM were predominantly related to neural regulation, which suggests that metastatic tumor growth within the brain may interfere with normal neural regulatory functions. Moreover, reduced expression of these proteins may reflect broader alterations in neurobiological processes involved in the brain’s response to various stimuli, including the infiltration and growth of metastatic cancer cells. Such perturbations could affect neurotransmission, synaptic plasticity, and neuromodulatory signaling, and these changes may create conditions that support tumor cell adaptation and proliferation within the brain.

Proteins in serum are derived from various sources. Solid tissues, particularly the liver, brain, and intestine, secrete a large number of proteins that enter the circulation and exert biological functions in serum. In this study, many of the proteins identified in the serum proteome were brain-associated, and these intracellular proteins may serve as signature markers that reflect tissue injury related to tumor formation. In addition, our results show that the differential proteins among BrM, NSCLC, and NC were mainly related to transport, cancer, and immune functions. Notably, proteins that were significantly upregulated in BrM were primarily enriched in the cytokine-cytokine receptor interaction pathway, and this pathway may be particularly relevant to BrM because it can influence metastatic behavior, tumor-immune interactions, and the overall tumor microenvironment.

Developing statistical models to predict the risk of NSCLC-BrM has gradually become a focus of translational and clinical research, because such models could assist clinicians in designing more rational preventive strategies. Several studies have attempted to establish BrM prediction models for NSCLC based on clinical variables, including age, histology, smoking status, and pT stage [37]. However, these models often show limited specificity and sensitivity because they rely mainly on clinical variables and therefore lack biological specificity for metastatic progression, with reported performance commonly below an AUC of 0.75 in many studies. Grinberg-Rashi reported that mRNA expression of N-cadherin, KIFC1, and FALZ1 in NSCLC specimens was associated with tumor metastasis [38], yet tissue-based mRNA data from NSCLC specimens cannot dynamically reflect changes in BrM risk throughout disease progression. In addition, other predictive models developed using single-omics data (for example, mRNA expression) also have important limitations, because mRNA levels do not always correlate with protein abundance, and tissue-only models do not capture circulating biomarkers that can support non-invasive monitoring. Wei et al. reported that cathepsin F (CTSF) and fibulin-1 (FBLN1) were specifically upregulated in both sera and tissues of patients with NSCLC BrM compared with NSCLC without BrM and primary brain tumors, and the combined diagnostic performance of CTSF and FBLN1 was superior to either marker alone [16]. In other studies, S100A7 expression has also been reported to be closely associated with BrM and may serve as a potential monitoring biomarker [39]. Although many studies have explored biomarkers related to NSCLC-BrM, most have focused on one marker or a small set of candidates, and, importantly, prior studies have rarely integrated paired tissue and serum proteomics to develop predictive models for NSCLC-BrM, leaving limited direct “head-to-head” comparison benchmarks.

Against this background, our integrated models using XGBoost and LASSO show several advantages. Notably, the main innovation of our study is not limited to predictive performance, because (1) we provide paired tissue-serum proteomic resources that offer complementary biological insights; (2) we applied a multi-step validation strategy, including cross-validation, technical replication, and biological validation of candidate biomarkers, to improve robustness; (3) the identified ComTS-4 panel has biological interpretability, with links to cytokine signaling, neural regulation, and cell cycle pathways; and (4) the compact four-protein signature supports practical clinical use in non-invasive testing.

In our internal comparative analysis, we further clarified the complementary strengths of XGBoost and LASSO. XGBoost showed stronger predictive performance than LASSO, largely because it can model nonlinear relationships and higher-order interactions between tissue and serum proteins, which is important for capturing the complex pathological processes associated with BrM. In contrast, LASSO produced a more compact model that improves interpretability and may reduce the costs associated with clinical assay development. Taken together, this dual-model strategy helps address different research and clinical needs, with XGBoost being particularly suitable when the primary goal is maximal predictive accuracy for stratifying high-risk patients.

Notably, previous proteomic studies in BrM and NSCLC have suggested that LAP3 overexpression is closely associated with cell growth, migration, and invasion in multiple cancers, including glioma [40], and this observation was also supported by our dataset. In breast cancer, LAP3 has been reported to promote migration and invasion by increasing fascin expression through the p38-Hsp27-NF-κB pathway and by enhancing MMP-2/9 production via the PI3K-Akt signaling axis [41]. Moreover, LAP3 can promote epithelial-mesenchymal transition (EMT) by upregulating vimentin through the MEK/Erk1/2 pathway, thereby increasing metastatic potential [42]. Together, these findings provide mechanistic support for the relevance of LAP3-related pathways to metastasis. In our study, LAP3 was downregulated in NSCLC-BrM, suggesting that dysregulation of LAP3-associated pathways may be involved in the multi-step process of BrM.

LZIC has also been implicated in cell cycle regulation and DNA damage response, and its downregulation may contribute to genomic instability and facilitate metastatic adaptation under stress conditions such as ionizing radiation. Consistent with this, another study reported a significant decrease in LZIC and suggested that it may serve as a tumor marker associated with cell proliferation, invasion, and metastasis [43]. In addition, LZIC is critical for regulation of the G2/M checkpoint during DNA damage responses, particularly under ionizing radiation, and loss of LZIC can disrupt this checkpoint, leading to genomic instability and potentially promoting tumor progression and metastasis [44]. In line with these reports, our dataset showed that LZIC was significantly downregulated in the BrM/NSCLC group. Regarding PSMA4, several studies have reported associations with metastasis across different cancers. For example, in lung adenocarcinoma (LUAD), PSMA4 overexpression has been reported to potentially activate WNT signaling through effects on AXIN, thereby promoting tumor progression [45]. In metastatic gastric cancer (mGC), PSMA4 has also been identified as a highly significant protein in proteomic analyses of mGC-derived exosomes [46]. By contrast, definitive evidence for a direct role of RIC8B in tumor metastasis remains limited; nevertheless, RIC8B participates in several cellular processes, including G protein signaling. Specifically, RIC8B has been described as a guanine nucleotide exchange factor (GEF) for G protein α subunits, facilitating GTP loading and thereby activating G protein signaling [47]. Since G protein signaling is involved in key cellular behaviors such as proliferation, differentiation, and migration, it is biologically plausible that RIC8B-related signaling could be relevant to metastatic processes [48].

Our study has several limitations. First, cohort size was largely determined by our exclusion criteria, particularly the restriction to patients without prior treatments, and the serum-tissue correlation analysis was further constrained by the availability of paired specimens. These factors support the need for validation in larger, dedicated cohorts to improve generalizability. Second, by focusing on treatment-naive or surgically resected NSCLC patients, we established a clearer baseline for proteomic comparison; however, future studies that evaluate the influence of different therapeutic strategies will be important for interpreting these findings in broader and more heterogeneous clinical settings. Third, although the identified biomarker proteins show potential relevance to the pathogenesis of LC-derived BrM, additional functional validation is required to confirm their biological roles and to strengthen mechanistic understanding.

Although the machine learning model developed in this study showed high sensitivity and specificity in initial validation, its real-world diagnostic performance still requires confirmation in large-scale, prospective, multicenter clinical studies. Such validation is a key step for biomarker translation, yet it is challenging because it is time-consuming and resource-intensive. In parallel, standardization of the detection workflow remains a major barrier to clinical implementation. To achieve high reproducibility across laboratories, experimental protocols, reagents, instrumentation, and data analysis pipelines must be carefully harmonized, and this process is technically demanding. In addition, the biomarkers themselves introduce biological variability, because expression levels can be influenced by disease stage, therapeutic interventions, circadian rhythms, and even individual diet and exercise, which complicates the establishment of stable and widely applicable reference ranges. In summary, translating the blood-based biomarkers identified in this study into clinical practice will require rigorous evaluation across multiple dimensions, including data robustness, technical feasibility, and clinical and ethical compliance. Ultimately, only biomarkers that consistently demonstrate reliable measurements, clear clinical validity, and strong diagnostic performance will be positioned to move from bench to bedside and provide meaningful benefit for patients.

In conclusion, we established an integrated pipeline to discover protein candidate biomarkers associated with NSCLC-BrM, and this strategy achieved substantial depth in both tissue and serum proteomic profiling using our newly developed RSP-MOSF method. Our study provides a useful proteomic resource that may help researchers better understand biological responses linked to BrM, while also offering insights into mechanisms related to BrM formation and identifying a set of candidate biomarkers that could support clinical decision-making. Taken together, these findings may contribute to improving diagnostic accuracy and supporting more effective treatment strategies for patients with BrM.

## Materials and methods

### Study design and participants

The study was conducted following the Declaration of Helsinki and the Department of Health and Human Services Belmont Report, and it was approved by the Research Ethics Committee of Ruijin Hospital, Shanghai Jiao Tong University. Written informed consent was obtained from all patients or their legal representatives before participation. All patients enrolled in this study had histologically confirmed NSCLC or NSCLC-BrM and were treated at either Shanghai Pulmonary Hospital or Ruijin Hospital between January 2021 and October 2023. The Karnofsky performance status (KPS) score of all patients was at least 70, and all had a life expectancy of at least 6 months. Epidemiological data were collected using a structured questionnaire and were supplemented by medical record review, including demographics, smoking history, medical history, treatment regimens, and disease stage. Cancer tissue and matched normal adjacent tissue were surgically obtained from consecutive patients with NSCLC-BrM who underwent tumor resection at Ruijin Hospital.

Patients with NSCLC confirmed by postoperative pathological examination were enrolled in the NSCLC group if they had no history of other systemic malignant tumors and had no intracranial metastases detected by head MRI within six months after enrollment. Moreover, patients with intracranial lesions confirmed by postoperative pathology as metastases from NSCLC were enrolled in the NSCLC-BrM group if they had no history of other systemic malignant tumors. Patients with acute infections, diabetes, systemic inflammatory diseases, psychiatric conditions, recent surgical intervention, a previous or secondary malignancy, and/or a history of chemotherapy, radiotherapy, other anti-tumor therapy, severe coagulopathy, or other central nervous system diseases were excluded. NSCLC tumors were staged according to the 7th edition of the Union for International Cancer Control (UICC) staging system, and the clinical characteristics of the cases are summarized in Supplementary Table 1.

A total of 14 resected tissue specimens were collected from seven Chinese patients diagnosed with NSCLC-BrM, and 89 serum samples were collected from patients diagnosed with NSCLC (*n* = 32), BrM (*n* = 22), and matched normal controls (NC, *n* = 35). The diagnoses of NSCLC and BrM were confirmed by postoperative pathology. The healthy control group consisted of outpatients without cancer who underwent routine physical examinations. Notably, all patients with either NSCLC-BrM or NSCLC alone had not received surgical intervention or anti-tumor therapy at baseline.

The collected brain tumor tissues were obtained after surgery and washed three times with phosphate-buffered saline (PBS); the samples were then placed into 2 mL cryogenic storage vials (Corning, New York, USA) and promptly transferred to liquid nitrogen for proteomic analysis, or immediately fixed in 4% paraformaldehyde for immunohistochemical staining (IHC). Subsequently, tissues for proteomic analysis were stored at −80 °C. Serum samples were collected before surgical resection using intravenous tubes without anticoagulants, allowed to clot at room temperature for 30 minutes, and then centrifuged at 1500 × g for 10 minutes at 4 °C, after which the serum was stored at −80 °C. Each aliquot was used only once to avoid freeze-thaw cycles. IHC samples with >50% necrotic area, severe hemorrhage, or tissue fragmentation that prevented cellular evaluation were excluded from quantitative analysis.

### Sample preparation of DIA-MS-based proteomics

The preparation of serum samples involved several steps, including low-abundance protein enrichment, protein denaturation, reduction, alkylation, digestion, peptide cleanup, and a key quality control (QC) step for determining peptide concentration. Initially, 1 mg of nanoparticle magnetic beads was diluted in 100 µL of wash buffer (LABP magnetic bead, OSFP0002, Shanghai Omicsolution Co., Ltd., China), after which 100 µL of serum was added and incubated at 37 °C with shaking at 1000 rpm for 1 hour. After incubation, the sample was placed on a magnetic rack, and the supernatant was removed and discarded by magnetic separation. At this stage, low-abundance proteins were adsorbed onto the magnetic beads, which were then washed three times with wash buffer. The magnetic beads were subsequently resuspended in 40 µL of lysis buffer (buffer A) and heated with stirring at 95 °C and 1000 rpm for 5 minutes. After cooling to room temperature, Lys-C, trypsin, and buffer B were added, and the sample was incubated at 37 °C for 2 hours with shaking at 500 rpm. Digestion was stopped by adding buffer C, and the peptide-containing supernatant was collected by centrifugation at 20,000 g for 10 minutes, after which peptides were desalted using a C18 desalting column. Peptides were eluted twice with 30 µL of elution buffer, dried, and redissolved in buffer A (0.1% formic acid [FA]). Before MS analysis, a QC step was performed in which peptide concentrations were measured using a NanoDrop to ensure consistent sample loading across all serum samples, thereby minimizing technical variation during MS detection.

To increase the reproducibility of sample handling for high-quality data generation, tissue samples were prepared using a newly developed automated sample preparation platform (EasyPept Auto100, Shanghai Omicsolution Co., Ltd., China). In brief, tissue samples were lysed with a lysis buffer [100 µL of 1% sodium deoxycholate (SDC), 10 mM tris-(2-carboxyethyl)-phosphine (TCEP), 20 mM chloroacetamide (CAA), 0.1% RapiGest surfactant, and 1× protease/phosphatase inhibitor in 50 mM ammonium bicarbonate (ABC)] in a 95 °C heat module for 60 minutes. Following the lysis process, ultrasonication was performed for 10 cycles (30 seconds ON/OFF) to disrupt the tissue structures further. The lysed samples were subsequently transferred to a 96-well plate and digested in a heat module maintained at 37 °C, with agitation at 900 rpm for 2 hours. The digestion reaction was quenched by adding 2% trifluoroacetic acid (TFA), followed by incubation at 37 °C for 30 minutes. The mixture was centrifuged at 13,000 × g for 10 minutes to remove precipitates, and the supernatant was desalted using a DesaltingTip (Shanghai Omicsolution, OSFP0200-Y).

### LC-MS/MS in data-independent acquisition mode

For serum proteomics, DIA-based LC-MS/MS acquisition was performed on a NanoElute 2 liquid chromatograph (Bruker Daltonik, Bremen, Germany) coupled to a timsTOF HT mass spectrometer (Bruker Daltonik, Bremen, Germany) equipped with PASEF technology (Bruker Daltonics, Bremen, Germany) and an AUR3-15075C18 column (75 μm × 15 cm, 1.7 μm, 120 Å; IonOpticks). Peptides were separated at a flow rate of 300 nL/min using mobile phase A (0.1% FA in water) and mobile phase B (0.1% FA in 80% acetonitrile), with the following gradient: 0–15 min, 4–28% B; 15–16.5 min, 28–90% B; and 16.5–20 min, 90% B.

The timsTOF parameters were set as follows: capillary voltage, 1600 V (42 nA); dry gas, 30 L/min; and dry temperature, 180 °C. The MS was operated in diaPASEF mode with 24 × 25 Th precursor isolation windows covering an m/z range of 400–1000. To optimize the MS1 cycle time, three steps were repeated within an eight-scan diaPASEF sequence. During PASEF MS/MS scanning, collision energy was linearly ramped from 59 eV at 1/K0 = 1.6 Vs/cm^2^ to 20 eV at 1/K0 = 0.6 Vs/cm^2^.

For tissue proteomics, DIA LC-MS/MS analysis was performed as described in a previous study [49].

### Database search

The DIA files were searched against the human Swiss-Prot database (20,596 entries) using Spectronaut 17 (Biognosys AG, Switzerland) with the default settings for directDIA analysis, while retention time prediction was set to dynamic iRT and data extraction was performed using extensive mass calibration in Spectronaut 17. The extraction window was adjusted based on iRT calibration and gradient stability, and FDR cutoffs were set at 1% at both the precursor and protein levels. Decoy generation used a mutation approach similar to a scrambled strategy, but implemented random amino-acid position swaps (minimum = 2; maximum = length/2). Local normalization was applied, and peptides passing the 1% FDR cutoff were used to calculate major group quantities using the MaxLFQ method. Carbamidomethylation of cysteine was specified as a fixed modification, whereas oxidation of methionine and protein N-terminal acetylation were specified as variable modifications.

### Statistical analysis

Label-free quantified (LFQ) data were normalized by sum intensity, filtered to retain proteins with valid intensity values in at least 50% of samples, imputed using a random forest approach (R package “missforest”), and then log_2_ transformed. Differential protein expression between groups was assessed using Student’s *t*-test, with thresholds defined as FDR (Benjamini-Hochberg) < 0.01 and log2 fold-change > 1 or < −1 for serum proteomics, and *p*-value < 0.05 with log2 fold-change > 1 or < −1 for tissue proteomics. Principal component analysis (PCA) was performed in R using “FactoMineR” and “factoextra”. Z-scores were used to generate a K-means clustered heatmap with “pheatmap” in R (clustering_method = “ward.D”; nstart = 24). The correlation between serum and tissue proteomics was evaluated using pairwise Pearson correlation coefficients utilizing the corr.test() function in the R package “psych”, and *p*-values from the pairwise correlation tests were corrected using the Benjamini-Hochberg false discovery rate (FDR) method.

The Annotation and Integrated Discovery (DAVID, https://david.ncifcrf.gov/; Metascape, https://www.metascape.org) were used for GO Biological Processes (GOBP) enrichment analysis. The Kyoto Encyclopedia of Genes and Genomes (KEGG, https://www.kegg.jp/kegg/mapper/) analysis was used to enrich functional pathways. Pathways exhibiting a significance level with a *p*-value < 0.05 were defined as significantly regulated. Visualization for this study was predominantly facilitated using R software (version 4.4.1).

For IHC analysis, the mean optical density (OD) was quantified using ImageJ. Briefly, images were imported into ImageJ and separated into DAB and nuclear channels, after which the DAB channel was converted to OD values; thresholds were then applied to identify positively stained areas, and the mean OD was calculated for each image. Analyses were independently performed by two observers who were blinded to group allocation, and the Pearson correlation coefficient (r) was calculated to assess inter-observer agreement. Sample coding and decoding were performed by an independent researcher who was not involved in data analysis; the Pearson r was 0.9633, and measurements from one observer were used for subsequent quantitative analyses. The variance similar between the groups that are being statistically compared. Statistical analysis was performed using one-way ANOVA followed by Tukey’s post hoc test.

### Screening potential drug targets of BrM and NSCLC

The following two criteria were applied to identify potential drug targets for patients with BrM or NSCLC: (1) candidates had to be upregulated proteins or proteins specifically associated with BrM (or NSCLC), and (2) candidates had to be annotated as potential drug targets in the Drug Gene Interaction Database (DGIdb).

### Machine learning strategy

The machine learning workflow was designed to integrate multi-step validation, including serum-tissue correlation screening and algorithmic feature selection, with model construction, and this design was intended to ensure that the final biomarker panel is both biologically meaningful and statistically robust, as illustrated in Fig. 5a. The process included the following sequential steps.

- Sample partitioning: Serum samples from patients with NSCLC and BrM (*n* = 54 total: NSCLC, *n* = 32; BrM, *n* = 22) were randomly divided into a training set (70%, *n* = 37) and a test set (30%, *n* = 17), and stratified sampling was used to maintain the NSCLC:BrM ratio in both subsets.
- Preliminary feature filtering via serum-tissue correlation: To ensure that serum proteins selected as features reflect tumor-related changes, we first filtered for proteins showing a significant positive correlation between serum and tissue abundance (*p*-value < 0.05), which excluded serum proteins whose expression was not aligned with tissue abundance and narrowed the candidate feature pool to 27 DEPs.
- XGBoost model training and feature importance ranking: Using the R packages “caret” and “xgboost”, we tuned hyperparameters by five-fold cross-validation in the training set, after which the trained XGBoost model calculated feature importance scores based on each protein’s contribution to classification performance and identified nine high-impact variables.
- LASSO regression for feature selection: We applied LASSO regression using the R packages “glmnet” and “cv.glmnet”, again using five-fold cross-validation on the same training set, and the LASSO penalty shrank coefficients of less informative features toward zero, resulting in six key variables that minimized prediction error.
- Biomarker panel refinement and model validation: The overlap between the features identified by XGBoost and LASSO was used to derive a compact four-protein panel comprising proteasome subunit alpha type-4 (PSMA4), cytosol aminopeptidase (LAP3), protein LZIC (LZIC), and Synembryn-B (RIC8B), and this integration was intended to leverage complementary strengths of the two algorithms. The performance of the panel was validated in the test set using receiver operating characteristic (ROC) curves and confusion matrices, with BrM defined as the positive class. Additionally, SHAP analysis (R package “shapviz”) was performed to quantify the contribution of each protein to model predictions and to support interpretability of the final classifier.
- Nested cross-validation and statistical validation: The final model based on the refined 4-protein panel was further evaluated using nested cross-validation (comprising 3 outer folds and 5 inner folds) to assess generalizability, and performance metrics were quantified by calculating the mean area under the curve (AUC), with 95% confidence intervals estimated by bootstrapping. Moreover, model significance was further assessed using a permutation test.

### Quantification of PSMA4, LAP3, LZIC, and RIC8B by Enzyme-Linked Immunosorbent Assay (ELISA)

Serum concentrations of PSMA4, LAP3, and LZIC were measured using colorimetric ELISA kits (EIAab; catalog numbers E4783h, E12482h, and E0536h, respectively), while RIC8B was quantified using an ELISA kit from Ruixin Biotech (catalog number RX101130H), following the manufacturer’s instructions. Before analysis, serum samples for PSMA4, LAP3, and LZIC were centrifuged at 12,000 × g for 10 minutes at 4 °C to obtain supernatants, while serum samples analyzed for RIC8B were centrifuged at 4000 rpm for 20 minutes at 4 °C to remove cell debris and polymers, after which the resulting supernatants were stored at −20 °C.

For PSMA4, LAP3, and LZIC, 100 μL of either the pretreated supernatant or serially diluted standard was added to the antibody-precoated 96-well plates and incubated at room temperature for 2 hours. For RIC8B, 50 μL of the pretreated supernatant or standard was added to the respective plate, followed by 100 μL of HRP-conjugated detection antibody, and the mixture was incubated at 37°C in the dark for 60 minutes.

After primary incubation for all proteins, the wells were emptied and washed five times with wash buffer (for RIC8B, a 1:20 dilution prepared from the 20× concentrated solution). During manual washing, each well was filled with wash buffer and allowed to stand for 20 seconds. Subsequently, 100 μL of enzyme-conjugated detection antibody was added to each well for PSMA4, LAP3, LZIC, and RIC8B, followed by incubation at room temperature for 1 hour before undergoing another wash cycle. Color development was initiated by adding substrate solution: for PSMA4, LAP3, and LZIC, the substrate solution was applied directly, whereas for RIC8B, substrate solutions A (0.01% hydrogen peroxide) and B (0.1% TMB) were mixed at a 1:1 ratio and used within 15 minutes. All plates were incubated in the dark for 15 minutes, and the reaction was then terminated with the stop solution. Absorbance was measured at 450 nm using a microplate reader.

Standard curves for PSMA4, LAP3, LZIC, and RIC8B were generated from serial dilutions, which demonstrated excellent linearity, with coefficients of determination (R^2^) as follows: LZIC, 0.999; LAP3, 0.999; PSMA4, 0.998; RIC8B, 0.996. The intra-assay coefficients of variation (CV) were: LZIC, 4.48% (95% CI: 2.88–6.67%); LAP3, 8.51% (95% CI: 4.35–13.03%); PSMA4, 11.95% (95% CI: 7.78–16.77%); RIC8B, 4.55% (95% CI: 2.45–7.36%). Protein concentrations in unknown samples were calculated by interpolation from the corresponding standard curves.

### Immumohistochemical staining (IHC)

Paraffin-embedded human brain sections were dewaxed in graded xylene and ethanol and then rinsed with distilled water. Antigen retrieval was performed with citrate buffer (pH 6.0) under high-pressure conditions. After natural cooling, sections were washed with PBS (3 × 5 minutes). Endogenous peroxidase activity was blocked by incubating the sections with 3% hydrogen peroxide for 15 minutes at room temperature in the dark, followed by PBS washes (3 × 5 minutes). To reduce nonspecific binding, sections were blocked with 3% goat serum for 30 minutes at room temperature. After blocking, slides were incubated overnight at 4 °C with appropriately diluted primary antibodies (LZIC, Rb, 1: 100, Proteintech, 14543-1-AP; LAP3, Rb, 1: 100, Proteintech, 14612-1-AP; PSMA4, Rb, 1: 100, Proteintech, 11943-2-AP; RIC8B, Rb, 1: 100, Proteintech, 17790-1-AP), while negative technical controls (without primary antibody) were included to confirm specificity of the secondary antibodies and chromogenic reaction.

On the following day, slides were washed with PBST (3 × 5 minutes) and incubated with secondary antibodies for 60 minutes at room temperature, followed by additional PBST washes (3 × 5 minutes). Immunoreactivity was visualized using freshly prepared DAB substrate, and the reaction was stopped by rinsing the slides with water once a brown coloration became evident. Nuclei were counterstained with hematoxylin, and the sections were dehydrated through graded ethanol and xylene. Finally, slides were mounted with neutral resin for microscopic evaluation.

## Supplementary information

Supplementary materials

## Data Availability

The raw MS data have been deposited in the ProteomeXchange Consortium via the iProX partner repository under the dataset identifier PXD057559. Reviewers can access the private dataset at: http://proteomecentral.proteomexchange.org/cgi/GetDataset?ID=PXD057559.
