# Supplementary material for: A comparative analysis of serum and tissue proteomic profiles in non-small cell lung cancer patients with or without brain metastasis
Source: Cell Death Discov. 2026 Apr 13;12:230. doi: 10.1038/s41420-026-03109-8 (PMC13183923; doi:10.1038/s41420-026-03109-8)
Supplement: Supplementary file 1 — Supplementary materials [file 41420_2026_3109_MOESM1_ESM.pdf]

Supplementary table 1 Pathological and clinical baseline characteristics of study participants.

	BrM patient N=22	NSCLC patient N=32	NC patient N=35	P value
Age (mean±SD)	63.77±4.17	64.18±4.66	63.88±4.06	0.932
Male (%)	15 (68.2)	13 (40.6)	14 (40.0)	0.075
Stage (%)				<0.001
I		15		
II		10		
III		7		
IV	22 (100)	0		
Smoking history (%)	5 (22.7)	7 (21.9)	3 (8.6)	0.243
Multiplicity of tumor				0.074
Solitary (%)	9(40.9)	21(65.6)	/	
Multiplex (%)	13 (59.1)	11(34.4)	/	
Other organization Metastasis (%)	5 (22.7)	0 (0)	/	0.008
NSCLC tissues Histology				0.698
Adenocarcinoma (%)	20 (90.9)	28 (87.5)	/	
Squamous carcinoma (%)	2 (9.1)	4(12.5)	/	

supplementary table 2 The list of differentially expressed proteins between BrM and NAT in serum samples by Student's t-test

NO.	Gene Name	Uniprot	Protein name	Drug (DrugBank)	Down in BrM/NSCLC	Up in BrM/NSCLC	Down in BrM/NC	Up in BrM/NC	Down in NSCLC/NC	Up in NSCLC/NC	FDR (BrM/NSCLC)	FDR (BrM/NC)	FDR (NSCLC/NC)	*p* -value (BrM/NSCLC)	p-value (BrM/NC)	p-value (NSCLC/NC)	Log2(FC (BrM/NSCLC))	Log2(FC (BrM/NC))	Log2(FC (NSCLC/NC))
1	IGLV8-61	A0A075B6I0	Immunoglobulin lambda variable 8-61		no	no	no	no	yes	no	0.280133767	0.287742164	0.009927729	0.255636342	0.191619351	0.008182392	0.69684024	-0.82608083	-1.52292107
2	IGLV7-46	A0A075B6I9	Immunoglobulin lambda variable 7-46		no	yes	no	no	no	no	4.34085E-07	0.03134118	0.006274106	2.34056E-07	0.01199128	0.005100021	1.757400272	0.800156536	-0.957243736
3	IGHV3-64	A0A075B6Q5	Immunoglobulin heavy variable 3-64		no	no	no	no	yes	no	0.163874225	0.39725858	0.003452808	0.145682478	0.290698961	0.002745641	0.645351807	-0.517923127	-1.163274934
4	IGKV1-27	A0A075B6S5	Immunoglobulin kappa variable 1-27		no	yes	no	no	no	no	0.001616227	0.811825408	0.013124806	0.001197341	0.741567749	0.010936347	1.174394717	0.130153429	-1.044241288
5	IGKV1-37	A0A075B6S9	Probable non-functional immunoglobulinn kappa variable 1-37		no	yes	no	no	no	no	0.000598239	0.105977246	0.009367468	0.000421505	0.05455879	0.00769516	1.278259747	0.510388496	-0.767871251
6	IGHV3OR15-7	A0A075B7D8	Immunoglobulin heavy variable 3/OR15-7 (pseudogene)		no	yes	no	no	no	no	0.00020943	0.170376225	0.019237181	0.000141012	0.09958556	0.01620386	1.251554321	0.521708529	-0.729845792
7	IGKV3D-15	A0A087WSY6	Immunoglobulin kappa variable 3D-15		no	no	no	no	yes	no	0.026641745	0.645432463	3.81882E-05	0.021921798	0.546877528	2.6993E-05	0.896126925	-0.222375613	-1.118502538
8	IGKV3D-11	A0A0A0MRZ8	Immunoglobulin kappa variable 3D-11		no	yes	no	no	no	no	8.17442E-05	0.380641154	0.000182221	5.31134E-05	0.272502502	0.000134499	1.16860125	0.290621539	-0.877979711
9	IGKJ1	A0A0A0MT89	Immunoglobulin kappa joining 1		no	yes	no	no	no	no	0.001276266	0.003361353	0.523131118	0.000936815	0.000732583	0.502272242	1.087224843	0.904261548	-0.182963295
10	IGHV5-51	A0A0C4DH38	Immunoglobulin heavy variable 5-51		no	no	no	no	yes	no	0.026924712	0.10941577	1.38882E-06	0.022166833	0.056864471	8.98944E-07	0.636875593	-0.498776856	-1.135652449
11	IGKV1-8	A0A0C4DH67	Immunoglobulin kappa variable 1-8		no	no	no	no	yes	no	0.001802855	0.512794652	2.90228E-05	0.001342134	0.405425401	2.03567E-05	0.960854935	-0.248845827	-1.209700762
12	IGKV1-12	A0A0C4DH73	Immunoglobulin kappa variable 1-12		no	yes	no	no	no	no	0.000418433	0.279489033	0.005076978	0.000291216	0.184890797	0.004097008	1.159240804	0.408591253	-0.750649552
13	A0A0G2JRQ6	A0A0G2JRQ6	Ig-like domain-containing protein		no	no	no	yes	no	no	0.546696076	0.000812206	0.000107025	0.520191101	0.000129909	7.79771E-05	0.169562746	1.119080637	0.949517891
14	IGHV3-64D	A0A0J9YX35	Immunoglobulin heavy variable 3-64D		no	no	no	no	yes	no	0.041674349	0.094992232	1.13808E-08	0.034970953	0.047689802	6.52835E-09	0.6897952	-0.678653146	-1.368448346
15	OR6C74	A6NCV1	olfactory receptor family 6 subfamily C member 74		yes	no	no	no	no	no	0.000295865	0.839128156	9.34687E-05	0.00020237	0.77715358	6.78464E-05	-1.056040507	-0.076897592	0.979142915
16	MATN2	O00339	matrilin 2		no	yes	no	no	no	no	3.08263E-11	0.078367372	1.99422E-10	1.02661E-11	0.037212962	1.04274E-10	1.25641922	0.266417214	-0.990002006
17	CCN1	O00622	cellular communication network factor 1		no	yes	no	no	no	no	6.78743E-05	0.027147707	0.013009679	4.36401E-05	0.010037394	0.010834522	1.059990408	0.602993433	-0.456996975
18	MAST4	O15021	microtubule associated serine/threonine kinase family member 4		no	no	no	no	yes	no	3.43222E-06	0.010300792	6.76591E-12	1.9766E-06	0.002963753	3.29865E-12	0.8808745	-0.501144317	-1.382018817
19	TRANK1	O15050	tetratricopeptide repeat and ankyrin repeat containing 1		no	no	no	no	yes	no	6.00365E-07	0.280327731	2.04284E-10	3.26977E-07	0.18557264	1.06909E-10	0.931387869	-0.160853026	-1.092240895
20	ABCC3	O15438	ATP binding cassette subfamily C member 3		no	yes	no	no	no	no	8.3025E-06	0.241607535	9.12509E-06	4.93184E-06	0.153262596	6.17297E-06	1.265880119	0.358503144	-0.907376975
21	GNG7	O60262	G protein subunit gamma 7		no	no	no	no	no	yes	0.03151632	0.042064014	1.97814E-10	0.026146979	0.017546919	1.03344E-10	-0.616468526	0.699691846	1.316160372
22	ZG16	O60844	zymogen granule protein 16		no	no	no	no	yes	no	9.46049E-05	0.155235875	4.60361E-09	6.18554E-05	0.088414814	2.56359E-09	0.966379855	-0.304334182	-1.270714037
23	LILRB3	O75022	leukocyte immunoglobulin-like receptor subfamily B member 3		no	yes	no	no	no	no	0.000962196	0.152685986	0.072778258	0.000694508	0.086616608	0.064435304	1.288723982	0.63797441	-0.650749572
24	LEFTY1	O75610	left-right determination factor 1		no	yes	no	no	no	no	1.88327E-07	0.015574129	6.41083E-06	9.8302E-08	0.005003198	4.29325E-06	1.246382265	0.563158321	-0.683223945
25	ATRN	O75882	attractin		no	no	no	no	no	yes	0.753966993	0.011995607	1.01871E-06	0.739277106	0.003614444	6.51291E-07	-0.113737744	1.104341538	1.218079282
26	TRIO	O75962	trio Rho guanine nucleotide exchange factor		no	yes	no	no	no	no	2.65296E-16	1.68362E-06	7.20821E-05	1.4545E-17	3.66171E-08	5.19958E-05	1.498579617	0.839722736	-0.658856881
27	SASH3	O75995	SAM and SH3 domain containing 3		no	no	no	no	yes	no	0.013782764	0.000168341	1.92441E-08	0.011059934	1.69221E-05	1.12396E-08	0.481739278	-0.783635489	-1.265374766
28	KRT33A	O76009	keratin 33A		no	no	no	no	yes	no	2.22083E-06	0.351669784	7.61318E-09	1.26085E-06	0.245547412	4.29125E-09	0.991904291	-0.237272003	-1.229176294
29	SRP72	O76094	signal recognition particle 72		no	no	no	no	no	yes	0.00136258	0.101841491	7.9949E-09	0.001002024	0.052143582	4.51728E-09	-0.695669576	0.371181826	1.066851402
30	SLIT2	O94813	slit guidance ligand 2		no	yes	no	no	no	no	7.45622E-13	0.0295823	3.6978E-13	1.75679E-13	0.011111941	1.69727E-13	1.212864345	0.311964916	-0.90089943
31	APOM	O95445	apolipoprotein M		no	no	no	no	yes	no	4.39452E-05	0.035031585	5.97915E-11	2.77769E-05	0.013714223	3.0424E-11	0.754228385	-0.370070098	-1.124298483
32	ABCA1	O95477	ATP binding cassette subfamily A member 1	ATP; Glyburide; Probucol	no	yes	no	no	no	no	6.05774E-06	0.04268395	0.002306069	3.5545E-06	0.017889739	0.001810791	1.064619948	0.488277767	-0.576342181
33	H6PD	O95479	hexose-6-phosphate dehydrogenase/glucose 1-	NADH	no	no	no	no	yes	no	0.067780476	0.020989797	1.71135E-05	0.058029523	0.007228023	1.18174E-05	0.471788136	-0.84519296	-1.316981096
34	DDAH2	O95865	DDAH family member 2, ADMA-independent	Citrulline	no	no	no	no	no	yes	0.000795722	0.000653306	1.31076E-09	0.000570021	0.000100053	7.09724E-10	-0.675888867	0.845000885	1.520889751
35	NDUFB10	O96000	NADH:ubiquinone oxidoreductase subunit B10	NADH	no	no	no	no	yes	no	0.004575832	1.90558E-06	9.92147E-07	0.003520509	4.31714E-08	6.33859E-07	0.836877811	-0.751128204	-1.588006015
36	ALDH1A1	P00352	aldehyde dehydrogenase 1 family member A1	NADH; Vitamin A; Tretinoin; 1,4-	no	no	no	no	no	yes	1.10245E-06	0.107753042	1.04005E-13	6.15414E-07	0.055699459	4.65594E-14	-0.960847136	0.338845556	1.299692692
37	HPR	P00739	haptoglobin-related protein	Zinc; Copper; Zinc acetate	no	no	no	no	yes	no	0.099857076	0.038612664	6.94073E-07	0.086843218	0.015680325	4.39968E-07	0.524740022	-0.708130087	-1.232870109
38	CFD	P00746	complement factor D	2-Aminobenzyl alcohol; 3,4-Dichloroisocoumarin	no	yes	no	no	no	no	5.87694E-11	9.14518E-05	1.19035E-06	2.06106E-11	7.54023E-06	7.6426E-07	1.492069372	0.735075989	-0.756993383
39	CFB	P00751	complement factor B	Zinc; 4-guanidinobenzoic acid; Diisopropylphosphono Group; MLN2222; Zinc acetate	no	yes	no	no	no	no	1.61485E-09	0.021067983	4.10396E-11	6.85599E-10	0.007300378	2.0715E-11	1.188640336	0.383763909	-0.804876427
40	ASS1	P00966	argininosuccinate synthase 1	Arginine; Aspartic acid; Citrulline; ATP	no	no	no	no	yes	no	0.000990695	0.131430831	1.18828E-07	0.000715976	0.071998131	7.24742E-08	0.942969267	-0.487468868	-1.430438135
41	SERPINA3	P01011	serpin family A member 3	Zinc; Zinc acetate; Zinc chloride; Zinc sulfate, unspecified form	no	no	no	yes	no	no	0.014510266	0.000501008	0.013359869	0.01166344	7.03727E-05	0.011144322	0.653160163	1.046050629	0.392890466
42	C3	P01024	complement C3	Human immunoglobulin G; Zinc; S-Hydroxycysteine; Mirococept; Copper; Zinc acetate; Zinc chloride; Zinc sulfate, unspecified form	no	yes	no	no	no	no	0.000634344	0.175384757	8.45478E-13	0.000448381	0.103069338	3.95348E-13	1.5048723	0.622268026	-0.882604274
43	C5	P01031	complement C5	Human immunoglobulin G; Eculizumab; Zinc; Copper; Ravulizumab; Zinc acetate; Zinc chloride; Zinc sulfate, unspecified form	no	no	no	yes	no	no	0.025065391	0.007822333	0.10322096	0.020579288	0.002080521	0.092323596	1.033035188	1.388666759	0.355631572
44	CST3	P01034	cystatin C		no	yes	no	no	no	no	3.20406E-08	0.196579645	6.61335E-10	1.5592E-08	0.118197186	3.53291E-10	1.180915211	0.248888203	-0.932027008
45	CSTA	P01040	cystatin A		no	yes	no	no	no	no	3.80147E-10	0.000466975	0.000496514	1.50887E-10	6.28417E-05	0.000375929	1.296469355	0.765392678	-0.531076676
46	NPPA	P01160	natriuretic peptide A		no	yes	no	no	no	no	7.40303E-07	0.000791379	0.005115444	4.07217E-07	0.000126219	0.004130367	1.402757652	0.934373648	-0.468384004
47	JCHAIN	P01591	joining chain of multimeric IgA and IgM	Zinc; Zinc acetate; Zinc chloride; Zinc sulfate, unspecified form	no	yes	no	no	no	no	0.001195093	0.863685292	4.5397E-06	0.0008729	0.808130344	3.01755E-06	1.019211594	0.064914651	-0.954296943
48	IGKV1-17	P01599	None	None	no	no	no	no	yes	no	0.62127508	0.018114348	0.00103136	0.597065901	0.006065475	0.000793965	0.214262308	-1.128567991	-1.342830298
49	IGKV1-5	P01602	None	None	no	no	no	no	yes	no	0.438400559	0.017853825	1.71471E-05	0.411584032	0.005970151	1.18483E-05	0.277661597	-0.997279232	-1.274940829
50	IGLV2-14	P01704	None	None	no	no	no	no	yes	no	0.255015961	0.052702051	0.001197048	0.231790674	0.02328251	0.000927482	0.487544583	-0.919633263	-1.407177845
51	IGLV3-19	P01714	None	None	no	no	yes	no	no	no	0.836760405	0.004292727	0.002544929	0.826144505	0.000999756	0.002006423	-0.04210051	-1.003558075	-0.961457565
52	IGLV6-57	P01721	None	None	no	yes	no	no	no	no	0.001720767	0.261303094	0.013485389	0.001279465	0.169781893	0.011255137	1.135242611	0.426008641	-0.70923397
53	CD8A	P01732	CD8 subunit alpha		no	yes	no	no	no	no	1.16973E-09	0.047176567	3.85648E-06	4.9079E-10	0.02028571	2.55643E-06	1.110671469	0.32675298	-0.783918489
54	IGHV3-9	P01782	None	None	no	no	no	no	yes	no	0.173449763	0.064331712	1.77293E-05	0.154902348	0.02935488	1.22747E-05	0.410154741	-0.667551867	-1.077706608
55	IGHM	P01871	None	None	no	no	no	no	yes	no	0.055558324	0.039287172	1.81232E-09	0.047219948	0.016092254	9.9197E-10	0.57465218	-0.654556424	-1.229208603
56	MB	P02144	myoglobin	Porphyrin Fe(III); N-Butyl Isocyanide; Biliverdine IX Alpha; Beta-D-Glucose; Methylethylamine; Tetrazolyl Histidine; Nitrosoethane; 1-Methylimidazole; Imidazole; 4-Methylimidazole; Ethyl Isocyanide; N-Propyl Isocyanide; Isocyanomethane; Nitrous acid; Carbon	no	yes	no	no	no	no	0.000387215	0.262246697	0.002199038	0.000268261	0.170751474	0.001724755	1.079989801	0.310889396	-0.769100405
57	APOC3	P02656	apolipoprotein C3	Copper	no	no	no	no	yes	no	2.9707E-07	0.382485988	3.38995E-14	1.58429E-07	0.274896224	1.48071E-14	0.901214341	-0.147013433	-1.048227774
58	SLC4A1	P02730	solute carrier family 4 member 1 (Diego blood group)		no	no	no	no	yes	no	0.743285786	0.120789064	0.000913001	0.728130435	0.064833864	0.00069954	0.174140502	-0.889160161	-1.063300664
59	C9	P02748	complement C9	Copper	no	yes	no	no	no	no	2.83182E-14	1.37597E-06	2.33781E-06	4.27275E-15	2.80556E-08	1.53065E-06	1.846393859	0.968850697	-0.877543162
60	AHSG	P02765	alpha 2-HS glycoprotein	Zinc; Copper; Zinc acetate; Zinc chloride; Zinc sulfate, unspecified form	no	no	no	no	yes	no	1.31515E-07	0.064213937	2.10857E-17	6.79327E-08	0.029241054	7.93938E-18	0.891114588	-0.283912672	-1.17502726
61	ANG	P03950	angiogenin		no	yes	no	no	no	no	3.72483E-07	0.186227845	7.72741E-09	1.99996E-07	0.110680723	4.35914E-09	1.199545461	0.279674329	-0.919871131
62	MMP1	P03956	matrix metallopeptidase 1	Marimastat; CGS-27023; N-[3-(N'-HYDROXYCARBOXAMIDO)-2-(2-METHYLPROPYL)-PROPANOYL]-O-TYROSINE-N-METHYLAMIDE; METHYLAMINO-PHENYLALANYL-LEUCYL-HYDROXAMIC ACID; [[1-[N-HYDROXY-ACETAMIDYL]-3-METHYL-BUTYL]-CARBONYL-LEUCINYL]-ALANINE ETHYL ESTER; N-HYDROXY-2-[4-(4-PHENOXY-BENZENESULFONYL)-TETRAHYDRO-	no	no	no	no	no	yes	0.000369574	0.000182067	1.27239E-11	0.000255872	1.88914E-05	6.27835E-12	-0.712174452	0.767408518	1.47958297

63	ANXA1	P04083	annexin A1	Amcinonide; Fluocinolone acetonide; Hydrocortisone; Methylprednisolone; Clobetasol propionate; Budesonide; Dexamethasone; Hydrocortisone aceponate; Hydrocortisone acetate; Hydrocortisone butyrate; Hydrocortisone cypionate; Hydrocortisone phosphate; Hydrocortisone probutate; Hydrocortisone valerate; Prednisolone phosphate; Dexamethasone acetate; Betamethasone phosphate	yes	no	no	no	no	no	1.29584E-05	0.011833298	0.061316035	7.81498E-06	0.00353873	0.053898101	-1.084218999	-0.699791536	0.384427463
64	APOB	P04114	apolipoprotein B	Zinc chloride; Zinc sulfate, unspecified form	no	no	no	no	yes	no	6.32658E-05	0.000860309	1.08801E-12	4.04477E-05	0.000141111	5.12699E-13	0.651714701	-0.526501852	-1.178216553
65	IGLV7-43	P04211	None	None	no	no	no	no	yes	no	0.036571858	0.063150917	8.51809E-07	0.030490357	0.028642532	5.42657E-07	0.67432809	-0.681733897	-1.356061988
66	SHBG	P04278	sex hormone binding globulin	Masoprocol; Diethylstilbestrol; Levonorgestrel; Progesterone; Spironolactone; Toremifene; Estrone; Tamoxifen; Clomifene; Dienestrol; Hesperetin; Zinc; Genistein; Naringenin; Quercetin; 4'-Hydroxyflavanone; Afimoxifene; Estriol; Phenolphthalein; Norethynodrel; Dioxybenzone; Zeranol; Gestrinone; Equol; Zinc acetate; Zinc	no	no	yes	no	no	no	0.024551417	0.000523195	0.044970228	0.020146179	7.46744E-05	0.038959255	-0.906504222	-1.601869543	-0.69536532
67	CYBB	P04839	cytochrome b-245 beta chain	Dextromethorphan	no	no	no	no	no	yes	0.10113878	0.090523289	0.000987983	0.088124094	0.044543857	0.000760124	-0.563792409	0.485911357	1.049703766
68	ALDOB	P05062	aldolase, fructose-bisphosphate B	1,6-Fructose Diphosphate (Linear Form); sn-glycerol 3-phosphate; Dihydroxyacetone phosphate	no	no	no	no	yes	no	4.78887E-06	0.612556744	3.80512E-07	2.7926E-06	0.510417695	2.38272E-07	0.944440407	-0.139651008	-1.084091416
69	ARG1	P05089	arginase 1	Ornithine; 2(S)-Amino-6-Boronohexanoic Acid; nor-NOHA; Dinor-N(Omega)-Hydroxy-L-Arginine; S-{2-[Amino(Dihydroxy)-Lambda~4~-Sulfanyl]Ethyl}-D-Cysteine; N(5)-[(hydroxyamino)(imino)methyl]-L-ornithine; S-2-(Boronoethyl)-L-Cysteine; Urea; Descarboxy-nor-N(Omega)-Hydroxy-L-Arginine; S,S-(2-Hydroxyethyl)Thiocysteine; DEHYDRO-2(S)-AMINO-6-	no	yes	no	no	no	no	6.3273E-06	0.530859261	1.63553E-10	3.71553E-06	0.424783623	8.49258E-11	1.146597055	0.163131946	-0.983465109
70	F13B	P05160	coagulation factor XIII B chain	Zinc; Catridecacog; Thrombin; Human thrombin; Zinc acetate	no	yes	no	no	no	no	6.18299E-08	0.006464734	2.3601E-07	3.0957E-08	0.001628633	1.45755E-07	1.146457885	0.534336876	-0.61212101
71	SERPIND1	P05546	serpin family D member 1	Ardeparin; Zinc; Sulodexide; Copper; Bemiparin; Zinc acetate; Zinc chloride; Zinc sulfate, unspecified form	no	no	no	no	yes	no	0.000596983	1.20501E-05	6.16179E-17	0.00042035	5.28509E-07	2.38152E-17	0.523569334	-0.771770209	-1.295339543
72	CCK	P06307	cholecystokinin	Camostat	no	no	no	no	yes	no	0.007753276	0.169416087	4.74688E-11	0.006098635	0.098870829	2.40463E-11	0.685495556	-0.386489919	-1.071985475
73	IGKV4-1	P06312	None	None	no	no	no	no	yes	no	0.001964497	0.496220798	1.51516E-07	0.001466919	0.385825377	9.28181E-08	0.849000021	-0.230806656	-1.079806678
74	S100A6	P06703	S100 calcium binding protein A6	Calcium citrate; Calcium Phosphate; Calcium phosphate dihydrate	yes	no	no	no	no	no	1.00843E-05	0.038944911	3.84809E-05	6.02684E-06	0.015863831	2.72174E-05	-1.073206951	-0.515126151	0.5580808
75	PROS1	P07225	protein S	Drotrecogin alfa; Menadione; Sodium tetradecyl sulfate; Kappadione	no	yes	no	no	no	no	8.04168E-09	0.125271452	1.64858E-08	3.70566E-09	0.067829355	9.59682E-09	1.213707687	0.260259439	-0.953448248
76	C8B	P07358	complement C8 beta chain	Zinc; Copper; Zinc acetate	no	yes	no	no	no	no	6.87539E-11	0.009652428	9.76361E-08	2.45549E-11	0.00273347	5.92806E-08	1.537929652	0.592810161	-0.945119491
77	CTSB	P07858	cathepsin B	2-Aminoethanimidic Acid; 3-Amino-4-Oxybenzyl-2-Butanone; 3-Methylphenylalanine; N-(3-Propylcarbamoyloxirane-2-Carbonyl)-Isoleucyl-Proline; 2-Pyridinethiol; Diphenylacetic acid; N-[1-Hydroxycarboxyethyl-Carbonyl]Leucylamino-2-Methyl-Butane; N-{[(2S,3S)-3-(Ethoxycarbonyl)-2-oxiranyl]carbonyl}-L-threonyl-L-isoleucine; N-{[(2S,3S)-3-(ETHOXYCARBONYL)OXIRAN-2-YL]CARBONYL}-L-ISOLEUCINE; BENZYL N-({(2S,3S)-3-[(PROPYLAMINO)CARBONYL]OXIRAN-2-YL}CARBONYL)-L-ISOLEUCYL-L-PROLINATE; METHYL N-({(2S,3S)-3-[(PROPYLAMINO)CARBONYL]OXIRAN-2-YL}CARBONYL)-L-ISOLEUCYL-L-PROLINATE; N-{[(2S,3S)-3-(ETHOXYCARBONYL)OXIRAN-2-YL]CARBONYL}-L-ISOLEUCYL-L-ALANINE; N-{[(2S,3S)-3-(ETHOXYCARBONYL)OXIRAN-2-	no	no	no	yes	no	no	0.000751346	4.32954E-05	0.01329749	0.000536189	2.90336E-06	0.011086263	0.667200017	1.077868364	0.410668347
78	MMP2	P08253	matrix metallopeptidase 2	Marimastat; Captopril; SC-74020; Halofuginone; AE-941; Endostatin	no	yes	no	no	no	no	7.85661E-10	0.009576415	5.46351E-07	3.22167E-10	0.002704336	3.4509E-07	1.142128398	0.420283435	-0.721844963
79	CTSG	P08311	cathepsin G	Bis-Napthyl Beta-Ketophosphonic Acid; 2-[3-({Methyl[1-(2-Naphthoyl)Piperidin-4-Yl]Amino}Carbonyl)-2-Naphthyl]-1-(1-Naphthyl)-2-Oxoethylphosphonic Acid	no	no	no	no	no	yes	0.453319699	0.002228735	0.000349081	0.426001385	0.000445343	0.000261455	-0.271087581	0.87681423	1.147901811
80	CFH	P08603	complement factor H	Zinc; Copper; Zinc acetate; Zinc chloride; Zinc sulfate, unspecified form	no	yes	no	no	no	no	6.1651E-12	0.143816952	1.84684E-17	1.78221E-12	0.080477542	6.9204E-18	1.061941545	0.171113658	-0.890827887
81	SNRPA	P09012	small nuclear ribonucleoprotein polypeptide A	Malonic acid	no	no	yes	no	no	no	0.000422137	0.000409533	0.842992869	0.000293985	5.16339E-05	0.831915936	-0.983648719	-1.031345271	-0.047696553
82	HNRNPA1	P09651	heterogeneous nuclear ribonucleoprotein A1		yes	no	no	no	no	no	7.60286E-05	0.014115862	0.054989759	4.92274E-05	0.00443314	0.048088008	-1.053583837	-0.614637794	0.438946043
83	C4A	P0C0L4	complement C4A (Chido/Rodgers blood group)		no	yes	no	no	no	no	0.000183484	0.502644567	3.41034E-07	0.00012296	0.393552248	2.12934E-07	1.011746134	0.187453827	-0.824292306
84	C4B	P0C0L5	complement C4B (Chido/Rodgers blood group)	Human immunoglobulin G; Zinc; Copper; Zinc acetate; Zinc chloride; Zinc sulfate, unspecified form	no	no	no	no	yes	no	0.064998687	0.011599305	1.34372E-09	0.055544868	0.003462351	7.28177E-10	0.503309014	-0.73729308	-1.240602095
85	IGHV1-8	P0DP01	None	None	no	yes	no	no	no	no	1.08264E-05	0.043089752	0.000498056	6.48507E-06	0.018079343	0.000377322	1.840142645	0.87760353	-0.962539115
86	IGHV3-30-3	P0DP02	None	None	no	no	no	no	yes	no	0.002538623	0.084639062	3.16846E-09	0.001912882	0.040958096	1.75292E-09	0.632968713	-0.377014706	-1.009983419
87	SPP1	P10451	secreted phosphoprotein 1		no	yes	no	no	no	no	7.68799E-13	3.64737E-05	4.2227E-07	1.82533E-13	2.26412E-06	2.65186E-07	1.802379409	0.845846865	-0.956532543
88	CD37	P11049	CD37 molecule		no	no	no	no	no	yes	0.014304902	0.11697523	5.93773E-08	0.011491885	0.062648266	3.55134E-08	-0.766727364	0.505997192	1.272724556
89	SLC2A1	P11166	solute carrier family 2 member 1	Resveratrol; Carboxymethylcellulose	no	no	no	no	yes	no	0.138035597	0.025969314	1.29487E-07	0.121461318	0.009484036	7.92059E-08	0.644095572	-1.007801782	-1.651897354
90	RALA	P11233	RAS like proto-oncogene A	Guanosine-5'-Diphosphate	no	no	no	no	no	yes	0.000853111	0.004009591	9.39453E-12	0.000612678	0.000912014	4.61E-12	-0.744825521	0.81561861	1.560444131
91	DEFA4	P12838	defensin alpha 4		no	no	no	no	yes	no	0.000691219	0.068924363	1.73109E-06	0.000490775	0.031820365	1.12556E-06	0.710307807	-0.393344566	-1.103652374
92	VCAN	P13611	versican	Hyaluronic acid	no	yes	no	no	no	no	4.25231E-05	0.017096725	4.55873E-05	2.68395E-05	0.005639518	3.24191E-05	1.147197851	0.661546132	-0.485651719
93	PDIA4	P13667	protein disulfide isomerase family A member 4		no	no	no	no	no	yes	0.00021815	0.005967298	6.80274E-21	0.000147279	0.001484389	2.15456E-21	-0.785270643	0.621400827	1.406671469
94	PLS3	P13797	plastin 3		no	no	no	no	no	yes	3.85203E-05	0.123190152	4.3315E-12	2.42257E-05	0.066479144	2.0843E-12	-0.850389971	0.325631308	1.176021278
95	HGF	P14210	hepatocyte growth factor	Heparin; O2-Sulfo-Glucuronic Acid; N,O6-Disulfo-Glucosamine; ABT-510; Foretinib	no	yes	no	no	no	no	3.29558E-10	2.96829E-05	0.000243115	1.29464E-10	1.74842E-06	0.000180657	1.467494857	0.900575694	-0.566919163
96	PRKCSH	P14314	PRKCSH beta subunit of glucosidase II		no	no	no	no	no	yes	0.002190351	0.000836701	5.13348E-11	0.001638545	0.000135722	2.60512E-11	-0.561140663	0.579915605	1.141056268
97	NCF1	P14598	neutrophil cytosolic factor 1		no	no	no	no	yes	no	0.013591314	0.013882005	3.56337E-06	0.010893989	0.004333802	2.35405E-06	0.505629801	-0.605300044	-1.110929846

98	HSP90B1	P14625	heat shock protein 90 beta family member 1	Rifabutin; 2-Chlorodideoxyadenosine; Geldanamycin; Diglyme; N-Ethyl-5'-Carboxamido Adenosine; Radicicol; METHYL 3-CHLORO-2-{3-[(2,5-DIHYDROXY-4-METHOXYPHENYL)AMINO]-3-OXOPROPYL}-4,6-DIHYDROXYBENZOATE; 2-(3-AMINO-2,5,6-TRIMETHOXYPHENYL)ETHYL 5-CHLORO-2,4-DIHYDROXYBENZOATE;	no	yes	no	no	no	no	1.33635E-05	0.010157132	0.003375079	8.06532E-06	0.002908476	0.002680795	1.082543853	0.675151813	-0.407392039
99	MMP9	P14780	matrix metallopeptidase 9	Glutathione; Marimastat; Minocycline; Captopril; Glucosamine; Zinc; 2-Amino-N,3,3-Trimethylbutanamide; 2-{[Formyl(Hydroxy)Amino]Methyl}-4-Methylpentanoic Acid; AE-941; PG-530742; Endostatin; 5-(4-PHENOXYPHENYL)-5-(4-PYRIMIDIN-2-YLPIPERAZIN-1-YL)PYRIMIDINE-2,4,6(2H,3H)-TRIONE; (2R)-2-AMINO-3,3,3-TRIFLUORO-N-HYDROXY-2-{[(4-PHENOXYPHENYL)SULFONYL]METHYL}PROPANAMIDE; (3R)-4,4-DIFLUORO-3-[(4-METHOXYPHENYL)SULFONYL]BUTAN	no	no	no	no	no	yes	0.362519109	0.004292727	0.001996574	0.335909188	0.000996611	0.001561435	-0.31196982	0.771090368	1.083060188
100	JUP	P14923	junction plakoglobin	Zinc; Zinc acetate	no	yes	no	no	no	no	6.58351E-07	0.036766616	2.60324E-05	3.6005E-07	0.014643342	1.82121E-05	1.739460514	0.747453362	-0.992007152
101	FABP4	P15090	fatty acid binding protein 4		no	yes	no	no	no	no	5.20409E-06	0.132326694	5.27195E-05	3.04652E-06	0.072728718	3.75749E-05	1.463765222	0.476352121	-0.987413101
102	ELN	P15502	elastin	Rofecoxib	no	yes	no	no	no	no	1.84643E-05	0.003322311	0.027861479	1.12945E-05	0.000722569	0.02365764	1.150753084	0.764256325	-0.386496758
103	IGLL1	P15814	immunoglobulin lambda like polypeptide 1	Copper	no	no	no	no	yes	no	0.165797441	0.417335363	0.002357167	0.147467321	0.310117805	0.001853051	0.641005631	-0.446625959	-1.08763159
104	DSP	P15924	desmoplakin	Zinc; Artenimol; Zinc acetate	no	yes	no	no	no	no	1.99546E-05	0.072555469	3.80068E-06	1.22603E-05	0.03379566	2.51771E-06	1.206448363	0.525014526	-0.681433837
105	TIMP2	P16035	TIMP metallopeptidase inhibitor 2		no	yes	no	no	no	no	1.43649E-07	0.225837741	2.12987E-09	7.45259E-08	0.140086936	1.17447E-09	1.22251049	0.262665402	-0.959845088
106	ANK1	P16157	ankyrin 1		yes	no	no	no	no	no	0.007522057	0.20144256	0.042509824	0.005906536	0.121942573	0.036712154	-1.146641057	-0.660436818	0.486204239
107	EPB42	P16452	erythrocyte membrane protein band 4.2		no	no	no	no	yes	no	0.282234563	0.041982696	2.83025E-05	0.257681307	0.017443649	1.98386E-05	0.44627307	-0.899418307	-1.345691377
108	CAPN2	P17655	calpain 2		no	no	no	no	no	yes	0.001017148	0.00031895	1.28187E-11	0.000735554	3.84417E-05	6.33093E-12	-0.601308274	0.717548516	1.31885679
109	IGFBP2	P18065	insulin like growth factor binding protein 2		no	yes	no	no	no	no	4.84152E-06	0.011400178	0.003060237	2.8277E-06	0.003373624	0.002425172	1.690583291	0.893901273	-0.796682017
110	GGT1	P19440	gamma-glutamyltransferase 1	Glutathione	yes	no	no	no	no	no	9.53575E-06	0.047419413	0.048492877	5.69034E-06	0.020454591	0.042186826	-1.205961828	-0.67458724	0.531374588
111	ITIH1	P19827	inter-alpha-trypsin inhibitor heavy chain 1	Zinc; Zinc acetate; Zinc chloride; Zinc sulfate, unspecified form	no	no	no	no	yes	no	0.158728813	0.002614822	5.44681E-11	0.140748657	0.000535523	2.76906E-11	0.295320776	-0.728066095	-1.02338687
112	TCN2	P20062	transcobalamin 2		no	yes	no	no	no	no	2.378E-08	0.013577523	1.16743E-05	1.13997E-08	0.004201834	7.98214E-06	1.243156507	0.517447438	-0.725709068
113	RAB4A	P20338	RAB4A, member RAS oncogene family		yes	no	no	no	no	no	3.67321E-15	5.46828E-07	4.21652E-05	3.84206E-16	8.03133E-09	2.98996E-05	-1.446149461	-0.846071773	0.600077688
114	LMNB1	P20700	lamin B1		no	yes	no	no	no	no	2.44979E-10	5.74017E-05	3.68149E-05	9.49068E-11	4.31748E-06	2.59889E-05	1.37566801	0.746781125	-0.628886885
115	OGN	P20774	osteoglycin		no	yes	no	no	no	no	8.50791E-12	0.000217934	4.2081E-07	2.54813E-12	2.36005E-05	2.64079E-07	1.515169148	0.65753701	-0.857632138
116	FLG	P20930	filaggrin		no	yes	no	no	no	no	0.000158909	0.009195913	0.138677699	0.000105627	0.002570632	0.125419432	1.183530419	0.835946217	-0.347584202
117	PTMS	P20962	parathymosin		no	no	no	no	no	yes	0.000123769	6.75973E-06	4.25247E-16	8.1597E-05	2.23589E-07	1.7033E-16	-0.636246464	0.826836881	1.463083345
118	TGM1	P22735	transglutaminase 1		no	yes	no	no	no	no	1.28836E-06	0.025969314	0.00110455	7.22695E-07	0.009473766	0.000851809	1.043391091	0.524455182	-0.518935909
119	GNLY	P22749	granulysin	3-(N-morpholino)propanesulfonic acid	no	no	no	no	yes	no	0.003622161	0.51102773	1.39535E-05	0.002762164	0.401968346	9.58471E-06	0.844889794	-0.193814449	-1.038704244
120	FBLN1	P23142	fibulin 1		yes	no	no	no	no	no	7.52243E-09	0.000434869	5.27038E-06	3.45275E-09	5.65507E-05	3.5104E-06	-1.337006495	-0.769587626	0.567418869
121	WARS1	P23381	tryptophanyl-tRNA synthetase 1	Tryptophan; Tryptophanyl-5'amp; Tryptophanamide	no	no	no	no	no	yes	0.002603407	0.023588159	7.03851E-11	0.001964057	0.008400676	3.5942E-11	-0.72678953	0.611171254	1.337960784
122	TNC	P24821	tenascin C		no	yes	no	no	no	no	1.59796E-08	0.000106968	0.001995871	7.53004E-09	9.35424E-06	0.001559981	1.411974392	0.958002045	-0.453972347
123	AZGP1	P25311	alpha-2-glycoprotein 1, zinc-binding	N-acetyl-alpha-neuraminic acid; Copper	no	yes	no	no	no	no	1.49978E-16	5.96511E-06	4.24573E-08	7.13532E-18	1.78386E-07	2.52397E-08	1.320080957	0.627139557	-0.6929414
124	RPL13	P26373	ribosomal protein L13		no	no	no	no	no	yes	0.001489669	0.598954572	6.32301E-05	0.001100884	0.49624087	4.54672E-05	-0.855913701	0.144504811	1.000418511
125	HMGB2	P26583	high mobility group box 2		yes	no	no	no	no	no	0.007550019	0.031550372	0.290522857	0.005935334	0.012151253	0.270119123	-1.164898621	-0.828797775	0.336100847
126	MARK3	P27448	microtubule affinity regulating kinase 3	Fostamatinib	no	no	no	no	no	yes	0.000299747	0.0001074	1.77123E-15	0.000205277	9.44067E-06	7.3273E-16	-0.633155634	0.859229794	1.492385428
127	APEX1	P27695	apurinic/apyrimidinic endodeoxyribonuclease 1	Lucanthone	no	no	yes	no	no	no	0.007385075	6.77001E-05	0.22131508	0.005795627	5.3068E-06	0.203465473	-0.785550703	-1.087047131	-0.301496428
128	GCA	P28676	grancalcin	Calcium citrate; Calcium Phosphate; Calcium phosphate dihydrate	yes	no	no	no	no	no	4.84694E-07	0.319925171	1.70184E-06	2.62521E-07	0.218163744	1.10577E-06	-1.197311273	-0.263169848	0.934141425
129	PCSK6	P29122	proprotein convertase subtilisin/kexin type 6		no	no	no	no	no	yes	6.46011E-05	0.008560324	3.11861E-11	4.13599E-05	0.002342744	1.56566E-11	-0.801725101	0.558727315	1.360452416
130	CCN2	P29279	cellular communication network factor 2		yes	no	no	no	no	no	0.001028706	0.513028241	6.28938E-05	0.000744378	0.405866474	4.51969E-05	-1.118233078	-0.27160375	0.846629328
131	SLC7A1	P30825	solute carrier family 7 member 1	L-Lysine; Arginine; Ornithine	no	no	no	no	yes	no	0.031172104	0.000594891	8.56811E-12	0.025847281	8.67942E-05	4.20059E-12	0.504029042	-0.918871108	-1.42290015
132	CASP14	P31944	caspase 14		no	no	no	no	yes	no	0.211910766	1.12279E-05	1.33389E-08	0.190690884	4.78216E-07	7.68787E-09	0.237747375	-0.897414153	-1.135161528
133	STIP1	P31948	stress induced phosphoprotein 1	Copper	no	no	no	no	no	yes	0.000332843	0.210315732	9.78348E-13	0.000229235	0.129240487	4.59695E-13	-0.914591196	0.348697079	1.263288275
134	ARRB2	P32121	arrestin beta 2		yes	no	no	no	no	no	3.28002E-12	0.001845077	0.001921992	8.88742E-13	0.000350289	0.001498753	-1.157151592	-0.616364007	0.540787585
135	RPL9	P32969	ribosomal protein L9		yes	no	no	no	no	no	0.001699607	0.002837437	0.583396545	0.001262348	0.000596939	0.561985072	-1.010021964	-0.839772711	0.170249254
136	CDH5	P33151	cadherin 5	Lenalidomide; FX06	no	no	yes	no	no	no	0.477591773	9.5554E-06	5.05194E-06	0.450325545	3.72344E-07	3.36262E-06	-0.152301101	-1.003301476	-0.851000374
137	RNASE4	P34096	ribonuclease A family member 4	2'-Deoxyuridine 3'-Monophosphate	no	yes	no	no	no	no	7.35588E-09	0.035851131	8.53403E-08	3.37297E-09	0.014094157	5.15831E-08	1.356877142	0.440161752	-0.91671539
138	PRSS3	P35030	serine protease 3	4-(1,3,2-Dioxaborolan-2-Yloxy)Butan-1-Aminium; 4-Hydroxybutan-1-Aminium; 4-(Hydroxymethyl)Benzamidine; Benzamidine; Diamino-N-[3-(1,3,2-dioxaborolan-2-yloxy)propyl]methaniminium; Guanidine-3-propanol; [4-(1,3,2-Dioxaborolan-2-Yloxy)Methyl]Benzamidine; 1,3,2-	no	no	no	no	yes	no	3.30136E-06	7.10514E-06	2.71021E-13	1.89824E-06	2.41452E-07	1.23538E-13	0.858326509	-0.737177025	-1.595503534
139	THBS2	P35442	thrombospondin 2		no	yes	no	no	no	no	1.12009E-07	0.026529855	8.78206E-09	5.74511E-08	0.009724809	4.98194E-09	1.330511882	0.516042546	-0.814469337
140	THBS4	P35443	thrombospondin 4		no	no	no	no	yes	no	0.000428259	0.197749328	3.49337E-10	0.000299024	0.119348484	1.83928E-10	0.844700607	-0.295781681	-1.140482288
141	TIMP3	P35625	TIMP metallopeptidase inhibitor 3	Pimagedine	no	no	no	no	no	yes	0.013058069	0.013214108	1.71569E-09	0.010454738	0.004067428	9.35975E-10	-0.737933648	0.861235591	1.59916924
142	CFHR2	P36980	complement factor H related 2		no	yes	no	no	no	no	0.000465818	0.908509393	7.28365E-05	0.000325461	0.871460981	5.2573E-05	1.000159515	0.040504215	-0.9596553
143	SRP14	P37108	signal recognition particle 14	Artenimol	yes	no	no	no	no	no	1.22709E-05	0.113552898	0.000395749	7.37809E-06	0.059889249	0.000297484	-1.393444031	-0.41998019	0.97346384
144	RPL3	P39023	ribosomal protein L3	(S)-3-phenyllactic acid; Omacetaxine mepesuccinate; Anisomycin; Puromycin; Xanthinol	yes	no	no	no	no	no	7.3673E-05	0.481096714	5.45769E-05	4.7602E-05	0.371449388	3.89481E-05	-1.192045564	-0.19557939	0.996466174
145	CAPG	P40121	capping actin protein, gelsolin like		no	no	no	no	yes	no	0.529027497	0.001833454	7.48729E-06	0.501434231	0.000346407	5.02771E-06	0.158291721	-0.998972821	-1.157264541
146	ARHGAP25	P42331	Rho GTPase activating protein 25		no	no	no	no	no	yes	0.008892562	0.030897648	8.09196E-18	0.007032777	0.011712374	2.98453E-18	-0.829848393	0.768927434	1.598775827
147	RPL35	P42766	ribosomal protein L35	Artenimol	yes	no	no	no	no	no	0.000426	0.724682299	0.000945704	0.000297061	0.636026105	0.000726311	-1.012438882	-0.094723977	0.917714906
148	UTRN	P46939	utrophin	Zinc; Zinc acetate; Zinc chloride; Zinc sulfate, unspecified form	yes	no	no	no	no	no	6.43054E-05	0.031153156	0.063405727	4.11415E-05	0.011885346	0.055792443	-1.385090567	-0.895166041	0.489924527
149	CXCL12	P48061	C-X-C motif chemokine ligand 12	CTCE-0214; Tinzaparin	no	yes	no	no	no	no	2.88488E-07	0.035851131	0.001968698	1.53459E-07	0.01408673	0.001536958	1.036834374	0.456031617	-0.580802757
150	SRP9	P49458	signal recognition particle 9		yes	no	no	no	no	no	2.67014E-06	0.000158194	0.00599214	1.52078E-06	1.54825E-05	0.004857244	-1.358831581	-0.629827014	0.729004567
151	CRIP1	P50238	cysteine rich protein 1		no	no	no	no	yes	no	0.29312266	0.011560943	9.24299E-09	0.268153444	0.003436329	5.26436E-09	0.431072913	-1.131192343	-1.562265256
152	BCAM	P50895	basal cell adhesion molecule (Lutheran blood group)		no	no	no	no	yes	no	0.005116209	0.000472088	1.58252E-10	0.003972968	6.4348E-05	8.21019E-11	0.553714038	-0.783548786	-1.337262824
153	HNRNPM	P52272	heterogeneous nuclear ribonucleoprotein M		yes	no	no	no	no	no	3.67793E-05	5.48758E-05	0.345289779	2.30641E-05	4.07776E-06	0.323073581	-1.177678814	-0.939380332	0.238298482
154	CRIP2	P52943	cysteine rich protein 2		no	no	no	no	yes	no	0.00983212	0.138906945	3.65821E-05	0.007796198	0.077191558	2.58081E-05	0.731896257	-0.339269271	-1.071165528
155	USP14	P54578	ubiquitin specific peptidase 14	Phenethyl Isothiocyanate	no	no	no	no	no	yes	0.001550574	0.004070768	4.37226E-16	0.001147298	0.000935151	1.75326E-16	-0.660106072	0.692360418	1.35246649
156	ANTXR2	P58335	ANTXR cell adhesion molecule 2	MDX-1303	no	no	no	no	yes	no	0.058338409	0.007740347	9.42785E-14	0.049668269	0.002044591	4.21199E-14	0.435191248	-0.701648821	-1.136840069
157	GABARAPL2	P60520	GABA type A receptor associated protein like 2		yes	no	no	no	no	no	2.1359E-07	0.000961582	0.000966926	1.11876E-07	0.000163822	0.000743134	-1.408191208	-0.91731527	0.490875938
158	RPS20	P60866	ribosomal protein S20		yes	no	no	no	no	no	5.64727E-09	0.014115862	4.01848E-06	2.55112E-09	0.004438789	2.66746E-06	-1.607900082	-0.640958087	0.966941995
159	B2M	P61769	beta-2-microglobulin	3-Indolebutyric Acid; N-Formylmethionine; Copper	no	yes	no	no	no	no	4.04622E-08	0.008958115	6.25807E-05	1.99469E-08	0.00248814	4.4899E-05	1.222769198	0.540230538	-0.68253866
160	RPS18	P62269	ribosomal protein S18	Artenimol	yes	no	no	no	no	no	7.11273E-07	0.481136169	6.09201E-06	3.90282E-07	0.371697856	4.07422E-06	-1.122066789	-0.155513479	0.96655331
161	SNRPD1	P62314	small nuclear ribonucleoprotein D1 polypeptide		yes	no	no	no	no	no	2.71885E-05	0.007740347	0.006226635	1.69019E-05	0.002043679	0.005055791	-1.468959867	-0.901734548	0.567225319

162	RPS15	P62841	ribosomal protein S15		no	no	no	no	no	yes	0.001375777	0.222521079	3.18574E-07	0.001012976	0.137626313	1.98044E-07	-0.929011965	0.364324882	1.293336847
163	RPL11	P62913	ribosomal protein L11	(S)-3-phenyllactic acid; Anisomycin;	yes	no	no	no	no	no	1.2131E-05	0.029516197	0.002143206	7.28852E-06	0.011073589	0.001679022	-1.048605632	-0.361199316	0.687406316
164	RPL8	P62917	ribosomal protein L8	(S)-3-phenyllactic acid; Anisomycin;	yes	no	no	no	no	no	4.77243E-07	0.020036511	0.000105478	2.57975E-07	0.006827121	7.67545E-05	-1.512198375	-0.575281713	0.936916662
165	HBB	P68871	hemoglobin subunit beta	Iron Dextran; Zinc; 4-Carboxycinnamic Acid; Pentaerythritol tetranitrate; 2-[(2-methoxy-5-methylphenoxy)methyl]pyridine; 4-[(5-methoxy-2-methylphenoxy)methyl]pyridine; Sebacic acid; 2-[4-({[(3,5-DICHLOROPHENYL)AMINO]CARBONYL}AMINO)PHENOXY]-2-METHYLPROPANOIC ACID; 2,6-dicarboxynaphthalene; Efaproxiral; Trimesic acid; Nitrous acid; Copper; Ferric pyrophosphate; Sodium ferric gluconate complex; Ferric pyrophosphate citrate; Zinc	yes	no	no	no	no	no	0.004185705	0.30624238	0.003943254	0.003207081	0.207029285	0.003153531	-1.217562225	-0.448514231	0.769047995
166	HBA1	P69905	hemoglobin subunit alpha 1		yes	no	no	no	no	no	0.001629375	0.404510403	0.000410239	0.001208558	0.298388281	0.000308748	-1.343064799	-0.364908247	0.978156552
167	SRSF3	P84103	serine and arginine rich splicing factor 3		no	no	yes	no	no	no	0.036583481	9.20893E-06	0.014706621	0.030516624	3.48394E-07	0.012294389	-0.676309571	-1.378308463	-0.701998891
168	EWSR1	Q01844	EWS RNA binding protein 1		no	no	yes	no	no	no	0.012641822	0.00023939	0.080849554	0.010110021	2.66779E-05	0.07198431	-0.803036116	-1.243949178	-0.440913062
169	TAGLN	Q01995	transgelin	Artenimol	no	yes	no	no	no	no	1.41809E-09	0.000122256	0.000141228	5.99491E-10	1.12451E-05	0.000103601	1.358245322	0.693170127	-0.665075195
170	DSG1	Q02413	desmoglein 1		no	yes	no	no	no	no	3.52388E-05	0.290604227	1.12687E-08	2.20821E-05	0.193955608	6.44366E-09	1.258003841	0.333888858	-0.924114983
171	RPL6	Q02878	ribosomal protein L6		yes	no	no	no	no	no	0.000522495	0.469555156	0.000388813	0.000366244	0.359985194	0.000292094	-1.109950712	-0.216814624	0.893136088
172	CFHR3	Q02985	complement factor H related 3		no	yes	no	no	no	no	3.60779E-09	0.000957932	0.000102163	1.60365E-09	0.000162766	7.42962E-05	1.715774582	0.932324558	-0.783450024
173	TNFAIP2	Q03169	TNF alpha induced protein 2		no	no	no	no	no	yes	9.29095E-05	0.286525981	2.98501E-11	6.06627E-05	0.190454741	1.49589E-11	-0.906818611	0.284767858	1.191586468
174	LMNB2	Q03252	lamin B2		no	yes	no	no	no	no	1.13164E-08	0.000110938	0.00440478	5.26081E-09	1.00635E-05	0.003534602	1.206961149	0.834558847	-0.372402302
175	CFHR1	Q03591	complement factor H related 1		no	no	no	no	yes	no	1.09262E-05	0.3245782	6.50932E-10	6.55067E-06	0.221925013	3.47439E-10	0.911507853	-0.202764084	-1.114271937
176	TLE3	Q04726	TLE family member 3, transcriptional corepressor		no	yes	no	no	no	no	6.24364E-09	0.000434096	0.043929018	2.84033E-09	5.60568E-05	0.037997506	1.200971379	0.825366336	-0.375605042
177	DPT	Q07507	dermatopontin		no	yes	no	no	no	no	6.86009E-08	0.313677824	8.81772E-09	3.45646E-08	0.213335031	5.00616E-09	1.193306132	0.210420907	-0.982885224
178	TGM3	Q08188	transglutaminase 3	5'-Guanosine-Diphosphate-Monothiophosphate; Guanosine-5'-Monophosphate; B-2-Octylglucoside;	no	yes	no	no	no	no	2.56592E-07	0.005193483	0.000576733	1.35679E-07	0.001254248	0.00043745	1.393870076	0.802891253	-0.590978823
179	LGALS3BP	Q08380	galectin 3 binding protein		no	no	no	no	no	yes	0.002375764	0.004569216	2.47493E-07	0.001781554	0.001080712	1.53071E-07	-0.875217015	0.616451658	1.491668673
180	ASPH	Q12797	aspartate beta-hydroxylase	Aspartic acid	no	yes	no	no	no	no	4.6619E-07	0.010294811	0.000221424	2.51578E-07	0.002957368	0.000164237	1.068620009	0.521867849	-0.54675216
181	TRDN	Q13061	triadin		no	yes	no	no	no	no	1.31755E-07	0.061689983	1.36327E-05	6.81164E-08	0.027840155	9.35819E-06	1.154142459	0.38062017	-0.773522289
182	PLA2G7	Q13093	phospholipase A2 group VII	Rilapladib; (1R)-1,2,2-trimethylpropyl (R)-methylphosphinate	no	no	no	no	yes	no	5.227E-07	0.037402582	1.75416E-14	2.83968E-07	0.015032211	7.51896E-15	0.940986681	-0.39523616	-1.336222842
183	SPP2	Q13103	secreted phosphoprotein 2		no	no	no	no	yes	no	0.004978852	0.039604359	5.46359E-16	0.003855396	0.016268802	2.20078E-16	0.808599163	-0.636707042	-1.445306205
184	CBX3	Q13185	chromobox 3		no	no	no	no	no	yes	1.97642E-05	0.088920364	2.7579E-10	1.21344E-05	0.043714814	1.44705E-10	-0.819596243	0.348465568	1.168061811
185	DNAJC3	Q13217	DnaJ heat shock protein family (Hsp40) member C3		no	no	no	no	yes	no	4.82506E-06	0.000274485	3.9935E-18	2.81589E-06	3.15901E-05	1.45662E-18	0.625332897	-0.507403161	-1.132736058
186	SRSF9	Q13242	serine and arginine rich splicing factor 9		yes	no	no	no	no	no	3.44239E-08	0.000675018	0.00381019	1.68298E-08	0.000104054	0.003041937	-1.247494625	-0.658283511	0.589211114
187	SF3B2	Q13435	splicing factor 3b subunit 2		no	no	no	no	no	yes	0.857303707	0.078717178	2.3205E-08	0.849146309	0.037450402	1.36055E-08	-0.086416403	0.985717714	1.072134118
188	OS9	Q13438	OS9 endoplasmic reticulum lectin		no	yes	no	no	no	no	1.04385E-12	2.35302E-05	0.000227652	2.54932E-13	1.19992E-06	0.00016896	1.309528689	0.762793797	-0.546734892
189	FKBP5	Q13451	FKBP prolyl isomerase 5		yes	no	no	no	no	no	2.03855E-10	0.018863053	1.85542E-06	7.82354E-11	0.006358909	1.20892E-06	-1.355239584	-0.522149255	0.833090329
190	HDAC1	Q13547	histone deacetylase 1	Arsenic trioxide; Zinc; Vorinostat; Belinostat; Pracinostat; Romidepsin; Panobinostat; Fingolimod; Mocetinostat; Abexinostat; Zinc acetate; Zinc chloride; Zinc sulfate, unspecified form	yes	no	no	no	no	no	5.46467E-11	0.003371329	8.72358E-05	1.90904E-11	0.000736285	6.31244E-05	-1.404414634	-0.624114212	0.780300422
191	CAMK2B	Q13554	calcium/calmodulin dependent protein kinase II beta	[4-({4-[(5-cyclopropyl-1H-pyrazol-3-yl)amino]-6-(methylamino)pyrimidin-2-yl}amino)phenyl]acetonitrile; Fostamatinib	no	no	no	no	no	yes	0.00027971	0.030994473	3.1728E-11	0.000190867	0.011782675	1.59431E-11	-0.742080185	0.459529908	1.201610093
192	APOF	Q13790	apolipoprotein F		no	yes	no	no	no	no	3.78051E-11	3.7498E-05	1.24285E-05	1.29329E-11	2.35851E-06	8.50902E-06	1.369090086	0.794555518	-0.574534568
193	SPTAN1	Q13813	spectrin alpha, non-erythrocytic 1		no	no	no	no	no	yes	0.001472502	0.292855539	1.91144E-07	0.001087529	0.196235576	1.177E-07	-0.965504444	0.307180608	1.272685052
194	PKP1	Q13835	plakophilin 1		no	yes	no	no	no	no	1.57789E-05	0.004561895	0.011256077	9.57315E-06	0.001076913	0.009318012	1.075684474	0.745119264	-0.33056521
195	DOCK1	Q14185	dedicator of cytokinesis 1		yes	no	no	no	no	no	1.82257E-07	0.025459348	0.052944208	9.50511E-08	0.009193974	0.046203237	-1.180573985	-0.708608427	0.471965558
196	RCAN2	Q14206	regulator of calcineurin 2		no	no	no	no	no	yes	2.43501E-05	0.129649018	4.63838E-09	1.50712E-05	0.070728327	2.58715E-09	-0.922681353	0.346188997	1.26887035
197	GAS6	Q14393	growth arrest specific 6		no	yes	no	no	no	no	0.000100874	0.037906148	0.000820366	6.60916E-05	0.015320473	0.000627076	1.113036711	0.579076196	-0.533960515
198	DIP2A	Q14689	disco interacting protein 2 homolog A		no	no	no	no	no	yes	5.22135E-08	0.027427808	1.66024E-11	2.58584E-08	0.01017824	8.22975E-12	-0.882729553	0.401146966	1.283876519
199	GPNMB	Q14956	glycoprotein nmb	Glembatumumab vedotin	no	yes	no	no	no	no	0.000643438	0.021519295	0.042657479	0.000455684	0.007517615	0.036858999	1.264683058	0.87493091	-0.389752147
200	PAFAH1B3	Q15102	platelet activating factor acetylhydrolase 1b catalytic subunit 3	(1R)-1,2,2-trimethylpropyl (R)-methylphosphinate	no	no	no	no	no	yes	0.000169459	0.05715528	7.98018E-09	0.000113178	0.025508813	4.50535E-09	-0.814315337	0.414550907	1.228866244
201	PDGFRL	Q15198	platelet derived growth factor receptor like		no	yes	no	no	no	no	5.69096E-16	0.001445916	6.81347E-07	3.79053E-17	0.00026075	4.31592E-07	1.426446063	0.579748796	-0.846697267
202	PPP1R7	Q15435	protein phosphatase 1 regulatory subunit 7		no	no	no	no	no	yes	2.97621E-05	0.0010974	1.07219E-22	1.86098E-05	0.000191933	3.00232E-23	-0.98681665	0.824844308	1.811660958
203	CST6	Q15828	cystatin E/M		no	yes	no	no	no	no	2.74384E-07	0.254564338	1.8083E-08	1.45584E-07	0.163788563	1.0545E-08	1.082537129	0.21968525	-0.862851879
204	ADIPOQ	Q15848	adiponectin, C1Q and collagen domain containing		no	no	no	yes	no	no	0.046372482	0.000859211	0.000316252	0.03914451	0.000140541	0.000236436	0.509082458	1.012361627	0.503279168
205	MIA	Q16674	MIA SH3 domain containing		no	no	no	no	yes	no	1.69244E-06	0.024485655	2.46578E-14	9.53961E-07	0.008809067	1.06363E-14	0.97158582	-0.452490821	-1.424076641
206	HAGH	Q16775	hydroxyacylglutathione hydrolase	Glutathione; S-(N-hydroxy-N-bromophenylcarbamoyl)glutathione	yes	no	no	no	no	no	0.000151024	0.107753042	0.000474677	0.000100318	0.055707395	0.00035875	-1.082492922	-0.47665805	0.605834872
207	H2AC20	Q16777	H2A clustered histone 20		no	no	no	yes	no	no	0.424743582	0.000222993	0.142652953	0.397800174	2.44514E-05	0.129273179	0.431653798	1.163799302	0.732145504
208	KRT71	Q3SY84	keratin 71		no	yes	no	no	no	no	1.12696E-07	0.021955192	0.000449173	5.79052E-08	0.007724609	0.000338661	1.604641027	0.732398636	-0.872242391
209	SVEP1	Q4LDE5	sushi, von Willebrand factor type A, EGF and pentraxin domain containing 1		no	no	no	no	no	yes	0.006042821	0.172921711	9.68501E-08	0.004717617	0.10130846	5.87156E-08	-0.892452477	0.518012172	1.410464648
210	FNDC1	Q4ZHG4	fibronectin type III domain containing 1		no	no	no	no	yes	no	2.45066E-05	0.706538099	3.46852E-08	1.51792E-05	0.612738524	2.05094E-08	0.966305477	-0.084474427	-1.050779904
211	ASPRV1	Q53RT3	aspartic peptidase retroviral like 1		no	yes	no	no	no	no	0.001153522	0.795763558	3.2698E-07	0.000839923	0.721848048	2.03566E-07	1.098368725	0.107354516	-0.991014209
212	MIA3	Q5JRA6	MIA SH3 domain ER export factor 3		no	yes	no	no	no	no	1.51448E-06	0.008813426	8.37211E-07	8.51597E-07	0.002439965	5.32599E-07	1.56655122	0.833991914	-0.732559306
213	ACBD5	Q5T8D3	acyl-CoA binding domain containing 5		no	no	no	no	yes	no	0.000159487	0.266583792	1.22669E-07	0.000106084	0.174282038	7.49797E-08	0.811600694	-0.253207645	-1.064808338
214	BROX	Q5VW32	BRO1 domain and CAAX motif containing		yes	no	no	no	no	no	4.592E-11	0.052330133	1.14375E-09	1.58546E-11	0.023047073	6.17738E-10	-1.291934163	-0.339259875	0.952674288
215	CAVIN1	Q6NZI2	caveolae associated protein 1		no	no	no	no	no	yes	9.78933E-06	0.000277521	1.51611E-13	5.8461E-06	3.23166E-05	6.84893E-14	-0.952881574	0.784499101	1.737380675
216	LLGL2	Q6P1M3	LLGL scribble cell polarity complex component 2		no	no	no	no	yes	no	3.88061E-06	0.097621541	3.21306E-11	2.24186E-06	0.049275214	1.61599E-11	0.997364596	-0.365093003	-1.362457599
217	SBSN	Q6UWP8	suprabasin		no	no	no	no	yes	no	0.000718458	0.000466975	2.65187E-13	0.000511743	6.28334E-05	1.20758E-13	0.551329719	-0.59436786	-1.145697579
218	PLXDC2	Q6UX71	plexin domain containing 2		no	no	no	no	no	yes	0.001955469	0.008731104	3.76123E-09	0.001458406	0.002409262	2.08427E-09	-0.868005397	0.660340355	1.528345752
219	ANGPTL8	Q6UXH0	angiopoietin like 8		no	yes	no	no	no	no	9.90937E-05	0.037065146	0.012952078	6.48801E-05	0.014863006	0.010780683	1.027884978	0.481472661	-0.546412317
220	ADAMTSL4	Q6UY14	ADAMTS like 4		no	yes	no	no	no	no	6.05739E-09	0.003800032	3.06639E-05	2.75119E-09	0.000852295	2.15356E-05	1.073547193	0.470169521	-0.603377672
221	THSD4	Q6ZMP0	thrombospondin type 1 domain containing 4		no	yes	no	no	no	no	3.55868E-09	0.000156942	0.003609014	1.5802E-09	1.52888E-05	0.002874783	1.245469753	0.729245745	-0.516224008
222	NCCRP1	Q6ZVX7	NCCRP1, F-box associated domain containing		no	yes	no	no	no	no	9.0303E-07	0.057194519	0.000255285	5.0082E-07	0.025560333	0.000189816	1.048191659	0.417723133	-0.630468525
223	DHX30	Q7L2E3	DExH-box helicase 30		no	yes	no	no	no	no	1.09708E-11	0.002837437	1.60253E-07	3.37029E-12	0.00059809	9.83881E-08	1.339515333	0.54251283	-0.797002503
224	HDGFL2	Q7Z4V5	HDGF like 2		yes	no	no	no	no	no	8.89201E-07	0.219573433	0.000275639	4.92747E-07	0.135604254	0.000205574	-1.038650125	-0.305706354	0.732943771
225	ARMC8	Q8IUR7	armadillo repeat containing 8		no	no	yes	no	no	no	0.00263747	0.000640648	0.038003854	0.00199095	9.68259E-05	0.03266575	-0.740286789	-1.142202447	-0.401915657
226	AEBP1	Q8IUX7	AE binding protein 1		no	yes	no	no	no	no	1.75342E-05	0.066191303	0.003578074	1.07096E-05	0.030381418	0.002848516	1.006700214	0.447233117	-0.559467096
227	ARHGAP12	Q8IWW6	Rho GTPase activating protein 12		no	yes	no	no	no	no	0.000638982	0.032698102	0.053722872	0.000451949	0.012652551	0.046907103	1.043418572	0.73092766	-0.312490912
228	SCUBE1	Q8IWY4	signal peptide, CUB domain and EGF like domain containing 1		no	yes	no	no	no	no	5.75103E-08	0.001152852	0.001054935	2.85858E-08	0.000202691	0.000812592	1.574203164	0.911466057	-0.662737107
229	CACNA2D3	Q8IZS8	calcium voltage-gated channel auxiliary subunit alpha2delta 3	Amlodipine; Nilvadipine	no	no	no	no	yes	no	2.87175E-08	0.168874044	5.02724E-10	1.39099E-08	0.098190764	2.66737E-10	0.924827407	-0.171723891	-1.096551299
230	GALNT16	Q8N428	polypeptide N-acetylgalactosaminyltransferase 16		no	no	no	no	yes	no	1.69668E-07	0.391199714	6.11227E-10	8.82553E-08	0.283920516	3.25138E-10	0.978634727	-0.129312134	-1.107946861
231	SFRP1	Q8N474	secreted frizzled related protein 1		no	yes	no	no	no	no	1.75221E-05	0.093476568	1.48545E-06	1.06943E-05	0.04669265	9.63155E-07	1.510397735	0.571342587	-0.939055148
232	SUMF2	Q8NBJ7	sulfatase modifying factor 2		no	no	no	no	no	yes	0.001616348	0.000310849	1.36945E-13	0.001198162	3.69019E-05	6.17399E-14	-0.498793395	0.699927289	1.198720685
233	PCSK9	Q8NBP7	proprotein convertase subtilisin/kexin type 9	Alirocumab	no	yes	no	no	no	no	3.64279E-07	0.007740347	0.000255594	1.95262E-07	0.002044686	0.000190161	1.017038986	0.521008952	-0.496030033
234	AGR3	Q8TD06	anterior gradient 3, protein disulphide isomerase family member		no	yes	no	no	no	no	1.82544E-05	0.328029638	7.74639E-05	1.11578E-05	0.225236809	5.59832E-05	1.024890752	0.249644154	-0.775246598

235	TSKU	Q8WUA8	tsukushi, small leucine rich proteoglycan		no	no	no	yes	no	no	0.308685946	0.000201119	0.000669039	0.283090328	2.12328E-05	0.000508373	0.330257568	1.248537529	0.91827996
236	MADD	Q8WXG6	MAP kinase activating death domain		yes	no	no	no	no	no	1.76273E-08	0.027438493	0.003665511	8.37039E-09	0.010194637	0.002924769	-1.407591957	-0.659663129	0.747928827
237	TTN	Q8WZ42	titin		no	no	no	no	yes	no	4.07971E-05	0.344762719	1.96263E-06	2.57131E-05	0.239474965	1.28144E-06	0.816397543	-0.278869708	-1.095267251
238	H1-10	Q92522	H1.10 linker histone		no	no	no	no	no	yes	0.048116318	0.561692847	0.009762474	0.04069999	0.456582224	0.008028495	-0.867028743	0.176328336	1.043357079
239	NCSTN	Q92542	nicastrin		no	no	no	no	no	yes	0.000876167	0.39492847	5.94764E-05	0.00063003	0.287520169	4.26063E-05	-0.996201621	0.17778547	1.17398709
240	HSPH1	Q92598	heat shock protein family H (Hsp110) member 1	Phenethyl Isothiocyanate	no	no	no	no	no	yes	0.023085691	0.210315732	3.30257E-08	0.018922526	0.12931511	1.94832E-08	-0.647243872	0.433746814	1.080990686
241	SEPTIN8	Q92599	septin 8		no	no	no	no	no	yes	2.94131E-05	0.162179091	2.08968E-11	1.83515E-05	0.093269514	1.03963E-11	-0.940776894	0.357905335	1.298682229
242	DHX38	Q92620	DEAH-box helicase 38		no	no	no	no	no	yes	0.006059095	0.546136682	1.46257E-08	0.004733067	0.440720177	8.45598E-09	-0.836441414	0.214584404	1.051025818
243	FRZB	Q92765	frizzled related protein		no	yes	no	no	no	no	1.22227E-07	0.006297874	0.000146551	6.29688E-08	0.001583743	0.000107706	1.767118029	0.85666604	-0.910451989
244	DDX17	Q92841	DEAD-box helicase 17		yes	no	no	no	no	no	1.78853E-08	0.100162841	6.2196E-06	8.50101E-09	0.051102564	4.16237E-06	-1.206405033	-0.31174534	0.894659693
245	ATP6V0A1	Q93050	ATPase H+ transporting V0 subunit a1	Tiludronic acid	no	no	yes	no	no	no	0.000877672	9.69859E-08	0.096612229	0.00063151	8.34949E-10	0.086281243	-0.918353766	-1.339839529	-0.421485763
246	RNASE6	Q93091	ribonuclease A family member 6		no	yes	no	no	no	no	3.69689E-07	0.129522197	8.88997E-05	1.98329E-07	0.070541767	6.43687E-05	1.172534679	0.373498107	-0.799036572
247	FERMT2	Q96AC1	FERM domain containing kindlin 2		no	no	no	no	no	yes	0.011853557	4.07042E-05	3.75264E-18	0.009452768	2.63874E-06	1.36707E-18	-0.441135294	0.873929983	1.315065277
248	PBXIP1	Q96AQ6	PBX homeobox interacting protein 1		no	no	no	no	yes	no	5.06528E-07	0.940656356	5.03128E-12	2.74723E-07	0.913449052	2.43015E-12	0.987087613	-0.0162752	-1.003362813
249	CYFIP2	Q96F07	cytoplasmic FMR1 interacting protein 2		no	no	no	no	no	yes	8.47652E-05	0.021955192	9.69255E-08	5.52683E-05	0.007715317	5.88053E-08	-0.707624881	0.597427309	1.30505219
250	SFRP2	Q96HF1	secreted frizzled related protein 2		no	no	no	no	yes	no	0.000308844	0.007956699	1.09222E-12	0.000211866	0.002141495	5.15182E-13	0.898965305	-0.645893389	-1.544858694
251	CNDP1	Q96KN2	carnosine dipeptidase 1		no	no	yes	no	no	no	0.011131531	0.000506314	0.057629663	0.008861849	7.15768E-05	0.050501028	-0.913015115	-1.390049139	-0.477034023
252	JSRP1	Q96MG2	junctional sarcoplasmic reticulum protein 1		no	yes	no	no	no	no	6.93957E-06	0.199340694	2.3409E-06	4.09394E-06	0.120489572	1.53373E-06	1.320823103	0.384322006	-0.936501097
253	PGLYRP2	Q96PD5	peptidoglycan recognition protein 2	Zinc; Copper; Zinc acetate; Zinc chloride; Zinc sulfate, unspecified form	no	no	yes	no	no	no	0.000734343	5.95016E-06	3.88388E-07	0.00052339	1.69851E-07	2.43556E-07	-0.790225066	-1.3890438	-0.598818733
254	HHIP	Q96QV1	hedgehog interacting protein		no	no	no	no	yes	no	1.48188E-06	0.017031226	9.65765E-13	8.3259E-07	0.005610195	4.53345E-13	0.770714787	-0.326309986	-1.097024774
255	VPS13A	Q96RL7	vacuolar protein sorting 13 homolog A		no	no	no	no	no	yes	0.120306343	0.021039466	6.8854E-08	0.105206725	0.007273726	4.13062E-08	-0.783501371	1.589078709	2.37258008
256	MMP19	Q99542	matrix metallopeptidase 19	Marimastat	no	no	no	yes	no	no	0.00070637	2.33059E-06	0.000941195	0.000502812	5.80801E-08	0.000721995	0.775855956	1.290329296	0.514473341
257	PIK3R4	Q99570	phosphoinositide-3-kinase regulatory subunit 4		no	yes	no	no	no	no	2.32789E-16	0.000480826	3.54607E-12	1.22397E-17	6.66664E-05	1.69832E-12	1.241658123	0.403369293	-0.83828883
258	SOST	Q9BQB4	sclerostin	Romosozumab	no	yes	no	no	no	no	2.05287E-08	0.036961638	5.45264E-05	9.80392E-09	0.014758771	3.88875E-05	1.127070728	0.424566514	-0.702504214
259	APOL2	Q9BQE5	apolipoprotein L2		no	no	no	no	yes	no	0.054359459	0.136473814	1.12738E-07	0.04613288	0.075379057	6.8603E-08	0.649810548	-0.582250551	-1.2320611
260	SPON2	Q9BUD6	spondin 2		no	yes	no	no	no	no	1.19124E-07	8.58601E-05	0.077896234	6.1316E-08	7.00263E-06	0.06917835	1.071870496	0.751736929	-0.320133567
261	MENT	Q9BUN1	chromosome 1 open reading frame 56		no	no	no	no	yes	no	8.28297E-06	0.390750141	4.03766E-09	4.91649E-06	0.283280574	2.24111E-09	0.913537762	-0.163758386	-1.077296148
262	CHID1	Q9BWS9	chitinase domain containing 1		no	yes	no	no	no	no	6.60385E-08	0.092589489	2.19864E-11	3.31838E-08	0.0459801	1.09583E-11	1.258186261	0.368179656	-0.890006605
263	ASPN	Q9BXN1	asporin		no	yes	no	no	no	no	3.14255E-06	0.008109459	0.003518463	1.80124E-06	0.002197307	0.002799466	1.263197088	0.618224218	-0.64497287
264	ANGPTL4	Q9BY76	angiopoietin like 4		no	yes	no	no	no	no	2.13281E-09	0.000927781	1.63852E-06	9.171E-10	0.000156883	1.06388E-06	1.823778627	0.854827935	-0.968950692
265	REG4	Q9BYZ8	regenerating family member 4		no	yes	no	no	no	no	2.87981E-09	0.000204822	0.000132216	1.25788E-09	2.17165E-05	9.67509E-05	1.672811123	0.909680257	-0.763130866
266	FAM107B	Q9H098	family with sequence similarity 107 member B		yes	no	no	no	no	no	3.47633E-11	0.000659742	2.7595E-05	1.17348E-11	0.000101338	1.93303E-05	-1.737637592	-0.864171489	0.873466102
267	SIL1	Q9H173	SIL1 nucleotide exchange factor		no	yes	no	no	no	no	1.032E-06	0.093031464	4.19181E-08	5.74683E-07	0.046283891	2.49001E-08	1.213743814	0.38713731	-0.826606505
268	CLEC4M	Q9H2X3	C-type lectin domain family 4 member M		no	yes	no	no	no	no	1.04975E-07	0.009973361	0.000131697	5.3653E-08	0.002833392	9.6311E-05	1.00487218	0.437182907	-0.567689273
269	DOCK5	Q9H7D0	dedicator of cytokinesis 5		yes	no	no	no	no	no	1.38749E-08	0.025969314	0.002995197	6.50682E-09	0.00946999	0.002372272	-1.61627744	-0.799934965	0.816342474
270	UBE2Z	Q9H832	ubiquitin conjugating enzyme E2 Z		yes	no	no	no	no	no	0.000108884	0.258013903	0.012667487	7.15367E-05	0.16635876	0.010526584	-1.029367856	-0.407378776	0.621989079
271	MMP25	Q9NPA2	matrix metallopeptidase 25	Marimastat; Fenofibrate; Fenofibric acid	no	no	no	no	yes	no	0.021278513	0.015836664	4.10489E-08	0.017393039	0.005123416	2.43466E-08	0.679562161	-0.708221682	-1.387783843
272	CRTAC1	Q9NQ79	cartilage acidic protein 1		yes	no	no	no	no	no	4.30064E-06	0.00049887	0.264968306	2.5001E-06	6.98464E-05	0.244918597	-1.064059406	-0.84382576	0.220233646
273	MXRA5	Q9NR99	matrix remodeling associated 5		no	yes	no	no	no	no	0.000299747	0.285752287	3.68386E-06	0.000205355	0.189810989	2.43699E-06	1.218020322	0.374237147	-0.843783174
274	STRN4	Q9NRL3	striatin 4		no	yes	no	no	no	no	5.27132E-06	0.011564417	0.061573419	3.08827E-06	0.003442602	0.054152245	2.284685065	1.531230959	-0.753454106
275	DDX19A	Q9NUU7	DEAD-box helicase 19A		no	no	no	no	no	yes	0.041830599	0.077125072	8.03918E-13	0.035139977	0.036273595	3.74822E-13	-0.849372213	0.871036627	1.72040884
276	SDAD1	Q9NVU7	SDA1 domain containing 1		no	no	no	no	no	yes	0.044034823	0.051546217	1.09341E-09	0.037071455	0.022585044	5.89066E-10	-0.590500188	0.721290747	1.311790936
277	CCDC88C	Q9P219	coiled-coil domain containing 88C		no	no	no	no	yes	no	0.000219308	0.112432402	2.81074E-09	0.00014816	0.059094511	1.55247E-09	0.653458664	-0.355984418	-1.009443082
278	DIP2B	Q9P265	disco interacting protein 2 homolog B		no	no	no	no	no	yes	0.002702446	0.000152858	5.16233E-19	0.002041222	1.48218E-05	1.81746E-19	-0.604589546	0.943042686	1.547632232
279	DNAJB11	Q9UBS4	DnaJ heat shock protein family (Hsp40) member B11		no	no	no	no	no	yes	0.001243956	0.001537031	1.33051E-13	0.000910844	0.00028136	5.99241E-14	-0.760132313	0.895685388	1.6558177
280	GHRL	Q9UBU3	ghrelin and obestatin prepropeptide		no	no	no	no	yes	no	0.000271719	6.56872E-05	3.25004E-13	0.000185045	5.0443E-06	1.4888E-13	0.818321549	-0.997666607	-1.815988156
281	SEL1L	Q9UBV2	SEL1L adaptor subunit of SYVN1 ubiquitin ligase		no	no	no	no	no	yes	0.095494994	0.021482956	0.000129857	0.082730598	0.007495186	9.49068E-05	-0.464345075	0.536236666	1.00058174
282	KLK11	Q9UBX7	kallikrein related peptidase 11		no	no	no	no	yes	no	0.001378824	0.052047169	8.98972E-06	0.001016469	0.022875285	6.06918E-06	0.932739491	-0.519946459	-1.45268595
283	CIDEB	Q9UHD4	cell death inducing DFFA like effector b		no	no	no	no	yes	no	0.017179534	0.026644339	1.96932E-06	0.013917991	0.009815064	1.2867E-06	0.525421893	-0.478586099	-1.004007992
284	CD84	Q9UIB8	CD84 molecule		no	no	no	no	no	yes	0.180129006	0.085321677	2.32298E-07	0.161193832	0.041404402	1.43357E-07	-0.910953849	1.429119135	2.340072984
285	ANAPC5	Q9UJX4	anaphase promoting complex subunit 5		no	yes	no	no	no	no	3.84121E-13	0.007587078	1.03096E-05	8.07577E-14	0.0019677	7.01164E-06	1.108133241	0.435118674	-0.673014567
286	PCOLCE2	Q9UKZ9	procollagen C-endopeptidase enhancer 2		no	yes	no	no	no	no	2.2917E-07	0.040814	9.03642E-06	1.20452E-07	0.016884088	6.1089E-06	1.150111053	0.407644665	-0.742466387
287	AGO1	Q9UL18	argonaute RISC component 1		no	no	no	no	no	yes	0.003394973	0.000866311	9.18818E-09	0.00257661	0.000142488	5.22898E-09	-0.523003483	0.805539488	1.328542971
288	MINPP1	Q9UNW1	multiple inositol-polyphosphate phosphatase 1		no	no	no	no	yes	no	0.00443004	0.001833454	2.8109E-13	0.003404326	0.000346421	1.28382E-13	0.632254896	-0.743416167	-1.375671062
289	THRAP3	Q9Y2W1	thyroid hormone receptor associated protein 3		no	no	yes	no	no	no	0.000171582	4.14048E-05	0.654512203	0.000114751	2.7203E-06	0.635235677	-0.967067163	-1.054975931	-0.087908768
290	HECTD4	Q9Y4D8	None	None	no	no	no	no	no	yes	0.017316528	0.007587078	8.37444E-16	0.014036823	0.001971652	3.41883E-16	-0.636356402	0.826731396	1.463087798
291	NUB1	Q9Y5A7	negative regulator of ubiquitin like proteins 1		no	no	no	no	no	yes	0.002969497	0.372879623	2.92748E-07	0.002248314	0.265256461	1.81724E-07	-0.769991691	0.243493567	1.013485258
292	TNPO3	Q9Y5L0	transportin 3		no	no	no	no	no	yes	0.321615626	0.011123437	3.72195E-08	0.295676531	0.003250846	2.20248E-08	-0.300038862	0.940629554	1.240668416
293	HS3ST3B1	Q9Y662	heparan sulfate-glucosamine 3-sulfotransferase 3B1		no	yes	no	no	no	no	5.28639E-14	1.0703E-07	0.000413823	8.86254E-15	1.0669E-09	0.000311633	1.330269776	0.876932795	-0.453336981
294	COLEC10	Q9Y6Z7	collectin subfamily member 10		no	no	no	no	yes	no	0.172918136	0.356685688	4.40507E-07	0.15434922	0.249623766	2.77038E-07	0.623178873	-0.503757413	-1.126936286
295	IGLV3-10	A0A075B6K4	None	None	no	yes	no	no	yes	no	0.000853485	0.304765931	8.21468E-08	0.000613334	0.205754979	4.96156E-08	1.195212481	-0.396838683	-1.592051164
296	IGHV6-1	A0A0B4J1U7	None	None	no	yes	no	no	yes	no	0.008706652	0.975208345	0.00087189	0.006880109	0.966370934	0.000667646	1.00757657	-0.013368568	-1.020945138
297	IGHV3-72	A0A0B4J1Y9	None	None	no	no	yes	no	yes	no	0.083324841	0.001584823	5.15267E-09	0.071885137	0.000291544	2.88101E-09	0.480946511	-1.082375747	-1.563322258
298	IGKV3D-20	A0A0C4DH25	None	None	no	yes	no	yes	no	no	6.10318E-07	0.000223066	0.328148476	3.32951E-07	2.45605E-05	0.306886477	2.956681274	2.377102964	-0.57957831
299	IGHV1-18	A0A0C4DH31	None	None	no	yes	no	no	yes	no	0.000299805	0.970357442	3.93143E-05	0.00020553	0.956172696	2.78246E-05	1.057242004	0.01539425	-1.041847754
300	IGKV3D-7	A0A0C4DH55	None	None	no	yes	no	yes	no	no	1.44582E-07	3.6219E-05	0.924779488	7.50751E-08	2.19907E-06	0.920589277	2.963624248	2.90007871	-0.063545538
301	C3orf85	A0A1B0GTC6	None	None	no	no	yes	no	yes	no	0.116294328	2.85043E-06	3.08691E-09	0.101645563	7.23264E-08	1.70641E-09	0.358599939	-1.123604527	-1.482204466
302	ARHGAP10	A1A4S6	Rho GTPase activating protein 10		yes	no	no	no	no	yes	4.35805E-15	0.774867335	8.02618E-12	4.7589E-16	0.697204619	3.92763E-12	-2.816995575	0.133334492	2.950330067
303	PGP	A6NDG6	phosphoglycolate phosphatase		yes	no	no	no	no	yes	6.17856E-08	0.001084597	5.5742E-17	3.09068E-08	0.00018822	2.14684E-17	-1.006024226	0.680132458	1.686156684
304	TMEM212	A6NML5	transmembrane protein 212		no	no	yes	no	yes	no	0.18662534	2.35641E-05	6.78225E-14	0.167345513	1.22785E-06	3.00853E-14	0.371029327	-1.473693103	-1.84472243
305	SMIM1	B2RUZ4	small integral membrane protein 1 (Vel blood group)		yes	no	yes	no	no	no	4.2653E-07	0.000918859	0.003098216	2.29789E-07	0.000154462	0.002458077	-2.297603088	-1.404823193	0.892779895
306	WASH3P	C4AMC7	None	None	yes	no	no	no	no	yes	3.65685E-14	0.480723822	8.18762E-22	5.76613E-15	0.370943665	2.42253E-22	-2.315725456	0.178054727	2.493780184
307	NACA	E9PAV3	None	None	yes	no	no	no	no	yes	3.31962E-12	0.204839502	9.43677E-20	9.00974E-13	0.124370155	3.19405E-20	-2.676903573	-0.384017481	2.292886092
308	CNK3/IPCEF1	G9CGD6	None	None	yes	no	no	no	no	yes	7.69497E-12	0.11419554	7.94754E-14	2.28025E-12	0.060538641	3.53624E-14	-1.45610216	0.322896021	1.778998181
309	KIF2A	O00139	kinesin family member 2A		yes	no	no	no	no	yes	2.98001E-11	0.364978181	7.12999E-36	9.91088E-12	0.257981859	2.22913E-37	-4.595058901	0.461009652	5.056068553
310	MYO1C	O00159	myosin IC		yes	no	no	no	no	yes	4.74597E-13	0.444217908	9.976E-14	1.0365E-13	0.336333541	4.4614E-14	-1.785626177	0.165435186	1.951061364
311	SNAP23	O00161	synaptosome associated protein 23		yes	no	no	no	no	yes	1.62541E-14	0.020349026	1.24518E-28	2.2168E-15	0.006952046	1.88441E-29	-3.476394602	0.774192375	4.250586977
312	CCL24	O00175	C-C motif chemokine ligand 24		no	yes	no	yes	no	no	8.79113E-11	2.0276E-06	0.001691188	3.1946E-11	4.86917E-08	0.001316475	1.618740916	1.049338889	-0.569402027
313	GTPBP1	O00178	GTP binding protein 1		yes	no	no	no	no	yes	1.06133E-09	0.031377559	7.10175E-31	4.42902E-10	0.012070479	7.78715E-32	-2.605617948	0.752915768	3.358533717
314	STXBP3	O00186	syntaxin binding protein 3		yes	no	no	no	no	yes	1.19196E-11	0.972485538	3.16302E-30	3.69416E-12	0.96301507	3.86957E-31	-3.059381877	-0.012345788	3.047036089
315	AP3B1	O00203	adaptor related protein complex 3 subunit beta 1		yes	no	no	no	no	yes	2.9137E-16	0.418887708	5.39378E-27	1.63706E-17	0.311461137	1.01179E-27	-3.790052589	0.239498668	4.029551257
316	PSMD11	O00231	proteasome 26S subunit, non-ATPase 11		yes	no	no	no	no	yes	2.20614E-08	0.064736314	1.80159E-14	1.05559E-08	0.02962559	7.7386E-15	-1.831553463	0.660233451	2.491786914
317	PSMD9	O00233	proteasome 26S subunit, non-ATPase 9		yes	no	no	no	no	yes	4.3018E-09	0.173367909	6.31749E-21	1.92188E-09	0.101663905	1.98889E-21	-1.267509559	0.268615184	1.536124743
318	SUPT5H	O00267	SPT5 homolog, DSIF elongation factor subunit		yes	no	no	no	no	yes	7.33271E-10	0.306566297	5.28827E-17	2.99355E-10	0.207526075	2.03432E-17	-1.780087961	0.237736503	2.017824464
319	CLIC1	O00299	chloride intracellular channel 1	Copper; Artenimol	yes	no	no	no	no	yes	4.17207E-12	0.036399451	1.98316E-38	1.1588E-12	0.014444203	1.97687E-40	-3.976802454	0.831057951	4.807860405
320	EIF3F	O00303	eukaryotic translation initiation factor 3 subunit F	Quercetin	yes	no	no	no	no	yes	4.64165E-14	0.570828999	1.06984E-26	7.59237E-15	0.466595158	2.10381E-27	-3.482130561	-0.184700339	3.297430222
321	IPO5	O00410	importin 5		yes	no	no	no	no	yes	1.1221E-11	0.7950002	3.16187E-24	3.45731E-12	0.720074943	8.0372E-25	-3.044347762	-0.101564736	2.942783027
322	DNM1L	O00429	dynamin 1 like		yes	no	no	no	no	yes	7.01846E-10	0.373540083	2.55325E-18	2.84936E-10	0.265895546	9.25511E-19	-3.788175682	0.521620502	4.309796184
323	EXOC5	O00471	exocyst complex component 5		yes	no	no	no	no	yes	4.76366E-19	0.099566059	2.14079E-17	4.10102E-21	0.050707861	8.0704E-18	-2.198906384	0.402946589	2.601852974
324	BTN3A3	O00478	butyrophilin subfamily 3 member A3		yes	no	no	no	no	yes	2.18817E-11	0.431733319	2.14977E-13	7.08899E-12	0.323751084	9.75042E-14	-1.83413924	-0.177170962	1.656968277
325	BIN1	O00499	bridging integrator 1		no	no	yes	no	yes	no	0.025085084	0.000650365	8.96601E-15	0.020618189	9.90134E-05	3.81066E-15	0.667974741	-1.154907131	-1.822881872
326	CLDN5	O00501	claudin 5		yes	no	no	no	no	yes	1.09613E-13	0.044786751	3.06962E-25	2.01148E-14	0.018962461	7.10729E-26	-2.763758946	0.576910233	3.340669179
327	STK25	O00506	serine/threonine kinase 25		yes	no	no	no	no	yes	1.07949E-17	0.508948149	3.20045E-25	2.3967E-19	0.399733376	7.4537E-26	-3.455213846	0.199716463	3.654930309
328	SDCBP	O00560	syndecan binding protein		no	no	yes	no	yes	no	0.707257147	1.06581E-08	1.07539E-15	0.688798257	7.76152E-11	4.40485E-16	0.098886612	-2.03005704	-2.128943652
329	DDX3X	O00571	DEAD-box helicase 3 X-linked		yes	no	no	no	no	yes	5.97019E-14	0.300488203	8.72666E-26	1.00901E-14	0.202197887	1.90982E-26	-3.930146069	-0.377208012	3.552938057
330	PPP6C	O00743	protein phosphatase 6 catalytic subunit		yes	no	no	no	no	yes	2.32789E-16	0.007320256	2.47842E-29	1.25518E-17	0.001890596	3.43632E-30	-2.939553076	0.659103303	3.598656378
331	TXNDC9	O14530	thioredoxin domain containing 9		yes	no	no	no	no	yes	6.12865E-14	0.009576415	1.18004E-20	1.03857E-14	0.002707605	3.80691E-21	-2.802420577	0.879572979	3.681993556
332	EIF1AY	O14602	eukaryotic translation initiation factor 1A Y-linked		yes	no	no	no	no	yes	2.07859E-09	0.76673599	1.90478E-16	8.90957E-10	0.685789236	7.50864E-17	-1.897113056	0.094156944	1.99127
333	AP3D1	O14617	adaptor related protein complex 3 subunit delta 1		yes	no	no	no	no	yes	1.84613E-17	0.438322921	2.02408E-29	5.35354E-19	0.330678597	2.77887E-30	-3.735470649	0.221905806	3.957376454
334	ADAM10	O14672	ADAM metallopeptidase domain 10	XL784	yes	no	no	no	no	yes	1.60517E-08	0.000160048	3.04505E-17	7.57504E-09	1.5809E-05	1.15759E-17	-1.263330866	0.777594591	2.040925457

335	NHERF1	O14745	NHERF family PDZ scaffold protein 1		yes	no	no	no	no	yes	2.34523E-15	0.007173515	3.86855E-31	2.25278E-16	0.001842947	4.08416E-32	-2.657958989	0.645563061	3.30352205
336	APOL1	O14791	apolipoprotein L1	Zinc; Zinc acetate; Zinc chloride; Zinc sulfate, unspecified form	no	yes	no	no	yes	no	3.6337E-10	0.392923852	5.22585E-12	1.4357E-10	0.285746616	2.52886E-12	1.096628612	-0.099122792	-1.195751404
337	PSMA7	O14818	proteasome 20S subunit alpha 7	acetylleucyl-leucyl-norleucinal; (3AR,6R,6AS)-6-((S)-((S)-CYCLOHEX-2-ENYL)(HYDROXY)METHYL)-6A-METHYL-4-OXO-HEXAHYDRO-2H-FURO[3,2-C]PYRROLE-6-	yes	no	no	no	no	yes	3.88714E-10	0.156219338	6.62581E-29	1.54464E-10	0.089116514	9.72706E-30	-1.984755575	0.361262682	2.346018257
338	MYL12B	O14950	myosin light chain 12A	4-[4-(2,5-DIOXO-PYRROLIDIN-1-YL)-PHENYLAMINO]-4-HYDROXY-BUTYRIC ACID	yes	no	no	no	no	yes	1.3387E-10	0.770832284	8.97067E-22	4.94961E-11	0.691897941	2.66234E-22	-3.120693147	0.127196646	3.247889793
339	LECT2	O14960	leukocyte cell derived chemotaxin 2		no	yes	no	no	yes	no	0.000149201	0.360808654	2.93474E-12	9.90389E-05	0.254380728	1.40155E-12	1.446782399	-0.363462174	-1.810244573
340	PPP1R12A	O14974	protein phosphatase 1 regulatory subunit 12A		yes	no	no	no	no	yes	1.36012E-17	0.671870957	1.16956E-26	3.32789E-19	0.575367517	2.3105E-27	-3.384269962	-0.113865536	3.270404426
341	HNRNPDL	O14979	heterogeneous nuclear ribonucleoprotein D like		yes	no	no	no	no	yes	1.33245E-15	0.227324815	1.25458E-14	1.11088E-16	0.141936382	5.36054E-15	-2.050620033	-0.225661693	1.82495834
342	XPO1	O14980	exportin 1	Selinexor	yes	no	no	no	no	yes	3.00094E-19	2.74954E-05	1.34422E-30	2.03961E-21	1.55729E-06	1.55923E-31	-2.417498665	0.726214662	3.143713327
343	PLXNB2	O15031	plexin B2		yes	no	no	no	no	yes	2.92546E-08	0.002244568	6.97308E-21	1.41833E-08	0.000451558	2.21167E-21	-1.607118235	0.836426428	2.443544662
344	FYB1	O15117	FYN binding protein 1		yes	no	no	no	no	yes	3.32183E-14	0.987805867	1.58535E-31	5.0723E-15	0.98377766	1.53722E-32	-4.653997724	-0.006754197	4.647243527
345	SCAMP2	O15127	secretory carrier membrane protein 2		yes	no	no	no	no	yes	2.16708E-10	0.013619324	2.51772E-14	8.33642E-11	0.004220941	1.08717E-14	-2.720558478	0.71361446	3.434172938
346	PLSCR1	O15162	phospholipid scramblase 1		yes	no	no	no	no	yes	9.64587E-08	0.378138695	6.76461E-12	4.89942E-08	0.270196974	3.29495E-12	-1.489267154	0.227057799	1.716324952
347	PMM2	O15305	phosphomannomutase 2		yes	no	no	no	no	yes	2.53005E-06	0.014908399	7.96678E-21	1.43985E-06	0.004751535	2.53768E-21	-1.598974505	0.777449229	2.376423735
348	CHAD	O15335	chondroadherin		no	yes	no	no	yes	no	1.72913E-09	0.003116926	1.70437E-17	7.37249E-10	0.000668014	6.36339E-18	1.99048389	-0.788181516	-2.778665406
349	GPR37	O15354	G protein-coupled receptor 37		no	yes	no	no	yes	no	2.41811E-08	0.546214529	6.93769E-09	1.1603E-08	0.441277982	3.90107E-09	1.241090258	0.13767564	-1.103414618
350	EIF3D	O15371	eukaryotic translation initiation factor 3 subunit D		yes	no	no	no	no	yes	3.96899E-15	0.388895555	2.08118E-26	4.19539E-16	0.281055011	4.20573E-27	-3.933677297	-0.294675147	3.63900215
351	EIF3H	O15372	eukaryotic translation initiation factor 3 subunit H		yes	no	no	no	no	yes	2.282E-13	0.386600951	4.27518E-27	4.54952E-14	0.278696018	7.94211E-28	-3.542825458	-0.292898603	3.249926855
352	STX7	O15400	syntaxin 7		yes	no	yes	no	no	no	5.22344E-07	0.002768468	2.042E-06	2.83538E-07	0.000572008	1.33604E-06	-2.131308687	-1.228809385	0.902499302
353	ABCC4	O15439	ATP binding cassette subfamily C member 4 (PEL blood group)	ATP	yes	no	no	no	no	yes	1.85204E-05	0.09457252	2.54883E-08	1.13371E-05	0.047418736	1.50019E-08	-1.416291219	0.383357411	1.79964863
354	CCL16	O15467	C-C motif chemokine ligand 16		no	yes	no	no	yes	no	1.08314E-12	0.067028755	6.92136E-13	2.66491E-13	0.030826546	3.22077E-13	1.901912803	0.430884966	-1.471027837
355	YKT6	O15498	YKT6 v-SNARE homolog		yes	no	no	no	no	yes	9.46445E-15	0.028927229	3.81607E-21	1.16215E-15	0.010813305	1.18615E-21	-2.182720175	0.523884254	2.706604429
356	DDX3Y	O15523	DEAD-box helicase 3 Y-linked		yes	no	no	no	no	yes	2.99064E-16	0.717003634	7.30219E-27	1.70837E-17	0.625222332	1.39625E-27	-3.549175908	-0.117216071	3.431959837
357	PDPK1	O15530	3-phosphoinositide dependent protein kinase 1	Celecoxib; Inositol 1,3,4,5-Tetrakisphosphate; 7-Hydroxystaurosporine; Bisindolylmaleimide VIII; Staurosporine; Bisindolylmaleimide I; Dexfosfoserine; 10,11-dimethoxy-4-methyldibenzo[c,f]-2,7-naphthyridine-3,6-diamine; 5-HYDROXY-3-[(1R)-1-(1H-PYRROL-2-YL)ETHYL]-2H-INDOL-2-ONE; 1-{2-OXO-3-[(1R)-1-(1H-PYRROL-2-YL)ETHYL]-2H-INDOL-5-YL}UREA; 2-(1H-imidazol-1-yl)-9-methoxy-8-(2-methoxyethoxy)benzo[c][2,7]naphthyridin-4-amine; 3-(1H-indol-3-yl)-4-(1-{2-[(2S)-1-methylpyrrolidinyl]ethyl}-1H-indol-3-yl)-1H-pyrrole-2,5-dione; 3-[1-(3-	yes	no	no	no	no	yes	4.50447E-13	0.157502629	1.40697E-07	9.77635E-14	0.089935538	8.61265E-08	-1.31483475	-0.292862204	1.021972546
358	CLDN3	O15551	claudin 3		yes	no	no	no	no	yes	1.52665E-11	0.00412909	3.45674E-20	4.86286E-12	0.000952291	1.14337E-20	-2.582288964	0.867359753	3.449648718
359	ASAP2	O43150	ArfGAP with SH3 domain, ankyrin repeat and PH		yes	no	no	no	no	yes	4.08795E-13	0.431733319	1.74084E-11	8.74269E-14	0.323642685	8.64502E-12	-3.249070513	-0.368081696	2.880988817
360	SEPTIN4	O43236	septin 4		yes	no	no	no	no	yes	6.36099E-06	0.994512108	7.75947E-07	3.7382E-06	0.994512108	4.92922E-07	-2.066913587	0.003246929	2.070160516
361	PSMD3	O43242	proteasome 26S subunit, non-ATPase 3		yes	no	no	no	no	yes	4.68586E-15	0.012876957	7.74297E-28	5.20091E-16	0.00392085	1.32266E-28	-2.177068894	0.520782517	2.69785141
362	MTSS1	O43312	MTSS I-BAR domain containing 1		yes	no	no	no	no	yes	3.05043E-15	0.396830852	3.39626E-32	3.12368E-16	0.29020616	2.90844E-33	-4.407980712	0.318091882	4.726072594
363	PPIP5K2	O43314	diphosphoinositol pentakisphosphate kinase 2		yes	no	no	no	no	yes	2.6351E-12	0.116537595	2.19439E-23	7.04446E-13	0.062255471	5.89612E-24	-2.121941274	0.384199059	2.506140334
364	EEF1E1	O43324	eukaryotic translation elongation factor 1 epsilon 1		yes	no	no	no	no	yes	9.29321E-18	0.016264791	1.05976E-26	1.89486E-19	0.005306139	2.07919E-27	-2.472022961	0.436216774	2.908239734
365	TXNL1	O43396	thioredoxin like 1		yes	no	no	no	no	yes	4.60003E-12	0.002098873	8.43374E-20	1.29643E-12	0.000412737	2.83545E-20	-2.285788346	0.999113377	3.284901723
366	EIF4G3	O43432	eukaryotic translation initiation factor 4 gamma 3		yes	no	no	no	no	yes	1.84613E-17	0.005339303	1.03684E-23	4.85528E-19	0.001299142	2.71542E-24	-2.995947539	0.813255931	3.80920347
367	WIPF1	O43516	WAS/WASL interacting protein family member 1		yes	no	no	no	no	yes	3.52537E-14	0.846160642	1.67897E-29	5.44699E-15	0.784816871	2.27463E-30	-3.71255918	-0.074413731	3.638145449
368	DENR	O43583	density regulated re-initiation and release factor		yes	no	no	no	no	yes	3.832E-07	0.591189601	1.16926E-15	2.05924E-07	0.487255951	4.79464E-16	-1.671554041	-0.174771609	1.496782432
369	CHMP2A	O43633	charged multivesicular body protein 2A		yes	no	no	no	no	yes	4.11081E-12	0.080916876	1.36384E-27	1.13806E-12	0.038753574	2.3668E-28	-1.912034675	0.375969632	2.288004307
370	NCK2	O43639	NCK adaptor protein 2		yes	no	no	no	no	yes	2.47926E-14	0.019185962	2.56351E-38	3.6397E-15	0.006493844	2.78769E-40	-3.707983922	0.726629867	4.434613789
371	RGS10	O43665	regulator of G protein signaling 10		yes	no	no	no	no	yes	1.67108E-12	0.058524342	7.31709E-23	4.23258E-13	0.026225906	2.02571E-23	-2.581479539	0.587804302	3.169283841
372	ACTN4	O43707	actinin alpha 4		yes	no	no	no	no	yes	1.89155E-05	0.574107149	0.001121065	1.15876E-05	0.470315236	0.000865561	-1.492663503	-0.233057856	1.259605647
373	SYNGR2	O43760	synaptogyrin 2		yes	no	no	no	no	yes	9.534E-09	0.33655303	1.06428E-08	4.41493E-09	0.232704995	6.08091E-09	-1.676881948	-0.268361018	1.40852093
374	ENSA	O43768	endosulfine alpha		yes	no	no	no	no	yes	9.1219E-14	0.35803738	7.819E-28	1.60508E-14	0.251396648	1.33918E-28	-3.561329228	-0.310843195	3.250486033
375	NARS1	O43776	asparaginyl-tRNA synthetase 1	Asparagine	yes	no	no	no	no	yes	1.41091E-13	0.072446597	2.7249E-25	2.65305E-14	0.033712123	6.25974E-26	-2.55619702	0.478277654	3.034474674
376	KRT86	O43790	keratin 86		no	yes	no	no	yes	no	9.64716E-10	0.002100245	3.28989E-06	3.99525E-10	0.000413959	2.16742E-06	2.0846132	0.912780444	-1.171832756
377	LANCL1	O43813	LanC like glutathione S-transferase 1		yes	no	no	no	no	yes	2.60012E-14	0.724605948	2.42729E-28	3.86424E-15	0.635630773	3.87134E-29	-2.086026965	0.07503311	2.161060075
378	CALU	O43852	calumenin		yes	no	no	no	no	yes	1.27731E-14	0.09933264	5.12525E-22	1.68417E-15	0.050543976	1.48393E-22	-2.125244983	0.381512042	2.506757025
379	KALRN	O60229	kalirin RhoGEF kinase		yes	no	no	no	no	yes	4.04133E-15	0.031354804	4.99635E-10	4.3215E-16	0.012047519	2.64872E-10	-3.43714403	-0.923624877	2.513519153
380	GMFG	O60234	glia maturation factor gamma		yes	no	no	no	no	yes	1.7969E-14	0.961240433	1.74893E-26	2.52397E-15	0.941641058	3.5264E-27	-3.098710695	0.017376108	3.116086803
381	PRPSAP2	O60256	phosphoribosyl pyrophosphate synthetase associated protein 2		yes	no	no	no	no	yes	1.00044E-13	0.085321677	2.88172E-34	1.79056E-14	0.041396966	1.39712E-35	-2.772694261	0.4365093	3.209203561
382	KIAA0513	O60268	KIAA0513		yes	no	no	no	no	yes	1.08698E-17	0.006041407	8.23836E-33	2.46256E-19	0.001505561	6.27116E-34	-4.613719955	0.901844316	5.515564271
383	ACSL4	O60488	acyl-CoA synthetase long chain family member 4	Icosapent; Troglitazone; Rosiglitazone	yes	no	no	no	no	yes	9.68055E-09	0.258486306	7.37258E-34	4.48718E-09	0.167483197	4.24249E-35	-2.54803051	0.413207881	2.961238391
384	SNX3	O60493	sorting nexin 3	S,S-(2-Hydroxyethyl)Thiocysteine	yes	no	no	no	no	yes	1.00898E-12	0.95575484	2.82989E-28	2.45502E-13	0.934133277	4.56475E-29	-3.027704136	0.020821286	3.048525422
385	DOK2	O60496	docking protein 2		yes	no	no	no	no	yes	7.056E-18	0.020268743	3.29628E-34	1.2149E-19	0.006915434	1.67278E-35	-3.541370903	0.582503022	4.123873925
386	SYNCRIP	O60506	synaptotagmin binding cytoplasmic RNA interacting		yes	no	no	no	no	yes	2.26011E-13	0.066662827	6.17176E-24	4.49565E-14	0.03062805	1.58279E-24	-3.824075853	-0.670698627	3.153377226
387	EXOC3	O60645	exocyst complex component 3		yes	no	no	no	no	yes	5.81478E-09	0.017300974	3.63914E-31	2.63017E-09	0.005719343	3.82547E-32	-2.926651872	1.006204254	3.932856126
388	PLIN3	O60664	perilipin 3	Idursulfase; Galsulfase	yes	no	no	no	no	yes	4.5422E-12	0.051808126	1.01947E-20	1.27396E-12	0.022746749	3.27506E-21	-1.447448902	0.35182674	1.799275642
389	SNX2	O60749	sorting nexin 2		yes	no	no	no	no	yes	1.2053E-14	0.000529431	8.68857E-32	1.56192E-15	7.60442E-05	8.16453E-33	-2.645390819	0.865433039	3.510823858
390	CCDC22	O60826	coiled-coil domain containing 22		yes	no	no	no	no	yes	1.54616E-15	0.01743596	8.64948E-11	1.33108E-16	0.00580672	4.44036E-11	-1.939142625	-0.541194417	1.397948208
391	EIF5B	O60841	eukaryotic translation initiation factor 5B		yes	no	no	no	no	yes	7.0768E-17	0.823582341	2.71251E-25	2.98207E-18	0.75753156	6.1944E-26	-3.708696945	0.067028266	3.775725211
392	DNAJA2	O60884	DnaJ heat shock protein family (Hsp40) member A2		yes	no	no	no	no	yes	6.9674E-13	0.598954572	1.73459E-29	1.62267E-13	0.496871192	2.35785E-30	-2.624738762	0.146402413	2.771141176
393	CUTA	O60888	cutA divalent cation tolerance homolog		yes	no	no	no	no	yes	1.38675E-16	0.012429231	3.03505E-21	6.34625E-18	0.003750733	9.37882E-22	-2.533081752	0.450879024	2.983960776
394	CLASP2	O75122	cytoplasmic linker associated protein 2		yes	no	no	no	no	yes	3.99509E-13	0.180317721	4.6946E-10	8.47169E-14	0.106458537	2.48663E-10	-2.394480345	-0.456894205	1.93758614
395	CPNE3	O75131	copine 3		yes	no	no	no	no	yes	5.5842E-15	0.211709105	1.12047E-21	6.35086E-16	0.130363695	3.35075E-22	-2.022244659	0.269592329	2.291836988
396	DNAJC13	O75165	DnaJ heat shock protein family (Hsp40) member C13		yes	no	no	no	no	yes	0.004407139	0.900212104	0.000110842	0.00338473	0.858199487	8.08085E-05	-1.067964725	0.067207864	1.135172589
397	TBCA	O75347	tubulin folding cofactor A		yes	no	no	no	no	yes	9.58808E-13	0.719239539	1.32685E-22	2.31122E-13	0.629415478	3.73345E-23	-1.951055084	0.086994941	2.038050025
398	ATP6V1G1	O75348	ATPase H+ transporting V1 subunit G1		yes	no	no	no	no	yes	2.08379E-17	0.242044317	2.30144E-23	6.60921E-19	0.153649337	6.21104E-24	-3.22819732	-0.308578719	2.919618602
399	VPS4B	O75351	vacuolar protein sorting 4 homolog B		yes	no	no	no	no	yes	6.61835E-13	0.665202656	3.63886E-21	1.52939E-13	0.567848575	1.12942E-21	-2.609673673	-0.127311632	2.482362041
400	SH3BGRL	O75368	SH3 domain binding glutamate rich protein like		yes	no	no	no	no	yes	3.15001E-17	0.204938414	1.49144E-31	1.11328E-18	0.124523069	1.42589E-32	-3.099518525	0.284575027	3.384093552
401	FLNB	O75369	filamin B		no	no	no	yes	no	yes	0.158416288	0.00341197	7.23684E-07	0.140399755	0.000747111	4.59065E-07	-0.350335106	1.123383533	1.473718638
402	CS	O75390	citrate synthase	Trifluoroacetonyl coenzyme A; Coenzyme A; Oxaloacetate Ion; alpha-Fluoro-carboxymethyldethia coenzyme A complex; D-Malic acid; Nitromethyldethia coenzyme A; Citric acid	yes	no	no	no	no	yes	9.91053E-08	0.018598333	2.62927E-11	5.04732E-08	0.006252815	1.31523E-11	-2.755612198	1.031979524	3.787591722
403	BANF1	O75531	barrier to autointegration nuclear assembly factor 1		yes	no	no	no	no	yes	3.72593E-11	0.094424294	6.10681E-16	1.26786E-11	0.047276323	2.47095E-16	-2.256448456	0.514238876	2.770687332
404	SKAP2	O75563	src kinase associated phosphoprotein 2		yes	no	no	no	no	yes	5.7514E-13	0.282356697	1.3651E-32	1.30662E-13	0.187043721	1.10098E-33	-4.353479727	0.459027871	4.812507598
405	FCN3	O75636	ficolin 3	Zinc; Zinc acetate	no	no	no	yes	no	yes	0.015054002	0.00180307	3.41678E-08	0.012134603	0.000339046	2.0188E-08	-1.263064046	1.419913419	2.682977465
406	EIF3G	O75821	eukaryotic translation initiation factor 3 subunit G		yes	no	no	no	no	yes	1.24211E-16	0.09291157	7.46598E-27	5.51547E-18	0.046182144	1.43772E-27	-2.369226958	0.316654515	2.685881473
407	EIF3J	O75822	eukaryotic translation initiation factor 3 subunit J		yes	no	no	no	no	yes	7.37587E-10	0.768824031	2.82989E-28	3.01786E-10	0.689050264	4.55616E-29	-2.645385812	0.111541272	2.756927084

408	IDH1	O75874	isocitrate dehydrogenase (NADP(+)) 1	Isocitric Acid; Nicotinamide adenine dinucleotide phosphate; Ivosidenib	yes	no	no	no	no	yes	4.46545E-05	0.424937277	3.22427E-14	2.82454E-05	0.316729416	1.40541E-14	-1.058939202	0.215880976	1.274820178
409	TNFSF13	O75888	TNF superfamily member 13		yes	no	no	no	no	yes	1.74848E-06	0.384171473	1.07885E-10	9.86344E-07	0.276596497	5.57266E-11	-1.422795014	0.302599425	1.725394439
410	DNAJC8	O75937	DnaJ heat shock protein family (Hsp40) member C8		yes	no	no	no	no	yes	1.80502E-13	0.026644339	8.41462E-17	3.50862E-14	0.009799875	3.27511E-17	-2.876253928	-0.663975991	2.212277937
411	FLOT1	O75955	flotillin 1		yes	no	yes	no	no	no	5.89515E-08	4.05949E-10	0.002181743	2.93823E-08	1.65543E-12	0.001710202	-1.783582506	-2.479422908	-0.695840402
412	ECM2	O94769	extracellular matrix protein 2		no	yes	no	no	yes	no	1.00426E-12	0.009985414	1.23917E-10	2.43897E-13	0.002841341	6.4064E-11	2.28437411	0.512591897	-1.771782213
413	STK10	O94804	serine/threonine kinase 10	Fostamatinib	yes	no	no	no	no	yes	1.54601E-11	0.11194192	2.85066E-25	4.93153E-12	0.058735271	6.57448E-26	-2.875810329	-0.500266138	2.375544191
414	UFL1	O94874	UFM1 specific ligase 1		yes	no	no	no	no	yes	7.43296E-12	0.04798325	1.97697E-28	2.19587E-12	0.020763028	3.11729E-29	-3.5983243	0.81118121	4.40950551
415	CDK14	O94921	cyclin dependent kinase 14		yes	no	yes	no	no	no	0.000268598	0.007041721	0.267635102	0.000182676	0.001799516	0.247626134	-1.302823384	-1.019910654	0.28291273
416	ATE1	O95260	arginyltransferase 1		yes	no	no	no	no	yes	2.80669E-14	0.266583792	1.53154E-29	4.19669E-15	0.174324095	2.06101E-30	-3.696279343	0.382477119	4.078756462
417	IPO7	O95373	importin 7		yes	no	no	no	no	yes	4.84306E-12	0.008177055	7.38917E-33	1.37198E-12	0.002223032	5.55778E-34	-2.517896462	0.718014379	3.235910841
418	TNFAIP8	O95379	TNF alpha induced protein 8		yes	no	no	no	no	yes	1.99728E-12	0.1224058	5.31185E-16	5.17645E-13	0.065830493	2.13725E-16	-2.656783225	-0.487490903	2.169292322
419	PGM3	O95394	phosphoglucomutase 3		no	yes	no	yes	no	no	0.000188159	0.000138571	0.131954299	0.000126349	1.33108E-05	0.119159455	1.948942975	2.324883438	0.375940462
420	AHSA1	O95433	activator of HSP90 ATPase activity 1		yes	no	no	no	no	yes	1.99935E-10	0.000859211	1.64288E-27	7.65496E-11	0.00014038	2.89569E-28	-1.838228804	0.850694924	2.688923727
421	ACSL3	O95573	acyl-CoA synthetase long chain family member 3		yes	no	no	no	no	yes	1.06114E-14	0.005420131	6.02522E-22	1.33183E-15	0.0013235	1.76634E-22	-2.339817385	0.756479728	3.096297113
422	SNAP29	O95721	synaptosome associated protein 29		yes	no	no	no	no	yes	4.18123E-16	0.000169961	5.5901E-21	2.57656E-17	1.72502E-05	1.75276E-21	-2.589885163	0.678092825	3.267977987
423	OXSR1	O95747	oxidative stress responsive kinase 1	Fostamatinib	yes	no	no	no	no	yes	0.00016441	0.47739743	4.35264E-13	0.000109433	0.36751166	2.00573E-13	-1.334841643	0.269413282	1.604254926
424	AP2A1	O95782	adaptor related protein complex 2 subunit alpha 1		yes	no	no	no	no	yes	4.04629E-11	0.058456307	1.20568E-36	1.39338E-11	0.026168931	3.35869E-38	-2.871021539	0.600625839	3.471647378
425	STAU1	O95793	staufen double-stranded RNA binding protein 1		no	no	no	yes	no	yes	0.212702588	0.006284958	8.11801E-13	0.191596169	0.001577647	3.78865E-13	-0.396570082	1.013320766	1.409890849
426	CAVIN2	O95810	caveolae associated protein 2		yes	no	no	no	no	yes	6.71825E-11	0.099877656	6.8614E-35	2.38655E-11	0.050911809	3.04675E-36	-4.655954718	0.874643549	5.530598267
427	MAP4K4	O95819	mitogen-activated protein kinase kinase kinase kinase 4	Fostamatinib	yes	no	no	no	no	yes	1.63617E-14	0.172720995	3.48929E-34	2.23889E-15	0.101112608	1.80235E-35	-4.107375854	0.466981796	4.57435765
428	MPIG6B	O95866	megakaryocyte and platelet inhibitory receptor G6b		yes	no	no	no	no	yes	3.48002E-09	0.077483605	5.13375E-24	1.53739E-09	0.036547545	1.31193E-24	-3.343620591	0.853332521	4.196953113
429	TXNDC12	O95881	thioredoxin domain containing 12		yes	no	no	no	no	yes	0.0011471	0.69059913	1.25345E-06	0.000834727	0.595160646	8.0648E-07	-1.264609555	-0.18826189	1.076347664
430	ADH1B	P00325	alcohol dehydrogenase 1B (class I), beta polypeptide	NADH; Fomepizole; N-Benzylformamide; 4-Iodopyrazole; Nicotinamide adenine dinucleotide phosphate; Cyclohexanol; N-Heptylformamide; Glycerin	no	yes	no	no	yes	no	6.08432E-07	0.536955533	2.44361E-08	3.31646E-07	0.431851414	1.43495E-08	1.929748225	0.243499677	-1.686248548
431	ADH1C	P00326	alcohol dehydrogenase 1C (class I), gamma polypeptide	NADH; Fomepizole; 2,3,4,5,6-Pentafluorobenzyl Alcohol; N-1-methylheptylformamide; 2-Ethoxyethanol; Cholic Acid; 4-Iodopyrazole; Pyrazole; Para-Bromobenzyl Alcohol; 3-Butylthiolane 1-Oxide; 5-beta-D-ribofuranosylnicotinamide adenine dinucleotide; (R)-N-(1-Methyl-Hexyl)-Formamide; Trifluoroethanol; Cyclohexylformamide; Cpad; N-Formylpiperidine; 2,3-Difluorobenzyl Alcohol; 2,4-Difluorobenzyl Alcohol 2,4-	no	yes	no	no	yes	no	2.26008E-05	0.38426651	3.97464E-05	1.3968E-05	0.276839036	2.81485E-05	1.392337009	0.322387558	-1.069949451
432	LDHA	P00338	lactate dehydrogenase A	NADH; Etheno-NAD; Nicotinamide; Oxamic Acid; Stiripentol; Copper; Artenimol	yes	no	no	no	no	yes	6.50103E-12	0.006830476	1.27942E-20	1.88521E-12	0.001742437	4.13915E-21	-1.663674892	0.606396099	2.270070992
433	SOD1	P00441	superoxide dismutase 1	Dopamine; Isoprenaline; Zinc; 5-fluorouridine; S-oxy-L-cysteine; Arimoclomol; Cannabidiol; Medical Cannabis; Nabiximols; Zinc acetate; Zinc	yes	no	no	no	no	yes	2.27269E-14	0.227047217	2.70719E-26	3.28496E-15	0.141233734	5.58121E-27	-2.666671216	0.307043504	2.97371472
434	HPRT1	P00492	hypoxanthine phosphoribosyltransferase 1	Mercaptopurine; 5-O-phosphono-alpha-D-ribofuranosyl diphosphate; 5-monophosphate-9-beta-D-ribofuranosyl xanthine; 3H-pyrazolo[4,3-d]pyrimidin-7-ol;	yes	no	no	no	no	yes	1.61817E-14	0.000136693	2.13784E-33	2.19959E-15	1.30685E-05	1.43235E-34	-2.563232205	0.982506059	3.545738264
435	PGK1	P00558	phosphoglycerate kinase 1	Adenosine-5'-[Beta, Gamma-Methylene]Triphosphate; 3-phospho-D-glyceric acid; Copper; Artenimol	yes	no	no	no	no	yes	1.08991E-13	0.000675018	5.67304E-30	1.99019E-14	0.000104908	7.14592E-31	-2.27690503	0.816663206	3.093568236
436	F9	P00740	coagulation factor IX	Antihemophilic factor, human recombinant; Menadione; TTP889; Turoctocog alfa; Kappadione; Coagulation factor VII human; Antihemophilic factor human; Emicizumab; Lonoctocog alfa; Moroctocog alfa; Turoctocog alfa pegol	no	yes	no	no	yes	no	3.91763E-13	0.068924363	4.26855E-13	8.28968E-14	0.031854486	1.96504E-13	2.11399778	0.342965845	-1.771031935
437	F10	P00742	coagulation factor X	Antihemophilic factor, human recombinant; Coagulation factor VIIa Recombinant Human; Coagulation Factor IX (Recombinant); Fondaparinux; Heparin; Enoxaparin; gamma-carboxy-L-glutamic acid; 4-[(5-CHLOROINDOL-2-YL)SULFONYL]-2-(2-METHYLPROPYL)-1-[[5-(PYRIDIN-4-YL)PYRIMIDIN-2-YL]CARBONYL]PIPERAZINE; SSR-126517E; LY-517717; Rivaroxaban; Lanoteplase; Idraparinux; rNAPc2; Apixaban; Otamixaban; Eribaxaban; (2R)-2-(5-CHLORO-2-THIENYL)-N-{(3S)-1-[(1S)-1-METHYL-2-MORPHOLIN-4-YL-2-OXOETHYL]-2-OXOPYRROLIDIN-3-YL}PROPENE-1-SULFONAMIDE; THIENO[3,2-B]PYRIDINE-2-SULFONIC ACID [1-(1-AMINO-ISOQUINOLIN-7-YLMETHYL)-2-OXO-PYRROLDIN-3-YL]-AMIDE; 2-(5-CHLORO-2-THIENYL)-N-{(3S)-1-[(1S)-1-METHYL-2-MORPHOLIN-4-YL-2-OXOETHYL]-2-OXOPYRROLIDIN-3-YL}ETHANESULFONAMIDE; GW-813893; 2-({4-[(5-CHLORO-1H-INDOL-2-YL)SULFONYL]PIPERAZIN-1-YL}CARBONYL)THIENO[3,2-	no	yes	no	no	yes	no	4.08866E-10	0.787155203	7.54667E-17	1.63213E-10	0.711542651	2.92703E-17	2.151907676	-0.079802359	-2.231710035
438	F12	P00748	coagulation factor XII	Zinc; Human C1-esterase inhibitor; Ethanolamine oleate; Conestat alfa; Nafamostat; Zinc acetate	no	yes	no	no	yes	no	3.84306E-05	0.64239485	5.80665E-06	2.41519E-05	0.543430533	3.87811E-06	1.145982995	0.132167053	-1.013815942
439	PLAT	P00750	plasminogen activator, tissue type	Aminocaproic acid; Iloprost; Human C1-esterase inhibitor; 5-(DIMETHYLAMINO)-2-NAPHTHALENESULFONIC ACID; Dexibuprofen; Conestat alfa	no	yes	no	no	yes	no	0.000674053	0.02655027	4.63537E-09	0.000478281	0.009744322	2.58337E-09	1.19474279	-0.686737895	-1.881480685

440	CA2	P00918	carbonic anhydrase 2	Methyclothiazide; Topiramate; Ethoxzolamide; Sulpiride; Bendroflumethiazide; Celecoxib; Benzthiazide; Valdecoxib; Cyclothiazide; Furosemide; Methazolamide; Hydroflumethiazide; Acetazolamide; Dorzolamide; Chlorothiazide; Zonisamide; Trichlormethiazide; Ethinamate; Diazoxide; Diclofenamide; Brinzolamide; Quinethazone; p-Hydroxymercuribenzoic acid; N-Benzyl-4-Sulfamoyl-Benzamide; 4-Flourobenzenesulfonamide; Formic acid; AL5424; N-(2-Flouro-Benzyl)-4-Sulfamoyl-Benzamide; 3,5-Difluorobenzenesulfonamide; AL7089A; 4-(Aminosulfonyl)-N-[(2,4,6-Trifluorophenyl)Methyl]-Benzamide; Irosustat; 4-(Aminosulfonyl)-N-[(4-Fluorophenyl)Methyl]-Benzamide; (R)-N-(3-Indol-1-Yl-2-Methyl-Propyl)-4-Sulfamoyl-Benzamide; Aminodi(Ethyloxy)Ethylaminocarbonylbenzenesulfonamide; AL7182; N-(2,3,4,5,6-Pentaflouro-Benzyl)-4-Sulfamoyl-Benzamide; Cyanamide; 4-(Aminosulfonyl)-N-[(3,4,5-Trifluorophenyl)Methyl]-Benzamide; Dansylamide; Sulfamic Acid	yes	no	no	no	no	yes	3.40148E-10	0.610330557	5.72588E-17	1.33778E-10	0.508009621	2.21044E-17	-1.680108548	-0.12813429	1.551974259
441	SERPINC1	P01008	serpin family C member 1	Ardeparin; Fondaparinux; Heparin; Enoxaparin; N-Formylmethionine; SR-123781A; Sulodexide; Danaparoid; Dalteparin; Tinzaparin; Nadroparin; Copper; Protamine sulfate; Bemiparin; Antithrombin	no	yes	no	no	yes	no	1.83019E-10	0.882259189	3.67304E-19	6.94926E-11	0.829491535	1.27816E-19	1.17821636	-0.025872439	-1.204088799
442	TIMP1	P01033	TIMP metallopeptidase inhibitor 1		no	no	no	yes	no	yes	0.149932111	6.53248E-06	5.32769E-07	0.132472866	2.09895E-07	3.3627E-07	-0.419875299	1.192681995	1.612557294
443	KNG1	P01042	kininogen 1	Zinc; Copper; Zinc acetate; Zinc chloride; Zinc sulfate, unspecified form	no	yes	no	no	yes	no	1.08263E-13	0.014618859	4.70595E-16	1.97199E-14	0.004630078	1.89133E-16	1.457179808	-0.241384317	-1.698564125
444	NRAS	P01111	NRAS proto-oncogene, GTPase		yes	no	no	no	no	yes	5.52409E-09	0.64239485	2.84889E-26	2.48797E-09	0.543243982	5.88625E-27	-2.841379529	0.200221407	3.041600937
445	KRAS	P01116	KRAS proto-oncogene, GTPase	[(3,7,11-TRIMETHYL-DODECA-2,6,10-TRIENYLOXYCARBAMOYL)-METHYL]-PHOSPHONIC ACID; Farnesyl diphosphate; MRTX849; AMG-510	yes	no	no	no	no	yes	6.71407E-12	0.513544174	6.78776E-26	1.95612E-12	0.406507327	1.46704E-26	-2.88185668	0.223243929	3.105100609
446	TGFB1	P01137	transforming growth factor beta 1	Hyaluronidase (ovine); Terazosin; Hyaluronidase (human recombinant); Foreskin fibroblast (neonatal); Foreskin keratinocyte (neonatal); Hyaluronidase	yes	no	no	no	no	yes	1.16271E-09	0.564235179	1.00701E-18	4.87317E-10	0.460182747	3.58634E-19	-2.896826648	0.261237576	3.158064224
447	GCG	P01275	glucagon		no	yes	no	no	yes	no	1.55887E-06	0.093398603	4.90014E-14	8.77966E-07	0.046593503	2.15366E-14	1.407252908	-0.478843564	-1.886096473
448	IGF2	P01344	insulin like growth factor 2		no	yes	no	no	yes	no	3.39915E-12	0.045543939	6.76352E-14	9.241E-13	0.019392408	2.99715E-14	2.164931741	0.458278756	-1.706652985
449	IGKV1D-33	P01593	None	None	no	yes	no	no	yes	no	0.000165899	0.465903288	3.54461E-05	0.000110574	0.356341074	2.49906E-05	1.101351708	-0.332515405	-1.433867113
450	IGKV1D-16	P01601	None	None	no	yes	no	no	yes	no	0.005069122	0.874548021	6.28685E-05	0.00392989	0.820656525	4.51502E-05	1.205468586	-0.091360253	-1.296828839
451	IGKV3-20	P01619	None	None	no	yes	no	no	yes	no	4.26477E-06	0.02136424	8.6013E-11	2.47731E-06	0.007444087	4.41172E-11	1.177197958	-0.550954167	-1.728152126
452	IGLV1-47	P01700	None	None	no	yes	no	no	yes	no	8.76562E-05	0.890137781	1.2258E-08	5.7193E-05	0.841738808	7.03712E-09	1.068496749	-0.048949346	-1.117446095
453	IGLV1-51	P01701	None	None	no	yes	no	no	yes	no	4.03414E-05	0.65326731	2.07387E-07	2.54076E-05	0.55440402	1.27797E-07	1.411452696	0.179096434	-1.232356262
454	IGLV2-11	P01706	None	None	no	yes	no	no	yes	no	0.001113076	0.612826263	4.07092E-06	0.000807951	0.511636097	2.70411E-06	1.259463807	0.225003947	-1.03445986
455	IGLV2-8	P01709	None	None	no	yes	no	no	yes	no	0.003825036	0.969242054	0.001116258	0.00292727	0.952992867	0.000861344	1.028949027	-0.019849394	-1.048798421
456	IGLV3-1	P01715	None	None	no	yes	no	no	yes	no	5.88978E-06	0.551033076	3.34194E-06	3.45328E-06	0.44716821	2.20323E-06	1.790007924	0.283873483	-1.506134441
457	IGHV3-23	P01764	None	None	no	yes	no	no	yes	no	2.01787E-06	0.930752225	1.868E-09	1.14471E-06	0.899439807	1.0233E-09	1.952204761	-0.04390556	-1.996110321
458	IGHV2-5	P01817	None	None	no	yes	no	no	yes	no	0.001325593	0.245454751	1.54225E-06	0.000973623	0.156704007	1.00068E-06	1.127936418	-0.438549491	-1.566485909
459	PIGR	P01833	polymeric immunoglobulin receptor		no	yes	no	no	yes	no	5.64611E-09	0.007822333	1.74777E-06	2.54804E-09	0.002077053	1.1372E-06	2.024256431	0.854466667	-1.169789764
460	HLA-B	P01889	major histocompatibility complex, class I, B	Abacavir; N-Formylmethionine; Coccidioides immitis spherule	yes	no	no	no	no	yes	5.90621E-11	0.091379405	2.12426E-19	2.074E-11	0.045172148	7.35358E-20	-2.388619284	0.505744348	2.894363632
461	HBD	P02042	hemoglobin subunit delta		yes	no	no	no	no	yes	0.004546368	0.890137781	1.14296E-05	0.00349578	0.84154369	7.80959E-06	-1.163233687	0.067097897	1.230331584
462	COL1A1	P02452	collagen type I alpha 1 chain	Collagenase clostridium histolyticum; Halofuginone; Clove oil; Vonicog Alfa; Von Willebrand Factor Human	no	yes	no	yes	no	no	0.001736259	0.003626153	0.656977009	0.001291771	0.000804476	0.637925568	2.179640365	2.271605813	0.091965448
463	KRT14	P02533	keratin 14	Zinc; Zinc acetate	no	yes	no	no	yes	no	0.000408534	0.415889376	0.000116247	0.000283771	0.308854865	8.48017E-05	1.427365693	0.373982058	-1.053383636
464	KRT6A	P02538	keratin 6A	Zinc; Zinc acetate	no	yes	no	no	yes	no	0.000292579	0.402716984	6.38168E-05	0.000199914	0.296594088	4.5918E-05	1.423418351	0.368545829	-1.054872522
465	APOA1	P02647	apolipoprotein A1	Zinc; Copper; Zinc acetate; Zinc chloride; Zinc sulfate, unspecified form	no	yes	no	no	yes	no	2.1829E-12	0.015814088	1.20964E-14	5.71688E-13	0.00510584	5.16302E-15	1.137714987	-0.195387623	-1.33310261
466	APOE	P02649	apolipoprotein E	Zinc; Copper; Zinc acetate; Zinc chloride; Zinc sulfate, unspecified form	no	yes	no	no	yes	no	1.88845E-10	0.020939221	3.96551E-15	7.20053E-11	0.007191631	1.67281E-15	1.261590969	-0.325249681	-1.58684065
467	APOC1	P02654	apolipoprotein C1		no	yes	no	no	yes	no	6.13799E-08	0.003155236	1.53151E-12	3.06761E-08	0.000679083	7.26546E-13	1.149198263	-0.46081106	-1.610009323
468	APOC2	P02655	apolipoprotein C2	Copper	no	yes	no	no	yes	no	9.94371E-12	0.592627953	2.52309E-14	3.03673E-12	0.489784045	1.09063E-14	1.170200032	0.082166759	-1.088033272
469	MBP	P02686	myelin basic protein		yes	no	no	no	no	yes	4.26723E-23	0.427874313	1.33692E-26	1.9335E-26	0.319500167	2.65929E-27	-3.263566891	-0.169496238	3.094070653
470	CRP	P02741	C-reactive protein	N,N,N-Trimethyl-2-(phosphonooxy)ethanaminium; CRx-139	no	yes	no	no	yes	no	2.17843E-07	0.452242793	4.86374E-09	1.14202E-07	0.344048776	2.71506E-09	3.392406354	0.450379248	-2.942027106
471	APCS	P02743	amyloid P component, serum	Zinc; Methyl 4,6-O-[(1R)-1-carboxyethylidene]-beta-D-galactopyranoside; Bis-1,2-{[(Z)-2-carboxy-2-methyl-1,3-dioxane]-5-yloxycarbamoyl}-ethane; BIS-1,2-{[(Z)-2CARBOXY-2-METHYL-1,3-DIOXANE]-5-YLOXYCARBONYL}-PIPERAZINE;	no	yes	no	yes	no	no	6.97763E-05	0.007822333	4.22015E-05	4.49578E-05	0.002079893	2.99445E-05	2.353098853	1.634643796	-0.718455056
472	C1QA	P02745	complement C1q A chain	Cetuximab; Etanercept; Palivizumab; Daclizumab; Bevacizumab	no	yes	no	no	yes	no	7.3623E-05	0.283097032	5.26691E-11	4.75364E-05	0.187790691	2.67522E-11	1.123110013	-0.285673315	-1.408783328
473	C1QB	P02746	complement C1q B chain	Cetuximab; Palivizumab; Daclizumab; Bevacizumab; Zinc; Zinc acetate; Zinc chloride; Zinc sulfate, unspecified form	no	yes	no	no	yes	no	2.77854E-06	0.295179733	7.29357E-12	1.58504E-06	0.19794563	3.55921E-12	1.45886147	-0.286841299	-1.745702769
474	C1QC	P02747	complement C1q C chain	Cetuximab; Palivizumab; Daclizumab; Bevacizumab; Zinc; Copper; Zinc acetate; Zinc chloride; Zinc sulfate, unspecified form	no	yes	no	no	yes	no	6.93568E-07	0.161406158	2.82903E-11	3.80253E-07	0.092517911	1.41644E-11	1.512236372	-0.340762031	-1.852998404
475	FN1	P02751	fibronectin 1	Zinc; Lanoteplase; Ocriplasmin; Zinc acetate; Zinc chloride; Zinc sulfate,	no	no	no	yes	no	yes	0.0820723	1.0703E-07	2.43336E-05	0.07076737	1.01291E-09	1.69905E-05	0.451634393	1.463403884	1.011769491
476	RBP4	P02753	retinol binding protein 4	Tretinoin	no	yes	no	no	yes	no	7.55368E-09	0.003123038	2.0853E-16	3.47052E-09	0.000670739	8.22972E-17	1.305712591	-0.541595626	-1.847308218
477	PPBP	P02775	pro-platelet basic protein	Ethanesulfonic acid; Copper	yes	no	no	no	no	yes	2.23132E-05	0.216423801	1.87715E-11	1.37802E-05	0.133462978	9.33046E-12	-2.250946107	-0.664581762	1.586364345
478	PF4	P02776	platelet factor 4	Drotrecogin alfa; Heparin	yes	no	no	no	no	yes	1.58413E-11	0.069671828	2.34452E-18	5.06032E-12	0.032263076	8.48785E-19	-2.534262508	0.653916142	3.18817865
479	CXCL10	P02778	C-X-C motif chemokine ligand 10	N-Methylleucine; Eldelumab; Clove oil	no	yes	no	yes	no	no	0.000328738	0.000648399	0.792971508	0.00022611	9.84204E-05	0.778958871	1.052058391	1.094517427	0.042459036

480	TFRC	P02786	transferrin receptor	Iron; Ferrous sulfate anhydrous; Ferric cation; Ferrous gluconate; Ferrous succinate; Ferrous ascorbate; Ferrous fumarate; Ferrous glycine sulfate; Tetraferric tricitrate decahydrate; Ferric derisomaltose	no	no	yes	no	yes	no	0.963651863	0.000313611	9.4867E-08	0.961468691	3.7514E-05	5.74704E-08	-0.013377803	-1.226883234	-1.213505431
481	F11	P03951	coagulation factor XI	Coagulation Factor IX (Recombinant); Human C1-esterase inhibitor; 3-Hydroxypropyl 3-[(7-carbamimidoyl-1-naphthyl)carbamoyl]benzenesulfonate; (1R)-2-[(Diaminomethylene)amino]-1-{4-[(4R)-4-(hydroxymethyl)-1,3,2-dioxaborolan-2-yl]phenyl}ethyl nicotinate; (R)-1-(4-(4-(Hydroxymethyl)-1,3,2-dioxaborolan-2-YL)phenethyl)guanidine; 6-CARBAMIMIDOYL-4-(3-HYDROXY-2-METHYL-BENZOYLAMINO)-NAPHTHALENE-2-CARBOXYLIC ACID METHYL ESTER; (R)-1-(4-(4-(Hydroxymethyl)-1,3,2-dioxaborolan-2-YL)phenyl)guanidine; N-(7-CARBAMIMIDOYL-NAPHTHALEN-1-YL)-3-HYDROXY-2-METHYL-BENZAMIDE; 4-METHYL-PENTANOIC ACID {1-[4-GUANIDINO-1-(THIAZOLE-2-CARBONYL)-BUTYLCARBAMOYL]-2-METHYL-PROPYL}-AMIDE; (R)-1-(4-(4-(hydroxymethyl)-1,3,2-dioxaborolan-2-yl)benzyl)guanidine; Conestat alfa; Thrombin; Human thrombin; Coagulation	no	yes	no	no	yes	no	1.24839E-11	3.63077E-05	8.17064E-16	3.886E-12	2.22091E-06	3.33193E-16	2.16066801	-0.758539576	-2.919207585
482	KLKB1	P03952	kallikrein B1	Zinc; Ecallantide; Human C1-esterase inhibitor; Conestat alfa; Gabexate; Zinc acetate; Zinc chloride; Zinc sulfate, unspecified form; Lanadelumab	no	yes	no	no	yes	no	1.61721E-08	0.000871236	5.59105E-14	7.6427E-09	0.000145602	2.46746E-14	1.552845668	-0.710064156	-2.262909824
483	C4BPA	P04003	complement component 4 binding protein alpha	Zinc; Copper; Zinc acetate; Zinc chloride; Zinc sulfate, unspecified form	no	yes	no	no	yes	no	9.10147E-12	0.052508609	6.76713E-11	2.75065E-12	0.02317326	3.45256E-11	1.388216689	0.284073829	-1.10414286
484	PROC	P04070	protein C, inactivator of coagulation factors Va and VIIIa	Menadione; Sodium tetradecyl sulfate; Cupric Chloride; Kappadione; Protein S	no	yes	no	no	yes	no	5.7514E-13	0.300444223	1.2114E-13	1.3108E-13	0.201991459	5.445E-14	2.458913631	0.229675	-2.229238631
485	CSTB	P04080	cystatin B	Cupric Chloride	yes	no	no	no	no	yes	9.54704E-08	0.176554283	3.98389E-11	4.8449E-08	0.103840116	2.0091E-11	-1.197640424	0.287847838	1.485488262
486	HRG	P04196	histidine rich glycoprotein	Zinc chloride; Zinc sulfate, unspecified form	no	yes	no	no	yes	no	1.04982E-07	0.366959979	5.19411E-17	5.3704E-08	0.25988149	1.99574E-17	1.41645235	-0.216240702	-1.632693052
487	KRT1	P04264	keratin 1	Zinc; Copper; Zinc acetate	no	yes	no	no	yes	no	1.23289E-05	0.130967319	7.55403E-05	7.41859E-06	0.071625534	5.45588E-05	1.850367897	0.651188389	-1.199179508
488	VWF	P04275	von Willebrand factor	Antihemophilic factor, human recombinant; Egaptivon pegol; Caplacizumab; Simoctocog alfa; Antihemophilic Factor (Recombinant), PEGylated; Susoctocog alfa; Efmoroctocog alfa; Lonoctocog alfa; Moroctocog alfa	no	no	no	yes	no	yes	0.578742505	7.88269E-12	1.8444E-17	0.553306155	1.42867E-14	6.9029E-18	-0.200986479	3.554144165	3.755130645
489	SEMG1	P04279	semenogelin 1	Zinc; Zinc acetate; Zinc chloride; Zinc sulfate, unspecified form	no	yes	no	no	yes	no	7.15679E-06	0.982271759	4.30209E-08	4.22857E-06	0.97482282	2.55942E-08	2.22347004	0.013365007	-2.210105033
490	GAPDH	P04406	glyceraldehyde-3-phosphate dehydrogenase	NADH; Adenosine-5-Diphosphoribose; Thionicotinamide-Adenine-Dinucleotide; 4-(2-Aminoethyl)Benzenesulfonyl Fluoride; Xanthinol; Copper; Artenimol	yes	no	no	no	no	yes	1.75337E-14	0.719239539	6.53366E-24	2.45487E-15	0.62961975	1.68152E-24	-2.664503999	-0.099182977	2.565321022
491	ASL	P04424	argininosuccinate lyase	Arginine; Argininosuccinate; 2-(N-morpholino)ethanesulfonic acid	yes	no	no	no	no	yes	4.37587E-05	0.195208848	8.74108E-11	2.76391E-05	0.117107619	4.49134E-11	-1.224912755	0.438227546	1.6631403
492	HLA-A	P04439	major histocompatibility complex, class I, A	3-Indolebutyric Acid; Nelipepimut-S; Coccidioides immitis spherule	yes	no	no	no	no	yes	6.45338E-11	0.863685292	1.78244E-21	2.28661E-11	0.808799737	5.40304E-22	-3.278204611	0.081175842	3.359380452
493	CAPNS1	P04632	calpain small subunit 1	PD150606	yes	no	no	no	no	yes	1.84613E-17	0.001940372	2.96183E-31	5.07263E-19	0.000372776	2.9927E-32	-2.674001319	0.666446809	3.340448127
494	GNAI2	P04899	G protein subunit alpha i2		yes	no	no	no	no	yes	1.32858E-09	0.771690989	5.45395E-26	5.60449E-10	0.693018369	1.15899E-26	-3.137819946	0.135896092	3.273716038
495	IGF1	P05019	insulin like growth factor 1	N,N-Bis(3-(D-gluconamido)propyl)deoxycholamide; N-Dodecyl-N,N-Dimethyl-3-Ammonio-1-Propanesulfonate	no	yes	no	no	yes	no	3.16213E-10	0.078717178	1.0026E-09	1.23648E-10	0.037432942	5.39684E-10	2.00634293	0.450376411	-1.555966518
496	ATP1A1	P05023	ATPase Na+/K+ transporting subunit alpha 1	Digoxin; Acetyldigitoxin; Hydroflumethiazide; Etacrynic acid; Trichlormethiazide; Deslanoside; Ouabain; Diazoxide; Bretylium; Ciclopirox; Bepridil; Potassium cation; Aluminium; Magnesium cation; Digitoxin; Almitrine; Istaroxime; Magnesium acetate; Potassium acetate; Potassium sulfate; Potassium; Magnesium levulinate; Magnesium lactate; Aluminium phosphate; Aluminum acetate	yes	no	no	no	no	yes	2.69305E-09	0.804487861	1.09998E-12	1.17386E-09	0.733759526	5.19339E-13	-1.792058218	0.069076073	1.861134291
497	ITGB3	P05106	integrin subunit beta 3	Abciximab; Eptifibatide; Antithymocyte immunoglobulin (rabbit); Levothyroxine; Tirofiban; Resveratrol; Lefradafiban; LM-609; Fradafiban; Ferric maltol	yes	no	no	no	no	yes	2.00729E-08	0.146665293	1.60242E-32	9.56804E-09	0.082270789	1.33596E-33	-4.259925285	0.923005114	5.182930399
498	S100A8	P05109	S100 calcium binding protein A8	Calcium; Zinc; Copper; Zinc acetate; Zinc chloride; Zinc sulfate, unspecified form	no	no	yes	no	yes	no	0.354319446	0.001323934	1.13006E-08	0.327669229	0.000235753	6.47212E-09	0.367840686	-1.451451547	-1.819292232
499	SERPINA5	P05154	serpin family A member 5	Urokinase; Drotrecogin alfa; Tifuvirtide; PPL-100	no	yes	no	no	yes	no	7.02807E-09	0.259673853	7.10142E-14	3.21629E-09	0.168605632	3.15333E-14	1.452644416	-0.22384928	-1.676493696
500	PCCA	P05165	propionyl-CoA carboxylase subunit alpha	Biotin	no	no	no	yes	no	yes	0.059693071	0.000244995	0.002649568	0.050848652	2.77429E-05	0.002092522	0.95161993	2.650881764	1.699261834
501	EIF2S1	P05198	eukaryotic translation initiation factor 2 subunit alpha		yes	no	no	no	no	yes	1.90846E-13	0.105977246	4.6975E-31	3.74429E-14	0.054597249	4.98058E-32	-3.199606094	0.485430331	3.685036425
502	RPLP1	P05386	ribosomal protein lateral stalk subunit P1		yes	no	no	no	no	yes	5.04626E-10	0.826191944	6.27405E-14	2.03497E-10	0.761054927	2.77741E-14	-2.25588003	-0.074801196	2.181078834
503	RPLP2	P05387	ribosomal protein lateral stalk subunit P2		yes	no	no	no	no	yes	3.18164E-09	0.47739743	1.98593E-15	1.39836E-09	0.367419043	8.26047E-16	-1.814171484	0.191173164	2.005344648
504	RPLP0	P05388	ribosomal protein lateral stalk subunit P0		yes	no	no	no	no	yes	1.53607E-09	0.89494657	8.64425E-15	6.51453E-10	0.848719153	3.66607E-15	-1.97727091	0.042206782	2.019477692
505	CLEC3B	P05452	C-type lectin domain family 3 member B	Tenecteplase; Lanoteplase; Copper	no	no	yes	no	yes	no	0.000369453	2.66612E-07	2.11419E-18	0.00025562	3.68534E-09	7.63486E-19	0.801439905	-1.363410277	-2.164850182
506	SSB	P05455	small RNA binding exonuclease protection factor La		yes	no	no	no	no	yes	1.88449E-06	0.077524596	1.03083E-06	1.06392E-06	0.036602007	6.59508E-07	-1.551475754	-0.525885379	1.025590375
507	ITGB1	P05556	integrin subunit beta 1	Antithymocyte immunoglobulin (rabbit)	yes	no	no	no	no	yes	1.8797E-10	0.109314243	1.27091E-32	7.14577E-11	0.056762176	1.01926E-33	-3.9346657	0.761393628	4.696059328
508	PRKCB	P05771	protein kinase C beta	Vitamin E; Phosphorylcolamine; Enzastaurin; Ellagic acid; Cholecystokinin; alpha-Tocopherol succinate; D-alpha-	yes	no	no	no	no	yes	2.49503E-12	0.042036859	1.17199E-37	6.63608E-13	0.0174852	1.80655E-39	-4.180629783	0.838550562	5.019180345
509	KRT8	P05787	keratin 8	Tenecteplase; Lanoteplase; Copper	no	yes	no	no	yes	no	3.51955E-09	0.023158649	1.95457E-09	1.55822E-09	0.008216231	1.07426E-09	1.951456929	0.680223911	-1.271233018
510	IGHV4-34	P06331	None	None	no	yes	no	no	yes	no	6.91694E-05	0.381916221	7.83642E-12	4.45041E-05	0.273761423	3.82767E-12	1.261571215	-0.306505859	-1.568077074
511	GSN	P06396	gelsolin	Zinc; Latrunculin A; Copper; Zinc acetate	yes	no	no	no	no	yes	3.16227E-11	0.426528234	1.52624E-15	1.05457E-11	0.318301768	6.29998E-16	-2.171932567	0.191069457	2.363002024
512	PTMA	P06454	prothymosin alpha		no	no	no	yes	no	yes	0.544092921	0.000317357	1.41721E-06	0.517221091	3.81058E-05	9.18263E-07	0.170491296	1.233221837	1.062730541

513	ATP5F1B	P06576	ATP synthase F1 subunit beta	Quercetin; 1-ACETYL-2-CARBOXYPIPERIDINE; AUROVERTIN B; Piceatannol; N1-(2-AMINO-4-METHYLPENTYL)OCTAHYDRO-PYRROLO[1,2-A] PYRIMIDINE; Phenethyl Isothiocyanate	yes	no	no	no	no	yes	0.000214158	0.002775184	3.12221E-06	0.000144293	0.00057591	2.05554E-06	-1.42377335	0.45429417	1.87806752
514	S100A9	P06702	S100 calcium binding protein A9	Calcium; Zinc; Zinc acetate; Zinc chloride; Zinc sulfate, unspecified form	no	no	yes	no	yes	no	0.14212736	2.99511E-05	3.67801E-10	0.125254968	1.77779E-06	1.93983E-10	0.451817354	-1.507956138	-1.959773493
515	EIF4E	P06730	eukaryotic translation initiation factor 4E	7-methyl-GpppA; 7-methyl-7,8-dihydroguanosine-5'-diphosphate; 7-methyl-guanosine-5'-triphosphate; LY2275796; S-[(1-Hydroxy-2,2,5,5-tetramethyl-2,5-dihydro-1H-pyrrol-3-yl)methyl]	yes	no	no	no	no	yes	1.30263E-15	0.003110465	3.69975E-35	1.08011E-16	0.00066522	1.42491E-36	-3.346525032	0.835809475	4.182334507
516	CKM	P06732	creatine kinase, M-type	Creatine; (Diaminomethyl-Methyl-Amino)-Acetic Acid; D-arginine; Phosphocreatine	no	no	yes	no	yes	no	0.001031652	4.07042E-05	1.41235E-10	0.000746978	2.62689E-06	7.31454E-11	0.688508889	-1.260119937	-1.948628826
517	ENO1	P06733	enolase 1	Zinc; Copper; Artenimol; Zinc acetate; Zinc chloride; Zinc sulfate, unspecified form	yes	no	no	no	no	yes	1.04294E-13	0.537645766	4.41721E-20	1.88552E-14	0.43265015	1.46706E-20	-2.692259947	-0.169004438	2.523255509
518	PYGL	P06737	glycogen phosphorylase L		yes	no	no	no	no	yes	4.55269E-12	0.097724534	3.05009E-18	1.28102E-12	0.049419312	1.10699E-18	-1.897009521	0.420933623	2.317943144
519	GPI	P06744	glucose-6-phosphate isomerase	alpha-D-glucose 6-phosphate; 6-phospho-D-gluconic acid; 5-phospho-D-arabinohydroxamic acid; D-glucitol 6-phosphate; 5-Phosphoarabinonic Acid; Glucose-6-Phosphate; D-erythrose 4-phosphate; Fructose-6-phosphate; Copper;	yes	no	no	no	no	yes	1.80522E-12	0.018752102	1.21159E-20	4.62142E-13	0.006313009	3.91419E-21	-2.768998437	0.84849463	3.617493067
520	SERPINE2	P07093	serpin family E member 2		yes	no	no	no	no	yes	2.61838E-05	0.658275573	2.62991E-13	1.62418E-05	0.559250883	1.19639E-13	-1.3945025	0.166840898	1.561343398
521	DBI	P07108	diazepam binding inhibitor, acyl-CoA binding protein	Coenzyme A; Hexadecanal	yes	no	no	no	no	yes	2.9341E-07	0.719239539	2.06578E-15	1.56344E-07	0.629446933	8.63003E-16	-1.81989543	0.138699091	1.958594521
522	FABP1	P07148	fatty acid binding protein 1		no	yes	no	no	yes	no	3.24086E-11	0.000434096	1.20552E-07	1.08611E-11	5.59431E-05	7.3631E-08	2.224588879	0.854982582	-1.369606297
523	P4HB	P07237	prolyl 4-hydroxylase subunit beta	Zinc; Ribostamycin; Copper; Artenimol; Zinc acetate; Zinc chloride; Zinc sulfate, unspecified form	yes	no	no	no	no	yes	2.5279E-07	2.66612E-07	2.98105E-12	1.33454E-07	3.74488E-09	1.42502E-12	-1.60106541	0.932015269	2.533080679
524	H1-0	P07305	H1.0 linker histone		yes	no	no	no	no	yes	3.82213E-05	0.079976101	6.34116E-10	2.4003E-05	0.038085583	3.37889E-10	-1.134702579	0.428540929	1.563243508
525	ADH1A	P07327	alcohol dehydrogenase 1A (class I), alpha polypeptide	NADH; Fomepizole; 4-Iodopyrazole; N-Cyclopentyl-N-Cyclobutylformamide	no	yes	no	no	yes	no	0.000662	0.091268139	8.33154E-11	0.000469129	0.045034437	4.26581E-11	1.332764709	-0.723490623	-2.056255331
526	FES	P07332	FES proto-oncogene, tyrosine kinase	Fostamatinib	yes	no	no	no	no	yes	1.05584E-12	0.049668491	7.63984E-29	2.58817E-13	0.021649791	1.12849E-29	-2.99828058	0.615763382	3.614043961
527	C8A	P07357	complement C8 alpha chain	Zinc; Zinc acetate	no	yes	no	no	yes	no	1.25014E-10	0.026413238	6.97579E-17	4.61652E-11	0.009670093	2.69929E-17	1.593861483	0.443337075	-1.150524408
528	C8G	P07360	complement C8 gamma chain	Zinc; Lauric acid; Citric acid; Zinc acetate	no	yes	no	yes	no	no	2.11819E-05	0.005792263	2.3612E-07	1.30335E-05	0.00143035	1.4593E-07	1.692743619	1.107448358	-0.585295261
529	PRSS1	P07477	serine protease 1	2H-Benzimidazol-2-amine; Bis(5-Amidino-Benzimidazolyl)Methane; 2-{2-hydroxy-[1,1'-biphenyl]-3-yl}-1H-1,3-benzodiazole-5-carboximidamide; Nalpha-(2-Naphthylsulfonylglycyl)-3-Amidino-D,L-Phenylalanine-Isopropylester; CRA_17693; N-Alpha-(2-Naphthylsulfonyl)-N(3-Amidino-L-Phenylalaninyl)Isopipecolinic Acid Methyl Ester; Hemi-Babim; CRA_10991; Monoisopropylphosphorylserine; [4-(6-Chloro-Naphthalene-2-Sulfonyl)-Piperazin-1-Yl]-(3,4,5,6-Tetrahydro-2h-[1,4']Bipyridinyl-4-Yl)-Methanone; Bis(5-Amidino-2-Benzimidazolyl)Methanone; 2-(2-Hydroxy-5-Methoxy-Phenyl)-1h-Benzoimidazole-5-Carboxamidine; 5-Amidino-Benzimidazole; Amylamine; CRA_16847; Bis-Benzamidine; CRA_17312; Zk-806450; [4-({[5-Benzyloxy-1-(3-Carbamimidoyl-Benzyl)-1h-Indole-2-Carbonyl]-Amino}-Methyl)-Phenyl]-Trimethyl-Ammonium; 2-(2-hydroxy-phenyl)-3H-benzoimidazole-5-carboxamidine; CRA-9334; 4-{[1-Methyl-5-(2-Methyl-Benzoimidazol-1-Ylmethyl)-1h-Benzoimidazol-2-Ylmethyl]-Amino}-	no	yes	no	no	yes	no	1.20498E-11	0.408726277	7.17454E-20	3.73997E-12	0.302238915	2.40885E-20	1.300749066	-0.126337003	-1.427086069
530	CTSL	P07711	cathepsin L	L-cysteic acid; Felbinac; Fostamatinib	no	yes	no	no	yes	no	5.98255E-11	0.54394491	1.25651E-08	2.10352E-11	0.438458539	7.22477E-09	1.418445085	0.145040577	-1.273404508
531	APRT	P07741	adenine phosphoribosyltransferase	Adenine; 5-O-phosphono-alpha-D-ribofuranosyl diphosphate; 9-Deazaadenine;	yes	no	no	no	no	yes	1.94375E-15	0.168874044	7.90346E-32	1.78787E-16	0.098259031	7.26961E-33	-2.914022477	0.328222395	3.242244871
532	EPRS1	P07814	glutamyl-prolyl-tRNA synthetase 1	Glutamic acid; Proline; 5'-O-(L-Prolylsulfamoyl)adenosine; 5'-O-(L-Cysteinylsulfamoyl)adenosine; 5'-O-(N-(Alanyl)sulfamoyl)adenosine	yes	no	no	no	no	yes	5.83966E-10	0.943437215	2.01369E-20	2.3655E-10	0.918643667	6.5876E-21	-1.591208496	0.017660888	1.608869384

533	HSP90AA1	P07900	heat shock protein 90 alpha family class A member 1	Rifabutin; Nedocromil; 9-Butyl-8-(2,5-Dimethoxy-Benzyl)-9h-Purin-6-Ylamine; Geldanamycin; 8-(2-Chloro-3,4,5-Trimethoxy-Benzyl)-2-Fluoro-9-Pent-4-Ylnyl-9h-Purin-6-Ylamine; 9-Butyl-8-(3,4,5-Trimethoxybenzyl)-9h-Purin-6-Amine; 4-(1,3-Benzodioxol-5-Yl)-5-(5-Ethyl-2,4-Dihydroxyphenyl)-2h-Pyrazole-3-Carboxylic Acid; 8-(2,5-Dimethoxy-Benzyl)-2-Fluoro-9h-Purin-6-Ylamine; 8-(2,5-Dimethoxy-Benzyl)-2-Fluoro-9-Pent-9h-Purin-6-Ylamine; 9-Butyl-8-(2-Chloro-3,4,5-Trimethoxy-Benzyl)-9h-Purin-6-Ylamine; 4-(1h-Imidazol-4-Yl)-3-(5-Ethyl-2,4-Dihydroxy-Phenyl)-1h-Pyrazole; 9-Butyl-8-(3-Methoxybenzyl)-9h-Purin-6-Amine; 9-Butyl-8-(4-Methoxybenzyl)-9h-Purin-6-Amine; 9-Butyl-8-(2,5-Dimethoxy-Benzyl)-2-Fluoro-9h-Purin-6-Ylamine; Quercetin; 8-Benzo[1,3]Dioxol-,5-Ylmethyl-9-Butyl-2-Fluoro-9h-Purin-6-Ylamine; 8-(2-Chloro-3,4,5-Trimethoxy-Benzyl)-9-Pent-4-Ylnyl-9h-Purin-6-Ylamine; N-[4-(AMINOSULFONYL)BENZYL]-5-(5-CHLORO-2,4-DIHYDROXYPHENYL)-1H-PYRAZOLE-4-CARBOXAMIDE; Tanespimycin; SNX-5422; N-(4-	yes	no	no	no	no	yes	8.06694E-08	0.022848515	8.05873E-26	4.08281E-08	0.008085496	1.75634E-26	-1.580914837	0.606137916	2.187052752
534	YES1	P07947	YES proto-oncogene 1, Src family tyrosine kinase	Dasatinib; Fostamatinib	yes	no	no	no	no	yes	1.89172E-11	0.464962028	1.52893E-32	6.09431E-12	0.355410485	1.26776E-33	-3.968975496	0.327661755	4.296637251
535	LYN	P07948	LYN proto-oncogene, Src family tyrosine kinase	Dasatinib; 1-Tert-Butyl-3-(4-Chloro-Phenyl)-1h-Pyrazolo[3,4-D]Pyrimidin-4-Ylamine; Bosutinib; Ponatinib; Nintedanib;	yes	no	no	no	no	yes	7.80642E-15	0.018123375	6.17134E-31	9.32629E-16	0.006076709	6.65509E-32	-3.137995986	0.670271572	3.808267558
536	TPM2	P07951	tropomyosin 2		yes	no	no	no	no	yes	6.22841E-07	0.01374019	2.60406E-14	3.40065E-07	0.004270852	1.12799E-14	-1.989639823	1.108118191	3.097758014
537	SFTPB	P07988	surfactant protein B		no	yes	no	yes	no	no	0.000542915	8.16546E-06	0.004509482	0.000381787	2.85105E-07	0.003622706	1.297964917	2.000817512	0.702852595
538	THBS1	P07996	thrombospondin 1		yes	no	no	no	no	yes	1.97531E-06	0.349923625	2.9423E-17	1.11788E-06	0.243852531	1.11719E-17	-2.15724668	0.437652565	2.594899245
539	COL1A2	P08123	collagen type I alpha 2 chain	Collagenase clostridium histolyticum	no	yes	no	yes	no	no	2.49443E-06	7.35068E-05	0.00430779	1.41844E-06	5.86189E-06	0.003454821	3.382834916	3.063992146	-0.31884277
540	RHOC	P08134	ras homolog family member C		yes	no	no	no	no	yes	6.40579E-14	0.702965851	7.9559E-32	1.08843E-14	0.608366459	7.35389E-33	-4.258088602	-0.157115347	4.100973255
541	PFKM	P08237	phosphofructokinase, muscle		yes	no	no	no	no	yes	6.74953E-13	0.005930346	6.3643E-25	1.56276E-13	0.00147251	1.53124E-25	-1.655092401	0.520401502	2.175493904
542	HSP90AB1	P08238	heat shock protein 90 alpha family class B member 1	Geldanamycin; 9-Butyl-8-(3,4,5-Trimethoxybenzyl)-9h-Purin-6-Amine; Radicicol; Tanespimycin; SNX-5422; CCT-018159; 8-(6-BROMO-BENZO[1,3]DIOXOL-5-YLSULFANYL)-9-(3-ISOPROPYLAMINO-PROPYL)-ADENINE; 4-{4-[4-(3-AMINOPROPOXY)PHENYL]-1H-PYRAZOL-5-YL}-6-CHLOROBENZENE-1,3-DIOL; (5E)-14-CHLORO-15,17-DIHYDROXY-4,7,8,9,10,11-HEXAHYDRO-2-BENZOXACYCLOPENTADECINE-1,12(3H,13H)-DIONE; (5Z)-12-CHLORO-13,15-DIHYDROXY-4,7,8,9-TETRAHYDRO-2-BENZOXACYCLOTRIDECINE-1,10(3H,11H)-DIONE; (5E)-12-CHLORO-13,15-DIHYDROXY-4,7,8,9-TETRAHYDRO-2-BENZOXACYCLOTRIDECINE-1,10(3H,11H)-DIONE; (5Z)-13-CHLORO-14,16-DIHYDROXY-3,4,7,8,9,10-HEXAHYDRO-1H-2-BENZOXACYCLOTETRADECINE-1,11(12H)-DIONE; METHYL 3-CHLORO-2-{3-[(2,5-DIHYDROXY-4-	yes	no	no	no	no	yes	1.8944E-09	0.002002308	5.88206E-30	8.10293E-10	0.000388304	7.46251E-31	-1.830548157	0.821017985	2.651566142
543	SRPRA	P08240	SRP receptor subunit alpha		yes	no	no	no	no	yes	1.81294E-17	0.292855539	4.16177E-23	4.68226E-19	0.196254346	1.13709E-23	-2.604846964	0.252894272	2.857741235
544	ELANE	P08246	elastase, neutrophil expressed	Alpha-1-proteinase inhibitor; Filgrastim; Mdl 101,146; Freselestat; Elafin	no	no	no	yes	no	yes	0.482662517	1.69854E-05	0.003829161	0.455544188	7.69617E-07	0.003058818	0.259978143	1.296453584	1.036475441
545	SOD3	P08294	superoxide dismutase 3		no	yes	no	no	yes	no	2.93848E-06	0.327171695	6.33245E-10	1.68027E-06	0.224143001	3.37138E-10	1.118221961	-0.200199416	-1.318421378
546	ADH4	P08319	alcohol dehydrogenase 4 (class II), pi polypeptide	NADH; Cyclohexylformamide	no	yes	no	no	yes	no	4.83965E-05	0.534882909	9.36454E-06	3.06562E-05	0.429457415	6.34344E-06	1.760869935	0.308880596	-1.451989339
547	MGP	P08493	matrix Gla protein	Calcium	no	yes	no	no	yes	no	8.73698E-06	0.000409533	3.18751E-14	5.19785E-06	5.19571E-05	1.38794E-14	1.06291743	-0.769630438	-1.832547867
548	ITGA2B	P08514	integrin subunit alpha 2b	Abciximab; Tirofiban; Lefradafiban;	yes	no	no	no	no	yes	2.0245E-08	0.256617199	6.58615E-32	9.65925E-09	0.165225664	5.87889E-33	-4.147062841	0.709699572	4.856762413
549	PLEK	P08567	pleckstrin		yes	no	no	no	no	yes	1.20569E-10	0.150090208	5.01984E-35	4.43599E-11	0.084668015	2.0698E-36	-4.192279416	0.699311998	4.891591415
550	PTPRC	P08575	protein tyrosine phosphatase receptor type C		yes	no	no	no	no	yes	8.81213E-07	0.070598985	2.26947E-05	4.87921E-07	0.032756393	1.57947E-05	-1.583737169	-0.569781272	1.013955897
551	HCK	P08631	HCK proto-oncogene, Src family tyrosine kinase	1-Ter-Butyl-3-P-Tolyl-1h-Pyrazolo[3,4-D]Pyrimidin-4-Ylamine; Phosphonotyrosine; Quercetin; Bosutinib; Fostamatinib	yes	no	no	no	no	yes	2.5279E-07	0.040476998	8.75219E-17	1.33533E-07	0.016726335	3.41442E-17	-2.037794049	0.842440184	2.880234232
552	ITGA5	P08648	integrin subunit alpha 5	Resveratrol	yes	no	no	no	no	yes	6.76477E-15	0.474427839	1.52661E-27	7.93872E-16	0.364365739	2.67E-28	-3.117094638	0.208323069	3.325417707
553	VIM	P08670	vimentin	Artenimol; Phenethyl Isothiocyanate	yes	no	no	no	no	yes	3.17199E-05	0.250706477	2.34128E-06	1.98483E-05	0.160852003	1.53504E-06	-1.51646158	0.202569732	1.719031312
554	RPS17	P08708	ribosomal protein S17	Artenimol	yes	no	no	no	no	yes	6.27298E-09	0.843766055	5.22294E-14	2.85652E-09	0.781831256	2.2979E-14	-1.576502133	0.055646539	1.632148672

555	F7	P08709	coagulation factor VII	Coagulation factor VIIa Recombinant Human; Coagulation Factor IX (Recombinant); (2R)-({4-[AMINO(IMINO)METHYL]PHENYL}AMINO){5-ETHOXY-2-FLUORO-3-[(3R)-TETRAHYDROFURAN-3-YLOXY]PHENYL}ACETICACID; 3-({1-[3-CARBAMIMIDOYL-1-(4-CARBAMIMIDOYL-BENZYLCARBAMOYL)-PROPYLCARBAMOYL]-2-METHYL-BUTYLSULFAMOYL}-METHYL)-BENZOIC ACID; 2-[2-ETHANESULFONYLAMINO-3-(5-PROPOXY-1H-INDOL-3-YL)-PROPIONYLAMINO]-PENTANEDIOIC ACID 5-AMIDE 1-(4-CARBAMIMIDOYL-BENZYLAMIDE); 2-[2-ETHANESULFONYLAMINO-3-(1H-INDOL-3-YL)-PROPIONYLAMINO]-PENTANEDIOIC ACID 5-AMIDE 1-(4-CARBAMIM IDOYL-BENZYLAMIDE); N-[1-(4-CARBAMIMIDOYL-BENZYLCARBAMOYL)-3-METHYLSULFANYL-PROPYL]-3-HYDROXY-2-PROPOXYAMINO-BUTYRAMID; rNAPc2; 2-(4-HYDROXY-	no	yes	no	no	yes	no	2.14682E-09	0.064127052	7.21244E-16	9.24094E-10	0.029172433	2.93465E-16	1.64015469	-0.391055114	-2.031209804
556	GNAI3	P08754	G protein subunit alpha i3		yes	no	no	no	no	yes	1.51183E-08	0.207595408	6.24888E-19	7.11732E-09	0.126823562	2.20848E-19	-1.807221209	0.385660137	2.192881346
557	ANXA5	P08758	annexin A5	Fluocinolone acetonide; L-Alpha-Glycerophosphorylserine; L-thioproline; K201 free base; sn-glycero-3-phosphoethanolamine; 1,4-Dideoxy-O2-Sulfo-Glucuronic Acid; N,O6-Disulfo-Glucosamine; 1,4-Dideoxy-5-Dehydro-O2-	yes	no	no	no	no	yes	1.59554E-16	0.000475936	2.69267E-26	7.66323E-18	6.56546E-05	5.53906E-27	-3.317076143	0.996052136	4.313128279
558	KRT16	P08779	keratin 16	Zinc; Zinc acetate	no	yes	no	no	yes	no	0.00017026	0.389609245	0.000138774	0.00011379	0.28213662	0.000101739	1.72733018	0.447538508	-1.279791672
559	ENO2	P09104	enolase 2	2-Phosphoglycolic Acid	yes	no	no	no	no	yes	9.25586E-10	0.748724506	1.12375E-17	3.82481E-10	0.665279947	4.15995E-18	-2.75346516	-0.134374835	2.619090325
560	ACAA1	P09110	acetyl-CoA acyltransferase 1	Trimetazidine	yes	no	no	no	no	yes	2.1225E-09	0.120244947	1.54049E-08	9.11704E-10	0.064453907	8.94141E-09	-1.862239499	-0.417226091	1.445013408
561	GSTP1	P09211	glutathione S-transferase pi 1	Glutathione; Troglitazone; Clozapine; Etacrynic acid; Clomipramine; (9R,10R)-9-(S-glutathionyl)-10-hydroxy-9,10-dihydrophenanthrene; S-Hydroxycysteine; Cibacron Blue; Glutathione sulfonic acid; Deoxycholic acid; S-(4-nitrobenzyl)glutathione; 2-(N-morpholino)ethanesulfonic acid; S-Hexylglutathione; Carbocisteine; Canfosfamide; Ezatiostat; Exisulind; S-NONYL-CYSTEINE; S-(4-	yes	no	no	no	no	yes	3.67321E-15	0.001387645	3.39854E-30	3.84463E-16	0.000248984	4.2039E-31	-3.22800169	0.935730888	4.163732579
562	SPARC	P09486	secreted protein acidic and cysteine rich	Calcium citrate; Calcium Phosphate; Calcium phosphate dihydrate	yes	no	no	no	no	yes	5.45783E-15	0.002808392	7.49734E-24	6.15768E-16	0.000588706	1.94064E-24	-2.79706303	0.804307445	3.601370475
563	ANXA4	P09525	annexin A4	Fluocinolone acetonide; Copper	yes	no	no	no	no	yes	1.10085E-15	0.000221253	1.50001E-22	8.77883E-17	2.40601E-05	4.23429E-23	-2.401775087	0.72686529	3.128640377
564	INHBB	P09529	inhibin subunit beta B		no	yes	no	no	yes	no	3.26379E-10	0.039971108	1.9111E-09	1.27771E-10	0.016462953	1.04777E-09	1.528314974	0.413178677	-1.115136297
565	CNP	P09543	2',3'-cyclic nucleotide 3' phosphodiesterase		yes	no	no	no	no	yes	1.4753E-09	0.812146947	2.02318E-28	6.24345E-10	0.742229448	3.19932E-29	-2.253575258	0.07892812	2.332503379
566	CTSH	P09668	cathepsin H	N1-(1-Dimethylcarbamoyl-2-Phenyl-Ethyl)-2-Oxo-N4-(2-Pyridin-2-Yl-Ethyl)-Succinamide; p-Toluenesulfonic acid; N-[1-Hydroxycarboxyethyl-Carbonyl]Leucylamino-2-Methyl-Butane; CARBOBENZYLOXY-(L)-LEUCINYL-(L)LEUCINYL	no	yes	no	no	yes	no	8.42113E-07	0.011927763	1.70262E-14	4.65509E-07	0.003577788	7.29034E-15	1.107655208	-0.518010656	-1.625665864
567	ALDOC	P09972	aldolase, fructose-bisphosphate C		yes	no	no	no	no	yes	2.27396E-15	0.512633196	1.29046E-20	2.16371E-16	0.404624842	4.18071E-21	-3.425379336	0.248561407	3.673940743
568	SAA1	P0DJI8	serum amyloid A1		no	yes	no	yes	no	no	1.47449E-05	0.002134871	0.064327136	8.92579E-06	0.000423685	0.056690657	1.886955925	1.251099943	-0.635855982
569	SAA2	P0DJI9	serum amyloid A2		no	yes	no	yes	no	no	1.27441E-05	0.000870129	0.10545496	7.67997E-06	0.000144299	0.094369527	2.292568514	1.666949526	-0.625618988
570	HSPA1A	P0DMV8	heat shock protein family A (Hsp70) member 1A		yes	no	no	no	no	yes	2.56115E-13	0.351665705	6.37833E-27	5.17528E-14	0.245385222	1.20804E-27	-2.883577587	-0.256762115	2.626815473
571	CALM1	P0DP23	calmodulin 1		yes	no	no	no	no	yes	1.73337E-12	0.398647991	2.3223E-33	4.40607E-13	0.292076938	1.58889E-34	-3.282359645	0.281374781	3.563734426
572	RAP2A	P10114	RAP2A, member of RAS oncogene family	Guanosine-5'-Triphosphate; Guanosine-5'-Diphosphate	yes	no	no	no	no	yes	9.43971E-11	0.504624392	1.05379E-26	3.43626E-11	0.395559673	2.06269E-27	-3.219992443	0.275465996	3.495458439
573	SRGN	P10124	serglycin		yes	no	no	no	no	yes	2.14985E-05	0.882700916	3.16875E-18	1.32478E-05	0.830306797	1.15149E-18	-1.798650865	0.072241186	1.870892051
574	RO60	P10155	Ro60, Y RNA binding protein		yes	no	no	no	no	yes	3.88165E-14	0.591444513	2.71599E-25	6.19094E-15	0.488106604	6.21465E-26	-2.485598557	0.136014308	2.621612866
575	RRAS	P10301	RAS related		yes	no	no	no	no	yes	5.11669E-13	0.22729033	1.6953E-26	1.13601E-13	0.141811864	3.38752E-27	-3.102875355	0.389328654	3.49220401
576	HLA-C	P10321	major histocompatibility complex, class I, C		yes	no	no	no	no	yes	4.84694E-07	0.039604359	2.00334E-12	2.62662E-07	0.01627601	9.53107E-13	-1.504446548	0.53835656	2.042803108
577	H1-4	P10412	H1.4 linker histone, cluster member	Copper	yes	no	yes	no	no	no	0.000167594	0.008177055	0.038280447	0.00011178	0.002220371	0.032938182	-1.979989169	-1.071118927	0.908870242
578	TXN	P10599	thioredoxin	Phenethyl Isothiocyanate	yes	no	no	no	no	yes	7.38732E-12	0.082289781	8.00694E-31	2.17235E-12	0.03959753	8.92482E-32	-2.007090508	0.380541908	2.387632416
579	C7	P10643	complement C7		no	yes	no	yes	no	no	0.000127599	0.007928273	0.000640343	8.42951E-05	0.002130252	0.000485988	1.475901405	1.065419368	-0.410482037
580	PRKAR1A	P10644	protein kinase cAMP-dependent type I regulatory subunit alpha	(Rp)-cAMPS; Cyclic GMP; Cyclic adenosine monophosphate; GEM-231	yes	no	no	no	no	yes	2.97403E-11	0.512794652	3.29264E-32	9.8775E-12	0.405295468	2.80479E-33	-3.741040318	0.279726847	4.020767165
581	CHGA	P10645	chromogranin A		no	yes	no	no	yes	no	1.04614E-10	0.000821613	2.46044E-08	3.82999E-11	0.000131786	1.44594E-08	1.993632276	0.857618097	-1.136014178
582	TFPI	P10646	tissue factor pathway inhibitor	Coagulation factor VIIa Recombinant Human; Dalteparin; Andexanet alfa	no	yes	no	no	yes	no	4.40696E-06	0.849223669	5.52603E-08	2.5639E-06	0.788042625	3.3001E-08	1.155910929	0.051563603	-1.104347326
583	PF4V1	P10720	platelet factor 4 variant 1		yes	no	no	no	no	yes	6.78712E-06	0.241607535	2.8412E-15	3.99896E-06	0.153162819	1.19467E-15	-3.27659303	-0.831897257	2.444695773
584	ESD	P10768	esterase D	Glutathione	yes	no	no	no	no	yes	3.4864E-06	0.065016911	1.40302E-18	2.00938E-06	0.02978346	5.02848E-19	-1.249393386	0.487376382	1.736769768
585	HSPD1	P10809	heat shock protein family D (Hsp60) member 1	Copper	yes	no	no	no	no	yes	1.16504E-08	0.012664272	1.85119E-12	5.42664E-09	0.003844614	8.79879E-13	-3.065912199	0.892986085	3.958898284
586	HSPA8	P11142	heat shock protein family A (Hsp70) member 8	Dasatinib; (2R,3R,4S,5R)-2-[6-amino-8-[(3,4-dichlorophenyl)methylamino]purin-9-yl]-5-(hydroxymethyl)oxolane-3,4-diol; Copper; Artenimol	yes	no	no	no	no	yes	1.14663E-11	0.279487948	6.61929E-27	3.54847E-12	0.184763442	1.25668E-27	-3.6108712	-0.419617041	3.191254158
587	SLC2A3	P11169	solute carrier family 2 member 3		yes	no	no	no	no	yes	3.52717E-09	0.208365248	3.55686E-22	1.56461E-09	0.127549366	1.01694E-22	-4.252833406	0.75595877	5.008792176
588	EPB41	P11171	erythrocyte membrane protein band 4.1		yes	no	no	no	no	yes	3.04289E-06	0.08891827	5.76297E-08	1.74135E-06	0.043673496	3.4442E-08	-1.708385676	-0.628073997	1.080311679
589	PYGB	P11216	glycogen phosphorylase B	Pyridoxal phosphate; Alvocidib	yes	no	no	no	no	yes	2.31909E-09	0.181707573	1.25755E-15	1.00245E-09	0.10736143	5.16809E-16	-2.354715834	0.541041583	2.895757417

590	PYGM	P11217	glycogen phosphorylase, muscle associated	Pyridoxal phosphate; Beta-D-Glucopyranose Spirohydantoin; (5S,7R,8S,9S,10R)-3-Amino-8,9,10-trihydroxy-7-(hydroxymethyl)-6-oxa-1,3-diazaspiro[4.5]decane-2,4-dione; alpha-D-glucose 6-phosphate; N-beta-D-glucopyranosylacetamide; Monofluorophosphate ion; Beta-D-Glucose; 3,8,9,10-tetrahydroxy-7-hydroxymethyl-6-oxa-1,3-diaza-spiro[4.5]decane-2,4-dione; Nojirimycine Tetrazole; Indirubin-5-sulphonate; 2-Deoxy-Glucose-6-Phosphate; C-(1-hydrogyl-beta-D-glucopyranosyl) formamide; alpha-D-glucopyranosyl-2-carboxylic acid amide; alpha-D-glucose-1-phosphate; 8,9,10-Trihydroxy-7-hydroxymethyl-2-thioxo-6-oxa-1,3-diaza-spiro[4.5]decan-4-one; 4-{2,4-Bis[(3-Nitrobenzoyl)Amino]Phenoxy}Phthalic Acid; 2-(Beta-D-Glucopyranosyl)-5-Methyl-1,2,3-Benzimidazole; N-acetyl-N'-beta-D-glucopyranosyl urea; 2-(Beta-D-Glucopyranosyl)-5-Methyl-1,3,4-Benzothiazole; C-(1-Azido-Alpha-D-Glucopyranosyl) Formamide; 2-(Beta-D-Glucopyranosyl)-5-Methyl-1,3,4-Oxadiazole; CP-320626; (3,4,5-Trihydroxy-	yes	no	no	no	no	yes	2.57748E-17	0.011186792	4.05016E-26	8.64221E-19	0.003284568	8.47835E-27	-2.864608088	0.583845941	3.44845403
591	RALB	P11234	RAS like proto-oncogene B		yes	no	no	no	no	yes	2.65823E-11	0.01822362	9.91211E-37	8.78048E-12	0.006118578	2.6049E-38	-3.123155872	0.81948687	3.942642742
592	SPTB	P11277	spectrin beta, erythrocytic		yes	no	no	no	no	yes	0.000418035	0.972485538	1.35362E-05	0.000290749	0.963232164	9.27969E-06	-1.472823757	-0.019903753	1.452920004
593	LAMP1	P11279	lysosomal associated membrane protein 1		yes	no	no	no	no	yes	3.18715E-08	0.310330303	1.10663E-09	1.54953E-08	0.210689905	5.96685E-10	-2.315499902	0.246010494	2.561510395
594	FGFR1	P11362	fibroblast growth factor receptor 1	Palifermin; Sorafenib; Heparin; 3-[4-(1-formylpiperazin-4-yl)-benzylidenyl]-2-indolinone; XL999; 3-(3-methoxybenzyl)-1H-pyrrolo[2,3-b]pyridine; (E)-[4-(3,5-Difluorophenyl)-3H-pyrrolo[2,3-b]pyridin-3-ylidene](3-methoxyphenyl)methanol; 3-[(3-(2-CARBOXYETHYL)-4-METHYLPYRROL-2-YL)METHYLENE]-2-INDOLINONE; Regorafenib; Ponatinib; Lenvatinib; Nintedanib; Fostamatinib;	no	yes	no	no	yes	no	7.30715E-12	0.086123362	4.32156E-14	2.14546E-12	0.041988553	1.8935E-14	2.099327427	0.406108559	-1.693218868
595	G6PD	P11413	glucose-6-phosphate dehydrogenase	Glycolic acid; Nicotinamide adenine dinucleotide phosphate; 16-Bromoepiandrosterone; Artenimol	yes	no	no	no	no	yes	2.39454E-15	0.00086807	5.52533E-34	2.32185E-16	0.000143171	2.97922E-35	-3.222657023	0.954348053	4.177005076
596	MTHFD1	P11586	methylenetetrahydrofolate dehydrogenase, cyclohydrolase and formyltetrahydrofolate synthetase 1	Tetrahydrofolic acid; NADH; LY374571; Nicotinamide adenine dinucleotide phosphate; LY249543	yes	no	no	no	no	yes	3.2641E-10	0.01480115	2.2901E-30	1.27931E-10	0.004701226	2.70828E-31	-2.767561351	0.859172351	3.626733701
597	SCGB1A1	P11684	secretoglobin family 1A member 1	4,4'-BIS([H]METHYLSULFONYL)-2,2',5,5'-TETRACHLOROBIPHENYL	no	yes	no	no	yes	no	1.93601E-07	0.037641111	9.02807E-06	1.01143E-07	0.015176574	6.09916E-06	1.575152457	0.521928924	-1.053223533
598	ADH5	P11766	alcohol dehydrogenase 5 (class III), chi polypeptide	NADH; Lauric acid; 12-Hydroxydodecanoic Acid; S-Hydroxymethyl Glutathione	yes	no	no	no	no	yes	3.86611E-13	3.52253E-06	1.59773E-20	8.14563E-14	9.41681E-08	5.2051E-21	-1.223134383	0.860290145	2.083424528
599	PABPC1	P11940	poly(A) binding protein cytoplasmic 1		yes	no	no	no	no	yes	1.09435E-09	0.186227845	1.03664E-33	4.57673E-10	0.110707264	6.27403E-35	-1.862689222	0.318725741	2.181414963
600	HARS1	P12081	histidyl-tRNA synthetase 1	Histidine	yes	no	no	no	no	yes	1.55974E-13	0.026783734	8.09506E-21	2.9753E-14	0.009890686	2.5822E-21	-1.598808154	0.416986244	2.015794398
601	COL6A1	P12109	collagen type VI alpha 1 chain		yes	no	yes	no	no	no	0.000500275	0.002044267	0.879309907	0.000350215	0.000398294	0.870943116	-1.200814589	-1.169566404	0.031248185
602	FCGR2A	P12318	Fc gamma receptor IIa	Cetuximab; Etanercept; Human immunoglobulin G; Abciximab; Alemtuzumab; Daclizumab; Bevacizumab;	yes	no	no	no	no	yes	1.17523E-14	0.004575738	2.72074E-25	1.50698E-15	0.001084328	6.23785E-26	-2.674676939	0.746744604	3.421421543
603	ANXA3	P12429	annexin A3	Fluocinolone acetonide; Ethanolamine; Difluocortolone	yes	no	no	no	no	yes	1.53885E-17	0.037641111	1.66294E-23	3.90464E-19	0.015179243	4.41542E-24	-2.865797402	0.500161163	3.365958565
604	MYH7	P12883	myosin heavy chain 7	4-[4-(2,5-DIOXO-PYRROLIDIN-1-YL)-PHENYLAMINO]-4-HYDROXY-BUTYRIC ACID	no	yes	no	no	yes	no	5.48077E-05	0.935275385	0.001180594	3.4916E-05	0.906034786	0.000914198	1.051181436	0.034605815	-1.016575621
605	SRC	P12931	SRC proto-oncogene, non-receptor tyrosine kinase	Dasatinib; RU84687; RU79256; N6-Benzyl Adenosine-5'-Diphosphate; RU85493; RU78262; Phosphonotyrosine; Malonic acid; RU83876; RU90395; RU79072; RU78783; 1-Tert-Butyl-3-(4-Chloro-Phenyl)-1h-Pyrazolo[3,4-D]Pyrimidin-4-Ylamine; PASBN; 2-[4-[(Z)-2-Acetamido-3-oxo-3-[[(3S)-2-oxo-1-[(4-phenylphenyl)methyl]azepan-3-yl]amino]prop-1-enyl]-2-formylphenyl]acetic acid; PAS219; DPI59; RU82197; Phenylphosphate; RU78300; RU79073; RU82209; ISO24; RU85053; RU78299; Oxalic Acid; RU78191; Citric acid; Paratoulene phosphate; 4-[(4-METHYL-1-PIPERAZINYL)METHYL]-N-[3-[[4-(3-PYRIDINYL)-2-PYRIMIDINYL]AMINO]PHENYL]-BENZAMIDE; Purvalanol A; XL228; KX-01; Bosutinib; 1-[1-(3-aminophenyl)-3-tert-butyl-1H-pyrazol-5-yl]-3-naphthalen-1-ylurea; 1-[1-(3-aminophenyl)-3-tert-butyl-1H-pyrazol-5-yl]-3-phenylurea; 3-[4-AMINO-1-(1-METHYLETHYL)-1H-PYRAZOLO[3,4-D]PYRIMIDIN-3-YL]PHENOL; PD-168393; [4-({4-[(5-cyclopropyl-1H-pyrazol-3-	yes	no	no	no	no	yes	5.31154E-14	0.014969765	1.17199E-37	8.92878E-15	0.004788697	1.86134E-39	-3.846804926	0.823321241	4.670126167
606	LAMP2	P13473	lysosomal associated membrane protein 2		yes	no	no	no	no	yes	3.99166E-06	0.505186377	2.2268E-05	2.30963E-06	0.396458	1.54877E-05	-1.748500814	-0.151049798	1.597451017
607	RNH1	P13489	ribonuclease/angiogenin inhibitor 1		yes	no	no	no	no	yes	4.01605E-15	0.116323248	8.06382E-28	4.27627E-16	0.062035552	1.38477E-28	-3.245730692	0.460529779	3.706260471

608	EEF2	P13639	eukaryotic translation elongation factor 2	Adenosine-5-Diphosphoribose; Diphthamide; Guanosine-5'-Diphosphate; N~2~,N~2~-DIMETHYL-N~1~-(6-OXO-5,6-DIHYDROPHENANTHRIDIN-2-YL)GLYCINAMIDE; Esketamine; Moxetumomab Pasudotox	yes	no	no	no	no	yes	7.07884E-11	0.000602973	1.12401E-20	2.5403E-11	8.89845E-05	3.62108E-21	-1.259483142	0.593261653	1.852744795
609	KRT10	P13645	keratin 10	Zinc; Copper; Zinc acetate	no	yes	no	no	yes	no	5.74375E-05	0.181916334	1.45903E-05	3.66174E-05	0.107649629	1.00354E-05	1.669830184	0.563994511	-1.105835674
610	KRT13	P13646	keratin 13		no	yes	no	yes	no	no	1.12327E-07	0.003464202	9.46267E-05	5.76647E-08	0.000761277	6.87298E-05	1.992686472	1.038820417	-0.953866056
611	KRT5	P13647	keratin 5	Zinc; Zinc acetate	no	yes	no	no	yes	no	0.00039116	0.52341163	3.42312E-05	0.000271171	0.41597825	2.40875E-05	1.487595991	0.299668756	-1.187927235
612	C6	P13671	complement C6		no	yes	no	no	yes	no	9.7426E-07	0.027603028	3.50405E-16	5.42089E-07	0.010268276	1.39718E-16	1.81763744	0.7445893	-1.07304814
613	ACP5	P13686	acid phosphatase 5, tartrate resistant		no	yes	no	no	yes	no	2.59673E-09	0.898422104	5.37229E-13	1.12953E-09	0.85282932	2.49019E-13	1.868254076	0.03712628	-1.831127796
614	TPT1	P13693	tumor protein, translationally-controlled 1	Calcium citrate; Calcium Phosphate; Calcium phosphate dihydrate	yes	no	no	no	no	yes	1.32205E-12	0.69560622	1.43539E-31	3.28866E-13	0.601051681	1.3658E-32	-2.868034852	0.121341824	2.989376676
615	ALAD	P13716	aminolevulinate dehydratase	Aminolevulinic acid; Delta-Amino Valeric Acid; Laevulinic Acid; 4-Oxosebacic Acid; Porphobilinogen; 3-(2-Aminoethyl)-4-(Aminomethyl)Heptanedioic Acid; 4,7-Dioxosebacic Acid; 5-hydroxyvaleric acid	yes	no	no	no	no	yes	7.12188E-14	0.001920959	3.06566E-20	1.22301E-14	0.000367306	1.00985E-20	-2.056986467	0.829872184	2.886858651
616	GYS1	P13807	glycogen synthase 1		yes	no	no	no	no	yes	5.08193E-17	2.96829E-05	1.57018E-25	1.98027E-18	1.73224E-06	3.50748E-26	-2.565251979	0.860012975	3.425264953
617	CD59	P13987	CD59 molecule (CD59 blood group)		yes	no	yes	no	no	no	0.00155193	0.006536131	0.6203208	0.001149005	0.001657823	0.600427567	-1.164778547	-1.064836574	0.099941973
618	CD99	P14209	CD99 molecule (Xg blood group)		yes	no	yes	no	no	no	1.20737E-10	8.16546E-06	0.014295531	4.44762E-11	2.92284E-07	0.01194425	-2.132965743	-1.469025089	0.663940654
619	HCLS1	P14317	hematopoietic cell-specific Lyn substrate 1		yes	no	no	no	no	yes	2.15943E-16	0.092443803	4.88086E-13	1.11543E-17	0.045865865	2.25798E-13	-1.910880852	-0.357975886	1.552904965
620	NID1	P14543	nidogen 1	Urokinase	yes	no	no	no	no	yes	6.70737E-12	0.270637358	2.36863E-16	1.95113E-12	0.177440986	9.36933E-17	-2.521533183	0.304426689	2.825959873
621	PKM	P14618	pyruvate kinase M1/2	Pyruvic acid; L-Phospholactate; 2-Phosphoglycolic Acid; 6-(2-fluorobenzyl)-2,4-dimethyl-4,6-dihydro-5H-thieno[2',3':4,5]pyrrolo[2,3-d]pyridazin-5-one; 1-[(2,6-difluorophenyl)sulfonyl]-4-(2,3-dihydro-1,4-benzodioxin-6-ylsulfonyl)piperazine; 1-(2,3-dihydro-1,4-benzodioxin-6-ylsulfonyl)-4-[(4-methoxyphenyl)sulfonyl]piperazine; Copper;	yes	no	no	no	no	yes	1.04838E-15	0.016223655	8.50374E-33	8.31295E-17	0.005278017	6.5117E-34	-4.026259991	0.779201933	4.805461924
622	MYL6B	P14649	myosin light chain 6B	4-[4-(2,5-DIOXO-PYRROLIDIN-1-YL)-PHENYLAMINO]-4-HYDROXY-BUTYRIC ACID	yes	no	no	no	no	yes	1.40745E-11	0.539575511	2.03234E-19	4.43853E-12	0.434447523	6.98933E-20	-3.299857976	-0.251839299	3.048018678
623	DARS1	P14868	aspartyl-tRNA synthetase 1	Aspartic acid	yes	no	no	no	no	yes	1.23392E-11	0.126264465	7.97699E-31	3.83537E-12	0.068481452	8.85529E-32	-3.004793414	0.481813571	3.486606985
624	AKR1B1	P15121	aldo-keto reductase family 1 member B	Glutathione; NADH; Sulindac; Inhibitor Idd 384; alpha-D-glucose 6-phosphate; Alrestatin; Fidarestat; (S,R)-fidarestat; Zenarestat; Tolrestat; N-Acetylalanine; Sorbinil; IDD552; Cacodylic acid; Nicotinamide adenine dinucleotide phosphate; Citric acid; Ranirestat; Pimagedine; QR-333; Exisulind; (2-{[(4-BROMO-2-FLUOROBENZYL)AMINO]CARBONYL}-5-CHLOROPHENOXY)ACETIC ACID; (5-CHLORO-2-{[(3-NITROBENZYL)AMINO]CARBONYL}PHENOXY)ACETIC ACID; Lidorestat; {3-[(5-CHLORO-1,3-BENZOTHIAZOL-2-YL)METHYL]-2,4-DIOXO-3,4-DIHYDROPYRIMIDIN-1(2H)-YL}ACETIC ACID; 3-[5-(3-nitrophenyl)thiophen-2-yl]propanoic acid; CP-744809; (R)-minalrestat; 4-[3-(3-NITROPHENYL)-1,2,4-OXADIAZOL-5-YL]BUTANOIC ACID; {4-[(CARBOXYMETHOXY)CARBONYL]-3,3-DIOXIDO-1-OXONAPHTHO[1,2-D]ISOTHIAZOL-2(1H)-YL}ACETIC ACID; 2-(CARBOXYMETHYL)-1-OXO-1,2-DIHYDRONAPHTHO[1,2-	yes	no	no	no	no	yes	9.89085E-12	0.003892114	1.29778E-16	3.0161E-12	0.000876475	5.09231E-17	-1.588569531	0.708673084	2.297242615
625	RAC2	P15153	Rac family small GTPase 2		yes	no	no	no	no	yes	5.26085E-19	0.134259406	2.0776E-20	4.76742E-21	0.073973465	6.80609E-21	-2.906345579	0.416240916	3.322586495
626	GSPT1	P15170	G1 to S phase transition 1	Guanosine-5'-Diphosphate	yes	no	no	no	no	yes	3.74629E-11	0.168874044	6.84817E-32	1.27818E-11	0.098151063	6.17483E-33	-3.095752328	0.478215497	3.573967825
627	VAV1	P15498	vav guanine nucleotide exchange factor 1		yes	no	no	no	no	yes	1.50801E-11	0.391199714	1.4534E-29	4.78984E-12	0.283961007	1.94929E-30	-2.737202709	0.2583103	2.995513009
628	NME1	P15531	NME/NM23 nucleoside diphosphate kinase 1	Zinc; 2'-Deoxyguanosine-5'-Triphosphate; Selenocysteine; 2',3'-Dehydro-2',3'-Deoxy-Thymidine 5'-Diphosphate; Adenosine Phosphonoacetic Acid; Stavudine triphosphate; Thymidine-5'- Diphosphate; 2'-Deoxyguanosine-5'-Diphosphate; 2',3'-dideoxy-3'-fluoro-urididine-5'-diphosphate; Guanosine-5'-Diphosphate; 3'-Deoxy 3'-Amino Adenosine-5'-Diphosphate; Zidovudine diphosphate; Copper; Zinc	yes	no	no	no	no	yes	1.05049E-10	0.068924363	1.36619E-21	3.8507E-11	0.03183283	4.12272E-22	-1.847484475	0.464925991	2.312410466
629	CD1E	P15812	CD1e molecule		no	yes	no	no	yes	no	1.60414E-11	0.003038802	9.27904E-09	5.13876E-12	0.00064714	5.28909E-09	2.319145942	0.86712815	-1.452017792
630	RPS2	P15880	ribosomal protein S2	Copper	yes	no	no	no	no	yes	1.23078E-10	0.021325164	3.32401E-11	4.53944E-11	0.007420809	1.6733E-11	-2.308267541	-0.666917114	1.641350426
631	SELP	P16109	selectin P	Heparin; N-acetyl-alpha-neuraminic acid; Dalteparin; Nadroparin; Crizanlizumab	yes	no	no	no	no	yes	3.89415E-12	0.017340893	7.82021E-27	1.07455E-12	0.005767202	1.50947E-27	-4.06656815	1.091263736	5.157831885
632	SPN	P16150	sialophorin		yes	no	no	no	no	yes	3.34282E-08	0.69206141	1.12714E-12	1.63279E-08	0.59673442	5.3267E-13	-1.575932345	0.124193485	1.70012583
633	CBR1	P16152	carbonyl reductase 1	Rutin; Resveratrol; Heptaethylene glycol; 2-(2-{2-[2-(2-{2-[2-(2-Ethoxy-Ethoxy)-Ethoxy]-Ethoxy}-Ethoxy)-Ethoxy]-Ethoxy}-Ethoxy)-Ethanol, Polyethyleneglycol Peg400; Quercetin; 3-(4-Amino-1-Tert-Butyl-1h-Pyrazolo[3,4-D]Pyrimidin-3-Yl)Phenol; Curcumin; Curcumin sulfate	yes	no	no	no	no	yes	1.27731E-14	0.833364923	1.04506E-23	1.68018E-15	0.768884306	2.74169E-24	-2.094574181	0.048671298	2.143245479
634	PECAM1	P16284	platelet and endothelial cell adhesion molecule 1		yes	no	no	no	no	yes	2.15766E-09	0.07256014	2.96935E-31	9.29739E-10	0.033830713	3.01375E-32	-3.986108788	0.970893384	4.957002172
635	PPP3CB	P16298	protein phosphatase 3 catalytic subunit beta		no	no	no	yes	no	yes	0.018010332	9.20893E-06	1.35219E-11	0.014640026	3.51753E-07	6.68436E-12	-0.494820713	1.435015724	1.929836437
636	H1-2	P16403	H1.2 linker histone, cluster member		yes	no	no	no	no	yes	5.22286E-05	0.025849021	0.008036549	3.32256E-05	0.009368864	0.006579993	-1.991159619	-0.833079656	1.158079963
637	CRISP2	P16562	cysteine rich secretory protein 2		no	yes	no	no	yes	no	3.29228E-10	0.327440739	1.72217E-09	1.29185E-10	0.22462405	9.40287E-10	1.177545591	0.142488924	-1.035056667
638	ATP2A2	P16615	ATPase sarcoplasmic/endoplasmic reticulum Ca2+ transporting 2	Istaroxime	yes	no	no	no	no	yes	3.48557E-05	0.1224058	0.000370197	2.18262E-05	0.065889484	0.000277606	-2.335102217	-0.726441802	1.608660416
639	CD36	P16671	CD36 molecule (CD36 blood group)		yes	no	no	no	no	yes	4.76105E-09	0.618570364	3.43694E-21	2.13136E-09	0.518231356	1.06519E-21	-3.753467114	0.286707705	4.040174819
640	PLCG2	P16885	phospholipase C gamma 2		yes	no	no	no	no	yes	1.65852E-14	0.068924363	1.69196E-31	2.29202E-15	0.031739065	1.64826E-32	-2.773500645	0.457415447	3.230916092
641	STMN1	P16949	stathmin 1		yes	no	no	no	no	yes	0.000113098	0.898530936	1.55929E-07	7.43569E-05	0.853339756	9.56626E-08	-1.071810514	-0.043192155	1.028618359
642	YBX3	P16989	Y-box binding protein 3		yes	no	no	no	no	yes	0.000104039	0.737034803	1.04847E-06	6.83067E-05	0.65120882	6.7127E-07	-1.302124725	-0.127588743	1.174535982

643	PRKACA	P17612	protein kinase cAMP-activated catalytic subunit alpha	Pentanal; Balanol Analog 2; 3-[(3-sec-butyl-4-hydroxybenzoyl)amino]azepan-4-yl 4-(2-hydroxy-5-methoxybenzoyl)benzoate; Phosphonothreonine; Balanol Analog 1; 3,5-Diiodotyrosine; Balanol; Dexfosfoserine; S,S-(2-Hydroxyethyl)Thiocysteine; Hydroxyfasudil; (2S)-1-(3H-Indol-3-yl)-3-{[5-(6-isoquinolinyl)-3-pyridinyl]oxy}-2-propanamine; (2S)-1-{[5-(1H-Indazol-5-yl)-3-pyridinyl]oxy}-3-(7aH-indol-3-yl)-2-propanamine; (1S)-2-(1H-INDOL-3-YL)-1-[({5-[(E)-2-PYRIDIN-4-YLVINYL]PYRIDIN-3-YL}OXY)METHYL]ETHYLAMINE; (2S)-1-(6H-INDOL-3-YL)-3-{[5-(7H-PYRAZOLO[3,4-C]PYRIDIN-5-YL)PYRIDIN-3-YL]OXY}PROPAN-2-AMINE; (1S)-1-(1H-INDOL-3-YLMETHYL)-2-(2-PYRIDIN-4-YL-[1,7]NAPHTYRIDIN-5-YLOXY)-EHYLAMINE; N-[(1S)-2-AMINO-1-(2,4-DICHLOROBENZYL)ETHYL]-5-[2-(METHYLAMINO)PYRIMIDIN-4-YL]THIOPHENE-2-CARBOXAMIDE; 3-(1H-indol-3-yl)-4-{1-[2-(1-methylpyrrolidin-2-yl)ethyl]-1H-indol-3-yl}-1H-pyrrole-2,5-dione; (4R,2S)-5'-(4-(4-	yes	no	no	no	no	yes	4.78734E-13	0.316129663	1.75786E-27	1.0477E-13	0.215289028	3.12226E-28	-2.943740991	0.312591885	3.256332877
644	PFKL	P17858	phosphofructokinase, liver type		yes	no	no	no	no	yes	1.90846E-13	0.002233632	3.9252E-35	3.74425E-14	0.000447334	1.54732E-36	-3.069860898	0.916570254	3.986431152
645	IGFBP3	P17936	insulin like growth factor binding protein 3	Alitretinoin; Mecasermin; Lonafarnib	no	yes	no	no	yes	no	2.80257E-15	0.423692945	2.05362E-15	2.83178E-16	0.315609969	8.56992E-16	1.706992556	0.102125707	-1.604866849
646	TCP1	P17987	t-complex 1		yes	no	no	no	no	yes	7.2761E-13	0.930752225	6.38343E-31	1.70776E-13	0.899544402	6.94166E-32	-3.904069735	-0.038005466	3.866064269
647	ARF4	P18085	ADP ribosylation factor 4	Guanosine-5'-Diphosphate	yes	no	no	no	no	yes	6.00545E-18	0.142107684	8.26638E-24	9.79593E-20	0.079327896	2.14993E-24	-4.162607704	0.544006874	4.706614577
648	RPL7	P18124	ribosomal protein L7		yes	no	no	no	no	yes	0.000346013	0.972423879	1.51773E-05	0.000238933	0.961741001	1.04598E-05	-1.11696911	-0.010660637	1.106308473
649	PTPRA	P18433	protein tyrosine phosphatase receptor type A		yes	no	no	no	no	yes	7.29768E-13	0.689624047	1.13132E-21	1.71613E-13	0.593695373	3.38831E-22	-2.740281714	-0.122847317	2.617434397
650	RHCE	P18577	Rh blood group CcEe antigens		no	no	yes	no	yes	no	0.121656482	0.002127658	1.64858E-08	0.106491249	0.00042129	9.59865E-09	0.609801963	-1.307469126	-1.917271088
651	PGAM1	P18669	phosphoglycerate mutase 1	Copper; Artenimol	yes	no	no	no	no	yes	1.27731E-14	0.92598814	5.7359E-13	1.68036E-15	0.89368135	2.66393E-13	-2.742888978	0.04149449	2.784383468
652	PAM	P19021	peptidylglycine alpha-amidating monooxygenase	Ascorbic acid; N-Alpha-Acetyl-3,5-Diiodotyrosylglycine; N-alpha-Acetyl-3,5-diiodotyrosyl-D-threonine	no	yes	no	no	yes	no	1.71772E-09	0.00017356	2.93193E-17	7.3083E-10	1.77728E-05	1.11193E-17	1.183445633	-0.599811095	-1.783256729
653	CDH2	P19022	cadherin 2		no	yes	no	no	yes	no	9.29321E-18	1.00615E-05	1.97663E-14	1.82795E-19	4.0217E-07	8.49943E-15	2.338726111	0.612874994	-1.725851116
654	CD58	P19256	CD58 molecule		yes	no	no	no	no	yes	1.03411E-07	0.136947192	4.31532E-09	5.28065E-08	0.075764622	2.40109E-09	-1.836600041	-0.485333629	1.351266412
655	EIF2AK2	P19525	eukaryotic translation initiation factor 2 alpha kinase 2	Fostamatinib	yes	no	no	no	no	yes	3.39651E-09	0.091478184	4.36196E-19	1.49896E-09	0.045262427	1.53173E-19	-2.275543887	0.615319277	2.890863163
656	TYMP	P19971	thymidine phosphorylase	Tipiracil	yes	no	no	no	no	yes	7.30474E-16	0.228993769	2.27457E-23	5.26259E-17	0.143185954	6.12187E-24	-3.094534933	0.325737618	3.420272551
657	EIF2S2	P20042	eukaryotic translation initiation factor 2 subunit beta		yes	no	no	no	no	yes	1.13258E-11	0.748724506	2.49054E-29	3.49473E-12	0.665445378	3.46441E-30	-3.337931248	0.12807736	3.466008608
658	ANXA7	P20073	annexin A7		yes	no	no	no	no	yes	1.3967E-10	0.024551856	8.39873E-35	5.18302E-11	0.008844656	3.84355E-36	-3.080655	0.833526609	3.914181609
659	RAB5A	P20339	RAB5A, member RAS oncogene family	L-methionine (S)-S-oxide; Guanosine-5'-Triphosphate; Guanosine-5'-Diphosphate	yes	no	no	no	no	yes	3.44377E-11	0.804487861	6.67605E-22	1.15937E-11	0.733771665	1.96621E-22	-2.596597353	-0.084675673	2.51192168
660	PSMB1	P20618	proteasome 20S subunit beta 1	Bortezomib; (3AR,6R,6AS)-6-((S)-((S)-CYCLOHEX-2-ENYL)(HYDROXY)METHYL)-6A-METHYL-4-OXO-HEXAHYDRO-2H-FURO[3,2-C]PYRROLE-6-CARBALDEHYDE; Carfilzomib	yes	no	no	no	no	yes	9.1484E-14	0.001648645	2.63475E-25	1.61662E-14	0.000305526	6.00488E-26	-2.055505675	0.719586176	2.775091851
661	PZP	P20742	None	None	yes	no	no	no	no	yes	0.005236666	0.680343995	0.000189548	0.004069272	0.583948653	0.000139993	-1.046112262	0.173447239	1.2195595
662	CAST	P20810	calpastatin	Calcium; Artenimol	yes	no	no	no	no	yes	1.19811E-10	0.045543939	1.92263E-21	4.39725E-11	0.019357129	5.84543E-22	-1.228492327	0.34322855	1.571720877
663	C4BPB	P20851	complement component 4 binding protein beta	Zinc; Zinc acetate; Zinc chloride; Zinc sulfate, unspecified form	no	yes	no	no	yes	no	1.06146E-09	0.210444367	1.23556E-08	4.43439E-10	0.129489556	7.09873E-09	1.310617169	0.222434254	-1.088182915
664	ATP6V1B2	P21281	ATPase H+ transporting V1 subunit B2	Gallium nitrate; 4-(2-Aminoethyl)Benzenesulfonyl Fluoride	yes	no	no	no	no	yes	1.38813E-19	0.189088527	3.35278E-26	7.54762E-22	0.113007597	6.95775E-27	-2.720498155	0.245458709	2.965956864
665	ATP6V1C1	P21283	ATPase H+ transporting V1 subunit C1	D-tartaric acid; Thonzonium	yes	no	no	no	no	yes	3.91763E-13	0.258486306	1.24216E-30	8.27222E-14	0.167255354	1.42958E-31	-2.738366564	0.310124037	3.048490602
666	CSRP1	P21291	cysteine and glycine rich protein 1	Artenimol	yes	no	no	no	no	yes	2.13526E-12	0.181809492	7.048E-36	5.57275E-13	0.107504027	2.17156E-37	-4.477756958	0.585396798	5.063153756
667	CD9	P21926	CD9 molecule	C31G	yes	no	no	no	no	yes	5.99929E-08	0.772257596	7.1914E-27	2.99285E-08	0.693877124	1.36855E-27	-3.796737	0.194028008	3.990765008
668	OSBP	P22059	oxysterol binding protein		yes	no	no	no	no	yes	8.67215E-10	0.00045463	2.34183E-29	3.57181E-10	6.05624E-05	3.22572E-30	-1.810644556	0.938800013	2.749444569
669	PCMT1	P22061	protein-L-isoaspartate (D-aspartate) O-methyltransferase	S-adenosyl-L-homocysteine	yes	no	no	no	no	yes	5.35714E-13	0.246478319	1.45052E-32	1.20153E-13	0.157692518	1.18302E-33	-2.632774625	0.294516205	2.92729083
670	FBL	P22087	fibrillarin		yes	no	yes	no	no	no	0.000273063	6.42717E-10	0.141549941	0.000186208	2.91217E-12	0.128209484	-1.522512109	-2.085534274	-0.563022165
671	TNXB	P22105	tenascin XB		no	yes	no	no	yes	no	2.42491E-17	0.386895334	3.65107E-22	8.02076E-19	0.279083539	1.04553E-22	3.925615498	-0.24301505	-4.168630548
672	CDH3	P22223	cadherin 3		no	yes	no	yes	no	no	4.16383E-06	9.00195E-06	0.421190936	2.41491E-06	3.34177E-07	0.399625655	2.608669016	2.866752176	0.25808316
673	UBA1	P22314	ubiquitin like modifier activating enzyme 1	Hexatantalum Dodecabromide; Quercetin	yes	no	no	no	no	yes	6.37497E-18	0.000497299	2.22975E-27	1.06875E-19	6.94011E-05	4.03113E-28	-2.838209009	0.775311261	3.61352027
674	NME2	P22392	NME/NM23 nucleoside diphosphate kinase 2		yes	no	no	no	no	yes	5.7514E-13	0.003310215	3.80299E-28	1.31182E-13	0.000718438	6.20334E-29	-2.429489989	0.797213308	3.226703297
675	GAL	P22466	galanin and GMAP prepropeptide		no	no	yes	no	yes	no	0.227659283	2.71501E-05	6.43327E-10	0.205790789	1.46391E-06	3.43088E-10	0.278971456	-1.057214183	-1.336185639
676	HNRNPA2B1	P22626	heterogeneous nuclear ribonucleoprotein A2/B1	Copper; Artenimol	yes	no	yes	no	no	no	1.28903E-08	0.000131771	0.004703639	6.03337E-09	1.22994E-05	0.003785076	-1.706855248	-1.036714562	0.670140686
677	CBL	P22681	Cbl proto-oncogene		yes	no	yes	no	no	no	1.12873E-07	5.33463E-05	0.017299374	5.80475E-08	3.8916E-06	0.014532415	-2.713761253	-2.152095712	0.56166554
678	IGFBP4	P22692	insulin like growth factor binding protein 4		no	yes	no	no	yes	no	2.97438E-16	0.000291646	3.1507E-13	1.68463E-17	3.43579E-05	1.44044E-13	2.33904774	0.63413151	-1.70491623
679	PRKACB	P22694	protein kinase cAMP-activated catalytic subunit beta	Phosphonothreonine; Fostamatinib	yes	no	no	no	no	yes	4.24537E-12	0.024108573	4.00905E-39	1.18109E-12	0.008618787	3.08808E-41	-3.497135331	0.791949496	4.289084827
680	PROZ	P22891	protein Z, vitamin K dependent plasma glycoprotein		no	yes	no	no	yes	no	2.01213E-06	0.47739743	4.73197E-11	1.14054E-06	0.367289382	2.39493E-11	1.519519541	-0.21453287	-1.734052411
681	ITGA6	P23229	integrin subunit alpha 6		yes	no	no	no	no	yes	3.22554E-09	0.085940286	4.74478E-34	1.41912E-09	0.041821417	2.51536E-35	-4.015758794	0.939342739	4.955101533
682	PPIB	P23284	peptidylprolyl isomerase B	Proline; 1,4-Dithiothreitol	yes	no	no	no	no	yes	9.91594E-14	0.678066984	2.59478E-20	1.77022E-14	0.581595288	8.51211E-21	-1.54176033	0.065908839	1.607669169
683	HRC	P23327	histidine rich calcium binding protein		no	yes	no	no	yes	no	3.26947E-08	0.042064014	3.49839E-07	1.59252E-08	0.017572733	2.18749E-07	1.56353492	0.536608846	-1.026926074
684	ANOS1	P23352	anosmin 1		no	no	no	yes	no	yes	0.887016978	4.72398E-06	9.51467E-10	0.879782585	1.30568E-07	5.113E-10	-0.029815821	1.26649528	1.2963111
685	RPS3	P23396	ribosomal protein S3		yes	no	no	no	no	yes	3.05203E-07	0.026745129	4.47069E-11	1.62904E-07	0.009864311	2.26067E-11	-1.166271276	0.424125417	1.590396693
686	JAK1	P23458	Janus kinase 1	Myricetin; 2-tert-butyl-9-fluoro-1,6-dihydrobenzo[h]imidazo[4,5-f]isoquinolin-7-one; Ruxolitinib; Tofacitinib; Baricitinib; Fostamatinib; Fedratinib; Upadacitinib	yes	no	no	no	no	yes	7.11909E-10	0.032019534	3.2618E-25	2.89667E-10	0.012346454	7.61137E-26	-1.830199927	0.551579861	2.381779789
687	AHCY	P23526	adenosylhomocysteinase	(1'R,2'S)-9-(2-Hydroxy-3'-Keto-Cyclopenten-1-yl)Adenine; 3'-Oxo-Adenosine; D-Eritadenine; Copper	yes	no	no	no	no	yes	8.60769E-13	0.039221411	1.36724E-17	2.05929E-13	0.01604469	5.08609E-18	-1.65242526	0.444076	2.09650126
688	CFL1	P23528	cofilin 1	Dodecyldimethylamine N-oxide; Copper; Artenimol	yes	no	no	no	no	yes	3.96899E-15	0.097409869	7.03887E-23	4.20817E-16	0.049124234	1.9455E-23	-2.945008586	-0.43856403	2.506444556
689	BDNF	P23560	brain derived neurotrophic factor	CX717; Copper; Chondroitin sulfate; Esketamine	yes	no	no	no	no	yes	7.7211E-06	0.133089497	1.06811E-06	4.56899E-06	0.073208269	6.84325E-07	-1.936532977	-0.676554654	1.259978323
690	EIF4B	P23588	eukaryotic translation initiation factor 4B		yes	no	no	no	no	yes	2.7739E-11	0.173367909	1.37106E-17	9.20024E-12	0.101726979	5.10652E-18	-2.233391764	-0.363952844	1.86943892

691	DTYMK	P23919	deoxythymidylate kinase	Thymidine monophosphate; 2',3'-Dideoxythymidine-5'-Monophosphate; 3'-Fluoro-3'-deoxythymidine 5'-monophosphate; 3'-deoxy-3'-aminothymidine monophosphate; p1-(5'-adenosyl)p5-(5'-thymidyl)pentaphosphate; Zidovudine monophosphate; P1-(5'-Adenosyl)P5-(5'-(3'azido-3'-Deoxythymidyl))Pentaphosphate; Phosphoaminophosphonic Acid-Adenylate	yes	no	no	no	no	yes	2.04761E-15	0.123892688	4.22362E-27	1.91123E-16	0.066970537	7.8272E-28	-2.10947198	0.30176361	2.41123559
692	IGFBP6	P24592	insulin like growth factor binding protein 6		no	yes	no	no	yes	no	1.97703E-08	0.258486306	6.52999E-09	9.40592E-09	0.167465948	3.66591E-09	1.665375063	0.26396881	-1.401406253
693	IGFBP5	P24593	insulin like growth factor binding protein 5		no	yes	no	no	yes	no	3.97568E-11	0.090999098	1.58716E-18	1.36546E-11	0.044819221	5.70286E-19	1.509639248	-0.295460536	-1.805099784
694	ACP1	P24666	acid phosphatase 1	Adenine; 4-Nitrophenyl Phosphate	yes	no	no	no	no	yes	2.77262E-10	0.382203365	4.11753E-27	1.08292E-10	0.274140429	7.59328E-28	-2.143385117	0.245186183	2.3885713
695	MYL9	P24844	myosin light chain 9		yes	no	no	no	no	yes	4.97663E-10	0.402062746	1.95788E-23	2.00238E-10	0.295671879	5.22516E-24	-3.270629948	0.370036556	3.640666504
696	GRK2	P25098	G protein-coupled receptor kinase 2	ATP	yes	no	no	no	no	yes	2.549E-15	0.549333819	5.80862E-22	2.54092E-16	0.4448324	1.69495E-22	-1.998062769	-0.113588151	1.884474618
697	MPST	P25325	mercaptopyruvate sulfurtransferase		yes	no	no	no	no	yes	2.32789E-16	0.003823739	1.17682E-28	1.2418E-17	0.000859345	1.77029E-29	-2.760305131	0.726524307	3.486829438
698	DNAJB1	P25685	DnaJ heat shock protein family (Hsp40) member B1		yes	no	no	no	no	yes	2.47513E-08	0.49393452	9.95105E-22	1.19214E-08	0.383599351	2.97134E-22	-1.775596793	0.204638392	1.980235184
699	DNAJB2	P25686	DnaJ heat shock protein family (Hsp40) member B2		yes	no	no	no	no	yes	3.55013E-14	0.983814923	6.92653E-22	5.51741E-15	0.977574139	2.04312E-22	-1.933543871	-0.004622887	1.928920984
700	ATP5F1A	P25705	ATP synthase F1 subunit alpha	Quercetin; 1-ACETYL-2-CARBOXYPIPERIDINE; AUROVERTIN B; Piceatannol; N1-(2-AMINO-4-METHYLPENTYL)OCTAHYDRO-PYRROLO[1,2-A] PYRIMIDINE;	yes	no	no	no	no	yes	0.000331905	0.000393864	3.3145E-07	0.000228439	4.90768E-05	2.06499E-07	-1.526027034	0.794650193	2.320677228
701	CTSS	P25774	cathepsin S	Morpholine-4-Carboxylic Acid [1s-(2-Benzyloxy-1r-Cyano-Ethylcarbamoyl)-3-Methyl-Butyl]Amide; Morpholine-4-Carboxylic Acid (1-(3-Benzenesulfonyl-1-Phenethylallylcarbamoyl)-3-Methylbutyl)-Amide; Morpholine-4-Carboxylic Acid [1-(2-Benzylsulfanyl-1-Formyl-Ethylcarbamoyl)-2-Phenyl-Ethyl]-Amide; N-[1-(AMINOMETHYL)CYCLOPROPYL]-3-(MORPHOLIN-4-YLSULFONYL)-N~2~-[(1S)-2,2,2-TRIFLUORO-1-(4-FLUOROPHENYL)ETHYL]-L-ALANINAMIDE; N-(1-CYANOCYCLOPROPYL)-3-({[(2S)-5-OXOPYRROLIDIN-2-YL]METHYL}SULFONYL)-N~2~-[(1S)-2,2,2-TRIFLUORO-1-(4-FLUOROPHENYL)ETHYL]-L-ALANINAMIDE; N-[1-(AMINOMETHYL)CYCLOPROPYL]-3-(BENZYLSULFONYL)-N~2~-[(1S)-2,2,2-TRIFLUORO-1-(4-HYDROXYPHENYL)ETHYL]-L-ALANINAMIDE; N~2~-1,3-BENZOXAZOL-2-YL-3-CYCLOHEXYL-N-{2-[(4-METHOXYPHENYL)AMINO]ETHYL}-L-	no	yes	no	no	yes	no	3.60401E-12	0.912424584	2.57603E-15	9.8306E-13	0.877283628	1.08084E-15	1.585354761	-0.019959223	-1.605313984
702	PSMA3	P25788	proteasome 20S subunit alpha 3	(3AR,6R,6AS)-6-((S)-((S)-CYCLOHEX-2-ENYL)(HYDROXY)METHYL)-6A-METHYL-4-OXO-HEXAHYDRO-2H-FURO[3,2-C]PYRROLE-6-CARBALDEHYDE; Phenethyl	yes	no	no	no	no	yes	1.83331E-11	0.44585444	1.1183E-16	5.89784E-12	0.337976655	4.37288E-17	-2.03356441	0.226489608	2.260054018
703	PSMA4	P25789	proteasome 20S subunit alpha 4	(3AR,6R,6AS)-6-((S)-((S)-CYCLOHEX-2-ENYL)(HYDROXY)METHYL)-6A-METHYL-4-OXO-HEXAHYDRO-2H-FURO[3,2-C]PYRROLE-6-	yes	no	no	no	no	yes	7.14061E-17	0.000243451	9.17233E-29	3.04131E-18	2.73566E-05	1.36733E-29	-2.068864014	0.670364239	2.739228253
704	S100P	P25815	S100 calcium binding protein P	Cromoglicic acid	no	yes	no	no	yes	no	9.32142E-07	0.244495423	5.22581E-09	5.1781E-07	0.155759205	2.92428E-09	1.176438204	-0.355412092	-1.531850297
705	COL5A3	P25940	collagen type V alpha 3 chain		no	yes	no	no	yes	no	6.46341E-06	0.524115762	9.48507E-12	3.80131E-06	0.417121303	4.65873E-12	1.21907015	0.174401535	-1.044668615
706	MSN	P26038	moesin		yes	no	no	no	no	yes	1.4301E-11	0.306566297	4.90259E-15	4.51645E-12	0.207462673	2.07477E-15	-1.67908818	-0.219503401	1.459584779
707	DDX6	P26196	DEAD-box helicase 6	D-tartaric acid	yes	no	no	no	no	yes	4.08299E-17	0.007115837	1.18174E-28	1.48002E-18	0.001824904	1.78305E-29	-2.456378264	0.543256951	2.999635215
708	U2AF2	P26368	U2 small nuclear RNA auxiliary factor 2		yes	no	yes	no	no	no	0.001134198	0.000149116	0.795465538	0.000824825	1.43913E-05	0.782129687	-1.162676732	-1.240013039	-0.077336307
709	S100A4	P26447	S100 calcium binding protein A4	Trifluoperazine; Copper	yes	no	no	no	no	yes	2.49035E-12	0.134414913	7.56807E-22	6.61236E-13	0.074120049	2.23579E-22	-2.110879428	-0.340670444	1.770208984
710	PTBP1	P26599	polypyrimidine tract binding protein 1		yes	no	no	no	no	yes	7.20784E-13	0.62891314	2.82068E-28	1.6852E-13	0.528891159	4.52434E-29	-2.500302388	0.128935397	2.629237784
711	TARS1	P26639	threonyl-tRNA synthetase 1	Threonine	yes	no	no	no	no	yes	2.42957E-10	0.017485904	1.86527E-34	9.39024E-11	0.005839199	8.95872E-36	-1.844185507	0.533197407	2.377382913
712	VARS1	P26640	valyl-tRNA synthetase 1	Valine	yes	no	no	no	no	yes	6.62432E-14	0.127594862	2.66021E-30	1.12857E-14	0.069260827	3.20623E-31	-2.88792189	0.403149627	3.291071517
713	EEF1G	P26641	eukaryotic translation elongation factor 1 gamma		yes	no	no	no	no	yes	6.03669E-15	0.003618025	5.95231E-35	7.02958E-16	0.00079672	2.58913E-36	-3.292215365	0.841991057	4.134206422
714	STOM	P27105	stomatin		yes	no	no	no	no	yes	6.6754E-08	0.164526751	2.95064E-14	3.35736E-08	0.094973757	1.28213E-14	-3.112111749	-0.686449881	2.425661869
715	MAOB	P27338	monoamine oxidase B	Amphetamine; Phentermine; Tranylcypromine; Phenelzine; Zonisamide; Selegiline; Pioglitazone; Moclobemide; Isocarboxazid; Ephedra sinica root; Rasagiline; MMDA; 4-Methoxyamphetamine; Metamfetamine; Pargyline; Isatin; (R)-N-methyl-N-2-propynyl-1-indanamine; Farnesol; N-Dodecyl-N,N-Dimethyl-3-Ammonio-1-Propanesulfonate; Flavin adenine dinucleotide; N-Propargyl-1(S)-Aminoindan; Dodecyldimethylamine N-oxide; 5-Hydroxy-N-Propargyl-1(R)-Aminoindan; N-METHYL-N-[(1R)-1-METHYL-2-PHENYLETHYL]PROP-2-EN-1-AMINE; Nialamide; Nomifensine; Zimelidine; Safinamide; 7-[(3-CHLOROBENZYL)OXY]-2-OXO-2H-CHROMENE-4-CARBALDEHYDE; 7-[(3-CHLOROBENZYL)OXY]-4-[(METHYLAMINO)METHYL]-2H-CHROMEN-2-ONE; N-(2-AMINOETHYL)-P-CHLOROBENZAMIDE; (1Z)-4-(4-FLUOROPHENYL)-2-METHYLIDENEBUTAN-1-IMINE; 4-	yes	no	yes	no	no	no	6.75516E-05	2.31536E-05	0.15543088	4.33714E-05	1.14352E-06	0.141627322	-1.292319122	-1.835535002	-0.54321588
716	YWHAQ	P27348	tyrosine 3-monooxygenase/tryptophan 5-monooxygenase activation protein theta	Phenethyl Isothiocyanate	yes	no	no	no	no	yes	5.69639E-11	0.021955192	2.17497E-36	1.99516E-11	0.00772958	6.20858E-38	-4.183817852	1.091746171	5.275564023
717	CD82	P27701	CD82 molecule		yes	no	no	no	no	yes	6.87539E-11	0.497904398	4.76937E-12	2.45795E-11	0.388628476	2.29932E-12	-1.55428697	-0.145905834	1.408381137

718	MAP4	P27816	microtubule associated protein 4	Paclitaxel; Docetaxel; Artenimol	yes	no	no	no	no	yes	5.88599E-15	0.047344335	4.5406E-25	6.80076E-16	0.020384355	1.07189E-25	-2.6202158	0.455102276	3.075318076
719	CFP	P27918	complement factor properdin		no	yes	no	no	yes	no	0.005799223	0.900212104	1.23295E-11	0.004524813	0.857920035	6.07814E-12	1.5114671	0.085338836	-1.426128263
720	PIK3R1	P27986	phosphoinositide-3-kinase regulatory subunit 1	Isoprenaline; SF1126; Enzastaurin;	yes	no	no	no	no	yes	8.35725E-16	0.932942427	9.421E-14	6.17232E-17	0.902506607	4.20466E-14	-2.467171185	0.031900341	2.499071527
721	LOX	P28300	lysyl oxidase		no	yes	no	no	yes	no	6.75516E-05	0.210315732	2.86724E-10	4.33496E-05	0.129168163	1.50572E-10	1.060174476	-0.302406645	-1.362581121
722	LAP3	P28838	leucine aminopeptidase 3	Leucine Phosphonic Acid; (2S)-3-[(R)-[(1S)-1-amino-3-phenylpropyl](hydroxy)phosphoryl]-2-	yes	no	no	no	no	yes	7.07188E-16	0.61430308	3.40651E-21	5.06278E-17	0.513542901	1.05421E-21	-2.780963585	0.150965646	2.931929231
723	TPP2	P29144	tripeptidyl peptidase 2		yes	no	no	no	no	yes	9.29321E-18	0.002347551	1.7319E-34	1.87019E-19	0.000474403	8.16121E-36	-2.496799724	0.568672847	3.065472572
724	IMPA1	P29218	inositol monophosphatase 1	Lithium cation; L-Myo-Inositol-1-Phosphate; Lithium citrate; Lithium succinate; Lithium carbonate	yes	no	yes	no	no	no	6.10659E-08	0.000520921	0.057959216	3.04914E-08	7.38777E-05	0.050816077	-1.938198043	-1.434600496	0.503597546
725	PTPN6	P29350	protein tyrosine phosphatase non-receptor type 6	Tiludronic acid	yes	no	no	no	no	yes	1.17509E-12	0.90492225	1.85625E-29	2.90711E-13	0.865970001	2.53164E-30	-2.888293121	-0.0398371	2.848456021
726	TKT	P29401	transketolase	Copper	yes	no	no	no	no	yes	2.47575E-09	0.413459672	3.40923E-19	1.07578E-09	0.306675796	1.18481E-19	-1.983941623	0.251689325	2.235630947
727	PML	P29590	PML nuclear body scaffold	Arsenic trioxide	yes	no	no	no	no	yes	3.79618E-05	0.906180866	0.000133228	2.38229E-05	0.867585034	9.75516E-05	-1.105834554	0.049790533	1.155625087
728	EEF1D	P29692	eukaryotic translation elongation factor 1 delta		yes	no	no	no	no	yes	4.05985E-13	0.007215614	1.5696E-28	8.6458E-14	0.001857031	2.40383E-29	-2.874673273	0.831645455	3.706318727
729	AQP1	P29972	aquaporin 1 (Colton blood group)	Acetazolamide; B-nonylglucoside; Mersalyl	no	no	yes	no	yes	no	0.53136697	0.00087119	1.61219E-08	0.50440136	0.000144869	9.36486E-09	0.23065422	-1.539450979	-1.770105199
730	PRDX6	P30041	peroxiredoxin 6	2-(N-morpholino)ethanesulfonic acid;	yes	no	no	no	no	yes	4.02594E-11	0.397923472	2.34075E-24	1.38454E-11	0.291365804	5.89326E-25	-2.04655936	0.220621534	2.267180894
731	PRDX5	P30044	peroxiredoxin 5	Auranofin; Diminazene	yes	no	no	no	no	yes	2.79533E-17	0.095041762	3.20065E-31	9.62598E-19	0.047757732	3.3023E-32	-3.273734035	0.410845878	3.684579913
732	RPL12	P30050	ribosomal protein L12		yes	no	no	no	no	yes	3.88622E-05	0.524115762	1.85994E-08	2.44584E-05	0.417487771	1.08546E-08	-1.010298612	0.185799851	1.196098463
733	ECHS1	P30084	enoyl-CoA hydratase, short chain 1	Hexanoyl-CoA; Octanoyl-Coenzyme A; Acetoacetyl-CoA; 4-(N,N-Dimethylamino)cinnamoyl-CoA; Omega-3-carboxylic acids	yes	no	no	no	no	yes	0.003484507	0.138906945	0.00010636	0.002650009	0.077226471	7.74449E-05	-1.021993508	0.339644325	1.361637833
734	CMPK1	P30085	cytidine/uridine monophosphate kinase 1	Gemcitabine; Cytidine-5'-Monophosphate; Uridine-5'-Diphosphate; P1-(adenosine-5'-P5-(uridine-5')pentaphosphate; Tetrafluoroaluminate Ion	yes	no	no	no	no	yes	3.66547E-11	0.0295823	4.2401E-26	1.24397E-11	0.011125197	8.89519E-27	-2.25012289	0.615815176	2.865938067
735	PEBP1	P30086	phosphatidylethanolamine binding protein 1	Copper	yes	no	no	no	no	yes	2.03807E-16	0.048305619	3.81281E-24	1.03427E-17	0.02092441	9.72637E-25	-1.924064232	0.329312821	2.253377053
736	PDIA3	P30101	protein disulfide isomerase family A member 3	Zinc; Copper; Zinc acetate; Zinc chloride; Zinc sulfate, unspecified form	yes	no	no	no	no	yes	1.82384E-12	0.002667797	2.33216E-24	4.67737E-13	0.00054879	5.85419E-25	-1.922708247	0.632381279	2.555089526
737	PPP2R1A	P30153	protein phosphatase 2 scaffold subunit Aalpha	2,6,8-Trimethyl-3-Amino-9-Benzyl-9-Methoxynonanoic Acid; (2S,3S,4E,6E,8S,9S)-3-amino-9-methoxy-2,6,8-trimethyl-10-phenyldeca-4,6-dienoic	yes	no	no	no	no	yes	5.00883E-13	0.391526965	1.27036E-28	1.10299E-13	0.284375951	1.92827E-29	-3.241287964	0.288644048	3.529932012
738	FCER1G	P30273	Fc epsilon receptor Ig	Benzylpenicilloyl polylysine	yes	no	no	no	no	yes	1.75116E-14	0.082059394	4.70259E-21	2.44385E-15	0.039449487	1.46809E-21	-2.702952146	0.497614036	3.200566182
739	NMT1	P30419	N-myristoyltransferase 1	(1-Methyl-1h-Imidazol-2-Yl)-(3-Methyl-4-{3-[(Pyridin-3-Ylmethyl)-Amino]-Propoxy}-Benzofuran-2-Yl)-Methanone	yes	no	no	no	no	yes	1.19878E-14	0.444828778	4.90885E-25	1.54804E-15	0.336997606	1.16771E-25	-3.56693786	-0.248485563	3.318452298
740	ADSL	P30566	adenylosuccinate lyase		yes	no	no	no	no	yes	1.7308E-10	0.584761737	4.27048E-22	6.54048E-11	0.480633344	1.22871E-22	-2.164478897	-0.15839846	2.006080437
741	PKLR	P30613	pyruvate kinase L/R	Pyruvic acid; 2-Phosphoglycolic Acid; beta-D-fructofuranose 1,6-bisphosphate	yes	no	no	no	no	yes	1.13933E-11	0.107007765	5.16964E-26	3.52071E-12	0.055225122	1.09389E-26	-3.113930797	0.58323688	3.697167677
742	SRI	P30626	sorcin	Calcium citrate; Calcium Phosphate; Calcium phosphate dihydrate	yes	no	no	no	no	yes	5.51966E-08	0.489435635	3.5912E-26	2.73857E-08	0.379661897	7.50134E-27	-2.158090317	0.246660678	2.404750995
743	SERPINB1	P30740	serpin family B member 1		yes	no	no	no	no	yes	6.23475E-13	0.176554283	7.19691E-33	1.43227E-13	0.103916635	5.38057E-34	-3.509726622	0.458857687	3.968584309
744	GDI1	P31150	GDP dissociation inhibitor 1		no	no	no	yes	no	yes	0.672161997	0.001903273	1.25344E-06	0.651147417	0.0003622	8.05901E-07	-0.17188373	1.831470229	2.003353959
745	PRKAR2B	P31323	protein kinase cAMP-dependent type II regulatory subunit beta	Cyclic adenosine monophosphate	yes	no	no	no	no	yes	5.14221E-17	0.633096636	9.51123E-22	2.02706E-18	0.532983031	2.8357E-22	-3.271489469	0.158388747	3.429878216
746	SLC6A4	P31645	solute carrier family 6 member 4	Fluvoxamine; Amphetamine; Phentermine; Tramadol; Citalopram; Benzatropine; Venlafaxine; Atomoxetine; Amitriptyline; Protriptyline; Loxapine; Meperidine; Imipramine; Fluoxetine; Duloxetine; Dextromethorphan; Nortriptyline; Amoxapine; Fenfluramine; Mazindol; Trazodone; Verapamil; Paroxetine; Trimipramine; Minaprine; Pseudoephedrine; Cocaine; Cyclobenzaprine; Dopamine; Sertraline; Sibutramine; Chlorpheniramine; Doxepin; Nefazodone; Desipramine; Escitalopram; Dexfenfluramine; Aripiprazole; Clomipramine; Ephedra sinica root; MMDA; Midomafetamine; 4-Methoxyamphetamine; Metamfetamine; Nomifensine; Zimelidine; Amineptine; Bicifadine; Milnacipran; OPC-14523; CRx-119; Amitifadine; Lumateperone; Mianserin; Tesofensine; Tapentadol; Vilazodone; Desvenlafaxine; Dexmethylphenidate;	yes	no	no	no	no	yes	9.78272E-12	0.002148866	3.17744E-15	2.97427E-12	0.000428053	1.33893E-15	-1.916251367	0.481623924	2.39787529
747	DNAJA1	P31689	DnaJ heat shock protein family (Hsp40) member A1		yes	no	no	no	no	yes	2.93639E-13	0.487991017	1.4234E-26	5.98719E-14	0.378099066	2.83778E-27	-2.131220473	0.15849342	2.289713894
748	HNRNPH1	P31943	heterogeneous nuclear ribonucleoprotein H1	Copper	yes	no	no	no	no	yes	5.22351E-15	0.080520124	3.77309E-16	5.84598E-16	0.038527073	1.50787E-16	-2.281091812	-0.409534586	1.871557226
749	YWHAB	P31946	tyrosine 3-monooxygenase/tryptophan 5-monooxygenase activation protein beta	Copper; Phenethyl Isothiocyanate	yes	no	no	no	no	yes	2.07268E-11	0.012487363	8.64705E-37	6.70545E-12	0.00378525	2.16553E-38	-3.855077398	1.070310792	4.92538819
750	KIF5B	P33176	kinesin family member 5B		yes	no	no	no	no	yes	7.81132E-15	0.186960112	1.08484E-29	9.37925E-16	0.111312002	1.42548E-30	-3.576239869	0.407662918	3.983902787
751	HSPA4	P34932	heat shock protein family A (Hsp70) member 4	Phenethyl Isothiocyanate	yes	no	no	no	no	yes	1.58472E-15	0.011049028	6.27411E-26	1.38582E-16	0.003219087	1.35034E-26	-2.169948601	0.482786043	2.652734644
752	GRK5	P34947	G protein-coupled receptor kinase 5		yes	no	no	no	no	yes	2.67984E-14	0.45044508	4.91314E-30	3.99487E-15	0.342477047	6.12194E-31	-4.362113814	0.310340152	4.672453965
753	GPC1	P35052	glypican 1		no	yes	no	yes	no	no	1.03355E-06	0.000175984	0.065032032	5.76014E-07	1.81805E-05	0.057370805	1.271579811	1.030454659	-0.241125152
754	RDX	P35241	radixin	1D-myo-inositol 1,4,5-trisphosphate	yes	no	no	no	no	yes	2.69444E-10	0.684077172	1.73454E-21	1.04994E-10	0.587680253	5.24999E-22	-1.692478433	0.097973607	1.79045204
755	RPL22	P35268	ribosomal protein L22		yes	no	yes	no	no	no	6.64433E-07	0.000135287	0.055998721	3.63677E-07	1.27502E-05	0.04899571	-2.118922164	-1.632102705	0.486819459
756	KRT9	P35527	keratin 9	Zinc; Copper; Zinc acetate; Zinc chloride; Zinc sulfate, unspecified form	no	yes	no	no	yes	no	2.64881E-05	0.225837741	0.000200103	1.64426E-05	0.140082813	0.00014806	1.906627164	0.593377255	-1.313249908
757	SAA4	P35542	serum amyloid A4, constitutive		no	yes	no	no	yes	no	2.61855E-11	0.604352357	2.55283E-13	8.6138E-12	0.502485988	1.16017E-13	1.107459965	-0.064195818	-1.171655783
758	MYH9	P35579	myosin heavy chain 9	Artenimol	yes	no	no	no	no	yes	6.33516E-12	0.328029638	1.52555E-19	1.83424E-12	0.225473929	5.20943E-20	-3.250780438	-0.368652348	2.88212809
759	MYH10	P35580	myosin heavy chain 10		yes	no	no	no	no	yes	1.60517E-08	0.818943664	2.10046E-19	7.57856E-09	0.751780636	7.26167E-20	-2.382876096	-0.096322832	2.286553264
760	ADD1	P35611	adducin 1		yes	no	no	no	no	yes	2.54289E-13	0.893634071	3.91195E-28	5.11573E-14	0.846364508	6.41652E-29	-3.208958863	-0.049423173	3.15953569
761	ADD2	P35612	adducin 2		yes	no	no	no	no	yes	3.20431E-06	0.240940672	9.38444E-06	1.83954E-06	0.15240289	6.36118E-06	-1.700148534	-0.388461863	1.31168667
762	BSG	P35613	basigin (Ok blood group)		yes	no	no	no	no	yes	2.11369E-09	0.886626619	5.60663E-14	9.06962E-10	0.83560642	2.47688E-14	-2.247851003	-0.055506788	2.192344216
763	NUP214	P35658	nucleoporin 214		no	yes	no	no	yes	no	1.0927E-08	0.906958463	5.56063E-13	5.06988E-09	0.86956235	2.58001E-13	1.432882059	0.03242966	-1.400452399
764	MYH11	P35749	myosin heavy chain 11	Tetrafluoroaluminate Ion	yes	no	no	no	no	yes	3.44124E-10	0.155516859	9.1337E-14	1.35498E-10	0.088645314	4.06816E-14	-2.558318705	-0.510584676	2.047734029
765	PPM1A	P35813	protein phosphatase, Mg2+/Mn2+ dependent 1A		yes	no	no	no	no	yes	2.86962E-21	0.000898086	6.1837E-34	3.9007E-24	0.000150563	3.44629E-35	-3.182473751	0.638556709	3.82103046
766	IGFALS	P35858	insulin like growth factor binding protein acid labile subunit	Zinc; Copper; Zinc acetate; Zinc chloride; Zinc sulfate, unspecified form	no	yes	no	no	yes	no	5.59861E-14	0.504624392	2.01385E-17	9.43671E-15	0.395393986	7.55537E-18	2.204146919	0.154934714	-2.049212205
767	KRT2	P35908	keratin 2	Zinc; Copper; Anthralin; Zinc acetate	no	yes	no	no	yes	no	9.34628E-05	0.169636295	1.48145E-05	6.10663E-05	0.099076205	1.01963E-05	1.649881736	0.594942593	-1.054939143
768	PSMC2	P35998	proteasome 26S subunit, ATPase 2		yes	no	no	no	no	yes	2.65721E-11	0.078196218	5.9209E-22	8.7555E-12	0.037096257	1.73039E-22	-2.308305364	0.521441691	2.829747055
769	CHI3L1	P36222	chitinase 3 like 1		no	yes	no	yes	no	no	6.78712E-06	0.000429899	0.129849823	3.99659E-06	5.51252E-05	0.117200203	1.338932146	1.062657389	-0.276274758
770	ATP6V1E1	P36543	ATPase H+ transporting V1 subunit E1		yes	no	no	no	no	yes	1.95439E-15	0.550185784	1.68795E-28	1.8065E-16	0.445982043	2.64626E-29	-2.83821606	0.155207958	2.993424019
771	PGM1	P36871	phosphoglucomutase 1	Alpha-D-Glucose 1,6-Bisphosphate; Alpha-D-Glucose-1-Phosphate-6-Vanadate	yes	no	no	no	no	yes	1.55341E-15	0.516930976	4.04916E-19	1.34437E-16	0.409422449	1.41638E-19	-2.611358535	-0.171706381	2.439652153
772	GNL1	P36915	G protein nucleolar 1 (putative)		yes	no	no	no	no	yes	1.65774E-10	0.920810139	7.22064E-21	6.24189E-11	0.887015113	2.29346E-21	-2.332851451	0.033944579	2.36679603
773	SERPINF1	P36955	serpin family F member 1	Copper	no	yes	no	no	yes	no	2.82188E-14	0.369731221	1.61609E-14	4.23247E-15	0.26268172	6.91249E-15	1.550172898	0.111794634	-1.438378264

774	GMPR	P36959	guanosine monophosphate reductase	Guanosine-5'-Monophosphate	yes	no	no	no	no	yes	4.68976E-14	0.093115823	7.46277E-27	7.71355E-15	0.046410242	1.43084E-27	-2.92182882	0.472527537	3.394356357
775	GPX4	P36969	glutathione peroxidase 4	Glutathione	yes	no	no	no	no	yes	2.17785E-16	0.001388006	5.35249E-29	1.13481E-17	0.000249677	7.76075E-30	-2.215251431	0.645815572	2.861067003
776	HPCAL1	P37235	hippocalcin like 1		yes	no	no	no	no	yes	2.98276E-12	0.014759635	1.15434E-30	8.06653E-13	0.004681352	1.31805E-31	-2.882567255	0.743217881	3.625785137
777	TALDO1	P37837	transaldolase 1		yes	no	no	no	no	yes	8.49834E-15	5.99603E-05	7.97014E-23	1.03582E-15	4.53709E-06	2.21373E-23	-2.315742128	0.852505568	3.168247696
778	SNCA	P37840	synuclein alpha	Resveratrol; Copper	yes	no	no	no	no	yes	6.24611E-11	0.138906945	1.05718E-24	2.20468E-11	0.077134599	2.59146E-25	-2.178260085	0.404380359	2.582640443
779	ETFB	P38117	electron transfer flavoprotein subunit beta		yes	no	no	no	no	yes	0.000206587	0.520770475	6.98939E-06	0.000139004	0.413171319	4.6902E-06	-1.292979466	0.205672966	1.498652432
780	ATP6V1A	P38606	ATPase H+ transporting V1 subunit A	Alendronic acid; Etidronic acid; Bafilomycin A1; Bafilomycin B1	yes	no	no	no	no	yes	3.67965E-11	0.188220957	4.63763E-29	1.25045E-11	0.112318532	6.68223E-30	-2.735364251	0.417256199	3.152620451
781	COL18A1	P39060	collagen type XVIII alpha 1 chain		no	yes	no	no	yes	no	1.37917E-10	0.933005373	1.40332E-13	5.10547E-11	0.902990248	6.33305E-14	1.149195359	-0.014381935	-1.163577294
782	CCT6A	P40227	chaperonin containing TCP1 subunit 6A		yes	no	no	no	no	yes	2.55606E-15	0.291641496	7.98247E-29	2.55953E-16	0.195044335	1.18272E-29	-3.236368761	0.288197601	3.524566362
783	USP8	P40818	ubiquitin specific peptidase 8		yes	no	no	no	no	yes	1.19685E-17	0.108078912	1.30345E-19	2.76571E-19	0.055924838	4.4413E-20	-2.325619507	-0.276094339	2.049525167
784	MDH1	P40925	malate dehydrogenase 1	NADH; Nicotinamide adenine dinucleotide phosphate; Artenimol	yes	no	no	no	no	yes	3.12631E-17	0.000282855	6.77176E-31	1.09074E-18	3.31941E-05	7.39463E-32	-3.129691253	0.964656137	4.09434739
785	HADHA	P40939	hydroxyacyl-CoA dehydrogenase trifunctional multienzyme complex subunit alpha	NADH	yes	no	no	no	no	yes	1.0065E-07	0.209337098	9.62473E-07	5.13513E-08	0.128296696	6.14029E-07	-2.719890648	-0.443843995	2.276046654
786	EIF2S3	P41091	eukaryotic translation initiation factor 2 subunit gamma	Guanosine-5'-Diphosphate	yes	no	no	no	no	yes	1.43565E-12	0.478239812	1.50807E-27	3.58425E-13	0.36859353	2.63076E-28	-2.981585964	0.219057993	3.200643957
787	PTGDS	P41222	prostaglandin D2 synthase	Vitamin A	no	yes	no	no	yes	no	1.40222E-09	0.611157642	2.99858E-15	5.92147E-10	0.508974964	1.2622E-15	1.426997699	-0.101144092	-1.528141791
788	CSK	P41240	C-terminal Src kinase	Dasatinib; Staurosporine; TG-100801; Fostamatinib	yes	no	no	no	no	yes	1.17523E-14	0.008109459	3.14988E-31	1.50288E-15	0.002195733	3.22552E-32	-3.213399238	0.799719078	4.013118316
789	IARS1	P41252	isoleucyl-tRNA synthetase 1	Isoleucine	yes	no	no	no	no	yes	2.79051E-12	0.942901616	8.09044E-23	7.51049E-13	0.917384925	2.25081E-23	-2.220346212	-0.02028563	2.200060581
790	TMPO	P42166	thymopoietin		yes	no	yes	no	no	no	7.39737E-11	1.93769E-06	0.015560932	2.66131E-11	4.55902E-08	0.013029724	-1.656906473	-1.197283692	0.459622782
791	PI4KA	P42356	phosphatidylinositol 4-kinase alpha		yes	no	no	no	no	yes	2.3571E-15	0.499156235	6.20535E-18	2.27486E-16	0.390594843	2.27745E-18	-2.391355115	-0.175204756	2.216150359
792	WAS	P42768	WASP actin nucleation promoting factor	(S)-wiskostatin	yes	no	no	no	no	yes	5.2543E-13	0.982271759	2.4666E-30	1.17371E-13	0.975150623	2.95054E-31	-3.689918349	0.009099148	3.699017498
793	ECE1	P42892	endothelin converting enzyme 1	5-(2-hydroxyethyl)nonane-1,9-diol	yes	no	no	no	no	yes	1.6192E-11	0.66827171	5.73858E-26	5.19437E-12	0.571074058	1.22988E-26	-3.490029099	0.187420092	3.677449191
794	PAFAH1B1	P43034	platelet activating factor acetylhydrolase 1b regulatory subunit 1		yes	no	no	no	no	yes	8.28973E-07	0.025890088	5.1706E-22	4.57494E-07	0.009408179	1.4994E-22	-1.469529223	0.653359943	2.122889166
795	GRK6	P43250	G protein-coupled receptor kinase 6		yes	no	no	no	no	yes	1.72878E-15	0.969242054	7.23563E-27	1.54314E-16	0.952964361	1.38024E-27	-3.606236039	0.015052463	3.621288503
796	SYK	P43405	spleen associated tyrosine kinase	Staurosporine; N-(2-hydroxy-1,1-dimethylethyl)-1-methyl-3-(1H-pyrrolo[2,3-b]pyridin-2-yl)-1H-indole-5-carboxamide; Tamatinib; 2-{2-[(3,5-dimethylphenyl)amino]pyrimidin-4-yl}-N-[(1S)-2-hydroxy-1-methylethyl]-4-methyl-1,3-thiazole-5-carboxamide; 2-{[(1R,2S)-2-aminocyclohexyl]amino}-4-[(3-methylphenyl)amino]pyrimidine-5-carboxamide; Ellagic acid; Fostamatinib	yes	no	no	no	no	yes	3.65685E-14	0.633440568	7.97193E-27	5.76246E-15	0.53355959	1.54598E-27	-3.092578516	0.152633644	3.24521216
797	RANBP1	P43487	RAN binding protein 1	Copper; Phenethyl Isothiocyanate	yes	no	no	no	no	yes	1.63441E-05	0.872661978	1.56862E-12	9.94566E-06	0.818095892	7.44864E-13	-1.333303662	0.059423962	1.392727624
798	BAG6	P46379	BAG cochaperone 6		yes	no	no	no	no	yes	5.61217E-08	0.108880651	0.000698661	2.78701E-08	0.056438362	0.000531514	-1.628124147	-0.615510484	1.012613663
799	NSF	P46459	N-ethylmaleimide sensitive factor, vesicle fusing ATPase	1-Ethyl-Pyrrolidine-2,5-Dione	yes	no	no	no	no	yes	5.04005E-13	0.382350072	2.58247E-21	1.11214E-13	0.274418901	7.93347E-22	-2.242995576	0.204191227	2.447186803
800	MAP2K3	P46734	mitogen-activated protein kinase kinase 3	Fostamatinib	yes	no	no	no	no	yes	1.27282E-17	0.00022128	4.03562E-33	2.99893E-19	2.41633E-05	2.88912E-34	-2.890239799	0.866887872	3.75712767
801	RPL27A	P46776	ribosomal protein L27a		yes	no	no	no	no	yes	0.00503036	0.740680052	1.29603E-06	0.00389756	0.655772008	8.35048E-07	-1.052650037	0.13994104	1.192591076
802	RPL5	P46777	ribosomal protein L5		yes	no	no	no	no	yes	3.54448E-06	0.78960605	6.32077E-08	2.04606E-06	0.714473621	3.78616E-08	-1.481027992	-0.086436077	1.394591915
803	RPS9	P46781	ribosomal protein S9	Artenimol	yes	no	no	no	no	yes	0.000641567	0.002363313	2.29195E-11	0.000454068	0.000478659	1.14442E-11	-1.066346211	0.897805167	1.964151377
804	RPS10	P46783	ribosomal protein S10		yes	no	no	no	no	yes	9.99255E-08	0.094015295	1.17198E-06	5.09362E-08	0.047028947	7.51939E-07	-1.684475829	-0.498018983	1.186456846
805	IQGAP1	P46940	IQ motif containing GTPase activating protein 1	Artenimol	yes	no	no	no	no	yes	2.59746E-07	0.380140446	3.7591E-08	1.37464E-07	0.271971801	2.22616E-08	-1.620362892	-0.242021877	1.378341016
806	GYG1	P46976	glycogenin 1	Uridine diphosphate glucose; Uridine-5'-Diphosphate	yes	no	no	no	no	yes	5.99895E-16	0.082931896	6.03349E-20	4.1044E-17	0.040000698	2.01481E-20	-2.969359355	0.517114675	3.48647403
807	QARS1	P47897	glutaminyl-tRNA synthetase 1		yes	no	no	no	no	yes	3.5894E-18	0.000602558	8.06408E-27	4.87911E-20	8.84589E-05	1.56751E-27	-2.02296165	0.622597935	2.645559586
808	LIMS1	P48059	LIM zinc finger domain containing 1		yes	no	no	no	no	yes	5.54969E-10	0.346469737	1.20568E-36	2.24174E-10	0.241288621	3.38705E-38	-4.605141981	0.540664575	5.145806555
809	PIP4K2A	P48426	phosphatidylinositol-5-phosphate 4-kinase type 2 alpha		yes	no	no	no	no	yes	6.14942E-11	0.262884486	9.51129E-31	2.16776E-11	0.171404973	1.07309E-31	-3.863150681	-0.484246709	3.378903972
810	ARCN1	P48444	archain 1		yes	no	no	no	no	yes	1.6822E-13	0.061774359	1.12586E-33	3.23941E-14	0.027906224	6.93777E-35	-2.994760133	0.517425786	3.512185919
811	GCLM	P48507	glutamate-cysteine ligase modifier subunit	Glutamic acid; Cysteine	yes	no	no	no	no	yes	2.52524E-15	0.04650424	1.77185E-27	2.49435E-16	0.019933399	3.15513E-28	-2.726387925	0.49636756	3.222755486
812	CD151	P48509	CD151 molecule (Raph blood group)		yes	no	no	no	no	yes	1.71492E-09	0.019236899	2.87861E-30	7.28861E-10	0.006519801	3.4825E-31	-3.169731688	1.025849227	4.195580915
813	CCT5	P48643	chaperonin containing TCP1 subunit 5		yes	no	no	no	no	yes	1.18548E-14	0.063105045	2.57432E-31	1.5255E-15	0.028593133	2.58948E-32	-3.35919432	0.558609059	3.917803379
814	KRT6C	P48668	keratin 6C		no	yes	no	yes	no	no	0.000400201	0.004532512	0.941766114	0.00027762	0.001067923	0.938352372	1.306228162	1.281847698	-0.024380464
815	IDH2	P48735	isocitrate dehydrogenase (NADP(+)) 2	Isocitric Acid; Enasidenib	yes	no	no	no	no	yes	9.43971E-11	0.001470483	8.72454E-16	3.43884E-11	0.000267845	3.56571E-16	-2.859130994	0.965088834	3.824219829
816	MARCKSL1	P49006	MARCKS like 1		no	yes	no	no	yes	no	3.33492E-06	0.825587902	8.40197E-10	1.91905E-06	0.759750353	4.50745E-10	1.069273746	-0.058741252	-1.128014998
817	ALDH9A1	P49189	aldehyde dehydrogenase 9 family member A1	NADH	yes	no	no	no	no	yes	4.83726E-11	0.55088657	3.07574E-20	1.67452E-11	0.44679971	1.01456E-20	-2.132638036	0.167944111	2.300582147
818	LMAN1	P49257	lectin, mannose binding 1	Antihemophilic factor, human recombinant; Lonoctocog alfa; Moroctocog alfa	yes	no	no	no	no	yes	1.58262E-05	0.0094345	7.88265E-08	9.609E-06	0.002654656	4.75388E-08	-1.680498767	0.539030194	2.219528961
819	SLC11A2	P49281	solute carrier family 11 member 2	Ferric maltol	yes	no	no	no	no	yes	9.30986E-12	0.563336554	2.36982E-21	2.81879E-12	0.458428841	7.25872E-22	-2.508214909	-0.1768671	2.331347809
820	FASN	P49327	fatty acid synthase	Cerulenin; Orlistat	yes	no	no	no	no	yes	0.000183815	0.09911592	1.55253E-21	0.000123265	0.050343881	4.69207E-22	-1.767394618	0.810770412	2.57816503
821	CCT3	P49368	chaperonin containing TCP1 subunit 3	Phosphoaminophosphonic Acid-Adenylate Ester; Artenimol	yes	no	no	no	no	yes	4.39534E-14	0.007540619	5.85982E-32	7.06999E-15	0.001950926	5.12436E-33	-2.830642228	0.732864042	3.563506269
822	ARRB1	P49407	arrestin beta 1		yes	no	no	no	no	yes	5.88893E-12	0.03442498	1.14054E-29	1.69704E-12	0.013414356	1.50548E-30	-2.684741598	0.610026935	3.294768534
823	AARS1	P49588	alanyl-tRNA synthetase 1	Alanine	yes	no	no	no	no	yes	1.30815E-11	0.364978181	8.71105E-23	4.10486E-12	0.257838702	2.43136E-23	-1.734960479	0.193398978	1.928359457
824	CARS1	P49589	cysteinyl-tRNA synthetase 1	Cysteine	yes	no	no	no	no	yes	6.7456E-08	0.788473308	1.36197E-15	3.39572E-08	0.713091401	5.60958E-16	-1.456246868	-0.073413522	1.382833346
825	HARS2	P49590	histidyl-tRNA synthetase 2, mitochondrial		yes	no	no	no	no	yes	5.85957E-12	0.990010376	6.28712E-33	1.68592E-12	0.989113221	4.61492E-34	-4.643723841	-0.005241085	4.638482756
826	SARS1	P49591	seryl-tRNA synthetase 1	Serine	yes	no	no	no	no	yes	4.60221E-13	0.037225547	1.67922E-25	1.00093E-13	0.014944193	3.76626E-26	-2.373947304	0.544853306	2.91880061
827	DGKG	P49619	diacylglycerol kinase gamma	Phosphatidyl serine	yes	no	no	no	no	yes	2.0751E-20	0.082931896	5.28955E-06	3.76095E-23	0.040019243	3.52557E-06	-2.403381932	-0.673460171	1.729921761
828	NUMB	P49757	NUMB endocytic adaptor protein		no	no	no	yes	no	yes	0.046399842	0.000264757	1.57238E-13	0.03918863	3.03505E-05	7.11026E-14	-0.638482105	1.588949475	2.227431579
829	EIF2B2	P49770	eukaryotic translation initiation factor 2B subunit beta		yes	no	no	no	no	yes	8.58808E-12	0.162630223	1.7836E-27	2.57603E-12	0.093657913	3.18413E-28	-3.068964927	0.468469786	3.537434713
830	HINT1	P49773	histidine triad nucleotide binding protein 1	Adenosine phosphate; Guanosine-5'-Monophosphate; 5'-O-(N-Ethyl-Sulfamoyl)Adenosine; Adenosine-5'-ditungstate; 8-Bromo-Adenosine-5'-	yes	no	no	no	no	yes	1.58472E-15	0.658431331	1.84051E-13	1.38456E-16	0.559905433	8.33109E-14	-2.146606331	0.140086381	2.286692712
831	KLK7	P49862	kallikrein related peptidase 7	L-alanyl-N-[(1S,2R)-1-benzyl-2-hydroxypropyl]-L-alaninamide	no	yes	no	no	yes	no	1.73647E-08	0.287742164	4.64647E-14	8.22996E-09	0.191522673	2.03796E-14	1.431584367	-0.257364696	-1.688949063
832	MRE11	P49959	MRE11 homolog, double strand break repair nuclease		yes	no	no	no	no	yes	6.99154E-06	0.159291752	2.14406E-16	4.12776E-06	0.091085723	8.47132E-17	-1.272055944	0.376836419	1.648892362
833	SULT1A1	P50225	sulfotransferase family 1A member 1	Adenosine 3',5'-diphosphate; P-Nitrophenol	yes	no	no	no	no	yes	9.38905E-18	0.002805605	2.47181E-31	1.95694E-19	0.000583495	2.44157E-32	-4.142935555	0.89646067	5.039396226
834	MMP14	P50281	matrix metallopeptidase 14	Marimastat	no	yes	no	yes	no	no	4.89056E-09	2.74954E-05	0.004897137	2.19377E-09	1.54704E-06	0.003947443	1.830709327	1.30247457	-0.528234757
835	GDI2	P50395	GDP dissociation inhibitor 2		yes	no	no	no	no	yes	1.05666E-12	0.546214529	1.38353E-29	2.59497E-13	0.441243733	1.84688E-30	-2.872760889	0.18234532	3.055106209
836	SERPINH1	P50454	serpin family H member 1		yes	no	no	no	no	yes	5.07681E-11	0.283097032	2.03646E-17	1.76205E-11	0.187781202	7.65279E-18	-2.250351439	0.341341346	2.591692785
837	DNM2	P50570	dynamin 2		yes	no	no	no	no	yes	1.79935E-15	0.014618859	7.80195E-33	1.62243E-16	0.004628938	5.9036E-34	-2.686853711	0.562999578	3.249853289

838	METAP2	P50579	methionyl aminopeptidase 2	Methionine; Nitroxoline; 3,5,6,8-Tetramethyl-N-Methyl Phenanthrolinium; Fumagillin; D-Methionine; N'-(2s,3r)-3-Amino-4-Cyclohexyl-2-Hydroxy-Butano-N-(4-Methylphenyl)Hydrazide; (2R,3R,4S,5R,6E)-3,4,5-Trihydroxy-N-[(3S,6R)-6-hydroxy-2-oxo-3-azepanyl]-2-methoxy-8,8-dimethyl-6-nonenamide; tert-butanol; (2s,3r)-3-Amino-2-Hydroxy-5-(Ethylsulfanyl)Pentanoyl-((S)-(-)-(1-Naphthyl)Ethyl)Amide; Ovalicin; PPI-2458; 5-BROMO-2-{[(4-CHLOROPHENYL)SULFONYL]AMINO} BENZOIC ACID; 5-METHYL-2-[(PHENYLSULFONYL)AMINO]BENZOIC ACID; 2-[(PHENYLSULFONYL)AMINO]-5,6,7,8-TETRAHYDRONAPHTHALENE-1-CARBOXYLIC ACID; 2-[({2-[(1Z)-3-(DIMETHYLAMINO)PROP-1-ENYL]-4-FLUOROPHENYL}SULFONYL)AMINO]-5,6,7,8-TETRAHYDRONAPHTHALENE-1-CARBOXYLIC ACID; N'-((2S,3R)-3-AMINO-2-HYDROXY-5-(ISOPROPYLSULFANYL)PENTANOYL)-N-3-CHLOROBENZOYL HYDRAZIDE; 3-	yes	no	no	no	no	yes	4.48009E-10	0.721932442	4.10646E-20	1.79447E-10	0.632631329	1.362E-20	-2.12016728	0.110349947	2.230517228
839	CCT8	P50990	chaperonin containing TCP1 subunit 8		yes	no	no	no	no	yes	4.23352E-15	0.00164575	1.57658E-31	4.58455E-16	0.000304244	1.52157E-32	-3.589710117	0.994688714	4.584398831
840	CCT4	P50991	chaperonin containing TCP1 subunit 4		yes	no	no	no	no	yes	2.58447E-16	0.107007765	6.10551E-31	1.40524E-17	0.05522204	6.55643E-32	-3.499808052	0.450414959	3.950223011
841	ANXA11	P50995	annexin A11		yes	no	no	no	no	yes	4.50578E-16	0.816799323	1.04772E-23	2.89905E-17	0.748701871	2.75342E-24	-3.459059552	-0.082207754	3.376851798
842	RAB5C	P51148	RAB5C, member RAS oncogene family		yes	no	no	no	no	yes	2.24353E-11	0.759851236	2.64597E-20	7.27852E-12	0.677785	8.69202E-21	-2.539862802	0.102531654	2.642394456
843	RAB7A	P51149	RAB7A, member RAS oncogene family	Guanosine-5'-Diphosphate	yes	no	no	no	no	yes	6.75898E-11	0.113808906	6.30772E-32	2.40714E-11	0.060127406	5.60179E-33	-2.799573344	0.512704178	3.312277522
844	RAB13	P51153	RAB13, member RAS oncogene family		yes	no	no	no	no	yes	6.945E-20	0.000506314	1.99038E-29	2.51744E-22	7.14864E-05	2.72359E-30	-3.103330029	0.759685484	3.863015513
845	MMP15	P51511	matrix metallopeptidase 15	Marimastat	no	yes	no	no	yes	no	2.2645E-15	2.96829E-05	1.50829E-08	2.13853E-16	1.72325E-06	8.74084E-09	1.841903507	0.777519077	-1.06438443
846	P2RX1	P51575	purinergic receptor P2X 1		yes	no	no	no	no	yes	5.7235E-10	0.043383081	1.00293E-12	2.31585E-10	0.018241731	4.71701E-13	-1.570435408	0.469253133	2.039688541
847	PSMD7	P51665	proteasome 26S subunit, non-ATPase 7		yes	no	no	no	no	yes	1.28809E-16	0.0094345	1.59313E-28	5.83639E-18	0.002652462	2.4543E-29	-1.972512101	0.451847124	2.424359224
848	RPS6KA3	P51812	ribosomal protein S6 kinase A3	Acetylsalicylic acid; Fostamatinib	yes	no	no	no	no	yes	1.24197E-13	0.93115471	4.4961E-22	2.30724E-14	0.900355302	1.29566E-22	-3.089971583	-0.032481948	3.057489635
849	HNRNPA3	P51991	heterogeneous nuclear ribonucleoprotein A3		yes	no	yes	no	no	no	6.45338E-11	9.20893E-06	0.00073119	2.28542E-11	3.54671E-07	0.000556923	-2.031352895	-1.317712376	0.713640518
850	ARHGDIA	P52565	Rho GDP dissociation inhibitor alpha		yes	no	no	no	no	yes	2.5177E-15	0.90145161	5.2428E-30	2.47549E-16	0.861423401	6.55647E-31	-2.563761451	0.03088035	2.594641802
851	ARHGDIB	P52566	Rho GDP dissociation inhibitor beta		yes	no	no	no	no	yes	6.9674E-13	0.328029638	1.91679E-23	1.6201E-13	0.225430069	5.1068E-24	-2.559403221	0.289503069	2.84890629
852	BLVRA	P53004	biliverdin reductase A	NADH	yes	no	no	no	no	yes	1.01624E-17	9.81042E-05	6.68571E-36	2.21023E-19	8.18323E-06	2.02964E-37	-2.535511135	0.770912265	3.3064234
853	COPB1	P53618	COPI coat complex subunit beta 1		yes	no	no	no	no	yes	9.81156E-15	0.179983841	1.11817E-09	1.22255E-15	0.106098313	6.03415E-10	-2.323086278	-0.426914706	1.896171572
854	COPA	P53621	COPI coat complex subunit alpha		yes	no	no	no	no	yes	8.33096E-11	0.016484954	2.91245E-25	3.02361E-11	0.005415311	6.73018E-26	-2.918594125	0.926128358	3.844722483
855	AP2S1	P53680	adaptor related protein complex 2 subunit sigma 1		yes	no	no	no	no	yes	8.2492E-13	0.000409318	5.18359E-33	1.96605E-13	5.13734E-05	3.73444E-34	-2.178656639	0.854202522	3.032859162
856	IST1	P53990	IST1 factor associated with ESCRT-III		yes	no	no	no	no	yes	5.94109E-18	0.646669926	3.16693E-25	9.13568E-20	0.548219045	7.34694E-26	-2.467896994	-0.096456955	2.37144004
857	SUB1	P53999	SUB1 regulator of transcription		yes	no	yes	no	no	no	0.000924255	6.02029E-07	0.466833105	0.000666284	9.27457E-09	0.444411578	-1.281743378	-1.535621981	-0.253878603
858	RARS1	P54136	arginyl-tRNA synthetase 1		yes	no	no	no	no	yes	2.02198E-10	0.021325164	3.3983E-30	7.75076E-11	0.007414402	4.18821E-31	-2.208721049	0.649689293	2.858410341
859	YARS1	P54577	tyrosyl-tRNA synthetase 1	Tyrosine; Beta-(2-Naphthyl)-Alanine; Resveratrol; Tyrosinal; N6-ISOPENTENYL-ADENOSINE-5'-MONOPHOSPHATE; p-Benzoyl-L-phenylalanine; 4-(2,2,2-TRIFLUOROETHYL)-L-	yes	no	no	no	no	yes	4.33336E-10	0.2342269	8.68857E-32	1.73374E-10	0.14751611	8.1886E-33	-2.703664886	0.407381545	3.111046431
860	HSPA2	P54652	heat shock protein family A (Hsp70) member 2	Quercetin	yes	no	no	no	no	yes	1.27731E-14	0.322872252	4.54286E-25	1.67605E-15	0.220612304	1.07565E-25	-2.412806011	0.212316763	2.625122774
861	ATP1B3	P54709	ATPase Na+/K+ transporting subunit beta 3		yes	no	no	no	no	yes	5.48495E-09	0.151778514	5.26945E-14	2.46786E-09	0.085895498	2.32314E-14	-1.815764693	0.370633144	2.186397837
862	FXYD2	P54710	FXYD domain containing ion transport regulator 2	Cyclothiazide; Thimerosal	no	yes	no	no	yes	no	0.000205111	0.091379405	1.00023E-08	0.000137918	0.045170054	5.70588E-09	1.007325232	-0.430460136	-1.437785368
863	NAPA	P54920	NSF attachment protein alpha		yes	no	no	no	no	yes	3.40579E-14	0.000675018	3.87401E-21	5.2468E-15	0.000104703	1.20591E-21	-2.83971712	-0.914601708	1.925115412
864	EIF5	P55010	eukaryotic translation initiation factor 5		yes	no	no	no	no	yes	1.43655E-10	0.669391438	3.04244E-23	5.34394E-11	0.572334229	8.24367E-24	-2.542268426	-0.145944635	2.396323791
865	PSMD4	P55036	proteasome 26S subunit ubiquitin receptor, non-ATPase		yes	no	no	no	no	yes	2.97212E-13	0.526400815	8.74862E-20	6.11392E-14	0.419784973	2.9532E-20	-2.941099148	0.219018768	3.160117916
866	DRG2	P55039	developmentally regulated GTP binding protein 2		yes	no	no	no	no	yes	1.26432E-11	0.081503631	1.64233E-27	3.94134E-12	0.039071519	2.88544E-28	-2.973791381	0.59968534	3.573476722
867	APOC4	P55056	apolipoprotein C4		no	yes	no	no	yes	no	3.53815E-07	0.815357003	2.31457E-08	1.89492E-07	0.746271476	1.35602E-08	1.075211963	-0.052470641	-1.127682604
868	CSE1L	P55060	chromosome segregation 1 like		yes	no	no	no	no	yes	4.14518E-14	0.81390777	5.12134E-22	6.64881E-15	0.744207467	1.48048E-22	-3.232726882	0.0866698	3.319396682
869	HADHB	P55084	hydroxyacyl-CoA dehydrogenase trifunctional multienzyme complex subunit beta		yes	no	no	no	no	yes	2.43592E-06	0.010716847	8.7314E-09	1.38407E-06	0.00310774	4.94925E-09	-2.302529047	0.725994872	3.028523919
870	MANF	P55145	mesencephalic astrocyte derived neurotrophic factor		yes	no	no	no	no	yes	9.9481E-11	0.011962939	7.0912E-33	3.62855E-11	0.00359376	5.2694E-34	-2.60310356	0.793255586	3.396359146
871	MTTP	P55157	microsomal triglyceride transfer protein	Hesperetin; Implitapide; SLx-4090;	no	yes	no	no	yes	no	2.92423E-09	0.576109997	5.51753E-09	1.27993E-09	0.472217029	3.09252E-09	1.305690928	0.122562876	-1.183128052
872	NCKAP1L	P55160	NCK associated protein 1 like		yes	no	no	no	no	yes	1.44486E-16	0.001993778	1.66223E-33	6.80859E-18	0.000385747	1.06949E-34	-3.575839245	0.852311729	4.428150974
873	CCL18	P55774	C-C motif chemokine ligand 18		no	yes	no	yes	no	no	3.46261E-11	1.03594E-05	0.004480494	1.16728E-11	4.27141E-07	0.003597388	2.134742029	1.37750979	-0.757232239
874	NPEPPS	P55786	aminopeptidase puromycin sensitive	Artenimol; Tosedostat	yes	no	no	no	no	yes	1.84613E-17	0.700427087	1.85725E-22	5.30573E-19	0.605851975	5.26796E-23	-2.703868961	0.103706772	2.807575733
875	SUMO3	P55854	small ubiquitin like modifier 3		yes	no	no	no	no	yes	2.38684E-11	0.659815867	1.031E-25	7.80834E-12	0.561739218	2.2797E-26	-2.557367352	-0.138878484	2.418488867
876	EIF3B	P55884	eukaryotic translation initiation factor 3 subunit B		yes	no	no	no	no	yes	4.11457E-15	0.986791254	4.85943E-27	4.4371E-16	0.982320065	9.07151E-28	-3.145300911	0.004667665	3.149968576
877	EEFSEC	P57772	eukaryotic elongation factor, selenocysteine-tRNA		yes	no	no	no	no	yes	5.08348E-11	0.389609245	2.72332E-21	1.76667E-11	0.28227693	8.39082E-22	-2.174516771	0.253479688	2.427996459
878	MTPN	P58546	myotrophin		yes	no	no	no	no	yes	3.80269E-14	0.026037484	4.50523E-35	6.01332E-15	0.009520729	1.8372E-36	-3.70027875	0.731827687	4.432106436
879	TPI1	P60174	triosephosphate isomerase 1	Zinc; N-Hydroxy-4-Phosphono-Butanamide; 2-Phosphoglycolic Acid; Phosphoglycolohydroxamic Acid; 3-(2-Benzothiazolylthio)-1-Propanesulfonic Acid; [2(Formyl-Hydroxy-Amino)-Ethyl]-Phosphonic Acid; 5-fluorotryptophan; 2-Carboxyethylphosphonic Acid; tert-butanol; Dihydroxyacetone phosphate; 1,4-Dithiothreitol; 3-phospho-D-glyceric acid; 3-(BUTYLSULPHONYL)-PROPANOIC ACID; Artenimol; Zinc acetate; Zinc	yes	no	no	no	no	yes	2.06854E-12	0.02098603	2.17188E-31	5.37053E-13	0.007217216	2.13547E-32	-3.720503235	0.884911265	4.6054145
880	EIF3E	P60228	eukaryotic translation initiation factor 3 subunit E		yes	no	no	no	no	yes	6.08987E-16	0.942901616	2.12273E-27	4.2218E-17	0.917694912	3.79918E-28	-3.339373457	-0.022314172	3.317059285
881	MYL6	P60660	myosin light chain 6		yes	no	no	no	no	yes	4.88365E-12	0.243858807	1.15592E-19	1.38743E-12	0.154911666	3.92289E-20	-3.480648616	-0.451082281	3.029566335
882	EIF4A1	P60842	eukaryotic translation initiation factor 4A1	Copper	yes	no	no	no	no	yes	1.44486E-16	0.007593368	1.79522E-33	6.78817E-18	0.001978335	1.16319E-34	-3.131544863	0.630031553	3.761576416
883	PRPS1	P60891	phosphoribosyl pyrophosphate synthetase 1		yes	no	no	no	no	yes	1.71669E-10	0.925139475	5.56441E-18	6.47939E-11	0.892023925	2.03717E-18	-1.828406628	-0.027686943	1.800719685
884	CDC42	P60953	cell division cycle 42	Aminophosphonic acid-guanylate ester; Guanosine-5'-Diphosphate	yes	no	no	no	no	yes	9.46566E-13	0.186730409	3.29802E-35	2.26884E-13	0.111090633	1.25525E-36	-3.982774537	0.509073704	4.491848241
885	DSTN	P60981	destrin, actin depolymerizing factor	Dodecyldimethylamine N-oxide	yes	no	no	no	no	yes	2.65721E-11	0.030340796	3.79917E-35	8.76505E-12	0.011451692	1.48042E-36	-3.422190552	0.810051752	4.232242304
886	KRTDAP	P60985	keratinocyte differentiation associated protein		no	no	yes	no	yes	no	0.129832221	2.07469E-05	2.57603E-15	0.113889977	9.96455E-07	1.0799E-15	0.338737153	-1.180492493	-1.519229646
887	RAB8A	P61006	RAB8A, member RAS oncogene family		yes	no	no	no	no	yes	7.32529E-14	0.017300974	4.87962E-37	1.2679E-14	0.00572257	1.09619E-38	-3.815608866	0.810949281	4.626558147
888	RAB2A	P61019	RAB2A, member RAS oncogene family		yes	no	no	no	no	yes	1.86399E-13	0.00867067	1.5409E-24	3.6317E-14	0.0023808	3.81909E-25	-2.620218763	0.684925593	3.305144355
889	RAB5B	P61020	RAB5B, member RAS oncogene family		yes	no	no	no	no	yes	1.77012E-12	0.010096503	9.21677E-14	4.52354E-13	0.002882101	4.10933E-14	-2.588625639	-0.766998762	1.821626876
890	RAB10	P61026	RAB10, member RAS oncogene family		yes	no	no	no	no	yes	1.59323E-10	0.139773917	3.07604E-28	5.97733E-11	0.077835135	4.97574E-29	-3.264710251	0.595372957	3.860083208
891	UBE2D3	P61077	ubiquitin conjugating enzyme E2 D3		yes	no	no	no	no	yes	7.2761E-13	0.661808122	6.89793E-32	1.70568E-13	0.564351103	6.28221E-33	-2.724700605	0.125024976	2.849725581
892	UBE2M	P61081	ubiquitin conjugating enzyme E2 M		yes	no	no	no	no	yes	2.48491E-12	0.037025362	8.24027E-32	6.57539E-13	0.014830277	7.69142E-33	-2.468241695	0.545088674	3.013330369
893	UBE2N	P61088	ubiquitin conjugating enzyme E2 N		yes	no	no	no	no	yes	1.49311E-14	0.762464994	9.3204E-29	2.00931E-15	0.68135124	1.39363E-29	-2.425993205	-0.071863808	2.354129397
894	RAB14	P61106	RAB14, member RAS oncogene family		yes	no	no	no	no	yes	5.42738E-12	0.694324105	2.26009E-28	1.54928E-12	0.599314644	3.58419E-29	-3.349593066	-0.148645599	3.200947466
895	COPS2	P61201	COP9 signalosome subunit 2		yes	no	no	no	no	yes	5.9314E-16	0.498479809	1.41268E-27	4.03131E-17	0.389387943	2.45795E-28	-2.446604352	0.147541315	2.594145667

896	ARF3	P61204	ADP ribosylation factor 3		yes	no	no	no	no	yes	5.49958E-13	0.577649007	1.57347E-31	1.24302E-13	0.474263707	1.51145E-32	-3.658646735	0.205523592	3.864170327
897	ABCE1	P61221	ATP binding cassette subfamily E member 1		yes	no	no	no	no	yes	9.72415E-15	0.024356357	1.48873E-32	1.20726E-15	0.008740478	1.22093E-33	-2.852675421	0.562585263	3.415260683
898	RAP1B	P61224	RAP1B, member of RAS oncogene family		yes	no	no	no	no	yes	1.99067E-10	0.279487948	9.16995E-36	7.6127E-11	0.184613039	3.04147E-37	-4.152453698	0.531906823	4.684360521
899	RAP2B	P61225	RAP2B, member of RAS oncogene family		yes	no	no	no	no	yes	4.25585E-10	0.754878544	6.69831E-32	1.7008E-10	0.672105273	6.00936E-33	-3.637258289	-0.151621223	3.485637066
900	RPS3A	P61247	ribosomal protein S3A		yes	no	no	no	no	yes	1.9119E-06	0.692108954	7.66016E-08	1.08026E-06	0.597089012	4.60928E-08	-1.175683603	0.106405539	1.282089142
901	RPL26	P61254	ribosomal protein L26		yes	no	no	no	no	yes	2.21443E-05	0.612826263	2.2698E-08	1.36659E-05	0.511076119	1.32877E-08	-1.225793186	0.132824914	1.3586181
902	ATP6V0D1	P61421	ATPase H+ transporting V0 subunit d1		yes	no	yes	no	no	no	1.10779E-07	1.06581E-08	0.768259458	5.67701E-08	8.20971E-11	0.752246801	-1.876493874	-1.793693548	0.082800327
903	RHOA	P61586	ras homolog family member A	Guanosine-5'-Diphosphate	yes	no	no	no	no	yes	1.38827E-16	0.562888431	1.09878E-31	6.4161E-18	0.457809123	1.04053E-32	-3.687080926	0.173571326	3.860652252
904	UFM1	P61960	ubiquitin fold modifier 1		yes	no	no	no	no	yes	4.53644E-14	0.016968456	1.57238E-28	7.35861E-15	0.00558183	2.41522E-29	-2.843841037	0.613709976	3.457551013
905	HNRNPK	P61978	heterogeneous nuclear ribonucleoprotein K	Artenimol; Phenethyl Isothiocyanate	yes	no	no	no	no	yes	2.05422E-13	0.133738118	2.30144E-23	4.05817E-14	0.073625652	6.21504E-24	-2.697387853	-0.398125316	2.299262537
906	RPS7	P62081	ribosomal protein S7		yes	no	no	no	no	yes	2.80676E-08	0.00520097	1.07214E-05	1.35823E-08	0.001258413	7.31114E-06	-1.935357206	-0.865904166	1.06945304
907	PPP1CA	P62136	protein phosphatase 1 catalytic subunit alpha	2,6,8-Trimethyl-3-Amino-9-Benzyl-9-Methoxynonanoic Acid	yes	no	no	no	no	yes	4.39067E-16	0.000305015	2.3223E-33	2.74541E-17	3.60711E-05	1.5871E-34	-2.929536791	0.929550666	3.859087457
908	PPP1CB	P62140	protein phosphatase 1 catalytic subunit beta		yes	no	no	no	no	yes	9.16976E-15	0.062489627	7.5865E-23	1.12181E-15	0.028285971	2.10373E-23	-2.370209497	0.452345429	2.822554926
909	PSMC1	P62191	proteasome 26S subunit, ATPase 1		yes	no	no	no	no	yes	5.37761E-12	0.014908399	5.36291E-27	1.53263E-12	0.004755556	1.00357E-27	-1.848187195	0.505170324	2.35335752
910	RPS8	P62241	ribosomal protein S8	Artenimol	yes	no	no	no	no	yes	7.36686E-07	0.499086809	1.66181E-10	4.04858E-07	0.390314378	8.63657E-11	-1.868302413	-0.247060997	1.621241416
911	RPS16	P62249	ribosomal protein S16		yes	no	no	no	no	yes	0.002241692	0.016223655	7.70863E-13	0.001678983	0.005271917	3.59061E-13	-1.150648012	0.956095182	2.106743195
912	UBE2H	P62256	ubiquitin conjugating enzyme E2 H		yes	no	no	no	no	yes	2.14849E-14	0.378461711	2.23132E-26	3.07623E-15	0.270599266	4.52936E-27	-3.828171888	0.31069169	4.138863578
913	YWHAE	P62258	tyrosine 3-monooxygenase/tryptophan 5-monooxygenase activation protein epsilon	Fusicoccin; Phenethyl Isothiocyanate	yes	no	no	no	no	yes	4.60436E-11	0.01078664	4.99587E-38	1.59181E-11	0.003132866	6.79095E-40	-4.308461069	1.252517642	5.56097871
914	RPS13	P62277	ribosomal protein S13	Artenimol	yes	no	no	no	no	yes	7.70069E-07	0.965260223	2.54828E-08	4.23939E-07	0.94652011	1.49872E-08	-1.404532798	0.014597383	1.419130181
915	SNRPE	P62304	small nuclear ribonucleoprotein polypeptide E		yes	no	yes	no	no	no	1.27828E-07	6.75727E-05	0.034468299	6.59123E-08	5.2662E-06	0.029548718	-2.200488381	-1.525533809	0.674954572
916	LSM3	P62310	LSM3 homolog, U6 small nuclear RNA and mRNA degradation associated		yes	no	no	no	no	yes	7.08505E-10	0.900212104	6.60224E-20	2.8796E-10	0.857492455	2.21072E-20	-1.753761984	-0.03590712	1.717854864
917	LSM6	P62312	LSM6 homolog, U6 small nuclear RNA and mRNA degradation associated	Uridine; Uridine monophosphate; Citric acid	yes	no	no	no	no	yes	1.05336E-12	0.218021034	1.49572E-24	2.57732E-13	0.134546737	3.70034E-25	-2.116309741	0.294014023	2.410323763
918	SNRPD3	P62318	small nuclear ribonucleoprotein D3 polypeptide		yes	no	yes	no	no	no	0.005116209	0.000124176	0.670862552	0.003973349	1.15343E-05	0.653536243	-1.069724833	-1.215876981	-0.146152148
919	TMSB4X	P62328	thymosin beta 4 X-linked		yes	no	no	no	no	yes	2.51051E-15	0.000640648	1.92566E-25	2.45705E-16	9.69186E-05	4.34517E-26	-3.387938473	-0.969224743	2.41871373
920	ARF6	P62330	ADP ribosylation factor 6	5'-Guanosine-Diphosphate-Monothiophosphate; Guanosine-5'-	yes	no	no	no	no	yes	1.66251E-16	0.187954417	2.52655E-27	8.13554E-18	0.112074315	4.59061E-28	-3.322541249	0.348138264	3.670679514
921	PSMC6	P62333	proteasome 26S subunit, ATPase 6		yes	no	no	no	no	yes	2.42483E-12	0.031110764	1.80948E-15	6.40541E-13	0.011855076	7.50191E-16	-1.717817728	0.578490376	2.296308104
922	RAB11A	P62491	RAB11A, member RAS oncogene family	5'-Guanosine-Diphosphate-Monothiophosphate; Guanosine-5'-	yes	no	no	no	no	yes	5.12894E-11	0.36871788	2.41027E-24	1.78479E-11	0.261352733	6.08301E-25	-3.077167622	0.333295069	3.410462691
923	ETF1	P62495	eukaryotic translation termination factor 1		yes	no	no	no	no	yes	2.32399E-14	0.005040574	2.68493E-21	3.36963E-15	0.001214839	8.2604E-22	-2.555149517	0.854965318	3.410114835
924	RPS4X	P62701	ribosomal protein S4 X-linked		yes	no	no	no	no	yes	1.30645E-07	0.669928619	1.14853E-11	6.74238E-08	0.573097069	5.64636E-12	-1.489096614	0.111303408	1.600400022
925	RPL23A	P62750	ribosomal protein L23a	(S)-3-phenyllactic acid; Anisomycin; Puromycin; Artenimol	yes	no	yes	no	no	no	1.43432E-08	0.000374728	0.000154743	6.73293E-09	4.65227E-05	0.000113866	-2.156913505	-1.25527032	0.901643185
926	RAB1A	P62820	RAB1A, member RAS oncogene family		yes	no	no	no	no	yes	3.06828E-13	0.026644339	2.25186E-30	6.36733E-14	0.009812747	2.65284E-31	-3.232636249	0.71964503	3.952281279
927	RAN	P62826	RAN, member RAS oncogene family	Guanosine-5'-Diphosphate	yes	no	no	no	no	yes	4.16244E-11	0.161406158	2.63287E-22	1.43526E-11	0.092565483	7.49182E-23	-2.433558102	0.422830021	2.856388123
928	RAP1A	P62834	RAP1A, member of RAS oncogene family		yes	no	no	no	no	yes	1.92867E-18	0.113474269	4.95203E-25	2.27211E-20	0.059693533	1.18023E-25	-3.172986756	0.418399528	3.591386284
929	RPS24	P62847	ribosomal protein S24		yes	no	no	no	no	yes	3.27652E-08	0.389609245	2.87348E-10	1.59744E-08	0.282143269	1.5103E-10	-2.053319722	-0.289676275	1.763643447
930	RPS25	P62851	ribosomal protein S25		yes	no	no	no	no	yes	7.59164E-08	0.033482016	5.92569E-06	3.83538E-08	0.012986228	3.9603E-06	-1.719917821	-0.610480393	1.109437429
931	GNB1	P62873	G protein subunit beta 1		yes	no	no	no	no	yes	7.71033E-10	0.717003634	5.09904E-28	3.1582E-10	0.625496256	8.43294E-29	-3.574266432	0.184045876	3.758312308
932	GNB2	P62879	G protein subunit beta 2		yes	no	no	no	no	yes	5.17493E-14	0.049255176	7.89547E-17	8.6288E-15	0.021447315	3.06947E-17	-2.430616089	0.588806939	3.019423027
933	RPL31	P62899	ribosomal protein L31		yes	no	no	no	no	yes	0.000203247	0.163911787	1.41315E-08	0.000136573	0.094544497	8.16389E-09	-1.251383345	0.440499095	1.69188244
934	PPIA	P62937	peptidylprolyl isomerase A	Cyclosporine; Proline; (3r)-1-Acetyl-3-Methylpiperidine; Ethyl Oxo(Piperidin-1-Yl)Acetate; Sanglifehrin A; Copper;	yes	no	no	no	no	yes	1.56489E-12	0.110933595	7.637E-32	3.94945E-13	0.057954887	6.98991E-33	-3.654892388	0.574127522	4.229019909
935	FKBP1A	P62942	FKBP prolyl isomerase 1A	Pimecrolimus; Tacrolimus; Sirolimus; (3R)-4-(p-toluenesulfonyl)-1,4-thiazane-3-carboxylicacid-L-phenylalanine ethyl ester; {3-[3-(3,4-Dimethoxy-Phenyl)-1-(1-{1-[2-(3,4,5-Trimethoxy-Phenyl)-Butyryl]-Piperidin-2yl}-Vinyloxy)-Propyl]-Phenoxy}-Acetic Acid; Gpi-1046; Methyl Methylsulfinylmethyl Sulfide; FKB-001; Heptyl glucoside; L-709,587; (3r)-4-(P-Toluenesulfonyl)-1,4-Thiazane-3-Carboxylicacid-L-Leucine; 4-hydroxybutan-2-one; GPI-1485; Myristic acid; (21S)-1AZA-4,4-DIMETHYL-6,19-DIOXA-	yes	no	no	no	no	yes	1.72878E-15	0.715099703	3.47045E-23	1.5411E-16	0.622431594	9.41912E-24	-3.494149817	-0.127604639	3.366545177
936	GRB2	P62993	growth factor receptor bound protein 2	Pegademase; 4-[(10s,14s,18s)-18-(2-Amino-2-Oxoethyl)-14-(1-Naphthylmethyl)-8,17,20-Trioxo-7,16,19-Triazaspiro[5.14]Icos-11-En-10-	yes	no	no	no	no	yes	5.94109E-18	0.000214316	7.39149E-31	9.15256E-20	2.29174E-05	8.13835E-32	-2.93280483	0.808763808	3.741568638
937	RAC1	P63000	Rac family small GTPase 1	Azathioprine; Guanosine-5'-Diphosphate	yes	no	no	no	no	yes	1.03263E-13	0.141309514	4.53833E-34	1.85752E-14	0.078754283	2.36478E-35	-3.199257046	0.428112172	3.627369218
938	AP2B1	P63010	adaptor related protein complex 2 subunit beta 1		yes	no	no	no	no	yes	5.07115E-13	0.013495948	6.26163E-27	1.12131E-13	0.004170474	1.1831E-27	-2.571233948	0.697148685	3.268382633
939	PPP3R1	P63098	protein phosphatase 3 regulatory subunit B, alpha	Myristic acid; Voclosporin	yes	no	no	no	no	yes	4.90202E-17	5.48758E-05	3.02411E-30	1.86037E-18	4.06193E-06	3.68594E-31	-2.14918771	0.7186533	2.86784101
940	YWHAZ	P63104	tyrosine 3-monooxygenase/tryptophan 5-monooxygenase activation protein zeta	Phenethyl Isothiocyanate	yes	no	no	no	no	yes	8.93535E-10	0.013192273	1.04898E-38	3.68831E-10	0.004040769	8.5553E-41	-4.640085489	1.480099596	6.120185085
941	DYNLL1	P63167	dynein light chain LC8-type 1		yes	no	no	no	no	yes	5.53475E-13	0.023557754	7.3212E-30	1.25391E-13	0.008379174	9.38785E-31	-3.662666804	0.810286229	4.472953034
942	SKP1	P63208	S-phase kinase associated protein 1	1-naphthaleneacetic acid; (2S)-2-(1H-indol-3-yl)hexanoic acid; (2S)-2-(1H-indol-3-yl)pentanoic acid; (2S)-8-[(tert-butoxycarbonyl)amino]-2-(1H-indol-3-yl)octanoic acid; Indoleacetic acid	yes	no	no	no	no	yes	2.95521E-13	0.376245555	5.34934E-25	6.03896E-14	0.268370063	1.27735E-25	-2.109535624	0.20554726	2.315082884
943	GNG5	P63218	G protein subunit gamma 5		yes	no	no	no	no	yes	4.68722E-15	0.138906945	1.06893E-20	5.22454E-16	0.077094359	3.43879E-21	-2.712470903	0.421664866	3.134135769
944	EIF5A	P63241	eukaryotic translation initiation factor 5A		yes	no	no	no	no	yes	5.63521E-11	0.985929854	7.11772E-25	1.97118E-11	0.98101584	1.72219E-25	-2.572510365	0.00598597	2.578496335
945	TMSB10	P63313	thymosin beta 10		yes	no	no	no	no	yes	4.59308E-09	0.015814088	3.95953E-22	2.05409E-09	0.005108946	1.13565E-22	-2.036125608	0.760363751	2.796489359
946	PPP2CA	P67775	protein phosphatase 2 catalytic subunit alpha	Vitamin E; 2,6,8-Trimethyl-3-Amino-9-Benzyl-9-Methoxynonanoic Acid; (2S,3S,4E,6E,8S,9S)-3-amino-9-methoxy-2,6,8-trimethyl-10-phenyldeca-4,6-dienoic	yes	no	no	no	no	yes	1.11008E-15	0.080458797	5.89928E-35	8.94623E-17	0.038456167	2.53934E-36	-3.435682566	0.477228157	3.912910724
947	YBX1	P67809	Y-box binding protein 1		yes	no	no	no	no	yes	1.25842E-09	0.111408255	5.83049E-07	5.29709E-10	0.058354301	3.68534E-07	-2.458195806	-0.646802081	1.811393725
948	UBE2L3	P68036	ubiquitin conjugating enzyme E2 L3		yes	no	no	no	no	yes	1.14552E-09	0.396714124	8.02327E-26	4.79594E-10	0.289941043	1.74498E-26	-1.901347005	0.22643073	2.127777735
949	EEF1A1	P68104	eukaryotic translation elongation factor 1 alpha 1	Zinc; Guanosine-5'-Diphosphate; Copper; Artenimol; Zinc acetate; Zinc chloride; Zinc sulfate, unspecified form	yes	no	no	no	no	yes	2.17316E-09	0.759851236	7.27357E-18	9.37402E-10	0.677535957	2.67609E-18	-1.604698821	-0.078774068	1.525924753
950	EIF4G2	P78344	eukaryotic translation initiation factor 4 gamma 2		yes	no	no	no	no	yes	7.80642E-15	0.705114049	1.71825E-28	9.33799E-16	0.61086455	2.70155E-29	-3.716003632	0.139123678	3.85512731
951	PIP4K2B	P78356	phosphatidylinositol-5-phosphate 4-kinase type 2 beta	Fostamatinib	yes	no	no	no	no	yes	1.483E-13	0.6137978	8.16527E-32	2.80764E-14	0.512842384	7.58442E-33	-4.058081962	-0.193040942	3.86504102
952	CCT2	P78371	chaperonin containing TCP1 subunit 2		yes	no	no	no	no	yes	4.84593E-16	0.206372407	1.00869E-25	3.16182E-17	0.125768413	2.22123E-26	-3.457065425	0.356573931	3.813639355
953	SRPX	P78539	sushi repeat containing protein X-linked		no	yes	no	yes	no	no	3.8134E-08	1.85061E-10	0.111683069	1.87646E-08	5.03111E-13	0.100094749	1.235308187	1.515437798	0.28012961
954	NUCB2	P80303	nucleobindin 2	Calcium citrate; Calcium Phosphate; Calcium phosphate dihydrate	yes	no	no	no	no	yes	2.31415E-07	0.005524699	8.54543E-25	1.21737E-07	0.001356768	2.07537E-25	-1.255752663	0.643913954	1.899666617
955	S100A12	P80511	S100 calcium binding protein A12	Olopatadine; Amlexanox	no	yes	no	no	yes	no	0.004650917	0.287742164	1.04573E-07	0.003584599	0.191654273	6.35872E-08	1.141336619	-0.487997338	-1.629333957
956	DCD	P81605	dermcidin	Zinc; Zinc acetate; Zinc chloride; Zinc sulfate, unspecified form	no	yes	no	no	yes	no	0.000532162	0.900283251	5.32847E-09	0.000373261	0.859031814	2.98413E-09	1.16688344	0.050561953	-1.116321487
957	ARF5	P84085	ADP ribosylation factor 5		yes	no	no	no	no	yes	4.11254E-16	0.001470483	4.73299E-25	2.5156E-17	0.000267512	1.12374E-25	-3.270032882	0.841647331	4.111680213
958	RHOG	P84095	ras homolog family member G		yes	no	no	no	no	yes	1.51646E-12	0.044786751	7.49734E-24	3.81349E-13	0.018973997	1.94313E-24	-2.36225294	0.552801311	2.915054251
959	HSPG2	P98160	heparan sulfate proteoglycan 2	Palifermin	no	yes	no	no	yes	no	1.67308E-08	0.441764457	4.75771E-15	7.91434E-09	0.333875448	2.00915E-15	1.125809685	-0.132089396	-1.257899081

960	ARHGAP4	P98171	Rho GTPase activating protein 4		yes	no	no	no	no	yes	4.14889E-12	0.053996078	3.86887E-27	1.15049E-12	0.024049907	7.09965E-28	-3.135958078	0.642927392	3.77888547
961	EFNB1	P98172	ephrin B1		yes	no	no	no	no	yes	2.14744E-13	0.598954572	4.50843E-13	4.2618E-14	0.49668305	2.07956E-13	-2.498506236	-0.181766526	2.31673971
962	RBM3	P98179	RNA binding motif protein 3		yes	no	no	no	no	yes	2.5279E-07	0.744203152	1.43574E-12	1.33554E-07	0.659565639	6.80463E-13	-1.706680586	0.11482306	1.821503646
963	MPP1	Q00013	MAGUK p55 scaffold protein 1		yes	no	no	no	no	yes	1.2907E-11	0.116653306	3.62238E-38	4.02941E-12	0.062370141	4.59567E-40	-2.961934907	0.47525574	3.437190647
964	HDLBP	Q00341	high density lipoprotein binding protein		yes	no	no	no	no	yes	3.24709E-11	0.0487661	6.17309E-23	1.09021E-11	0.021161545	1.69501E-23	-2.066944134	0.531770222	2.598714356
965	PURA	Q00577	purine rich element binding protein A		yes	no	no	no	no	yes	1.33445E-13	0.012479159	6.00211E-34	2.49717E-14	0.003777108	3.31789E-35	-3.455515716	0.82623653	4.281752245
966	FKBP3	Q00688	FKBP prolyl isomerase 3		yes	no	no	no	no	yes	1.88845E-10	0.66109838	5.74554E-25	7.20469E-11	0.563146785	1.37456E-25	-2.895118065	-0.167044056	2.72807401
967	PLCB2	Q00722	phospholipase C beta 2		yes	no	no	no	no	yes	2.34263E-18	0.863685292	6.39561E-26	2.97208E-20	0.808316618	1.37939E-26	-3.479182931	-0.049042316	3.430140615
968	SORD	Q00796	sorbitol dehydrogenase	NADH; Cp-166572, 2-Hydroxymethyl-4-(4-N,N-Dimethylaminosulfonyl-1-Piperazino)-Pyrimidine	no	no	no	yes	no	yes	0.834075276	1.20501E-05	1.68112E-16	0.823115519	5.321E-07	6.61173E-17	-0.052535492	1.525478435	1.578013927
969	HNRNPU	Q00839	heterogeneous nuclear ribonucleoprotein U		yes	no	yes	no	no	no	0.001882034	2.02846E-07	0.325114793	0.001402785	2.6654E-09	0.303754736	-1.206102148	-1.556898124	-0.350795976
970	SPTBN1	Q01082	spectrin beta, non-erythrocytic 1	Calcium; 1D-myo-inositol 1,4,5-	yes	no	no	no	no	yes	3.35798E-13	0.877949185	2.00877E-25	6.99896E-14	0.824668843	4.5418E-26	-2.674133069	0.04698951	2.721122579
971	SRSF2	Q01130	serine and arginine rich splicing factor 2		yes	no	yes	no	no	no	0.000220449	5.17749E-05	0.51689774	0.00014913	3.68312E-06	0.495584784	-1.184338617	-1.005185926	0.179152691
972	AMPD2	Q01433	adenosine monophosphate deaminase 2		yes	no	no	no	no	yes	1.74959E-15	0.942901616	6.24181E-16	1.56963E-16	0.91689178	2.5284E-16	-3.21839675	0.03235738	3.25075413
973	CAP1	Q01518	cyclase associated actin cytoskeleton regulatory protein 1		yes	no	no	no	no	yes	4.13931E-09	0.25099732	2.40152E-28	1.84365E-09	0.161379791	3.81936E-29	-2.998750457	0.490883394	3.489633851
974	DR1	Q01658	down-regulator of transcription 1		yes	no	no	no	no	yes	6.87222E-11	0.246478319	3.36379E-16	2.45058E-11	0.157632199	1.33972E-16	-2.088030232	0.281981504	2.370011736
975	PLCB3	Q01970	phospholipase C beta 3		yes	no	no	no	no	yes	1.71948E-13	0.017310191	9.91253E-29	3.31898E-14	0.005747935	1.48666E-29	-3.399167474	0.765327992	4.164495466
976	EIF4G1	Q04637	eukaryotic translation initiation factor 4 gamma 1		yes	no	no	no	no	yes	1.88272E-14	0.000437971	6.86675E-32	2.66157E-15	5.75495E-05	6.2227E-33	-2.905583949	0.96592168	3.871505629
977	KRT17	Q04695	keratin 17		no	yes	no	no	yes	no	7.76602E-06	0.075458131	3.69203E-05	4.59909E-06	0.035421216	2.60801E-05	1.903161413	0.796602301	-1.106559112
978	HGFAC	Q04756	HGF activator		no	yes	no	no	yes	no	0.000146867	0.20601772	1.23267E-06	9.74236E-05	0.125458911	7.91994E-07	1.053329366	-0.414429668	-1.467759034
979	PTPN12	Q05209	protein tyrosine phosphatase non-receptor type 12	Tiludronic acid	yes	no	no	no	no	yes	2.14183E-16	0.002874739	8.48313E-34	1.09663E-17	0.000608293	4.95842E-35	-3.556278529	0.865440124	4.421718653
980	PTK2	Q05397	protein tyrosine kinase 2	Endostatin; 7-PYRIDIN-2-YL-N-(3,4,5-TRIMETHOXYPHENYL)-7H-PYRROLO[2,3-D]PYRIMIDIN-2-AMINE; 2-({5-CHLORO-2-[(2-METHOXY-4-MORPHOLIN-4-YLPHENYL)AMINO]PYRIMIDIN-4-YL}AMINO)-N-METHYLBENZAMIDE;	yes	no	no	no	no	yes	8.72903E-14	0.426528234	3.70304E-13	1.52669E-14	0.318177625	1.70135E-13	-3.93654337	-0.413799115	3.522744255
981	PRKCD	Q05655	protein kinase C delta	13-Acetylphorbol; Ingenol mebutate; Fostamatinib	yes	no	no	no	no	yes	3.74629E-11	0.770832284	6.12671E-25	1.27682E-11	0.691498297	1.46852E-25	-3.153191556	-0.119263783	3.033927773
982	CALD1	Q05682	caldesmon 1		yes	no	no	no	no	yes	6.603E-12	0.444217908	2.47741E-31	1.91777E-12	0.336156358	2.45833E-32	-4.048261679	0.33504026	4.383301938
983	COL14A1	Q05707	collagen type XIV alpha 1 chain		no	yes	no	no	yes	no	8.89518E-09	0.797370859	1.11181E-18	4.11105E-09	0.72393645	3.96968E-19	1.559680993	-0.06508911	-1.624770103
984	BTK	Q06187	Bruton tyrosine kinase	Dasatinib; Inositol 1,3,4,5-Tetrakisphosphate; XL418; Ibrutinib; Acalabrutinib; Fostamatinib; Zanubrutinib	yes	no	no	no	no	yes	2.98276E-12	0.09773339	1.11233E-34	8.06846E-13	0.049508804	5.19121E-36	-4.54344284	0.74694614	5.29038898
985	PSME1	Q06323	proteasome activator subunit 1	Copper	yes	no	no	no	no	yes	1.30815E-11	0.015735857	2.98084E-31	4.10759E-12	0.005062283	3.03892E-32	-1.951332849	0.537573341	2.48890619
986	APLP2	Q06481	amyloid beta precursor like protein 2	Zinc; Zinc acetate; Zinc chloride; Zinc sulfate, unspecified form	no	no	no	yes	no	yes	0.035633586	1.98292E-05	2.06812E-19	0.029675819	9.34405E-07	7.12176E-20	-0.629796794	1.752837512	2.382634306
987	PRDX1	Q06830	peroxiredoxin 1	Zinc; Copper; Artenimol; Zinc acetate; Zinc chloride; Zinc sulfate, unspecified form	yes	no	no	no	no	yes	2.35212E-12	0.310330303	6.3259E-35	6.20268E-13	0.210777129	2.7803E-36	-2.801791661	0.286066541	3.087858202
988	KLC1	Q07866	kinesin light chain 1		yes	no	no	no	no	yes	3.35006E-14	0.097724534	6.112E-30	5.14577E-15	0.049460038	7.78193E-31	-3.630045641	0.541791007	4.171836648
989	SRSF1	Q07955	serine and arginine rich splicing factor 1	Copper	yes	no	yes	no	no	no	1.18596E-08	1.00615E-05	0.012924215	5.53484E-09	4.05741E-07	0.010751635	-1.970809795	-1.345180918	0.625628877
990	ARHGAP1	Q07960	Rho GTPase activating protein 1		yes	no	no	no	no	yes	1.97845E-17	0.956142025	4.47069E-11	5.88846E-19	0.935346911	2.26051E-11	-3.550073399	-0.033634156	3.516439243
991	DHX9	Q08211	DExH-box helicase 9		yes	no	no	no	no	yes	2.9778E-09	0.430028238	1.73715E-15	1.30608E-09	0.321887924	7.17844E-16	-1.843075236	-0.213818228	1.629257008
992	CRYZ	Q08257	crystallin zeta	Dicoumarol; Nicotinamide adenine dinucleotide phosphate; Medical Cannabis;	yes	no	no	no	no	yes	2.71992E-07	0.046289886	6.45331E-08	1.44192E-07	0.019799571	3.86848E-08	-1.704576848	-0.645065498	1.05951135
993	MFGE8	Q08431	milk fat globule EGF and factor V/VIII domain		no	yes	no	no	yes	no	0.000605686	0.820173648	1.07084E-06	0.000427027	0.75328137	6.86563E-07	1.247215968	0.095844734	-1.151371234
994	DMTN	Q08495	dematin actin binding protein		yes	no	no	no	no	yes	1.98733E-10	0.209337098	1.36378E-25	7.59094E-11	0.128335588	3.03405E-26	-3.496342225	-0.527634493	2.968707732
995	DSC1	Q08554	desmocollin 1		no	yes	no	no	yes	no	1.30815E-11	0.487798474	4.22963E-17	4.1002E-12	0.377728859	1.61941E-17	1.998438296	0.166373062	-1.832065234
996	CD47	Q08722	CD47 molecule		yes	no	no	no	no	yes	4.6507E-12	0.971477602	6.35589E-24	1.31282E-12	0.958502892	1.63289E-24	-3.366220634	-0.015825142	3.350395491
997	FCHO2	Q0JRZ9	FCH and mu domain containing endocytic adaptor 2		yes	no	no	no	no	yes	4.83007E-07	0.078196218	5.20129E-18	2.6131E-07	0.037068503	1.89952E-18	-1.395294392	0.460305609	1.855600001
998	NEXN	Q0ZGT2	nexilin F-actin binding protein		yes	no	no	no	no	yes	7.42018E-12	0.041252851	3.21273E-34	2.18873E-12	0.017105476	1.60127E-35	-3.977351973	0.865107392	4.842459365
999	AP1B1	Q10567	adaptor related protein complex 1 subunit beta 1		yes	no	no	no	no	yes	1.19734E-13	0.377285361	8.15695E-29	2.21253E-14	0.26941628	1.21227E-29	-2.711093002	0.240791578	2.95188458
1000	BST1	Q10588	bone marrow stromal cell antigen 1	Etheno-NAD; Nicotinamide; Adenosine 5'-[gamma-thio]triphosphate; Nicotinamide Mononucleotide; Etheno-Nadp	yes	no	no	no	no	yes	6.37518E-11	0.945726335	3.67526E-12	2.25312E-11	0.922158166	1.76186E-12	-1.592102887	-0.014232323	1.577870564
1001	WASHC5	Q12768	WASH complex subunit 5		yes	no	no	no	no	yes	3.92723E-16	0.496324866	3.89461E-16	2.36666E-17	0.386580174	1.55819E-16	-2.875669966	-0.222622691	2.653047274
1002	STX4	Q12846	syntaxin 4		yes	no	no	no	no	yes	8.08429E-12	0.131493135	6.2695E-22	2.39928E-12	0.072206015	1.84364E-22	-2.126649254	0.371997329	2.498646584
1003	AIMP1	Q12904	aminoacyl tRNA synthetase complex interacting multifunctional protein 1		yes	no	no	no	no	yes	2.75873E-09	0.070366541	8.17572E-30	1.20374E-09	0.032616661	1.05206E-30	-2.549621404	0.631855412	3.181476816
1004	ILF2	Q12905	interleukin enhancer binding factor 2		no	yes	no	no	yes	no	0.000849175	0.726300507	7.33798E-08	0.000609467	0.638433612	4.4121E-08	1.183304729	0.139245434	-1.044059296
1005	LMAN2	Q12907	lectin, mannose binding 2		yes	no	no	no	no	yes	8.20297E-05	0.110037977	2.8895E-06	5.33732E-05	0.057387273	1.89971E-06	-1.401884753	0.291385099	1.693269852
1006	PTPRJ	Q12913	protein tyrosine phosphatase receptor type J		yes	no	no	no	no	yes	6.90461E-15	0.006536131	9.19332E-30	8.13411E-16	0.001658465	1.19134E-30	-3.465823407	0.851818356	4.317641764
1007	PTP4A2	Q12974	protein tyrosine phosphatase 4A2		yes	no	no	no	no	yes	2.41098E-17	0.020643833	5.76272E-22	7.86546E-19	0.007062118	1.67894E-22	-1.848054671	0.292573757	2.140628428
1008	STK4	Q13043	serine/threonine kinase 4		yes	no	no	no	no	yes	5.18987E-14	0.116880394	3.62361E-14	8.67723E-15	0.062544515	1.58441E-14	-2.715570789	-0.492189777	2.223381011
1009	FLII	Q13045	FLII actin remodeling protein		yes	no	no	no	no	yes	1.0516E-16	0.007255691	2.25183E-26	4.62187E-18	0.001870634	4.5812E-27	-3.251072774	0.682834801	3.933907576
1010	LCP2	Q13094	lymphocyte cytosolic protein 2		yes	no	no	no	no	yes	6.24862E-13	0.419633474	3.27809E-31	1.43829E-13	0.312205784	3.40137E-32	-4.611235807	0.370511455	4.981747263
1011	GPS1	Q13098	G protein pathway suppressor 1		yes	no	no	no	no	yes	5.15674E-13	0.857436171	7.21222E-25	1.14958E-13	0.798338327	1.74832E-25	-3.068801033	-0.065395603	3.00340543
1012	EIF2B5	Q13144	eukaryotic translation initiation factor 2B subunit epsilon		yes	no	no	no	no	yes	2.56621E-13	0.636843178	1.09113E-24	5.19752E-14	0.538157012	2.67961E-25	-3.121513009	0.164398928	3.285911937
1013	PAK1	Q13153	p21 (RAC1) activated kinase 1	Fostamatinib	yes	no	no	no	no	yes	7.13393E-20	0.272593724	4.82626E-21	2.90917E-22	0.178970687	1.50889E-21	-2.41851068	-0.215269745	2.203240935
1014	PAK2	Q13177	p21 (RAC1) activated kinase 2	Fostamatinib	yes	no	no	no	no	yes	2.03245E-18	0.059613322	3.14043E-20	2.48646E-20	0.026848954	1.03732E-20	-2.178354187	-0.334492855	1.843861332
1015	STK3	Q13188	serine/threonine kinase 3	Fostamatinib	yes	no	no	no	no	yes	8.36117E-16	0.001123776	4.4703E-29	6.2131E-17	0.000197055	6.42086E-30	-2.770053168	0.819819381	3.589872549
1016	PSMD2	Q13200	proteasome 26S subunit ubiquitin receptor, non-ATPase		yes	no	no	no	no	yes	3.68101E-10	0.013989759	4.04596E-17	1.45606E-10	0.004373781	1.54542E-17	-1.618695003	0.645571588	2.264266591
1017	MMRN1	Q13201	multimerin 1		yes	no	no	no	no	yes	4.43479E-06	0.113482792	1.64656E-09	2.5821E-06	0.059800855	8.96766E-10	-2.455389204	-0.845096167	1.610293037
1018	SEMA3B	Q13214	semaphorin 3B		no	yes	no	no	yes	no	7.37808E-05	0.108698173	1.16298E-12	4.77051E-05	0.056294523	5.50139E-13	1.002726473	-0.412333656	-1.415060129
1019	G3BP1	Q13283	G3BP stress granule assembly factor 1		yes	no	no	no	no	yes	2.27965E-15	0.053024009	7.46277E-27	2.17945E-16	0.023472794	1.43372E-27	-2.811388405	0.46611346	3.277501865
1020	EIF3I	Q13347	eukaryotic translation initiation factor 3 subunit I		yes	no	no	no	no	yes	8.29493E-16	0.900283251	1.14054E-29	6.08871E-17	0.859083157	1.51418E-30	-3.358810924	0.038646282	3.397457206
1021	UBE2V1	Q13404	ubiquitin conjugating enzyme E2 V1		yes	no	no	no	no	yes	2.74504E-16	0.511199498	7.27801E-28	1.51742E-17	0.402798336	1.23663E-28	-2.70233295	-0.148757787	2.553575163
1022	DYNC1I2	Q13409	dynein cytoplasmic 1 intermediate chain 2		yes	no	no	no	no	yes	1.9096E-09	5.96511E-06	1.21673E-15	8.17658E-10	1.77838E-07	4.99484E-16	-1.158912797	0.660261523	1.81917432
1023	ILK	Q13418	integrin linked kinase		yes	no	no	no	no	yes	2.00381E-11	0.283715108	4.99587E-38	6.46448E-12	0.18832924	6.7848E-40	-4.898069259	0.556128293	5.454197552
1024	MSLN	Q13421	mesothelin	Amatuximab	no	yes	no	no	yes	no	0.00035601	0.588868116	6.92547E-08	0.000245997	0.484895349	4.15779E-08	1.009905599	-0.154500461	-1.16440606
1025	PDAP1	Q13442	PDGFA associated protein 1		yes	no	no	no	no	yes	1.57064E-12	0.565067127	6.97028E-24	3.97108E-13	0.461117307	1.80021E-24	-3.26626246	-0.205359843	3.060902617
1026	MYO9B	Q13459	myosin IXB		yes	no	no	no	no	yes	9.14409E-07	0.109560427	1.21713E-18	5.07545E-07	0.056989293	4.35673E-19	-1.697479491	0.545937247	2.243416738
1027	ROCK1	Q13464	Rho associated coiled-coil containing protein kinase 1	Hydroxyfasudil; (S)-2-METHYL-1-[(4-METHYL-5-ISOQUINOLINE)SULFONYL]-HOMOPIPERAZINE; Fasudil; Y-27632;	yes	no	no	no	no	yes	1.75813E-16	0.043706063	2.48898E-31	8.84242E-18	0.018397341	2.48108E-32	-2.971118912	0.487258955	3.458377867
1028	PICALM	Q13492	phosphatidylinositol binding clathrin assembly protein		yes	no	no	no	no	yes	5.84212E-13	0.011538074	5.78484E-34	1.33678E-13	0.003424304	3.17157E-35	-2.707480616	0.689219988	3.396700604
1029	MTM1	Q13496	myotubularin 1		yes	no	no	no	no	yes	9.42157E-16	0.394957074	1.99013E-15	7.19103E-17	0.287941066	8.28697E-16	-2.524512924	-0.246430184	2.27808274
1030	ASAH1	Q13510	N-acylsphingosine amidohydrolase 1		yes	no	no	no	no	yes	0.000504046	0.00023939	3.81431E-09	0.000353083	2.67287E-05	2.11541E-09	-1.065106541	0.954691765	2.019798305
1031	PIN1	Q13526	peptidylprolyl cis/trans isomerase, NIMA-interacting 1	Beta-(2-Naphthyl)-Alanine; 3,6,9,12,15,18-HEXAOXAICOSANE	yes	no	no	no	no	yes	9.86147E-13	0.342899236	2.48181E-17	2.38159E-13	0.237869837	9.40097E-18	-2.481241524	-0.263540364	2.217701159
1032	SNX1	Q13596	sorting nexin 1		yes	no	no	no	no	yes	1.96837E-14	0.000258819	3.60001E-23	2.80821E-15	2.94352E-05	9.80337E-24	-1.896567661	0.813408809	2.70997647
1033	DNASE1L3	Q13609	deoxyribonuclease 1L3		no	yes	no	no	yes	no	2.4108E-09	0.965260223	2.46187E-15	1.04647E-09	0.947328339	1.03071E-15	1.890536467	-0.013882836	-1.904419303
1034	CUL3	Q13618	cullin 3		yes	no	no	no	no	yes	1.45095E-11	0.074360329	1.04892E-24	4.59375E-12	0.034838505	2.56646E-25	-1.47996981	0.325539189	1.805508999
1035	ENPP2	Q13822	ectonucleotide pyrophosphatase/phosphodiesterase 2		no	yes	no	no	yes	no	2.42881E-10	0.680343995	3.68364E-14	9.37627E-11	0.584164871	1.61232E-14	2.191229463	0.129921314	-2.061308148
1036	BLMH	Q13867	bleomycin hydrolase		yes	no	no	no	no	yes	7.56636E-06	0.01366271	5.61092E-17	4.47399E-06	0.004240578	2.16352E-17	-1.318700141	0.735727029	2.05442717
1037	CKAP5	Q14008	cytoskeleton associated protein 5		yes	no	no	no	no	yes	2.82188E-14	0.005476846	9.89741E-36	4.24497E-15	0.001340053	3.40826E-37	-3.982836735	0.972047237	4.954883973
1038	CIRBP	Q14011	cold inducible RNA binding protein		yes	no	no	no	no	yes	3.01954E-13	0.231038181	6.26626E-21	6.22514E-14	0.144987712	1.96761E-21	-2.666129364	0.320414937	2.986544301
1039	COTL1	Q14019	coactosin like F-actin binding protein 1		yes	no	no	no	no	yes	2.40317E-13	0.053202665	2.13784E-33	4.81287E-14	0.023575989	1.43362E-34	-3.177956945	0.576737437	3.754694381

1040	NID2	Q14112	nidogen 2		yes	no	no	no	no	yes	5.81478E-09	0.036041258	4.24195E-12	2.63416E-09	0.014256465	2.03737E-12	-1.38715857	0.34181378	1.72897235
1041	SEPTIN6	Q14141	septin 6		yes	no	no	no	no	yes	3.03899E-13	0.168874044	1.08842E-30	6.27902E-14	0.098401459	1.23785E-31	-4.006997921	0.521040378	4.528038298
1042	EIF3A	Q14152	eukaryotic translation initiation factor 3 subunit A		yes	no	no	no	no	yes	5.85233E-15	0.814683941	3.50825E-26	6.73535E-16	0.745286306	7.29826E-27	-3.157070187	-0.070956311	3.086113876
1043	ARHGEF7	Q14155	Rho guanine nucleotide exchange factor 7		yes	no	no	no	no	yes	1.25528E-09	0.014244885	6.43088E-25	5.27819E-10	0.004493059	1.55017E-25	-2.260135798	0.81265059	3.072786389
1044	DCTN1	Q14203	dynactin subunit 1		yes	no	no	no	no	yes	1.84613E-17	3.64737E-05	6.79227E-28	5.13049E-19	2.25479E-06	1.14487E-28	-2.577125004	0.937724972	3.514849976
1045	DYNC1H1	Q14204	dynein cytoplasmic 1 heavy chain 1		yes	no	no	no	no	yes	8.43605E-07	0.519389106	2.12589E-09	4.66716E-07	0.411840025	1.17131E-09	-1.486122028	0.215295336	1.701417364
1046	EBI3	Q14213	Epstein-Barr virus induced 3		no	yes	no	yes	no	no	4.42105E-08	1.34587E-07	0.860151624	2.18148E-08	1.40259E-09	0.850408175	1.756894553	1.713226304	-0.043668249
1047	FLOT2	Q14254	flotillin 2		yes	no	yes	no	no	no	3.51878E-06	1.18648E-10	0.000261874	2.02963E-06	2.68799E-13	0.000195071	-1.430912198	-2.342935747	-0.912023549
1048	TRIM25	Q14258	tripartite motif containing 25		yes	no	no	no	no	yes	1.50951E-13	0.101009873	9.36805E-33	2.86582E-14	0.051580483	7.30088E-34	-2.834491382	0.422256916	3.256748298
1049	FGL2	Q14314	fibrinogen like 2		no	yes	no	no	yes	no	1.57141E-10	0.634397225	5.17766E-12	5.88835E-11	0.534759562	2.5032E-12	1.311238092	0.088600927	-1.222637165
1050	GNA13	Q14344	G protein subunit alpha 13		yes	no	no	no	no	yes	4.65481E-14	0.017310191	6.20727E-28	7.63499E-15	0.005740641	1.03547E-28	-2.601661312	0.618385854	3.220047166
1051	SPARCL1	Q14515	SPARC like 1		no	yes	no	no	yes	no	2.28381E-11	0.00483609	6.46296E-11	7.44025E-12	0.001159172	3.29444E-11	1.703703115	0.5732479	-1.130455215
1052	PDIA5	Q14554	protein disulfide isomerase family A member 5		yes	no	no	no	no	yes	1.52156E-13	0.038746834	3.79853E-32	2.89558E-14	0.01576559	3.27976E-33	-4.60741388	0.893704042	5.501117922
1053	PRPSAP1	Q14558	phosphoribosyl pyrophosphate synthetase associated protein 1		yes	no	no	no	no	yes	3.50506E-11	0.497904398	9.05982E-25	1.18476E-11	0.388712858	2.20441E-25	-2.075173645	0.179404528	2.254578173
1054	DSC3	Q14574	desmocollin 3		no	yes	no	no	yes	no	5.13659E-09	0.016346583	1.2064E-17	2.30879E-09	0.005340229	4.47687E-18	1.722080909	-0.578118102	-2.300199011
1055	IHH	Q14623	Indian hedgehog signaling molecule		no	no	yes	no	yes	no	0.027067842	1.01025E-05	1.75339E-09	0.022296936	4.11975E-07	9.58127E-10	-0.592750773	-1.618396122	-1.025645348
1056	ITIH4	Q14624	inter-alpha-trypsin inhibitor heavy chain 4	Zinc; Zinc acetate; Zinc chloride; Zinc sulfate, unspecified form	no	yes	no	no	yes	no	3.33192E-07	0.112152321	2.02455E-14	1.78145E-07	0.058896484	8.72381E-15	1.171582116	-0.307941002	-1.479523119
1057	RASA3	Q14644	RAS p21 protein activator 3		yes	no	no	no	no	yes	3.24086E-11	0.196476369	1.8216E-37	1.08665E-11	0.118046065	3.38402E-39	-4.255680254	0.593440844	4.849121098
1058	CLINT1	Q14677	clathrin interactor 1		yes	no	no	no	no	yes	8.01692E-13	0.877949185	3.45096E-33	1.90706E-13	0.825041508	2.45492E-34	-4.456606944	-0.075599954	4.381006991
1059	MESD	Q14696	mesoderm development LRP chaperone		yes	no	no	no	no	yes	4.44074E-15	9.89791E-05	1.73827E-26	4.86932E-16	8.38654E-06	3.48914E-27	-2.360630463	0.867432102	3.228062565
1060	GANAB	Q14697	glucosidase II alpha subunit	Miglitol	yes	no	no	no	no	yes	1.0005E-10	0.002837437	5.23753E-14	3.65383E-11	0.000599114	2.3067E-14	-2.073121306	0.557289556	2.630410862
1061	PPP2R5D	Q14738	protein phosphatase 2 regulatory subunit B'delta		yes	no	no	no	no	yes	5.04626E-10	0.128788889	1.35781E-24	2.03356E-10	0.070044522	3.35299E-25	-1.621974834	0.334570235	1.956545069
1062	LBR	Q14739	lamin B receptor		yes	no	no	no	no	yes	9.51096E-10	0.876179871	1.8549E-19	3.93453E-10	0.822584818	6.3707E-20	-2.326426464	0.058870694	2.385297159
1063	LTBP1	Q14766	latent transforming growth factor beta binding protein 1		yes	no	no	no	no	yes	6.19425E-08	0.971477602	3.44181E-17	3.10414E-08	0.958834601	1.31154E-17	-2.721239632	-0.019633201	2.701606431
1064	LTBP2	Q14767	latent transforming growth factor beta binding protein 2		no	yes	no	no	yes	no	1.51022E-05	0.31724079	4.16691E-08	9.15578E-06	0.216189464	2.47334E-08	1.53162791	0.345871338	-1.185756572
1065	GOLGB1	Q14789	golgin B1		yes	no	no	no	no	yes	0.000214262	0.870487884	1.75885E-10	0.000144459	0.815663318	9.15685E-11	-1.67801323	-0.093378192	1.584635039
1066	LASP1	Q14847	LIM and SH3 protein 1		yes	no	no	no	no	yes	4.09541E-13	0.08375637	1.05607E-27	8.77721E-14	0.040492998	1.8279E-28	-3.425704999	-0.559577354	2.866127645
1067	DRAP1	Q14919	DR1 associated protein 1		yes	no	no	no	no	yes	1.02215E-11	0.102133806	4.67393E-14	3.12618E-12	0.052339526	2.05212E-14	-2.253508373	-0.435389888	1.818118485
1068	KPNB1	Q14974	karyopherin subunit beta 1		yes	no	no	no	no	yes	5.63868E-15	0.013401255	6.29082E-23	6.43837E-16	0.00413514	1.73019E-23	-3.228266595	0.853149662	4.081416256
1069	GAPVD1	Q14C86	GTPase activating protein and VPS9 domains 1		yes	no	no	no	no	yes	5.81478E-09	0.262709705	3.68955E-19	2.6347E-09	0.171171978	1.28725E-19	-1.452337636	0.260071272	1.712408908
1070	SEPTIN2	Q15019	septin 2		yes	no	no	no	no	yes	1.7078E-12	0.266583792	6.08953E-32	4.33333E-13	0.174420932	5.35283E-33	-4.056848777	0.44746321	4.504311987
1071	ACAP1	Q15027	ArfGAP with coiled-coil, ankyrin repeat and PH domains		yes	no	no	no	no	yes	1.24569E-13	0.311895585	6.1949E-18	2.3198E-14	0.211981594	2.27081E-18	-2.539085761	-0.286838045	2.252247716
1072	EFTUD2	Q15029	elongation factor Tu GTP binding domain containing 2		yes	no	no	no	no	yes	1.35684E-12	0.475066647	1.63162E-11	3.38134E-13	0.365071605	8.08046E-12	-1.738932971	-0.166394759	1.572538212
1073	EIF4H	Q15056	eukaryotic translation initiation factor 4H		yes	no	no	no	no	yes	1.38457E-14	0.330314407	2.34075E-24	1.83815E-15	0.227243021	5.89696E-25	-2.958377321	0.279902925	3.238280246
1074	POSTN	Q15063	periostin		no	yes	no	no	yes	no	0.000165114	0.331146396	1.96582E-05	0.000109976	0.228366477	1.3628E-05	1.067778416	-0.278720939	-1.346499355
1075	PCOLCE	Q15113	procollagen C-endopeptidase enhancer		no	yes	no	no	yes	no	4.01395E-10	0.227047217	3.89209E-13	1.59867E-10	0.141393225	1.78997E-13	1.431919791	0.233202984	-1.198716807
1076	PLCB4	Q15147	phospholipase C beta 4		yes	no	no	no	no	yes	3.56715E-13	0.889767054	2.21128E-08	7.48342E-14	0.840581925	1.2925E-08	-2.115527729	0.066399708	2.181927437
1077	PLEC	Q15149	plectin		yes	no	no	no	no	yes	5.12438E-09	0.008268046	1.80162E-17	2.30098E-09	0.002251516	6.73466E-18	-1.98996086	0.748218941	2.7381798
1078	PPP2R5A	Q15172	protein phosphatase 2 regulatory subunit B'alpha		yes	no	no	no	no	yes	1.6717E-16	0.001470483	2.36266E-26	8.28262E-18	0.000266546	4.82809E-27	-3.206086779	0.84596788	4.052054658
1079	PTGES3	Q15185	prostaglandin E synthase 3	Acetaminophen; Grn163l; Copper	yes	no	no	no	no	yes	7.11707E-14	0.257517546	7.46213E-26	1.21896E-14	0.165922044	1.61617E-26	-3.43286366	-0.366053719	3.06680994
1080	PTPA	Q15257	protein phosphatase 2 phosphatase activator		yes	no	no	no	no	yes	6.42933E-17	0.057194519	2.47193E-27	2.6801E-18	0.025578156	4.48016E-28	-2.995607717	0.492834435	3.488442152
1081	RAB35	Q15286	RAB35, member RAS oncogene family		yes	no	no	no	no	yes	1.72913E-09	0.002808392	1.2663E-21	7.37162E-10	0.000589164	3.81554E-22	-1.582430116	0.763604406	2.346034522
1082	PCBP1	Q15365	poly(rC) binding protein 1		yes	no	no	no	no	yes	5.14386E-14	0.046085104	5.36449E-33	8.55367E-15	0.019691098	3.88908E-34	-3.620503512	0.651898242	4.272401754
1083	PCBP2	Q15366	poly(rC) binding protein 2		yes	no	no	no	no	yes	1.6655E-14	0.025477088	3.20065E-31	2.30921E-15	0.009211924	3.30651E-32	-3.63159244	0.715817759	4.347410199
1084	ANGPT1	Q15389	angiopoietin 1		no	no	no	yes	no	yes	0.021412422	0.004726261	1.12809E-08	0.017512199	0.001126422	6.4557E-09	-0.662149598	1.055975577	1.718125175
1085	RSU1	Q15404	Ras suppressor protein 1		yes	no	no	no	no	yes	4.49101E-10	0.249108292	3.76834E-37	1.80088E-10	0.159488	7.51277E-39	-4.585035531	0.643884576	5.228920107
1086	FCN2	Q15485	ficolin 2		yes	no	no	no	no	yes	0.004914515	0.95575484	0.000769597	0.003803349	0.934535091	0.000587223	-1.627278917	0.028603878	1.655882796
1087	MAPRE1	Q15691	microtubule associated protein RP/EB family member 1		yes	no	no	no	no	yes	2.34792E-14	0.047176567	7.50326E-36	3.4256E-15	0.020285185	2.41383E-37	-3.133703798	0.533916545	3.667620343
1088	SLC1A5	Q15758	solute carrier family 1 member 5	Asparagine; Fluciclovine (18F)	no	no	yes	no	yes	no	0.212302188	0.000110938	3.36145E-06	0.191139306	1.01036E-05	2.21761E-06	-0.341958753	-1.35179583	-1.009837078
1089	CD226	Q15762	CD226 molecule		yes	no	no	no	no	yes	4.00078E-13	0.028966997	2.34766E-29	8.50189E-14	0.010841296	3.24439E-30	-3.848389582	0.865421835	4.713811417
1090	VAMP3	Q15836	vesicle associated membrane protein 3		yes	no	no	no	no	yes	1.794E-10	0.000774724	2.97672E-16	6.79558E-11	0.000123212	1.18421E-16	-2.198331744	0.830586478	3.028918222
1091	SEPTIN7	Q16181	septin 7		yes	no	no	no	no	yes	2.12697E-12	0.898756756	1.38925E-32	5.54151E-13	0.854028971	1.12676E-33	-4.38731867	0.063977298	4.451295968
1092	CCDC6	Q16204	coiled-coil domain containing 6		yes	no	no	no	no	yes	1.19293E-18	0.000276622	1.66686E-25	1.35131E-20	3.20722E-05	3.73099E-26	-2.172428509	0.689069587	2.861498096
1093	IGFBP7	Q16270	insulin like growth factor binding protein 7	Insulin pork	no	yes	no	no	yes	no	1.78368E-10	0.921670716	1.96934E-15	6.74842E-11	0.888261718	8.18254E-16	1.900836421	-0.02615622	-1.926992641
1094	EXT1	Q16394	exostosin glycosyltransferase 1		no	yes	no	no	yes	no	6.76455E-16	0.036961638	1.92906E-18	4.79754E-17	0.01476639	6.95755E-19	2.556917649	0.434843933	-2.122073716
1095	PSMD5	Q16401	proteasome 26S subunit, non-ATPase 5		yes	no	no	no	no	yes	3.25756E-14	0.795763558	6.67715E-20	4.9594E-15	0.721411261	2.23883E-20	-1.836788154	-0.056430687	1.780357467
1096	DDB1	Q16531	damage specific DNA binding protein 1		yes	no	no	no	no	yes	3.39777E-13	0.012914013	6.67028E-24	7.09729E-14	0.003937984	1.7197E-24	-1.999280463	0.580930092	2.580210555
1097	PPP2R5E	Q16537	protein phosphatase 2 regulatory subunit B'epsilon		yes	no	no	no	no	yes	1.6717E-16	0.531245062	5.88026E-12	8.33197E-18	0.425333042	2.8562E-12	-2.829934047	0.272125382	3.102059429
1098	CDC37	Q16543	cell division cycle 37, HSP90 cochaperone		yes	no	no	no	no	yes	2.94705E-14	0.004532512	8.18984E-31	4.47332E-15	0.001063935	9.16579E-32	-2.663406191	0.71954657	3.382952761
1099	CCL14	Q16627	C-C motif chemokine ligand 14		no	yes	no	no	yes	no	4.89894E-13	9.14518E-05	6.79861E-09	1.07435E-13	7.54157E-06	3.81979E-09	2.039247308	0.862323898	-1.17692341
1100	DBN1	Q16643	drebrin 1		yes	no	no	no	no	yes	9.50451E-13	0.007619353	8.22846E-23	2.28246E-13	0.001991511	2.29293E-23	-3.184839822	0.881302218	4.06614204
1101	UGP2	Q16851	UDP-glucose pyrophosphorylase 2		yes	no	no	no	no	yes	2.32789E-16	0.08017777	1.55127E-25	1.25184E-17	0.038217949	3.4582E-26	-2.892818986	0.432299043	3.325118029
1102	INF2	Q27J81	inverted formin 2		yes	no	no	no	no	yes	2.17923E-14	0.022407945	2.74845E-36	3.13012E-15	0.007919437	7.97014E-38	-3.931001315	0.744006707	4.675008022
1103	BLTP1	Q2LD37	bridge-like lipid transfer protein family member 1		no	yes	no	no	yes	no	2.24634E-11	0.591444513	5.45504E-10	7.29781E-12	0.487965475	2.89683E-10	1.111051066	0.085396595	-1.025654471
1104	EML3	Q32P44	EMAP like 3		yes	no	no	no	no	yes	1.6326E-16	1.8596E-07	4.54286E-25	7.91517E-18	2.19074E-09	1.07654E-25	-2.196698014	0.96389563	3.160593644
1105	TKFC	Q3LXA3	triokinase and FMN cyclase		yes	no	no	no	no	yes	2.58431E-14	0.920754438	1.08846E-13	3.82903E-15	0.886159956	4.87762E-14	-1.767870421	-0.026430759	1.741439663
1106	RABL6	Q3YEC7	RAB, member RAS oncogene family like 6		yes	no	no	no	no	yes	7.46622E-14	0.627212542	1.32445E-23	1.30244E-14	0.526608446	3.49866E-24	-3.171004875	-0.162278858	3.008726017
1107	ERFE	Q4G0M1	erythroferrone		no	yes	no	yes	no	no	6.78712E-06	0.002046181	0.035423968	4.00093E-06	0.000400521	0.030384038	1.670898327	1.162099605	-0.508798722
1108	ANO6	Q4KMQ2	anoctamin 6		yes	no	no	no	no	yes	7.67912E-13	0.000653306	1.15331E-18	1.81975E-13	9.99744E-05	4.12307E-19	-2.543897398	0.877474962	3.42137236
1109	GRIPAP1	Q4V328	GRIP1 associated protein 1		yes	no	no	no	no	yes	1.47284E-22	0.004532512	1.67389E-18	1.3347E-25	0.00106681	6.02965E-19	-2.182658339	0.680166762	2.862825101
1110	ARHGAP15	Q53QZ3	Rho GTPase activating protein 15		yes	no	no	no	no	yes	2.50462E-14	0.358710645	9.5371E-20	3.68827E-15	0.252150446	3.23233E-20	-2.841406453	-0.277009605	2.564396847
1111	ACTBL2	Q562R1	actin beta like 2		yes	no	no	no	no	yes	0.000125003	0.014115862	6.80031E-21	8.24671E-05	0.004437451	2.15071E-21	-2.432555291	1.612800376	4.045355666
1112	CCDC93	Q567U6	coiled-coil domain containing 93		yes	no	no	no	no	yes	8.13213E-12	0.357534241	5.3768E-06	2.41716E-12	0.250684266	3.58616E-06	-1.917656792	-0.368267285	1.549389508
1113	FLG2	Q5D862	filaggrin 2		no	yes	no	no	yes	no	2.4341E-05	0.519389106	8.7042E-09	1.50546E-05	0.411736239	4.92988E-09	1.601426451	0.269239493	-1.332186958
1114	PPP6R3	Q5H9R7	protein phosphatase 6 regulatory subunit 3		yes	no	no	no	no	yes	3.7579E-11	0.80303176	1.09766E-16	1.28385E-11	0.731049101	4.2872E-17	-2.271849119	-0.086261109	2.18558801
1115	DOCK11	Q5JSL3	dedicator of cytokinesis 11		yes	no	no	no	no	yes	1.45095E-11	0.236047127	4.04071E-08	4.59544E-12	0.14882404	2.39477E-08	-1.329000664	-0.266322355	1.06267831
1116	GNAS	Q5JWF2	GNAS complex locus	Colforsin; 2',5'-DIDEOXY-ADENOSINE 3'-MONOPHOSPHATE	yes	no	no	no	no	yes	1.94846E-12	0.162997269	2.82866E-21	5.02435E-13	0.093943147	8.7282E-22	-1.967975334	0.331491409	2.299466742
1117	SAMD9	Q5K651	sterile alpha motif domain containing 9		yes	no	no	no	no	yes	1.21599E-12	0.404380133	1.21911E-26	3.0138E-13	0.298108961	2.41392E-27	-2.672078077	-0.232520183	2.439557894
1118	LRRK2	Q5S007	leucine rich repeat kinase 2	Fostamatinib	no	yes	no	no	yes	no	1.378E-11	0.00402535	7.2908E-07	4.33941E-12	0.000922725	4.62819E-07	1.801446975	0.715979628	-1.085467347
1119	LY6G6F	Q5SQ64	lymphocyte antigen 6 family member G6F		yes	no	no	no	no	yes	4.21082E-13	0.041252851	1.6455E-22	9.06782E-14	0.017121709	4.6599E-23	-3.542492465	0.71597182	4.258464285
1120	HP1BP3	Q5SSJ5	heterochromatin protein 1 binding protein 3	Artenimol	yes	no	yes	no	no	no	0.001827464	0.005524699	0.420829906	0.001361282	0.001356589	0.398901751	-1.289436554	-1.037603879	0.251832675
1121	MPP7	Q5T2T1	MAGUK p55 scaffold protein 7		yes	no	no	no	no	yes	0.000789474	0.314503331	7.69565E-08	0.000565036	0.214038968	4.63413E-08	-1.118288125	0.438037446	1.556325571
1122	UBR4	Q5T4S7	ubiquitin protein ligase E3 component n-recognin 4	Verpasep caltespen; PCL-016	yes	no	no	no	no	yes	1.603E-10	0.695745004	8.30934E-21	6.02123E-11	0.601486846	2.66562E-21	-2.3052831	-0.126280992	2.179002108
1123	YOD1	Q5VVQ6	YOD1 deubiquitinase		yes	no	no	no	no	yes	5.17093E-11	0.160619712	2.56237E-26	1.80174E-11	0.09191785	5.25941E-27	-1.936188196	0.334704107	2.270892303
1124	FOCAD	Q5VW36	focadhesin		yes	no	no	no	no	yes	7.86544E-11	0.007700063	9.34745E-13	2.83327E-11	0.002020089	4.3836E-13	-1.360220609	0.69687979	2.057100399
1125	PEAR1	Q5VY43	platelet endothelial aggregation receptor 1		yes	no	no	no	no	yes	5.51688E-17	0.185222472	9.20735E-24	2.19975E-18	0.109941748	2.40301E-24	-3.246325124	0.312216396	3.558541521
1126	CARMIL1	Q5VZK9	capping protein regulator and myosin 1 linker 1		yes	no	no	no	no	yes	7.36165E-10	0.237399692	1.66557E-05	3.0087E-10	0.14994797	1.14862E-05	-1.889615238	-0.476460278	1.41315496
1127	STEAP3	Q658P3	STEAP3 metalloreductase		yes	no	yes	no	no	no	0.000363446	1.76639E-05	0.090704923	0.0002513	8.16367E-07	0.080882323	-1.173222083	-1.585825434	-0.412603352
1128	MAP1S	Q66K74	microtubule associated protein 1S		yes	no	no	no	no	yes	1.90774E-14	0.053981363	8.01049E-26	2.70558E-15	0.023976823	1.73857E-26	-2.531246863	0.446587252	2.977834115
1129	RAP1GAP2	Q684P5	RAP1 GTPase activating protein 2		yes	no	no	no	no	yes	3.81525E-11	0.016378131	1.38353E-29	1.3069E-11	0.005357957	1.8493E-30	-3.703573866	1.078545278	4.782119144
1130	DMKN	Q6E0U4	dermokine		no	no	yes	no	yes	no	0.000534048	9.78376E-06	7.24653E-17	0.000375068	3.85676E-07	2.80733E-17	0.774967771	-1.110726938	-1.885694708
1131	LAMTOR1	Q6IAA8	late endosomal/lysosomal adaptor, MAPK and MTOR activator 1		yes	no	no	no	no	yes	3.51955E-09	0.013044124	1.27969E-06	1.55964E-09	0.00398357	8.23941E-07	-2.060480959	-0.506947623	1.553533335
1132	KRT80	Q6KB66	keratin 80		no	no	no	yes	no	yes	0.52705051	0.00058678	2.69776E-05	0.499109908	8.53449E-05	1.88855E-05	-0.136066195	1.085377273	1.221443468
1133	JMJD6	Q6NYC1	jumonji domain containing 6, arginine demethylase and lysine hydroxylase		yes	no	no	no	no	yes	1.953E-05	0.304775604	1.44304E-18	1.19728E-05	0.205899604	5.17848E-19	-1.641504255	0.395139939	2.036644194
1134	CC2D1A	Q6P1N0	coiled-coil and C2 domain containing 1A		yes	no	no	no	no	yes	3.27046E-09	0.900179739	4.14864E-19	1.44185E-09	0.856537133	1.45306E-19	-1.922234538	-0.042153752	1.880080786
1135	EDC4	Q6P2E9	enhancer of mRNA decapping 4		yes	no	no	no	no	yes	1.14178E-08	0.028717832	4.71241E-13	5.31312E-09	0.010722018	2.17792E-13	-1.26735677	0.544045146	1.811401915

1136	PGBD2	Q6P3X8	piggyBac transposable element derived 2		no	yes	no	no	yes	no	2.96866E-08	0.030732962	3.44055E-13	1.44061E-08	0.011627559	1.57763E-13	1.819768206	-0.606677665	-2.426445871
1137	NOTUM	Q6P988	notum, palmitoleoyl-protein carboxylesterase		no	yes	no	no	yes	no	9.44686E-06	0.888152273	9.11761E-10	5.63302E-06	0.838251557	4.8955E-10	1.07597484	-0.038674684	-1.114649524
1138	LARP1	Q6PKG0	La ribonucleoprotein 1, translational regulator		yes	no	no	no	no	yes	8.4337E-15	0.371566539	6.94408E-23	1.02412E-15	0.26415401	1.91615E-23	-2.581921833	0.200019459	2.781941292
1139	APOA5	Q6Q788	apolipoprotein A5		no	yes	no	no	yes	no	3.99872E-10	0.634397225	1.04064E-10	1.59079E-10	0.534940297	5.37059E-11	2.029018082	0.112708396	-1.916309686
1140	OLFML1	Q6UWY5	olfactomedin like 1		no	yes	no	no	yes	no	2.18385E-12	0.276363367	5.41195E-18	5.72927E-13	0.182196964	1.9789E-18	2.058384401	0.247292566	-1.811091835
1141	VIT	Q6UXI7	vitrin		no	yes	no	no	yes	no	8.21178E-11	0.077380789	5.35578E-12	2.96919E-11	0.036458674	2.59659E-12	1.980605388	0.474236873	-1.506368515
1142	PACS1	Q6VY07	phosphofurin acidic cluster sorting protein 1		yes	no	no	no	no	yes	2.97543E-15	0.187853094	1.63008E-28	3.0334E-16	0.11192878	2.526E-29	-3.644992923	0.395550652	4.040543575
1143	CD109	Q6YHK3	CD109 molecule		yes	no	no	no	no	yes	7.01708E-14	0.209337098	1.25118E-16	1.19866E-14	0.128428831	4.89814E-17	-2.460080437	0.252364922	2.712445358
1144	VPS13C	Q709C8	vacuolar protein sorting 13 homolog C		yes	no	no	no	no	yes	1.36662E-15	0.068924363	5.53729E-34	1.15175E-16	0.031815124	3.01076E-35	-2.832170537	0.41484999	3.247020528
1145	UNC13D	Q70J99	unc-13 homolog D		yes	no	no	no	no	yes	5.9314E-16	0.748724506	2.33385E-17	4.02676E-17	0.665947533	8.82994E-18	-3.806394442	0.150662214	3.957056656
1146	CCDC80	Q76M96	coiled-coil domain containing 80		no	yes	no	no	yes	no	2.71582E-07	0.245842537	4.3042E-12	1.43851E-07	0.157062971	2.06921E-12	1.100055978	-0.222368883	-1.322424861
1147	SND1	Q7KZF4	staphylococcal nuclease and tudor domain containing 1		yes	no	no	no	no	yes	5.90038E-15	0.034849404	2.51513E-29	6.84412E-16	0.013611321	3.52142E-30	-3.264839413	0.640171382	3.905010795
1148	MARK2	Q7KZI7	microtubule affinity regulating kinase 2	Fostamatinib	yes	no	no	no	no	yes	1.5469E-12	0.000963488	2.26882E-22	3.89704E-13	0.000164583	6.44562E-23	-1.318997359	0.575837464	1.894834823
1149	BZW1	Q7L1Q6	basic leucine zipper and W2 domains 1		yes	no	no	no	no	yes	1.0044E-13	0.860086593	2.85066E-25	1.80219E-14	0.802409739	6.56578E-26	-3.74021807	-0.072575219	3.667642851
1150	EIF3M	Q7L2H7	eukaryotic translation initiation factor 3 subunit M		yes	no	no	no	no	yes	3.8535E-14	0.382485988	3.86134E-25	6.11744E-15	0.27487293	9.04537E-26	-3.321700012	-0.272265766	3.049434246
1151	CYFIP1	Q7L576	cytoplasmic FMR1 interacting protein 1		yes	no	no	no	no	yes	2.51175E-13	0.044104517	1.17199E-37	5.04171E-14	0.018585048	2.07207E-39	-4.373266381	0.787001999	5.16026838
1152	TAOK1	Q7L7X3	TAO kinase 1	Fostamatinib	yes	no	no	no	no	yes	1.42092E-15	0.027236765	2.1106E-32	1.21039E-16	0.010095004	1.78832E-33	-4.376397788	0.756241558	5.132639345
1153	MOB1B	Q7L9L4	MOB kinase activator 1B		yes	no	no	no	no	yes	5.2229E-16	0.022003663	7.15628E-28	3.43145E-17	0.007756615	1.21271E-28	-2.949979385	0.545463538	3.495442923
1154	CHMP1B	Q7LBR1	charged multivesicular body protein 1B		yes	no	no	no	no	yes	2.3769E-09	0.747270911	5.00571E-20	1.02959E-09	0.662623096	1.66706E-20	-1.900697353	-0.097537895	1.803159458
1155	MICAL3	Q7RTP6	microtubule associated monooxygenase, calponin and LIM domain containing 3		yes	no	no	no	no	yes	2.89649E-09	0.279487948	1.30249E-10	1.26648E-09	0.184683448	6.73966E-11	-1.792034385	-0.329010651	1.463023733
1156	ZC3HAV1	Q7Z2W4	zinc finger CCCH-type containing, antiviral 1		yes	no	no	no	no	yes	7.52667E-12	0.776321826	1.6633E-27	2.22697E-12	0.700342889	2.93922E-28	-2.588764286	0.089855439	2.678619725
1157	UBE3B	Q7Z3V4	ubiquitin protein ligase E3B		no	yes	no	no	yes	no	9.29321E-18	0.488060598	1.1451E-17	1.88595E-19	0.37837412	4.24417E-18	2.46094702	0.166706012	-2.294241007
1158	KRT28	Q7Z3Y7	keratin 28		no	yes	no	no	yes	no	3.90662E-06	0.536955533	1.01559E-10	2.25865E-06	0.431681981	5.2367E-11	2.271883739	0.30938381	-1.962499929
1159	CLASP1	Q7Z460	cytoplasmic linker associated protein 1	Fostamatinib	yes	no	no	no	no	yes	6.00545E-18	0.4112761	3.51554E-18	9.73154E-20	0.304497122	1.2791E-18	-2.918809497	0.250902381	3.169711878
1160	DHX29	Q7Z478	DExH-box helicase 29		yes	no	no	no	no	yes	1.46106E-13	0.003912456	8.92299E-34	2.75397E-14	0.000882829	5.29638E-35	-3.506128502	0.967347083	4.473475584
1161	DCXR	Q7Z4W1	dicarbonyl and L-xylulose reductase	Dihydrogenphosphate; Nicotinamide adenine dinucleotide phosphate	yes	no	no	no	no	yes	5.75325E-12	0.004855274	7.13901E-18	1.65012E-12	0.00116597	2.62335E-18	-2.159737075	0.645435901	2.805172976
1162	VMO1	Q7Z5L0	vitelline membrane outer layer 1 homolog		no	yes	no	no	yes	no	2.44979E-10	0.149253923	7.86511E-08	9.50167E-11	0.084128627	4.73973E-08	1.325180668	0.310114815	-1.015065853
1163	PODN	Q7Z5L7	podocan		no	yes	no	no	yes	no	8.35521E-12	0.010157132	1.01089E-10	2.49861E-12	0.002911172	5.20788E-11	1.90603285	0.574380498	-1.331652352
1164	HUWE1	Q7Z6Z7	HECT, UBA and WWE domain containing E3 ubiquitin protein ligase 1		yes	no	no	no	no	yes	7.92706E-07	0.940656356	5.38703E-12	4.3712E-07	0.914100533	2.61419E-12	-1.598023764	0.029866963	1.627890727
1165	KRT77	Q7Z794	keratin 77		no	yes	no	no	yes	no	7.36686E-07	0.032510805	3.44282E-05	4.04893E-07	0.012565345	2.42417E-05	2.541633078	1.067991705	-1.473641373
1166	PXK	Q7Z7A4	PX domain containing serine/threonine kinase like		yes	no	no	no	no	yes	1.41298E-10	0.064289739	2.63738E-22	5.24986E-11	0.029304702	7.51658E-23	-2.507025388	0.608738407	3.115763795
1167	ADGRG6	Q86SQ4	adhesion G protein-coupled receptor G6		no	yes	no	no	yes	no	2.12518E-07	0.36906996	8.12205E-09	1.11218E-07	0.262044688	4.59281E-09	1.209169994	0.190504496	-1.018665498
1168	MTDH	Q86UE4	metadherin		yes	no	no	no	no	yes	1.77632E-19	0.893634071	8.16937E-24	1.1268E-21	0.846664632	2.121E-24	-3.382183633	0.033882234	3.416065867
1169	FERMT3	Q86UX7	FERM domain containing kindlin 3		yes	no	no	no	no	yes	4.49612E-10	0.039221411	1.50215E-35	1.80497E-10	0.0160016	5.44505E-37	-3.946021191	0.999760868	4.945782059
1170	VPS36	Q86VN1	vacuolar protein sorting 36 homolog		yes	no	no	no	no	yes	2.56115E-13	0.167503348	1.09059E-25	5.17568E-14	0.097147388	2.4164E-26	-1.590463772	0.233710849	1.824174621
1171	CAND1	Q86VP6	cullin associated and neddylation dissociated 1		yes	no	no	no	no	yes	5.14345E-08	0.1224058	2.64014E-24	2.54492E-08	0.065889168	6.67512E-25	-1.904517552	0.481383312	2.385900864
1172	PKHD1L1	Q86WI1	PKHD1 like 1		yes	no	no	no	no	yes	0.000136661	0.040117546	1.15803E-13	9.05297E-05	0.016541444	5.19459E-14	-1.424187549	0.832877621	2.25706517
1173	CHSY1	Q86X52	chondroitin sulfate synthase 1		no	yes	no	no	yes	no	0.001433581	0.98125015	1.34967E-08	0.001057485	0.973247203	7.78489E-09	1.058906971	-0.010163803	-1.069070773
1174	STX12	Q86Y82	syntaxin 12		yes	no	no	no	no	yes	1.75005E-12	0.000638836	1.3869E-09	4.45639E-13	9.55215E-05	7.52833E-10	-2.043995922	-0.754220201	1.289775722
1175	TDRP	Q86YL5	testis development related protein		yes	no	no	no	no	yes	2.94705E-14	0.18514256	8.60976E-34	4.47249E-15	0.109810427	5.07145E-35	-4.183400297	0.490156597	4.673556894
1176	MIB1	Q86YT6	MIB E3 ubiquitin protein ligase 1		yes	no	no	no	no	yes	4.34393E-15	0.332763558	1.89985E-15	4.72381E-16	0.229632487	7.8852E-16	-1.549644541	0.201333429	1.750977971
1177	HRNR	Q86YZ3	hornerin	Zinc; Zinc acetate	no	yes	no	no	yes	no	0.00212468	0.762464994	0.000123694	0.001588455	0.680946719	9.02904E-05	1.316738366	0.15260904	-1.164129326
1178	H2AC21	Q8IUE6	H2A clustered histone 21		no	no	no	yes	no	yes	0.535157946	0.002244568	1.35821E-05	0.508484917	0.000450759	9.31732E-06	-0.342643412	1.580087683	1.922731095
1179	SLC44A2	Q8IWA5	solute carrier family 44 member 2 (CTL2 blood group)		yes	no	no	no	no	yes	1.78372E-13	0.015557539	1.52555E-19	3.45914E-14	0.004990819	5.2119E-20	-1.838837264	0.497546825	2.336384089
1180	SULF2	Q8IWU5	sulfatase 2		no	yes	no	yes	no	no	5.84423E-08	0.00072251	2.62872E-07	2.90755E-08	0.000114253	1.62821E-07	1.864377843	1.100457747	-0.763920096
1181	FAM20C	Q8IXL6	FAM20C golgi associated secretory pathway kinase		no	yes	no	no	yes	no	2.43349E-07	0.885753879	4.6732E-12	1.28125E-07	0.833981224	2.25084E-12	1.138033874	-0.037229981	-1.175263855
1182	GSPT2	Q8IYD1	G1 to S phase transition 2		yes	no	no	no	no	yes	9.52909E-15	0.045952148	1.07436E-26	1.17441E-15	0.019592647	2.11756E-27	-3.411841999	0.622113154	4.033955153
1183	CPNE9	Q8IYJ1	copine family member 9		yes	no	no	no	no	yes	1.06238E-13	0.563871979	1.20787E-13	1.92548E-14	0.459354067	5.42363E-14	-2.409702509	0.21000269	2.619705199
1184	KIAA0319L	Q8IZA0	KIAA0319 like		no	yes	no	no	yes	no	3.29268E-08	0.300444223	1.15974E-07	1.6068E-08	0.202020492	7.06774E-08	1.433402617	0.190782253	-1.242620364
1185	ABI1	Q8IZP0	abl interactor 1		yes	no	no	no	no	yes	4.06546E-13	0.030281744	3.8994E-37	8.67618E-14	0.011415682	8.12743E-39	-4.128076844	0.832026302	4.960103146
1186	RPTOR	Q8N122	regulatory associated protein of MTOR complex 1		no	yes	no	no	yes	no	7.22084E-08	0.148442953	7.81469E-07	3.64477E-08	0.083469735	4.96783E-07	1.430274988	0.372051103	-1.058223885
1187	LRRC47	Q8N1G4	leucine rich repeat containing 47		yes	no	no	no	no	yes	3.8535E-14	0.85577175	5.23184E-28	6.12858E-15	0.796057727	8.67627E-29	-3.646580034	0.070706749	3.717286783
1188	KRT78	Q8N1N4	keratin 78		no	yes	no	no	yes	no	1.10641E-05	0.169415787	0.000230308	6.64249E-06	0.098793891	0.000171036	1.673934073	0.555065904	-1.118868169
1189	MINK1	Q8N4C8	misshapen like kinase 1	Fostamatinib	yes	no	no	no	no	yes	1.52211E-14	0.430028238	8.68648E-20	2.05523E-15	0.321790756	2.92829E-20	-3.023987168	-0.254858589	2.769128579
1190	MYCT1	Q8N699	MYC target 1		yes	no	no	no	no	yes	2.37776E-11	0.011245008	8.47874E-26	7.76042E-12	0.003311851	1.85172E-26	-3.090426378	0.942232673	4.032659051
1191	OTUD6B	Q8N6M0	OTU deubiquitinase 6B		yes	no	no	no	no	yes	3.85523E-12	0.707767488	5.99567E-23	1.06032E-12	0.614125392	1.64358E-23	-2.478845107	-0.109990527	2.36885458
1192	LRRC57	Q8N9N7	leucine rich repeat containing 57		yes	no	no	no	no	yes	4.90202E-17	0.002775184	1.04663E-24	1.88796E-18	0.000575372	2.55611E-25	-2.042794979	0.593670828	2.636465807
1193	MLKL	Q8NB16	mixed lineage kinase domain like pseudokinase		yes	no	no	no	no	yes	1.2996E-14	0.564235179	1.19584E-14	1.71945E-15	0.460081153	5.09871E-15	-2.199324764	-0.156625854	2.04269891
1194	SLC43A3	Q8NBI5	solute carrier family 43 member 3		yes	no	no	no	no	yes	1.7015E-08	0.14360441	1.38882E-06	8.05648E-09	0.08029354	8.99215E-07	-1.402213562	-0.354574305	1.047639258
1195	TXNDC5	Q8NBS9	thioredoxin domain containing 5		yes	no	no	no	no	yes	3.88014E-07	3.7498E-05	1.06801E-12	2.08687E-07	2.36168E-06	5.02794E-13	-1.311055755	0.75519278	2.066248535
1196	SERBP1	Q8NC51	SERPINE1 mRNA binding protein 1		yes	no	no	no	no	yes	4.52796E-12	0.004015839	8.4174E-12	1.26791E-12	0.000915255	4.12288E-12	-2.495427813	-0.869457558	1.625970255
1197	FAM98A	Q8NCA5	family with sequence similarity 98 member A		yes	no	no	no	no	yes	1.73907E-13	0.36906996	8.57231E-17	3.36468E-14	0.26189346	3.34036E-17	-2.334644758	0.276756213	2.611400971
1198	DIPK2A	Q8NDZ4	divergent protein kinase domain 2A		yes	no	no	no	no	yes	1.7036E-05	0.39492847	1.82328E-10	1.03745E-05	0.287692916	9.50054E-11	-1.081461284	0.248759104	1.330220388
1199	TMEM104	Q8NE00	transmembrane protein 104		yes	no	no	no	no	yes	2.40878E-10	0.045963051	6.25697E-16	9.28803E-11	0.019618121	2.53737E-16	-2.969458602	0.713465293	3.682923895
1200	ABCF1	Q8NE71	ATP binding cassette subfamily F member 1		yes	no	no	no	no	yes	1.07096E-14	0.900212104	4.99406E-26	1.35614E-15	0.857856233	1.05448E-26	-3.333509951	0.042069463	3.375579414
1201	REPS2	Q8NFH8	RALBP1 associated Eps domain containing 2		yes	no	no	no	no	yes	3.8308E-19	0.467332715	2.00022E-14	2.95077E-21	0.357646106	8.60992E-15	-3.033313858	-0.234700875	2.798612983
1202	FDCSP	Q8NFU4	follicular dendritic cell secreted protein		no	yes	no	no	yes	no	0.001156743	0.214059668	5.98576E-11	0.000842793	0.131908087	3.04848E-11	1.3598644	-0.53997681	-1.89984121
1203	TRIM58	Q8NG06	tripartite motif containing 58		yes	no	no	no	no	yes	4.68586E-15	0.447275592	2.39727E-21	5.20179E-16	0.339459273	7.35366E-22	-2.928732541	-0.213923435	2.714809106
1204	SVIP	Q8NHG7	small VCP interacting protein		yes	no	no	no	no	yes	2.58126E-12	0.258137569	1.79523E-19	6.87711E-13	0.166555459	6.14951E-20	-1.798227663	0.248539083	2.046766746
1205	RIN3	Q8TB24	Ras and Rab interactor 3		yes	no	no	no	no	yes	1.27642E-17	0.776321826	1.74596E-24	3.06526E-19	0.70001092	4.35897E-25	-2.941624829	-0.073623718	2.868001111
1206	NDNF	Q8TB73	neuron derived neurotrophic factor		no	yes	no	no	yes	no	6.07464E-05	0.1261724	5.72013E-10	3.87819E-05	0.06837435	3.0402E-10	1.059523907	-0.386675491	-1.446199398
1207	PIP4K2C	Q8TBX8	phosphatidylinositol-5-phosphate 4-kinase type 2	Fostamatinib	yes	no	no	no	no	yes	1.29573E-15	0.000105309	1.27679E-24	1.06852E-16	9.11376E-06	3.14714E-25	-2.379864266	0.858521338	3.238385604
1208	PLEKHO2	Q8TD55	pleckstrin homology domain containing O2		yes	no	no	no	no	yes	2.99064E-16	0.001152852	5.70645E-30	1.72094E-17	0.000203199	7.21386E-31	-2.985256609	0.811298013	3.796554622
1209	TRAF3IP1	Q8TDR0	TRAF3 interacting protein 1		no	yes	no	no	yes	no	3.52334E-08	0.258486306	1.28484E-15	1.72575E-08	0.16735241	5.28607E-16	1.435849969	-0.272202141	-1.70805211
1210	NEK7	Q8TDX7	NIMA related kinase 7		yes	no	no	no	no	yes	2.23405E-20	0.116323248	6.5112E-12	5.72909E-23	0.06198634	3.16562E-12	-3.073232174	-0.533777098	2.539455076
1211	DTD1	Q8TEA8	D-aminoacyl-tRNA deacylase 1		yes	no	no	no	no	yes	2.46632E-08	0.775636043	1.77991E-25	1.18679E-08	0.698318449	4.00822E-26	-2.904756023	-0.138721631	2.766034392
1212	PARD3	Q8TEW0	par-3 family cell polarity regulator		yes	no	no	no	no	yes	4.5028E-14	0.071977674	5.02429E-15	7.28364E-15	0.033428688	2.12855E-15	-1.729275429	0.43317738	2.162452809
1213	UBASH3B	Q8TF42	ubiquitin associated and SH3 domain containing B		yes	no	no	no	no	yes	6.21846E-16	0.074132757	8.8911E-20	4.33911E-17	0.034664705	3.00533E-20	-3.794534854	0.742890305	4.537425159
1214	PDCD6IP	Q8WUM4	programmed cell death 6 interacting protein		yes	no	no	no	no	yes	9.73539E-08	0.659815867	4.48181E-21	4.95371E-08	0.56175533	1.39714E-21	-2.336381954	-0.182571153	2.153810801
1215	BRK1	Q8WUW1	BRICK1 subunit of SCAR/WAVE actin nucleating		yes	no	no	no	no	yes	1.02794E-15	0.042064014	8.64705E-37	8.08366E-17	0.017562371	2.19409E-38	-4.337609744	0.672036107	5.009645851
1216	SCFD1	Q8WVM8	sec1 family domain containing 1		yes	no	no	no	no	yes	8.72028E-16	0.005040574	1.20274E-19	6.51947E-17	0.001215036	4.09269E-20	-2.426746619	0.820854058	3.247600676
1217	PCNP	Q8WW12	PEST proteolytic signal containing nuclear protein		yes	no	no	no	no	yes	2.70585E-11	0.983814923	8.25819E-21	8.96228E-12	0.977441343	2.63798E-21	-1.995202765	-0.005664956	1.989537809
1218	SLC44A1	Q8WWI5	solute carrier family 44 member 1		yes	no	no	no	no	yes	8.88281E-10	0.303638786	8.30577E-24	3.6626E-10	0.204856435	2.16394E-24	-2.865133167	0.409014971	3.274148138
1219	OIT3	Q8WWZ8	oncoprotein induced transcript 3		yes	no	no	no	no	yes	7.02526E-05	0.82959387	2.98254E-05	4.52965E-05	0.764564537	2.09332E-05	-1.154373967	-0.055122459	1.099251508
1220	DNAJC9	Q8WXX5	DnaJ heat shock protein family (Hsp40) member C9		yes	no	no	no	no	yes	7.02171E-11	0.415009781	5.79563E-14	2.51662E-11	0.308013603	2.563E-14	-1.033288931	0.131696406	1.164985337
1221	LZIC	Q8WZA0	leucine zipper and CTNNBIP1 domain containing		yes	no	no	no	no	yes	4.44558E-16	0.000264757	9.83301E-26	2.79989E-17	3.03473E-05	2.1564E-26	-1.761080352	0.677114054	2.438194406
1222	DDX1	Q92499	DEAD-box helicase 1		yes	no	no	no	no	yes	1.67058E-13	0.594596929	5.43508E-26	3.20945E-14	0.491680741	1.15252E-26	-3.203473462	-0.165947287	3.037526175
1223	LPIN2	Q92539	lipin 2		yes	no	no	no	no	yes	4.86407E-06	0.053981363	1.1598E-11	2.84307E-06	0.023994434	5.70705E-12	-1.050032881	0.434853592	1.484886473
1224	ELMO1	Q92556	engulfment and cell motility 1		yes	no	no	no	no	yes	4.20501E-16	0.480723822	2.19808E-27	2.61027E-17	0.370742574	3.96391E-28	-3.075863343	-0.17736708	2.898496264
1225	AP3S1	Q92572	adaptor related protein complex 3 subunit sigma 1		yes	no	no	no	no	yes	1.01624E-17	0.150790454	2.26143E-26	2.18848E-19	0.085260672	4.61098E-27	-3.666626986	-0.372324236	3.294302751
1226	DOCK2	Q92608	dedicator of cytokinesis 2		yes	no	no	no	no	yes	1.02679E-11	0.04896443	2.08257E-12	3.14505E-12	0.021276343	9.91743E-13	-1.6825645	-0.381944797	1.300619704
1227	MYO18A	Q92614	myosin XVIIIA		yes	no	no	no	no	yes	1.60624E-14	0.970357442	2.19808E-27	2.1761E-15	0.956287919	3.95667E-28	-3.030206135	-0.011925968	3.018280167
1228	ARHGAP45	Q92619	Rho GTPase activating protein 45		yes	no	no	no	no	yes	1.278E-15	0.007822333	7.65339E-38	1.04811E-16	0.002073381	1.14437E-39	-4.345181546	0.894141194	5.23932274
1229	NUP205	Q92621	nucleoporin 205		no	yes	no	no	yes	no	1.96287E-05	0.835605363	3.47781E-06	1.20422E-05	0.772376502	2.29595E-06	1.573312519	0.071181532	-1.502130987
1230	PXDN	Q92626	peroxidasin		no	yes	no	no	yes	no	2.42885E-08	0.124359671	9.04135E-09	1.16655E-08	0.067279314	5.13722E-09	1.355930209	0.305183765	-1.050746445
1231	TFG	Q92734	trafficking from ER to golgi regulator		yes	no	no	no	no	yes	5.68254E-15	0.592627953	1.40754E-15	6.51419E-16	0.489651925	5.80361E-16	-1.873448564	0.134551979	2.008000544
1232	HTRA1	Q92743	HtrA serine peptidase 1		no	yes	no	no	yes	no	2.68116E-11	0.11419554	2.45283E-15	8.86835E-12	0.060445246	1.02581E-15	2.320441851	0.430146774	-1.890295077
1233	TAF15	Q92804	TATA-box binding protein associated factor 15		yes	no	no	no	no	yes	1.09986E-11	0.053343978	1.26545E-12	3.38382E-12	0.02366278	5.9918E-13	-2.524235942	-0.62458732	1.899648622

1234	INPP5D	Q92835	inositol polyphosphate-5-phosphatase D		yes	no	no	no	no	yes	5.97255E-17	0.14742208	1.56552E-23	2.46263E-18	0.082828899	4.14967E-24	-2.106149451	0.288802051	2.394951502
1235	RAD50	Q92878	RAD50 double strand break repair protein		no	yes	no	yes	no	no	0.000131877	0.000110938	0.115680048	8.73008E-05	9.91638E-06	0.103834244	1.214303374	1.545951602	0.331648228
1236	OSTF1	Q92882	osteoclast stimulating factor 1		yes	no	no	no	no	yes	7.14695E-09	0.76853712	2.79022E-16	3.27393E-09	0.688398108	1.10749E-16	-2.434027074	-0.126893607	2.307133467
1237	ARHGEF1	Q92888	Rho guanine nucleotide exchange factor 1		yes	no	no	no	no	yes	2.34792E-14	0.131435368	2.25141E-21	3.42283E-15	0.07206017	6.87561E-22	-2.327766143	0.315111156	2.642877299
1238	UFD1	Q92890	ubiquitin recognition factor in ER associated degradation		yes	no	no	no	no	yes	8.23633E-15	0.039221411	3.67177E-29	9.92689E-16	0.016041709	5.20737E-30	-2.862360788	0.493870141	3.356230929
1239	RAB8B	Q92930	RAB8B, member RAS oncogene family	Phosphoaminophosphonic acid guanylate ester; Guanosine-5'-Diphosphate	yes	no	no	no	no	yes	4.57112E-14	0.04550327	9.31295E-33	7.43558E-15	0.019318788	7.21574E-34	-2.925876069	0.536812499	3.462688568
1240	PRG4	Q92954	proteoglycan 4		no	yes	no	no	yes	no	0.001182553	0.548207615	6.72529E-10	0.000862669	0.443384954	3.59881E-10	1.378364159	-0.263378367	-1.641742526
1241	USP9X	Q93008	ubiquitin specific peptidase 9 X-linked		yes	no	no	no	no	yes	4.78737E-09	0.022880555	1.20294E-25	2.14532E-09	0.008107201	2.67078E-26	-1.776464562	0.610058167	2.386522729
1242	CUL5	Q93034	cullin 5		yes	no	no	no	no	yes	2.68099E-05	0.196165807	7.86961E-18	1.66544E-05	0.117770591	2.89895E-18	-1.510466752	0.487275341	1.997742094
1243	ATP2A3	Q93084	ATPase sarcoplasmic/endoplasmic reticulum Ca2+ transporting 3		yes	no	no	no	no	yes	1.05461E-05	0.529338235	8.42805E-07	6.3076E-06	0.422846991	5.36539E-07	-2.391661518	0.333592593	2.725254111
1244	WBP2	Q969T9	WW domain binding protein 2		yes	no	no	no	no	yes	2.2645E-15	0.04453981	8.38252E-22	2.14445E-16	0.018808837	2.48399E-22	-2.660374843	-0.476839634	2.183535209
1245	TMBIM1	Q969X1	transmembrane BAX inhibitor motif containing 1		yes	no	no	no	no	yes	7.13573E-12	0.021123627	4.64118E-10	2.08866E-12	0.007331535	2.45623E-10	-2.643391466	-0.643569943	1.999821522
1246	EXOC4	Q96A65	exocyst complex component 4		yes	no	no	no	no	yes	9.42157E-16	0.086199045	6.2045E-31	7.25401E-17	0.042064509	6.71897E-32	-3.4625977	0.499439308	3.962037008
1247	LRRC59	Q96AG4	leucine rich repeat containing 59		yes	no	no	no	no	yes	2.16197E-15	0.714942308	5.64163E-27	2.02777E-16	0.621322767	1.06084E-27	-4.788135307	0.155245969	4.943381276
1248	ESAM	Q96AP7	endothelial cell adhesion molecule		yes	no	no	no	no	yes	3.26E-15	0.549333819	4.2094E-17	3.36783E-16	0.445042532	1.60976E-17	-2.818317132	0.180636524	2.998953656
1249	VPS33A	Q96AX1	VPS33A core subunit of CORVET and HOPS		no	no	no	yes	no	yes	0.458483636	0.003782757	1.38882E-06	0.431269609	0.000845397	8.98473E-07	0.270637648	1.337443463	1.066805815
1250	AIDA	Q96BJ3	axin interactor, dorsalization associated		yes	no	no	no	no	yes	3.2291E-09	0.615044878	6.98832E-16	1.42215E-09	0.514441706	2.84029E-16	-1.874595814	-0.152882124	1.72171369
1251	ARL8A	Q96BM9	ADP ribosylation factor like GTPase 8A		yes	no	no	no	no	yes	2.39295E-18	0.000640648	3.89069E-25	3.14433E-20	9.69535E-05	9.13176E-26	-3.423512326	0.778419565	4.201931891
1252	DOCK10	Q96BY6	dedicator of cytokinesis 10		yes	no	no	no	no	yes	3.59225E-08	0.116493333	2.64594E-16	1.76113E-08	0.062179042	1.04902E-16	-1.97376608	0.566731069	2.540497149
1253	SYTL4	Q96C24	synaptotagmin like 4	Insulin pork	yes	no	no	no	no	yes	4.82889E-14	0.14478688	7.42256E-31	7.96427E-15	0.081151505	8.20618E-32	-4.325476107	0.590434354	4.91591046
1254	CTHRC1	Q96CG8	collagen triple helix repeat containing 1		no	yes	no	no	yes	no	8.36506E-07	0.059540007	9.25592E-11	4.6203E-07	0.026788957	4.76426E-11	1.273041663	-0.399350785	-1.672392447
1255	CCDC124	Q96CT7	coiled-coil domain containing 124		yes	no	no	no	no	yes	1.71424E-07	0.103944767	2.22661E-13	8.92459E-08	0.053361767	1.0109E-13	-1.862226233	-0.528410397	1.333815836
1256	AP2M1	Q96CW1	adaptor related protein complex 2 subunit mu 1		yes	no	no	no	no	yes	3.35006E-14	0.021955192	2.3223E-33	5.13914E-15	0.007719278	1.57284E-34	-2.836101302	0.576450182	3.412551484
1257	REPS1	Q96D71	RALBP1 associated Eps domain containing 1		yes	no	no	no	no	yes	3.55572E-14	0.093115823	2.09487E-21	5.54223E-15	0.046375923	6.38808E-22	-2.310273012	0.380092956	2.690365967
1258	MRPL38	Q96DV4	mitochondrial ribosomal protein L38		yes	no	no	no	no	yes	2.68085E-09	0.935275385	4.96791E-20	1.16733E-09	0.905616056	1.65222E-20	-2.468349151	-0.03184557	2.436503581
1259	DAZAP1	Q96EP5	DAZ associated protein 1		yes	no	no	no	no	yes	1.51922E-19	0.72764738	1.13023E-23	8.94874E-22	0.640161242	2.97537E-24	-2.955305541	0.083609498	3.03891504
1260	CHMP6	Q96FZ7	charged multivesicular body protein 6		yes	no	no	no	no	yes	8.28735E-12	0.020781146	4.8913E-07	2.47081E-12	0.007127924	3.08504E-07	-2.34353488	-0.838240957	1.505293923
1261	DCUN1D1	Q96GG9	defective in cullin neddylation 1 domain containing 1		yes	no	no	no	no	yes	9.70907E-10	0.815418742	1.64233E-27	4.02528E-10	0.746697452	2.88728E-28	-2.080000461	0.071335533	2.151335994
1262	PDLIM5	Q96HC4	PDZ and LIM domain 5		yes	no	no	no	no	yes	4.67806E-15	0.087986218	6.07899E-20	5.15074E-16	0.043096104	2.03276E-20	-2.996922336	0.585551777	3.582474113
1263	DDRGK1	Q96HY6	DDRGK domain containing 1		yes	no	no	no	no	yes	6.00721E-16	4.99255E-05	2.53451E-25	4.13727E-17	3.52894E-06	5.76494E-26	-2.072552215	0.776175653	2.848727867
1264	SUCLG2	Q96I99	succinate-CoA ligase GDP-forming subunit beta	Phenethyl Isothiocyanate	no	no	no	yes	no	yes	0.020834839	2.01997E-07	4.48986E-07	0.0170115	2.56272E-09	2.82661E-07	1.301821778	3.97712091	2.675299132
1265	CPB2	Q96IY4	carboxypeptidase B2	AZD-9684; Prothrombin	no	yes	no	no	yes	no	2.26204E-11	0.724817221	9.43952E-16	7.35906E-12	0.636472938	3.8622E-16	1.335518809	-0.061714007	-1.397232816
1266	ELMO2	Q96JJ3	engulfment and cell motility 2		yes	no	no	no	no	yes	2.08379E-17	0.085172931	4.18186E-27	6.57723E-19	0.041255035	7.73086E-28	-2.455989846	0.346485734	2.80247558
1267	BTF3L4	Q96K17	basic transcription factor 3 like 4		yes	no	no	no	no	yes	1.07028E-11	0.951427404	5.6069E-22	3.28309E-12	0.928775834	1.63101E-22	-2.676950302	-0.022720285	2.654230017
1268	USP47	Q96K76	ubiquitin specific peptidase 47		yes	no	no	no	no	yes	1.84613E-17	0.000168341	4.74505E-26	5.2281E-19	1.69332E-05	9.9975E-27	-2.450309785	0.911484942	3.361794727
1269	EXOC2	Q96KP1	exocyst complex component 2		yes	no	no	no	no	yes	9.42157E-16	0.359205314	8.26471E-21	7.25721E-17	0.252924811	2.64405E-21	-3.952818182	-0.353248843	3.599569339
1270	CNDP2	Q96KP4	carnosine dipeptidase 2		yes	no	no	no	no	yes	9.41955E-14	0.244933163	1.91578E-30	1.6688E-14	0.156260033	2.24825E-31	-3.91642882	0.41274368	4.329172499
1271	PRRC1	Q96M27	proline rich coiled-coil 1		yes	no	no	no	no	yes	3.73743E-16	0.950128877	7.7221E-16	2.23535E-17	0.926881501	3.14552E-16	-1.879264433	-0.015500777	1.863763655
1272	CCDC43	Q96MW1	coiled-coil domain containing 43		yes	no	no	no	no	yes	1.02027E-15	0.250815425	1.67692E-28	7.95134E-17	0.161035549	2.6158E-29	-3.084766974	0.299229324	3.383996297
1273	PRAP1	Q96NZ9	proline rich acidic protein 1		no	yes	no	no	yes	no	3.78372E-08	0.164709178	4.66429E-13	1.86014E-08	0.095153694	2.15356E-13	1.476848199	-0.296977533	-1.773825732
1274	ARAP1	Q96P48	ArfGAP with RhoGAP domain, ankyrin repeat and PH domain 1		yes	no	no	no	no	yes	1.03803E-18	0.00067844	9.33762E-30	1.05275E-20	0.000105747	1.21427E-30	-2.858681577	0.723664117	3.582345694
1275	SERPINB12	Q96P63	serpin family B member 12		no	yes	no	no	yes	no	1.43159E-07	0.033486351	3.66502E-07	7.42068E-08	0.013018255	2.29333E-07	1.727523181	0.666853306	-1.060669876
1276	ERMAP	Q96PL5	erythroblast membrane associated protein (Scianna blood group)		no	no	yes	no	yes	no	0.008090846	1.05001E-05	1.76847E-16	0.006375161	4.37702E-07	6.96332E-17	0.589778096	-1.241027028	-1.830805124
1277	VPS35	Q96QK1	VPS35 retromer complex component		yes	no	no	no	no	yes	2.48099E-08	0.966275174	1.24694E-09	1.19609E-08	0.94920008	6.74039E-10	-2.344529953	0.025113752	2.369643705
1278	SNX18	Q96RF0	sorting nexin 18		yes	no	no	no	no	yes	1.03803E-18	0.000116793	8.87962E-28	1.02152E-20	1.06897E-05	1.53291E-28	-2.92760009	0.922670163	3.850270253
1279	F2RL3	Q96RI0	F2R like thrombin or trypsin receptor 3	Thrombin	yes	no	no	no	no	yes	3.54337E-14	0.249833813	1.66321E-18	5.49085E-15	0.160178906	5.98362E-19	-2.727216232	0.309408324	3.036624556
1280	ERBIN	Q96RT1	erbb2 interacting protein		yes	no	no	no	no	yes	3.42005E-13	0.040858626	5.98384E-22	7.15932E-14	0.016921062	1.7515E-22	-2.824785844	0.688313266	3.51309911
1281	PEBP4	Q96S96	phosphatidylethanolamine binding protein 4		no	yes	no	no	yes	no	1.74398E-08	0.3964278	1.85474E-19	8.27346E-09	0.289552158	6.36174E-20	1.632372723	-0.213258945	-1.845631668
1282	MYADM	Q96S97	myeloid associated differentiation marker		yes	no	no	no	no	yes	6.55132E-10	0.405614453	1.0661E-14	2.65674E-10	0.299386472	4.53589E-15	-2.982409176	0.342871694	3.32528087
1283	RUFY1	Q96T51	RUN and FYVE domain containing 1		yes	no	no	no	no	yes	7.15194E-10	0.972423879	9.11808E-11	2.91651E-10	0.961310052	4.68918E-11	-1.454721357	-0.009138046	1.445583311
1284	CYTH2	Q99418	cytohesin 2	Inositol 1,3,4,5-Tetrakisphosphate; Formic acid; 1D-myo-inositol 1,4,5-trisphosphate; S,S-(2-Hydroxyethyl)Thiocysteine; Brefeldin	yes	no	no	no	no	yes	7.10225E-18	2.66304E-05	4.85501E-31	1.25504E-19	1.42383E-06	5.16959E-32	-2.487370488	0.853402076	3.340772564
1285	CNN2	Q99439	calponin 2		yes	no	no	no	no	yes	1.94846E-12	0.010017148	1.08889E-35	5.03226E-13	0.00285491	3.79904E-37	-4.043898594	1.07587461	5.119773204
1286	CDC5L	Q99459	cell division cycle 5 like		no	yes	no	yes	no	no	0.004700708	0.000847234	0.305966017	0.003629364	0.000137815	0.285032229	1.211993172	1.588596581	0.376603409
1287	PSMD1	Q99460	proteasome 26S subunit, non-ATPase 1		yes	no	no	no	no	yes	9.65239E-12	0.01127807	5.09601E-19	2.93027E-12	0.003326699	1.7918E-19	-1.691856416	0.611837739	2.303694155
1288	AGAP2	Q99490	ArfGAP with GTPase domain, ankyrin repeat and PH domain 2		yes	no	no	no	no	yes	2.178E-17	0.857436171	6.31749E-21	7.00671E-19	0.798383023	1.98942E-21	-2.491314643	-0.040751143	2.4505635
1289	PARK7	Q99497	Parkinsonism associated deglycase	Copper	yes	no	no	no	no	yes	1.32814E-13	0.321300221	1.68442E-24	2.47935E-14	0.219247002	4.18242E-25	-2.977495857	0.318122785	3.295618642
1290	DNAJC2	Q99543	DnaJ heat shock protein family (Hsp40) member C2		yes	no	no	no	no	yes	6.76455E-16	0.458040825	3.50464E-23	4.80872E-17	0.348667234	9.52779E-24	-2.936345214	0.197090529	3.133435743
1291	EIF3C	Q99613	eukaryotic translation initiation factor 3 subunit C		yes	no	no	no	no	yes	2.21231E-14	0.440552339	1.00386E-25	3.18765E-15	0.332563965	2.20605E-26	-3.344951864	-0.240030619	3.104921244
1292	DNAJC7	Q99615	DnaJ heat shock protein family (Hsp40) member C7		yes	no	no	no	no	yes	1.04246E-13	0.13075409	7.07579E-30	1.87992E-14	0.07139043	9.04111E-31	-2.58529907	0.377627381	2.96292645
1293	MAP3K5	Q99683	mitogen-activated protein kinase kinase kinase 5		yes	no	no	no	no	yes	0.000318233	0.971599068	6.63427E-10	0.000218452	0.959712718	3.54709E-10	-1.042797167	0.012670302	1.055467469
1294	MGLL	Q99685	monoglyceride lipase		yes	no	no	no	no	yes	2.40014E-13	0.142979741	1.40819E-28	4.79594E-14	0.079879484	2.14386E-29	-3.947039238	0.544592216	4.491631454
1295	COL12A1	Q99715	collagen type XII alpha 1 chain		no	yes	no	no	yes	no	5.41809E-13	0.72052526	9.26124E-19	1.22011E-13	0.631071739	3.2899E-19	1.816230754	-0.072085938	-1.888316691
1296	SEPTIN5	Q99719	septin 5		yes	no	no	no	no	yes	1.03028E-12	0.038498762	5.77295E-35	2.51152E-13	0.015612321	2.43264E-36	-4.246883413	0.86686563	5.113749043
1297	HNRNPAB	Q99729	heterogeneous nuclear ribonucleoprotein A/B		yes	no	yes	no	no	no	4.95056E-06	9.89791E-05	0.607677442	2.89586E-06	8.38242E-06	0.586751531	-1.197641944	-1.079938781	0.117703163
1298	NAP1L4	Q99733	nucleosome assembly protein 1 like 4		yes	no	no	no	no	yes	3.75812E-12	0.035027158	3.40227E-29	1.0285E-12	0.013696619	4.79432E-30	-2.495546844	0.586808721	3.082355564
1299	NAPG	Q99747	NSF attachment protein gamma		yes	no	no	no	no	yes	9.57263E-15	0.109028847	2.67074E-27	1.18411E-15	0.056564581	4.8647E-28	-2.312356954	0.356617388	2.668974341
1300	SLC29A1	Q99808	solute carrier family 29 member 1 (Augustine blood	Troglitazone; Ethanol; Fostamatinib	no	no	yes	no	yes	no	0.638948164	0.000963637	5.07487E-09	0.616076888	0.000165045	2.83521E-09	0.125475953	-1.077775052	-1.203251005
1301	TSG101	Q99816	tumor susceptibility 101		yes	no	no	no	no	yes	4.90202E-17	0.402716984	6.02897E-27	1.86819E-18	0.296450985	1.13641E-27	-2.335554911	0.166244946	2.501799857
1302	CCT7	Q99832	chaperonin containing TCP1 subunit 7		yes	no	no	no	no	yes	6.77286E-12	0.733472604	3.32912E-31	1.97631E-12	0.647396753	3.46941E-32	-3.537903411	-0.134443118	3.403460294
1303	PTPN18	Q99952	protein tyrosine phosphatase non-receptor type 18		yes	no	no	no	no	yes	3.51056E-19	0.014827884	6.15268E-32	2.54504E-21	0.004716436	5.43622E-33	-4.714435569	0.727023034	5.441458603
1304	SH3GL1	Q99961	SH3 domain containing GRB2 like 1, endophilin A2		yes	no	no	no	no	yes	2.41932E-14	0.024336309	1.43076E-33	3.54074E-15	0.008722257	8.94631E-35	-3.315018115	0.672691631	3.987709747
1305	RARRES2	Q99969	retinoic acid receptor responder 2		no	yes	no	no	yes	no	1.86755E-12	0.858386926	5.49868E-16	4.79792E-13	0.800435112	2.2199E-16	1.821089387	-0.037930549	-1.859019936
1306	GDF15	Q99988	growth differentiation factor 15		no	yes	no	yes	no	no	6.05739E-09	8.65243E-07	0.182251962	2.75286E-09	1.45057E-08	0.167057417	1.560565304	1.308908244	-0.25165706
1307	MICU1	Q9BPX6	mitochondrial calcium uptake 1		yes	no	no	no	no	yes	9.11551E-16	0.009195913	1.15214E-24	6.85625E-17	0.002563181	2.83468E-25	-2.164124065	0.565342396	2.72946646
1308	C7orf25	Q9BPX7	chromosome 7 open reading frame 25		yes	no	no	no	no	yes	1.40737E-06	0.013214108	8.41153E-13	7.90089E-07	0.004071406	3.92945E-13	-1.660670504	1.022439294	2.683109799
1309	VPS25	Q9BRG1	vacuolar protein sorting 25 homolog		yes	no	no	no	no	yes	6.92936E-15	0.018877921	1.98955E-23	8.19467E-16	0.006372475	5.31868E-24	-1.934205545	0.484724422	2.418929966
1310	PYM1	Q9BRP8	PYM homolog 1, exon junction complex associated		yes	no	no	no	no	yes	1.96837E-14	0.873158027	6.8017E-28	2.80942E-15	0.818956554	1.14954E-28	-3.814869806	-0.063004329	3.751865477
1311	ERP44	Q9BS26	endoplasmic reticulum protein 44		yes	no	no	no	no	yes	1.25826E-06	0.004730735	7.17499E-10	7.04669E-07	0.001129632	3.8427E-10	-1.696040439	0.62249262	2.318533059
1312	PI4K2A	Q9BTU6	phosphatidylinositol 4-kinase type 2 alpha		no	no	yes	no	yes	no	0.018230137	1.46389E-07	6.20754E-09	0.014826957	1.59191E-09	3.48207E-09	-0.583339719	-1.756295283	-1.172955564
1313	CHRDL1	Q9BU40	chordin like 1		no	yes	no	no	yes	no	4.84287E-08	0.612826263	9.3142E-08	2.39401E-08	0.511752969	5.6341E-08	1.20242519	0.091492807	-1.110932383
1314	PDCD10	Q9BUL8	programmed cell death 10		yes	no	no	no	no	yes	9.87078E-12	0.560957981	9.1629E-34	3.00551E-12	0.455730703	5.4803E-35	-4.125862885	0.26112654	4.386989425
1315	VAMP8	Q9BV40	vesicle associated membrane protein 8		yes	no	no	no	no	yes	1.52291E-10	0.000453684	3.24544E-14	5.6859E-11	6.02308E-05	1.41611E-14	-2.535379603	0.774913428	3.31029303
1316	C1QTNF3	Q9BXJ4	C1q and TNF related 3		no	yes	no	no	yes	no	0.000101123	0.800011208	1.31858E-08	6.63005E-05	0.727151102	7.59365E-09	1.422516626	-0.096628364	-1.51914499
1317	CFHR5	Q9BXR6	complement factor H related 5		no	yes	no	no	yes	no	6.96748E-09	0.047545313	3.1923E-13	3.18225E-09	0.020545251	1.46091E-13	1.907049953	0.545753899	-1.361296054
1318	AP1M1	Q9BXS5	adaptor related protein complex 1 subunit mu 1		yes	no	no	no	no	yes	3.4143E-17	0.388895555	3.68721E-19	1.22216E-18	0.28104804	1.28476E-19	-2.728670741	-0.210257867	2.518412874
1319	CHMP4A	Q9BY43	charged multivesicular body protein 4A		yes	no	no	no	no	yes	2.06308E-17	0.20601772	1.18813E-21	6.35656E-19	0.125404468	3.57462E-22	-2.312886853	-0.246950253	2.0659366
1320	EIF2A	Q9BY44	eukaryotic translation initiation factor 2A		yes	no	no	no	no	yes	8.41739E-10	0.627499318	1.69072E-24	3.45544E-10	0.527133546	4.20573E-25	-2.565243555	0.177675863	2.742919417
1321	OSBPL8	Q9BZF1	oxysterol binding protein like 8		yes	no	no	no	no	yes	6.54845E-07	0.045543939	2.13371E-18	3.57835E-07	0.019397962	7.71499E-19	-2.044119059	0.825653654	2.869772713
1322	UACA	Q9BZF9	uveal autoantigen with coiled-coil domains and ankyrin repeats		yes	no	no	no	no	yes	1.67224E-11	0.000103097	1.27703E-16	5.37209E-12	8.82823E-06	5.00511E-17	-1.372703735	0.942334454	2.315038189
1323	UBL5	Q9BZL1	ubiquitin like 5		yes	no	no	no	no	yes	2.92623E-07	0.72764738	1.42361E-10	1.55791E-07	0.640606643	7.37928E-11	-1.484990723	0.117595893	1.602586616
1324	PPP1R12C	Q9BZL4	protein phosphatase 1 regulatory subunit 12C		no	no	no	yes	no	yes	2.13327E-05	6.91671E-12	1.1561E-21	1.3136E-05	9.40196E-15	3.47301E-22	-0.921903837	2.48651094	3.408414777
1325	UBE2O	Q9C0C9	ubiquitin conjugating enzyme E2 O		yes	no	no	no	no	yes	2.45371E-11	0.524115762	2.82802E-29	8.04933E-12	0.417389064	3.9723E-30	-2.984844508	0.225593531	3.210438039
1326	PITHD1	Q9GZP4	PITH domain containing 1		yes	no	no	no	no	yes	9.85427E-20	0.005868549	3.86862E-28	4.91151E-22	0.001454507	6.32791E-29	-1.690550009	0.359783246	2.050333256

1327	IMUP	Q9GZP8	chromosome 19 open reading frame 33		yes	no	no	no	no	yes	3.67321E-15	0.045178116	5.55296E-26	3.83011E-16	0.01916027	1.18255E-26	-4.857120681	0.816427152	5.673547833
1328	KLC2	Q9H0B6	kinesin light chain 2		yes	no	no	no	no	yes	9.61146E-16	0.001460636	1.23735E-22	7.44703E-17	0.000264066	3.47601E-23	-2.625933606	0.91806862	3.544002226
1329	RAB1B	Q9H0U4	RAB1B, member RAS oncogene family		yes	no	no	no	no	yes	1.43956E-10	0.031354804	9.91211E-37	5.36165E-11	0.012029929	2.56345E-38	-3.578790837	0.905784033	4.484574871
1330	EHD4	Q9H223	EH domain containing 4		yes	no	no	no	no	yes	2.91696E-12	0.091268139	4.45164E-29	7.86403E-13	0.04499564	6.36201E-30	-3.254470331	0.58419013	3.838660461
1331	VPS16	Q9H269	VPS16 core subunit of CORVET and HOPS complexes		yes	no	no	no	no	yes	7.78737E-16	0.428900782	1.06967E-18	5.64558E-17	0.32065532	3.81437E-19	-2.02860385	-0.142361211	1.886242639
1332	SH3BGRL3	Q9H299	SH3 domain binding glutamate rich protein like 3		yes	no	no	no	no	yes	1.02233E-10	0.088220034	9.51129E-31	3.7382E-11	0.043250601	1.07208E-31	-3.915873509	0.797311324	4.713184833
1333	SLK	Q9H2G2	STE20 like kinase	K-00546; 2-[4-[4-[(5-cyclopropyl-1H-pyrazol-3-yl)amino]quinazolin-2-yl]iminocyclohexa-2,5-dien-1-yl]acetonitrile; Fostamatinib	yes	no	no	no	no	yes	6.31658E-08	0.0487661	2.53407E-31	3.1683E-08	0.021168067	2.53752E-32	-2.548515257	0.807112945	3.355628203
1334	TAOK3	Q9H2K8	TAO kinase 3		yes	no	no	no	no	yes	1.40453E-15	0.122977286	9.72313E-30	1.19006E-16	0.06630855	1.26881E-30	-3.584053673	0.435921882	4.019975555
1335	CRISPLD1	Q9H336	cysteine rich secretory protein LCCL domain containing		yes	no	no	no	no	yes	7.957E-05	0.273452009	6.02207E-13	5.16286E-05	0.179781996	2.79957E-13	-1.15540915	0.355906199	1.511315349
1336	BOLA2	Q9H3K6	bolA family member 2		yes	no	no	no	no	yes	2.54223E-15	0.001563547	9.06812E-13	2.52265E-16	0.000286922	4.2485E-13	-1.980901946	-0.65560803	1.325293916
1337	UNC45A	Q9H3U1	unc-45 myosin chaperone A		yes	no	no	no	no	yes	4.28117E-12	0.059100466	3.39744E-19	1.19493E-12	0.026564414	1.17918E-19	-3.020699241	0.765898221	3.786597462
1338	CHMP4B	Q9H444	charged multivesicular body protein 4B		yes	no	no	no	no	yes	1.47924E-09	0.229492409	2.23457E-17	6.26683E-10	0.143913681	8.44418E-18	-2.631866619	-0.452710964	2.179155655
1339	FN3K	Q9H479	fructosamine 3 kinase		yes	no	no	no	no	yes	3.83548E-15	0.007619353	2.19807E-27	4.03186E-16	0.001995463	3.94398E-28	-2.954398875	0.720533041	3.674931917
1340	WNK1	Q9H4A3	WNK lysine deficient protein kinase 1		yes	no	no	no	no	yes	3.75418E-08	0.85577175	6.71938E-25	1.84392E-08	0.795349921	1.62276E-25	-1.776187583	0.058793478	1.834981061
1341	RNPEP	Q9H4A4	arginyl aminopeptidase		no	yes	no	yes	no	no	0.001123992	0.000217934	0.01945782	0.000816893	2.35938E-05	0.016407342	1.205707978	1.643414416	0.437706438
1342	DEF6	Q9H4E7	DEF6 guanine nucleotide exchange factor		yes	no	yes	no	no	no	8.32373E-11	6.80627E-09	0.776617834	3.01721E-11	4.00913E-11	0.761486631	-1.801110024	-1.765027856	0.036082168
1343	EHD1	Q9H4M9	EH domain containing 1		yes	no	no	no	no	yes	4.41175E-14	0.019886889	1.25511E-35	7.11637E-15	0.006749107	4.49268E-37	-3.21827077	0.664936746	3.883207516
1344	AAMDC	Q9H7C9	adipogenesis associated Mth938 domain containing		yes	no	no	no	no	yes	1.03747E-09	0.042438482	9.07847E-09	4.32476E-10	0.0177484	5.16243E-09	-2.10609285	-0.503744901	1.602347949
1345	PLEKHF2	Q9H8W4	pleckstrin homology and FYVE domain containing 2		yes	no	no	no	no	yes	1.1578E-13	0.291641496	1.73745E-26	2.12989E-14	0.194937639	3.47963E-27	-2.941851042	0.308569167	3.250420209
1346	PSTPIP2	Q9H939	proline-serine-threonine phosphatase interacting protein		yes	no	no	no	no	yes	8.85272E-12	0.175243244	3.74918E-36	2.66745E-12	0.102906771	1.1042E-37	-4.907581761	0.689247732	5.596829493
1347	UPF2	Q9HAU5	UPF2 regulator of nonsense mediated mRNA decay		yes	no	no	no	no	yes	5.7514E-13	0.467709239	6.78701E-19	1.31341E-13	0.358146178	2.40175E-19	-2.38615762	0.220857995	2.607015615
1348	CACYBP	Q9HB71	calcyclin binding protein		yes	no	no	no	no	yes	3.8253E-11	0.706538099	2.98643E-26	1.31207E-11	0.612468916	6.18395E-27	-2.96236663	0.145077584	3.107444214
1349	PARVB	Q9HBI1	parvin beta		yes	no	no	no	no	yes	3.68382E-10	0.058783791	7.15056E-36	1.45884E-10	0.026368805	2.26796E-37	-4.243506615	0.964298823	5.207805438
1350	EML4	Q9HC35	EMAP like 4		yes	no	no	no	no	yes	1.35127E-15	0.838570423	8.26471E-21	1.13269E-16	0.776257079	2.64755E-21	-2.404412711	-0.052943521	2.351469189
1351	SPON1	Q9HCB6	spondin 1		no	yes	no	no	yes	no	8.9839E-09	0.400037245	5.27797E-12	4.15612E-09	0.293457318	2.55648E-12	1.841966071	0.233946065	-1.608020007
1352	CHMP1A	Q9HD42	charged multivesicular body protein 1A		yes	no	no	no	no	yes	7.10185E-11	0.062013833	3.2669E-10	2.55177E-11	0.028042504	1.71857E-10	-1.44080095	0.593389976	2.034190926
1353	APMAP	Q9HDC9	adipocyte plasma membrane associated protein		yes	no	yes	no	no	no	0.00351193	0.006150477	0.827415222	0.002674922	0.001535529	0.816168074	-1.133797369	-1.194562491	-0.060765122
1354	VTA1	Q9NP79	vesicle trafficking 1		yes	no	no	no	no	yes	2.57056E-15	0.014244885	1.23285E-22	2.5857E-16	0.004498725	3.4578E-23	-2.484354934	-0.52418083	1.960174104
1355	RIC8A	Q9NPQ8	RIC8 guanine nucleotide exchange factor A		yes	no	no	no	no	yes	7.02807E-09	0.003626153	3.39789E-25	3.21602E-09	0.000802037	7.94431E-26	-1.652016625	0.773934852	2.425951477
1356	SPINK5	Q9NQ38	serine peptidase inhibitor Kazal type 5		no	yes	no	no	yes	no	5.40117E-11	0.000970095	5.89815E-20	1.88441E-11	0.00016703	1.96694E-20	1.492017364	-0.603891724	-2.095909088
1357	MEPE	Q9NQ76	matrix extracellular phosphoglycoprotein		no	yes	no	no	yes	no	3.92544E-12	0.194178708	2.83696E-14	1.09497E-12	0.116313662	1.23017E-14	1.709542363	-0.191233871	-1.900776234
1358	EIF2B3	Q9NR50	eukaryotic translation initiation factor 2B subunit		yes	no	no	no	no	yes	1.30446E-09	0.357534241	2.03049E-23	5.49682E-10	0.250776169	5.43733E-24	-2.594732469	0.336896607	2.931629076
1359	CTPS2	Q9NRF8	CTP synthase 2		yes	no	no	no	no	yes	3.32908E-16	0.000103957	3.52209E-37	1.9694E-17	8.94959E-06	6.86225E-39	-2.935880088	0.971921648	3.907801736
1360	OLFML3	Q9NRN5	olfactomedin like 3		no	yes	no	no	yes	no	1.42304E-07	0.694750219	2.7775E-13	7.36991E-08	0.599997244	1.26731E-13	1.895955735	0.134560209	-1.761395527
1361	HEBP1	Q9NRV9	heme binding protein 1		yes	no	no	no	no	yes	2.88569E-06	0.891563875	1.78011E-09	1.64878E-06	0.843895304	9.73537E-10	-1.071197738	-0.039197703	1.032000034
1362	VPS45	Q9NRW7	vacuolar protein sorting 45 homolog		yes	no	no	no	no	yes	1.64433E-15	0.030994473	7.6034E-17	1.4454E-16	0.011773019	2.95248E-17	-1.769683663	-0.355645124	1.414038539
1363	FAM114A2	Q9NRY5	family with sequence similarity 114 member A2		yes	no	no	no	no	yes	2.96512E-13	0.672155285	7.72621E-21	6.08609E-14	0.575915561	2.45754E-21	-2.615670494	0.117481357	2.733151852
1364	LTBP3	Q9NS15	latent transforming growth factor beta binding protein 3		no	yes	no	no	yes	no	1.09262E-05	0.666506791	3.07084E-11	6.5547E-06	0.569263843	1.54029E-11	1.206326194	-0.122369972	-1.328696166
1365	RGS18	Q9NS28	regulator of G protein signaling 18		yes	no	no	no	no	yes	1.65484E-15	0.00448135	9.27999E-33	1.46214E-16	0.001049777	7.14815E-34	-3.655955719	0.853839478	4.509795197
1366	LANCL2	Q9NS86	LanC like glutathione S-transferase 2		yes	no	no	no	no	yes	1.65852E-14	0.898756756	2.19736E-17	2.28278E-15	0.854368679	8.2936E-18	-1.506755424	-0.025910592	1.480844831
1367	HOMER2	Q9NSB8	homer scaffold protein 2		no	yes	no	no	yes	no	2.58241E-08	0.152390396	7.85557E-11	1.24615E-08	0.086379876	4.01499E-11	1.876448574	0.458849245	-1.41759933
1368	BMP2K	Q9NSY1	BMP2 inducible kinase	Fostamatinib	yes	no	no	no	no	yes	8.36334E-05	0.066191303	5.30125E-13	5.44545E-05	0.030356193	2.45486E-13	-1.037500893	0.542636541	1.580137434
1369	ATG3	Q9NT62	autophagy related 3		yes	no	no	no	no	yes	5.01819E-14	0.236047127	6.16878E-22	8.32196E-15	0.1489867	1.81122E-22	-2.479727446	0.282798881	2.762526327
1370	SACM1L	Q9NTJ5	SAC1 like phosphatidylinositide phosphatase		yes	no	yes	no	no	no	0.000102034	0.001594558	0.387252726	6.69436E-05	0.000294058	0.365144052	-1.614246763	-1.257844338	0.356402425
1371	OLA1	Q9NTK5	Obg like ATPase 1		yes	no	no	no	no	yes	4.93263E-11	0.736275918	2.19281E-25	1.70977E-11	0.650204696	4.96785E-26	-2.027776818	0.086655996	2.114432815
1372	ACSS1	Q9NUB1	acyl-CoA synthetase short chain family member 1	ATP	no	yes	no	yes	no	no	0.004590195	0.001737705	0.787640039	0.003533639	0.00032518	0.772650967	1.146457646	1.239988179	0.093530533
1373	CYRIB	Q9NUQ9	CYFIP related Rac1 interactor B		yes	no	no	no	no	yes	2.01416E-11	0.042064014	4.12209E-22	6.507E-12	0.01756561	1.18414E-22	-2.012949868	0.530795002	2.54374487
1374	EXOC1	Q9NV70	exocyst complex component 1		yes	no	no	no	no	yes	5.11166E-12	0.908553362	9.46505E-19	1.45452E-12	0.872738164	3.36659E-19	-3.1170934	0.051201697	3.168295097
1375	SEPTIN11	Q9NVA2	septin 11		yes	no	no	no	no	yes	8.93086E-12	0.739280394	2.12244E-33	2.69504E-12	0.653862859	1.38483E-34	-4.496667161	0.168273895	4.664941055
1376	DNAJC17	Q9NVM6	DnaJ heat shock protein family (Hsp40) member C17		yes	no	yes	no	no	no	7.42018E-12	3.96911E-05	0.15072674	2.18551E-12	2.53577E-06	0.136999474	-1.335818554	-1.025531553	0.310287001
1377	UGGT1	Q9NYU2	UDP-glucose glycoprotein glucosyltransferase 1		yes	no	no	no	no	yes	0.000552401	0.092109388	2.52232E-07	0.000388708	0.04565821	1.56117E-07	-1.065402769	0.53682959	1.602232359
1378	FAM120A	Q9NZB2	family with sequence similarity 120 member A		no	no	yes	no	no	yes	0.040523524	0.003752779	2.44785E-25	0.033931795	0.000836596	5.55674E-26	-1.246621762	2.096469734	3.343091495
1379	EHD3	Q9NZN3	EH domain containing 3		yes	no	no	no	no	yes	1.09104E-13	0.004772964	1.17199E-37	1.9972E-14	0.001141878	1.92449E-39	-3.862558406	0.991390974	4.85394938
1380	ARHGEF12	Q9NZN5	Rho guanine nucleotide exchange factor 12		yes	no	no	no	no	yes	2.72157E-17	0.275773305	1.01821E-08	9.24867E-19	0.181683002	5.81308E-09	-1.720894536	-0.271243088	1.449651449
1381	KLK14	Q9P0G3	kallikrein related peptidase 14		no	yes	no	no	yes	no	2.20958E-10	0.035851131	3.97262E-19	8.50996E-11	0.014100037	1.38781E-19	1.10238847	-0.345044572	-1.447433041
1382	STK26	Q9P289	serine/threonine kinase 26	2-[4-[4-[(5-cyclopropyl-1H-pyrazol-3-yl)amino]quinazolin-2-yl]iminocyclohexa-2,5-dien-1-yl]acetonitrile; Fostamatinib	yes	no	no	no	no	yes	1.15471E-15	0.577649007	3.41074E-28	9.41766E-17	0.474213557	5.54806E-29	-3.88714311	0.186370183	4.073513293
1383	RRBP1	Q9P2E9	ribosome binding protein 1	Radezolid	yes	no	no	no	no	yes	1.43003E-17	7.54231E-06	1.74893E-26	3.56373E-19	2.59726E-07	3.52236E-27	-2.291813967	0.808118164	3.099932131
1384	LARS1	Q9P2J5	leucyl-tRNA synthetase 1	Leucine	yes	no	no	no	no	yes	7.98386E-16	0.034365956	4.11753E-27	5.8242E-17	0.013375784	7.57477E-28	-2.740075341	0.518403593	3.258478933
1385	GMPR2	Q9P2T1	guanosine monophosphate reductase 2		yes	no	no	no	no	yes	0.000114697	0.857634645	9.57895E-06	7.54601E-05	0.799235554	6.50604E-06	-1.248070663	-0.082455291	1.165615372
1386	DPM3	Q9P2X0	dolichyl-phosphate mannosyltransferase subunit 3,		yes	no	no	no	no	yes	0.00026433	0.272713176	4.54911E-05	0.000179414	0.17917268	3.23199E-05	-1.525658165	0.193587873	1.719246038
1387	EIF3K	Q9UBQ5	eukaryotic translation initiation factor 3 subunit K		yes	no	no	no	no	yes	3.14248E-15	0.051546217	3.16693E-28	3.23219E-16	0.02257568	5.13712E-29	-2.840261254	0.468087463	3.308348717
1388	GRHPR	Q9UBQ7	glyoxylate and hydroxypyruvate reductase		yes	no	no	no	no	yes	2.23405E-20	0.000364042	3.91515E-28	6.07355E-23	4.47011E-05	6.43952E-29	-2.787689847	0.627262995	3.414952842
1389	DNAJB9	Q9UBS3	DnaJ heat shock protein family (Hsp40) member B9		no	yes	no	no	yes	no	1.9298E-06	0.710462097	2.09937E-13	1.09125E-06	0.6167854	9.51233E-14	1.286093757	-0.108750426	-1.394844183
1390	BIN2	Q9UBW5	bridging integrator 2		yes	no	no	no	no	yes	3.23104E-11	0.08561519	2.98216E-33	1.08043E-11	0.041624422	2.0944E-34	-3.950700659	0.762341541	4.7130422
1391	CLIP2	Q9UDT6	CAP-Gly domain containing linker protein 2		yes	no	no	no	no	yes	9.3355E-20	0.03800956	1.3051E-26	4.22995E-22	0.015396713	2.5901E-27	-3.411664072	0.425734435	3.837398507
1392	TJP2	Q9UDY2	tight junction protein 2		yes	no	no	no	no	yes	2.12022E-12	0.036399451	6.56452E-34	5.5143E-13	0.014464123	3.68827E-35	-4.331211389	0.917888537	5.249099926
1393	VTI1B	Q9UEU0	vesicle transport through interaction with t-SNAREs 1B		yes	no	no	no	no	yes	7.38943E-14	1.19545E-05	3.49826E-07	1.28235E-14	5.14578E-07	2.18582E-07	-2.043477297	-0.983726166	1.059751131
1394	ADD3	Q9UEY8	adducin 3		yes	no	no	no	no	yes	1.8631E-15	0.49393452	1.14054E-29	1.70524E-16	0.383565216	1.50953E-30	-3.86698609	0.222848059	4.089834149
1395	DNAH17	Q9UFH2	dynein axonemal heavy chain 17		no	yes	no	no	yes	no	2.16708E-10	0.013182859	3.33973E-07	8.32892E-11	0.004031912	2.08222E-07	1.511346039	0.504941234	-1.006404805
1396	LAMTOR3	Q9UHA4	late endosomal/lysosomal adaptor, MAPK and MTOR activator 3		yes	no	no	no	no	yes	1.47619E-08	0.532172289	4.11103E-09	6.93615E-09	0.426931817	2.2837E-09	-1.507536582	0.100970696	1.608507279
1397	SRP68	Q9UHB9	signal recognition particle 68		yes	no	no	no	no	yes	3.12089E-09	0.096918446	2.87159E-14	1.37025E-09	0.048832493	1.24648E-14	-1.382460577	0.407162706	1.789623283
1398	SEPTIN9	Q9UHD8	septin 9		yes	no	no	no	no	yes	7.47067E-13	0.352590974	2.9743E-22	1.76358E-13	0.246350377	8.49029E-23	-2.427502781	-0.238419932	2.189082848
1399	TAC3	Q9UHF0	tachykinin precursor 3	Copper	no	yes	no	no	yes	no	2.5827E-10	0.207003977	4.9031E-17	1.00406E-10	0.126247102	1.87948E-17	1.446875118	-0.259272585	-1.706147703
1400	EIF2B4	Q9UI10	eukaryotic translation initiation factor 2B subunit delta		yes	no	no	no	no	yes	5.9679E-12	0.361679137	3.73571E-24	1.7225E-12	0.2553222	9.51278E-25	-2.672398946	0.291828062	2.964227008
1401	ATP6V1H	Q9UI12	ATPase H+ transporting V1 subunit H		yes	no	no	no	no	yes	4.74108E-13	0.006217428	6.29725E-25	1.03328E-13	0.001557878	1.51225E-25	-2.087575075	0.647701609	2.735276684
1402	TAGLN3	Q9UI15	transgelin 3		yes	no	no	no	no	yes	8.25159E-11	0.565704205	4.16586E-19	2.98732E-11	0.46189351	1.46098E-19	-3.676501133	0.300588824	3.977089957
1403	LNPEP	Q9UIQ6	leucyl and cystinyl aminopeptidase		yes	no	yes	no	no	no	9.77347E-10	1.06581E-08	0.273891157	4.05641E-10	7.86272E-11	0.253662676	-1.585026984	-1.364618129	0.220408855
1404	DBNL	Q9UJU6	drebrin like		yes	no	no	no	no	yes	1.17509E-12	0.008476093	1.84919E-37	2.90598E-13	0.002315851	3.51908E-39	-3.614237197	0.939949207	4.554186404
1405	SERPINA10	Q9UK55	serpin family A member 10		no	yes	no	no	yes	no	1.10093E-06	0.341581922	4.77323E-08	6.14064E-07	0.23664647	2.84404E-08	1.634274627	0.286420758	-1.347853869
1406	TNIK	Q9UKE5	TRAF2 and NCK interacting kinase	Fostamatinib	yes	no	no	no	no	yes	5.64062E-16	0.227047217	5.77232E-32	3.73145E-17	0.140944289	5.02168E-33	-4.16805629	0.393901699	4.561957989
1407	VAV3	Q9UKW4	vav guanine nucleotide exchange factor 3		yes	no	no	no	no	yes	5.94447E-17	0.087609572	4.86333E-15	2.41553E-18	0.042792532	2.05595E-15	-1.432954289	0.327845673	1.760799962
1408	CDV3	Q9UKY7	CDV3 homolog		yes	no	no	no	no	yes	1.51187E-12	0.86256804	6.50651E-23	3.78823E-13	0.806288114	1.79246E-23	-2.725964247	-0.060577126	2.665387121
1409	RAB21	Q9UL25	RAB21, member RAS oncogene family		yes	no	no	no	no	yes	5.94447E-17	0.684394094	1.72779E-23	2.42411E-18	0.588572719	4.59545E-24	-2.394990612	-0.090621092	2.304369519
1410	PSME2	Q9UL46	proteasome activator subunit 2		yes	no	no	no	no	yes	7.49502E-17	0.010788379	1.63525E-28	3.22622E-18	0.00313826	2.54142E-29	-2.140170712	0.475749344	2.615920056
1411	MCTS1	Q9ULC4	MCTS1 re-initiation and release factor		yes	no	no	no	no	yes	2.44584E-08	0.131493135	2.04313E-15	1.17582E-08	0.072211001	8.5169E-16	-1.916826895	-0.455849609	1.460977286
1412	ASAP1	Q9ULH1	ArfGAP with SH3 domain, ankyrin repeat and PH		yes	no	no	no	no	yes	2.00857E-13	0.010583164	1.4705E-33	3.9498E-14	0.003064178	9.32802E-35	-2.889039015	0.723636892	3.612675908
1413	CORO1C	Q9ULV4	coronin 1C		yes	no	no	no	no	yes	5.43472E-08	0.091916972	1.35359E-30	2.69397E-08	0.045521183	1.57623E-31	-3.113571892	0.831373638	3.94494553
1414	SYNRG	Q9UMZ2	synergin gamma		yes	no	no	no	no	yes	1.26194E-06	0.194122332	2.32049E-05	7.07302E-07	0.116191935	1.61814E-05	-1.803182437	-0.557388365	1.245794072
1415	VPS4A	Q9UN37	vacuolar protein sorting 4 homolog A		yes	no	no	no	no	yes	3.79568E-12	0.39492847	2.76706E-24	1.0405E-12	0.287741269	7.00855E-25	-2.147991135	0.204692988	2.352684123
1416	G3BP2	Q9UN86	G3BP stress granule assembly factor 2		yes	no	no	no	no	yes	4.14329E-19	0.000110353	5.58695E-29	3.37921E-21	9.80028E-06	8.12602E-30	-2.913814824	0.781653908	3.695468732
1417	PACSIN2	Q9UNF0	protein kinase C and casein kinase substrate in neurons		yes	no	no	no	no	yes	2.75568E-10	0.268108912	5.89893E-35	1.07505E-10	0.175661752	2.51246E-36	-4.167862997	0.55626799	4.724130988
1418	SNX6	Q9UNH7	sorting nexin 6		yes	no	no	no	no	yes	1.48144E-14	0.002808392	2.51513E-29	1.98689E-15	0.000588851	3.5146E-30	-2.607218246	0.731029311	3.338247557
1419	PSMD13	Q9UNM6	proteasome 26S subunit, non-ATPase 13		yes	no	no	no	no	yes	1.8631E-15	0.039221411	7.88015E-27	1.70075E-16	0.016047546	1.52461E-27	-2.332143609	0.453281483	2.785425092
1420	NSFL1C	Q9UNZ2	NSFL1 cofactor		yes	no	no	no	no	yes	1.21501E-14	0.000823472	1.04797E-33	1.58001E-15	0.000132573	6.4103E-35	-2.192884023	0.655697666	2.848561688
1421	EXOC7	Q9UPT5	exocyst complex component 7		yes	no	no	no	no	yes	1.84613E-17	0.003782757	1.95981E-32	5.32987E-19	0.000846707	1.64279E-33	-3.343053447	0.718795401	4.061848848
1422	SHOC2	Q9UQ13	SHOC2 leucine rich repeat scaffold protein		yes	no	no	no	no	yes	7.08419E-15	0.01575006	4.21463E-25	8.40987E-16	0.005073989	9.93026E-26	-2.596645622	0.634873988	3.23151961

1423	DNM3	Q9UQ16	dynamin 3		yes	no	no	no	no	yes	6.45406E-09	0.058981645	1.59872E-20	2.94191E-09	0.026484282	5.21557E-21	-2.703680162	0.799819695	3.503499857
1424	SMC3	Q9UQE7	structural maintenance of chromosomes 3		yes	no	no	no	no	yes	5.54969E-10	1.93769E-06	2.03646E-17	2.24301E-10	4.56547E-08	7.65863E-18	-1.034713853	0.705233711	1.739947564
1425	CHMP2B	Q9UQN3	charged multivesicular body protein 2B		yes	no	no	no	no	yes	4.0655E-10	0.751401312	8.93813E-18	1.62104E-10	0.668668861	3.30067E-18	-2.04686245	0.100663569	2.147526019
1426	RTRAF	Q9Y224	RNA transcription, translation and transport factor		yes	no	no	no	no	yes	3.97854E-16	0.197749328	5.80281E-23	2.4156E-17	0.119162866	1.58808E-23	-3.46538662	-0.346182695	3.119203925
1427	PIN4	Q9Y237	peptidylprolyl cis/trans isomerase, NIMA-interacting 4		yes	no	no	no	no	yes	8.78387E-12	0.766851687	8.70945E-15	2.63874E-12	0.686240182	3.69767E-15	-1.992744976	-0.088588089	1.904156887
1428	HIGD1A	Q9Y241	HIG1 hypoxia inducible domain family member 1A		no	yes	no	no	yes	no	3.28824E-07	0.102916586	2.15225E-09	1.75661E-07	0.052787302	1.18778E-09	1.060110391	-0.199923163	-1.260033554
1429	HPSE	Q9Y251	heparanase		yes	no	no	no	no	yes	1.09457E-07	0.322093678	2.80698E-24	5.6043E-08	0.219934378	7.12237E-25	-3.110798962	0.532043707	3.642842669
1430	EIF3L	Q9Y262	eukaryotic translation initiation factor 3 subunit L		yes	no	no	no	no	yes	4.05144E-15	0.508948149	2.1072E-26	4.35067E-16	0.39987136	4.26787E-27	-3.126019566	0.180548642	3.306568208
1431	ST3GAL6	Q9Y274	ST3 beta-galactoside alpha-2,3-sialyltransferase 6		yes	no	no	no	no	yes	8.43268E-10	0.123794585	3.38518E-12	3.46936E-10	0.066861416	1.61973E-12	-1.4794189	0.269903717	1.749322617
1432	FARSA	Q9Y285	phenylalanyl-tRNA synthetase subunit alpha	Phenylalanine	yes	no	no	no	no	yes	4.29103E-09	0.069304689	1.77769E-24	1.91512E-09	0.032061662	4.44625E-25	-2.902185337	0.781156216	3.683341553
1433	DRG1	Q9Y295	developmentally regulated GTP binding protein 1		yes	no	no	no	no	yes	3.31997E-13	0.09911592	5.47181E-16	6.9047E-14	0.050312828	2.20658E-16	-1.961282678	0.455603164	2.416885842
1434	NCKAP1	Q9Y2A7	NCK associated protein 1		yes	no	no	no	no	yes	1.41105E-14	0.028609309	6.96019E-34	1.87969E-15	0.010668537	3.94211E-35	-3.969144239	0.765302406	4.734446644
1435	WDR37	Q9Y2I8	WD repeat domain 37		yes	no	no	no	no	yes	2.50807E-15	0.846004632	8.30694E-19	2.44329E-16	0.784288843	2.94714E-19	-3.082937609	-0.073211798	3.009725811
1436	EPB41L3	Q9Y2J2	erythrocyte membrane protein band 4.1 like 3		yes	no	no	no	no	yes	2.49035E-12	0.033438401	1.54231E-17	6.60732E-13	0.012954161	5.75136E-18	-1.643667846	0.492795143	2.136462989
1437	SSUH2	Q9Y2M2	ssu-2 homolog		no	yes	no	no	yes	no	7.96149E-06	0.154033751	7.9833E-10	4.71846E-06	0.087590556	4.27922E-10	1.83913105	0.573157882	-1.265973168
1438	LAMTOR2	Q9Y2Q5	late endosomal/lysosomal adaptor, MAPK and MTOR activator 2		yes	no	no	no	no	yes	8.33642E-13	0.495778567	1.20622E-11	1.99062E-13	0.385256114	5.94094E-12	-1.894206022	-0.125259783	1.768946238
1439	TMA7	Q9Y2S6	translation machinery associated 7 homolog		yes	no	no	no	no	yes	6.8233E-13	0.001033994	4.89712E-12	1.58293E-13	0.000178501	2.36313E-12	-2.483023401	-0.927025826	1.555997576
1440	AP3M1	Q9Y2T2	adaptor related protein complex 3 subunit mu 1		yes	no	no	no	no	yes	1.02794E-15	0.298899304	5.07471E-21	8.10428E-17	0.200710815	1.58886E-21	-3.87222202	-0.38566462	3.4865574
1441	CARHSP1	Q9Y2V2	calcium regulated heat stable protein 1		yes	no	no	no	no	yes	7.77394E-07	0.728589982	4.00113E-10	4.28324E-07	0.641766617	2.11206E-10	-1.385375299	-0.104593879	1.28078142
1442	GIT1	Q9Y2X7	GIT ArfGAP 1		yes	no	no	no	no	yes	7.78472E-18	0.000638836	1.88684E-31	1.41091E-19	9.54381E-05	1.84666E-32	-2.915403077	0.787952696	3.703355773
1443	DERA	Q9Y315	deoxyribose-phosphate aldolase		yes	no	no	no	no	yes	5.34936E-15	0.013866971	1.78933E-15	6.01106E-16	0.004316543	7.41029E-16	-2.276823588	-0.571801569	1.705022019
1444	LSM2	Q9Y333	LSM2 homolog, U6 small nuclear RNA and mRNA degradation associated		yes	no	no	no	no	yes	1.16779E-08	6.61732E-05	3.95938E-25	5.44473E-09	5.12715E-06	9.31091E-26	-1.263427221	0.875766297	2.139193519
1445	CAB39	Q9Y376	calcium binding protein 39		yes	no	no	no	no	yes	2.63141E-12	0.261733022	6.58623E-28	7.02267E-13	0.170179831	1.10715E-28	-2.56768442	0.315643936	2.883328355
1446	SBDS	Q9Y3A5	SBDS ribosome maturation factor		yes	no	no	no	no	yes	7.20398E-12	0.024097625	2.5452E-30	2.1119E-12	0.008603955	3.05608E-31	-3.194061382	0.780648092	3.974709474
1447	RNF11	Q9Y3C5	ring finger protein 11		yes	no	no	no	no	yes	3.51955E-09	0.113482792	6.68063E-07	1.55893E-09	0.059780195	4.22572E-07	-1.927057631	-0.478038079	1.449019551
1448	UFC1	Q9Y3C8	ubiquitin-fold modifier conjugating enzyme 1		yes	no	no	no	no	yes	2.79679E-16	0.078196218	1.01743E-30	1.5587E-17	0.037086253	1.15251E-31	-2.989646284	0.430995194	3.420641479
1449	CHMP3	Q9Y3E7	charged multivesicular body protein 3		yes	no	no	no	no	yes	1.28809E-16	0.250883216	9.08733E-22	5.80782E-18	0.16119275	2.70108E-22	-2.174345991	-0.196597428	1.977748563
1450	RTCB	Q9Y3I0	RNA 2',3'-cyclic phosphate and 5'-OH ligase	Sucrose	yes	no	no	no	no	yes	4.50578E-16	0.376245555	6.34057E-28	2.87419E-17	0.268503285	1.06299E-28	-3.655834133	-0.25849565	3.397338483
1451	FBXO7	Q9Y3I1	F-box protein 7		yes	no	no	no	no	yes	2.99335E-08	0.228175701	1.13947E-14	1.45395E-08	0.142571043	4.8532E-15	-1.269994862	0.279595282	1.549590143
1452	CCDC9	Q9Y3X0	coiled-coil domain containing 9		yes	no	no	no	no	yes	1.06225E-14	0.340920117	1.17471E-19	1.33804E-15	0.236033502	3.99198E-20	-2.673817032	-0.24845909	2.425357942
1453	HBS1L	Q9Y450	HBS1 like translational GTPase		yes	no	no	no	no	yes	1.60357E-11	0.139003275	9.11353E-30	5.12967E-12	0.077343009	1.17687E-30	-3.109457524	0.517526109	3.626983633
1454	ATP6V0A2	Q9Y487	ATPase H+ transporting V0 subunit a2		no	yes	no	yes	no	no	4.16481E-07	1.7666E-06	0.398957737	2.24186E-07	3.92223E-08	0.376903889	1.682923963	1.830688078	0.147764116
1455	TLN1	Q9Y490	talin 1		yes	no	no	no	no	yes	2.95112E-09	0.014933528	6.9837E-34	1.29304E-09	0.004770339	3.98707E-35	-3.843827682	1.287470008	5.13129769
1456	DAAM1	Q9Y4D1	dishevelled associated activator of morphogenesis 1		yes	no	no	no	no	yes	3.26628E-11	0.037927158	4.57606E-26	1.09813E-11	0.015346149	9.62071E-27	-2.969452012	0.760794425	3.730246437
1457	USP15	Q9Y4E8	ubiquitin specific peptidase 15		yes	no	no	no	no	yes	2.56015E-11	0.536955533	1.60633E-20	8.41009E-12	0.431426875	5.24769E-21	-1.991399908	0.164820101	2.15622001
1458	RIPOR2	Q9Y4F9	RHO family interacting cell polarization regulator 2		no	no	no	yes	no	yes	0.844226289	4.27668E-05	2.12473E-09	0.833961694	2.84854E-06	1.16971E-09	0.036534844	1.053334481	1.016799638
1459	RAPGEF2	Q9Y4G8	Rap guanine nucleotide exchange factor 2		yes	no	no	no	no	yes	1.48045E-14	0.010157132	1.53192E-28	1.97886E-15	0.002913214	2.33918E-29	-2.125807688	0.535786314	2.661594003
1460	MYO5A	Q9Y4I1	myosin VA		yes	no	no	no	no	yes	3.62192E-11	0.72764738	9.76259E-10	1.22755E-11	0.640560167	5.25066E-10	-1.850673351	0.133179871	1.983853221
1461	HYOU1	Q9Y4L1	hypoxia up-regulated 1		yes	no	no	no	no	yes	4.74915E-10	0.109912691	1.29495E-13	1.9087E-10	0.057272132	5.8264E-14	-1.780842264	0.343468156	2.12431042
1462	ARIH1	Q9Y4X5	ariadne RBR E3 ubiquitin protein ligase 1		yes	no	no	no	no	yes	8.64831E-09	0.001346313	7.35144E-19	3.98912E-09	0.000240348	2.60481E-19	-1.37494922	0.805138581	2.1800878
1463	LSM4	Q9Y4Z0	LSM4 homolog, U6 small nuclear RNA and mRNA degradation associated		yes	no	no	no	no	yes	4.67269E-07	0.097710638	2.40754E-19	2.52372E-07	0.049364459	8.34514E-20	-1.412705635	0.450918205	1.863623839
1464	PRRC2C	Q9Y520	proline rich coiled-coil 2C		yes	no	no	no	no	yes	3.636E-14	0.524135579	1.72987E-19	5.7003E-15	0.417741044	5.91777E-20	-1.887497875	0.137849856	2.025347731
1465	ANGPTL3	Q9Y5C1	angiopoietin like 3		no	yes	no	no	yes	no	1.36837E-13	0.015864685	2.19388E-11	2.56686E-14	0.005139669	1.09247E-11	1.992915131	0.54147246	-1.45144267
1466	PCYT1B	Q9Y5K3	phosphate cytidylyltransferase 1B, choline	Choline; Choline salicylate	yes	no	no	no	no	yes	1.55537E-10	0.109827136	3.05655E-14	5.81415E-11	0.057177789	1.32954E-14	-1.270691489	0.284575135	1.555266624
1467	CD2AP	Q9Y5K6	CD2 associated protein		yes	no	no	no	no	yes	6.78521E-12	0.11419554	4.66813E-16	1.98299E-12	0.060434119	1.87402E-16	-1.572764283	0.326016127	1.89878041
1468	ATP6V1D	Q9Y5K8	ATPase H+ transporting V1 subunit D		yes	no	no	no	no	yes	9.88636E-13	0.748724506	8.47279E-13	2.39208E-13	0.665753038	3.96574E-13	-1.544437428	0.082182616	1.626620044
1469	CDC42BPB	Q9Y5S2	CDC42 binding protein kinase beta		yes	no	no	no	no	yes	9.45257E-14	0.009195913	2.88659E-30	1.67893E-14	0.002570856	3.50524E-31	-3.156468383	0.824944478	3.981412861
1470	SNX5	Q9Y5X3	sorting nexin 5		yes	no	no	no	no	yes	7.61602E-13	0.644175288	1.1547E-21	1.80134E-13	0.545520441	3.46359E-22	-2.354967243	-0.118928301	2.236038942
1471	LRRFIP2	Q9Y608	LRR binding FLII interacting protein 2		yes	no	no	no	no	yes	1.23596E-13	0.853990663	1.21299E-17	2.29047E-14	0.792853134	4.50679E-18	-2.639225138	0.066076309	2.705301446
1472	F11R	Q9Y624	F11 receptor		yes	no	no	no	no	yes	1.19734E-13	0.129649018	6.20727E-28	2.21348E-14	0.070696475	1.03783E-28	-3.388632668	0.490352831	3.878985499
1473	CLIC4	Q9Y696	chloride intracellular channel 4		yes	no	no	no	no	yes	1.11008E-15	0.138067608	2.08651E-19	8.98344E-17	0.07644704	7.20398E-20	-2.56014248	0.427531938	2.987674418
1474	SAR1B	Q9Y6B6	secretion associated Ras related GTPase 1B	Guanosine-5'-Diphosphate	yes	no	no	no	no	yes	9.42157E-16	0.024450511	1.70865E-25	7.16555E-17	0.008785344	3.84001E-26	-4.382099969	0.812563638	5.194663607
1475	EMILIN1	Q9Y6C2	elastin microfibril interfacer 1		yes	no	no	no	no	yes	7.62504E-08	0.04650424	1.79976E-14	3.85571E-08	0.019918932	7.72257E-15	-2.297612353	0.74775503	3.045367383
1476	STK24	Q9Y6E0	serine/threonine kinase 24	Fostamatinib	yes	no	no	no	no	yes	4.12095E-13	0.239897861	7.88253E-35	8.85061E-14	0.151634579	3.5716E-36	-4.149746852	0.460633273	4.610380125
1477	BZW2	Q9Y6E2	basic leucine zipper and W2 domains 2		yes	no	no	no	no	yes	5.15674E-13	0.436369065	1.67316E-27	1.14779E-13	0.328611412	2.96424E-28	-3.94638156	0.302317935	4.248699495
1478	IGF2BP2	Q9Y6M1	insulin like growth factor 2 mRNA binding protein 2		no	no	yes	no	yes	no	0.027708429	0.007587078	4.22132E-12	0.022862279	0.001973259	2.02555E-12	1.027939989	-1.365046223	-2.392986212
1479	CSNK1G3	Q9Y6M4	casein kinase 1 gamma 3	Purvalanol A; {4-Amino-2-[(4-methoxyphenyl)amino]-1,3-thiazol-5-yl}(4-methoxyphenyl)methanone; 4-Amino-2-[(3-chlorophenyl)amino]-5-(4-fluorobenzoyl)-1,3-thiazol-3-ium; K-00546	yes	no	no	no	no	yes	2.61771E-13	0.776095046	1.26975E-23	5.3137E-14	0.699226867	3.34841E-24	-1.91448858	0.064585292	1.979073872
1480	ROBO1	Q9Y6N7	roundabout guidance receptor 1		no	no	no	yes	no	yes	0.707257147	0.001050699	8.88717E-06	0.688990877	0.000181861	5.99189E-06	-0.113526672	1.04374601	1.157272682
1481	WASF2	Q9Y6W5	WASP family member 2		yes	no	no	no	no	yes	2.03845E-15	0.052508609	2.12529E-33	1.89344E-16	0.023162107	1.40179E-34	-3.614798268	0.570928559	4.185726827
1482	PDLIM1	O00151	PDZ and LIM domain 1		yes	no	no	yes	no	yes	4.43073E-13	5.38717E-05	1.11134E-35	9.59623E-14	3.95433E-06	3.92771E-37	-3.798234829	1.706689196	5.504924024
1483	RAB27B	O00194	RAB27B, member RAS oncogene family		yes	no	no	yes	no	yes	1.34676E-11	0.000136693	6.29953E-29	4.23495E-12	1.30247E-05	9.21952E-30	-3.903103106	1.864186393	5.767289499
1484	PGRMC1	O00264	progesterone receptor membrane component 1	Dextromethorphan; Nortriptyline; Sertraline	yes	no	no	yes	no	yes	0.000920617	1.51279E-07	2.79066E-07	0.000663245	1.71362E-09	1.73104E-07	-1.509365445	1.088757482	2.598122927
1485	ARPC1B	O15143	actin related protein 2/3 complex subunit 1B	N-[2-(2-methyl-1H-indol-3-yl)ethyl]thiophene-2-carboxamide; (2S)-2-(3-bromophenyl)-3-(5-chloro-2-	yes	no	no	yes	no	yes	1.29161E-11	0.008610625	2.35503E-33	4.03811E-12	0.002360411	1.62195E-34	-3.860911066	1.145353107	5.006264173
1486	ARPC2	O15144	actin related protein 2/3 complex subunit 2	N-[2-(2-methyl-1H-indol-3-yl)ethyl]thiophene-2-carboxamide; (2S)-2-(3-bromophenyl)-3-(5-chloro-2-	yes	no	no	yes	no	yes	2.84201E-13	0.000472088	2.12529E-33	5.78189E-14	6.4123E-05	1.40595E-34	-3.714063233	1.364475897	5.078539131
1487	ARPC3	O15145	actin related protein 2/3 complex subunit 3	N-[2-(2-methyl-1H-indol-3-yl)ethyl]thiophene-2-carboxamide; (2S)-2-(3-bromophenyl)-3-(5-chloro-2-	yes	no	no	yes	no	yes	1.89935E-13	2.35302E-05	1.01505E-36	3.70919E-14	1.21542E-06	2.71355E-38	-3.909605674	1.823055335	5.73266101
1488	SLC16A3	O15427	solute carrier family 16 member 3	Pyruvic acid	yes	no	no	yes	no	yes	1.10373E-08	9.81042E-05	3.36372E-17	5.12606E-09	8.22351E-06	1.28026E-17	-1.952679166	1.172729769	3.125408935
1489	ARPC5	O15511	actin related protein 2/3 complex subunit 5	N-[2-(2-methyl-1H-indol-3-yl)ethyl]thiophene-2-carboxamide; (2S)-2-(3-bromophenyl)-3-(5-chloro-2-	yes	no	no	yes	no	yes	8.14901E-12	5.46828E-07	6.02679E-38	2.42587E-12	8.17641E-09	8.73844E-40	-3.508064207	2.472591502	5.980655709
1490	ARHGAP6	O43182	Rho GTPase activating protein 6		yes	no	no	yes	no	yes	5.84385E-12	0.000160048	1.17199E-37	1.67875E-12	1.57454E-05	2.06623E-39	-4.084004195	1.803746798	5.887750993
1491	HNRNPR	O43390	heterogeneous nuclear ribonucleoprotein R		yes	no	yes	no	no	yes	2.57825E-14	2.15647E-05	3.5954E-10	3.80839E-15	1.05528E-06	1.89463E-10	-2.972271655	-1.357867435	1.61440422
1492	AKR7A2	O43488	aldo-keto reductase family 7 member A2		yes	no	no	yes	no	yes	1.83882E-15	0.000540113	4.36965E-37	1.66635E-16	7.8068E-05	9.50353E-39	-3.441808668	1.02336386	4.465172528
1493	AHCYL1	O43865	adenosylhomocysteinase like 1		yes	no	no	yes	no	yes	1.56006E-13	0.003723534	1.21305E-32	2.98298E-14	0.000828389	9.6736E-34	-3.773393804	1.074666451	4.848060255
1494	PIP5K1C	O60331	phosphatidylinositol-4-phosphate 5-kinase type 1		yes	no	no	yes	no	yes	4.17554E-18	2.12265E-10	1.14557E-33	6.05425E-20	6.73247E-13	7.11113E-35	-2.739068907	1.652108543	4.39117745
1495	DIAPH1	O60610	diaphanous related formin 1		yes	no	no	yes	no	yes	3.04762E-13	1.29485E-06	2.03475E-32	6.31065E-14	2.52282E-08	1.71483E-33	-2.391893907	1.441501877	3.833395784
1496	USO1	O60763	USO1 vesicle transport factor		yes	no	yes	no	no	yes	1.11008E-15	0.002046181	6.14744E-08	9.00333E-17	0.000400438	3.67955E-08	-2.979409194	-1.125994244	1.85341495
1497	SRGAP2	O75044	SLIT-ROBO Rho GTPase activating protein 2		yes	no	no	yes	no	yes	1.57551E-07	6.43307E-05	2.51415E-28	8.18808E-08	4.89694E-06	4.02128E-29	-1.332081143	1.060183467	2.392264611
1498	WDR1	O75083	WD repeat domain 1		yes	no	no	yes	no	yes	9.84078E-10	0.001093881	3.69097E-41	4.08881E-10	0.000190822	5.01717E-44	-4.391385798	1.935769388	6.327155185
1499	ROCK2	O75116	Rho associated coiled-coil containing protein kinase 2	Fasudil; Y-27632; Fostamatinib; Ripasudil; Netarsudil	yes	no	no	yes	no	yes	1.94682E-12	0.002596536	1.97531E-39	5.0104E-13	0.000530602	1.27206E-41	-3.573058785	1.102015877	4.675074662
1500	STX11	O75558	syntaxin 11		yes	no	no	yes	no	yes	5.11669E-13	0.002003681	7.02053E-37	1.13382E-13	0.000389479	1.68594E-38	-3.764096264	1.171832338	4.935928601
1501	RP2	O75695	RP2 activator of ARL3 GTPase		yes	no	no	yes	no	yes	8.86248E-14	5.96511E-06	1.45723E-23	1.55405E-14	1.74457E-07	3.85601E-24	-1.827430679	1.124802961	2.952233639
1502	ENDOD1	O94919	endonuclease domain containing 1		yes	no	no	yes	no	yes	2.88503E-06	1.54931E-05	1.6903E-11	1.64709E-06	6.9498E-07	8.38641E-12	-2.478396696	1.549706434	4.02810313
1503	RTN3	O95197	reticulon 3		yes	no	no	yes	no	yes	8.17494E-06	0.000529431	1.92541E-09	4.84866E-06	7.59247E-05	1.05649E-09	-2.450981494	1.160242954	3.611224448
1504	CYB5R3	P00387	cytochrome b5 reductase 3	NADH; Flavin adenine dinucleotide; Copper	yes	no	no	yes	no	yes	2.18975E-07	2.72302E-05	7.50559E-14	1.14895E-07	1.50525E-06	3.33619E-14	-2.881218526	1.828756294	4.709974819

1505	GSR	P00390	glutathione-disulfide reductase	Glutathione; NADH; Carmustine; 3,6-dihydroxy-xanthene-9-propionic acid; 3-sulfino-L-alanine; Glutathionylspermidine disulfide; 3-(Prop-2-Ene-1-Sulfinyl)-Propene-1-Thiol; Flavin adenine dinucleotide; Glutathione disulfide; 3-nitro-L-tyrosine; 2-(2-PHENYL-3-PYRIDIN-2-YL-4,5,6,7-TETRAHYDRO-2H-ISOPHOSPHINDOL-1-YL)PYRIDINE; 6-(3-METHYL-1,4-DIOXO-1,4-DIHYDRONAPHTHALEN-2-	yes	no	no	yes	no	yes	1.97845E-17	0.000367647	4.67316E-22	5.91653E-19	4.5477E-05	1.3488E-22	-2.672182737	1.072570146	3.744752883
1506	F13A1	P00488	coagulation factor XIII A chain	N-Acetyl-Serine; Thrombin; Prothrombin; Human thrombin; Anti-inhibitor coagulant complex	yes	no	no	yes	no	yes	4.14518E-14	1.10365E-06	1.00826E-32	6.63504E-15	2.05028E-08	7.94914E-34	-3.235397071	1.851160653	5.086557724
1507	PNP	P00491	purine nucleoside phosphorylase	Cladribine; Didanosine; 8-azaguanine; 2,6-Diamino-(S)-9-[2-(Phosphonomethoxy)Propyl]Purine; Immucillin-G; Guanine; 2-Amino-7-[2-(2-Hydroxy-1-Hydroxymethyl-Ethylamino)-Ethyl]-1,7-Dihydro-Purin-6-One; Peldesine; 9-deazainosine; Guanosine; Ribose-1-Phosphate; (3R,4R)-3-Hydroxy-4-(hydroxymethyl)-1-[(4-oxo-4,4a,5,7a-tetrahydro-3H-pyrrolo[3,2-d]pyrimidin-7-yl)methyl]pyrrolidinium; 3'-deoxyguanosine; (2S,3R,4S,5S)-3,4-Dihydroxy-2-[(methylsulfanyl)methyl]-5-(4-oxo-4,5-dihydro-1H-pyrrolo[3,2-d]pyrimidin-7-yl)pyrrolidinium; Hypoxanthine; 9-(5,5-Difluoro-5-Phosphonopentyl)Guanine; Inosine; 9-DEAZAINOSINE-2',3'-O-ETHYLIDENEPHOSPHONATE; GUANOSINE-2',3'-O-ETHYLIDENEPHOSPHONATE;	yes	no	no	yes	no	yes	5.49958E-13	0.000186151	3.78018E-30	1.24345E-13	1.93995E-05	4.69311E-31	-3.134426736	1.358870188	4.493296924
1508	CAT	P04040	catalase	Fomepizole; Cannabidiol; Medical Cannabis; Nabiximols	yes	no	no	yes	no	yes	2.06385E-07	0.000470198	3.50825E-26	1.07915E-07	6.34885E-05	7.31217E-27	-1.928402762	1.281634569	3.21003733
1509	ALDOA	P04075	aldolase, fructose-bisphosphate A	Zinc; 1,6-Fructose Diphosphate (Linear Form); Dihydroxyacetone phosphate; 1,6-DI-O-PHOSPHONO-D-MANNITOL; N-(4-CHLOROPHENYL)-3-(PHOSPHONOOXY)NAPHTHALENE-2-CARBOXAMIDE; Artenimol; Zinc acetate; Zinc chloride; Zinc sulfate, unspecified form	yes	no	no	yes	no	yes	2.05238E-13	4.07042E-05	1.83652E-34	4.04524E-14	2.65582E-06	8.73739E-36	-3.704432021	1.684170086	5.388602107
1510	HSPB1	P04792	heat shock protein family B (small) member 1	Apatorsen; Artenimol; Phenethyl	yes	no	no	yes	no	yes	6.16707E-13	0.00179378	4.3406E-35	1.41393E-13	0.000336486	1.7504E-36	-3.618876805	1.153604115	4.77248092
1511	APP	P05067	amyloid beta precursor protein	Deferoxamine; Aluminium; Zinc; L-methionine (R)-S-oxide; Resveratrol; Tromethamine; Phenserine; Tetrathiomolybdate; CAD106; Mito-4509; Edonerpic; Dimercaprol; Copper; Florbetaben (18F); Florbetapir (18F); Flutemetamol (18F); Zinc acetate; Aluminium phosphate; Aluminum acetate;	yes	no	no	yes	no	yes	6.50418E-05	0.008842638	2.7642E-20	4.16715E-05	0.002452059	9.09294E-21	-1.593287165	1.086353745	2.67964091
1512	TPM3	P06753	tropomyosin 3	Phenethyl Isothiocyanate	yes	no	no	yes	no	yes	3.54327E-11	4.20063E-05	3.1706E-34	1.19928E-11	2.77885E-06	1.56591E-35	-3.298188765	1.813258136	5.111446901
1513	LDHB	P07195	lactate dehydrogenase B	NADH; 4-Hydroxy-1,2,5-oxadiazole-3-carboxylic acid; Oxamic Acid; Stiripentol; Artenimol	yes	no	no	yes	no	yes	5.39895E-13	0.000466975	4.5823E-34	1.21336E-13	6.25797E-05	2.40846E-35	-2.909936215	1.097895843	4.007832058
1514	GPX1	P07203	glutathione peroxidase 1	Glutathione; Cannabidiol; Medical Cannabis; Nabiximols	yes	no	no	yes	no	yes	2.02834E-17	0.000191999	1.03664E-33	6.15762E-19	2.01829E-05	6.29408E-35	-3.719144398	1.070646395	4.789790793
1515	PGK2	P07205	phosphoglycerate kinase 2		yes	no	no	yes	no	yes	1.19552E-08	0.000343274	9.15797E-22	5.58488E-09	4.16844E-05	2.72623E-22	-1.612992105	1.010707132	2.623699237
1516	CTSD	P07339	cathepsin D	Insulin pork; S-Methylcysteine; 1h-Benoximidazole-2-Carboxylic Acid; 2-Morpholinoethylamine; 5-AMINO-6-CYCLOHEXYL-4-HYDROXY-2-ISOBUTYL-HEXANOIC ACID; CYCLOHEXYLMETHYL-2,3-DIHYDROXY-5-METHYL-HEXYLAMIDE	yes	no	no	yes	no	yes	9.65148E-08	1.06402E-06	2.9052E-16	4.90664E-08	1.92845E-08	1.15445E-16	-1.430839223	1.134563408	2.565402631
1517	GP1BA	P07359	glycoprotein Ib platelet subunit alpha	Ibuprofen; Egaptivon pegol; liposomal prostaglandin E1; Dexibuprofen	yes	no	no	yes	no	yes	1.81039E-09	4.72633E-05	1.97531E-39	7.73537E-10	3.29794E-06	1.34253E-41	-4.662597221	2.934412721	7.597009942
1518	CAPN1	P07384	calpain 1	4-[[(2S)-2-[[(2S)-3-Carboxy-2-hydroxypropanoyl]amino]-4-methylpentanoyl]amino]butyl-(diaminomethylidene)azanium; N-[(benzyloxy)carbonyl]-L-leucyl-N-[(1S)-3-fluoro-1-(4-hydroxybenzyl)-2-oxopropyl]-L-leucinamide; (2S)-4-METHYL-2-(3-PHENYLTHIOUREIDO)-N-((3S)-TETRAHYDRO-2-HYDROXY-3-FURANYL)PENTANAMIDE	yes	no	no	yes	no	yes	9.29321E-18	8.62245E-07	3.13202E-34	1.79231E-19	1.40647E-08	1.53266E-35	-2.845432229	1.213305299	4.058737528
1519	TUBB	P07437	tubulin beta class I	Vinorelbine; Vincristine; Vinblastine; Podofilox; Colchicine; Epothilone D; Patupilone; CYT997; CA4P; ZEN-012; Copper; Artenimol; Vinflunine; Depatuximab mafodotin; Milataxel;	yes	no	no	yes	no	yes	1.48619E-11	6.53248E-06	7.5591E-36	4.71378E-12	2.10152E-07	2.46604E-37	-4.19832702	2.568326193	6.766653214
1520	PFN1	P07737	profilin 1	7-HYDROXY-4-METHYL-3-(2-HYDROXY-ETHYL)COUMARIN;	yes	no	no	yes	no	yes	1.52665E-11	0.009844825	3.02908E-38	4.85767E-12	0.002792415	3.43122E-40	-4.074105494	1.146249744	5.220355238
1521	HMBS	P08397	hydroxymethylbilane synthase		yes	no	no	yes	no	yes	0.000408171	0.000870129	5.70996E-19	0.000283334	0.000144076	2.01543E-19	-1.111227661	1.233866956	2.345094617
1522	IGFBP1	P08833	insulin like growth factor binding protein 1		no	yes	no	yes	yes	no	7.96108E-09	0.000823747	3.60265E-06	3.66491E-09	0.000132874	2.38163E-06	3.606358365	1.915967424	-1.690390941
1523	CD63	P08962	CD63 molecule		yes	no	no	yes	no	yes	4.95056E-13	7.29882E-07	1.00112E-23	1.08791E-13	1.15749E-08	2.61733E-24	-3.145103107	1.932415626	5.077518733
1524	HMGB1	P09429	high mobility group box 1	Chloroquine; Ethyl pyruvate	yes	no	yes	no	no	yes	1.31707E-08	0.00052254	9.315E-05	6.17059E-09	7.43442E-05	6.75728E-05	-2.18067004	-1.176834922	1.003835118
1525	TPM1	P09493	tropomyosin 1	Artenimol; Phenethyl Isothiocyanate	yes	no	no	yes	no	yes	3.80546E-12	0.003203191	1.36371E-22	1.04491E-12	0.000692307	3.84334E-23	-2.811675651	1.093751773	3.905427424
1526	CLTA	P09496	clathrin light chain A		yes	no	no	yes	no	yes	3.88743E-12	6.23547E-06	2.09862E-23	1.07094E-12	1.94947E-07	5.62929E-24	-3.042313164	1.718979375	4.761292539
1527	CLTB	P09497	clathrin light chain B		yes	no	no	yes	no	yes	8.7923E-12	2.3634E-09	1.45105E-20	2.64526E-12	1.28504E-11	4.71411E-21	-1.771220372	1.196830435	2.968050808
1528	TUBA3C	P0DPH7	tubulin alpha 3c		yes	no	no	yes	no	yes	1.24453E-08	6.56872E-05	3.57125E-43	5.81947E-09	5.05973E-06	1.61815E-46	-4.260799359	2.837649365	7.098448724
1529	LIPC	P11150	lipase C, hepatic type		no	yes	no	yes	yes	no	1.37511E-07	0.007717584	1.65901E-09	7.11541E-08	0.002028182	9.04301E-10	2.510003116	1.162282428	-1.347720688
1530	ACTN1	P12814	actinin alpha 1	Human calcitonin; Copper	yes	no	no	yes	no	yes	1.80504E-07	1.68362E-06	5.36091E-24	9.40553E-08	3.62943E-08	1.37241E-24	-3.16794489	3.265407193	6.433352083
1531	GP1BB	P13224	glycoprotein Ib platelet subunit beta		yes	no	no	yes	no	yes	4.48797E-08	0.00023939	3.60789E-39	2.21653E-08	2.67917E-05	2.61559E-41	-4.362623539	2.78662201	7.149245549
1532	ICAM2	P13598	intercellular adhesion molecule 2		yes	no	no	yes	no	yes	9.9365E-13	0.000680398	4.45437E-27	2.40871E-13	0.000106669	8.29519E-28	-2.690403855	1.025544502	3.715948357

1533	FDPS	P14324	farnesyl diphosphate synthase	Pamidronic acid; Zoledronic acid; Alendronic acid; Ibandronate; Risedronic acid; Dimethylallyl Diphosphate; Isopentyl Pyrophosphate; Geranyl Diphosphate; ISOPENTENYL PYROPHOSPHATE; Incadronic acid; Minodronic acid; Farnesyl diphosphate; Geranylgeranyl diphosphate	yes	no	no	yes	no	yes	3.25088E-16	5.31933E-05	1.60401E-29	1.88542E-17	3.83223E-06	2.16581E-30	-2.852499537	1.032530093	3.88502963
1534	GP9	P14770	glycoprotein IX platelet	Quinine	yes	no	no	yes	no	yes	1.24838E-09	2.8397E-05	1.88729E-40	5.24354E-10	1.62122E-06	5.98598E-43	-4.514063473	2.916796298	7.430859771
1535	PRKCA	P17252	protein kinase C alpha	Phosphatidyl serine; Vitamin E; Tamoxifen; Ingenol mebutate; Aprinocarsen; Midostaurin; Perifosine; Ellagic acid; Bryostatin 1; alpha-Tocopherol succinate; D-alpha-Tocopherol acetate	yes	no	no	yes	no	yes	1.49218E-12	0.002334382	4.95824E-34	3.73213E-13	0.000470684	2.65099E-35	-3.542816122	1.15137792	4.694194041
1536	ITGA2	P17301	integrin subunit alpha 2		yes	no	no	yes	no	yes	5.5781E-09	0.001493505	2.46631E-23	2.51482E-09	0.000272715	6.67145E-24	-2.62041444	1.284326804	3.904741244
1537	CTPS1	P17812	CTP synthase 1	L-Glutamine	yes	no	no	yes	no	yes	1.00495E-09	0.000474464	9.41639E-33	4.18007E-10	6.51394E-05	7.38122E-34	-2.767671759	1.396824599	4.164496358
1538	PSMC3	P17980	proteasome 26S subunit, ATPase 3	Phenethyl Isothiocyanate	yes	no	no	yes	no	yes	4.93307E-14	2.35302E-05	1.17501E-30	8.15845E-15	1.20352E-06	1.34697E-31	-2.267616052	1.072450687	3.340066738
1539	VCL	P18206	vinculin		yes	no	no	yes	no	yes	1.57141E-10	0.000124176	7.63632E-41	5.88755E-11	1.15029E-05	1.73002E-43	-4.39917692	2.242286953	6.641463873
1540	GNAZ	P19086	G protein subunit alpha z		yes	no	no	yes	no	yes	1.483E-13	0.002429898	1.53917E-35	2.80876E-14	0.000494347	5.64897E-37	-3.615631065	1.055181167	4.670812232
1541	FST	P19883	follistatin	D-Myo-Inositol-Hexasulphate	no	yes	no	yes	yes	no	1.04184E-16	1.0703E-07	1.14018E-07	4.53179E-18	1.03938E-09	6.94338E-08	2.600926686	1.419882864	-1.181043821
1542	FLNA	P21333	filamin A	Artenimol	yes	no	no	yes	no	yes	7.15142E-10	0.001649866	2.40815E-33	2.91306E-10	0.0003065	1.66944E-34	-3.654259584	1.592580895	5.246840479
1543	EEF1B2	P24534	eukaryotic translation elongation factor 1 beta 2		yes	no	no	yes	no	yes	7.46622E-14	5.61814E-05	1.61545E-38	1.30086E-14	4.20024E-06	1.53713E-40	-3.550343141	1.451311759	5.0016549
1544	PSMA1	P25786	proteasome 20S subunit alpha 1	(3AR,6R,6AS)-6-((S)-((S)-CYCLOHEX-2-ENYL)(HYDROXY)METHYL)-6A-METHYL-4-OXO-HEXAHYDRO-2H-FURO[3,2-C]PYRROLE-6-	yes	no	no	yes	no	yes	4.72602E-12	0.000164867	5.88524E-29	1.33622E-12	1.64344E-05	8.58653E-30	-2.117260899	1.008305063	3.125565961
1545	PSMA2	P25787	proteasome 20S subunit alpha 2	(3AR,6R,6AS)-6-((S)-((S)-CYCLOHEX-2-ENYL)(HYDROXY)METHYL)-6A-METHYL-4-OXO-HEXAHYDRO-2H-FURO[3,2-C]PYRROLE-6-	yes	no	no	yes	no	yes	1.02175E-09	0.000276622	4.94791E-17	4.2546E-10	3.20866E-05	1.8989E-17	-1.601437999	1.129303889	2.730741888
1546	CALR	P27797	calreticulin	Antihemophilic factor, human recombinant; Tenecteplase; Melatonin; Lanoteplase; Copper; Calcium citrate; Calcium Phosphate; Lonoctocog alfa; Moroctocog alfa; Calcium phosphate dihydrate	yes	no	no	yes	no	yes	3.50206E-10	1.06581E-08	2.0809E-21	1.38051E-10	7.65267E-11	6.33604E-22	-1.390225572	1.23522389	2.625449462
1547	CANX	P27824	calnexin	Antihemophilic factor, human recombinant; Tenecteplase; Lanoteplase; Calcium citrate; Calcium Phosphate; Lonoctocog alfa; Moroctocog alfa; Calcium phosphate	yes	no	no	yes	no	yes	1.43275E-05	2.07469E-05	4.21489E-10	8.66015E-06	9.92755E-07	2.22871E-10	-2.2200292	1.352121153	3.572150353
1548	PSMA5	P28066	proteasome 20S subunit alpha 5	(3AR,6R,6AS)-6-((S)-((S)-CYCLOHEX-2-ENYL)(HYDROXY)METHYL)-6A-METHYL-4-OXO-HEXAHYDRO-2H-FURO[3,2-C]PYRROLE-6-	yes	no	no	yes	no	yes	7.04788E-08	2.42106E-05	5.58066E-22	3.55428E-08	1.28348E-06	1.62084E-22	-1.592144804	1.407970687	3.000115491
1549	PSMB4	P28070	proteasome 20S subunit beta 4	(3AR,6R,6AS)-6-((S)-((S)-CYCLOHEX-2-ENYL)(HYDROXY)METHYL)-6A-METHYL-4-OXO-HEXAHYDRO-2H-FURO[3,2-C]PYRROLE-6-	yes	no	no	yes	no	yes	7.02748E-13	2.72581E-05	1.30687E-20	1.63985E-13	1.51914E-06	4.23977E-21	-1.83418647	1.166059458	3.000245928
1550	EPHB2	P29323	EPH receptor B2	Phosphoaminophosphonic Acid-Adenylate Ester; Fostamatinib	yes	no	no	yes	no	yes	8.31772E-12	0.008058744	4.03646E-35	2.48363E-12	0.002173488	1.60946E-36	-3.893213648	1.122561368	5.015775015
1551	ERP29	P30040	endoplasmic reticulum protein 29		yes	no	no	yes	no	yes	5.9314E-16	2.72302E-05	1.89073E-35	4.00457E-17	1.49858E-06	7.02491E-37	-3.100248909	1.197571228	4.297820137
1552	CORO1A	P31146	coronin 1A		yes	no	no	yes	no	yes	2.91309E-15	6.59736E-06	1.4705E-33	2.95665E-16	2.15229E-07	9.26215E-35	-3.014395802	1.387951983	4.402347785
1553	HNRNPH3	P31942	heterogeneous nuclear ribonucleoprotein H3	Copper	yes	no	yes	no	no	yes	1.60743E-13	6.81484E-05	6.70367E-12	3.08085E-14	5.4037E-06	3.26223E-12	-3.060518815	-1.37313908	1.687379735
1554	TAGLN2	P37802	transgelin 2	Artenimol	yes	no	no	yes	no	yes	4.27581E-12	0.004688365	4.15229E-41	1.19149E-12	0.001115266	7.52567E-44	-4.315352023	1.240058599	5.555410622
1555	RPS19	P39019	ribosomal protein S19	Artenimol	yes	no	yes	no	no	yes	1.77761E-09	0.000699146	1.03146E-05	7.58726E-10	0.000110241	7.01971E-06	-2.397643182	-1.217682477	1.179960705
1556	GP5	P40197	glycoprotein V platelet		yes	no	no	yes	no	yes	6.59856E-11	0.002148866	1.45327E-20	2.34104E-11	0.00042841	4.72792E-21	-3.22571117	1.495904675	4.721615845
1557	STAT3	P40763	signal transducer and activator of transcription 3	ENMD-1198	yes	no	no	yes	no	yes	6.76455E-16	0.000409533	8.56449E-20	4.81212E-17	5.18912E-05	2.88329E-20	-2.493747006	1.0680724	3.561819405
1558	MDH2	P40926	malate dehydrogenase 2	NADH; Citric acid; Xanthinol	yes	no	no	yes	no	yes	2.10657E-05	0.000359483	1.45446E-09	1.29525E-05	4.38156E-05	7.90168E-10	-2.436647903	1.303200933	3.739848837
1559	GARS1	P41250	glycyl-tRNA synthetase 1	Glycine	yes	no	yes	no	no	yes	6.02304E-11	0.009359956	3.65546E-09	2.12048E-11	0.002621067	2.024E-09	-2.932171068	-1.085366352	1.846804716
1560	TEC	P42680	tec protein tyrosine kinase	Fostamatinib; Zanubrutinib	yes	no	no	yes	no	yes	4.57064E-16	8.16546E-06	7.37875E-34	2.9615E-17	2.89263E-07	4.27947E-35	-3.046831238	1.305355928	4.352187166
1561	RANGAP1	P46060	Ran GTPase activating protein 1		yes	no	no	yes	no	yes	0.000178943	0.000602973	9.9204E-18	0.000119836	8.90663E-05	3.66789E-18	-1.007066899	1.004724265	2.011791164
1562	CAPZA2	P47755	capping actin protein of muscle Z-line subunit alpha 2		yes	no	no	yes	no	yes	1.07156E-13	0.000186745	3.34075E-34	1.94697E-14	1.95461E-05	1.71049E-35	-3.685564339	1.398239901	5.083804239
1563	CAPZB	P47756	capping actin protein of muscle Z-line subunit beta		yes	no	no	yes	no	yes	1.65852E-14	0.001093881	7.02053E-37	2.2912E-15	0.000190491	1.67436E-38	-3.887611813	1.147550203	5.035162016
1564	MAPKAPK2	P49137	MAPK activated protein kinase 2	Staurosporine; 3-{[(1R)-1-phenylethyl]amino}-4-(pyridin-4-ylamino)cyclobut-3-ene-1,2-dione; (4R)-N-[4-({[2-(DIMETHYLAMINO)ETHYL]AMINO}CARBONYL)-1,3-THIAZOL-2-YL]-4-METHYL-1-OXO-2,3,4,9-TETRAHYDRO-1H-BETA-CARBOLINE-6-CARBOXAMIDE; (10R)-10-methyl-3-(6-methylpyridin-3-yl)-9,10,11,12-tetrahydro-8H-[1,4]diazepino[5',6':4,5]thieno[3,2-f]quinolin-8-one; (3R)-3-(aminomethyl)-9-methoxy-1,2,3,4-tetrahydro-5H-[1]benzothieno[3,2-e][1,4]diazepin-5-one; 2-[2-(2-FLUOROPHENYL)PYRIDIN-4-YL]-1,5,6,7-TETRAHYDRO-4H-PYRROLO[3,2-C]PYRIDIN-4-ONE; 2-(2-	yes	no	no	yes	no	yes	1.19293E-18	6.02698E-06	1.47156E-30	1.30482E-20	1.82967E-07	1.72027E-31	-2.799536749	1.151832537	3.951369286
1565	PSMB2	P49721	proteasome 20S subunit beta 2	(3AR,6R,6AS)-6-((S)-((S)-CYCLOHEX-2-ENYL)(HYDROXY)METHYL)-6A-METHYL-4-OXO-HEXAHYDRO-2H-FURO[3,2-C]PYRROLE-6-CARBALDEHYDE; Carfilzomib	yes	no	no	yes	no	yes	3.19826E-11	0.002452628	3.08391E-27	1.06802E-11	0.000500083	5.64522E-28	-2.607738978	1.025666166	3.633405144
1566	GNAQ	P50148	G protein subunit alpha q		yes	no	no	yes	no	yes	2.37177E-09	0.006168251	3.79853E-32	1.0263E-09	0.001542761	3.28735E-33	-3.274637763	1.276685187	4.55132295
1567	GNG10	P50151	G protein subunit gamma 10		yes	no	no	yes	no	yes	8.59231E-08	2.07898E-05	3.19281E-25	4.3526E-08	1.00793E-06	7.42143E-26	-1.671909914	1.397641916	3.06955183
1568	VASP	P50552	vasodilator stimulated phosphoprotein		yes	no	no	yes	no	yes	1.51646E-12	2.35302E-05	4.14133E-40	3.80798E-13	1.20984E-06	2.01079E-42	-3.963601878	1.946069257	5.909671135
1569	DUSP3	P51452	dual specificity phosphatase 3		yes	no	no	yes	no	yes	6.945E-20	5.3249E-05	8.471E-27	2.29588E-22	3.86037E-06	1.65044E-27	-2.877616947	1.05704533	3.934662277
1570	HSD17B4	P51659	hydroxysteroid 17-beta dehydrogenase 4	NADH; (R)-3-hydroxydecanoyl-CoA	yes	no	no	yes	no	yes	8.29319E-15	0.000134936	1.62611E-33	1.0033E-15	1.2656E-05	1.03888E-34	-4.216737187	1.509618659	5.726355846
1571	PGD	P52209	phosphogluconate dehydrogenase	Gadopentetic acid; Dacarbazine; Ketotifen; 6-phospho-D-gluconic acid; Nicotinamide 8-bromo-adenine dinucleotide phosphate	yes	no	no	yes	no	yes	1.03803E-18	1.36719E-06	3.56171E-31	1.08177E-20	2.7257E-08	3.72793E-32	-2.868956389	1.146802075	4.015758464
1572	CAPZA1	P52907	capping actin protein of muscle Z-line subunit alpha 1		yes	no	no	yes	no	yes	9.1219E-14	8.55463E-05	1.54101E-38	1.6078E-14	6.93828E-06	1.39648E-40	-3.781877553	1.497598844	5.279476397
1573	PRKAG1	P54619	protein kinase AMP-activated non-catalytic subunit		yes	no	no	yes	no	yes	0.002412153	6.81484E-05	3.88675E-23	0.001812121	5.38969E-06	1.06018E-23	-1.414535175	2.358717609	3.773252784
1574	VCP	P55072	valosin containing protein	Phosphoaminophosphonic Acid-Adenylate Ester; Phenethyl Isothiocyanate	yes	no	no	yes	no	yes	2.5094E-10	0.000434712	8.90254E-35	9.74425E-11	5.63333E-05	4.11445E-36	-3.026063432	1.445116987	4.471180419
1575	NAP1L1	P55209	nucleosome assembly protein 1 like 1		yes	no	no	yes	no	yes	1.10532E-12	0.001940372	4.87962E-37	2.72448E-13	0.000372302	1.10549E-38	-4.042710362	1.29730769	5.340018052
1576	ARPC4	P59998	actin related protein 2/3 complex subunit 4	N-[2-(2-methyl-1H-indol-3-yl)ethyl]thiophene-2-carboxamide; (2S)-2-(3-bromophenyl)-3-(5-chloro-2-	yes	no	no	yes	no	yes	2.61826E-10	0.008688489	5.27741E-29	1.01907E-10	0.002392164	7.62798E-30	-3.661319735	1.274635772	4.935955507
1577	ACTB	P60709	actin beta	Quercetin; Phenethyl Isothiocyanate	yes	no	no	yes	no	yes	6.48765E-08	0.003915614	6.6672E-40	3.25705E-08	0.000885316	3.62512E-42	-3.821149968	1.813270445	5.634420413

1578	PSMA6	P60900	proteasome 20S subunit alpha 6	(3AR,6R,6AS)-6-((S)-((S)-CYCLOHEX-2-ENYL)(HYDROXY)METHYL)-6A-METHYL-4-OXO-HEXAHYDRO-2H-FURO[3,2-C]PYRROLE-6-	yes	no	no	yes	no	yes	1.29719E-12	1.72736E-05	5.74678E-33	3.22094E-13	7.905E-07	4.19226E-34	-2.514854233	1.332906878	3.847761111
1579	ACTR3	P61158	actin related protein 3	N-[2-(2-methyl-1H-indol-3-yl)ethyl]thiophene-2-carboxamide; (2S)-2-(3-bromophenyl)-3-(5-chloro-2-	yes	no	no	yes	no	yes	2.28341E-09	2.35302E-05	7.88253E-35	9.85993E-10	1.19055E-06	3.54627E-36	-3.033442896	2.095475593	5.128918489
1580	ACTR2	P61160	actin related protein 2	N-[2-(2-methyl-1H-indol-3-yl)ethyl]thiophene-2-carboxamide; (2S)-2-(3-bromophenyl)-3-(5-chloro-2-	yes	no	no	yes	no	yes	9.56469E-14	0.005669949	1.6001E-28	1.70318E-14	0.001395008	2.47229E-29	-3.830617329	1.103668205	4.934285534
1581	HSPE1	P61604	heat shock protein family E (Hsp10) member 1	Phenethyl Isothiocyanate	yes	no	no	yes	no	yes	0.001056212	0.00043877	3.40107E-07	0.000765718	5.80521E-05	2.12201E-07	-1.735668392	1.105263863	2.840932255
1582	YWHAG	P61981	tyrosine 3-monooxygenase/tryptophan 5-monooxygenase activation protein gamma		yes	no	no	yes	no	yes	2.96168E-13	0.000312988	9.16995E-36	6.06561E-14	3.72976E-05	3.08893E-37	-3.985503206	1.504905951	5.490409157
1583	ACTA2	P62736	actin alpha 2, smooth muscle	Phenethyl Isothiocyanate	yes	no	no	yes	no	yes	1.39114E-10	0.000217934	1.7383E-24	5.15611E-11	2.34774E-05	4.33196E-25	-2.257858038	1.257282731	3.515140769
1584	TPM4	P67936	tropomyosin 4	Phenethyl Isothiocyanate	yes	no	no	yes	no	yes	1.60752E-10	0.000225912	1.16849E-36	6.04548E-11	2.49762E-05	3.17667E-38	-3.669279683	1.798461113	5.467740796
1585	TUBA4A	P68366	tubulin alpha 4a	Vincristine; Podofilox; Epothilone D; Patupilone; CYT997; Cabazitaxel; Phenethyl Isothiocyanate	yes	no	no	yes	no	yes	3.56535E-10	1.1934E-06	1.31738E-41	1.40708E-10	2.27109E-08	1.19382E-44	-4.414462024	3.410353928	7.824815952
1586	TUBB4B	P68371	tubulin beta 4B class IVb	Albendazole; Mebendazole; Epothilone D; Patupilone; Oxibendazole; CYT997; Phenethyl Isothiocyanate	yes	no	no	yes	no	yes	1.44619E-10	4.62841E-05	2.30625E-40	5.39292E-11	3.18767E-06	9.40474E-43	-4.49361871	2.497799229	6.99141794
1587	PAFAH1B2	P68402	platelet activating factor acetylhydrolase 1b catalytic subunit 2		yes	no	no	yes	no	yes	2.37776E-11	9.65493E-05	1.03041E-24	7.76786E-12	8.00568E-06	2.51184E-25	-1.922472668	1.042409965	2.964882633
1588	GSTO1	P78417	glutathione S-transferase omega 1	Glutathione	yes	no	no	yes	no	yes	6.61258E-13	0.000110338	6.56218E-33	1.52506E-13	9.74896E-06	4.84656E-34	-2.989628885	1.302606152	4.292235037
1589	CLTC	Q00610	clathrin heavy chain		yes	no	no	yes	no	yes	8.65034E-09	0.000437646	6.0335E-16	3.99397E-09	5.71101E-05	2.43855E-16	-2.394175056	1.195163106	3.589338161
1590	PFKP	Q01813	phosphofructokinase, platelet		yes	no	no	yes	no	yes	2.05513E-13	2.36943E-05	4.86684E-31	4.06928E-14	1.24537E-06	5.20423E-32	-3.720970415	1.846963699	5.567934114
1591	RHAG	Q02094	Rh associated glycoprotein		no	yes	yes	no	yes	no	0.001232048	0.000612805	1.90033E-10	0.000901008	9.07962E-05	9.91066E-11	1.088431848	-1.102530509	-2.190962357
1592	UBXN1	Q04323	UBX domain protein 1		yes	no	no	yes	no	yes	2.37958E-09	4.56929E-05	6.81452E-29	1.03183E-09	3.12625E-06	1.0035E-29	-1.647771758	1.098309356	2.746081113
1593	YWHAH	Q04917	tyrosine 3-monooxygenase/tryptophan 5-monooxygenase activation protein eta	Phenethyl Isothiocyanate	yes	no	no	yes	no	yes	5.51678E-12	0.000364042	2.00752E-38	1.57729E-12	4.46667E-05	2.09211E-40	-4.12539908	1.667815404	5.793214484
1594	PLP2	Q04941	proteolipid protein 2		yes	no	no	yes	no	yes	4.21082E-13	1.94664E-05	4.08781E-20	9.08178E-14	9.0849E-07	1.35396E-20	-2.92997964	1.239606573	4.169586212
1595	DNM1	Q05193	dynamin 1		yes	no	no	yes	no	yes	1.97948E-12	0.001169797	1.17199E-37	5.12134E-13	0.000206715	2.05494E-39	-3.298710788	1.123520277	4.422231065
1596	AIMP2	Q13155	aminoacyl tRNA synthetase complex interacting multifunctional protein 2		yes	no	no	yes	no	yes	2.26133E-12	0.003179151	3.5348E-17	5.94278E-13	0.000685671	1.34858E-17	-2.234578722	1.018198079	3.252776801
1597	NNT	Q13423	nicotinamide nucleotide transhydrogenase	NADH; 7-thionicotinamide-adenine-dinucleotide phosphate; Nicotinamide adenine dinucleotide phosphate; Xanthinol	yes	no	yes	no	no	yes	4.00334E-08	0.000136056	0.002911383	1.97174E-08	1.28844E-05	0.002303251	-2.693215287	-1.422182634	1.271032653
1598	IQGAP2	Q13576	IQ motif containing GTPase activating protein 2		yes	no	no	yes	no	yes	3.69158E-10	0.002984188	1.17199E-37	1.46359E-10	0.000632805	2.12412E-39	-4.12792339	1.581786044	5.709709434
1599	FHL1	Q13642	four and a half LIM domains 1		yes	no	no	yes	no	yes	5.5181E-12	0.001943122	1.91866E-35	1.58017E-12	0.000375066	7.21564E-37	-4.161569437	1.43927067	5.600840107
1600	TUBB2A	Q13885	tubulin beta 2A class IIa	CYT997; Phenethyl Isothiocyanate	yes	no	no	yes	no	yes	1.8206E-10	0.002763018	2.06986E-19	6.90457E-11	0.00056963	7.13713E-20	-3.201185822	1.524815367	4.726001189
1601	MLEC	Q14165	malectin		yes	no	no	yes	no	yes	4.1105E-06	2.33059E-06	1.9358E-10	2.38211E-06	5.74094E-08	1.01044E-10	-2.2716866	1.308502514	3.580189114
1602	EIF2B1	Q14232	eukaryotic translation initiation factor 2B subunit alpha		yes	no	no	yes	no	yes	1.98762E-08	0.000967014	6.46981E-21	9.46527E-09	0.000166062	2.04032E-21	-1.850786694	1.108932929	2.959719623
1603	CTTN	Q14247	cortactin		yes	no	no	yes	no	yes	4.63696E-14	0.000110938	3.81642E-37	7.56368E-15	9.97304E-06	7.78156E-39	-3.783880155	1.437858937	5.221739092
1604	FLNC	Q14315	filamin C		yes	no	no	yes	no	yes	5.29139E-07	0.004292727	1.84545E-21	2.87706E-07	0.000999167	5.60241E-22	-2.075852215	1.146735454	3.222587668
1605	PDIA6	Q15084	protein disulfide isomerase family A member 6		yes	no	no	yes	no	yes	9.57263E-13	1.92072E-07	1.03624E-22	2.30316E-13	2.34977E-09	2.89697E-23	-2.669215028	1.648830024	4.318045052
1606	MAPRE2	Q15555	microtubule associated protein RP/EB family member 2		yes	no	no	yes	no	yes	8.43268E-10	0.000170785	1.42519E-39	3.46801E-10	1.74113E-05	8.39484E-42	-3.788494852	2.034566792	5.823061644
1607	TRIP10	Q15642	thyroid hormone receptor interactor 10		yes	no	no	yes	no	yes	4.2174E-13	0.000208441	3.31336E-30	9.1151E-14	2.21947E-05	4.06851E-31	-3.089886531	1.250861191	4.340747723
1608	MYLK	Q15746	myosin light chain kinase	Fostamatinib	yes	no	no	yes	no	yes	2.69151E-12	0.000600524	8.29164E-37	7.23183E-13	8.78882E-05	2.02877E-38	-3.497030868	1.313384101	4.810414969
1609	STXBP2	Q15833	syntaxin binding protein 2		yes	no	no	yes	no	yes	7.15961E-14	0.00402535	5.17484E-35	1.23274E-14	0.000921831	2.15716E-36	-3.727465849	1.017683248	4.745149096
1610	ZYX	Q15942	zyxin	Artenimol	yes	no	no	yes	no	yes	1.68236E-10	0.001942867	6.02318E-38	6.34219E-11	0.000374136	8.46029E-40	-4.400523009	1.712557458	6.113080467
1611	SYPL1	Q16563	synaptophysin like 1		yes	no	no	yes	no	yes	0.003807383	0.005201918	2.60154E-16	0.002912036	0.001261	1.03024E-16	-1.428188695	1.491078635	2.91926733
1612	LRRFIP1	Q32MZ4	LRR binding FLII interacting protein 1		yes	no	no	yes	no	yes	1.07096E-14	7.75838E-14	5.64317E-26	1.35872E-15	7.0307E-17	1.20688E-26	-1.751976237	1.251295963	3.0032722
1613	TBC1D10B	Q4KMP7	TBC1 domain family member 10B		yes	no	no	yes	no	yes	2.33849E-12	0.000416848	4.45164E-29	6.15614E-13	5.30739E-05	6.37389E-30	-2.682251313	1.130564589	3.812815902
1614	HSP90AB2P	Q58FF8	None	None	yes	no	no	yes	no	yes	4.84306E-12	3.92754E-10	5.35329E-28	1.3737E-12	1.42367E-12	8.90194E-29	-1.818292141	1.832237929	3.65053007
1615	WDR44	Q5JSH3	WD repeat domain 44		yes	no	no	yes	no	yes	1.17523E-14	4.51841E-05	4.67445E-36	1.50108E-15	3.07096E-06	1.39789E-37	-3.674027737	1.457872959	5.131900697
1616	FNBP1L	Q5T0N5	formin binding protein 1 like		yes	no	no	yes	no	yes	5.29057E-13	8.04475E-05	2.41899E-30	1.18421E-13	6.45184E-06	2.88262E-31	-2.910441308	1.315072248	4.225513555
1617	TWF2	Q6IBS0	twinfilin actin binding protein 2		yes	no	no	yes	no	yes	4.50578E-16	4.42531E-05	5.62196E-26	2.89139E-17	2.98763E-06	1.19979E-26	-3.750036352	1.553008456	5.303044808
1618	TMED8	Q6PL24	transmembrane p24 trafficking protein family member 8		yes	no	no	yes	no	yes	1.44123E-15	3.06323E-06	3.08084E-38	1.23422E-16	8.05017E-08	3.62944E-40	-3.586204534	1.630332936	5.21653747
1619	DOK3	Q7L591	docking protein 3		yes	no	no	yes	no	yes	5.53394E-15	8.55463E-05	5.48987E-37	6.26862E-16	6.92291E-06	1.26861E-38	-3.80055028	1.402676144	5.203226424
1620	RASGRP2	Q7LDG7	RAS guanyl releasing protein 2		yes	no	no	yes	no	yes	6.43754E-15	1.0005E-06	4.14133E-40	7.52553E-16	1.74005E-08	2.0641E-42	-3.761431378	1.892708941	5.654140319
1621	KTN1	Q86UP2	kinectin 1		yes	no	no	yes	no	yes	3.612E-14	3.45082E-08	8.22668E-28	5.6463E-15	2.81444E-10	1.41646E-28	-1.579512229	1.151430271	2.7309425
1622	PACS2	Q86VP3	phosphofurin acidic cluster sorting protein 2		yes	no	no	yes	no	yes	1.49902E-08	0.00877497	1.07735E-32	7.05023E-09	0.002425343	8.54262E-34	-2.992111068	1.206221242	4.19833231
1623	TREML1	Q86YW5	triggering receptor expressed on myeloid cells like 1		yes	no	no	yes	no	yes	1.20474E-10	8.85372E-06	6.6926E-21	4.42702E-11	3.20932E-07	2.11361E-21	-3.222591208	2.087438733	5.310029941
1624	ARHGAP18	Q8N392	Rho GTPase activating protein 18		yes	no	no	yes	no	yes	2.43458E-11	0.000110938	3.51961E-38	7.97553E-12	1.00289E-05	4.30583E-40	-4.265751522	2.036733314	6.302484835
1625	TC2N	Q8N9U0	tandem C2 domains, nuclear		yes	no	no	yes	no	yes	4.84152E-17	0.000694867	2.86454E-33	1.79884E-18	0.000109252	1.99882E-34	-4.351576667	1.162467296	5.514043964
1626	CCNY	Q8ND76	cyclin Y		yes	no	no	yes	no	yes	4.54396E-12	0.000175492	2.41899E-30	1.27651E-12	1.80501E-05	2.87835E-31	-2.349080996	1.088148675	3.437229671
1627	DTX3L	Q8TDB6	deltex E3 ubiquitin ligase 3L		yes	no	no	yes	no	yes	5.89515E-08	0.001654649	2.55725E-14	2.93613E-08	0.000308138	1.10656E-14	-1.403670892	1.010934487	2.414605379
1628	TMEM40	Q8WWA1	transmembrane protein 40		yes	no	no	yes	no	yes	4.16335E-17	0.00402535	3.30399E-33	1.52801E-18	0.000922894	2.3354E-34	-5.081078138	1.086119102	6.16719724
1629	ARHGEF2	Q92974	Rho/Rac guanine nucleotide exchange factor 2		yes	no	no	yes	no	yes	4.15237E-18	4.67972E-05	8.48139E-27	5.83251E-20	3.24421E-06	1.65631E-27	-2.59468168	1.028919563	3.623601242
1630	PPP1R14A	Q96A00	protein phosphatase 1 regulatory inhibitor subunit 14A		yes	no	no	yes	no	yes	1.73994E-14	1.68362E-06	9.16995E-36	2.42031E-15	3.57117E-08	3.11621E-37	-3.359607096	1.775038253	5.134645349
1631	ZNF462	Q96JM2	zinc finger protein 462		no	yes	no	yes	no	yes	0.001252955	1.33293E-18	2.59846E-15	0.000918569	6.03955E-22	1.09142E-15	1.134175843	4.523309713	3.38913387
1632	PIP5K1A	Q99755	phosphatidylinositol-4-phosphate 5-kinase type 1 alpha		yes	no	no	yes	no	yes	1.88658E-10	1.10901E-05	3.46939E-29	7.18046E-11	4.67322E-07	4.90462E-30	-1.956584678	1.324914869	3.281499546
1633	TUBA1C	Q9BQE3	tubulin alpha 1c	Epothilone D; Patupilone; CYT997; 2-MERCAPTO-N-[1,2,3,10-TETRAMETHOXY-9-OXO-5,6,7,9-TETRAHYDRO-BENZO[A]HEPTALEN-7-	yes	no	no	yes	no	yes	6.69141E-09	0.000424239	7.91904E-41	3.05313E-09	5.42072E-05	2.15289E-43	-4.450077671	2.414845113	6.864922784
1634	FERMT1	Q9BQL6	FERM domain containing kindlin 1		yes	no	no	yes	no	yes	2.49011E-05	0.002090943	7.54979E-28	1.54348E-05	0.00041023	1.28624E-28	-1.91999907	1.486072553	3.406071623
1635	CORO1B	Q9BR76	coronin 1B		yes	no	no	yes	no	yes	1.11891E-06	5.0182E-06	3.27842E-34	6.25108E-07	1.40973E-07	1.64886E-35	-1.961888396	2.132388186	4.094276582
1636	ESYT1	Q9BSJ8	extended synaptotagmin 1		yes	no	no	yes	no	yes	6.74407E-11	3.90942E-05	5.64594E-19	2.39877E-11	2.47992E-06	1.99028E-19	-2.817645891	1.453014642	4.270660533
1637	TMEM109	Q9BVC6	transmembrane protein 109		yes	no	no	yes	no	yes	0.000342489	0.00077139	1.51185E-08	0.000236188	0.000122332	8.76832E-09	-1.947605076	1.258816746	3.206421822
1638	JAM3	Q9BX67	junctional adhesion molecule 3		yes	no	no	yes	no	yes	3.25879E-16	7.13293E-10	2.45884E-26	1.90478E-17	3.55515E-12	5.03578E-27	-2.920473401	1.640125539	4.56059894
1639	NAA15	Q9BXJ9	N-alpha-acetyltransferase 15, NatA auxiliary subunit		yes	no	no	yes	no	yes	2.07205E-08	0.008058744	1.67692E-28	9.90491E-09	0.002176263	2.62138E-29	-2.376293219	1.00447782	3.380771039
1640	UBA5	Q9GZZ9	ubiquitin like modifier activating enzyme 5		yes	no	no	yes	no	yes	7.98178E-11	0.000106303	5.93236E-26	2.87879E-11	9.24791E-06	1.2741E-26	-2.280719599	1.24627464	3.526994239
1641	TUBB1	Q9H4B7	tubulin beta 1 class VI	Vindesine; Paclitaxel; Docetaxel; Epothilone D; Patupilone; CYT997; ZEN-012; Cabazitaxel; Eribulin; Milataxel; Phenethyl	yes	no	no	yes	no	yes	6.93173E-11	2.95687E-06	2.30625E-40	2.48123E-11	7.63668E-08	8.49632E-43	-4.990406575	3.321833073	8.312239648
1642	SMOC1	Q9H4F8	SPARC related modular calcium binding 1		no	yes	no	yes	yes	no	3.75245E-12	9.00195E-06	2.42903E-10	1.02525E-12	3.34463E-07	1.27229E-10	2.41355032	1.375809008	-1.037741313
1643	RAB18	Q9NP72	RAB18, member RAS oncogene family		yes	no	no	yes	no	yes	7.25746E-14	2.72302E-05	2.11782E-24	1.25287E-14	1.49581E-06	5.30655E-25	-2.707246548	1.197639917	3.904886466
1644	CASS4	Q9NQ75	Cas scaffold protein family member 4		yes	no	no	yes	no	yes	1.85313E-14	0.003626153	1.05469E-38	2.61135E-15	0.000804074	9.07979E-41	-5.093974644	1.25208384	6.346058484
1645	RTN4	Q9NQC3	reticulon 4		yes	no	no	yes	no	yes	9.35779E-08	3.59821E-05	6.63485E-16	4.74462E-08	2.16838E-06	2.69362E-16	-3.444323385	2.318575087	5.762898472
1646	PDLIM7	Q9NR12	PDZ and LIM domain 7		yes	no	no	yes	no	yes	3.59634E-12	0.00171485	4.35045E-37	9.79339E-13	0.000320126	9.26466E-39	-4.031257416	1.354574859	5.385832274
1647	SAR1A	Q9NR31	secretion associated Ras related GTPase 1A		yes	no	no	yes	no	yes	3.32908E-16	0.000680398	5.4414E-30	1.97603E-17	0.000106506	6.82949E-31	-4.890323803	1.432927748	6.323251552
1648	RAB6B	Q9NRW1	RAB6B, member RAS oncogene family		yes	no	no	yes	no	yes	2.16528E-12	0.000475936	1.28372E-30	5.66092E-13	6.57728E-05	1.48323E-31	-3.655755366	1.432124154	5.08787952
1649	TMEM30A	Q9NV96	transmembrane protein 30A		yes	no	no	yes	no	yes	0.003476056	6.23547E-06	2.05093E-09	0.002639723	1.94129E-07	1.12815E-09	-1.064952569	1.333423722	2.398376291
1650	RIC8B	Q9NVN3	RIC8 guanine nucleotide exchange factor B		yes	no	no	yes	no	yes	1.76801E-12	6.77845E-06	2.93672E-27	4.51016E-13	2.27279E-07	5.36247E-28	-1.885018826	1.072838437	2.957857263
1651	CMTM6	Q9NX76	CKLF like MARVEL transmembrane domain containing		yes	no	no	yes	no	yes	2.66861E-12	0.000927781	1.02272E-25	7.15821E-13	0.000156612	2.25675E-26	-3.042385449	1.146090921	4.18847637
1652	TMOD3	Q9NYL9	tropomodulin 3		yes	no	no	yes	no	yes	1.00568E-14	4.32051E-06	1.56747E-22	1.25768E-15	1.17458E-07	4.4318E-23	-3.302502477	1.497790706	4.800293183
1653	ITSN2	Q9NZM3	intersectin 2		yes	no	no	yes	no	yes	1.18699E-06	0.001250432	2.32399E-21	6.6422E-07	0.000222098	7.10782E-22	-1.577935219	1.112927714	2.690862933
1654	GMIP	Q9P107	GEM interacting protein		yes	no	no	yes	no	yes	8.16345E-11	0.000540113	4.52869E-28	2.94802E-11	7.80268E-05	7.46916E-29	-2.227692936	1.0473231	3.275016036
1655	SLAIN2	Q9P270	SLAIN motif family member 2		yes	no	no	yes	no	yes	7.63156E-09	0.003942878	1.01096E-29	3.50976E-09	0.000893321	1.32382E-30	-2.643703935	1.161628943	3.805332878
1656	ATP5IF1	Q9UII2	ATP synthase inhibitory factor subunit 1		yes	no	no	yes	no	yes	4.23636E-05	1.20501E-05	2.4625E-12	2.67196E-05	5.35073E-07	1.17379E-12	-1.411865536	1.326595646	2.738461182

1657	NUDC	Q9Y266	nuclear distribution C, dynein complex regulator	Phenethyl Isothiocyanate	yes	no	no	yes	no	yes	9.30986E-12	1.0005E-06	3.88667E-29	2.82206E-12	1.76799E-08	5.52974E-30	-1.534962995	1.064867167	2.599830162
1658	FHOD1	Q9Y613	formin homology 2 domain containing 1		yes	no	no	yes	no	yes	4.48317E-12	0.000638836	1.501E-32	1.25334E-12	9.525E-05	1.2378E-33	-3.492709207	1.393344656	4.886053863

supplementary table 2 The list of differentially expressed proteins between BrM and NAT in tissue samples by Student's t-test

NO.	Gene Names	Uniprot	Protein name	Drug (DrugBank)	Log2(FC(BrM/NAT))	*p* -value	FDR	Group
1	GTPBP10	A4D1E9	GTP binding protein 10		1.602667886	0.00171236	0.05086288	Up in BrM
2	NUDT19	A8MXV4	nudix hydrolase 19		1.863633871	0.00100451	0.04322166	Up in BrM
3	SMIM1	B2RUZ4	small integral membrane protein 1 (Vel blood group)		1.356038162	0.00272496	0.05914721	Up in BrM
4	QSOX1	O00391	quiescin sulfhydryl oxidase 1		1.570913737	0.00663961	0.07906805	Up in BrM
5	GOLIM4	O00461	golgi integral membrane protein 4		2.039958829	0.0000116	0.01508854	Up in BrM
6	PLOD2	O00469	procollagen-lysine,2-oxoglutarate 5-dioxygenase 2	Ascorbic acid	1.922742388	0.00109465	0.04500749	Up in BrM
7	PES1	O00541	pescadillo ribosomal biogenesis factor 1		1.328761146	0.00188593	0.05289456	Up in BrM
8	MPHOSPH10	O00566	M-phase phosphoprotein 10		1.591569844	0.00693414	0.07944195	Up in BrM
9	MAN2B1	O00754	mannosidase alpha class 2B member 1		1.606288244	0.00369312	0.06474551	Up in BrM
10	CYB561D	O14569	cytochrome b561 family member D2		1.161842266	0.00835231	0.0838895	Up in BrM
11	CHD1	O14646	chromodomain helicase DNA binding protein 1		1.121988955	0.001118	0.04558136	Up in BrM
12	TOR1B	O14657	torsin family 1 member B		1.662081615	0.00053959	0.03619764	Up in BrM
13	APAF1	O14727	apoptotic peptidase activating factor 1	ATP	1.041204218	0.00734153	0.07967639	Up in BrM
14	GEMIN2	O14893	gem nuclear organelle associated protein 2		1.633870358	0.0039394	0.06646771	Up in BrM
15	HAT1	O14929	histone acetyltransferase 1		1.017017269	0.00328855	0.06358946	Up in BrM
16	MYL12B	O14950	myosin light chain 12A	4-[4-(2,5-DIOXO-PYRROLIDIN-1-YL)-PHENYLAMINO]-4-HYDROXY-BUTYRIC ACID	1.342710466	0.00154307	0.05056962	Up in BrM
17	RER1	O15258	retention in endoplasmic reticulum sorting receptor 1		1.205343207	0.00854795	0.08484956	Up in BrM
18	PMM2	O15305	phosphomannomutase 2		2.161020449	0.00117191	0.04625996	Up in BrM
19	HMGB3	O15347	high mobility group box 3		3.071525484	0.00057599	0.03677272	Up in BrM
20	SLC16A3	O15427	solute carrier family 16 member 3	Pyruvic acid	2.093421026	0.00096121	0.04297036	Up in BrM
21	RRP8	O43159	ribosomal RNA processing 8		1.497393412	0.00114959	0.04605447	Up in BrM
22	URB1	O60287	URB1 ribosome biogenesis homolog		1.552490946	0.00744815	0.08003436	Up in BrM
23	PLOD3	O60568	procollagen-lysine,2-oxoglutarate 5-dioxygenase 3	Ascorbic acid	2.193711097	0.00438051	0.06941291	Up in BrM
24	DIAPH1	O60610	diaphanous related formin 1		1.016673854	0.00641549	0.07834571	Up in BrM
25	UGDH	O60701	UDP-glucose 6-dehydrogenase	NADH; Copper	2.347766537	0.00356884	0.06474209	Up in BrM
26	MRPS14	O60783	mitochondrial ribosomal protein S14		1.283627032	0.00663572	0.07906805	Up in BrM
27	EFCAB14	O75071	EF-hand calcium binding domain 14		1.48274901	0.00032404	0.03330738	Up in BrM
28	MRPL33	O75394	mitochondrial ribosomal protein L33		2.275723876	0.00129051	0.04752087	Up in BrM
29	NME6	O75414	NME/NM23 nucleoside diphosphate kinase 6		1.773933074	0.00010322	0.02067526	Up in BrM
30	TECTA	O75443	tectorin alpha		1.124078175	0.00236763	0.0581633	Up in BrM
31	BANF1	O75531	barrier to autointegration nuclear assembly factor 1		1.713701713	0.00629418	0.07752356	Up in BrM
32	NPM3	O75607	nucleophosmin/nucleoplasmin 3		2.490368332	0.00081611	0.04060776	Up in BrM
33	NUP155	O75694	nucleoporin 155		1.091049617	0.00704894	0.07948878	Up in BrM
34	CRTAP	O75718	cartilage associated protein		1.247826836	0.00933344	0.0881655	Up in BrM
35	CPD	O75976	carboxypeptidase D	Guanidinoethylmercaptosuccinic acid	1.992683864	0.00945097	0.08874968	Up in BrM
36	CLPX	O76031	caseinolytic mitochondrial matrix peptidase chaperone subunit X		1.452947209	0.00695693	0.07944195	Up in BrM
37	UNC5C	O95185	unc-5 netrin receptor C		1.116080749	0.00362298	0.06474551	Up in BrM
38	FKBP9	O95302	FKBP prolyl isomerase 9		1.772314268	0.0068571	0.07944195	Up in BrM
39	SVIL	O95425	supervillin		1.672778292	0.00012321	0.02313639	Up in BrM
40	H6PD	O95479	hexose-6-phosphate dehydrogenase/glucose 1-dehydrogenase	NADH	1.394691995	0.00479197	0.07171434	Up in BrM
41	SEC24A	O95486	SEC24 homolog A, COPII coat complex component		1.153765662	0.00692201	0.07944195	Up in BrM
42	KRT75	O95678	keratin 75		4.006357385	0.00045704	0.03510169	Up in BrM
43	STAU1	O95793	staufen double-stranded RNA binding protein 1		1.423309909	0.00156268	0.05065405	Up in BrM

44	NAPSA	O96009	napsin A aspartic peptidase		4.330112323	0.00012632	0.02313639	Up in BrM
45	PLAT	P00750	plasminogen activator, tissue type	Aminocaproic acid; Iloprost; Human C1-esterase inhibitor; 5-(DIMETHYLAMINO)-2-NAPHTHALENESULFONIC ACID; Dexibuprofen; Conestat alfa	1.109301115	0.0000863	0.02036635	Up in BrM
46	LDLR	P01130	low density lipoprotein receptor	Porfimer sodium	1.390326433	0.00105168	0.04393504	Up in BrM
47	TGFB1	P01137	transforming growth factor beta 1	Hyaluronidase (ovine); Terazosin; Hyaluronidase (human recombinant); Foreskin fibroblast (neonatal); Foreskin keratinocyte (neonatal); Hyaluronidase	1.181462226	0.00486425	0.07183273	Up in BrM
48	COL3A1	P02461	collagen type III alpha 1 chain	Collagenase clostridium histolyticum	3.477216127	0.00788764	0.08242517	Up in BrM
49	TFRC	P02786	transferrin receptor	Iron; Ferrous sulfate anhydrous; Ferric cation; Ferrous gluconate; Ferrous succinate; Ferrous ascorbate; Ferrous fumarate; Ferrous glycine sulfate; Tetraferric tricitrate decahydrate; Ferric derisomaltose	1.553384389	0.00101971	0.0435301	Up in BrM
50	RPN2	P04844	ribophorin II		1.892670178	0.00271215	0.05914721	Up in BrM
51	KRT18	P05783	keratin 18		3.016180518	0.00667003	0.07906871	Up in BrM
52	KRT8	P05787	keratin 8	Tenecteplase; Lanoteplase; Copper	3.984752793	0.00900127	0.08661177	Up in BrM
53	COL5A2	P05997	collagen type V alpha 2 chain		1.27014063	0.00640012	0.07834571	Up in BrM
54	GLA	P06280	galactosidase alpha	Migalastat	1.140457601	0.00024627	0.03118214	Up in BrM
55	CDK1	P06493	cyclin dependent kinase 1	Indirubin-3'-monoxime; Olomoucine; Hymenialdisine; SU9516; Alvocidib; Alsterpaullone; Seliciclib; AT-7519; Fostamatinib	2.069489256	0.00176238	0.05118114	Up in BrM
56	P4HB	P07237	prolyl 4-hydroxylase subunit beta	Zinc; Ribostamycin; Copper; Artenimol; Zinc acetate; Zinc chloride; Zinc sulfate, unspecified form	2.201746111	0.00027572	0.03217659	Up in BrM
57	HEXB	P07686	hexosaminidase subunit beta	Pyrimethamine; 2-Acetamido-2-Deoxy-D-Glucono-1,5-Lactone; N-Acetyl-glucosamine thiazoline; (2R,3R,4S,5R)-2-acetamido-3,4-dihydroxy-5-hydroxymethyl-piperidine	1.227162944	0.00612574	0.07737918	Up in BrM
58	GJB1	P08034	gap junction protein beta 1		1.336113934	0.00014351	0.02385308	Up in BrM
59	COL1A2	P08123	collagen type I alpha 2 chain	Collagenase clostridium histolyticum	2.787852023	0.00948136	0.08874968	Up in BrM
60	SRPRA	P08240	SRP receptor subunit alpha		1.166100504	0.005374	0.07550546	Up in BrM
61	ITGA5	P08648	integrin subunit alpha 5	Resveratrol	1.845810655	0.00953759	0.08874968	Up in BrM
62	MRPL3	P09001	mitochondrial ribosomal protein L3		1.889377141	0.00034461	0.03407695	Up in BrM
63	POTEJ	P0CG39	POTE ankyrin domain family member J		1.946726739	0.0000178	0.01742196	Up in BrM
64	SULT1A3	P0DMM9	sulfotransferase family 1A member 3		1.267113303	0.00272129	0.05914721	Up in BrM
65	HSPA5	P11021	heat shock protein family A (Hsp70) member 5	Antihemophilic factor, human recombinant; Acetylsalicylic acid; Copper; Lonoctocog alfa; Moroctocog	1.46933088	0.00206486	0.05505339	Up in BrM
66	IGF2R	P11717	insulin like growth factor 2 receptor	Mecasermin; alpha-D-mannose 6-phosphate; Cerliponase alfa; Mecasermin rinfabate	1.434472209	0.00118199	0.04625996	Up in BrM
67	PCNA	P12004	proliferating cell nuclear antigen	Liothyronine; Acetylsalicylic acid	1.598570094	0.00034247	0.03407695	Up in BrM

68	CDH1	P12830	cadherin 1		3.610148761	0.0000991	0.02036635	Up in BrM
69	PDIA4	P13667	protein disulfide isomerase family A member 4		2.176153176	0.0032549	0.06329383	Up in BrM
70	P4HA1	P13674	prolyl 4-hydroxylase subunit alpha 1	Ascorbic acid; Proline; Hydralazine	2.338593229	0.00020857	0.02909926	Up in BrM
71	CEACAM	P13688	CEA cell adhesion molecule 1	Technetium Tc-99m arcitumomab	2.709699756	0.00510537	0.07322183	Up in BrM
72	HSP90B1	P14625	heat shock protein 90 beta family member 1	Rifabutin; 2-Chlorodideoxyadenosine; Geldanamycin; Diglyme; N-Ethyl-5'-Carboxamido Adenosine; Radicicol; METHYL 3-CHLORO-2-{3-[(2,5-DIHYDROXY-4-METHOXYPHENYL)AMINO]-3-OXOPROPYL}-4,6-DIHYDROXYBENZOATE; 2-(3-AMINO-2,5,6-TRIMETHOXYPHENYL)ETHYL 5-CHLORO-2,4-	1.61094891	0.00302406	0.0614689	Up in BrM
73	JUP	P14923	junction plakoglobin	Zinc; Zinc acetate	2.133733377	0.00305432	0.06165467	Up in BrM
74	ANPEP	P15144	alanyl aminopeptidase, membrane	Ezetimibe; Icatibant	1.873039799	0.003696	0.06474551	Up in BrM
75	CD46	P15529	CD46 molecule		1.857521213	0.00240048	0.05823781	Up in BrM
76	DSP	P15924	desmoplakin	Zinc; Artenimol; Zinc acetate	2.391536168	0.0006941	0.03845173	Up in BrM
77	H1-5	P16401	H1.5 linker histone, cluster member		2.213671761	0.0088786	0.08605411	Up in BrM
78	CD36	P16671	CD36 molecule (CD36 blood group)		1.898530365	0.00230467	0.05752114	Up in BrM
79	YBX3	P16989	Y-box binding protein 3		1.863445843	0.0014455	0.04998058	Up in BrM
80	SDC1	P18827	syndecan 1		2.941720996	0.00088558	0.04142282	Up in BrM
81	NFKB1	P19838	nuclear factor kappa B subunit 1	Pseudoephedrine; Thalidomide; Pranlukast; HE3286; P54; NOX-700; SGN-30; Custirsen; Andrographolide; Triflusal	1.177143968	0.00819518	0.08368724	Up in BrM
82	M6PR	P20645	mannose-6-phosphate receptor, cation dependent	Alglucosidase alfa; 1-3 Sugar Ring of Pentamannosyl 6-Phosphate; alpha-D-mannose 6-phosphate	1.202759319	0.00395605	0.0665948	Up in BrM
83	EFNA1	P20827	ephrin A1		1.495543802	0.00350697	0.06446586	Up in BrM
84	NT5E	P21589	5'-nucleotidase ecto	Pentoxifylline	1.495867116	0.00422304	0.06816941	Up in BrM
85	BGN	P21810	biglycan		2.857436573	0.00696239	0.07944195	Up in BrM
86	TGM2	P21980	transglutaminase 2	Guanosine-5'-Diphosphate; Hexylresorcinol	1.718189468	0.00739179	0.07986812	Up in BrM
87	USF1	P22415	upstream transcription factor 1		1.013887143	0.00601268	0.07737918	Up in BrM
88	PPIB	P23284	peptidylprolyl isomerase B	Proline; 1,4-Dithiothreitol	1.096045659	0.00775845	0.08179349	Up in BrM
89	DTYMK	P23919	deoxythymidylate kinase	Thymidine monophosphate; 2',3'-Dideoxythymidine-5'-Monophosphate; 3'-Fluoro-3'-deoxythymidine 5'-monophosphate; 3'-deoxy-3'-aminothymidine monophosphate; p1-(5'-adenosyl)p5-(5'-thymidyl)pentaphosphate; Zidovudine monophosphate; P1-(5'-Adenosyl)P5-(5'-(3'azido-3'-Deoxythymidyl))Pentaphosphate;	1.294787518	0.00876389	0.08575938	Up in BrM

90	MCM3	P25205	minichromosome maintenance complex component 3		1.917907008	0.0071014	0.07948878	Up in BrM
91	S100P	P25815	S100 calcium binding protein P	Cromoglicic acid	3.214222475	0.00641849	0.07834571	Up in BrM
92	HMGB2	P26583	high mobility group box 2		1.522946258	0.00700018	0.07944195	Up in BrM
93	PTBP1	P26599	polypyrimidine tract binding protein 1		1.739142608	0.00303186	0.0614689	Up in BrM
94	DPP4	P27487	dipeptidyl peptidase 4	[illegible] Methyl-1-Pyrrolidin-1-Yl-Butan-1-One; 5-(Aminomethyl)-6-(2,4-Dichlorophenyl)-2-(3,5-Dimethoxyphenyl)Pyrimidin-4-Amine; (2s)-Pyrrolidin-2-Ylmethylamine; 4-Iodo-L-phenylalanine; Diisopropylphosphono Group; 1-(1-phenylcyclopentyl)methylamine; (S)-2-[(R)-3-amino-4-(2-fluorophenyl)butyryl]-1,2,3,4-tetrahydroisoquinoline-3-carboxamide; Vildagliptin; PSN9301; AMG-222; Bisegliptin; Alogliptin; Saxagliptin; (1S)-2-[(2S,5R)-2-(AMINOMETHYL)-5-PROP-1-YN-1-YLPYRROLIDIN-1-YL]-1-CYCLOPENTYL-2-OXOETHANAMINE; N-(TRANS-4-{(1S,2S)-2-AMINO-3-[(3S)-3-FLUOROPYRROLIDIN-1-YL]-1-METHYL-3-OXOPROPYL}CYCLOHEXYL)-N-METHYLACETAMIDE; (2S,3S)-4-cyclopropyl-3-{(3R,5R)-3-[2-fluoro-4-(methylsulfonyl)phenyl]-1,2,4-oxadiazolidin-5-yl}-1-[(3S)-3-[illegible]	1.922042104	0.0017559	0.05118114	Up in BrM
95	CALR	P27797	calreticulin	Antihemophilic factor, human recombinant; Tenecteplase; Melatonin; Lanoteplase; Copper; Calcium citrate; Calcium Phosphate; Lonoctocog alfa; [illegible]	1.010406546	0.0046867	0.07054432	Up in BrM
96	GRN	P28799	granulin precursor		1.490927301	0.003394	0.06388895	Up in BrM
97	SHC1	P29353	SHC adaptor protein 1		1.549112967	0.00246328	0.05866802	Up in BrM
98	CRABP2	P29373	cellular retinoic acid binding protein 2		2.440888212	0.00862196	0.08520936	Up in BrM
99	RBMS1	P29558	RNA binding motif single stranded interacting protein 1		1.321342935	0.00367289	0.06474551	Up in BrM
100	PML	P29590	PML nuclear body scaffold	Arsenic trioxide	1.23129242	0.00106141	0.04393504	Up in BrM
101	ERP29	P30040	endoplasmic reticulum protein 29		1.113629503	0.00536553	0.07550546	Up in BrM
102	PDIA3	P30101	protein disulfide isomerase family A member 3	Zinc; Copper; Zinc acetate; Zinc chloride; Zinc sulfate, unspecified form	1.290971459	0.00850156	0.08449638	Up in BrM
103	SDC4	P31431	syndecan 4		1.397310771	0.0059219	0.07737918	Up in BrM
104	S100A11	P31949	S100 calcium binding protein A11	Phosphonothreonine	2.519982215	0.00763409	0.08125	Up in BrM
105	PYCR1	P32322	pyrroline-5-carboxylate reductase 1	NADH; Proline	3.660330437	0.00362363	0.06474551	Up in BrM

106	MCM4	P33991	minichromosome maintenance complex component 4		1.977114881	0.00754887	0.08067698	Up in BrM
107	RFC4	P35249	replication factor C subunit 4		1.351036297	0.00550879	0.07563206	Up in BrM
108	MYH9	P35579	myosin heavy chain 9	Artenimol	1.6226335	0.00406135	0.06718394	Up in BrM
109	AHR	P35869	aryl hydrocarbon receptor	Omeprazole; Mexiletine; Nimodipine; Flutamide; Atorvastatin; Leflunomide; Ginseng; Indirubin-3'-monoxime; Resveratrol; Quercetin; beta-Naphthoflavone; Emodin; 1-[(4S)-4-amino-5-(1,3-benzothiazol-2-yl)-5-oxopentyl]guanidine; Diosmin; Indigotindisulfonic acid; Kynurenic Acid; Epigallocatechin gallate;	1.121099713	0.00174637	0.051096	Up in BrM
110	NUP62	P37198	nucleoporin 62		1.141735493	0.00616992	0.07737918	Up in BrM
111	GGCX	P38435	gamma-glutamyl carboxylase	Coagulation factor VIIa Recombinant Human; Coagulation Factor IX (Recombinant); Glutamic acid; Menadione; Phylloquinone; Anisindione; Kappadione; Coagulation	1.706963255	0.00350567	0.06446586	Up in BrM
112	DDOST	P39656	dolichyl-diphosphooligosaccharide--protein glycosyltransferase non-catalytic subunit		1.883562739	0.00192275	0.05326439	Up in BrM
113	CEACAM	P40199	CEA cell adhesion molecule 6		3.948875345	0.00054213	0.03619764	Up in BrM
114	RPL13A	P40429	ribosomal protein L13a	(S)-3-phenyllactic acid; Anisomycin; Puromycin	1.101959062	0.00493588	0.07261599	Up in BrM
115	ECE1	P42892	endothelin converting enzyme 1	5-(2-hydroxyethyl)nonane-1,9-diol	1.599748744	0.00727584	0.07967639	Up in BrM
116	SSR1	P43307	signal sequence receptor subunit 1		1.967371978	0.00046583	0.03510169	Up in BrM
117	NOP2	P46087	NOP2 nucleolar protein		2.451461418	0.0003896	0.03478999	Up in BrM
118	CD151	P48509	CD151 molecule (Raph blood group)		1.286879962	0.00665372	0.07906871	Up in BrM
119	LMAN1	P49257	lectin, mannose binding 1	Antihemophilic factor, human recombinant; Lonoctocog alfa;	1.471744156	0.00973204	0.08970571	Up in BrM
120	PCYT1A	P49585	phosphate cytidylyltransferase 1A, choline	Choline; Choline salicylate	1.126697485	0.00540743	0.07550546	Up in BrM
121	HARS2	P49590	histidyl-tRNA synthetase 2, mitochondrial		1.571750954	0.00057899	0.03677272	Up in BrM
122	CTCF	P49711	CCCTC-binding factor		1.312582228	0.00649906	0.07883643	Up in BrM
123	TMED10	P49755	transmembrane p24 trafficking protein 10		1.536247196	0.00990743	0.09052266	Up in BrM
124	MTHFS	P49914	methenyltetrahydrofolate synthetase		1.502369939	0.00124272	0.04708429	Up in BrM
125	MMP14	P50281	matrix metallopeptidase 14	Marimastat	1.700449072	0.00925153	0.08781643	Up in BrM
126	SERPINH	P50454	serpin family H member 1		3.043131285	0.00339104	0.06388895	Up in BrM
127	VASP	P50552	vasodilator stimulated phosphoprotein		1.408880269	0.00522358	0.07405923	Up in BrM
128	DAP	P51397	death associated protein		1.354789298	0.00548652	0.07563206	Up in BrM
129	LUM	P51884	lumican	Copper	2.866980264	0.00413367	0.06769856	Up in BrM
130	NUP98	P52948	nucleoporin 98 and 96 precursor		1.043708408	0.00600609	0.07737918	Up in BrM

131	METAP1	P53582	methionyl aminopeptidase 1	Ovalicin; 5-CHLORO-6-METHYL-N-(2-PHENYLETHYL)-2-PYRIDIN-2-YLPYRIMIDIN-4-AMINE; TERT-BUTYL {2-[(1,3-THIAZOL-2-YLAMINO)CARBONYL]PYRIDIN-3-YL}CARBAMATE; 3-[(2,2-DIMETHYLPROPANOYL)AMINO]-N-1,3-THIAZOL-2-YLPYRIDINE-2-CARBOXAMIDE	1.07532871	0.00928342	0.08800402	Up in BrM
132	ARSF	P54793	arylsulfatase F		1.505572757	0.00782704	0.08223722	Up in BrM
133	AK2	P54819	adenylate kinase 2	Bis(Adenosine)-5'-Pentaphosphate; Imidazole	1.648071431	0.00499081	0.07268088	Up in BrM
134	MANF	P55145	mesencephalic astrocyte derived neurotrophic factor		1.454275337	0.00793435	0.08270416	Up in BrM
135	BID	P55957	BH3 interacting domain death agonist		1.157177864	0.00158361	0.05084199	Up in BrM
136	RRP1	P56182	ribosomal RNA processing 1		1.243371028	0.00265266	0.05914721	Up in BrM
137	ANTXR2	P58335	ANTXR cell adhesion molecule 2	MDX-1303	1.811491511	0.00163545	0.05084199	Up in BrM
138	SEC61B	P60468	SEC61 translocon subunit beta		1.463763627	0.0067406	0.07938502	Up in BrM
139	ROMO1	P60602	reactive oxygen species modulator 1		1.385424144	0.00071796	0.03845173	Up in BrM
140	MYL6	P60660	myosin light chain 6		1.181496859	0.00585549	0.07737918	Up in BrM
141	DAD1	P61803	defender against cell death 1		1.748077462	0.00163203	0.05084199	Up in BrM
142	RPL30	P62888	ribosomal protein L30		1.093910065	0.00087471	0.04142282	Up in BrM
143	SUPT4H1	P63272	SPT4 homolog, DSIF elongation factor subunit		1.11860324	0.00702769	0.07945048	Up in BrM
144	SEC11A	P67812	SEC11 homolog A, signal peptidase complex subunit		2.249349802	0.00075073	0.03883914	Up in BrM
145	MRPS22	P82650	mitochondrial ribosomal protein S22		1.482249757	0.00716444	0.0795008	Up in BrM
146	MRPS10	P82664	mitochondrial ribosomal protein S10		1.613372277	0.0002086	0.02909926	Up in BrM
147	MRPS5	P82675	mitochondrial ribosomal protein S5		1.256252388	0.00314828	0.06258099	Up in BrM
148	MRPS21	P82921	mitochondrial ribosomal protein S21		1.598530622	0.00013885	0.02363612	Up in BrM
149	MRPS6	P82932	mitochondrial ribosomal protein S6		1.057799939	0.00836474	0.0838895	Up in BrM
150	MRPS9	P82933	mitochondrial ribosomal protein S9		1.461586424	0.00164803	0.05084199	Up in BrM
151	SP3	Q02447	Sp3 transcription factor		2.046876185	0.0060184	0.07737918	Up in BrM
152	PLOD1	Q02809	procollagen-lysine,2-oxoglutarate 5-dioxygenase 1	Ascorbic acid	1.852154851	0.00211115	0.05549443	Up in BrM
153	PTPN12	Q05209	protein tyrosine phosphatase non-receptor type 12	Tiludronic acid	1.411261929	0.00266235	0.05914721	Up in BrM
154	CKAP4	Q07065	cytoskeleton associated protein 4		2.489468326	0.00055144	0.03650707	Up in BrM
155	ENPEP	Q07075	glutamyl aminopeptidase	Glutamic acid	1.815977723	0.00435913	0.06935551	Up in BrM
156	GALNT1	Q10472	polypeptide N-acetylgalactosaminyltransferase 1		1.819488116	0.00093672	0.04260411	Up in BrM
157	NUP160	Q12769	nucleoporin 160		1.392422278	0.00096917	0.04297036	Up in BrM
158	TBL3	Q12788	transducin beta like 3		1.529342402	0.00159584	0.05084199	Up in BrM
159	LMAN2	Q12907	lectin, mannose binding 2		1.912440726	0.00045414	0.03510169	Up in BrM
160	EPS8	Q12929	EGFR pathway substrate 8, signaling adaptor		1.094011528	0.00838681	0.0838895	Up in BrM
161	MRPL28	Q13084	mitochondrial ribosomal protein L28		1.809318648	0.0000307	0.01997811	Up in BrM
162	PRDX4	Q13162	peroxiredoxin 4		1.671483532	0.00347593	0.0643459	Up in BrM
163	DNAJC3	Q13217	DnaJ heat shock protein family (Hsp40) member C3		1.170368815	0.00430371	0.06889469	Up in BrM
164	STX3	Q13277	syntaxin 3		1.691529625	0.00028828	0.03217659	Up in BrM
165	PTK7	Q13308	protein tyrosine kinase 7 (inactive)		2.486846858	0.00064265	0.03845173	Up in BrM
166	OS9	Q13438	OS9 endoplasmic reticulum lectin		1.233076837	0.0071023	0.07948878	Up in BrM
167	ARFRP1	Q13795	ADP ribosylation factor related protein 1		1.819156619	0.0000948	0.02036635	Up in BrM
168	BYSL	Q13895	bystin like		1.848481044	0.00173344	0.05100392	Up in BrM
169	DSG2	Q14126	desmoglein 2		2.777296525	0.00000329	0.01358434	Up in BrM
170	MLEC	Q14165	malectin		1.225494437	0.00481892	0.07178313	Up in BrM

171	ZMYM3	Q14202	zinc finger MYM-type containing 3		1.308655054	0.00578683	0.07727637	Up in BrM
172	ENDOG	Q14249	endonuclease G		1.461607892	0.00825751	0.08381865	Up in BrM
173	HLTF	Q14527	helicase like transcription factor		1.12450213	0.00460742	0.06997177	Up in BrM
174	SQLE	Q14534	squalene epoxidase	Naftifine; Terbinafine; Butenafine; Ellagic acid	1.134121779	0.00858608	0.08506302	Up in BrM
175	PDIA5	Q14554	protein disulfide isomerase family A member 5		1.828878147	0.00265782	0.05914721	Up in BrM
176	BMS1	Q14692	BMS1 ribosome biogenesis factor		1.122147972	0.00803735	0.08316259	Up in BrM
177	SPCS2	Q15005	signal peptidase complex subunit 2		1.432962203	0.00815566	0.08339268	Up in BrM
178	SUZ12	Q15022	SUZ12 polycomb repressive complex 2 subunit		1.367779717	0.00049705	0.03562344	Up in BrM
179	RRS1	Q15050	ribosome biogenesis regulator 1 homolog		2.154632708	0.00019781	0.02861609	Up in BrM
180	POSTN	Q15063	periostin		3.15555513	0.00078982	0.03985156	Up in BrM
181	OXA1L	Q15070	OXA1L mitochondrial inner membrane protein		1.135565093	0.00170349	0.05086288	Up in BrM
182	PDIA6	Q15084	protein disulfide isomerase family A member 6		1.82234787	0.00167825	0.05086288	Up in BrM
183	TMED2	Q15363	transmembrane p24 trafficking protein 2		1.226098998	0.00910442	0.08695736	Up in BrM
184	TCEA2	Q15560	transcription elongation factor A2		1.719260389	0.00067273	0.03845173	Up in BrM
185	TGFBI	Q15582	transforming growth factor beta induced		2.769347928	0.006906	0.07944195	Up in BrM
186	SLC1A5	Q15758	solute carrier family 1 member 5	Asparagine; Fluciclovine (18F)	3.218290382	0.00369643	0.06474551	Up in BrM
187	OCLN	Q16625	occludin		1.361881097	0.00612252	0.07737918	Up in BrM
188	CYP51A1	Q16850	cytochrome P450 family 51 subfamily A member 1	Tioconazole; Itraconazole; Levoketoconazole; (S)-econazole	1.265702864	0.008833	0.08594982	Up in BrM
189	PDS5A	Q29RF7	PDS5 cohesin associated factor A		1.329463618	0.00691095	0.07944195	Up in BrM
190	TSR1	Q2NL82	TSR1 ribosome maturation factor		1.131522696	0.00403299	0.06703339	Up in BrM
191	LARP7	Q4G0J3	La ribonucleoprotein 7, transcriptional regulator		1.11171972	0.00382604	0.06614708	Up in BrM
192	OCIAD2	Q56VL3	OCIA domain containing 2		2.703519593	0.00169937	0.05086288	Up in BrM
193	SFT2D3	Q587I9	SFT2 domain containing 3		2.42881298	0.00044386	0.03510169	Up in BrM
194	NAALAD L2	Q58DX5	N-acetylated alpha-linked acidic dipeptidase like 2		1.373786433	0.00614522	0.07737918	Up in BrM
195	TMEM41	Q5BJD5	transmembrane protein 41B		1.454927699	0.00836802	0.0838895	Up in BrM
196	TOR1AIP1	Q5JTV8	torsin 1A interacting protein 1		1.39075191	0.0000772	0.02036635	Up in BrM
197	NOL9	Q5SY16	nucleolar protein 9		1.679870055	0.00801625	0.08316259	Up in BrM
198	CEP85L	Q5SZL2	centrosomal protein 85 like		1.493789758	0.00214132	0.05549443	Up in BrM
199	ATAD3C	Q5T2N8	ATPase family AAA domain containing 3C		2.444871632	0.00227766	0.05721257	Up in BrM
200	IBA57	Q5T440	iron-sulfur cluster assembly factor IBA57		1.452558087	0.00505411	0.07290812	Up in BrM
201	ANKRD22	Q5VYY1	ankyrin repeat domain 22		3.026537828	0.00261937	0.05914721	Up in BrM
202	KRT79	Q5XKE5	keratin 79		4.77191069	0.0009736	0.04297036	Up in BrM
203	CD276	Q5ZPR3	CD276 molecule		1.688063981	0.00098033	0.04302448	Up in BrM
204	RPL7L1	Q6DKI1	ribosomal protein L7 like 1		1.209838592	0.00693698	0.07944195	Up in BrM
205	SLC25A24	Q6NUK1	solute carrier family 25 member 24		1.652286771	0.00238679	0.05823781	Up in BrM
206	ZCCHC8	Q6NZY4	zinc finger CCHC-type containing 8		1.082447299	0.00290869	0.06043263	Up in BrM
207	MRPL54	Q6P161	mitochondrial ribosomal protein L54		2.072500914	0.0000891	0.02036635	Up in BrM
208	PDXDC1	Q6P996	pyridoxal dependent decarboxylase domain containing 1		1.266774367	0.00930183	0.08800402	Up in BrM
209	WDR74	Q6RFH5	WD repeat domain 74		1.48995837	0.00068523	0.03845173	Up in BrM
210	SAMD1	Q6SPF0	sterile alpha motif domain containing 1		1.685433309	0.0046862	0.07054432	Up in BrM
211	CSPG4	Q6UVK1	chondroitin sulfate proteoglycan 4		1.225804789	0.0055057	0.07563206	Up in BrM
212	NPNT	Q6UXI9	nephronectin		1.155128143	0.00227054	0.05721257	Up in BrM
213	IKBIP	Q70UQ0	IKBKB interacting protein		1.471896892	0.00833912	0.0838895	Up in BrM
214	PARS2	Q7L3T8	prolyl-tRNA synthetase 2, mitochondrial	Proline	1.363907294	0.00342383	0.06414144	Up in BrM
215	HS2ST1	Q7LGA3	heparan sulfate 2-O-sulfotransferase 1		1.343474882	0.00768999	0.08162259	Up in BrM
216	SLC36A1	Q7Z2H8	solute carrier family 36 member 1	Alanine	1.135924988	0.00461284	0.06997177	Up in BrM

217	MRPL21	Q7Z2W9	mitochondrial ribosomal protein L21		1.110489498	0.00773505	0.08165707	Up in BrM
218	MRPL10	Q7Z7H8	mitochondrial ribosomal protein L10		1.799233189	0.00043316	0.03510169	Up in BrM
219	GALNT5	Q7Z7M9	polypeptide N-acetylgalactosaminyltransferase 5		1.350507335	0.00356393	0.06474209	Up in BrM
220	TMEM179B	Q7Z7N9	transmembrane protein 179B		1.289068134	0.0026889	0.05914721	Up in BrM
221	MRPL52	Q86TS9	mitochondrial ribosomal protein L52		1.402125668	0.00170974	0.05086288	Up in BrM
222	PPFIBP1	Q86W92	PPFIA binding protein 1		1.165356123	0.0070579	0.07948878	Up in BrM
223	PTRH1	Q86Y79	peptidyl-tRNA hydrolase 1 homolog		1.455111303	0.00638543	0.07834571	Up in BrM
224	AEBP1	Q8IUX7	AE binding protein 1		1.622841406	0.00321514	0.06316325	Up in BrM
225	FAM114A	Q8IWE2	family with sequence similarity 114 member A1		1.433195763	0.00169463	0.05086288	Up in BrM
226	MRPL41	Q8IXM3	mitochondrial ribosomal protein L41		1.403407143	0.00733516	0.07967639	Up in BrM
227	NRM	Q8IXM6	nurim		1.303303433	0.00086795	0.04142282	Up in BrM
228	FTSJ3	Q8IY81	FtsJ RNA 2'-O-methyltransferase 3		1.345233882	0.00657858	0.07906805	Up in BrM
229	ELMOD2	Q8IZ81	ELMO domain containing 2		1.272435225	0.00499177	0.07268088	Up in BrM
230	PELP1	Q8IZL8	proline, glutamate and leucine rich protein 1		1.206347209	0.00875922	0.08575938	Up in BrM
231	ABCA7	Q8IZY2	ATP binding cassette subfamily A member 7		1.769294465	0.00335624	0.06387132	Up in BrM
232	GALNT4	Q8N4A0	polypeptide N-acetylgalactosaminyltransferase 4		1.543220487	0.00910537	0.08695736	Up in BrM
233	ATPAF2	Q8N5M1	ATP synthase mitochondrial F1 complex assembly factor 2		1.000888662	0.0022155	0.05674587	Up in BrM
234	KRTCAP2	Q8N6L1	keratinocyte associated protein 2		2.124633244	0.00146977	0.04998058	Up in BrM
235	DDX51	Q8N8A6	DEAD-box helicase 51		1.235163031	0.00028132	0.03217659	Up in BrM
236	PDCD7	Q8N8D1	programmed cell death 7		1.089370218	0.00112611	0.04558136	Up in BrM
237	MRPL43	Q8N983	mitochondrial ribosomal protein L43		1.934080626	0.00280114	0.05995202	Up in BrM
238	KRI1	Q8N9T8	KRI1 homolog		1.51224012	0.00022603	0.03097849	Up in BrM
239	GOLM1	Q8NBJ4	golgi membrane protein 1		1.968840498	0.00187921	0.05289456	Up in BrM
240	COLGALT1	Q8NBJ5	collagen beta(1-O)galactosyltransferase 1		1.786573929	0.00985685	0.09046538	Up in BrM
241	SIDT2	Q8NBJ9	SID1 transmembrane family member 2		1.009934439	0.00696311	0.07944195	Up in BrM
242	TTC13	Q8NBP0	tetratricopeptide repeat domain 13		1.111201823	0.00601176	0.07737918	Up in BrM
243	TXNDC5	Q8NBS9	thioredoxin domain containing 5		1.944562684	0.0068464	0.07944195	Up in BrM
244	UXS1	Q8NBZ7	UDP-glucuronate decarboxylase 1		1.38855252	0.00190832	0.05318988	Up in BrM
245	TMEM104	Q8NE00	transmembrane protein 104		1.785519161	0.009677	0.08957202	Up in BrM
246	NUP43	Q8NFH3	nucleoporin 43		1.361934618	0.00191326	0.05318988	Up in BrM
247	WDR36	Q8NI36	WD repeat domain 36		1.750829803	0.00037124	0.03456622	Up in BrM
248	SLC35B2	Q8TB61	solute carrier family 35 member B2		2.140650097	0.0000271	0.01997811	Up in BrM
249	SLC25A40	Q8TBP6	solute carrier family 25 member 40		1.357406203	0.00119071	0.04625996	Up in BrM
250	TMEM167A	Q8TBQ9	transmembrane protein 167A		1.228524381	0.00559478	0.07575872	Up in BrM
251	STT3B	Q8TCJ2	STT3 oligosaccharyltransferase complex catalytic subunit B		1.378193807	0.00226414	0.05721257	Up in BrM
252	HM13	Q8TCT9	histocompatibility minor 13	Theophylline	2.060639009	0.00149053	0.04998058	Up in BrM
253	BLOC1S5	Q8TDH9	biogenesis of lysosomal organelles complex 1 subunit 5		1.148114275	0.00729075	0.07967639	Up in BrM
254	GPX8	Q8TED1	glutathione peroxidase 8 (putative)		2.067649294	0.0021657	0.05583658	Up in BrM
255	NOC3L	Q8WTT2	NOC3 like DNA replication regulator		1.640157987	0.00254146	0.05914721	Up in BrM
256	NUDT8	Q8WV74	nudix hydrolase 8		1.548046305	0.00405684	0.06718394	Up in BrM
257	LEO1	Q8WVC0	LEO1 homolog, Paf1/RNA polymerase II complex component		1.015259571	0.00719053	0.07967639	Up in BrM
258	TFB1M	Q8WVM0	transcription factor B1, mitochondrial		1.648178293	0.00036522	0.03456622	Up in BrM
259	HSD17B8	Q92506	hydroxysteroid 17-beta dehydrogenase 8	NADH; Nicotinamide adenine dinucleotide phosphate	1.262009267	0.00923345	0.087774	Up in BrM
260	ANP32B	Q92688	acidic nuclear phosphoprotein 32 family member B		1.682415203	0.00139222	0.04971197	Up in BrM
261	DPF2	Q92785	double PHD fingers 2		1.855325986	0.00782411	0.08223722	Up in BrM

262	TOP1MT	Q969P6	DNA topoisomerase I mitochondrial	Irinotecan; Topotecan	1.69435642	0.00042315	0.03510169	Up in BrM
263	UTP4	Q969X6	UTP4 small subunit processome component		1.4917316	0.00383759	0.06614708	Up in BrM
264	MRPL24	Q96A35	mitochondrial ribosomal protein L24		1.619896048	0.00024748	0.03118214	Up in BrM
265	TTC17	Q96AE7	tetratricopeptide repeat domain 17		1.895395858	0.000043	0.02036635	Up in BrM
266	FKBP10	Q96AY3	FKBP prolyl isomerase 10		1.78718435	0.00836685	0.0838895	Up in BrM
267	FAM136A	Q96C01	family with sequence similarity 136 member A		1.028977315	0.00715093	0.0795008	Up in BrM
268	PYCR2	Q96C36	pyrroline-5-carboxylate reductase 2	NADH; Proline	2.393532311	0.00770466	0.08165707	Up in BrM
269	FOXRED1	Q96CU9	FAD dependent oxidoreductase domain containing 1		1.44053096	0.00450593	0.06977687	Up in BrM
270	MALSU1	Q96EH3	mitochondrial assembly of ribosomal large subunit 1		1.76444854	0.00414439	0.06770899	Up in BrM
271	MRPL53	Q96EL3	mitochondrial ribosomal protein L53		1.637812139	0.0000983	0.02036635	Up in BrM
272	C1GALT1C1	Q96EU7	C1GALT1 specific chaperone 1		1.908194687	0.00015906	0.0249558	Up in BrM
273	DTNBP1	Q96EV8	dystrobrevin binding protein 1		1.141475762	0.00679502	0.07944195	Up in BrM
274	PTCD3	Q96EY7	pentatricopeptide repeat domain 3		1.398558018	0.00383279	0.06614708	Up in BrM
275	IMP4	Q96G21	IMP U3 small nucleolar ribonucleoprotein 4		1.297491409	0.00297295	0.06111763	Up in BrM
276	DUS3L	Q96G46	dihydrouridine synthase 3 like		1.473031777	0.00135757	0.04909861	Up in BrM
277	MRPL48	Q96GC5	mitochondrial ribosomal protein L48		2.000168163	0.00030277	0.03255968	Up in BrM
278	DDX27	Q96GQ7	DEAD-box helicase 27		1.345440702	0.00700659	0.07944195	Up in BrM
279	MARS2	Q96GW9	methionyl-tRNA synthetase 2, mitochondrial		1.561160204	0.00367514	0.06474551	Up in BrM
280	ERO1A	Q96HE7	endoplasmic reticulum oxidoreductase 1 alpha		2.309024458	0.00255475	0.05914721	Up in BrM
281	FMC1	Q96HJ9	formation of mitochondrial complex V assembly factor 1 homolog		1.714194725	0.0025764	0.05914721	Up in BrM
282	MED30	Q96HR3	mediator complex subunit 30		1.063888643	0.00042084	0.03510169	Up in BrM
283	TMEM41	Q96HV5	transmembrane protein 41A		1.66733806	0.00213738	0.05549443	Up in BrM
284	RCC1L	Q96I51	RCC1 like		1.484658279	0.00398955	0.0665948	Up in BrM
285	NARS2	Q96I59	asparaginyl-tRNA synthetase 2, mitochondrial	Asparagine	1.414344015	0.00592518	0.07737918	Up in BrM
286	CNOT6L	Q96LI5	CCR4-NOT transcription complex subunit 6 like		1.206316516	0.0016661	0.05084199	Up in BrM
287	ERGIC2	Q96RQ1	ERGIC and golgi 2		1.374347642	0.000077	0.02036635	Up in BrM
288	TMEM209	Q96SK2	transmembrane protein 209		1.363310062	0.00953967	0.08874968	Up in BrM
289	CNN2	Q99439	calponin 2		2.037988651	0.00549549	0.07563206	Up in BrM
290	NEU1	Q99519	neuraminidase 1	Oseltamivir	2.187632976	0.00052282	0.03614653	Up in BrM
291	SCAF11	Q99590	SR-related CTD associated factor 11		1.593431248	0.00504558	0.07290812	Up in BrM
292	TM9SF2	Q99805	transmembrane 9 superfamily member 2		1.48663551	0.00161774	0.05084199	Up in BrM
293	CPNE1	Q99829	copine 1	Theophylline	1.297840345	0.00724649	0.07967639	Up in BrM
294	VRK1	Q99986	VRK serine/threonine kinase 1		1.080092457	0.00153193	0.05056962	Up in BrM
295	MRPL57	Q9BQC6	mitochondrial ribosomal protein L57		1.285194088	0.00434204	0.06922449	Up in BrM
296	SELENOS	Q9BQE4	selenoprotein S		1.539134625	0.0000663	0.02036635	Up in BrM
297	MRPL45	Q9BRJ2	None	None	2.049783926	0.000089	0.02036635	Up in BrM
298	C7orf50	Q9BRJ6	chromosome 7 open reading frame 50		1.746417887	0.00960974	0.08916454	Up in BrM
299	SDF4	Q9BRK5	stromal cell derived factor 4		1.33081794	0.00139998	0.04971197	Up in BrM
300	ADPGK	Q9BRR6	ADP dependent glucokinase		1.101110209	0.00593971	0.07737918	Up in BrM
301	LLPH	Q9BRT6	LLP homolog, long-term synaptic facilitation factor		2.333171331	0.00040753	0.03510169	Up in BrM
302	ERP44	Q9BS26	endoplasmic reticulum protein 44		1.453050843	0.00223888	0.05701648	Up in BrM
303	NOL10	Q9BSC4	nucleolar protein 10		2.254976179	0.00171915	0.05087128	Up in BrM
304	PRADC1	Q9BSG0	protease associated domain containing 1		1.106797793	0.00601605	0.07737918	Up in BrM
305	TSEN34	Q9BSV6	tRNA splicing endonuclease subunit 34		1.084703826	0.00428719	0.06877108	Up in BrM
306	TMEM43	Q9BTV4	transmembrane protein 43		1.387385361	0.00587652	0.07737918	Up in BrM
307	MED18	Q9BUE0	mediator complex subunit 18		1.064389792	0.00024463	0.03118214	Up in BrM
308	HTATIP2	Q9BUP3	HIV-1 Tat interactive protein 2	Polyethylene glycol 400	1.283582051	0.00164898	0.05084199	Up in BrM
309	VAMP8	Q9BV40	vesicle associated membrane protein 8		1.881970638	0.00318129	0.06307664	Up in BrM

310	TMEM109	Q9BVC6	transmembrane protein 109		1.646862627	0.00901374	0.08661177	Up in BrM
311	TMED9	Q9BVK6	transmembrane p24 trafficking protein 9		1.827650866	0.0020175	0.05435962	Up in BrM
312	NUP58	Q9BVL2	nucleoporin 58		1.408843459	0.00067892	0.03845173	Up in BrM
313	TARS2	Q9BW92	threonyl-tRNA synthetase 2, mitochondrial	Threonine	2.030433723	0.00050571	0.03591423	Up in BrM
314	MRPL20	Q9BYC9	mitochondrial ribosomal protein L20		2.006175405	0.0000977	0.02036635	Up in BrM
315	NIFK	Q9BYG3	nucleolar protein interacting with the FHA domain of MKI67		1.500276755	0.00498829	0.07268088	Up in BrM
316	UACA	Q9BZF9	uveal autoantigen with coiled-coil domains and ankyrin repeats		1.495297797	0.00288308	0.06038225	Up in BrM
317	PRKD2	Q9BZL6	protein kinase D2		1.995408424	0.00281505	0.06004885	Up in BrM
318	EDEM3	Q9BZQ6	ER degradation enhancing alpha-mannosidase like protein 3		2.182729261	0.00147663	0.04998058	Up in BrM
319	WDR12	Q9GZL7	WD repeat domain 12		1.520651053	0.00265176	0.05914721	Up in BrM
320	YIPF3	Q9GZM5	Yip1 domain family member 3		1.381825885	0.00070006	0.03845173	Up in BrM
321	DERL2	Q9GZP9	derlin 2		1.463992013	0.00982275	0.09029444	Up in BrM
322	REXO4	Q9GZR2	REX4 homolog, 3'-5' exonuclease		2.793538519	0.0004673	0.03510169	Up in BrM
323	NAT10	Q9H0A0	N-acetyltransferase 10		1.663452499	0.00388714	0.06632907	Up in BrM
324	NT5C3A	Q9H0P0	5'-nucleotidase, cytosolic IIIA		1.402127799	0.00373897	0.06534408	Up in BrM
325	MAGT1	Q9H0U3	magnesium transporter 1		1.181341384	0.00809977	0.08316689	Up in BrM
326	MRPL18	Q9H0U6	mitochondrial ribosomal protein L18		1.617853962	0.0003608	0.03456622	Up in BrM
327	SIL1	Q9H173	SIL1 nucleotide exchange factor		2.012183118	0.00122354	0.04685441	Up in BrM
328	SPNS1	Q9H2V7	SPNS lysolipid transporter 1, lysophospholipid		1.081855489	0.00423249	0.0681737	Up in BrM
329	GHITM	Q9H3K2	growth hormone inducible transmembrane protein		1.03949681	0.00595037	0.07737918	Up in BrM
330	TXNIP	Q9H3M7	thioredoxin interacting protein		1.22506016	0.0013973	0.04971197	Up in BrM
331	POFUT1	Q9H488	protein O-fucosyltransferase 1		1.454396014	0.00345763	0.06431193	Up in BrM
332	CCDC86	Q9H6F5	coiled-coil domain containing 86		2.182141501	0.000072	0.02036635	Up in BrM
333	SP140L	Q9H930	SP140 nuclear body protein like		2.031168923	0.00474263	0.0711122	Up in BrM
334	PSTPIP2	Q9H939	proline-serine-threonine phosphatase interacting protein 2		1.372492369	0.00362853	0.06474551	Up in BrM
335	QTRT2	Q9H974	queuine tRNA-ribosyltransferase accessory subunit 2		1.394070197	0.00185395	0.05266557	Up in BrM
336	MRPL44	Q9H9J2	mitochondrial ribosomal protein L44		1.800966312	0.00057211	0.03677272	Up in BrM
337	SLC38A10	Q9HBR0	solute carrier family 38 member 10		1.875404566	0.00337671	0.06387132	Up in BrM
338	SLC25A19	Q9HC21	solute carrier family 25 member 19		1.530760611	0.00086583	0.04142282	Up in BrM
339	EML4	Q9HC35	EMAP like 4		1.85866205	0.00119617	0.04625996	Up in BrM
340	SDF2L1	Q9HCN8	stromal cell derived factor 2 like 1		1.350503515	0.00131653	0.04805952	Up in BrM
341	S100A14	Q9HCY8	S100 calcium binding protein A14		3.876279469	0.000047	0.02036635	Up in BrM
342	MRPL47	Q9HD33	mitochondrial ribosomal protein L47		1.92127055	0.00039253	0.03478999	Up in BrM
343	NOP10	Q9NPE3	NOP10 ribonucleoprotein		1.453914765	0.00180277	0.05179296	Up in BrM
344	GPR108	Q9NPR9	G protein-coupled receptor 108		1.739840042	0.00256237	0.05914721	Up in BrM
345	XPNPEP3	Q9NQH7	X-prolyl aminopeptidase 3		2.038292087	0.00282104	0.06004885	Up in BrM
346	AAAS	Q9NRG9	aladin WD repeat nucleoporin		1.19919914	0.0003166	0.03300736	Up in BrM
347	ABCB10	Q9NRK6	ATP binding cassette subfamily B member 10		1.120966007	0.00293493	0.06065515	Up in BrM
348	OLFML3	Q9NRN5	olfactomedin like 3		2.302054477	0.00000398	0.01358434	Up in BrM
349	NDUFA4L 2	Q9NRX3	NDUFA4 mitochondrial complex associated like 2	NADH	1.140887246	0.00610986	0.07737918	Up in BrM
350	DDX28	Q9NUL7	DEAD-box helicase 28		2.304775476	0.0000947	0.02036635	Up in BrM
351	TXLNG	Q9NUQ3	taxilin gamma		1.126219636	0.00057571	0.03677272	Up in BrM
352	EVA1B	Q9NVM1	eva-1 homolog B		1.104159001	0.00188214	0.05289456	Up in BrM
353	NLE1	Q9NVX2	notchless homolog 1		1.410927269	0.00148788	0.04998058	Up in BrM
354	MRPL22	Q9NWU5	mitochondrial ribosomal protein L22		1.440271559	0.00118369	0.04625996	Up in BrM
355	MRPL16	Q9NX20	mitochondrial ribosomal protein L16		1.370611388	0.00967726	0.08957202	Up in BrM
356	QPCTL	Q9NXS2	glutaminyl-peptide cyclotransferase like		1.31997267	0.00610475	0.07737918	Up in BrM
357	GAR1	Q9NY12	GAR1 ribonucleoprotein		1.818807041	0.00730199	0.07967639	Up in BrM

358	DDX56	Q9NY93	DEAD-box helicase 56		1.620968373	0.00454492	0.06977687	Up in BrM
359	B3GNT2	Q9NY97	UDP-GlcNAc:betaGal beta-1,3-N-acetylglucosaminyltransferase 2		1.515941053	0.00557955	0.07575872	Up in BrM
360	UTP6	Q9NYH9	UTP6 small subunit processome component		1.244014681	0.00810164	0.08316689	Up in BrM
361	FKBP11	Q9NYL4	FKBP prolyl isomerase 11		1.856731186	0.00397573	0.0665948	Up in BrM
362	TMOD3	Q9NYL9	tropomodulin 3		1.317167723	0.00072586	0.03845173	Up in BrM
363	BET1L	Q9NYM9	Bet1 golgi vesicular membrane trafficking protein like		2.053522585	0.0000296	0.01997811	Up in BrM
364	UGGT1	Q9NYU2	UDP-glucose glycoprotein glucosyltransferase 1		1.08964116	0.00420482	0.06814947	Up in BrM
365	SELENON	Q9NZV5	selenoprotein N		1.16898993	0.00071537	0.03845173	Up in BrM
366	MRPL15	Q9P015	mitochondrial ribosomal protein L15		1.6236815	0.00255212	0.05914721	Up in BrM
367	COX16	Q9P0S2	cytochrome c oxidase assembly factor COX16		1.181500186	0.00297291	0.06111763	Up in BrM
368	TXNDC16	Q9P2K2	thioredoxin domain containing 16		1.490327811	0.0000698	0.02036635	Up in BrM
369	RBM27	Q9P2N5	RNA binding motif protein 27		1.246325305	0.00849717	0.08449638	Up in BrM
370	UXT	Q9UBK9	ubiquitously expressed prefoldin like chaperone		1.043957738	0.00995721	0.09087116	Up in BrM
371	DNAJB11	Q9UBS4	DnaJ heat shock protein family (Hsp40) member B11		1.513730466	0.00057025	0.03677272	Up in BrM
372	SLC25A10	Q9UBX3	solute carrier family 25 member 10		2.495287187	0.00037611	0.03456622	Up in BrM
373	TES	Q9UGI8	testin LIM domain protein		1.845151935	0.00063118	0.03845173	Up in BrM
374	CEMIP2	Q9UHN6	cell migration inducing hyaluronidase 2		1.711510533	0.00233579	0.05792761	Up in BrM
375	MRTO4	Q9UKD2	MRT4 homolog, ribosome maturation factor		1.784512193	0.00145641	0.04998058	Up in BrM
376	ERG28	Q9UKR5	ergosterol biosynthesis 28 homolog		1.649922485	0.00017369	0.02608668	Up in BrM
377	CCPG1	Q9ULG6	cell cycle progression 1		1.46316123	0.00325706	0.06329383	Up in BrM
378	NOB1	Q9ULX3	NIN1 (RPN12) binding protein 1 homolog		1.671570721	0.00201796	0.05435962	Up in BrM
379	SSR3	Q9UNL2	signal sequence receptor subunit 3		1.662025512	0.0079541	0.08273963	Up in BrM
380	WDR3	Q9UNX4	WD repeat domain 3		1.233937601	0.00619816	0.07747209	Up in BrM
381	NIP7	Q9Y221	nucleolar pre-rRNA processing protein NIP7	Radezolid	1.704855395	0.00415164	0.06770899	Up in BrM
382	ERGIC3	Q9Y282	ERGIC and golgi 3		1.307542978	0.00487466	0.07185064	Up in BrM
383	MRPS33	Q9Y291	mitochondrial ribosomal protein S33		1.599412114	0.00661881	0.07906805	Up in BrM
384	RCL1	Q9Y2P8	RNA terminal phosphate cyclase like 1		1.842905812	0.00106242	0.04393504	Up in BrM
385	MRPS28	Q9Y2Q9	mitochondrial ribosomal protein S28		1.298976231	0.00135625	0.04909861	Up in BrM
386	DDX52	Q9Y2R4	DExD-box helicase 52		1.25502064	0.00982466	0.09029444	Up in BrM
387	MRPS17	Q9Y2R5	mitochondrial ribosomal protein S17		1.804549201	0.0002742	0.03217659	Up in BrM
388	POLDIP2	Q9Y2S7	DNA polymerase delta interacting protein 2		1.533762608	0.00269758	0.05914721	Up in BrM
389	MRPS2	Q9Y399	mitochondrial ribosomal protein S2		1.177901865	0.0082489	0.08381865	Up in BrM
390	TMED5	Q9Y3A6	transmembrane p24 trafficking protein 5		1.789408948	0.00244586	0.05861487	Up in BrM
391	NOP16	Q9Y3C1	NOP16 nucleolar protein		1.521564084	0.00661581	0.07906805	Up in BrM
392	MRPS16	Q9Y3D3	mitochondrial ribosomal protein S16		1.206449573	0.00307686	0.06171514	Up in BrM
393	RPL36	Q9Y3U8	ribosomal protein L36		1.421339681	0.00017698	0.02608668	Up in BrM
394	OARD1	Q9Y530	O-acyl-ADP-ribose deacylase 1		1.169367383	0.00457319	0.06977687	Up in BrM
395	UTP18	Q9Y5J1	UTP18 small subunit processome component		1.337375916	0.0008138	0.04060776	Up in BrM
396	PEX16	Q9Y5Y5	peroxisomal biogenesis factor 16		1.553254212	0.00272876	0.05914721	Up in BrM
397	F11R	Q9Y624	F11 receptor		2.668515631	0.00043781	0.03510169	Up in BrM
398	ALG6	Q9Y672	ALG6 alpha-1,3-glucosyltransferase		1.314315206	0.00290456	0.06043263	Up in BrM
399	FKBP7	Q9Y680	FKBP prolyl isomerase 7		1.665488335	0.00590322	0.07737918	Up in BrM
400	SPCS1	Q9Y6A9	signal peptidase complex subunit 1		1.375557586	0.00601835	0.07737918	Up in BrM
401	ARHGEF3	A1IGU5	Rho guanine nucleotide exchange factor 37		-1.006230842	0.00649268	0.07883643	Down in BrM
402	MACROD	A1Z1Q3	mono-ADP ribosylhydrolase 2		-1.060671603	0.00173669	0.05100392	Down in BrM
403	GRID2IP	A4D2P6	Grid2 interacting protein		-1.92572659	0.00046649	0.03510169	Down in BrM
404	MAP1LC3 B2	A6NCE7	microtubule associated protein 1 light chain 3 beta 2		-2.445054487	0.00033612	0.03407695	Down in BrM
405	TUBAL3	A6NHL2	tubulin alpha like 3		-1.149220882	0.00715142	0.0795008	Down in BrM

406	DISP2	A7MBM2	dispatched RND transporter family member 2		-1.717220427	0.00988133	0.09046538	Down in BrM
407	FXYD1	O00168	FXYD domain containing ion transport regulator 1		-2.531937249	0.0009725	0.04297036	Down in BrM
408	BIN1	O00499	bridging integrator 1		-1.860903753	0.00160897	0.05084199	Down in BrM
409	CHL1	O00533	cell adhesion molecule L1 like		-1.803150954	0.00629837	0.07752356	Down in BrM
410	PLPP3	O14495	phospholipid phosphatase 3		-1.546032273	0.00584023	0.07737918	Down in BrM
411	DPYSL4	O14531	dihydropyrimidinase like 4		-2.086716604	0.00368077	0.06474551	Down in BrM
412	DYNC1I1	O14576	dynein cytoplasmic 1 intermediate chain 1		-2.325984354	0.00087494	0.04142282	Down in BrM
413	NHERF1	O14745	NHERF family PDZ scaffold protein 1		-1.11847805	0.00335381	0.06387132	Down in BrM
414	MRAS	O14807	muscle RAS oncogene homolog		-1.51854128	0.00155674	0.05065405	Down in BrM
415	CPLX1	O14810	complexin 1		-1.951460842	0.0004396	0.03510169	Down in BrM
416	NCAM2	O15394	neural cell adhesion molecule 2		-2.111543095	0.00610401	0.07737918	Down in BrM
417	TNRC18	O15417	trinucleotide repeat containing 18		-1.394497454	0.00345111	0.06431193	Down in BrM
418	CAPN5	O15484	calpain 5		-1.273344638	0.00454797	0.06977687	Down in BrM
419	FABP7	O15540	fatty acid binding protein 7		-2.416924386	0.0087933	0.08575938	Down in BrM
420	PLXNB1	O43157	plexin B1		-1.274981979	0.00028342	0.03217659	Down in BrM
421	PHGDH	O43175	phosphoglycerate dehydrogenase	NADH	-1.595765252	0.00333558	0.06387132	Down in BrM
422	SEPTIN4	O43236	septin 4		-3.075968842	0.0000385	0.02036635	Down in BrM
423	DYNC1LI	O43237	dynein cytoplasmic 1 light intermediate chain 2		-1.061706051	0.0000909	0.02036635	Down in BrM
424	HSPA12A	O43301	heat shock protein family A (Hsp70) member 12A		-2.642341837	0.00454998	0.06977687	Down in BrM
425	SYNJ1	O43426	synaptojanin 1		-1.951256077	0.00758324	0.08081895	Down in BrM
426	EPB41L2	O43491	erythrocyte membrane protein band 4.1 like 2		-1.089927406	0.00355968	0.06474209	Down in BrM
427	KRT86	O43790	keratin 86		-2.075850252	0.00696028	0.07944195	Down in BrM
428	LANCL1	O43813	LanC like glutathione S-transferase 1		-1.44251278	0.00922647	0.087774	Down in BrM
429	GNG7	O60262	G protein subunit gamma 7		-2.577903993	0.00244604	0.05861487	Down in BrM
430	KIF5C	O60282	kinesin family member 5C		-1.025478094	0.0071271	0.0795008	Down in BrM
431	LZTS3	O60299	leucine zipper tumor suppressor family member 3		-1.161712728	0.00125569	0.04716078	Down in BrM
432	SNAP91	O60641	synaptosome associated protein 91		-2.074236071	0.00456351	0.06977687	Down in BrM
433	GAS7	O60861	growth arrest specific 7		-2.203053862	0.00039611	0.03478999	Down in BrM
434	DNAJC6	O75061	DnaJ heat shock protein family (Hsp40) member C6		-1.952463245	0.00740395	0.07988904	Down in BrM
435	ADAM11	O75078	ADAM metallopeptidase domain 11		-1.483922549	0.00755491	0.08067698	Down in BrM
436	MEGF6	O75095	multiple EGF like domains 6		-3.038956231	0.00112264	0.04558136	Down in BrM
437	CLASP2	O75122	cytoplasmic linker associated protein 2		-1.460243814	0.00062248	0.03845173	Down in BrM
438	CAND2	O75155	cullin associated and neddylation dissociated 2 (putative)		-1.06243064	0.0000788	0.02036635	Down in BrM
439	SEMA7A	O75326	semaphorin 7A (JohnMiltonHagen blood group)		-1.519970609	0.00741442	0.07989165	Down in BrM
440	TRIM3	O75382	tripartite motif containing 3		-1.687229191	0.00224066	0.05701648	Down in BrM
441	HSBP1	O75506	heat shock factor binding protein 1		-1.301737685	0.00198961	0.05415626	Down in BrM
442	PALM	O75781	paralemmin		-1.989247259	0.00909948	0.08695736	Down in BrM
443	TNFSF13	O75888	TNF superfamily member 13		-1.152373443	0.00031689	0.03300736	Down in BrM
444	ALDH1L1	O75891	aldehyde dehydrogenase 1 family member L1	Tetrahydrofolic acid	-2.128122816	0.00360419	0.06474551	Down in BrM
445	NEBL	O76041	nebulette		-1.757210786	0.00398885	0.0665948	Down in BrM
446	SEC14L2	O76054	SEC14 like lipid binding 2	Vitamin E; alpha-Tocopherol succinate; D-alpha-Tocopherol acetate	-1.963233724	0.00708248	0.07948878	Down in BrM
447	SNCG	O76070	synuclein gamma		-3.901208929	0.0009253	0.04252032	Down in BrM
448	DDAH1	O94760	dimethylarginine dimethylaminohydrolase 1	Citrulline; Pantoprazole; Esomeprazole; Dexlansoprazole	-1.244211129	0.00481067	0.07178313	Down in BrM
449	TPPP	O94811	tubulin polymerization promoting protein		-2.905450178	0.00887399	0.08605411	Down in BrM
450	KBTBD11	O94819	kelch repeat and BTB domain containing 11		-2.057579296	0.0077217	0.08165707	Down in BrM
451	NFASC	O94856	neurofascin		-2.859060404	0.00617639	0.07737918	Down in BrM
452	EPN2	O95208	epsin 2		-1.392904891	0.00697552	0.07944195	Down in BrM

453	KIF4A	O95239	kinesin family member 4A		-1.647990005	0.00095421	0.04297036	Down in BrM
454	SBF1	O95248	SET binding factor 1		-1.43869589	0.009538	0.08874968	Down in BrM
455	MED26	O95402	mediator complex subunit 26		-1.919570918	0.00624541	0.07752356	Down in BrM
456	ATP6V1G	O95670	ATPase H+ transporting V1 subunit G2		-1.985918229	0.00783723	0.08223722	Down in BrM
457	HSPA4L	O95757	heat shock protein family A (Hsp70) member 4 like		-1.929281787	0.00214533	0.05549443	Down in BrM
458	BAG3	O95817	BAG cochaperone 3		-1.22392417	0.00714421	0.0795008	Down in BrM
459	HRAS	P01112	HRas proto-oncogene, GTPase	Hexane-1,6-Diol; Trifluoroethanol; Guanosine-5'-Triphosphate; Guanosine-5'-Diphosphate; N,N'-DIMETHYL-N-(ACETYL)-N'-(7-NITROBENZ-2-OXA-1,3-DIAZOL-4-	-1.444597519	0.00177356	0.05131507	Down in BrM
460	NPY	P01303	neuropeptide Y	Phentermine; 2-aminoisobutyric acid; L-tyrosinamide	-1.259187824	0.00164409	0.05084199	Down in BrM
461	IGKV1-39	P01597	None	None	-1.2709233	0.00641322	0.07834571	Down in BrM
462	PMP2	P02689	peripheral myelin protein 2	S-Hydroxycysteine; Palmitic Acid; Dodecyldimethylamine N-oxide; Oleic Acid	-2.71066778	0.00891694	0.08620009	Down in BrM
463	PRNP	P04156	prion protein (Kanno blood group)	Tetracycline; Copper	-1.594958014	0.0055595	0.07575872	Down in BrM
464	S100B	P04271	S100 calcium binding protein B	Olopatadine; Calcium; N-Formylmethionine; Arundic acid; (Z)-2-[2-(4-methylpiperazin-1-yl)benzyl]diazenecarbothioamide; 2-[(5-hex-1-yn-1-ylfuran-2-yl)carbonyl]-N-methylhydrazinecarbothioamide; Calcium citrate; Calcium Phosphate; Calcium phosphate dihydrate	-2.831318746	0.00735143	0.07967639	Down in BrM
465	ATP1B1	P05026	ATPase Na+/K+ transporting subunit beta 1		-1.489365251	0.00539222	0.07550546	Down in BrM
466	FYN	P06241	FYN proto-oncogene, Src family tyrosine kinase	Dasatinib; Triglyme; Fostamatinib	-1.294290572	0.00504284	0.07290812	Down in BrM
467	BCHE	P06276	butyrylcholinesterase	Choline; Tacrine; Dipivefrin; Pyridostigmine; Galantamine; Isoflurophate; Pralidoxime; Malathion; Donepezil; Hexafluronium; Demecarium; Rivastigmine; Edrophonium; Echothiophate; Ketamine; Mivacurium; Diethyl phosphonate; Methylphosphinic Acid; Butyric Acid; 9-N-Phenylmethylamino-Tacrine; 2-(N-morpholino)ethanesulfonic acid; Ethyl dihydrogen phosphate; Butyrylthiocholine; Phenserine; DODECANESULFONATE ION; 9-(3-IODOBENZYLAMINO)-1,2,3,4-TETRAHYDROACRIDINE; (1R)-menthyl hexyl phosphonate group; (1S)-	-1.122019652	0.00972219	0.08970571	Down in BrM
468	NEFL	P07196	neurofilament light chain		-3.281099401	0.00243507	0.05861487	Down in BrM
469	NEFM	P07197	neurofilament medium chain		-3.229623727	0.00298312	0.06116575	Down in BrM

470	PFKM	P08237	phosphofructokinase, muscle		-1.947630643	0.00794011	0.08270416	Down in BrM
471	ENO2	P09104	enolase 2	2-Phosphoglycolic Acid	-2.274753395	0.00499595	0.07268088	Down in BrM
472	QDPR	P09417	quinoid dihydropteridine reductase	NADH; 2-(N-morpholino)ethanesulfonic acid	-2.086470396	0.00561469	0.07588573	Down in BrM
473	GNAO1	P09471	G protein subunit alpha o1		-2.882355564	0.00803171	0.08316259	Down in BrM
474	CLTB	P09497	clathrin light chain B		-1.323207269	0.00940035	0.08869024	Down in BrM
475	CNP	P09543	2',3'-cyclic nucleotide 3' phosphodiesterase		-2.960400592	0.00367028	0.06474551	Down in BrM
476	ALDOC	P09972	aldolase, fructose-bisphosphate C		-2.395411178	0.00160452	0.05084199	Down in BrM
477	JMJD7	P0C870	jumonji domain containing 7		-1.258161687	0.00630149	0.07752356	Down in BrM
478	MAPT	P10636	microtubule associated protein tau	Lansoprazole; Astemizole; Paclitaxel; Docetaxel; Flortaucipir F-18	-2.571788482	0.00083184	0.04086994	Down in BrM
479	PYGB	P11216	glycogen phosphorylase B	Pyridoxal phosphate; Alvocidib	-1.707616315	0.00954298	0.08874968	Down in BrM
480	NEFH	P12036	neurofilament heavy chain		-3.263968426	0.00065424	0.03845173	Down in BrM
481	CKB	P12277	creatine kinase B	Creatine; Phosphocreatine	-2.833611627	0.00446042	0.06977231	Down in BrM
482	CKMT1A	P12532	creatine kinase, mitochondrial 1B		-2.273839668	0.00610062	0.07737918	Down in BrM
483	ATP1A3	P13637	ATPase Na+/K+ transporting subunit alpha 3	Ouabain	-2.088750989	0.00188909	0.05289456	Down in BrM
484	ATP1B2	P14415	ATPase Na+/K+ transporting subunit beta 2		-2.480405746	0.00516374	0.07347019	Down in BrM
485	ACYP2	P14621	acylphosphatase 2	Formic acid	-2.319098866	0.00037154	0.03456622	Down in BrM
486	GABRA1	P14867	gamma-aminobutyric acid type A receptor subunit alpha1	Lorazepam; Ethchlorvynol; Enflurane; Temazepam; Butabarbital; Butalbital; Phenytoin; Topiramate; Etomidate; Talbutal; Pentobarbital; Olanzapine; Clobazam; Meprobamate; Carisoprodol; Eszopiclone; Alprazolam; Secobarbital; Zolpidem; Metharbital; Picrotoxin; Methohexital; Chlordiazepoxide; Amoxapine; Adinazolam; Lamotrigine; Thiopental; Medroxyprogesterone acetate; Clorazepic acid; Acamprosate; Midazolam; Flurazepam; Isoflurane; Primidone; Halazepam; Propofol; Diazepam; Progabide; Oxazepam; Methylphenobarbital; Triazolam; Ethanol; Zaleplon; Methoxyflurane; Memantine; Clonazepam; Methyprylon; Thiamylal; Halothane; Phenobarbital; Desflurane; Zopiclone; Flumazenil; Estazolam; Sevoflurane; Dihydroquinidine barbiturate; Quinidine barbiturate; Amobarbital; Aprobarbital; Butobarbital; Heptabarbital; Hexobarbital; Ginkgo biloba; Glutethimide; Barbital; Camazepam; Dihydro-2-thioxo-5-((5-(2-(trifluoromethyl)phenyl)-2 [illegible]	-1.676915214	0.00085797	0.04142282	Down in BrM
487	GLUL	P15104	glutamate-ammonia ligase	L-Glutamine; Glutamic acid	-2.150618374	0.00697715	0.07944195	Down in BrM
488	ACAN	P16112	aggrecan	Ilomastat	-1.958037155	0.00071457	0.03845173	Down in BrM

489	STMN1	P16949	stathmin 1		-1.908991752	0.00066969	0.03845173	Down in BrM
490	ZNF28	P17035	zinc finger protein 28		-1.633386038	0.00961041	0.08916454	Down in BrM
491	GOT1	P17174	glutamic-oxaloacetic transaminase 1	Pyridoxal phosphate; Aspartic acid; Glutamic acid; Cysteine; Adapalene; Maleic Acid; Copper	-2.151306734	0.00099345	0.04311574	Down in BrM
492	GJA1	P17302	gap junction protein alpha 1	Carvedilol	-2.471607194	0.00064078	0.03845173	Down in BrM
493	CKMT2	P17540	creatine kinase, mitochondrial 2	Creatine; Phosphocreatine	-2.511010344	0.0048557	0.07183273	Down in BrM
494	GAP43	P17677	growth associated protein 43		-2.529770148	0.00732171	0.07967639	Down in BrM
495	CDH2	P19022	cadherin 2		-2.24298554	0.00303725	0.0614689	Down in BrM
496	GNAZ	P19086	G protein subunit alpha z		-2.488315764	0.00391271	0.06644808	Down in BrM
497	NEB	P20929	nebulin		-1.645090391	0.0010485	0.04393504	Down in BrM
498	ATP6V1B2	P21281	ATPase H+ transporting V1 subunit B2	Gallium nitrate; 4-(2-Aminoethyl)Benzenesulfonyl Fluoride	-1.47733168	0.00765738	0.08138704	Down in BrM
499	ATP6V1C	P21283	ATPase H+ transporting V1 subunit C1	D-tartaric acid; Thonzonium	-1.182911849	0.00755959	0.08067698	Down in BrM
500	CSRP1	P21291	cysteine and glycine rich protein 1	Artenimol	-1.224727345	0.00728777	0.07967639	Down in BrM
501	S1PR1	P21453	sphingosine-1-phosphate receptor 1	Fingolimod; Asfotase alfa; Siponimod; Ozanimod	-1.340612689	0.00813523	0.08338907	Down in BrM
502	PCMT1	P22061	protein-L-isoaspartate (D-aspartate) O-methyltransferase	S-adenosyl-L-homocysteine	-1.180006697	0.00236173	0.0581633	Down in BrM
503	S100A1	P23297	S100 calcium binding protein A1	Olopatadine	-3.043084128	0.00832078	0.0838895	Down in BrM
504	PTPRD	P23468	protein tyrosine phosphatase receptor type D		-1.192026059	0.00286842	0.06038225	Down in BrM
505	PTPRZ1	P23471	protein tyrosine phosphatase receptor type Z1		-2.127986522	0.00088038	0.04142282	Down in BrM
506	OMG	P23515	oligodendrocyte myelin glycoprotein		-2.683970823	0.00350717	0.06446586	Down in BrM
507	MT3	P25713	metallothionein 3	Zinc; Zinc acetate; Zinc chloride; Zinc sulfate, unspecified form	-2.458580178	0.00733863	0.07967639	Down in BrM
508	ITGB8	P26012	integrin subunit beta 8		-1.346913582	0.00575493	0.07720644	Down in BrM
509	CTNNA2	P26232	catenin alpha 2		-1.174691744	0.00409933	0.06727728	Down in BrM
510	ELAVL4	P26378	ELAV like RNA binding protein 4		-1.96089725	0.00128765	0.04752087	Down in BrM
511	CERS1	P27544	ceramide synthase 1		-1.707974222	0.00196481	0.05404597	Down in BrM
512	ITPKB	P27987	inositol-trisphosphate 3-kinase B		-1.263838812	0.00809505	0.08316689	Down in BrM

513	MAPK1	P28482	mitogen-activated protein kinase 1	Acetylsalicylic acid; Minocycline; Isoprenaline; Arsenic trioxide; Olomoucine; Phosphonothreonine; Purvalanol; SB220025; Seliciclib; Perifosine; N,N-DIMETHYL-4-(4-PHENYL-1H-PYRAZOL-3-YL)-1H-PYRROLE-2-CARBOXAMIDE; N-BENZYL-4-[4-(3-CHLOROPHENYL)-1H-PYRAZOL-3-YL]-1H-PYRROLE-2-CARBOXAMIDE; (S)-N-(1-(3-CHLORO-4-FLUOROPHENYL)-2-HYDROXYETHYL)-4-(4-(3-CHLOROPHENYL)-1H-PYRAZOL-3-YL)-1H-PYRROLE-2-CARBOXAMIDE; (3R,5Z,8S,9S,11E)-8,9,16-TRIHYDROXY-14-METHOXY-3-METHYL-3,4,9,10-TETRAHYDRO-1H-2-BENZOXACYCLOTETRADECINE-1,7(8H)-DIONE; 5-(2-PHENYLPYRAZOLO[1,5-A]PYRIDIN-3-YL)-1H-PYRAZOLO[3,4-C]PYRIDAZIN-3-AMINE; (1aR,8S,13S,14S,15aR)-5,13,14-trihydroxy-3-methoxy-8-methyl-8,9,13,14,15,15a-hexahydro-6H-[illegible]	-1.209105717	0.00455247	0.06977687	Down in BrM
514	IMPA1	P29218	inositol monophosphatase 1	Lithium cation; L-Myo-Inositol-1-Phosphate; Lithium citrate; Lithium succinate; Lithium carbonate	-1.647706287	0.00440187	0.06945479	Down in BrM
515	MARCKS	P29966	myristoylated alanine rich protein kinase C substrate		-1.4835022	0.00337574	0.06387132	Down in BrM
516	PEBP1	P30086	phosphatidylethanolamine binding protein 1	Copper	-1.475095482	0.00082985	0.04086994	Down in BrM
517	GDI1	P31150	GDP dissociation inhibitor 1		-2.011057594	0.00547297	0.07563206	Down in BrM
518	PFN2	P35080	profilin 2	Triglyme; Pentaglyme	-1.521104602	0.003343	0.06387132	Down in BrM
519	ADD1	P35611	adducin 1		-1.506451748	0.0023267	0.05788581	Down in BrM
520	ATP6V1E	P36543	ATPase H+ transporting V1 subunit E1		-1.313358468	0.00557003	0.07575872	Down in BrM
521	ATP6V1A	P38606	ATPase H+ transporting V1 subunit A	Alendronic acid; Etidronic acid; Bafilomycin A1; Bafilomycin B1	-1.534174393	0.00950201	0.08874968	Down in BrM
522	CAP2	P40123	cyclase associated actin cytoskeleton regulatory protein 2		-1.571779517	0.0034101	0.06403775	Down in BrM
523	MDH1	P40925	malate dehydrogenase 1	NADH; Nicotinamide adenine dinucleotide phosphate; Artenimol	-1.652265756	0.00164917	0.05084199	Down in BrM
524	TSPAN7	P41732	tetraspanin 7		-1.658346829	0.0040988	0.06727728	Down in BrM
525	SLC1A3	P43003	solute carrier family 1 member 3	Glutamic acid	-2.920805003	0.00456917	0.06977687	Down in BrM
526	GSTM5	P46439	glutathione S-transferase mu 5	Glutathione	-1.404607264	0.00830098	0.0838895	Down in BrM
527	MAP1B	P46821	microtubule associated protein 1B		-2.191175415	0.00063936	0.03845173	Down in BrM
528	GABRB2	P47870	gamma-aminobutyric acid type A receptor subunit beta2	Carisoprodol; Propofol; Ethanol; Ginkgo biloba; Fludiazepam; Fospropofol	-2.650393757	0.0000177	0.01742196	Down in BrM

529	PPP3CC	P48454	protein phosphatase 3 catalytic subunit gamma		-1.351562478	0.00809892	0.08316689	Down in BrM
530	SLC1A6	P48664	solute carrier family 1 member 6	Glutamic acid	-2.379763835	0.00942795	0.08874968	Down in BrM
531	AMPH	P49418	amphiphysin		-3.005279388	0.00824187	0.08381865	Down in BrM
532	INPP1	P49441	inositol polyphosphate-1-phosphatase		-1.11711517	0.00836408	0.0838895	Down in BrM
533	GNAQ	P50148	G protein subunit alpha q		-1.960370962	0.00209819	0.05549443	Down in BrM
534	ATP1A2	P50993	ATPase Na+/K+ transporting subunit alpha 2	Ouabain	-2.606591823	0.00891124	0.08620009	Down in BrM
535	PLCD1	P51178	phospholipase C delta 1	1D-myo-inositol 1,4,5-trisphosphate; Inositol 2,4,5-trisphosphate	-2.218958897	0.00118433	0.04625996	Down in BrM
536	NOVA1	P51513	NOVA alternative splicing regulator 1		-2.259046633	0.00671827	0.07938502	Down in BrM
537	RAP1GDS	P52306	Rap1 GTPase-GDP dissociation stimulator 1		-1.902545161	0.00292411	0.06059184	Down in BrM
538	RIDA	P52758	reactive intermediate imine deaminase A homolog		-1.143590611	0.00630152	0.07752356	Down in BrM
539	RCAN1	P53805	regulator of calcineurin 1	Dipyridamole	-1.063063754	0.0072802	0.07967639	Down in BrM
540	AQP4	P55087	aquaporin 4		-2.185971989	0.00106295	0.04393504	Down in BrM
541	CDH4	P55283	cadherin 4		-1.583825809	0.00028869	0.03217659	Down in BrM
542	ARPP19	P56211	cAMP regulated phosphoprotein 19		-1.385862736	0.00355492	0.06474209	Down in BrM
543	JAM2	P57087	junctional adhesion molecule 2		-1.410724584	0.00049264	0.03562344	Down in BrM
544	GABARAPL2	P60520	GABA type A receptor associated protein like 2		-1.888461012	0.00053862	0.03619764	Down in BrM
545	RAC3	P60763	Rac family small GTPase 3		-1.84932884	0.00425751	0.06843549	Down in BrM
546	USP46	P62068	ubiquitin specific peptidase 46		-1.563186909	0.0056392	0.07608534	Down in BrM
547	YWHAE	P62258	tyrosine 3-monooxygenase/tryptophan 5-monooxygenase activation protein epsilon	Fusicoccin; Phenethyl Isothiocyanate	-1.192629737	0.00539264	0.07550546	Down in BrM
548	GNAI1	P63096	G protein subunit alpha i1	Guanosine-5'-Diphosphate; Tetrafluoroaluminate Ion	-1.979373997	0.00347476	0.0643459	Down in BrM
549	SKP1	P63208	S-phase kinase associated protein 1	1-naphthaleneacetic acid; (2S)-2-(1H-indol-3-yl)hexanoic acid; (2S)-2-(1H-indol-3-yl)pentanoic acid; (2S)-8-[(tert-butoxycarbonyl)amino]-2-(1H-indol-3-yl)octanoic acid; Indoleacetic acid	-1.04629822	0.00275118	0.05920718	Down in BrM
550	FKBP1B	P68106	FKBP prolyl isomerase 1B		-1.513342955	0.00986786	0.09046538	Down in BrM
551	TUBA1B	P68363	tubulin alpha 1b	Epothilone D; Patupilone; CYT997; 2-MERCAPTO-N-[1,2,3,10-TETRAMETHOXY-9-OXO-5,6,7,9-TETRAHYDRO-BENZO[A]HEPTALEN-7-	-1.374437544	0.00892676	0.08620009	Down in BrM
552	TUBB4B	P68371	tubulin beta 4B class IVb	Albendazole; Mebendazole; Epothilone D; Patupilone; Oxibendazole; CYT997; Phenethyl Isothiocyanate	-1.092681176	0.00073247	0.03845173	Down in BrM
553	PIP4K2B	P78356	phosphatidylinositol-5-phosphate 4-kinase type 2 beta	Fostamatinib	-1.471141324	0.0074412	0.08003436	Down in BrM
554	CNTNAP1	P78357	contactin associated protein 1		-2.364408795	0.00702046	0.07945048	Down in BrM
555	MAP1A	P78559	microtubule associated protein 1A	Estramustine	-2.561582982	0.00076371	0.03899415	Down in BrM
556	ABAT	P80404	4-aminobutyrate aminotransferase	Pyridoxal phosphate; Pyruvic acid; Glutamic acid; Alanine; Phenelzine; Vigabatrin; (4e)-4-Aminohex-4-Enoic Acid; 4-Amino Hexanoic Acid	-1.987710844	0.00837956	0.0838895	Down in BrM

557	PITPNA	Q00169	phosphatidylinositol transfer protein alpha	1,2-diacyl-sn-glycero-3-phosphoinositol; (Z,Z)-4-Hydroxy-N,N,N-Trimethyl-10-Oxo-7-[(1-Oxo-9-Octadecenyl)Oxy]-3,5,9-Trioxa-4-Phosphaheptacos-18-En-1-Aminium-4-Oxide	-1.076064244	0.00731047	0.07967639	Down in BrM
558	PURA	Q00577	purine rich element binding protein A		-1.6128636	0.00314031	0.06258099	Down in BrM
559	SPTBN1	Q01082	spectrin beta, non-erythrocytic 1	Calcium; 1D-myo-inositol 1,4,5-trisphosphate	-1.668470263	0.00692044	0.07944195	Down in BrM
560	ANK2	Q01484	ankyrin 2		-2.203653794	0.00260867	0.05914721	Down in BrM
561	ATP2B2	Q01814	ATPase plasma membrane Ca2+ transporting 2		-2.573931402	0.00700214	0.07944195	Down in BrM
562	CNTN2	Q02246	contactin 2		-2.389194113	0.0006884	0.03845173	Down in BrM
563	DST	Q03001	dystonin		-1.526554569	0.00381697	0.06614708	Down in BrM
564	DNM1	Q05193	dynamin 1		-2.368795457	0.00807726	0.08316689	Down in BrM
565	EEF1A2	Q05639	eukaryotic translation elongation factor 1 alpha 2		-1.266950799	0.00666919	0.07906871	Down in BrM
566	KCNA1	Q09470	potassium voltage-gated channel subfamily A member 1	Amitriptyline; Isoflurane; Methoxyflurane; Desflurane; Dalfampridine; Tetraethylammonium;	-2.151560079	0.0004268	0.03510169	Down in BrM
567	SCRN1	Q12765	secernin 1		-1.659806261	0.00064685	0.03845173	Down in BrM
568	CNTN1	Q12860	contactin 1		-2.668264073	0.00517026	0.07347019	Down in BrM
569	TRAF3	Q13114	TNF receptor associated factor 3		-1.264046352	0.0000991	0.02036635	Down in BrM
570	GRM1	Q13255	glutamate metabotropic receptor 1	Glutamic acid; (S)-alpha-methyl-4-carboxyphenylglycine	-1.822583379	0.00753452	0.08067698	Down in BrM
571	LSAMP	Q13449	limbic system associated membrane protein		-2.382791947	0.00821642	0.08379459	Down in BrM
572	TUBB3	Q13509	tubulin beta 3 class III	Epothilone D; Patupilone; Ixabepilone; CYT997; ZEN-012; Phenethyl Isothiocyanate	-2.275377959	0.00722983	0.07967639	Down in BrM
573	PIN1	Q13526	peptidylprolyl cis/trans isomerase, NIMA-interacting 1	Beta-(2-Naphthyl)-Alanine; 3,6,9,12,15,18-HEXAOXAICOSANE	-1.366595663	0.00784729	0.08223722	Down in BrM
574	CAMK2B	Q13554	calcium/calmodulin dependent protein kinase II beta	[4-({4-[(5-cyclopropyl-1H-pyrazol-3-yl)amino]-6-(methylamino)pyrimidin-2-yl}amino)phenyl]acetonitrile;	-2.127333924	0.00722722	0.07967639	Down in BrM
575	MTMR2	Q13614	myotubularin related protein 2		-1.34387255	0.00604981	0.07737918	Down in BrM
576	FHL1	Q13642	four and a half LIM domains 1		-1.795049233	0.00810085	0.08316689	Down in BrM
577	SPTAN1	Q13813	spectrin alpha, non-erythrocytic 1		-1.519187257	0.00105507	0.04393504	Down in BrM
578	AUH	Q13825	AU RNA binding methylglutaconyl-CoA hydratase		-1.062888694	0.00444302	0.0696965	Down in BrM
579	TUBB2A	Q13885	tubulin beta 2A class IIa	CYT997; Phenethyl Isothiocyanate	-1.605733016	0.00393304	0.06646771	Down in BrM
580	MPP2	Q14168	MAGUK p55 scaffold protein 2		-1.726965811	0.00124763	0.04708429	Down in BrM
581	CRMP1	Q14194	collapsin response mediator protein 1		-2.087004175	0.00603942	0.07737918	Down in BrM
582	ARID5B	Q14865	AT-rich interaction domain 5B		-1.281244938	0.00715769	0.0795008	Down in BrM
583	CRYM	Q14894	crystallin mu	NRP409	-2.194512312	0.00256035	0.05914721	Down in BrM
584	HEPACA	Q14CZ8	hepatic and glial cell adhesion molecule		-2.510849605	0.00242221	0.05858302	Down in BrM
585	PEA15	Q15121	proliferation and apoptosis adaptor protein 15		-1.47734064	0.00578292	0.07727637	Down in BrM
586	LLGL1	Q15334	LLGL scribble cell polarity complex component 1		-1.974845844	0.00253792	0.05914721	Down in BrM
587	MAPRE2	Q15555	microtubule associated protein RP/EB family member 2		-1.623452975	0.00267485	0.05914721	Down in BrM
588	DLG2	Q15700	discs large MAGUK scaffold protein 2		-2.187506874	0.00197227	0.05406102	Down in BrM
589	ITSN1	Q15811	intersectin 1		-1.145849149	0.00656638	0.07906805	Down in BrM
590	ADIRF	Q15847	adipogenesis regulatory factor		-1.098703172	0.00320796	0.06316325	Down in BrM
591	SNCB	Q16143	synuclein beta		-2.629465369	0.00276877	0.05942199	Down in BrM
592	SEPTIN7	Q16181	septin 7		-1.676751522	0.00180997	0.05179296	Down in BrM

593	INA	Q16352	internexin neuronal intermediate filament protein alpha		-3.244394172	0.00202764	0.05443277	Down in BrM
594	DPYSL2	Q16555	dihydropyrimidinase like 2	Artenimol	-2.166724966	0.0025693	0.05914721	Down in BrM
595	MOG	Q16653	myelin oligodendrocyte glycoprotein		-3.647536725	0.00736385	0.07967639	Down in BrM
596	KYAT1	Q16773	kynurenine aminotransferase 1	Pyridoxal phosphate; Pyridoxamine-5'-Phosphate; D-Phenylalanine; N(6)-(pyridoxal phosphate)-L-lysine; Indoleacetic acid	-1.421417354	0.00500083	0.07268088	Down in BrM
597	RTN1	Q16799	reticulon 1		-2.29905663	0.00253782	0.05914721	Down in BrM
598	AAK1	Q2M2I8	AP2 associated kinase 1	Fostamatinib	-1.336590541	0.00264108	0.05914721	Down in BrM
599	PREPL	Q4J6C6	prolyl endopeptidase like		-1.460010746	0.00052548	0.03614653	Down in BrM
600	RFTN2	Q52LD8	raftlin family member 2		-2.134050022	0.00043235	0.03510169	Down in BrM
601	ATAT1	Q5SQI0	alpha tubulin acetyltransferase 1		-1.350588613	0.00431466	0.06892864	Down in BrM
602	TTBK1	Q5TCY1	tau tubulin kinase 1		-1.803580326	0.00629474	0.07752356	Down in BrM
603	FAM171A	Q5VUB5	family with sequence similarity 171 member A1		-1.170453092	0.00542224	0.07550546	Down in BrM
604	TAF3	Q5VWG9	TATA-box binding protein associated factor 3		-2.070364159	0.00121009	0.04656769	Down in BrM
605	KAZN	Q674X7	kazrin, periplakin interacting protein		-2.454247684	0.0004763	0.03543685	Down in BrM
606	MBLAC2	Q68D91	metallo-beta-lactamase domain containing 2		-1.523116846	0.0012398	0.04708429	Down in BrM
607	IQSEC1	Q6DN90	IQ motif and Sec7 domain ArfGEF 1		-1.213832674	0.00470276	0.07064999	Down in BrM
608	KCNIP4	Q6PIL6	potassium voltage-gated channel interacting protein 4		-2.198491665	0.0000528	0.02036635	Down in BrM
609	TOM1L2	Q6ZVM7	target of myb1 like 2 membrane trafficking protein		-1.299075694	0.0062822	0.07752356	Down in BrM
610	ILDR2	Q71H61	immunoglobulin like domain containing receptor 2		-1.053304043	0.00263654	0.05914721	Down in BrM
611	MTSS2	Q765P7	MTSS I-BAR domain containing 2		-1.603896081	0.00332624	0.06387132	Down in BrM
612	RUFY3	Q7L099	RUN and FYVE domain containing 3		-2.245128056	0.00151705	0.05043056	Down in BrM
613	ASRGL1	Q7L266	asparaginase and isoaspartyl peptidase 1	Aspartic acid; Asparagine	-1.674033286	0.00093803	0.04260411	Down in BrM
614	KIF21A	Q7Z4S6	kinesin family member 21A		-1.74030247	0.00930509	0.08800402	Down in BrM
615	PXK	Q7Z7A4	PX domain containing serine/threonine kinase like		-1.163989981	0.00213071	0.05549443	Down in BrM
616	RTN4RL2	Q86UN3	reticulon 4 receptor like 2		-1.393613389	0.00582421	0.07737918	Down in BrM
617	PACS2	Q86VP3	phosphofurin acidic cluster sorting protein 2		-1.204696082	0.00869651	0.08556315	Down in BrM
618	CEP97	Q8IW35	centrosomal protein 97		-1.092453127	0.00129237	0.04752087	Down in BrM
619	NAXD	Q8IW45	NAD(P)HX dehydratase		-1.007088957	0.0021447	0.05549443	Down in BrM
620	SCN4B	Q8IWT1	sodium voltage-gated channel beta subunit 4	Zonisamide	-1.846427825	0.00607637	0.07737918	Down in BrM
621	SIRT2	Q8IXJ6	sirtuin 2	Cambinol	-2.814477433	0.00613984	0.07737918	Down in BrM
622	SPART	Q8N0X7	spartin		-1.177303505	0.00068194	0.03845173	Down in BrM
623	CADM3	Q8N126	cell adhesion molecule 3		-2.246845745	0.0022119	0.05674587	Down in BrM
624	LGI4	Q8N135	leucine rich repeat LGI family member 4		-1.585047162	0.00660797	0.07906805	Down in BrM
625	PLCD3	Q8N3E9	phospholipase C delta 3		-1.683057693	0.00117493	0.04625996	Down in BrM
626	DCLK2	Q8N568	doublecortin like kinase 2	Fostamatinib	-1.81511542	0.00609279	0.07737918	Down in BrM
627	OXR1	Q8N573	oxidation resistance 1		-1.652028281	0.00845433	0.08424136	Down in BrM
628	IGSF1	Q8N6C5	immunoglobulin superfamily member 1		-1.705695039	0.00079071	0.03985156	Down in BrM
629	AMER2	Q8N7J2	APC membrane recruitment protein 2		-1.648647254	0.00356636	0.06474209	Down in BrM
630	GBP7	Q8N8V2	guanylate binding protein 7		-1.457985995	0.00029244	0.03217659	Down in BrM
631	CCNY	Q8ND76	cyclin Y		-1.357624767	0.00868135	0.08552165	Down in BrM
632	SETD9	Q8NE22	SET domain containing 9		-1.676202937	0.00366572	0.06474551	Down in BrM
633	CADM4	Q8NFZ8	cell adhesion molecule 4		-2.716516654	0.00027376	0.03217659	Down in BrM
634	SVIP	Q8NHG7	small VCP interacting protein		-1.886928121	0.00228856	0.05730195	Down in BrM
635	NCOA7	Q8NI08	nuclear receptor coactivator 7		-1.147446358	0.00508484	0.07315431	Down in BrM
636	DTD1	Q8TEA8	D-aminoacyl-tRNA deacylase 1		-1.173446024	0.00073832	0.03845173	Down in BrM
637	PRUNE2	Q8WUY3	prune homolog 2 with BCH domain		-1.516944524	0.00263637	0.05914721	Down in BrM
638	DNAJA4	Q8WW22	DnaJ heat shock protein family (Hsp40) member A4		-1.332869663	0.00357192	0.06474209	Down in BrM

639	PHYHIP	Q92561	phytanoyl-CoA 2-hydroxylase interacting protein		-1.585525076	0.00785317	0.08223722	Down in BrM
640	SEPTIN8	Q92599	septin 8		-2.089603179	0.00014883	0.02422219	Down in BrM
641	NRCAM	Q92823	neuronal cell adhesion molecule		-2.123820255	0.00706904	0.07948878	Down in BrM
642	IGSF8	Q969P0	immunoglobulin superfamily member 8		-2.322833226	0.00273884	0.05914721	Down in BrM
643	WBP2	Q969T9	WW domain binding protein 2		-1.115191037	0.00324549	0.06329383	Down in BrM
644	CMTM5	Q96DZ9	CKLF like MARVEL transmembrane domain containing 5		-1.810297341	0.00211721	0.05549443	Down in BrM
645	SGTB	Q96EQ0	small glutamine rich tetratricopeptide repeat co-chaperone beta		-1.445243193	0.00517263	0.07347019	Down in BrM
646	CNRIP1	Q96F85	cannabinoid receptor interacting protein 1		-2.421848328	0.00194143	0.05359175	Down in BrM
647	PDXP	Q96GD0	pyridoxal phosphatase	Pyridoxal phosphate	-1.738122217	0.00165666	0.05084199	Down in BrM
648	MAP6	Q96JE9	microtubule associated protein 6		-2.291178402	0.00656611	0.07906805	Down in BrM
649	SORCS2	Q96PQ0	sortilin related VPS10 domain containing receptor 2		-1.181120511	0.00585335	0.07737918	Down in BrM
650	LYSMD1	Q96S90	LysM domain containing 1		-1.570601308	0.00272127	0.05914721	Down in BrM
651	TBCB	Q99426	tubulin folding cofactor B		-1.342165568	0.0000576	0.02036635	Down in BrM
652	P2RX7	Q99572	purinergic receptor P2X 7		-2.162203737	0.00088496	0.04142282	Down in BrM
653	FEZ1	Q99689	fasciculation and elongation protein zeta 1		-2.331074691	0.00547206	0.07563206	Down in BrM
654	NAPG	Q99747	NSF attachment protein gamma		-1.083614344	0.00098765	0.04310353	Down in BrM
655	SH3GL2	Q99962	SH3 domain containing GRB2 like 2, endophilin A1		-2.131132411	0.00682461	0.07944195	Down in BrM
656	DPYSL5	Q9BPU6	dihydropyrimidinase like 5		-3.339409886	0.00396488	0.0665948	Down in BrM
657	REEP2	Q9BRK0	receptor accessory protein 2		-1.88379794	0.00270225	0.05914721	Down in BrM
658	TTYH2	Q9BSA4	tweety family member 2		-1.422234993	0.00500542	0.07268088	Down in BrM
659	TUBB6	Q9BUF5	tubulin beta 6 class V	CYT997; Artenimol	-1.485757867	0.0039224	0.06646771	Down in BrM
660	TUBB2B	Q9BVA1	tubulin beta 2B class IIb	CYT997; 2-MERCAPTO-N-[1,2,3,10-TETRAMETHOXY-9-OXO-5,6,7,9-TETRAHYDRO-BENZO[A]HEPTALEN-7-	-1.870955853	0.00143184	0.04998058	Down in BrM
661	UBXN6	Q9BZV1	UBX domain protein 6		-1.109824987	0.00287721	0.06038225	Down in BrM
662	TRIM2	Q9C040	tripartite motif containing 2		-1.571807743	0.00076321	0.03899415	Down in BrM
663	ARHGAP39	Q9C0H5	Rho GTPase activating protein 39		-1.417194105	0.0015804	0.05084199	Down in BrM
664	SRCIN1	Q9C0H9	SRC kinase signaling inhibitor 1		-2.141299497	0.00010989	0.02146046	Down in BrM
665	PITHD1	Q9GZP4	PITH domain containing 1		-1.437535462	0.00549186	0.07563206	Down in BrM
666	SRR	Q9GZT4	serine racemase	Pyridoxal phosphate; Serine	-1.386951139	0.00089081	0.04142282	Down in BrM
667	HAPLN2	Q9GZV7	hyaluronan and proteoglycan link protein 2		-2.857862823	0.00541395	0.07550546	Down in BrM
668	LHPP	Q9H008	phospholysine phosphohistidine inorganic pyrophosphate phosphatase		-1.481831026	0.00554054	0.07575872	Down in BrM
669	FXYD6	Q9H0Q3	FXYD domain containing ion transport regulator 6		-2.375278847	0.00300665	0.06132632	Down in BrM
670	HDHD2	Q9H0R4	haloacid dehalogenase like hydrolase domain containing 2		-1.280053249	0.00950089	0.08874968	Down in BrM
671	GABARAPL1	Q9H0R8	GABA type A receptor associated protein like 1		-1.663663277	0.00048407	0.03562344	Down in BrM
672	NAPB	Q9H115	NSF attachment protein beta		-2.636124992	0.00884583	0.08594982	Down in BrM
673	EPN3	Q9H201	epsin 3		-1.508545098	0.00030426	0.03255968	Down in BrM
674	SPTBN4	Q9H254	spectrin beta, non-erythrocytic 4		-1.549771495	0.00337473	0.06387132	Down in BrM
675	FN3K	Q9H479	fructosamine 3 kinase		-1.583664018	0.00386206	0.06630862	Down in BrM
676	MAP1LC3A	Q9H492	microtubule associated protein 1 light chain 3 alpha		-2.455019798	0.00259144	0.05914721	Down in BrM
677	EPB41L1	Q9H4G0	erythrocyte membrane protein band 4.1 like 1		-1.650833245	0.00285312	0.06038225	Down in BrM
678	MAP6D1	Q9H9H5	MAP6 domain containing 1		-2.657119609	0.0000749	0.02036635	Down in BrM
679	CELSR2	Q9HCU4	cadherin EGF LAG seven-pass G-type receptor 2		-1.250380504	0.00016484	0.02524896	Down in BrM
680	RTN4	Q9NQC3	reticulon 4		-1.486177969	0.00449114	0.06977687	Down in BrM

681	HINT3	Q9NQE9	histidine triad nucleotide binding protein 3		-1.27142784	0.00511766	0.07322183	Down in BrM
682	PLSCR4	Q9NRQ2	phospholipid scramblase 4		-1.452040607	0.00136399	0.04910359	Down in BrM
683	CDC42SE	Q9NRR3	CDC42 small effector 2		-1.547288044	0.00023452	0.03118214	Down in BrM
684	BRWD1	Q9NSI6	bromodomain and WD repeat domain containing 1		-1.532692819	0.00321799	0.06316325	Down in BrM
685	LRRC4B	Q9NT99	leucine rich repeat containing 4B		-1.327746482	0.00437109	0.06940432	Down in BrM
686	SEPTIN11	Q9NVA2	septin 11		-1.421845218	0.0036749	0.06474551	Down in BrM
687	TUBA8	Q9NY65	tubulin alpha 8	Epothilone D; Patupilone	-2.676194983	0.00209215	0.05549443	Down in BrM
688	NKIRAS1	Q9NYS0	NFKB inhibitor interacting Ras like 1		-1.690976638	0.0000882	0.02036635	Down in BrM
689	FMN2	Q9NZ56	formin 2		-1.147122507	0.00895431	0.08635936	Down in BrM
690	GPRC5B	Q9NZH0	G protein-coupled receptor class C group 5 member B		-2.382489874	0.0000828	0.02036635	Down in BrM
691	SACS	Q9NZJ4	sacsin molecular chaperone		-1.4592417	0.00000656	0.01358434	Down in BrM
692	TMOD2	Q9NZR1	tropomodulin 2		-2.272249663	0.0018065	0.05179296	Down in BrM
693	ADAM22	Q9P0K1	ADAM metallopeptidase domain 22		-2.013054937	0.001496	0.04998058	Down in BrM
694	PIPOX	Q9P0Z9	pipecolic acid and sarcosine oxidase	Glycine	-1.139295971	0.0015396	0.05056962	Down in BrM
695	NTM	Q9P121	neurotrimin		-2.11420108	0.00511374	0.07322183	Down in BrM
696	ZNF219	Q9P2Y4	zinc finger protein 219		-2.550213707	0.00465044	0.07026935	Down in BrM
697	NCDN	Q9UBB6	neurochondrin		-2.471653817	0.00973764	0.08970571	Down in BrM
698	CLIP2	Q9UDT6	CAP-Gly domain containing linker protein 2		-1.458166913	0.0000366	0.02036635	Down in BrM
699	SEPTIN3	Q9UH03	septin 3		-2.529581227	0.00789222	0.08242517	Down in BrM
700	ENOPH1	Q9UHY7	enolase-phosphatase 1	2-OXOHEPTYLPHOSPHONIC ACID	-1.276064229	0.00023632	0.03118214	Down in BrM
701	SH3BGRL	Q9UJC5	SH3 domain binding glutamate rich protein like 2		-1.309399491	0.00166025	0.05084199	Down in BrM
702	FBXO2	Q9UK22	F-box protein 2		-2.182291766	0.00559016	0.07575872	Down in BrM
703	ACSL6	Q9UKU0	acyl-CoA synthetase long chain family member 6		-1.871008446	0.00147986	0.04998058	Down in BrM
704	HCN2	Q9UL51	hyperpolarization activated cyclic nucleotide gated potassium and sodium channel 2	Cyclic GMP; Cyclic adenosine monophosphate; Ivabradine	-1.604954341	0.00149463	0.04998058	Down in BrM
705	NDRG4	Q9ULP0	NDRG family member 4		-2.386842787	0.00627962	0.07752356	Down in BrM
706	NDRG2	Q9UN36	NDRG family member 2		-2.124718967	0.00274082	0.05914721	Down in BrM
707	MAPRE3	Q9UPY8	microtubule associated protein RP/EB family member 3		-1.719602727	0.00239683	0.05823781	Down in BrM
708	CORO2B	Q9UQ03	coronin 2B		-2.086407272	0.00127514	0.04752087	Down in BrM
709	DNM3	Q9UQ16	dynamin 3		-1.970995203	0.00299872	0.06132467	Down in BrM
710	CTNND2	Q9UQB3	catenin delta 2		-1.578510436	0.00039635	0.03478999	Down in BrM
711	CFL2	Q9Y281	cofilin 2		-1.779498545	0.00287467	0.06038225	Down in BrM
712	DIP2C	Q9Y2E4	disco interacting protein 2 homolog C		-1.217019398	0.00129569	0.04752087	Down in BrM
713	EPB41L3	Q9Y2J2	erythrocyte membrane protein band 4.1 like 3		-2.133401307	0.00100696	0.04322166	Down in BrM
714	CRYL1	Q9Y2S2	crystallin lambda 1		-1.657380795	0.0090032	0.08661177	Down in BrM
715	SNX24	Q9Y343	sorting nexin 24		-1.059230991	0.00674752	0.07938502	Down in BrM
716	MTCL1	Q9Y4B5	microtubule crosslinking factor 1		-1.228130103	0.00378109	0.06593267	Down in BrM
717	MYO5A	Q9Y4I1	myosin VA		-2.011871367	0.00654732	0.07906805	Down in BrM
718	DTNA	Q9Y4J8	dystrobrevin alpha		-1.595361676	0.00012759	0.02313639	Down in BrM
719	SHANK1	Q9Y566	SH3 and multiple ankyrin repeat domains 1		-2.191020696	0.00420021	0.06814947	Down in BrM
720	PSAT1	Q9Y617	phosphoserine aminotransferase 1	Pyridoxal phosphate; Glutamic acid	-1.859373961	0.00498744	0.07268088	Down in BrM

supplementary table 3 Enriched pathways in fig2e									
**GroupID**	**Category**	**Term**	**Description**	**LogP**	**Log(q-value)**	**Genes**	**Symbols**	**InTerm_InList**	**Group**
1_Summary	GO Biological Processes	GO:0031175	neuron projection development	-24.52961814	-20.35381651	1002,1267,1272,1496,1501,1952,1996,2534,2596,3736,3800,3925,3996,4130,4131,4135,4137,4685,4744,4747,4852,4897,4974,5306,5364,5803,5881,6285,6456,6900,8482,8506,8522,9368,9638,9922,10381,10752,23111,23114,23136,23154,26227,50944,56896,57142,57731,349667,287,1073,1465,4703,8898,10529,11076,29767,6507,1000,5027,10486	CDH4,CNP,CNTN1,CTNNA2,CTNND2,CELSR2,ELAVL4,FYN,GAP43,KCNA1,KIF5C,STMN1,LLGL1,MAP1A,MAP1B,MAP6,MAPT,NCAM2,NEFH,NEFL,NPY,NRCAM,OMG,PITPNA,PLXNB1,PTPRZ1,RAC3,S100B,SH3GL2,CNTN2,SEMA7A,CNTNAP1,GAS7,NHERF1,FEZ1,IQSEC1,TUBB3,CHL1,SPART,NFASC,EPB41L3,NCDN,PHGDH,SHANK1,DPYSL5,RTN4,SPTBN4,RTN4RL2,ANK2,CFL2,CSRP1,NEB,MTMR2,NEBL,TPPP,TMOD2,SLC1A3,CDH2,P2RX7,CAP2	60/-	Down in BrM
2_Summary	GO Biological Processes	GO:0050808	synapse organization	-18.34344415	-15.12188502	1000,1501,2534,2554,2561,4131,4137,4744,4747,4897,5064,5217,5300,5364,5621,5789,5881,6620,6623,6900,8506,8898,9118,10486,26052,50944,57731,80725,80728,94030,287,667,1002,2697,10391,23136,816,9762,1496,55752	CDH2,CTNND2,FYN,GABRA1,GABRB2,MAP1B,MAPT,NEFH,NEFL,NRCAM,PALM,PFN2,PIN1,PLXNB1,PRNP,PTPRD,RAC3,SNCB,SNCG,CNTN2,CNTNAP1,MTMR2,INA,CAP2,DNM3,SHANK1,SPTBN4,SRCIN1,ARHGAP39,LRRC4B,ANK2,DST,CDH4,GJA1,CORO2B,EPB41L3,CAMK2B,LZTS3,CTNNA2,SEPTIN11	40/-	Down in BrM
3_Summary	GO Biological Processes	GO:0120035	regulation of plasma membrane bounded cell projection organization	-17.4943799	-14.35997095	482,816,989,1000,1002,1272,1400,1496,1996,2281,2534,2596,2664,3265,4131,4135,4137,4504,4747,4897,5027,5064,5217,5364,5621,5789,8482,9638,9762,22902,23111,26052,56896,57142,65009,79598,80725,347733,349667	ATP1B2,CAMK2B,SEPTIN7,CDH2,CDH4,CNTN1,CRMP1,CTNNA2,ELAVL4,FKBP1B,FYN,GAP43,GDI1,HRAS,MAP1B,MAP6,MAPT,MT3,NEFL,NRCAM,P2RX7,PALM,PFN2,PLXNB1,PRNP,PTPRD,SEMA7A,FEZ1,LZTS3,RUFY3,SPART,DNM3,DPYSL5,RTN4,NDRG4,CEP97,SRCIN1,TUBB2B,RTN4RL2	39/-	Down in BrM
4_Summary	GO Biological Processes	GO:0050804	modulation of chemical synaptic transmission	-17.27929394	-14.19050308	477,590,816,1000,1759,2534,2911,3265,4130,4131,4137,4355,4744,4747,5217,5594,5621,5789,6285,6507,6623,8898,9118,9581,10815,23154,26232,50944,54899,57142,57537,64854,347733,392862	ATP1A2,BCHE,CAMK2B,CDH2,DNM1,FYN,GRM1,HRAS,MAP1A,MAP1B,MAPT,MPP2,NEFH,NEFL,PFN2,MAPK1,PRNP,PTPRD,S100B,SLC1A3,SNCG,MTMR2,INA,PREPL,CPLX1,NCDN,FBXO2,SHANK1,PXK,RTN4,SORCS2,USP46,TUBB2B,GRID2IP	34/-	Down in BrM
5_Summary	GO Biological Processes	GO:0099003	vesicle-mediated transport in synapse	-15.61287285	-12.61316248	273,274,523,526,528,529,534,1000,1759,5027,6453,6456,6620,6623,8867,9829,9892,10815,22848,26052,287,477,478,481,482,610,1212,1808,2534,2911,3265,5594,5881,6507,6511,9368,22905,55040,56990,3800,1780,4644,23122	AMPH,BIN1,ATP6V1A,ATP6V1B2,ATP6V1C1,ATP6V1E1,ATP6V1G2,CDH2,DNM1,P2RX7,ITSN1,SH3GL2,SNCB,SNCG,SYNJ1,DNAJC6,SNAP91,CPLX1,AAK1,DNM3,ANK2,ATP1A2,ATP1A3,ATP1B1,ATP1B2,HCN2,CLTB,DPYSL2,FYN,GRM1,HRAS,MAPK1,RAC3,SLC1A3,SLC1A6,NHERF1,EPN2,EPN3,CDC42SE2,KIF5C,DYNC1I1,MYO5A,CLASP2	43/-	Down in BrM
6_Summary	GO Biological Processes	GO:0051493	regulation of cytoskeleton organization	-14.80019307	-11.90314504	118,274,1073,1496,1901,2770,3265,3925,4130,4131,4135,4137,4703,5027,5217,5364,5594,5881,6709,6711,10391,10982,11076,22924,23122,29767,50944,57026,57731,79598,79929,79969,1400,2534,2664,4504,5621,8482,8898,9638,22902,22933,23111,26052,26278,55466,56896,57142,146691,258010,349667,287,477,9829,23176,63908,84630	ADD1,BIN1,CFL2,CTNNA2,S1PR1,GNAI1,HRAS,STMN1,MAP1A,MAP1B,MAP6,MAPT,NEB,P2RX7,PFN2,PLXNB1,MAPK1,RAC3,SPTAN1,SPTBN1,CORO2B,MAPRE2,TPPP,MAPRE3,CLASP2,TMOD2,SHANK1,PDXP,SPTBN4,CEP97,MAP6D1,ATAT1,CRMP1,FYN,GDI1,MT3,PRNP,SEMA7A,MTMR2,FEZ1,RUFY3,SIRT2,SPART,DNM3,SACS,DNAJA4,DPYSL5,RTN4,TOM1L2,SVIP,RTN4RL2,ANK2,ATP1A2,DNAJC6,SEPTIN8,NAPB,TTBK1	57/-	Down in BrM
7_Summary	GO Biological Processes	GO:0030029	actin filament-based process	-13.64393695	-10.76916531	118,287,1073,1465,1901,2036,2037,2697,3996,4082,4644,4703,5217,5881,5910,6330,6709,6711,8522,9368,9922,10391,10486,10529,22808,22902,23136,29767,56776,57026,57731,80725,221061,1155,3892,3925,4137,4741,4744,4747,7461,9118,11076,23122	ADD1,ANK2,CFL2,CSRP1,S1PR1,EPB41L1,EPB41L2,GJA1,LLGL1,MARCKS,MYO5A,NEB,PFN2,RAC3,RAP1GDS1,SCN4B,SPTAN1,SPTBN1,GAS7,NHERF1,IQSEC1,CORO2B,CAP2,NEBL,MRAS,RUFY3,EPB41L3,TMOD2,FMN2,PDXP,SPTBN4,SRCIN1,FAM171A1,TBCB,KRT86,STMN1,MAPT,NEFM,NEFH,NEFL,CLIP2,INA,TPPP,CLASP2	44/-	Down in BrM
8_Summary	GO Biological Processes	GO:0042391	regulation of membrane potential	-13.27249651	-10.41891417	18,274,287,477,478,481,482,610,2273,2281,2554,2561,2697,2776,2911,3736,4137,4355,4897,5027,5348,6330,6511,7531,8506,8898,23111,50944,57731,491,816,6271,9368,10391,11076,58494,1272,2534,5533,5621,53826,80333,54899,8682,10776,6507,1827,5217,11345	ABAT,BIN1,ANK2,ATP1A2,ATP1A3,ATP1B1,ATP1B2,HCN2,FHL1,FKBP1B,GABRA1,GABRB2,GJA1,GNAQ,GRM1,KCNA1,MAPT,MPP2,NRCAM,P2RX7,FXYD1,SCN4B,SLC1A6,YWHAE,CNTNAP1,MTMR2,SPART,SHANK1,SPTBN4,ATP2B2,CAMK2B,S100A1,NHERF1,CORO2B,TPPP,JAM2,CNTN1,FYN,PPP3CC,PRNP,FXYD6,KCNIP4,PXK,PEA15,ARPP19,SLC1A3,RCAN1,PFN2,GABARAPL2	49/-	Down in BrM
9_Summary	GO Biological Processes	GO:0060322	head development	-12.89685895	-10.08278516	18,477,482,1000,1152,1267,1272,1496,1901,1952,1996,2534,3736,4747,4852,4897,5594,5881,6252,6271,7280,7531,8482,9118,9638,22933,23114,26227,57142,57447,79969,84159,84630,140733,166614,347733	ABAT,ATP1A2,ATP1B2,CDH2,CKB,CNP,CNTN1,CTNNA2,S1PR1,CELSR2,ELAVL4,FYN,KCNA1,NEFL,NPY,NRCAM,MAPK1,RAC3,RTN1,S100A1,TUBB2A,YWHAE,SEMA7A,INA,FEZ1,SIRT2,NFASC,PHGDH,RTN4,NDRG2,ATAT1,ARID5B,TTBK1,MACROD2,DCLK2,TUBB2B	36/-	Down in BrM
10_Summary	GO Biological Processes	GO:0007610	behavior	-12.78172581	-9.986135417	18,477,482,590,1267,1272,1827,1996,2534,2788,2911,4130,4137,4185,4852,5594,5621,5803,6285,6507,6623,6900,8867,10752,29767,50944,55074,57731,64854,65009,84630,163175	ABAT,ATP1A2,ATP1B2,BCHE,CNP,CNTN1,RCAN1,ELAVL4,FYN,GNG7,GRM1,MAP1A,MAPT,ADAM11,NPY,MAPK1,PRNP,PTPRZ1,S100B,SLC1A3,SNCG,CNTN2,SYNJ1,CHL1,TMOD2,SHANK1,OXR1,SPTBN4,USP46,NDRG4,TTBK1,LGI4	32/-	Down in BrM
11_Summary	GO Biological Processes	GO:0032535	regulation of cellular component size	-12.6073708	-9.846542511	118,274,361,1002,1073,1759,2664,4131,4137,4504,4703,4747,4897,5027,5217,6709,6711,8482,9368,22902,23111,29767,57026,57142,57731,176,816,4135,5364,5789,5803,8898,11076,22933,56896,58494,60484,84630,94030,163175,477,5910,1000,1400,2534,5621,26052,79598,349667,3707,2273,2697,2596	ADD1,BIN1,AQP4,CDH4,CFL2,DNM1,GDI1,MAP1B,MAPT,MT3,NEB,NEFL,NRCAM,P2RX7,PFN2,SPTAN1,SPTBN1,SEMA7A,NHERF1,RUFY3,SPART,TMOD2,PDXP,RTN4,SPTBN4,ACAN,CAMK2B,MAP6,PLXNB1,PTPRD,PTPRZ1,MTMR2,TPPP,SIRT2,DPYSL5,JAM2,HAPLN2,TTBK1,LRRC4B,LGI4,ATP1A2,RAP1GDS1,CDH2,CRMP1,FYN,PRNP,DNM3,CEP97,RTN4RL2,ITPKB,FHL1,GJA1,GAP43	53/-	Down in BrM
12_Summary	GO Biological Processes	GO:0000226	microtubule cytoskeleton organization	-12.55968131	-9.815243438	1155,1783,2697,3925,3996,4130,4131,4135,4137,4744,4747,7280,7461,10376,10381,10383,10982,11076,22924,23122,23255,24137,51807,56776,79861,79929,79969,84617,166614,347733,6711,10776,22933,219771	TBCB,DYNC1LI2,GJA1,STMN1,LLGL1,MAP1A,MAP1B,MAP6,MAPT,NEFH,NEFL,TUBB2A,CLIP2,TUBA1B,TUBB3,TUBB4B,MAPRE2,TPPP,MAPRE3,CLASP2,MTCL1,KIF4A,TUBA8,FMN2,TUBAL3,MAP6D1,ATAT1,TUBB6,DCLK2,TUBB2B,SPTBN1,ARPP19,SIRT2,CCNY	34/-	Down in BrM
13_Summary	GO Biological Processes	GO:0019725	cellular homeostasis	-9.581590093	-6.996853067	287,361,477,478,481,482,491,523,526,528,529,534,1073,2281,2911,4130,4504,5027,5213,5594,5621,5910,6507,7531,9368,9531,23241,57142,392862,3736,610,2534,5333,5348,6330,80333,2554,2561,94015	ANK2,AQP4,ATP1A2,ATP1A3,ATP1B1,ATP1B2,ATP2B2,ATP6V1A,ATP6V1B2,ATP6V1C1,ATP6V1E1,ATP6V1G2,CFL2,FKBP1B,GRM1,MAP1A,MT3,P2RX7,PFKM,MAPK1,PRNP,RAP1GDS1,SLC1A3,YWHAE,NHERF1,BAG3,PACS2,RTN4,GRID2IP,KCNA1,HCN2,FYN,PLCD1,FXYD1,SCN4B,KCNIP4,GABRA1,GABRB2,TTYH2	39/-	Down in BrM
14_Summary	GO Biological Processes	GO:0030705	cytoskeleton-dependent intracellular transport	-9.560221107	-6.986479466	667,1740,1780,3281,3800,4130,4131,4135,4137,4644,4747,5813,9531,10376,24137,79929,361,1783,1952,5414,9368,55605,56776,50944,481,1212,8774,51308,54899,57537,63908	DST,DLG2,DYNC1I1,HSBP1,KIF5C,MAP1A,MAP1B,MAP6,MAPT,MYO5A,NEFL,PURA,BAG3,TUBA1B,KIF4A,MAP6D1,AQP4,DYNC1LI2,CELSR2,SEPTIN4,NHERF1,KIF21A,FMN2,SHANK1,ATP1B1,CLTB,NAPG,REEP2,PXK,SORCS2,NAPB	31/-	Down in BrM
15_Summary	GO Biological Processes	GO:0010001	glial cell differentiation	-8.842033535	-6.299700357	176,1000,1267,1272,2596,3265,4137,5594,5803,8506,8613,11076,22933,26227,60484,163175,57142,287,1073,1465,4703,8898,10529,23136,29767,23114,4744	ACAN,CDH2,CNP,CNTN1,GAP43,HRAS,MAPT,MAPK1,PTPRZ1,CNTNAP1,PLPP3,TPPP,SIRT2,PHGDH,HAPLN2,LGI4,RTN4,ANK2,CFL2,CSRP1,NEB,MTMR2,NEBL,EPB41L3,TMOD2,NFASC,NEFH	27/-	Down in BrM
16_Summary	GO Biological Processes	GO:0099536	synaptic signaling	-8.295445496	-5.800885101	273,1267,1740,1837,2554,2561,2776,2911,4355,4852,5027,5594,5789,5881,6507,6511,6620,6623,8867,10815,29767,63908	AMPH,CNP,DLG2,DTNA,GABRA1,GABRB2,GNAQ,GRM1,MPP2,NPY,P2RX7,MAPK1,PTPRD,RAC3,SLC1A3,SLC1A6,SNCB,SNCG,SYNJ1,CPLX1,TMOD2,NAPB	22/-	Down in BrM
17_Summary	GO Biological Processes	GO:0061024	membrane organization	-7.919486863	-5.507113224	274,287,481,1000,1267,1759,4897,5027,5306,5375,5789,5881,6711,6900,8396,8774,8867,9368,9892,22848,22933,23136,26052,51308,57088,57142,57731,92154,4644,5064,9581,23122,23241,80333,1740,54557,3996,523,6252	BIN1,ANK2,ATP1B1,CDH2,CNP,DNM1,NRCAM,P2RX7,PITPNA,PMP2,PTPRD,RAC3,SPTBN1,CNTN2,PIP4K2B,NAPG,SYNJ1,NHERF1,SNAP91,AAK1,SIRT2,EPB41L3,DNM3,REEP2,PLSCR4,RTN4,SPTBN4,MTSS2,MYO5A,PALM,PREPL,CLASP2,PACS2,KCNIP4,DLG2,SGTB,LLGL1,ATP6V1A,RTN1	39/-	Down in BrM
18_Summary	GO Biological Processes	GO:0070507	regulation of microtubule cytoskeleton organization	-7.772656893	-5.382185096	2770,3925,4130,4131,4135,4137,10982,11076,22924,23122,79598,79929,79969,4744,9638	GNAI1,STMN1,MAP1A,MAP1B,MAP6,MAPT,MAPRE2,TPPP,MAPRE3,CLASP2,CEP97,MAP6D1,ATAT1,NEFH,FEZ1	15/-	Down in BrM
19_Summary	GO Biological Processes	GO:0021762	substantia nigra development	-7.752268754	-5.368858811	1152,1267,6271,7531,9118,22933,57447,84630	CKB,CNP,S100A1,YWHAE,INA,SIRT2,NDRG2,TTBK1	8/-	Down in BrM
20_Summary	GO Biological Processes	GO:0009063	amino acid catabolic process	-7.640911421	-5.291184591	18,549,883,1428,2752,2805,5860,10247,23576,51268,80150,26227,29968,58478,63826,6507,230,2026,4190,5213,10840,23305,51084,64122,153364,57026,3612,3707	ABAT,AUH,KYAT1,CRYM,GLUL,GOT1,QDPR,RIDA,DDAH1,PIPOX,ASRGL1,PHGDH,PSAT1,ENOPH1,SRR,SLC1A3,ALDOC,ENO2,MDH1,PFKM,ALDH1L1,ACSL6,CRYL1,FN3K,MBLAC2,PDXP,IMPA1,ITPKB	28/-	Down in BrM

1_Summary	GO Biological Processes	GO:0032543	mitochondrial translation	-36.63915771	-32.46335608	9553,10573,11222,28957,29074,29088,29093,51021,51116,51373,51642,51650,54460,54948,55037,55052,55173,56945,57129,63931,64965,64968,64969,64975,65080,78988,79590,80222,84545,116540,116541,122704,124995,219927,51106,165,290,1362,1803,1889,2028,5034,6156,9789,10347,23438,23478,23521,25873,25973,28972,79731,81502,92935,138428	MRPL33,MRPL28,MRPL3,MRPS28,MRPL18,MRPL15,MRPL22,MRPS16,MRPS2,MRPS17,MRPL48,MRPS33,MRPS21,MRPL16,PTCD3,MRPL20,MRPS10,MRPS22,MRPL47,MRPS14,MRPS9,MRPS6,MRPS5,MRPL41,MRPL44,MRPL57,MRPL24,TARS2,MRPL43,MRPL53,MRPL54,MRPL52,MRPL10,MRPL21,TFB1M,AEBP1,ANPEP,CPD,DPP4,ECE1,ENPEP,P4HB,RPL30,SPCS2,ABCA7,HARS2,SEC11A,RPL13A,RPL36,PARS2,SPCS1,NARS2,HM13,MARS2,PTRH1	55/-	Up in BrM
2_Summary	GO Biological Processes	GO:0042254	ribosome biogenesis	-34.74531538	-30.87054374	705,4839,8568,9790,9875,10171,10199,10360,10607,10885,11056,23212,23378,23481,27043,28987,51096,51106,51116,51154,51388,51491,54433,54475,54606,54663,55052,55226,55505,55661,55720,55759,55794,55813,57109,65095,79707,79954,84365,84916,85865,92856,115416,117246,134430,285855,317781,8487,9169,9631,23438,25973,51574,55596,56931,79042,79691,79731,80222,92935	BYSL,NOP2,RRP1,BMS1,URB1,RCL1,MPHOSPH10,NPM3,TBL3,WDR3,DDX52,RRS1,RRP8,PES1,PELP1,NOB1,UTP18,TFB1M,MRPS2,MRTO4,NIP7,NOP16,GAR1,NLE1,DDX56,WDR74,MRPL20,NAT10,NOP10,DDX27,TSR1,WDR12,DDX28,UTP6,REXO4,KRI1,NOL9,NOL10,NIFK,UTP4,GTPBP10,IMP4,MALSU1,FTSJ3,WDR36,RPL7L1,DDX51,GEMIN2,SCAF11,NUP155,HARS2,PARS2,LARP7,ZCCHC8,DUS3L,TSEN34,QTRT2,NARS2,TARS2,MARS2	60/-	Up in BrM
3_Summary	GO Biological Processes	GO:0051604	protein maturation	-20.22408728	-17.12746689	165,811,871,1362,1803,1889,2028,2923,3309,3998,4323,5034,5327,5479,5611,5768,7184,9601,9789,10130,10491,10549,10845,10954,10961,11328,23071,23173,23478,23753,27348,28972,30001,51303,51726,56886,60681,63929,64374,81502,8156	AEBP1,CALR,SERPINH1,CPD,DPP4,ECE1,ENPEP,PDIA3,HSPA5,LMAN1,MMP14,P4HB,PLAT,PPIB,DNAJC3,QSOX1,HSP90B1,PDIA4,SPCS2,PDIA6,CRTAP,PRDX4,CLPX,PDIA5,ERP29,FKBP9,ERP44,METAP1,SEC11A,SDF2L1,TOR1B,SPCS1,ERO1A,FKBP11,DNAJB11,UGGT1,FKBP10,XPNPEP3,SIL1,HM13,TXNDC5	41/-	Up in BrM
4_Summary	GO Biological Processes	GO:0042273	ribosomal large subunit biogenesis	-16.04876632	-13.07708467	4839,9875,23212,23481,51154,51388,51491,54475,54663,55052,55759,55794,79707,84365,115416,117246,285855,10171,65095	NOP2,URB1,RRS1,PES1,MRTO4,NIP7,NOP16,NLE1,WDR74,MRPL20,WDR12,DDX28,NOL9,NIFK,MALSU1,FTSJ3,RPL7L1,RCL1,KRI1	19/-	Up in BrM
5_Summary	GO Biological Processes	GO:0034976	response to endoplasmic reticulum stress	-12.32260914	-9.402080011	317,811,2923,3309,5034,5371,5611,7184,9601,10130,10952,10956,10959,23071,23753,30001,51009,55829,56886,80267,81502,201595,871,7873,23456,27348,3074,11079,948,6713,634,3949,9123,10845	APAF1,CALR,PDIA3,HSPA5,P4HB,PML,DNAJC3,HSP90B1,PDIA4,PDIA6,SEC61B,OS9,TMED2,ERP44,SDF2L1,ERO1A,DERL2,SELENOS,UGGT1,EDEM3,HM13,STT3B,SERPINH1,MANF,ABCB10,TOR1B,HEXB,RER1,CD36,SQLE,CEACAM1,LDLR,SLC16A3,CLPX	34/-	Up in BrM
6_Summary	GO Biological Processes	GO:0034975	protein folding in endoplasmic reticulum	-10.68583727	-7.788789238	811,2923,3309,5034,5611,7184,30001,10631	CALR,PDIA3,HSPA5,P4HB,DNAJC3,HSP90B1,ERO1A,POSTN	8/-	Up in BrM
7_Summary	GO Biological Processes	GO:0009100	glycoprotein metabolic process	-10.66034939	-7.785577753	1603,1650,2589,3074,4125,5373,6185,7358,8693,8985,9653,9761,10678,11227,23071,23509,29071,29929,56886,80267,84061,200185,201595,347734,204,1468,1841,4907,7040,9375,10201,80146	DAD1,DDOST,GALNT1,HEXB,MAN2B1,PMM2,RPN2,UGDH,GALNT4,PLOD3,HS2ST1,MLEC,B3GNT2,GALNT5,ERP44,POFUT1,C1GALT1C1,ALG6,UGGT1,EDEM3,MAGT1,KRTCAP2,STT3B,SLC35B2,AK2,SLC25A10,DTYMK,NT5E,TGFB1,TM9SF2,NME6,UXS1	32/-	Up in BrM
8_Summary	GO Biological Processes	GO:0000479	endonucleolytic cleavage of tricistronic rRNA transcript (SSU-rRNA, 5.8S rRNA, LSU-rRNA)	-8.945132982	-6.21648938	9790,10171,10607,23212,55720,65095,79707	BMS1,RCL1,TBL3,RRS1,TSR1,KRI1,NOL9	7/-	Up in BrM
9_Summary	GO Biological Processes	GO:0030199	collagen fibril organization	-8.228764713	-5.574870337	871,1278,1281,1290,4060,5033,8985,10491,60681,79709,4323,5768,7040,7045,10549,10631,30001,255743,317,5371,3856,5111	SERPINH1,COL1A2,COL3A1,COL5A2,LUM,P4HA1,PLOD3,CRTAP,FKBP10,COLGALT1,MMP14,QSOX1,TGFB1,TGFBI,PRDX4,POSTN,ERO1A,NPNT,APAF1,PML,KRT8,PCNA	22/-	Up in BrM
10_Summary	GO Biological Processes	GO:0006886	intracellular protein transport	-8.003936237	-5.372202649	811,3309,4074,4928,5371,6734,6809,7040,7184,9409,9631,10139,10628,10802,10952,10956,10959,10961,10972,23636,50999,51009,54732,55829,64374,81502,140823,637,983,1729,3009,5018,6747,9789,10347,10664,11079,23212,26092,27348,28972,999,3728,3875,6745,9375,10627,50848	CALR,HSPA5,M6PR,NUP98,PML,SRPRA,STX3,TGFB1,HSP90B1,PEX16,NUP155,ARFRP1,TXNIP,SEC24A,SEC61B,OS9,TMED2,ERP29,TMED10,NUP62,TMED5,DERL2,TMED9,SELENOS,SIL1,HM13,ROMO1,BID,CDK1,DIAPH1,H1-5,OXA1L,SSR3,SPCS2,ABCA7,CTCF,RER1,RRS1,TOR1AIP1,TOR1B,SPCS1,CDH1,JUP,KRT18,SSR1,TM9SF2,MYL12A,F11R	48/-	Up in BrM
11_Summary	GO Biological Processes	GO:0042060	wound healing	-7.968358183	-5.348859051	634,948,977,1265,1281,1832,2677,2923,3678,3998,4627,5327,5754,6385,7040,10627,23173,29071,60681,84062,983,1829,3728,7391,8673	CEACAM1,CD36,CD151,CNN2,COL3A1,DSP,GGCX,PDIA3,ITGA5,LMAN1,MYH9,PLAT,PTK7,SDC4,TGFB1,MYL12A,METAP1,C1GALT1C1,FKBP10,DTNBP1,CDK1,DSG2,JUP,USF1,VAMP8	25/-	Up in BrM
12_Summary	GO Biological Processes	GO:0044403	biological process involved in symbiotic interaction	-6.412503482	-3.899535246	290,983,1105,1803,3482,3678,3949,4179,5479,6510,7037,8673,50848,54606,140823,440026,2589,5725,7391,28972	ANPEP,CDK1,CHD1,DPP4,IGF2R,ITGA5,LDLR,CD46,PPIB,SLC1A5,TFRC,VAMP8,F11R,DDX56,ROMO1,TMEM41B,GALNT1,PTBP1,USF1,SPCS1	20/-	Up in BrM
13_Summary	GO Biological Processes	GO:0006997	nucleus organization	-6.39663643	-3.899535246	983,3148,4928,5371,7443,8815,9631,23378,26092,27348,51280,79188,637,948,1803,4627,5018,6809,8673,10347,10802,10959,10972,11079,27069,51272,54732,140823,440026,3998,50999,84062	CDK1,HMGB2,NUP98,PML,VRK1,BANF1,NUP155,RRP8,TOR1AIP1,TOR1B,GOLM1,TMEM43,BID,CD36,DPP4,MYH9,OXA1L,STX3,VAMP8,ABCA7,SEC24A,TMED2,TMED10,RER1,GHITM,BET1L,TMED9,ROMO1,TMEM41B,LMAN1,TMED5,DTNBP1	32/-	Up in BrM
14_Summary	GO Biological Processes	GO:0097435	supramolecular fiber organization	-6.394095642	-3.899535246	637,871,948,1265,1278,1281,1290,1729,1832,2059,3856,4060,5033,6840,7184,7408,8985,9050,9119,10491,29766,55761,60681,79709,338785,811,4627,4637,5754,6464,9631,50848,84062,653781	BID,SERPINH1,CD36,CNN2,COL1A2,COL3A1,COL5A2,DIAPH1,DSP,EPS8,KRT8,LUM,P4HA1,SVIL,HSP90B1,VASP,PLOD3,PSTPIP2,KRT75,CRTAP,TMOD3,TTC17,FKBP10,COLGALT1,KRT79,CALR,MYH9,MYL6,PTK7,SHC1,NUP155,F11R,DTNBP1,POTEJ	34/-	Up in BrM
15_Summary	GO Biological Processes	GO:0018126	protein hydroxylation	-6.376163997	-3.890558444	5033,5034,5351,5352,8985,10491,5479,51661,60681,983,2589,2677,5371,7443,8693,23173,25865,54814,200185,5831,10588,23438,25973,29920,79731,80222,92935,948,1382,3074,7358,7923,9123,23042,83440,254552,390916,1468,1595,1841,4907,60386	P4HA1,P4HB,PLOD1,PLOD2,PLOD3,CRTAP,PPIB,FKBP7,FKBP10,CDK1,GALNT1,GGCX,PML,VRK1,GALNT4,METAP1,PRKD2,QPCTL,KRTCAP2,PYCR1,MTHFS,HARS2,PARS2,PYCR2,NARS2,TARS2,MARS2,CD36,CRABP2,HEXB,UGDH,HSD17B8,SLC16A3,PDXDC1,ADPGK,NUDT8,NUDT19,SLC25A10,CYP51A1,DTYMK,NT5E,SLC25A19	42/-	Up in BrM
16_Summary	GO Biological Processes	GO:0035239	tube morphogenesis	-6.31128883	-3.834457201	290,317,634,1281,1464,1942,2028,3678,3949,4323,4627,5371,5754,6385,6464,7040,7045,7052,7408,9653,10553,10959,23509,23670,25865,60681,255743,196,1278,10541,2896,3728,81	ANPEP,APAF1,CEACAM1,COL3A1,CSPG4,EFNA1,ENPEP,ITGA5,LDLR,MMP14,MYH9,PML,PTK7,SDC4,SHC1,TGFB1,TGFBI,TGM2,VASP,HS2ST1,HTATIP2,TMED2,POFUT1,CEMIP2,PRKD2,FKBP10,NPNT,AHR,COL1A2,ANP32B,GRN,JUP,CALR	33/-	Up in BrM
17_Summary	GO Biological Processes	GO:0016485	protein processing	-5.768115614	-3.332676671	165,1362,1803,1889,2028,4323,5034,5327,9789,23478,28972,63929,81502	AEBP1,CPD,DPP4,ECE1,ENPEP,MMP14,P4HB,PLAT,SPCS2,SEC11A,SPCS1,XPNPEP3,HM13	13/-	Up in BrM
18_Summary	GO Biological Processes	GO:0006979	response to oxidative stress	-5.670587927	-3.25066115	948,983,4323,5111,5831,6382,6780,8815,10549,10628,29920,29957,30001,55829,57190,81502,140823,493869,8531	CD36,CDK1,MMP14,PCNA,PYCR1,SDC1,STAU1,BANF1,PRDX4,TXNIP,PYCR2,SLC25A24,ERO1A,SELENOS,SELENON,HM13,ROMO1,GPX8,YBX3	19/-	Up in BrM
19_Summary	GO Biological Processes	GO:0051276	chromosome organization	-5.57589169	-3.185419892	983,3009,3148,3149,4172,4173,4928,5111,5984,8086,8520,8815,9631,23212,23244,23636,26073,27436,54433,55505,116447,2021,6282,317,5371,5977,7037,7040,25865,55226,4907,5937,6596,10664,57109,64318,637,7443,79073,221443	CDK1,H1-5,HMGB2,HMGB3,MCM3,MCM4,NUP98,PCNA,RFC4,AAAS,HAT1,BANF1,NUP155,RRS1,PDS5A,NUP62,POLDIP2,EML4,GAR1,NOP10,TOP1MT,ENDOG,S100A11,APAF1,PML,DPF2,TFRC,TGFB1,PRKD2,NAT10,NT5E,RBMS1,HLTF,CTCF,REXO4,NOC3L,BID,VRK1,TMEM109,OARD1	40/-	Up in BrM
20_Summary	GO Biological Processes	GO:0061077	chaperone-mediated protein folding	-5.223871468	-2.854249809	3309,5479,9601,10491,23753,27348,30001,51303,56886	HSPA5,PPIB,PDIA4,CRTAP,SDF2L1,TOR1B,ERO1A,FKBP11,UGGT1	9/-	Up in BrM

supplementary table 3 Enriched pathways in fig2h									
GroupID	Category	Term	Description	LogP	Log(q-value)	Genes	Symbols	InTerm_InList	Group
1_Summary	GO Biological Processes	GO:0009101	glycoprotein biosynthetic process	-11.59342939	-7.419166004	746,2131,2525,4248,6482,8702,8703,10195,22856,51006,51172,54480,54916,55454,64132,79070,79695,84752,84920,90161,113189,126792,374907,4321,7298,7378,65258	TMEM258,EXT1,FUT3,MGAT3,ST3GAL1,B4GALT4,B4GALT3,ALG3,CHSY1,SLC35C2,NAGPA,CHPF2,TMEM260,CSGALNACT2,XYLT2,POGLUT2,GALNT12,B3GNT9,ALG10,HS6ST2,CHST14,B3GALT6,B3GNT8,MMP12,TYMS,UPP1,MPPE1	27/-	BrM-specific
2_Summary	GO Biological Processes	GO:0006023	aminoglycan biosynthetic process	-8.832004788	-5.134862661	2131,6482,8702,22856,54480,55454,64132,84752,113189,126792,374907,3161,8993,90161,2328,55,9645	EXT1,ST3GAL1,B4GALT4,CHSY1,CHPF2,CSGALNACT2,XYLT2,B3GNT9,CHST14,B3GALT6,B3GNT8,HMMR,PGLYRP1,HS6ST2,FMO3,ACP3,MICAL2	17/-	BrM-specific
3_Summary	GO Biological Processes	GO:0051276	chromosome organization	-8.699073137	-5.126869747	641,891,1060,2177,3024,3364,3833,3835,4085,9212,9319,10403,10445,10609,29128,51548,51659,54892,64151,64785,79003,84296,91603,92797,221150,286053,9650,10018,11065,51537,3978,10474,29997,55872,128239,728642,79441,115106,54780,56160,2131,4739,79621,1201,3985,7462,55686,84946,146439,3169,7051,29777,440335	BLM,CCNB1,CENPC,FANCD2,H1-1,HUS1,KIFC1,KIF22,MAD2L1,AURKB,TRIP13,NDC80,MCRS1,P3H4,UHRF1,SIRT6,GINS2,NCAPG2,NCAPG,GINS3,MIS12,GINS4,ZNF830,HELB,SKA3,NSMCE2,MTFR1,BCL2L11,UBE2C,MTFP1,LIG1,TADA3,NOP53,PBK,IQGAP3,CDK11A,HAUS3,HAUS1,NSMCE4A,NSMCE3,EXT1,NEDD9,RNASEH2B,CLN3,LIMK2,LAT2,MREG,LTV1,BICDL2,FOXA1,TGM1,ABT1,SMIM22	53/-	BrM-specific
4_Summary	GO Biological Processes	GO:0045229	external encapsulating structure organization	-8.290629291	-5.008299588	101,1295,1805,1999,2131,3491,4017,4316,4317,4321,4327,6692,7051,10609,25903,30008,54507,89932,169044,1435,3169,5311,8321,9451,9620,9645,10018,25960,27286,64220,80004,115908,348093,28982,79893	ADAM8,COL8A1,DPT,ELF3,EXT1,CCN1,LOXL2,MMP7,MMP8,MMP12,MMP19,SPINT1,TGM1,P3H4,OLFML2B,EFEMP2,ADAMTSL4,PAPLN,COL22A1,CSF1,FOXA1,PKD2,FZD1,EIF2AK3,CELSR1,MICAL2,BCL2L11,ADGRA2,SRPX2,STRA6,ESRP2,CTHRC1,RBPMS2,FLVCR1,GGNBP2	35/-	BrM-specific
5_Summary	GO Biological Processes	GO:0006259	DNA metabolic process	-7.918527769	-4.698506898	641,2140,2177,2966,3364,3835,3978,5557,7298,8930,9319,9582,10445,10714,29128,29997,51548,51659,54780,56160,57646,60489,64785,79621,84296,91603,92667,92797,115004,200316,286053,4170,7023,9923,10018,27086	BLM,EYA3,FANCD2,GTF2H2,HUS1,KIF22,LIG1,PRIM1,TYMS,MBD4,TRIP13,APOBEC3B,MCRS1,POLD3,UHRF1,NOP53,SIRT6,GINS2,NSMCE4A,NSMCE3,USP28,APOBEC3G,GINS3,RNASEH2B,GINS4,ZNF830,MGME1,HELB,CGAS,APOBEC3F,NSMCE2,MCL1,TFAP4,ZBTB40,BCL2L11,FOXP1	36/-	BrM-specific
6_Summary	GO Biological Processes	GO:0042254	ribosome biogenesis	-6.725027831	-3.842492072	6838,10200,10799,29777,29997,51018,51042,51118,51187,55035,55319,64216,81875,84946,88745,90459,153443,196074,84811,10849,83640	SURF6,MPHOSPH6,RPP40,ABT1,NOP53,RRP15,ZNF593,UTP11,RSL24D1,NOL8,TMA16,TFB2M,ISG20L2,LTV1,RRP36,ERI1,SRFBP1,METTL15,BUD13,POLR1G,RAMAC	21/-	BrM-specific
7_Summary	GO Biological Processes	GO:0098609	cell-cell adhesion	-4.887671256	-2.315467866	101,1364,1825,2131,3491,3694,3695,4072,4973,8416,9620,10205,11187,25945,27134,27286,51148,51206,51294,55971,81607	ADAM8,CLDN4,DSC3,EXT1,CCN1,ITGB6,ITGB7,EPCAM,OLR1,ANXA9,CELSR1,MPZL2,PKP3,NECTIN3,TJP3,SRPX2,CERCAM,GP6,PCDH12,BAIAP2L1,NECTIN4	21/-	BrM-specific
8_Summary	GO Biological Processes	GO:0002062	chondrocyte differentiation	-4.848935708	-2.30286016	861,1836,2131,4054,9451,22856,25987,57045,1435,3169,3491,7298,54,415,3694,10234,27086,28982,4053,60436,1796,4739,4920,10849,29760,51206	RUNX1,SLC26A2,EXT1,LTBP3,EIF2AK3,CHSY1,TSKU,TWSG1,CSF1,FOXA1,CCN1,TYMS,ACP5,ARSL,ITGB6,LRRC17,FOXP1,FLVCR1,LTBP2,TGIF2,DOK1,NEDD9,ROR2,POLR1G,BLNK,GP6	26/-	BrM-specific
9_Summary	GO Biological Processes	GO:0009615	response to virus	-4.843655086	-2.30286016	2131,3588,3660,3694,4321,5452,5551,5987,9213,9582,11035,23765,51311,60489,91351,115004,200316,7378,55,7298,8930,671,837,1435,2219,5971,8685,8993,23569,23601,55647,55765,120224,121260,340205,51548,7113,27074,3161	EXT1,IL10RB,IRF2,ITGB6,MMP12,POU2F2,PRF1,TRIM27,XPR1,APOBEC3B,RIPK3,IL17RA,TLR8,APOBEC3G,DDX60L,CGAS,APOBEC3F,UPP1,ACP3,TYMS,MBD4,BPI,CASP4,CSF1,FCN1,RELB,MARCO,PGLYRP1,PADI4,CLEC5A,RAB20,INAVA,TMEM45B,SLC15A4,TREML1,SIRT6,TMPRSS2,LAMP3,HMMR	39/-	BrM-specific
10_Summary	GO Biological Processes	GO:0006261	DNA-templated DNA replication	-4.796516193	-2.265705489	641,3978,5557,10714,51659,64785,84296,91603,92667,92797	BLM,LIG1,PRIM1,POLD3,GINS2,GINS3,GINS4,ZNF830,MGME1,HELB	10/-	BrM-specific
11_Summary	GO Biological Processes	GO:1905820	positive regulation of chromosome separation	-4.475030674	-2.012330153	9212,11065,54892,64151,286053,891,10445,51548,3024	AURKB,UBE2C,NCAPG2,NCAPG,NSMCE2,CCNB1,MCRS1,SIRT6,H1-1	9/-	BrM-specific
12_Summary	GO Biological Processes	GO:0001819	positive regulation of cytokine production	-4.380461381	-1.93859176	101,728,861,2219,4317,4321,5452,5987,8288,9451,10148,23601,23765,27086,51043,51311,55765,56919,115004,2874,7105,9212,29997,59307,60343,60489,121260,200316,5971,837,1435,4739,5328	ADAM8,C5AR1,RUNX1,FCN1,MMP8,MMP12,POU2F2,TRIM27,EPX,EIF2AK3,EBI3,CLEC5A,IL17RA,FOXP1,ZBTB7B,TLR8,INAVA,DHX33,CGAS,GPS2,TSPAN6,AURKB,NOP53,SIGIRR,FAM3A,APOBEC3G,SLC15A4,APOBEC3F,RELB,CASP4,CSF1,NEDD9,PLA	33/-	BrM-specific
13_Summary	GO Biological Processes	GO:0007093	mitotic cell cycle checkpoint signaling	-4.319561187	-1.893485833	641,2177,3364,4085,9212,9319,10403,29997,91603,57646,891,5311,7023,11065,11319,79621,286053,3169,728642,54892,64151,2140,3694	BLM,FANCD2,HUS1,MAD2L1,AURKB,TRIP13,NDC80,NOP53,ZNF830,USP28,CCNB1,PKD2,TFAP4,UBE2C,ECD,RNASEH2B,NSMCE2,FOXA1,CDK11A,NCAPG2,NCAPG,EYA3,ITGB6	23/-	BrM-specific
14_Summary	GO Biological Processes	GO:0001701	in utero embryonic development	-4.160713555	-1.797625153	891,1825,1999,3491,4071,5311,6692,8178,10018,28982,51294,54892,79621,79893,84296,91603,2131,3169,3364,8321,9620,115908,1295,4317,9645,57045,64220,348093,4311,4297	CCNB1,DSC3,ELF3,CCN1,TM4SF1,PKD2,SPINT1,ELL,BCL2L11,FLVCR1,PCDH12,NCAPG2,RNASEH2B,GGNBP2,GINS4,ZNF830,EXT1,FOXA1,HUS1,FZD1,CELSR1,CTHRC1,COL8A1,MMP8,MICAL2,TWSG1,STRA6,RBPMS2,MME,KMT2A	30/-	BrM-specific
15_Summary	GO Biological Processes	GO:0031214	biomineral tissue development	-4.145947152	-1.797625153	3694,4054,4920,7286,9451,9923,54757,54829,861,1836,2131,3491,23601,57045,115908	ITGB6,LTBP3,ROR2,TUFT1,EIF2AK3,ZBTB40,FAM20A,ASPN,RUNX1,SLC26A2,EXT1,CCN1,CLEC5A,TWSG1,CTHRC1	15/-	BrM-specific
16_Summary	GO Biological Processes	GO:0080135	regulation of cellular response to stress	-4.145819817	-1.797625153	1201,1388,1719,2140,4189,6944,9451,10018,10445,10474,10629,51043,51548,55765,55872,79621,92797,115004,117143,641,3001,3024,9212,27154,54780,56160,121260,286053,7799	CLN3,ATF6B,DHFR,EYA3,DNAJB9,VPS72,EIF2AK3,BCL2L11,MCRS1,TADA3,TAF6L,ZBTB7B,SIRT6,INAVA,PBK,RNASEH2B,HELB,CGAS,TADA1,BLM,GZMA,H1-1,AURKB,BRPF3,NSMCE4A,NSMCE3,SLC15A4,NSMCE2,PRDM2	29/-	BrM-specific

17_Summary	GO Biological Processes	GO:0048286	lung alveolus development	-4.145813731	-1.797625153	3169,3694,4054,4321,26154,64220,2131,4311,9620,80004,1435,1999,5311,7298,10018,11035,51043,57045,84876,128239,255738,6692,768,3491,8321,9645,115908,30008	FOXA1,ITGB6,LTBP3,MMP12,ABCA12,STRA6,EXT1,MME,CELSR1,ESRP2,CSF1,ELF3,PKD2,TYMS,BCL2L11,RIPK3,ZBTB7B,TWSG1,ORAI1,IQGAP3,PCSK9,SPINT1,CA9,CCN1,FZD1,MICAL2,CTHRC1,EFE	28/-	BrM-specific
18_Summary	GO Biological Processes	GO:0030097	hemopoiesis	-3.940983027	-1.630042506	843,861,1435,2131,2874,3694,4055,4189,4297,4495,5926,5971,6482,11035,23601,27086,28982,29760,51043,54892,57045,84138,728,7462,5551,10148,23765,51206,340205	CASP10,RUNX1,CSF1,EXT1,GPS2,ITGB6,LTBR,DNAJB9,KMT2A,MT1G,ARID4A,RELB,ST3GAL1,RIPK3,CLEC5A,FOXP1,FLVCR1,BLNK,ZBTB7B,NCAPG2,TWSG1,SLC7A6OS,C5AR1,LAT2,PRF1,EBI3,IL17RA,GP6,TREML1	29/-	BrM-specific
19_Summary	GO Biological Processes	GO:0070228	regulation of lymphocyte apoptotic process	-3.886854252	-1.604685474	101,6482,9212,10018,11035,27086	ADAM8,ST3GAL1,AURKB,BCL2L11,RIPK3,FOXP1	6/-	BrM-specific
20_Summary	GO Biological Processes	GO:0032963	collagen metabolic process	-3.730435556	-1.505562181	4316,4317,4321,4327,10609,55214,101,1201,4739,9645,57104	MMP7,MMP8,MMP12,MMP19,P3H4,P3H2,ADAM8,CLN3,NEDD9,MICAL2,PNPLA2	11/-	BrM-specific
1_Summary	GO Biological Processes	GO:0050804	modulation of chemical synaptic transmission	-14.56992815	-10.67668213	1268,2899,2904,2918,5071,6547,6860,9229,9465,9699,10675,22849,22871,26012,27345,51201,54413,55638,57338,57497,57596,58512,79772,84628,84866,585,64478,152789,796,2566,2571,6323,91978	CNR1,GRIK3,GRIN2B,GRM8,PRKN,SLC8A3,SYT4,DLGAP1,AKAP7,RIMS2,CSPG5,CPEB3,NLGN1,NSMF,KCNMB4,ZDHHC2,NLGN3,SYBU,JPH3,LRFN2,BEGAIN,DLGAP3,MCTP1,NTNG2,TMEM25,BBS4,CSMD1,JAKMIP1,CALCA,GABRG2,GAD1,SCN1A,TP	33/-	BrM-NAT-specific
2_Summary	GO Biological Processes	GO:0042391	regulation of membrane potential	-14.22387726	-10.52673513	1268,2555,2562,2566,2899,2904,3739,3748,3750,3752,3781,5071,6323,6334,6547,9465,9481,9699,22871,27345,54413,84866,138429,401190,2571,6860,9229,11141,91978,2918	CNR1,GABRA2,GABRB3,GABRG2,GRIK3,GRIN2B,KCNA4,KCNC3,KCND1,KCND3,KCNN2,PRKN,SCN1A,SCN8A,SLC8A3,AKAP7,SLC25A27,RIMS2,NLGN1,KCNMB4,NLGN3,TMEM25,PIP5KL1,RGS7BP,GAD1,SYT4,DLGAP1,IL1RAPL1,TPGS1,GRM8	30/-	BrM-NAT-specific
3_Summary	GO Biological Processes	GO:0098660	inorganic ion transmembrane transport	-9.258007743	-5.685804353	1910,2555,2562,2566,2904,3739,3748,3750,3752,3763,3781,6263,6323,6334,6535,6547,7881,9481,27345,57338,79041,116843,221074,1027,26249,51310,9465,138151,200845,493829	EDNRB,GABRA2,GABRB3,GABRG2,GRIN2B,KCNA4,KCNC3,KCND1,KCND3,KCNJ6,KCNN2,RYR3,SCN1A,SCN8A,SLC6A8,SLC8A3,KCNAB1,SLC25A27,KCNMB4,JPH3,TMEM38A,SLC18B1,SLC39A12,CDKN1B,KLHL3,SLC22A17,AKAP7,NACC2,KCTD6,TR	30/-	BrM-NAT-specific
4_Summary	GO Biological Processes	GO:0050808	synapse organization	-7.294425865	-4.161555169	2555,2562,2566,6547,9806,11141,22871,51201,54413,55638,58512,84628,145581,375323,2904,9229,9699,57596,84866,1005,50855,796,1910,3752,3781,4634,27345,79041,57497,266727,1268,2899,2918,6422,22849,56099,342035,5629,6860,30812,57165	GABRA2,GABRB3,GABRG2,SLC8A3,SPOCK2,IL1RAPL1,NLGN1,ZDHHC2,NLGN3,SYBU,DLGAP3,NTNG2,LRFN5,LHFPL4,GRIN2B,DLGAP1,RIMS2,BEGAIN,TMEM25,CDH7,PARD6A,CALCA,EDNRB,KCND3,KCNN2,MYL3,KCNMB4,TMEM38A,LRFN2,MDGA1,CNR1,GRIK3,GRM8,SFRP1,CPEB3,PCDHGB7,GLDN,PROX1,SYT4,SOX8,GJC2	41/-	BrM-NAT-specific
5_Summary	GO Biological Processes	GO:0046928	regulation of neurotransmitter secretion	-5.921812983	-3.089972283	5071,6860,9699,10675,22871,27345,79772,84258,585,2263,5629,1268,6422,11141,50855,55638,6535,116843,1027,84628,157285,672,147906,1910,3752,4547,493829,644150,51201,146722,29887,54413,91978	PRKN,SYT4,RIMS2,CSPG5,NLGN1,KCNMB4,MCTP1,SYT3,BBS4,FGFR2,PROX1,CNR1,SFRP1,IL1RAPL1,PARD6A,SYBU,SLC6A8,SLC18B1,CDKN1B,NTNG2,PRAG1,BRCA1,DACT3,EDNRB,KCND3,MTTP,TRIM72,WIPF3,ZDHHC2,CD300LF,SNX10,NLGN3,TPG	33/-	BrM-NAT-specific
6_Summary	GO Biological Processes	GO:0098962	regulation of postsynaptic neurotransmitter receptor activity	-5.573293716	-2.796970344	9229,57596,58512,22871	DLGAP1,BEGAIN,DLGAP3,NLGN1	4/-	BrM-NAT-specific
7_Summary	GO Biological Processes	GO:0034765	regulation of monoatomic ion transmembrane transport	-4.687404577	-2.05720924	117,796,2904,3763,3781,7881,9465,23072,57165,57338,79041,9481,22871,1268	ADCYAP1R1,CALCA,GRIN2B,KCNJ6,KCNN2,KCNAB1,AKAP7,HECW1,GJC2,JPH3,TMEM38A,SLC25A27,NLGN1,CNR1	14/-	BrM-NAT-specific
8_Summary	GO Biological Processes	GO:0072175	epithelial tube formation	-4.627733056	-2.021671399	585,2263,5629,6422,30812,51684,221074,26249,64478,1910,200845,1027,2674,4634,22846,221981,147906,54413,6547,117,10817,672,5071,2262,121227,796,29887,1268,2875,10634,133746	BBS4,FGFR2,PROX1,SFRP1,SOX8,SUFU,SLC39A12,KLHL3,CSMD1,EDNRB,KCTD6,CDKN1B,GFRA1,MYL3,VASH1,THSD7A,DACT3,NLGN3,SLC8A3,ADCYAP1R1,FRS3,BRCA1,PRKN,GPC5,LRIG3,CALCA,SNX10,CNR1,GPT,GAS2L1,JMY	31/-	BrM-NAT-specific
9_Summary	GO Biological Processes	GO:0003012	muscle system process	-4.597454011	-2.004589518	796,1910,3752,3781,4634,6263,6323,6334,6535,493829,585,1268,27345,6547,26249,3748,5629,10634,116154,133746,221981,644150	CALCA,EDNRB,KCND3,KCNN2,MYL3,RYR3,SCN1A,SCN8A,SLC6A8,TRIM72,BBS4,CNR1,KCNMB4,SLC8A3,KLHL3,KCNC3,PROX1,GAS2L1,PHACTR3,JMY,THSD7A,WIPF3	22/-	BrM-NAT-specific
10_Summary	GO Biological Processes	GO:0051051	negative regulation of transport	-4.275471035	-1.776751417	796,1268,5071,5569,6422,6860,7881,11141,23072,51684,79772,146722,2262,2263,147906,643965,50855,51201,55638	CALCA,CNR1,PRKN,PKIA,SFRP1,SYT4,KCNAB1,IL1RAPL1,HECW1,SUFU,MCTP1,CD300LF,GPC5,FGFR2,DACT3,TMEM88B,PARD6A,ZDHHC2,SYBU	19/-	BrM-NAT-specific
11_Summary	GO Biological Processes	GO:0055080	monoatomic cation homeostasis	-3.926327309	-1.500251955	796,1268,1910,6263,6547,9481,26249,26507,29887,51310,147138,221074,5071	CALCA,CNR1,EDNRB,RYR3,SLC8A3,SLC25A27,KLHL3,CNNM1,SNX10,SLC22A17,TMC8,SLC39A12,PRKN	13/-	BrM-NAT-specific
12_Summary	GO Biological Processes	GO:0001933	negative regulation of protein phosphorylation	-3.877433216	-1.474021846	1027,5071,5569,6422,6547,9479,27124,50855,138429,57467,1910,5629,7881,9806,23072,50859,51684,147138,796,2263,85417	CDKN1B,PRKN,PKIA,SFRP1,SLC8A3,MAPK8IP1,INPP5J,PARD6A,PIP5KL1,HHATL,EDNRB,PROX1,KCNAB1,SPOCK2,HECW1,SPOCK3,SUFU,TMC8,CALCA,FGFR2,CCNB3	21/-	BrM-NAT-specific
13_Summary	GO Biological Processes	GO:0031175	neuron projection development	-3.716391621	-1.355041596	585,1268,2263,2674,10675,22871,23057,54413,81551,84628,90249,221074,222950,729920,1005,5629	BBS4,CNR1,FGFR2,GFRA1,CSPG5,NLGN1,NMNAT2,NLGN3,STMN4,NTNG2,UNC5A,SLC39A12,NYAP1,CRPPA,CDH7,PROX1	16/-	BrM-NAT-specific
14_Summary	GO Biological Processes	GO:0097553	calcium ion transmembrane import into cytosol	-3.402159737	-1.108709949	2904,6263,6547,57338,79041,576,1910,79772,3748,1268	GRIN2B,RYR3,SLC8A3,JPH3,TMEM38A,ADGRB2,EDNRB,MCTP1,KCNC3,CNR1	10/-	BrM-NAT-specific
15_Summary	GO Biological Processes	GO:0030534	adult behavior	-3.385826476	-1.09903079	585,2566,5071,6323,54413,91978,2571	BBS4,GABRG2,PRKN,SCN1A,NLGN3,TPGS1,GAD1	7/-	BrM-NAT-specific
16_Summary	GO Biological Processes	GO:0031110	regulation of microtubule polymerization or depolymerization	-3.381199569	-1.09903079	1027,10634,81551,122060,221074,585,672,5071,5629,6422,150356,51350,79745,133746,644150	CDKN1B,GAS2L1,STMN4,SLAIN1,SLC39A12,BBS4,BRCA1,PRKN,PROX1,SFRP1,CHADL,KRT76,CLIP4,JMY,WIPF3	15/-	BrM-NAT-specific
17_Summary	GO Biological Processes	GO:1904862	inhibitory synapse assembly	-3.362544204	-1.09137081	2555,2562,2566,9481	GABRA2,GABRB3,GABRG2,SLC25A27	4/-	BrM-NAT-specific
18_Summary	GO Biological Processes	GO:0035418	protein localization to synapse	-3.198610749	-0.96883004	22871,51201,81831,375323,138151,2262,5071	NLGN1,ZDHHC2,NETO2,LHFPL4,NACC2,GPC5,PRKN	7/-	BrM-NAT-specific
19_Summary	GO Biological Processes	GO:0010975	regulation of neuron projection development	-3.152142615	-0.936920626	1268,6422,11141,22871,23072,26012,27124,84628,157285,221074,585,2904	CNR1,SFRP1,IL1RAPL1,NLGN1,HECW1,NSMF,INPP5J,NTNG2,PRAG1,SLC39A12,BBS4,GRIN2B	12/-	BrM-NAT-specific
20_Summary	GO Biological Processes	GO:0071407	cellular response to organic cyclic compound	-2.993513084	-0.82461417	672,1027,2562,2566,5071,6263,6422,9465,57476,79041,147138,2875,796,55504,147906,9479,138151	BRCA1,CDKN1B,GABRB3,GABRG2,PRKN,RYR3,SFRP1,AKAP7,GRAMD1B,TMEM38A,TMC8,GPT,CALCA,TNFRSF19,DACT3,MAPK8IP1,NACC2	17/-	BrM-NAT-specific

supplementary table 3 Enriched pathways in fig3h

Category	Term	Count	%	PValue	Genes	List Total	Pop Hits	Pop Total	Fold Enrichment	Bonferroni	Benjamini	FDR	group
KEGG_PATHWAY	hsa04060:Cytokine-cytokine receptor interaction	5	13.88888889	0.003285081	CCL24, CXCL10, GDF15, EBI3, CCL18	20	298	8840	7.416107383	0.176456423	0.193819758	0.193819758	BrM_cluster1
KEGG_PATHWAY	hsa04061:Viral protein interaction with cytokine and cytokine receptor	3	8.333333333	0.019114886	CCL24, CXCL10, CCL18	20	100	8840	13.26	0.679763755	0.397416718	0.397416718	BrM_cluster1
KEGG_PATHWAY	hsa05146:Amoebiasis	3	8.333333333	0.02020763	COL1A1, C8G, COL1A2	20	103	8840	12.87378641	0.700146487	0.397416718	0.397416718	BrM_cluster1
KEGG_PATHWAY	hsa03040:Spliceosome	7	17.07317073	0.0000502	HNRNPA3, U2AF2, SRSF1, SRSF2, HNRNPU, SNRPE, SNRPD3	29	218	8840	9.788041759	0.003158559	0.003163478	0.003163478	BrM_cluster2
KEGG_PATHWAY	hsa05100:Bacterial invasion of epithelial cells	14	8.484848485	2.59E-11	ACTR3, ACTR2, ARPC1B, CLTC, CLTB, CLTA, ARPC4, ARPC5, ACTB, DNM1, CTTN, ARPC2, ARPC3, VCL	120	78	8840	13.22222222	5.33E-09	5.33E-09	4.69E-09	BrM_cluster3
KEGG_PATHWAY	hsa04530:Tight junction	18	10.90909091	9E-11	ACTR3, VASP, ACTR2, ROCK2, ARPC1B, ACTN1, PRKAG1, ARPC4, ARPC5, TUBA4A, ACTB, TUBA1C, TUBA3C, CTTN, ARPC2, ARPC3, ARHGEF2, JAM3	120	170	8840	7.8	1.85E-08	9.27E-09	8.14E-09	BrM_cluster3
KEGG_PATHWAY	hsa05130:Pathogenic Escherichia coli infection	18	10.90909091	1.46E-09	ACTR3, ACTR2, ROCK2, ARPC1B, TUBB, ARPC4, ARPC5, TUBB4B, TUBA4A, ACTB, TUBA1C, TUBA3C, TUBB2A, CTTN, ARPC2, ARPC3, TUBB1, ARHGEF2	120	203	8840	6.532019704	0.000000301	0.0000001	8.82E-08	BrM_cluster3
KEGG_PATHWAY	hsa05132:Salmonella infection	19	11.51515152	5.58E-09	ACTR3, ACTR2, ROCK2, ARPC1B, FHOD1, TUBB, ARPC4, ARPC5, TUBB4B, TUBA4A, ACTB, TUBA1C, TUBA3C, TUBB2A, ARPC2, ARPC3, TUBB1, FLNA, PFN1	120	251	8840	5.576361222	0.00000115	0.000000287	0.000000253	BrM_cluster3
KEGG_PATHWAY	hsa04810:Regulation of actin cytoskeleton	18	10.90909091	9.82E-09	ACTR3, ACTR2, ROCK2, ARPC1B, ACTN1, ITGA2, ARPC4, ARPC5, IQGAP2, ACTB, MYLK, DIAPH1, ARPC2, ARPC3, PIP5K1A, PIP5K1C, PFN1, VCL	120	230	8840	5.765217391	0.00000202	0.000000405	0.000000356	BrM_cluster3
KEGG_PATHWAY	hsa05016:Huntington disease	19	11.51515152	0.000000153	GPX1, TUBB, CLTC, CLTB, CLTA, TUBB4B, TUBA4A, PSMA5, PSMA6, TUBA1C, PSMB4, TUBA3C, TUBB2A, PSMC3, PSMA1, PSMB2, PSMA2, GNAQ, TUBB1	120	311	8840	4.500535906	0.0000316	0.00000527	0.00000463	BrM_cluster3
KEGG_PATHWAY	hsa05131:Shigellosis	17	10.3030303	0.000000196	ACTR3, ACTR2, ROCK2, ARPC1B, ACTN1, ARPC4, ARPC5, FNBP1L, ACTB, DIAPH1, CTTN, ARPC2, ARPC3, CAPN1, ARHGEF2, PFN1, VCL	120	249	8840	5.029451138	0.0000403	0.00000576	0.00000506	BrM_cluster3
KEGG_PATHWAY	hsa04814:Motor proteins	15	9.090909091	0.000000356	TPM4, TPM3, TUBB, TPM1, TUBB4B, TUBA4A, ACTB, ACTA2, TUBA1C, TUBA3C, TUBB2A, CAPZB, CAPZA1, TUBB1, CAPZA2	120	197	8840	5.609137056	0.0000734	0.00000917	0.00000806	BrM_cluster3
KEGG_PATHWAY	hsa04820:Cytoskeleton in muscle cells	16	9.696969697	0.00000045	TPM4, TPM3, TMOD3, ITGA2, TPM1, FHL1, ACTB, PDLIM1, DIAPH1, CAPZB, CAPZA1, CAPZA2, ZYX, FLNC, PDLIM7, VCL	120	232	8840	5.08045977	0.0000927	0.0000103	0.00000905	BrM_cluster3
KEGG_PATHWAY	hsa04666:Fc gamma R-mediated phagocytosis	11	6.666666667	0.000000691	ACTR3, VASP, ACTR2, ARPC2, ARPC3, ARPC1B, PIP5K1A, PRKCA, ARPC4, PIP5K1C, ARPC5	120	98	8840	8.268707483	0.000142	0.0000142	0.0000125	BrM_cluster3
KEGG_PATHWAY	hsa04144:Endocytosis	16	9.696969697	0.00000129	ACTR3, ACTR2, ARPC1B, CLTC, CLTB, CLTA, ARPC4, ARPC5, DNM1, CAPZB, ARPC2, ARPC3, CAPZA1, CAPZA2, PIP5K1A, PIP5K1C	120	252	8840	4.677248677	0.000266	0.0000242	0.0000212	BrM_cluster3
KEGG_PATHWAY	hsa05014:Amyotrophic lateral sclerosis	19	11.51515152	0.00000204	VCP, GPX1, TUBB, TUBB4B, TUBA4A, ACTB, PSMA5, PSMA6, TUBA1C, PSMB4, TUBA3C, TUBB2A, PSMC3, PSMA1, PSMB2, PSMA2, TUBB1, CAT, PFN1	120	371	8840	3.772686433	0.000421	0.0000351	0.0000308	BrM_cluster3
KEGG_PATHWAY	hsa05135:Yersinia infection	12	7.272727273	0.00000225	ACTR3, ACTR2, ARPC2, ROCK2, ARPC3, GNAQ, ARPC1B, PIP5K1A, ARPC4, PIP5K1C, ARPC5, ACTB	120	138	8840	6.405797101	0.000464	0.0000357	0.0000314	BrM_cluster3
KEGG_PATHWAY	hsa05010:Alzheimer disease	19	11.51515152	0.00000429	RTN3, APP, TUBB, TUBB4B, TUBA4A, RTN4, PSMA5, PSMA6, TUBA1C, PSMB4, TUBA3C, TUBB2A, PSMC3, PSMA1, PSMB2, PSMA2, GNAQ, TUBB1, CAPN1	120	391	8840	3.579710145	0.000884	0.0000632	0.0000555	BrM_cluster3
KEGG_PATHWAY	hsa05022:Pathways of neurodegeneration - multiple diseases	21	12.72727273	0.00000595	APP, VCP, GPX1, TUBB, PRKCA, TUBB4B, TUBA4A, PSMA5, PSMA6, TUBA1C, PSMB4, TUBA3C, TUBB2A, PSMC3, PSMA1, PSMB2, PSMA2, GNAQ, TUBB1, CAT, CAPN1	120	483	8840	3.202898551	0.001225552	0.0000818	0.0000718	BrM_cluster3
KEGG_PATHWAY	hsa04611:Platelet activation	11	6.666666667	0.00000645	VASP, GP9, ROCK2, GP1BB, GNAQ, ITGA2, GP1BA, RASGRP2, GP5, ACTB, MYLK	120	125	8840	6.482666667	0.001327792	0.000083	0.000073	BrM_cluster3
KEGG_PATHWAY	hsa04145:Phagosome	12	7.272727273	0.00000791	TUBA1C, TUBB2A, TUBA3C, ITGA2, TUBB, TUBB1, CANX, CALR, TUBB4B, CORO1A, ACTB, TUBA4A	120	157	8840	5.630573248	0.001627503	0.0000958	0.0000842	BrM_cluster3
KEGG_PATHWAY	hsa04510:Focal adhesion	13	7.878787879	0.0000171	VASP, ROCK2, ACTN1, ITGA2, PRKCA, ACTB, MYLK, DIAPH1, ZYX, PIP5K1A, FLNA, PIP5K1C, VCL	120	203	8840	4.717569787	0.003517044	0.000196	0.000172	BrM_cluster3
KEGG_PATHWAY	hsa04540:Gap junction	9	5.454545455	0.0000309	TUBA1C, TUBB2A, TUBA3C, GNAQ, TUBB, TUBB1, PRKCA, TUBB4B, TUBA4A	120	92	8840	7.206521739	0.006339781	0.000326	0.000286	BrM_cluster3
KEGG_PATHWAY	hsa03050:Proteasome	7	4.242424242	0.0000316	PSMA5, PSMA6, PSMB4, PSMA1, PSMC3, PSMB2, PSMA2	120	46	8840	11.21014493	0.006497647	0.000326	0.000286	BrM_cluster3
KEGG_PATHWAY	hsa05012:Parkinson disease	14	8.484848485	0.0000678	TUBB, TUBB4B, TUBA4A, PSMA5, PSMA6, TUBA1C, PSMB4, TUBA3C, TUBB2A, PSMC3, PSMA1, PSMB2, PSMA2, TUBB1	120	271	8840	3.805658057	0.013877122	0.000665	0.000585	BrM_cluster3
KEGG_PATHWAY	hsa05020:Prion disease	14	8.484848485	0.000088	TUBB, TUBB4B, TUBA4A, PSMA5, PSMA6, TUBA1C, PSMB4, TUBA3C, TUBB2A, PSMC3, PSMA1, PSMB2, PSMA2, TUBB1	120	278	8840	3.709832134	0.017957424	0.000824	0.000724	BrM_cluster3
KEGG_PATHWAY	hsa04961:Endocrine and other factor-regulated calcium reabsorption	6	3.636363636	0.000696	GNAQ, CLTC, CLTB, CLTA, PRKCA, DNM1	120	53	8840	8.339622642	0.13363394	0.005994145	0.0052667	BrM_cluster3
KEGG_PATHWAY	hsa05017:Spinocerebellar ataxia	9	5.454545455	0.000698	PSMA5, PSMA6, PSMB4, PSMA1, PSMC3, PSMB2, PSMA2, GNAQ, PRKCA	120	144	8840	4.604166667	0.134034088	0.005994145	0.0052667	BrM_cluster3
KEGG_PATHWAY	hsa04670:Leukocyte transendothelial migration	7	4.242424242	0.004675442	VASP, ROCK2, ACTN1, PRKCA, VCL, ACTB, JAM3	120	116	8840	4.445402299	0.619169051	0.038525644	0.033850202	BrM_cluster3
KEGG_PATHWAY	hsa04141:Protein processing in endoplasmic reticulum	8	4.848484848	0.00793953	VCP, UBXN1, SAR1A, CANX, ERP29, CAPN1, CALR, PDIA6	120	170	8840	3.466666667	0.806421186	0.062905509	0.055271346	BrM_cluster3
KEGG_PATHWAY	hsa05410:Hypertrophic cardiomyopathy	6	3.636363636	0.010642844	TPM4, TPM3, ITGA2, TPM1, PRKAG1, ACTB	120	99	8840	4.464646465	0.889658595	0.081200955	0.07134647	BrM_cluster3
KEGG_PATHWAY	hsa04066:HIF-1 signaling pathway	6	3.636363636	0.015645875	LDHB, STAT3, PRKCA, PGK2, ALDOA, PFKP	120	109	8840	4.055045872	0.961168753	0.115108935	0.101139404	BrM_cluster3
KEGG_PATHWAY	hsa04918:Thyroid hormone synthesis	5	3.03030303	0.018230065	GPX1, GNAQ, CANX, GSR, PRKCA	120	75	8840	4.911111111	0.977405429	0.129496321	0.113780748	BrM_cluster3
KEGG_PATHWAY	hsa01200:Carbon metabolism	6	3.636363636	0.019956409	MDH2, CAT, PGK2, ALDOA, PGD, PFKP	120	116	8840	3.810344828	0.984276438	0.133226509	0.117058243	BrM_cluster3
KEGG_PATHWAY	hsa05205:Proteoglycans in cancer	8	4.848484848	0.020048649	CD63, CTTN, ROCK2, ITGA2, STAT3, FLNA, PRKCA, ACTB	120	204	8840	2.888888889	0.984578371	0.133226509	0.117058243	BrM_cluster3
KEGG_PATHWAY	hsa04512:ECM-receptor interaction	5	3.03030303	0.031771212	GP9, GP1BB, ITGA2, GP1BA, GP5	120	89	8840	4.138576779	0.998707394	0.204527175	0.179705916	BrM_cluster3
KEGG_PATHWAY	hsa04270:Vascular smooth muscle contraction	6	3.636363636	0.034408269	ACTA2, PPP1R14A, ROCK2, GNAQ, PRKCA, MYLK	120	134	8840	3.298507463	0.999262998	0.21479101	0.18872414	BrM_cluster3
KEGG_PATHWAY	hsa04210:Apoptosis	6	3.636363636	0.036329719	TUBA1C, TUBA3C, CAPN1, CTSD, ACTB, TUBA4A	120	136	8840	3.25	0.999511051	0.220115353	0.193402325	BrM_cluster3
KEGG_PATHWAY	hsa00480:Glutathione metabolism	4	2.424242424	0.04298246	GPX1, GSTO1, GSR, PGD	120	58	8840	5.08045977	0.999882646	0.252982481	0.222280723	BrM_cluster3
KEGG_PATHWAY	hsa04640:Hematopoietic cell lineage	5	3.03030303	0.044281742	GP9, GP1BB, ITGA2, GP1BA, GP5	120	99	8840	3.720538721	0.999911293	0.253389971	0.222638761	BrM_cluster3
KEGG_PATHWAY	hsa05146:Amoebiasis	5	3.03030303	0.049958461	ACTN1, GNAQ, HSPB1, PRKCA, VCL	120	103	8840	3.57605178	0.999974	0.278147109	0.244391391	BrM_cluster3

supplementary table 3 Enriched pathways in fig3i														
Category	Term	Count	%	PValue	Genes	List Total	Pop Hits	Pop Total	Fold Enrichment	Bonferroni	Benjamini	FDR	group
KEGG_PATHWAY	hsa04610:Complement and coagulation cascades	21	11.35135135	2.66E-22	C1QB, C1QA, CPB2, F10, SERPINC1, F12, F11, PLAT, C4BPA, C4BPB, TFPI, C8A, KNG1, SERPINA5, F7, F9, PROC, C6, CFHR5, KLKB1, C1QC	91	88	8840	23.18181818	3.49E-20	3.49E-20	3.33E-20	NSCLC_cluster1
KEGG_PATHWAY	hsa05150:Staphylococcus aureus infection	9	4.864864865	0.0000073	C1QB, C1QA, KRT17, KRT28, KRT16, KRT14, KRT10, KRT9, C1QC	91	100	8840	8.742857143	0.000956	0.000478	0.000456	NSCLC_cluster1
KEGG_PATHWAY	hsa04979:Cholesterol metabolism	6	3.243243243	0.000159	LIPC, APOC2, APOC1, ANGPTL3, APOA1, APOE	91	51	8840	11.42857143	0.020594533	0.006935968	0.00661829	NSCLC_cluster1
KEGG_PATHWAY	hsa04936:Alcoholic liver disease	8	4.324324324	0.000636	FABP1, C1QB, C1QA, ADH4, ADH1C, ADH1B, ADH1A, C1QC	91	144	8840	5.396825397	0.080007665	0.017705601	0.016894658	NSCLC_cluster1
KEGG_PATHWAY	hsa04974:Protein digestion and absorption	7	3.783783784	0.000676	COL18A1, CPB2, PRSS1, COL14A1, FXYD2, COL5A3, COL12A1	91	105	8840	6.476190476	0.084749913	0.017705601	0.016894658	NSCLC_cluster1
KEGG_PATHWAY	hsa00350:Tyrosine metabolism	4	2.162162162	0.005716101	ADH4, ADH1C, ADH1B, ADH1A	91	36	8840	10.79365079	0.528085525	0.12480153	0.11908543	NSCLC_cluster1
KEGG_PATHWAY	hsa05133:Pertussis	5	2.702702703	0.008080795	C1QB, C1QA, C4BPA, C4BPB, C1QC	91	78	8840	6.227106227	0.654542079	0.151226307	0.144299912	NSCLC_cluster1
KEGG_PATHWAY	hsa00071:Fatty acid degradation	4	2.162162162	0.009387862	ADH4, ADH1C, ADH1B, ADH1A	91	43	8840	9.03654485	0.709345179	0.153726234	0.146685338	NSCLC_cluster1
KEGG_PATHWAY	hsa00620:Pyruvate metabolism	4	2.162162162	0.011980848	ADH4, ADH1C, ADH1B, ADH1A	91	47	8840	8.267477204	0.793812399	0.174387905	0.166400673	NSCLC_cluster1
KEGG_PATHWAY	hsa04915:Estrogen signaling pathway	6	3.243243243	0.01352373	KRT17, KRT28, KRT16, KRT14, KRT10, KRT9	91	139	8840	4.193216855	0.831984182	0.177160866	0.169046628	NSCLC_cluster1
KEGG_PATHWAY	hsa00010:Glycolysis / Gluconeogenesis	4	2.162162162	0.030625017	ADH4, ADH1C, ADH1B, ADH1A	91	67	8840	5.799573561	0.983000867	0.347243358	0.331339082	NSCLC_cluster1
KEGG_PATHWAY	hsa00830:Retinol metabolism	4	2.162162162	0.031808552	ADH4, ADH1C, ADH1B, ADH1A	91	68	8840	5.714285714	0.985514859	0.347243358	0.331339082	NSCLC_cluster1
KEGG_PATHWAY	hsa00982:Drug metabolism - cytochrome P450	4	2.162162162	0.038081401	ADH4, ADH1C, ADH1B, ADH1A	91	73	8840	5.322896282	0.993818125	0.383743349	0.366167318	NSCLC_cluster1
KEGG_PATHWAY	hsa00980:Metabolism of xenobiotics by cytochrome P450	4	2.162162162	0.046379081	ADH4, ADH1C, ADH1B, ADH1A	91	79	8840	4.918625678	0.998012872	0.433975687	0.414098938	NSCLC_cluster1
KEGG_PATHWAY	hsa05322:Systemic lupus erythematosus	5	2.702702703	0.053079921	C1QB, C1QA, C6, C8A, C1QC	91	139	8840	3.494347379	0.999211057	0.444109107	0.423768231	NSCLC_cluster1
KEGG_PATHWAY	hsa05143:African trypanosomiasis	3	1.621621622	0.054242334	APOA1, APOL1, KNG1	91	37	8840	7.876447876	0.999328319	0.444109107	0.423768231	NSCLC_cluster1
KEGG_PATHWAY	hsa04512:ECM-receptor interaction	4	2.162162162	0.062012311	TNXB, CHAD, MEPE, HSPG2	91	89	8840	4.365971108	0.999772058	0.477859574	0.455972876	NSCLC_cluster1
KEGG_PATHWAY	hsa04975:Fat digestion and absorption	3	1.621621622	0.070746351	FABP1, MTTP, APOA1	91	43	8840	6.777408638	0.999933075	0.514876223	0.491294106	NSCLC_cluster1
KEGG_PATHWAY	hsa05142:Chagas disease	4	2.162162162	0.087434056	C1QB, C1QA, KNG1, C1QC	91	103	8840	3.772538141	0.999993768	0.602834808	0.575224053	NSCLC_cluster1
KEGG_PATHWAY	hsa04144:Endocytosis	87	8.1232493	1.28E-33	ARF3, WASHC5, ARF4, WIPF1, ARPC1B, CLTC, CLTB, CLTA, ARRB1, AP2A1, CAPZB, CHMP1B, KIF5B, CHMP1A, VPS35, VPS36, AP2M1, GIT1, RAB8A, SH3GL1, ACTR3, USP8, ACTR2, PDCD6IP, IST1, HLA-B, HLA-C, HLA-A, ARAP1, RHOA, DNM1, DNM2, DNM3, RUFY1, ACAP1, PARD3, RAB35, CHMP3, CHMP4B, CHMP4A, CHMP6, VPS25, ARF5, RAB7A, ARF6, RAB5B, TSG101, RAB5C, SRC, VPS4B, VPS4A, AGAP2, ASAP1, ASAP2, SNX3, CDC42, CYTH2, SNX1, GRK2, SNX2, GRK5, GRK6, AP2S1, PIP5K1A, PIP5K1C, SNX5, SNX6, HSPA8, VTA1, ARPC4, AP2B1, ARPC5, RAB11A, PML, RAB10, EHD1, EHD3, EHD4, ARPC2, ARPC3, CAPZA1, CAPZA2, CHMP2B, CHMP2A, VPS45, RAB5A, HSPA1A	708	252	8840	4.310599946	4.12E-31	4.12E-31	2.8E-31	NSCLC_cluster2
KEGG_PATHWAY	hsa04611:Platelet activation	48	4.481792717	9.26E-21	ITGB1, ROCK1, ROCK2, SRC, ITGB3, SNAP23, ITGA2B, GNAI3, PIK3R1, RASGRP2, ACTB, GNAI2, MYLK, MYL12B, RAP1B, GNA13, PPP1CB, RAP1A, PLCG2, PRKACA, PRKACB, LYN, VASP, ARHGEF12, FCER1G, PPP1R12A, SYK, GP1BB, ITGA2, GP1BA, GP5, RHOA, PPP1CA, VAMP8, GP9, PLCB3, PLCB4, FCGR2A, GNAQ, P2RX1, GNAS, BTK, LCP2, ARHGEF1, TLN1, PLCB2, F2RL3, FERMT3	708	125	8840	4.794576271	2.97E-18	1.49E-18	1.01E-18	NSCLC_cluster2
KEGG_PATHWAY	hsa04810:Regulation of actin cytoskeleton	66	6.162464986	1.65E-20	ITGB1, CYFIP1, NCKAP1, ITGB3, ARPC1B, ITGA2B, BRK1, ACTB, MYLK, PPP1CB, TMSB4X, CFL1, RAC2, PIP4K2A, PIP4K2B, PIP4K2C, RAC1, GIT1, VAV3, ACTR3, ACTR2, PPP1R12A, ARHGEF12, ACTN1, ITGA2, ACTN4, RHOA, VAV1, MYH9, ARHGEF1, ITGA6, ITGA5, ARHGEF7, PFN1, MYL9, VCL, ROCK1, ROCK2, SRC, PIK3R1, IQGAP1, IQGAP2, MYL12B, GNA13, CDC42, NRAS, PAK1, RRAS, PIP5K1A, MYH11, NCKAP1L, PIP5K1C, MYH10, WASF2, PAK2, GSN, RDX, MSN, ARPC4, ARPC5, PTK2, PPP1CA, DIAPH1, ARPC2, ARPC3, KRAS	708	230	8840	3.582903464	5.3E-18	1.77E-18	1.2E-18	NSCLC_cluster2
KEGG_PATHWAY	hsa04530:Tight junction	54	5.042016807	5.38E-19	ITGB1, ARPC1B, PRKAG1, MYL6B, F11R, YBX3, ACTB, TUBA1C, PPP2R1A, RAC1, PRKACA, PRKACB, RAB8A, RAB8B, MAP3K5, ACTR3, ACTR2, ACTN1, ACTN4, RHOA, TUBA4A, CLDN5, MYL6, CLDN3, CTTN, PARD3, MYH9, RAPGEF2, HCLS1, ARHGEF2, MYL9, ROCK1, ROCK2, SRC, WAS, MYL12B, PPP2CA, CDC42, RAP1A, TUBA3C, MYH11, MYH10, JAM3, VASP, NHERF1, HSPA4, RDX, MSN, ARPC4, ARPC5, ARPC2,	708	170	8840	3.966101695	1.73E-16	4.32E-17	2.93E-17	NSCLC_cluster2
KEGG_PATHWAY	hsa05100:Bacterial invasion of epithelial cells	36	3.361344538	1.08E-18	ITGB1, SRC, ARPC1B, CLTC, CLTB, ILK, CLTA, PIK3R1, SEPTIN11, ACTB, CDC42, RAC1, WASF2, ACTR3, ACTR2, SEPTIN2, RHOG, SEPTIN6, ARPC4, ARPC5, SEPTIN9, RHOA, DNM1, PTK2, CD2AP, DNM2, DNM3, ARHGAP10, CTTN, ARPC2, ARPC3, ELMO1, ELMO2, HCLS1, ITGA5, VCL	708	78	8840	5.762711864	3.47E-16	6.94E-17	4.71E-17	NSCLC_cluster2
KEGG_PATHWAY	hsa05132:Salmonella infection	65	6.069094304	1.03E-17	CYFIP1, NCKAP1, HSP90AB1, CSE1L, FHOD1, ARPC1B, BRK1, ACTB, TUBA1C, KIF5B, TUBB1, RAC1, SKP1, MAP2K3, ACTR3, ACTR2, HSP90AA1, EXOC7, TUBB, RHOG, DYNLL1, RHOA, TUBA4A, DNM2, TUBB2A, ELMO1, EXOC4, ELMO2, EXOC5, PFN1, MYL9, GAPDH, ARL8A, EXOC2, RAB7A, ARF6, DYNC1I2, RAB5B, RAB5C, ROCK2, DCTN1, TXN, KLC1, MYL12B, CDC42, CYTH2, PAK1, KLC2, TUBA3C, RRAS, RPS3, FLNA, NCKAP1L, VPS16, DYNC1H1, MLKL, ARPC4, ARPC5, TUBB4B, SNX18, PTPRC, ARPC2, ARPC3, ABI1, RAB5A	708	251	8840	3.233394107	3.3E-15	5.51E-16	3.74E-16	NSCLC_cluster2
KEGG_PATHWAY	hsa05130:Pathogenic Escherichia coli infection	53	4.948646125	1.39E-14	ITGB1, CYFIP1, NCKAP1, WIPF1, ARPC1B, BRK1, ACTB, TUBA1C, TUBB1, NCK2, RAC1, ACTR3, ACTR2, ARHGEF12, TUBB, RHOA, TUBA4A, CLDN5, CLDN3, TUBB2A, CTTN, MYH9, HCLS1, ARHGEF1, ARHGEF2, GAPDH, ARF6, RAB1A, ROCK1, ROCK2, SRC, GNA13, CDC42, CYTH2, PAK1, TUBA3C, RPS3, MYH11, NCKAP1L, MYH10, WASF2, PAK2, NHERF1, MYO5A, ARPC4, ARPC5, TUBB4B, MYO1C, FCGR2A, ARPC2, ARPC3, ABI1, PTPN6	708	203	8840	3.259859175	4.45E-12	6.39E-13	4.34E-13	NSCLC_cluster2

KEGG_PATHWAY	hsa04666:Fc gamma R-mediated phagocytosis	35	3.267973856	4.28E-14	ARPC1B, WAS, ASAP1, ASAP2, PIK3R1, CDC42, PAK1, INPP5D, CFL1, PLCG2, RAC2, PIP5K1A, PIP5K1C, RAC1, WASF2, VAV3, LYN, VASP, ACTR3, ACTR2, GSN, SYK, PRKCB, PRKCD, PRKCA, ARPC4, ARPC5, VAV1, DNM2, HCK, PTPRC, FCGR2A, ARPC2, ARPC3, ARF6	708	98	8840	4.459241324	1.38E-11	1.72E-12	1.17E-12	NSCLC_cluster2
KEGG_PATHWAY	hsa03050:Proteasome	24	2.240896359	5.86E-14	PSMD11, PSMD13, PSMA7, PSMA5, PSMD9, PSMA6, PSMA3, PSMB4, PSMC6, PSMD7, PSMA4, PSMC3, PSMD4, PSMA1, PSMB2, PSMA2, PSMC1, PSMD2, PSMC2, PSMD3, PSMB1, PSME1, PSMD1, PSME2	708	46	8840	6.514369934	1.88E-11	2.09E-12	1.42E-12	NSCLC_cluster2
KEGG_PATHWAY	hsa05135:Yersinia infection	40	3.734827264	1.08E-12	ITGB1, ROCK1, ROCK2, WIPF1, SRC, ARPC1B, WAS, PIK3R1, ACTB, CDC42, RPS6KA3, RAC2, PIP5K1A, PIP5K1C, RAC1, WASF2, VAV3, MAP2K3, ACTR3, ACTR2, ARHGEF12, FYB1, RHOG, ARPC4, ARPC5, RHOA, VAV1, PTK2, FCGR2A, ARPC2, ARPC3, GNAQ, ELMO1, ELMO2, LCP2, ARHGEF1, ITGA5, ARHGEF7, SKAP2, ARF6	708	138	8840	3.619094408	3.47E-10	3.47E-11	2.36E-11	NSCLC_cluster2
KEGG_PATHWAY	hsa04062:Chemokine signaling pathway	47	4.388422035	7.8E-12	ROCK1, ROCK2, SRC, GNAI3, WAS, ARRB1, PIK3R1, RASGRP2, GNAI2, RAP1B, CDC42, PAK1, NRAS, GNG10, RAP1A, GRK2, GNG5, GRK5, GRK6, PLCG2, RAC2, RAC1, PRKACA, PRKACB, PF4V1, VAV3, LYN, PRKCB, PRKCD, STAT3, PPBP, RHOA, VAV1, PTK2, HCK, PLCB3, PLCB4, GNAQ, GNB2, PARD3, GNB1, ELMO1, GRB2, KRAS, DOCK2, PLCB2, PF4	708	193	8840	3.040601856	2.5E-09	2.28E-10	1.55E-10	NSCLC_cluster2
KEGG_PATHWAY	hsa05131:Shigellosis	53	4.948646125	6.94E-11	ITGB1, ARPC1B, UBE2D3, ILK, FNBP1L, ACTB, SEPTIN11, CAPNS1, CAPN1, RAC1, SKP1, CAST, ACTR3, ACTR2, ACTN1, PRKCD, ACTN4, RHOA, PLCB3, PLCB4, CTTN, ELMO1, ELMO2, HCLS1, UBE2V1, ARHGEF2, ITGA5, TLN1, PFN1, MYL9, PLCB2, VCL, ARF6, ROCK1, ROCK2, SRC, PIK3R1, MYL12B, CDC42, CYTH2, PLCG2, WASF2, SEPTIN2, SEPTIN6, ARPC4, ARPC5, SEPTIN9, SEPTIN7, PTK2, DIAPH1,	708	249	8840	2.657636194	2.23E-08	1.86E-09	1.26E-09	NSCLC_cluster2
KEGG_PATHWAY	hsa04961:Endocrine and other factor-regulated calcium reabsorption	22	2.054154995	1.88E-10	PRKCB, CLTC, CLTB, CLTA, AP2A1, ATP1B3, PRKCA, AP2B1, ATP1A1, RAB11A, DNM1, DNM2, DNM3, PLCB3, PLCB4, GNAQ, AP2S1, GNAS, PRKACA, PLCB2, PRKACB, AP2M1	708	53	8840	5.182816331	6.03E-08	4.64E-09	3.15E-09	NSCLC_cluster2
KEGG_PATHWAY	hsa04145:Phagosome	39	3.641456583	3.34E-10	ATP6V1A, ITGB1, DYNC1I2, STX12, RAB5B, RAB5C, ITGB3, CORO1A, THBS1, ACTB, TUBA1C, TUBA3C, LAMP1, TUBB1, LAMP2, ATP6V1H, CD36, RAC1, ATP6V1E1, ATP6V1D, ATP6V1C1, DYNC1H1, ATP6V1G1, ITGA2, TUBB, HLA-B, HLA-C, HLA-A, TUBB4B, TUBA4A, FCGR2A, TUBB2A, CANX, ATP6V1B2, ITGA5, CALR, RAB5A, RAB7A, VAMP3	708	157	8840	3.101586959	0.000000107	7.66E-09	5.2E-09	NSCLC_cluster2
KEGG_PATHWAY	hsa04510:Focal adhesion	45	4.201680672	6.15E-10	ITGB1, ROCK1, ROCK2, SRC, ITGB3, ITGA2B, ILK, PIK3R1, THBS1, ACTB, MYLK, MYL12B, RAP1B, CDC42, PPP1CB, PAK1, RAP1A, RAC2, PIP5K1A, FLNA, PIP5K1C, RAC1, PAK2, VAV3, VASP, PPP1R12A, PRKCB, PDPK1, ACTN1, ITGA2, PRKCA, ACTN4, PARVB, RHOA, VAV1, PTK2, PPP1CA, DIAPH1, ZYX, GRB2, ITGA6, ITGA5, TLN1, MYL9, VCL	708	203	8840	2.76780496	0.000000197	1.32E-08	8.94E-09	NSCLC_cluster2
KEGG_PATHWAY	hsa04670:Leukocyte transendothelial migration	32	2.987861811	1.09E-09	ITGB1, ROCK1, ROCK2, GNAI3, PIK3R1, F11R, ACTB, GNAI2, MYL12B, RAP1B, CDC42, RAP1A, PLCG2, RAC2, RAC1, JAM3, VAV3, VASP, PRKCB, ACTN1, MSN, PRKCA, ACTN4, RHOA, VAV1, PTK2, CLDN5, CLDN3, PECAM1, ESAM, MYL9, VCL	708	116	8840	3.444379505	0.000000349	2.18E-08	1.48E-08	NSCLC_cluster2
KEGG_PATHWAY	hsa05205:Proteoglycans in cancer	44	4.108309991	2.41E-09	ITGB1, CD63, ROCK1, ROCK2, SRC, ITGB3, PIK3R1, IQGAP1, THBS1, ACTB, CDC42, PPP1CB, PAK1, NRAS, RRAS, PLCG2, FLNA, RAC1, PRKACA, PRKACB, EIF4B, VAV3, TGFB1, ARHGEF12, PPP1R12A, PRKCB, PDPK1, ITGA2, RDX, STAT3, MSN, PRKCA, RHOA, VAV1, PTK2, PPP1CA, CTTN, HCLS1, GRB2, KRAS, PTPN6, ARHGEF1, ITGA5, HPSE	708	204	8840	2.693032015	0.000000774	4.55E-08	3.09E-08	NSCLC_cluster2
KEGG_PATHWAY	hsa01200:Carbon metabolism	31	2.89449113	4.72E-09	GPI, PRPS1, ECHS1, ENO1, ENO2, ADH5, TKFC, ESD, PGK1, PGK2, G6PD, TPI1, PKLR, MDH1, MDH2, IDH1, PGAM1, IDH2, TALDO1, PGD, CS, PFKL, PKM, CAT, PGP, ALDOC, ALDOA, TKT, PFKM, GAPDH, PFKP	708	116	8840	3.336742646	0.00000152	8.42E-08	5.72E-08	NSCLC_cluster2
KEGG_PATHWAY	hsa05012:Parkinson disease	50	4.66853408	3.84E-08	PARK7, UBE2L3, TUBA1C, PSMD9, PSMD7, PSMD4, PSMD2, KIF5B, TUBB1, PSMD3, PSMD1, PRKACA, PRKACB, MAP3K5, TUBB, SLC11A2, TUBA4A, PSMA5, PSMA6, PSMA3, PSMA4, TUBB2A, PSMA1, PSMA2, PSMD11, PSMD13, GNAI3, TXN, KLC1, PSMA7, GNAI2, PSMB4, ATP5F1A, KLC2, TUBA3C, ATP5F1B, PSMB2, PSMB1, SNCA, SEPTIN5, TUBB4B, EIF2S1, SOD1, PSMC6, PSMC3, PSMC1, PSMC2, GNAS, UBA1, CALM1	708	271	8840	2.303667104	0.0000123	0.000000648	0.00000044	NSCLC_cluster2
KEGG_PATHWAY	hsa05017:Spinocerebellar ataxia	32	2.987861811	0.000000258	PSMD11, PSMD13, ATP2A3, ATP2A2, PIK3R1, PSMA7, PSMD9, PSMB4, PSMD7, PSMD4, PSMB2, PSMD2, PSMD3, PSMB1, PSMD1, MAP3K5, PRKCB, PRKCA, PSMA5, PSMA6, PLCB3, PSMA3, PSMC6, PLCB4, PSMA4, PSMC3, PSMA1, PSMA2, PSMC1, GNAQ, PSMC2, PLCB2	708	144	8840	2.774639046	0.0000828	0.00000414	0.00000281	NSCLC_cluster2
KEGG_PATHWAY	hsa04820:Cytoskeleton in muscle cells	43	4.014939309	0.000000355	ITGB1, ITGB3, ITGA2B, FHL1, ATP1A1, ENO1, NID1, ENO2, NID2, THBS1, ACTB, PDLIM1, CAPZB, CSRP1, MYH11, FLNC, PDLIM5, MYH10, LBR, PDLIM7, SPTBN1, TPM4, TPM3, TPM2, TMOD3, ITGA2, TPM1, AMPD2, ATP1B3, DIAPH1, INF2, DAAM1, CAPZA1, CAPZA2, ZYX, MYH9, ITGA6, VIM, ITGA5, TLN1, MYL9, VCL, PLEC	708	232	8840	2.31419248	0.000114	0.00000543	0.00000369	NSCLC_cluster2
KEGG_PATHWAY	hsa04270:Vascular smooth muscle contraction	30	2.801120448	0.000000559	ROCK1, ROCK2, MYL6B, MYLK, GNA13, PPP1CB, CALD1, MYH11, PRKACA, MYH10, PRKACB, PPP1R14A, ARHGEF12, PPP1R12A, PRKCB, PRKCD, PRKCA, RHOA, PPP1CA, ACTA2, PLCB3, MYL6, PLCB4, GNAQ, GNAS, MYH9, ARHGEF1, CALM1, PLCB2, MYL9	708	134	8840	2.795345307	0.000179	0.00000815	0.00000554	NSCLC_cluster2
KEGG_PATHWAY	hsa00010:Glycolysis / Gluconeogenesis	20	1.867413632	0.000000694	GPI, TPI1, PKLR, PGAM1, ENO1, ENO2, ADH5, LDHB, LDHA, PFKL, PKM, PGK1, ALDOC, PGK2, ALDOA, GAPDH, PFKM, PGM1, PFKP, ALDH9A1	708	67	8840	3.727127076	0.000223	0.00000951	0.00000646	NSCLC_cluster2
KEGG_PATHWAY	hsa04520:Adherens junction	24	2.240896359	0.000000711	YES1, ROCK1, ROCK2, SRC, ACTN1, WAS, PTPRJ, ACTN4, IQGAP1, RHOA, ACTB, MYL12B, CDC42, RAP1B, RAP1A, PDCD10, PARD3, RAC2, PTPN6, RAC1, WASF2, ACP1, MYL9, VCL	708	93	8840	3.222161473	0.000228	0.00000951	0.00000646	NSCLC_cluster2

KEGG_PATHWAY	hsa01230:Biosynthesis of amino acids	21	1.960784314	0.00000105	PRPS1, TPI1, PKLR, PGAM1, IDH1, IDH2, TALDO1, ENO1, ENO2, CS, PFKL, PKM, PGK1, ALDOC, ASL, PGK2, ALDOA, TKT, GAPDH, PFKM,	708	75	8840	3.496045198	0.000336	0.0000134	0.00000913	NSCLC_cluster2
KEGG_PATHWAY	hsa04141:Protein processing in endoplasmic reticulum	34	3.174603175	0.00000131	VCP, UBXN1, HSP90AB1, SAR1A, SAR1B, UBE2D3, RRBP1, DNAJB2, DNAJB1, LMAN1, GANAB, LMAN2, UFD1, CAPN1, UGGT1, TXNDC5, SKP1, MAP3K5, PDIA3, HSPA8, HSP90AA1, YOD1, PDIA6, SVIP, EIF2S1, DNAJA1, NSFL1C, CANX, DNAJA2, ERP29, HYOU1, P4HB, CALR, HSPA1A	708	170	8840	2.497175141	0.00042	0.0000162	0.000011	NSCLC_cluster2
KEGG_PATHWAY	hsa04015:Rap1 signaling pathway	39	3.641456583	0.00000166	ITGB1, RALB, SRC, ITGB3, ITGA2B, GNAI3, PIK3R1, RASGRP2, THBS1, ACTB, GNAI2, RAP1B, CDC42, NRAS, RAP1A, RRAS, RAC2, RAC1, VAV3, MAP2K3, VASP, FYB1, PRKCB, PRKCA, RHOA, VAV1, PLCB3, PLCB4, GNAQ, PARD3, GNAS, RAPGEF2, KRAS, LCP2, PFN1, CALM1, TLN1, PLCB2, F2RL3	708	212	8840	2.296929965	0.000533	0.0000198	0.0000134	NSCLC_cluster2
KEGG_PATHWAY	hsa00030:Pentose phosphate pathway	13	1.213818861	0.00000213	GPI, PRPS1, G6PD, TALDO1, PGD, DERA, PFKL, ALDOC, ALDOA, TKT, PFKM, PGM1, PFKP	708	31	8840	5.236012393	0.000682	0.0000244	0.0000165	NSCLC_cluster2
KEGG_PATHWAY	hsa05016:Huntington disease	50	4.66853408	0.00000276	CLTC, CLTB, CLTA, AP2A1, TUBA1C, PSMD9, PSMD7, PSMD4, PSMD2, KIF5B, TUBB1, PSMD3, PSMD1, AP2M1, MAP3K5, GPX1, TUBB, TUBA4A, PSMA5, PSMA6, PSMA3, PLCB3, PSMA4, PLCB4, TUBB2A, PSMA1, PSMA2, PLCB2, PSMD11, PSMD13, DCTN1, KLC1, PSMA7, PSMB4, ATP5F1A, KLC2, TUBA3C, ATP5F1B, PSMB2, PSMB1, AP2S1, BDNF, AP2B1, TUBB4B, SOD1, PSMC6, PSMC3, PSMC1, GNAO, PSMC2	708	311	8840	2.007375515	0.000886	0.0000306	0.0000208	NSCLC_cluster2
KEGG_PATHWAY	hsa04071:Sphingolipid signaling pathway	27	2.521008403	0.00000291	ASAH1, ROCK1, ROCK2, GNAI3, PIK3R1, GNAI2, PPP2CA, GNA13, NRAS, PPP2R1A, PPP2R5E, RAC2, RAC1, CTSD, MAP3K5, FCER1G, PRKCB, PDPK1, PPP2R5A, PRKCA, PPP2R5D, RHOA, PLCB3, PLCB4, GNAQ, KRAS, PLCB2	708	122	8840	2.763267574	0.000934	0.0000312	0.0000212	NSCLC_cluster2
KEGG_PATHWAY	hsa04962:Vasopressin-regulated water reabsorption	15	1.400560224	0.0000046	NSF, DYNC1H1, DYNC1I2, RAB5B, RAB5C, DCTN1, DYNLL1, RAB11A, ARHGDIA, ARHGDIB, GNAS, STX4, PRKACA, RAB5A, PRKACB	708	44	8840	4.256548536	0.001475912	0.0000476	0.0000324	NSCLC_cluster2
KEGG_PATHWAY	hsa05020:Prion disease	45	4.201680672	0.00000801	PSMD11, PSMD13, PIK3R1, KLC1, PSMA7, PSMD9, TUBA1C, PPP3R1, ATP5F1A, PSMB4, KLC2, PSMD7, TUBA3C, ATP5F1B, PSMD4, PSMB2, PSMD2, KIF5B, TUBB1, PSMD3, PSMB1, RAC2, PSMD1, RAC1, PRKACA, PRKACB, HSPA8, TUBB, PRKCD, TUBB4B, EIF2S1, TUBA4A, SOD1, PSMA5, PSMA6, PSMA3, PSMC6, TUBB2A, PSMA4, PSMC3, PSMA1, PSMA2, PSMC1, PSMC2, HSPA1A	708	278	8840	2.021094988	0.002566481	0.0000784	0.0000533	NSCLC_cluster2
KEGG_PATHWAY	hsa05163:Human cytomegalovirus infection	39	3.641456583	0.00000806	ROCK1, ROCK2, SRC, ITGB3, GNAI3, PIK3R1, GNAI2, GNA13, NRAS, PPP3R1, GNG10, GNG5, RAC2, RAC1, PRKACA, PRKACB, JAK1, PDIA3, ARHGEF12, PRKCB, STAT3, HLA-B, HLA-C, PRKCA, HLA-A, RHOA, PTK2, PLCB3, PLCB4, GNAQ, GNB2, GNB1, GNAS, GRB2, KRAS, ARHGEF1, CALR, CALM1, PLCB2	708	226	8840	2.154642268	0.002584522	0.0000784	0.0000533	NSCLC_cluster2
KEGG_PATHWAY	hsa04540:Gap junction	22	2.054154995	0.00000847	PRKCB, SRC, TUBB, GNAI3, PRKCA, TUBB4B, TUBA4A, GNAI2, TUBA1C, PLCB3, NRAS, TUBA3C, TUBB2A, PLCB4, GNAQ, TUBB1, GNAS, GRB2, KRAS, PRKACA, PLCB2, PRKACB	708	92	8840	2.985752886	0.002714984	0.00008	0.0000543	NSCLC_cluster2
KEGG_PATHWAY	hsa05022:Pathways of neurodegeneration - multiple diseases	67	6.255835668	0.00000876	APP, ATP2A3, ATP2A2, PARK7, UBE2L3, TUBA1C, PSMD9, PSMD7, PSMD4, PSMD2, KIF5B, TUBB1, PSMD3, PSMD1, CAPN1, RAC1, RAB8A, MAP3K5, MAP2K3, GPX1, PRKCB, TUBB, PRKCA, TUBA4A, PSMA5, PSMA6, PSMA3, PLCB3, PSMA4, PLCB4, TUBB2A, PSMA1, PSMA2, CAT, PLCB2, RAB1A, VCP, PSMD11, PSMD13, DCTN1, KLC1, PSMA7, NRAS, PPP3R1, PSMB4, ATP5F1A, KLC2, TUBA3C, ATP5F1B, PSMB2, PSMB1, SNCA, BDNF, SEPTIN5, TUBB4B, EIF2S1, SOD1, PSMC6, PSMC3, PSMC1, GNAO, PSMC2, CHMP2B, KRAS, UBA1, CALM1, RAB5A	708	483	8840	1.731995181	0.002808153	0.0000803	0.0000546	NSCLC_cluster2
KEGG_PATHWAY	hsa04721:Synaptic vesicle cycle	20	1.867413632	0.0000102	NAPA, NSF, ATP6V1A, ATP6V1G1, CLTC, CLTB, CLTA, AP2A1, AP2B1, DNM1, SLC6A4, DNM2, DNM3, ATP6V1B2, AP2S1, ATP6V1H, ATP6V1E1, ATP6V1D, ATP6V1C1, AP2M1	708	79	8840	3.160981191	0.003256153	0.0000906	0.0000615	NSCLC_cluster2
KEGG_PATHWAY	hsa05203:Viral carcinogenesis	36	3.361344538	0.000013	YWHAE, DDX3X, YWHAB, SRC, UBR4, PIK3R1, CDC42, NRAS, YWHAQ, RAC1, PRKACA, PRKACB, YWHAG, JAK1, YWHAH, LYN, RANBP1, GSN, SYK, ACTN1, STAT3, HLA-B, HLA-C, EIF2AK2, ACTN4, HLA-A, YWHAZ, RHOA, SND1, DDB1, PKM, HNRNPK, PSMC1, MAPKAPK2, GRB2, KRAS	708	205	8840	2.192641587	0.004174157	0.000113	0.0000768	NSCLC_cluster2
KEGG_PATHWAY	hsa04814:Motor proteins	35	3.267973856	0.0000136	DYNC1I2, DCTN1, MYL6B, KLC1, ACTB, MYL12B, TUBA1C, KLC2, TUBA3C, CAPZB, KIF5B, TUBB1, MYO18A, MYH11, MYH10, DYNC1H1, TPM4, TPM3, TPM2, TUBB, TPM1, MYO5A, MYO9B, TUBB4B, DYNLL1, TUBA4A, ACTA2, MYL6, MYO1C, TUBB2A, KIF2A, CAPZA1, CAPZA2, MYH9, MYL9	708	197	8840	2.21830279	0.004370794	0.000115	0.0000783	NSCLC_cluster2
KEGG_PATHWAY	hsa04360:Axon guidance	33	3.081232493	0.0000208	ITGB1, ROCK1, ROCK2, SRC, GNAI3, ILK, PIK3R1, GNAI2, MYL12B, CDC42, EFNB1, PAK1, NRAS, PPP3R1, RRAS, CFL1, PLCG2, NCK2, RAC2, RAC1, EPHB2, SRGAP2, PAK2, ARHGEF12, PDPK1, PRKCA, RHOA, PTK2, FES, PARD3, PLXNB2, KRAS, MYL9	708	184	8840	2.239314665	0.00664999	0.000169	0.000115	NSCLC_cluster2
KEGG_PATHWAY	hsa04664:Fc epsilon RI signaling pathway	18	1.680672269	0.0000211	LYN, MAP2K3, VAV3, FCER1G, SYK, PDPK1, PRKCA, PIK3R1, VAV1, NRAS, INPP5D, BTK, PLCG2, RAC2, LCP2, GRB2, KRAS, RAC1	708	69	8840	3.257184967	0.006743796	0.000169	0.000115	NSCLC_cluster2
KEGG_PATHWAY	hsa05010:Alzheimer disease	56	5.22875817	0.0000222	APP, ATP2A3, ATP2A2, TUBA1C, PSMD9, PSMD7, PSMD4, PSMD2, KIF5B, TUBB1, PSMD3, PSMD1, CAPN1, MAP3K5, TUBB, SLC11A2, ADAM10, TUBA4A, PSMA5, PSMA6, PSMA3, PLCB3, PSMA4, PLCB4, TUBB2A, PSMA1, PSMA2, GAPDH, PLCB2, RTN3, PSMD11, PSMD13, PIK3R1, KLC1, RTN4, PSMA7, NRAS, PPP3R1, PSMB4, ATP5F1A, KLC2, TUBA3C, ATP5F1B, PSMB2, PSMB1, SNCA, EIF2AK2, TUBB4B, EIF2S1, PSMC6, PSMC3, PSMC1, GNAO, PSMC2, KRAS, CALM1	708	391	8840	1.788258413	0.007110538	0.000174	0.000118	NSCLC_cluster2

KEGG_PATHWAY	hsa04921:Oxytocin signaling pathway	29	2.707749767	0.0000295	ROCK1, ROCK2, SRC, PRKAG1, GNAI3, MYL6B, ACTB, GNAI2, MYLK, PPP1CB, NRAS, PPP3R1, PRKACA, PRKACB, PPP1R12A, PRKCB, PRKCA, EEF2, RHOA, PPP1CA, PLCB3, MYL6, PLCB4, GNAQ, GNAS, KRAS, CALM1, PLCB2, MYL9	708	154	8840	2.351236334	0.009438183	0.000226	0.000153	NSCLC_cluster2
KEGG_PATHWAY	hsa05120:Epithelial cell signaling in Helicobacter pylori infection	18	1.680672269	0.0000315	LYN, ATP6V1A, ATP6V1G1, SRC, ADAM10, F11R, CDC42, PAK1, ATP6V1B2, PLCG2, ATP6V1H, CSK, RAC1, ATP6V1E1, ATP6V1D, GIT1, ATP6V1C1, JAM3	708	71	8840	3.165433278	0.010045156	0.000235	0.000159	NSCLC_cluster2
KEGG_PATHWAY	hsa05417:Lipid and atherosclerosis	36	3.361344538	0.000041	HSP90AB1, ROCK2, SRC, PIK3R1, HSPD1, RAP1B, CDC42, NRAS, PPP3R1, RAP1A, CD36, RAC1, MAP3K5, VAV3, LYN, MAP2K3, HSPA8, HSP90AA1, HSPA4, PDPK1, STAT3, PRKCA, HSPA2, EIF2S1, RHOA, VAV1, PTK2, MIB1, SELP, PLCB3, PLCB4, KRAS, ARHGEF1, CALM1, PLCB2, HSPA1A	708	216	8840	2.080979284	0.013069472	0.000299	0.000203	NSCLC_cluster2
KEGG_PATHWAY	hsa00970:Aminoacyl-tRNA biosynthesis	17	1.587301587	0.0000455	AARS1, HARS1, RARS1, YARS1, HARS2, DARS1, GARS1, SARS1, QARS1, NARS1, EPRS1, LARS1, IARS1, CARS1, FARSA, TARS1, VARS1	708	66	8840	3.216058894	0.014492131	0.000324	0.00022	NSCLC_cluster2
KEGG_PATHWAY	hsa04730:Long-term depression	16	1.493930906	0.0000533	LYN, GNAZ, PRKCB, GNAI3, PRKCA, GNAI2, PPP2CA, GNA13, PLCB3, NRAS, PLCB4, PPP2R1A, GNAQ, GNAS, KRAS, PLCB2	708	60	8840	3.329566855	0.016969234	0.000372	0.000253	NSCLC_cluster2
KEGG_PATHWAY	hsa04720:Long-term potentiation	17	1.587301587	0.0000554	PRKCB, PRKCA, PPP1CA, RAP1B, PPP1CB, RPS6KA3, PLCB3, NRAS, PPP3R1, RAP1A, PLCB4, GNAQ, KRAS, CALM1, PRKACA, PLCB2,	708	67	8840	3.168058015	0.017624239	0.000378	0.000257	NSCLC_cluster2
KEGG_PATHWAY	hsa05014:Amyotrophic lateral sclerosis	52	4.855275444	0.0000815	ACTB, TUBA1C, PSMD9, PSMD7, PSMD4, PSMD2, KIF5B, TUBB1, PSMD3, ANXA7, PSMD1, RAC1, RAB8A, MAP3K5, MAP2K3, GPX1, TUBB, ANXA11, TUBA4A, PSMA5, PSMA6, PSMA3, PSMA4, TUBB2A, PSMA1, PSMA2, CAT, PFN1, RAB1A, VCP, PSMD11, PSMD13, DCTN1, KLC1, PSMA7, PPP3R1, PSMB4, ATP5F1A, KLC2, TUBA3C, ATP5F1B, PSMB2, PSMB1, TUBB4B, EIF2S1, SOD1, PSMC6, PSMC3, PSMC1, PSMC2,	708	371	8840	1.750041878	0.025821409	0.000545	0.00037	NSCLC_cluster2
KEGG_PATHWAY	hsa04728:Dopaminergic synapse	25	2.33426704	0.000108	GNAI3, ARRB1, GNAI2, PPP2CA, PPP1CB, GNG10, GNG5, PPP2R1A, PPP2R5E, KIF5B, PRKACA, PRKACB, PRKCB, PPP2R5A, PRKCA, PPP2R5D, PPP1CA, PLCB3, PLCB4, GNAQ, GNB2, GNB1, GNAS, CALM1,	708	132	8840	2.364749187	0.034071838	0.000707	0.00048	NSCLC_cluster2
KEGG_PATHWAY	hsa05110:Vibrio cholerae infection	14	1.307189542	0.000135	ATP6V1A, ATP6V1G1, PRKCA, ACTB, ATP6V1B2, PLCG2, GNAS, ATP6V1H, ATP6V1E1, PRKACA, ATP6V1D, PRKACB, ATP6V1C1, TJP2	708	51	8840	3.427495292	0.042322297	0.000865	0.000587	NSCLC_cluster2
KEGG_PATHWAY	hsa05146:Amoebiasis	21	1.960784314	0.000158	RAB5B, TGFB1, RAB5C, PRKCB, ACTN1, HSPB1, PRKCA, PIK3R1, ACTN4, PTK2, PLCB3, PLCB4, GNAQ, PRDX1, GNAS, PRKACA, RAB5A, PLCB2, PRKACB, VCL, RAB7A	708	103	8840	2.545663979	0.049597277	0.000997	0.000677	NSCLC_cluster2
KEGG_PATHWAY	hsa04971:Gastric acid secretion	17	1.587301587	0.000271	PRKCB, GNAI3, ATP1B3, PRKCA, ATP1A1, ACTB, GNAI2, MYLK, PLCB3, PLCB4, CA2, GNAQ, GNAS, CALM1, PRKACA, PLCB2, PRKACB	708	76	8840	2.79289325	0.083213477	0.001641213	0.001114593	NSCLC_cluster2
KEGG_PATHWAY	hsa04922:Glucagon signaling pathway	21	1.960784314	0.000271	PYGB, PGAM1, PRKAG1, PYGM, PYGL, LDHB, GYS1, LDHA, PLCB3, PFKL, PPP3R1, PKM, PLCB4, GNAQ, GNAS, CALM1, PRKACA, PLCB2, PFKM, PRKACB, PFKP	708	107	8840	2.45049897	0.083319297	0.001641213	0.001114593	NSCLC_cluster2
KEGG_PATHWAY	hsa04261:Adrenergic signaling in cardiomyocytes	26	2.427637722	0.000488	GNAI3, ATP2A3, ATP2A2, ATP1A1, GNAI2, PPP2CA, PPP1CB, PPP2R1A, PPP2R5E, PRKACA, PRKACB, TPM4, TPM3, TPM2, TPM1, PPP2R5A, PRKCA, PPP2R5D, ATP1B3, PPP1CA, PLCB3, PLCB4, GNAQ, GNAS, CALM1, PLCB2	708	154	8840	2.108004989	0.14508144	0.002902052	0.001970864	NSCLC_cluster2
KEGG_PATHWAY	hsa00480:Glutathione metabolism	14	1.307189542	0.00053	G6PD, GPX1, GPX4, GSTO1, GSTP1, IDH1, LANCL1, GSR, IDH2, TXNDC12, PGD, PRDX6, LAP3, GCLM	708	58	8840	3.013832067	0.156388259	0.003091234	0.002099343	NSCLC_cluster2
KEGG_PATHWAY	hsa04919:Thyroid hormone signaling pathway	22	2.054154995	0.000615	PRKCB, PDPK1, SRC, ITGB3, ATP2A3, ATP2A2, ATP1B3, PRKCA, PIK3R1, ATP1A1, ACTB, PLCB3, PFKL, NRAS, PLCB4, PLCG2, KRAS, PRKACA, PLCB2, PFKM, PRKACB, PFKP	708	122	8840	2.251551357	0.179144058	0.003462227	0.002351295	NSCLC_cluster2
KEGG_PATHWAY	hsa04660:T cell receptor signaling pathway	22	2.054154995	0.000615	VAV3, PDPK1, PPP2R5A, PPP2R5D, PIK3R1, RHOA, VAV1, CDC42, PPP2CA, PAK1, NRAS, PPP3R1, PTPRC, TEC, PPP2R1A, PPP2R5E, NCK2, LCP2, GRB2, KRAS, PTPN6, PAK2	708	122	8840	2.251551357	0.179144058	0.003462227	0.002351295	NSCLC_cluster2
KEGG_PATHWAY	hsa04972:Pancreatic secretion	20	1.867413632	0.00066	PRKCB, ATP2A3, ATP2A2, ATP1B3, PRKCA, RAB27B, ATP1A1, RHOA, RAB11A, RAP1B, BST1, PLCB3, RAP1A, PLCB4, CA2, GNAQ, GNAS, RAC1, PLCB2, RAB8A	708	106	8840	2.355825605	0.19089977	0.003651079	0.002479549	NSCLC_cluster2
KEGG_PATHWAY	hsa04370:VEGF signaling pathway	14	1.307189542	0.000747	PRKCB, SRC, HSPB1, PRKCA, PIK3R1, PTK2, CDC42, NRAS, PPP3R1, MAPKAPK2, PLCG2, RAC2, KRAS, RAC1	708	60	8840	2.913370998	0.213370726	0.004066246	0.0027615	NSCLC_cluster2
KEGG_PATHWAY	hsa05170:Human immunodeficiency virus 1 infection	32	2.987861811	0.000773	CUL5, GNAI3, PIK3R1, GNAI2, PAK1, NRAS, PPP3R1, GNG10, GNG5, CFL1, PLCG2, RAC2, RAC1, PAK2, AP1M1, SKP1, MAP2K3, PDIA3, PRKCB, AP1B1, HLA-B, HLA-C, PRKCA, HLA-A, PTK2, DDB1, GNAQ, GNB2, GNB1, KRAS, CALR, CALM1	708	213	8840	1.875812313	0.219878771	0.004136832	0.002809438	NSCLC_cluster2
KEGG_PATHWAY	hsa04014:Ras signaling pathway	34	3.174603175	0.00124483	RAB5B, RALB, RAB5C, PIK3R1, STK4, RASGRP2, RAP1B, CDC42, PAK1, NRAS, GNG10, RAP1A, RRAS, GNG5, PLCG2, RAC2, SHOC2, RAC1, PRKACA, PAK2, PRKACB, PRKCB, BDNF, PRKCA, RHOA, RASA3, GNB2, GNB1, GRB2, KRAS, CALM1, RAB5A, EXOC2, ARF6	708	238	8840	1.783696529	0.329572331	0.006445009	0.004376984	NSCLC_cluster2
KEGG_PATHWAY	hsa05171:Coronavirus disease - COVID-19	34	3.174603175	0.00124483	RPL5, RPL31, RPLP1, RPL12, RPLP0, F13A1, PIK3R1, RPL7, RPS4X, RPS17, RPS16, RPS19, PLCG2, RPS3, RPLP2, RPS2, RPS10, RPS13, JAK1, RPS9, SYK, RPS7, PRKCB, RPS8, STAT3, EIF2AK2, PRKCA, RPS3A, SELP, RPS25, FCGR2A, RPL27A, RPL26, RPS24	708	238	8840	1.783696529	0.329572331	0.006445009	0.004376984	NSCLC_cluster2
KEGG_PATHWAY	hsa04072:Phospholipase D signaling pathway	24	2.240896359	0.001627664	DGKG, RALB, FCER1G, SYK, PRKCA, PIK3R1, RHOA, DNM1, DNM2, DNM3, GNA13, CYTH2, PLCB3, NRAS, PLCB4, RRAS, PLCG2, GNAS, PIP5K1A, GRB2, KRAS, PIP5K1C, PLCB2, ARF6	708	149	8840	2.011147765	0.407204422	0.008293333	0.005632233	NSCLC_cluster2
KEGG_PATHWAY	hsa04070:Phosphatidylinositol signaling system	18	1.680672269	0.00180636	DGKG, PRKCB, PRKCA, PIK3R1, MTM1, PLCB3, PLCB4, PPIP5K2, INPP5D, PI4KA, PLCG2, PIP5K1A, PIP4K2A, PIP4K2B, PIP5K1C, PIP4K2C, CALM1, PLCB2	708	98	8840	2.293324109	0.440306479	0.009060025	0.006152914	NSCLC_cluster2

KEGG_PATHWAY	hsa04912:GnRH signaling pathway	17	1.587301587	0.002654543	MAP2K3, PRKCB, SRC, PRKCD, PRKCA, CDC42, PLCB3, NRAS, PLCB4, GNAQ, GNAS, GRB2, KRAS, CALM1, PRKACA, PLCB2, PRKACB	708	93	8840	2.282364376	0.573968133	0.013109356	0.008902927	NSCLC_cluster2
KEGG_PATHWAY	hsa04722:Neurotrophin signaling pathway	20	1.867413632	0.002988444	YWHAE, PDPK1, BDNF, PRKCD, PIK3R1, RHOA, CDC42, RAP1B, RPS6KA3, NRAS, RAP1A, ARHGDIA, ARHGDIB, MAPKAPK2, PLCG2, GRB2, KRAS, RAC1, CALM1, MAP3K5	708	120	8840	2.080979284	0.617385299	0.014328324	0.009730762	NSCLC_cluster2
KEGG_PATHWAY	hsa04148:Efferocytosis	24	2.240896359	0.002990647	RAB5B, TGFB1, TMEM30A, RAB5C, ITGB3, ATP2A3, ADAM10, ATP2A2, ANO6, THBS1, PTK2, RAB14, BSG, MAPKAPK2, ELMO1, PECAM1, PTPN6, CD47, CALR, CD36, RAC1, RAB5A, RAB7A, VPS16	708	156	8840	1.920903955	0.617656593	0.014328324	0.009730762	NSCLC_cluster2
KEGG_PATHWAY	hsa04910:Insulin signaling pathway	22	2.054154995	0.00303701	PYGB, PKLR, EXOC7, PDPK1, PRKAG1, PYGM, PYGL, PIK3R1, PPP1CA, PPP1CB, GYS1, NRAS, PRKAR1A, PRKAR2B, FASN, TRIP10, GRB2, KRAS, CALM1, PRKACA, EIF4E, PRKACB	708	138	8840	1.990501924	0.62332167	0.014336475	0.009736298	NSCLC_cluster2
KEGG_PATHWAY	hsa04926:Relaxin signaling pathway	21	1.960784314	0.003316853	TGFB1, SRC, GNAI3, PRKCA, ARRB1, PIK3R1, GNAI2, ACTA2, GNG10, PLCB3, NRAS, PLCB4, GNG5, GNB2, GNB1, GNAS, GRB2, KRAS, PRKACA, PLCB2, PRKACB	708	130	8840	2.016949153	0.655781778	0.015430577	0.010479333	NSCLC_cluster2
KEGG_PATHWAY	hsa04152:AMPK signaling pathway	20	1.867413632	0.003611559	RAB2A, CAB39, PDPK1, PRKAG1, PPP2R5A, PPP2R5D, PIK3R1, EEF2, RAB10, PPP2CA, GYS1, PFKL, RAB14, PPP2R1A, PPP2R5E, FASN, CD36, PFKM, RAB8A, PFKP	708	122	8840	2.04686487	0.686955162	0.016561576	0.011247426	NSCLC_cluster2
KEGG_PATHWAY	hsa05230:Central carbon metabolism in cancer	14	1.307189542	0.003720477	G6PD, PGAM1, IDH1, IDH2, PIK3R1, LDHB, LDHA, PFKL, NRAS, PKM, KRAS, SLC16A3, PFKM, PFKP	708	71	8840	2.46200366	0.697749891	0.01667318	0.011323219	NSCLC_cluster2
KEGG_PATHWAY	hsa03250:Viral life cycle - HIV-1	13	1.213818861	0.003739779	TSG101, PDCD6IP, MAP1S, VPS4B, VPS4A, SUPT5H, XPO1, CHMP4B, PIN1, CHMP4A, CHMP6, PPIA, RAN	708	63	8840	2.576450543	0.699623718	0.01667318	0.011323219	NSCLC_cluster2
KEGG_PATHWAY	hsa04928:Parathyroid hormone synthesis, secretion and action	19	1.774042951	0.004309216	NHERF1, PRKCB, GNAI3, PRKCA, ARRB1, OXSR1, RHOA, GNAI2, GNA13, PLCB3, PLCB4, WNK1, NACA, GNAQ, GNAS, ARHGEF1, PRKACA, PLCB2, PRKACB	708	115	8840	2.062883812	0.749988233	0.01869268	0.012694717	NSCLC_cluster2
KEGG_PATHWAY	hsa04726:Serotonergic synapse	19	1.774042951	0.004309216	APP, PRKCB, GNAI3, PRKCA, SLC6A4, GNAI2, GNG10, PLCB3, NRAS, PLCB4, GNG5, GNAQ, GNB2, GNB1, GNAS, KRAS, PRKACA, PLCB2, PRKACB	708	115	8840	2.062883812	0.749988233	0.01869268	0.012694717	NSCLC_cluster2
KEGG_PATHWAY	hsa00051:Fructose and mannose metabolism	9	0.840336134	0.00450078	PFKL, TKFC, TPI1, PMM2, AKR1B1, ALDOC, ALDOA, PFKM, PFKP	708	34	8840	3.305084746	0.764962765	0.019263337	0.013082266	NSCLC_cluster2
KEGG_PATHWAY	hsa04750:Inflammatory mediator regulation of TRP channels	17	1.587301587	0.005033563	MAP2K3, PRKCB, SRC, PRKCD, PRKCA, PIK3R1, PPP1CA, PPP1CB, PLCB3, PLCB4, GNAQ, PLCG2, GNAS, CALM1, PRKACA, PLCB2, PRKACB	708	99	8840	2.144039263	0.802072378	0.020984073	0.014250866	NSCLC_cluster2
KEGG_PATHWAY	hsa05231:Choline metabolism in cancer	17	1.587301587	0.005033563	DGKG, PCYT1B, SLC44A1, SLC44A2, PRKCB, PDPK1, WAS, PRKCA, PIK3R1, NRAS, RAC2, PIP5K1A, GRB2, KRAS, PIP5K1C, RAC1, WASF2	708	99	8840	2.144039263	0.802072378	0.020984073	0.014250866	NSCLC_cluster2
KEGG_PATHWAY	hsa04010:MAPK signaling pathway	38	3.548085901	0.005204601	HSPB1, ARRB1, STK4, RASGRP2, STK3, RAP1B, CDC42, RPS6KA3, PAK1, NRAS, PPP3R1, RAP1A, RRAS, STMN1, RAC2, FLNA, RAC1, PRKACA, PAK2, PRKACB, MAP4K4, MAP3K5, MAP2K3, HSPA8, TGFB1, DUSP3, PRKCB, BDNF, PRKCA, PPM1A, TAOK3, TAOK1, MAPKAPK2, RAPGEF2, GRB2, KRAS, LAMTOR3, HSPA1A	708	300	8840	1.581544256	0.812699223	0.021418933	0.014546191	NSCLC_cluster2
KEGG_PATHWAY	hsa04662:B cell receptor signaling pathway	16	1.493930906	0.005393646	LYN, VAV3, SYK, PRKCB, PIK3R1, VAV1, NRAS, PPP3R1, INPP5D, BTK, PLCG2, RAC2, GRB2, KRAS, PTPN6, RAC1	708	91	8840	2.195318805	0.82378427	0.021915953	0.014883731	NSCLC_cluster2
KEGG_PATHWAY	hsa04066:HIF-1 signaling pathway	18	1.680672269	0.0056524	PRKCB, STAT3, PRKCA, ENO1, PIK3R1, ENO2, LDHB, LDHA, PFKL, PGK1, PLCG2, ALDOC, PGK2, ALDOA, GAPDH, EIF4E, PFKM, PFKP	708	109	8840	2.061887731	0.837904216	0.022680257	0.015402791	NSCLC_cluster2
KEGG_PATHWAY	hsa05169:Epstein-Barr virus infection	28	2.614379085	0.005913123	PSMD11, PSMD13, PIK3R1, PSMD7, PSMD4, PSMD2, PSMD3, PLCG2, PSMD1, CD58, RAC1, JAK1, LYN, MAP2K3, PDIA3, SYK, STAT3, HLA-B, HLA-C, EIF2AK2, HLA-A, PSMC6, PSMC3, PSMC1, PSMC2, BTK, VIM,	708	203	8840	1.722189753	0.850990721	0.023433489	0.015914332	NSCLC_cluster2
KEGG_PATHWAY	hsa04918:Thyroid hormone synthesis	14	1.307189542	0.006035747	GPX1, PRKCB, GSR, ATP1B3, PRKCA, ATP1A1, PLCB3, PLCB4, GNAQ, CANX, GNAS, PRKACA, PLCB2, PRKACB	708	75	8840	2.330696798	0.856775984	0.02362774	0.016046253	NSCLC_cluster2
KEGG_PATHWAY	hsa04915:Estrogen signaling pathway	21	1.960784314	0.007128527	HSPA8, HSP90AA1, HSP90AB1, SRC, PRKCD, GNAI3, PIK3R1, GNAI2, PLCB3, NRAS, PLCB4, GNAQ, GNAS, GRB2, KRAS, CALM1, PRKACA, CTSD, PLCB2, PRKACB, HSPA1A	708	139	8840	1.886355323	0.89938482	0.027241157	0.018500225	NSCLC_cluster2
KEGG_PATHWAY	hsa04114:Oocyte meiosis	21	1.960784314	0.007128527	YWHAE, YWHAB, PPP2R5A, PPP2R5D, SMC3, YWHAZ, PPP1CA, PPP2CA, PPP1CB, RPS6KA3, PPP3R1, SLK, YWHAQ, PPP2R1A, PPP2R5E, CALM1, PRKACA, PRKACB, YWHAG, SKP1, YWHAH	708	139	8840	1.886355323	0.89938482	0.027241157	0.018500225	NSCLC_cluster2
KEGG_PATHWAY	hsa04150:mTOR signaling pathway	23	2.147525677	0.007258707	ATP6V1A, ATP6V1G1, CAB39, PRKCB, PDPK1, PRKCA, PIK3R1, RHOA, RPS6KA3, NRAS, ATP6V1B2, ATP6V1H, GRB2, KRAS, LAMTOR2, LAMTOR1, ATP6V1E1, LAMTOR3, ATP6V1D, EIF4E, LPIN2, ATP6V1C1, EIF4B	708	158	8840	1.817564185	0.903531873	0.027412293	0.018616448	NSCLC_cluster2
KEGG_PATHWAY	hsa04217:Necroptosis	23	2.147525677	0.007811257	PYGB, HSP90AA1, HSP90AB1, MLKL, VPS4B, VPS4A, STAT3, EIF2AK2, PYGM, HMGB1, PYGL, CHMP1B, CHMP2B, CHMP1A, CHMP2A, CHMP4B, CHMP3, CHMP4A, CAPN1, DNM1L, CHMP6, PPIA, JAK1	708	159	8840	1.806132964	0.91931945	0.02915597	0.019800628	NSCLC_cluster2
KEGG_PATHWAY	hsa04935:Growth hormone synthesis, secretion and action	19	1.774042951	0.008038958	MAP2K3, PRKCB, STAT3, GNAI3, PRKCA, PIK3R1, PTK2, GNAI2, PLCB3, NRAS, PLCB4, GNAQ, PLCG2, GNAS, GRB2, KRAS, PRKACA, PLCB2, PRKACB	708	122	8840	1.944521626	0.925049985	0.029660984	0.020143597	NSCLC_cluster2
KEGG_PATHWAY	hsa05414:Dilated cardiomyopathy	17	1.587301587	0.008916118	ITGB1, TGFB1, TPM4, TPM3, TPM2, ITGB3, ITGA2, TPM1, ITGA2B, ATP2A3, ATP2A2, ACTB, GNAS, ITGA6, ITGA5, PRKACA, PRKACB	708	105	8840	2.021522733	0.943578751	0.032523566	0.022087655	NSCLC_cluster2
KEGG_PATHWAY	hsa04713:Circadian entrainment	16	1.493930906	0.009752842	PRKCB, GNAI3, PRKCA, GNAI2, GNG10, PLCB3, PLCB4, GNG5, GNAQ, GNB2, GNB1, GNAS, CALM1, PRKACA, PLCB2, PRKACB	708	97	8840	2.05952589	0.956977354	0.03517598	0.023888983	NSCLC_cluster2
KEGG_PATHWAY	hsa04612:Antigen processing and presentation	14	1.307189542	0.010403412	PDIA3, HSPA8, HSP90AA1, HSP90AB1, HSPA4, HLA-B, HLA-C, HLA-A, HSPA2, CANX, PSME1, PSME2, CALR, HSPA1A	708	80	8840	2.185028249	0.965159955	0.037105504	0.025199376	NSCLC_cluster2
KEGG_PATHWAY	hsa05134:Legionellosis	11	1.027077498	0.012332689	EEF1G, EEF1A1, HSPA8, RAB1A, HBS1L, VCP, SAR1A, SAR1B, RAB1B, HSPD1, HSPA1A	708	56	8840	2.452582728	0.981377916	0.043251795	0.029373493	NSCLC_cluster2
KEGG_PATHWAY	hsa00562:Inositol phosphate metabolism	13	1.213818861	0.012396153	TPI1, MTM1, PLCB3, PLCB4, INPP5D, PI4KA, PLCG2, PIP5K1A, PIP4K2A, PIP4K2B, PIP5K1C, PIP4K2C, PLCB2	708	73	8840	2.223512112	0.981758098	0.043251795	0.029373493	NSCLC_cluster2

KEGG_PATHWAY	hsa05032:Morphine addiction	15	1.400560224	0.01283329	PRKCB, GNAI3, PRKCA, ARRB1, GNAI2, GNG10, GRK2, GNG5, GRK5, GNB2, GRK6, GNB1, GNAS, PRKACA, PRKACB	708	91	8840	2.05811138	0.984174739	0.04429555	0.030082336	NSCLC_cluster2
KEGG_PATHWAY	hsa04130:SNARE interactions in vesicular transport	8	0.746965453	0.013830288	VAMP8, SNAP23, STX4, YKT6, SNAP29, STX11, VTI1B, VAMP3	708	33	8840	3.026878959	0.988558501	0.047228961	0.032074497	NSCLC_cluster2
KEGG_PATHWAY	hsa04371:Apelin signaling pathway	20	1.867413632	0.015598792	PRKAG1, GNAI3, GNAI2, MYLK, GNA13, ACTA2, GNG10, PLCB3, NRAS, PLCB4, GNG5, RRAS, GNAQ, GNB2, GNB1, KRAS, CALM1, PRKACA, PLCB2, PRKACB	708	140	8840	1.783696529	0.993569329	0.052707497	0.035795122	NSCLC_cluster2
KEGG_PATHWAY	hsa01232:Nucleotide metabolism	14	1.307189542	0.016910955	DTYMK, ADSL, GMPR2, NME2, GMPR, CTPS2, AMPD2, CTPS1, APRT, TYMP, NME1, PNP, CMPK1, HPRT1	708	85	8840	2.056497175	0.995809104	0.056546007	0.038401961	NSCLC_cluster2
KEGG_PATHWAY	hsa03010:Ribosome	23	2.147525677	0.018635931	RPL5, RPS9, RPS7, RPL31, RPS8, RPLP1, RPL12, RPLP0, RPS3A, RPL7, RPS4X, RPS25, RPS17, RPS16, RPS19, RPL27A, RPS3, RPLP2, RPL26, RPS2, RPS10, RPS24, RPS13	708	172	8840	1.669622914	0.997615051	0.061671484	0.041882815	NSCLC_cluster2
KEGG_PATHWAY	hsa04724:Glutamatergic synapse	17	1.587301587	0.020321653	PRKCB, GNAI3, PRKCA, GNAI2, GNG10, PLCB3, PPP3R1, PLCB4, GRK2, GNG5, GNAQ, GNB2, GNB1, GNAS, PRKACA, PLCB2, PRKACB	708	115	8840	1.845738148	0.99862657	0.065341392	0.044375151	NSCLC_cluster2
KEGG_PATHWAY	hsa04725:Cholinergic synapse	17	1.587301587	0.020321653	PRKCB, GNAI3, PRKCA, PIK3R1, GNAI2, GNG10, PLCB3, NRAS, PLCB4, GNG5, GNAQ, GNB2, GNB1, KRAS, PRKACA, PLCB2, PRKACB	708	115	8840	1.845738148	0.99862657	0.065341392	0.044375151	NSCLC_cluster2
KEGG_PATHWAY	hsa03018:RNA degradation	13	1.213818861	0.020355574	DDX6, ENO1, ENO2, LSM4, LSM3, HSPD1, LSM2, EDC4, PFKL, LSM6, PABPC1, PFKM, PFKP	708	78	8840	2.080979284	0.998641751	0.065341392	0.044375151	NSCLC_cluster2
KEGG_PATHWAY	hsa04966:Collecting duct acid secretion	7	0.653594771	0.021077565	ATP6V1A, ATP6V1G1, CA2, ATP6V1B2, ATP6V1E1, ATP6V1D, ATP6V1C1	708	28	8840	3.121468927	0.998927991	0.066989094	0.045494151	NSCLC_cluster2
KEGG_PATHWAY	hsa04024:cAMP signaling pathway	28	2.614379085	0.022640421	ROCK1, ROCK2, GNAI3, ATP2A3, ATP2A2, ATP1A1, PIK3R1, GNAI2, RAP1B, PPP1CB, PAK1, RAP1A, RRAS, RAC2, RAC1, PRKACA, PRKACB, VAV3, ABCC4, PPP1R12A, BDNF, ATP1B3, RHOA, VAV1, PPP1CA, GNAS, CALM1, MYL9	708	226	8840	1.546922654	0.99935812	0.071250735	0.04838835	NSCLC_cluster2
KEGG_PATHWAY	hsa04022:cGMP-PKG signaling pathway	22	2.054154995	0.023662413	VASP, PPP1R12A, ROCK1, ROCK2, ATP2A3, GNAI3, ATP2A2, ATP1B3, ATP1A1, RHOA, GNAI2, PPP1CA, MYLK, GNA13, PPP1CB, PLCB3, PPP3R1, PLCB4, GNAQ, CALM1, PLCB2, MYL9	708	166	8840	1.654754612	0.999541222	0.073744024	0.050081611	NSCLC_cluster2
KEGG_PATHWAY	hsa05165:Human papillomavirus infection	38	3.548085901	0.024927161	ATP6V1A, ITGB1, ITGB3, ITGA2B, UBR4, PIK3R1, THBS1, PPP2CA, CDC42, NRAS, PPP2R1A, PPP2R5E, ATP6V1H, ATP6V1E1, PRKACA, ATP6V1D, PRKACB, ATP6V1C1, JAK1, ATP6V1G1, NHERF1, ITGA2, HLA-B, PPP2R5A, HLA-C, EIF2AK2, PPP2R5D, HLA-A, PTK2, PKM, PSMC1, PARD3, ATP6V1B2, GNAS, GRB2, KRAS, ITGA6, ITGA5	708	333	8840	1.424814645	0.999697381	0.076938641	0.052251164	NSCLC_cluster2
KEGG_PATHWAY	hsa05410:Hypertrophic cardiomyopathy	15	1.400560224	0.025377282	ITGB1, TGFB1, TPM4, TPM3, TPM2, ITGB3, ITGA2, TPM1, ITGA2B, PRKAG1, ATP2A3, ATP2A2, ACTB, ITGA6, ITGA5	708	99	8840	1.891799349	0.999739069	0.077581977	0.052688071	NSCLC_cluster2
KEGG_PATHWAY	hsa04140:Autophagy - animal	22	2.054154995	0.028216984	RAB1A, ATG3, PDPK1, PRKCD, HMGB1, PIK3R1, EIF2S1, VAMP8, PPP2CA, NRAS, LAMP1, RRAS, LAMP2, KRAS, PRKACA, CTSD, YKT6, SNAP29, PRKACB, RAB8A, RAB7A, VPS16	708	169	8840	1.625380269	0.99989773	0.085449547	0.058031156	NSCLC_cluster2
KEGG_PATHWAY	hsa05223:Non-small cell lung cancer	12	1.120448179	0.029523478	EML4, NRAS, PDPK1, PRKCB, KIF5B, STAT3, PLCG2, GRB2, KRAS, PRKCA, PIK3R1, STK4	708	73	8840	2.052472719	0.999933596	0.087157212	0.059190879	NSCLC_cluster2
KEGG_PATHWAY	hsa04650:Natural killer cell mediated cytotoxicity	18	1.680672269	0.029592787	VAV3, FCER1G, SYK, PRKCB, ICAM2, PRKCA, PIK3R1, VAV1, PAK1, NRAS, PPP3R1, PLCG2, RAC2, LCP2, GRB2, KRAS, PTPN6, RAC1	708	130	8840	1.728813559	0.999935101	0.087157212	0.059190879	NSCLC_cluster2
KEGG_PATHWAY	hsa04933:AGE-RAGE signaling pathway in diabetic complications	15	1.400560224	0.02959544	TGFB1, PRKCB, PRKCD, STAT3, PRKCA, PIK3R1, CDC42, DIAPH1, PLCB3, NRAS, PLCB4, PLCG2, KRAS, RAC1, PLCB2	708	101	8840	1.854337976	0.999935158	0.087157212	0.059190879	NSCLC_cluster2
KEGG_PATHWAY	hsa00620:Pyruvate metabolism	9	0.840336134	0.031218875	GRHPR, LDHB, LDHA, PKM, PKLR, MDH1, MDH2, ADH5, ALDH9A1	708	47	8840	2.390912369	0.999962117	0.091102353	0.061870134	NSCLC_cluster2
KEGG_PATHWAY	hsa05418:Fluid shear stress and atherosclerosis	19	1.774042951	0.031802895	HSP90AA1, HSP90AB1, GSTO1, SRC, GSTP1, ITGB3, ITGA2B, PIK3R1, TXN, RHOA, PTK2, ACTB, SUMO3, PECAM1, RAC2, RAC1, ARHGEF2, CALM1, MAP3K5	708	141	8840	1.682493889	0.999968784	0.091970535	0.06245974	NSCLC_cluster2
KEGG_PATHWAY	hsa03015:mRNA surveillance pathway	15	1.400560224	0.034305493	UPF2, HBS1L, DAZAP1, PYM1, PPP2R5A, PPP2R5D, GSPT2, GSPT1, PPP1CA, PPP2CA, PPP1CB, PPP2R1A, PPP2R5E, ETF1, PABPC1	708	103	8840	1.818331414	0.999986399	0.098321995	0.066773193	NSCLC_cluster2
KEGG_PATHWAY	hsa00500:Starch and sucrose metabolism	8	0.746965453	0.037237033	GPI, GYS1, PYGB, UGP2, PYGM, PYGL, GYG1, PGM1	708	40	8840	2.497175141	0.999994875	0.105779537	0.071837816	NSCLC_cluster2
KEGG_PATHWAY	hsa04625:C-type lectin receptor signaling pathway	15	1.400560224	0.039534571	ARHGEF12, FCER1G, SYK, SRC, PRKCD, PIK3R1, RHOA, PAK1, NRAS, PPP3R1, RRAS, MAPKAPK2, PLCG2, KRAS, CALM1	708	105	8840	1.783696529	0.99999762	0.111321029	0.075601197	NSCLC_cluster2
KEGG_PATHWAY	hsa04924:Renin secretion	11	1.027077498	0.046572382	PLCB3, PPP3R1, PLCB4, GNAQ, GNAI3, GNAS, CALM1, PRKACA, PLCB2, PRKACB, GNAI2	708	69	8840	1.990501924	0.999999775	0.129997692	0.088285037	NSCLC_cluster2
KEGG_PATHWAY	hsa04151:PI3K-Akt signaling pathway	39	3.641456583	0.049653168	ITGB1, YWHAE, HSP90AB1, YWHAB, ITGB3, ITGA2B, PIK3R1, THBS1, PPP2CA, GYS1, NRAS, GNG10, YWHAQ, GNG5, PPP2R1A, PPP2R5E, RAC1, EIF4E, YWHAG, EIF4B, JAK1, YWHAH, HSP90AA1, SYK, PDPK1, BDNF, ITGA2, PPP2R5A, PRKCA, PPP2R5D, YWHAZ, PTK2, CDC37, GNB2, GNB1, GRB2, KRAS, ITGA6, ITGA5	708	362	8840	1.345163405	0.999999921	0.137402302	0.093313713	NSCLC_cluster2
KEGG_PATHWAY	hsa04512:ECM-receptor interaction	13	1.213818861	0.050280875	ITGB1, GP1BB, ITGB3, ITGA2, ITGA2B, GP1BA, THBS1, GP5, GP9, ITGA6, CD47, CD36, ITGA5	708	89	8840	1.823779598	0.999999936	0.137708473	0.093521642	NSCLC_cluster2
KEGG_PATHWAY	hsa05211:Renal cell carcinoma	11	1.027077498	0.050621806	RAP1B, CDC42, PAK1, NRAS, TGFB1, RAP1A, GRB2, KRAS, RAC1, PIK3R1, PAK2	708	70	8840	1.962066182	0.999999943	0.137708473	0.093521642	NSCLC_cluster2
KEGG_PATHWAY	hsa04931:Insulin resistance	15	1.400560224	0.051648158	PYGB, PRKCB, PDPK1, PRKCD, STAT3, PRKAG1, PYGM, PYGL, PIK3R1, PPP1CA, PTPA, PPP1CB, RPS6KA3, GYS1, CD36	708	109	8840	1.718239776	0.99999996	0.13931982	0.094615953	NSCLC_cluster2
KEGG_PATHWAY	hsa00071:Fatty acid degradation	8	0.746965453	0.052424921	HADHB, HADHA, ECHS1, ACSL4, ACSL3, ACAA1, ADH5, ALDH9A1	708	43	8840	2.32295362	0.999999969	0.140236664	0.095238607	NSCLC_cluster2
KEGG_PATHWAY	hsa04213:Longevity regulating pathway - multiple species	10	0.933706816	0.056766427	HSPA8, NRAS, CAT, PRKAG1, KRAS, PIK3R1, PRKACA, PRKACB, SOD1, HSPA1A	708	62	8840	2.01385092	0.999999993	0.150595232	0.102273398	NSCLC_cluster2
KEGG_PATHWAY	hsa04916:Melanogenesis	14	1.307189542	0.058454322	PRKCB, GNAI3, PRKCA, GNAI2, PLCB3, NRAS, PLCB4, GNAQ, GNAS, KRAS, CALM1, PRKACA, PLCB2, PRKACB	708	101	8840	1.730715444	0.999999996	0.153801945	0.104451165	NSCLC_cluster2
KEGG_PATHWAY	hsa05167:Kaposi sarcoma-associated herpesvirus infection	23	2.147525677	0.065505165	LYN, ATG3, SYK, SRC, STAT3, HLA-B, HLA-C, EIF2AK2, HLA-A, PIK3R1, HCK, GNG10, NRAS, PPP3R1, GNG5, GNB2, MAPKAPK2, GNB1, PLCG2, KRAS, RAC1, CALM1, JAK1	708	196	8840	1.465179292	1	0.170952504	0.116098585	NSCLC_cluster2

KEGG_PATHWAY	hsa04929:GnRH secretion	10	0.933706816	0.072558516	PLCB3, NRAS, PLCB4, PRKCB, GNAQ, KRAS, ARRB1, PRKCA, PIK3R1, PLCB2	708	65	8840	1.920903955	1	0.187832931	0.127562552	NSCLC_cluster2
KEGG_PATHWAY	hsa04137:Mitophagy - animal	14	1.307189542	0.074992169	USP8, USP15, RAB5B, VCP, RAB5C, SRC, HUWE1, EIF2S1, NRAS, RRAS, KRAS, ARIH1, RAB5A, RAB7A	708	105	8840	1.664783427	1	0.19257989	0.130786342	NSCLC_cluster2
KEGG_PATHWAY	hsa04960:Aldosterone-regulated sodium reabsorption	7	0.653594771	0.07894523	PDPK1, PRKCB, ATP1B3, KRAS, PRKCA, ATP1A1, PIK3R1	708	38	8840	2.300029735	1	0.201122373	0.13658778	NSCLC_cluster2
KEGG_PATHWAY	hsa04012:ErbB signaling pathway	12	1.120448179	0.080330451	PAK1, NRAS, SRC, PRKCB, NCK2, PLCG2, GRB2, KRAS, PRKCA, PIK3R1, PAK2, PTK2	708	86	8840	1.742215215	1	0.203039958	0.137890065	NSCLC_cluster2
KEGG_PATHWAY	hsa05160:Hepatitis C	19	1.774042951	0.083788626	YWHAE, YWHAB, STAT3, EIF2AK2, PIK3R1, EIF2S1, YWHAZ, PPP2CA, CLDN5, NRAS, CLDN3, YWHAQ, PPP2R1A, GRB2, KRAS, EIF3E, YWHAG, JAK1, YWHAH	708	159	8840	1.492022883	1	0.210126163	0.142702503	NSCLC_cluster2
KEGG_PATHWAY	hsa04970:Salivary secretion	13	1.213818861	0.085212891	PRKCB, ATP1B3, PRKCA, ATP1A1, BST1, PLCB3, PLCB4, GNAQ, GNAS, CALM1, PRKACA, PLCB2, PRKACB	708	97	8840	1.673364785	1	0.212041379	0.14400318	NSCLC_cluster2
KEGG_PATHWAY	hsa05415:Diabetic cardiomyopathy	23	2.147525677	0.095376529	G6PD, TGFB1, PRKCB, PRKCD, GSR, ATP2A3, ATP2A2, PRKCA, PIK3R1, PPP1CA, PTPA, PPP1CB, GYS1, ATP5F1A, PLCB3, PLCB4, ATP5F1B, RAC2, CD36, RAC1, GAPDH, CTSD, PLCB2	708	205	8840	1.400854348	1	0.235506661	0.159939103	NSCLC_cluster2
KEGG_PATHWAY	hsa04727:GABAergic synapse	12	1.120448179	0.09701757	NSF, GNG10, GNG5, SRC, PRKCB, GNB2, GNB1, GNAI3, PRKCA, PRKACA, PRKACB, GNAI2	708	89	8840	1.683488859	1	0.23633613	0.160502418	NSCLC_cluster2
KEGG_PATHWAY	hsa00630:Glyoxylate and dicarboxylate metabolism	6	0.56022409	0.097184951	CS, GRHPR, MDH1, MDH2, CAT, PGP	708	31	8840	2.416621104	1	0.23633613	0.160502418	NSCLC_cluster2
KEGG_PATHWAY	hsa05200:Pathways in cancer	52	4.855275444	0.099487209	ITGB1, HSP90AB1, ITGA2B, TFG, RAC2, RAC1, PRKACA, PRKACB, JAK1, SKP1, HSP90AA1, ARHGEF12, TPM3, PRKCB, GSTO1, ITGA2, PRKCA, RHOA, EML4, PLCB3, PLCB4, ARHGEF1, ITGA6, PLCB2, RALB, ROCK1, ROCK2, GSTP1, GNAI3, PIK3R1, STK4, RASGRP2, GNAI2, GNA13, CDC42, NRAS, GNG10, GNG5, PLCG2, TGFB1, STAT3, PTK2, PML, GNAQ, GNB2, GNB1, GNAS, CCDC6, KRAS, GRB2, CALM1, F2RL3	708	533	8840	1.218134215	1	0.240115744	0.163069259	NSCLC_cluster2

supplementary table 3 Enriched pathways in fig4c									
**GroupID**	**Category**	**Term**	**Description**	**LogP**	**Log(q-value)**	**Genes**	**Symbols**	**InTerm_InList**	**Group**
1_Summary	GO Biological Processes	GO:0002181	cytoplasmic translation	-12.87063422	-8.696370842	6133,6136,6141,6146,6176,6181,6189,6202,6218,6224,6229,6230,11224,740,4666,23438,64978,6949,9188,54464,51631,51747	RPL9,RPL12,RPL18,RPL22,RPLP1,RPLP2,RPS3A,RPS8,RPS17,RPS20,RPS24,RPS25,RPL35,MRPL49,NACA,HARS2,MRPL38,TCOF1,DDX21,XRN1,LUC7L2,LUC7L3	22/-	Specific tissue up
2_Summary	GO Biological Processes	GO:0033627	cell adhesion mediated by integrin	-12.11462192	-8.241388538	2200,3674,3678,3684,3688,3689,3690,7448,255743,965,999,1829,3875,4627,4907,5777,5788,5880,6282,10398,23607,50848,10232,50509,1291,1303,7040,1889,6789	FBN1,ITGA2B,ITGA5,ITGAM,ITGB1,ITGB2,ITGB3,VTN,NPNT,CD58,CDH1,DSG2,KRT18,MYH9,NT5E,PTPN6,PTPRC,RAC2,S100A11,MYL9,CD2AP,F11R,MSLN,COL5A3,COL6A1,COL12A1,TGFB1,ECE1,STK4	29/-	Specific tissue up
3_Summary	GO Biological Processes	GO:0009611	response to wounding	-10.96001785	-7.536869177	948,1291,1361,1861,2153,2683,3678,3688,3690,4627,4666,5327,5359,5777,7040,10398,11235,23607,790,7057,7448,1829	CD36,COL6A1,CPB2,TOR1A,F5,B4GALT1,ITGA5,ITGB1,ITGB3,MYH9,NACA,PLAT,PLSCR1,PTPN6,TGFB1,MYL9,PDCD10,CD2AP,CAD,THBS1,VTN,DSG2	22/-	Specific tissue up
4_Summary	GO Biological Processes	GO:0030198	extracellular matrix organization	-10.82252411	-7.536869177	871,1291,1303,1306,2683,3688,4060,5768,7040,7045,7448,7837,10631,50509,255743,999,10058	SERPINH1,COL6A1,COL12A1,COL15A1,B4GALT1,ITGB1,LUM,QSOX1,TGFB1,TGFBI,VTN,PXDN,POSTN,COL5A3,NPNT,CDH1,ABCB6	17/-	Specific tissue up
5_Summary	GO Biological Processes	GO:0030335	positive regulation of cell migration	-8.706305476	-5.57343478	1512,3309,3674,3678,3688,3690,3958,5788,5880,6282,6789,7040,7057,7448,8766,10512,10631,11235,1306,2683,4627,7045,7837,5371,255743,1861,23616,26146	CTSH,HSPA5,ITGA2B,ITGA5,ITGB1,ITGB3,LGALS3,PTPRC,RAC2,S100A11,STK4,TGFB1,THBS1,VTN,RAB11A,SEMA3C,POSTN,PDCD10,COL15A1,B4GALT1,MYH9,TGFBI,PXDN,PML,NPNT,TOR1A,SH3BP1,TRAF3IP1	28/-	Specific tissue up
6_Summary	GO Biological Processes	GO:0051604	protein maturation	-8.188082945	-5.189910823	871,1191,1512,1861,1889,2590,2678,3309,3690,5327,5479,5611,5768,10525,23385,552900,7057,5371,1075,1361,23607	SERPINH1,CLU,CTSH,TOR1A,ECE1,GALNT2,GGT1,HSPA5,ITGB3,PLAT,PPIB,DNAJC3,QSOX1,HYOU1,NCSTN,BOLA2,THBS1,PML,CTSC,CPB2,CD2AP	21/-	Specific tissue up
7_Summary	GO Biological Processes	GO:0007159	leukocyte cell-cell adhesion	-7.752140013	-4.781996615	3678,3688,3689,4907,5788,5880,50848,871,948,1291,1303,4060,7837,8766,8826,9050,10398,23607,23616,50509,2203,4627,6513,4637,9138,11235,26146,51765	ITGA5,ITGB1,ITGB2,NT5E,PTPRC,RAC2,F11R,SERPINH1,CD36,COL6A1,COL12A1,LUM,PXDN,RAB11A,IQGAP1,PSTPIP2,MYL9,CD2AP,SH3BP1,COL5A3,FBP1,MYH9,SLC2A1,MYL6,ARHGEF1,PDCD10,TRAF3IP1,STK26	28/-	Specific tissue up
8_Summary	GO Biological Processes	GO:0006911	phagocytosis, engulfment	-7.465888064	-4.546897188	948,3684,3689,4627,7057,23616,976,1191,1291,2683,3148,3688,5359,7037,7040,23607,50848,3690,8766,999,2040,3958,5880,11235,483,6510,6513,9685,1861,4000,1361	CD36,ITGAM,ITGB2,MYH9,THBS1,SH3BP1,ADGRE5,CLU,COL6A1,B4GALT1,HMGB2,ITGB1,PLSCR1,TFRC,TGFB1,CD2AP,F11R,ITGB3,RAB11A,CDH1,STOM,LGALS3,RAC2,PDCD10,ATP1B3,SLC1A5,SLC2A1,CLINT1,TOR1A,LMNA,CPB2	31/-	Specific tissue up
9_Summary	GO Biological Processes	GO:2001233	regulation of apoptotic signaling pathway	-6.818649412	-3.986808712	1075,1191,1512,3148,3958,4000,5371,5788,6789,7057,8531,10525,51060,3875,7040,8428,11235,2683,3688	CTSC,CLU,CTSH,HMGB2,LGALS3,LMNA,PML,PTPRC,STK4,THBS1,YBX3,HYOU1,TXNDC12,KRT18,TGFB1,STK24,PDCD10,B4GALT1,ITGB1	19/-	Specific tissue up
10_Summary	GO Biological Processes	GO:1903036	positive regulation of response to wounding	-6.715321461	-3.902785916	948,1361,3688,5327,7057,7448,8428,5777,871,1075,1191,1512,4627,5371,23385,3958,5788,9555,23616	CD36,CPB2,ITGB1,PLAT,THBS1,VTN,STK24,PTPN6,SERPINH1,CTSC,CLU,CTSH,MYH9,PML,NCSTN,LGALS3,PTPRC,MACROH2A1,SH3BP1	19/-	Specific tissue up
11_Summary	GO Biological Processes	GO:1901654	response to ketone	-6.659389302	-3.865337163	790,1829,2153,2203,3684,5788,7040,7057,10631,54464,1291,1512,1841,2200,3148,3417,3690,5371,6513,23607	CAD,DSG2,F5,FBP1,ITGAM,PTPRC,TGFB1,THBS1,POSTN,XRN1,COL6A1,CTSH,DTYMK,FBN1,HMGB2,IDH1,ITGB3,PML,SLC2A1,CD2AP	20/-	Specific tissue up
12_Summary	GO Biological Processes	GO:0001775	cell activation	-6.608645757	-3.850709707	1191,3133,3684,3688,3689,3690,4627,5327,5359,5777,5788,6146,7040,10398,10875,23385,23607,948,3958,5880,7037,9188,999,7045,7057,10631,1361,4907,7448,26146,965,3148,4060,1291,2683,4361,6789,1075,50848,2203,5611,8826,9555,1512,5371,6275,2200,2678,55379	CLU,HLA-E,ITGAM,ITGB1,ITGB2,ITGB3,MYH9,PLAT,PLSCR1,PTPN6,PTPRC,RPL22,TGFB1,MYL9,FGL2,NCSTN,CD2AP,CD36,LGALS3,RAC2,TFRC,DDX21,CDH1,TGFBI,THBS1,POSTN,CPB2,NT5E,VTN,TRAF3IP1,CD58,HMGB2,LUM,COL6A1,B4GALT1,MRE11,STK4,CTSC,F11R,FBP1,DNAJC3,IQGAP1,MACROH2A1,CTSH,PML,S100A4,FBN1,GGT1,LRR	49/-	Specific tissue up
13_Summary	GO Biological Processes	GO:0044319	wound healing, spreading of cells	-6.288873794	-3.737859703	1861,3678,3688,3690,11235,5359,6510,7037,50848,255743,2040,5479,5725	TOR1A,ITGA5,ITGB1,ITGB3,PDCD10,PLSCR1,SLC1A5,TFRC,F11R,NPNT,STOM,PPIB,PTBP1	13/-	Specific tissue up
14_Summary	GO Biological Processes	GO:0060627	regulation of vesicle-mediated transport	-6.27136132	-3.730566394	948,1191,1861,3684,3689,3690,3958,5359,5788,5880,7040,7448,8766,10960	CD36,CLU,TOR1A,ITGAM,ITGB2,ITGB3,LGALS3,PLSCR1,PTPRC,RAC2,TGFB1,VTN,RAB11A,LMAN2	14/-	Specific tissue up
15_Summary	GO Biological Processes	GO:0010763	positive regulation of fibroblast migration	-6.056179652	-3.580886275	3688,3690,7040,7057,2200,1291,3684,6513,10631,50848,790,1829,2683,3678,3674,3958,5880,1512,1361,5359,948,6789,8766,483,2040,5371,5777,4627,23607,23616,5788,2203,8531,999,7037,3148,11235,255743	ITGB1,ITGB3,TGFB1,THBS1,FBN1,COL6A1,ITGAM,SLC2A1,POSTN,F11R,CAD,DSG2,B4GALT1,ITGA5,ITGA2B,LGALS3,RAC2,CTSH,CPB2,PLSCR1,CD36,STK4,RAB11A,ATP1B3,STOM,PML,PTPN6,MYH9,CD2AP,SH3BP1,PTPRC,FBP1,YBX3,CDH1,TFRC,HMGB2,PDCD10,NPNT	38/-	Specific tissue up
16_Summary	GO Biological Processes	GO:0045785	positive regulation of cell adhesion	-5.788711235	-3.346841614	948,3133,3678,3689,3690,5777,5788,6789,7037,7040,7448,50848,255743,3309,4361,5371,5725,7057,8826,11235,133619,5880,10631,9555	CD36,HLA-E,ITGA5,ITGB2,ITGB3,PTPN6,PTPRC,STK4,TFRC,TGFB1,VTN,F11R,NPNT,HSPA5,MRE11,PML,PTBP1,THBS1,IQGAP1,PDCD10,PRRC1,RAC2,POSTN,MACROH2A1	24/-	Specific tissue up

17_Summary	GO Biological Processes	GO:1901342	regulation of vasculature development	-5.478829937	-3.104814717	948,1512,1634,3678,3688,3690,5371,5777,7040,7057,11235,8766,23616,1361,2683,3148,3150,6789,3133,4000,6282,999,8428,51765	CD36,CTSH,DCN,ITGA5,ITGB1,ITGB3,PML,PTPN6,TGFB1,THBS1,PDCD10,RAB11A,SH3BP1,CPB2,B4GALT1,HMGB2,HMGN1,STK4,HLA-	24/-	Specific tissue up
18_Summary	GO Biological Processes	GO:0048871	multicellular organismal-level homeostasis	-5.402708397	-3.047988952	948,1291,1512,3688,4907,5880,6218,6229,6510,6513,7040,23385,23607,50848	CD36,COL6A1,CTSH,ITGB1,NT5E,RAC2,RPS17,RPS24,SLC1A5,SLC2A1,TGFB1,NCSTN,CD2AP,F11R	14/-	Specific tissue up
19_Summary	GO Biological Processes	GO:0036293	response to decreased oxygen levels	-5.223167071	-2.91222655	1291,4000,5327,5371,6513,7037,7040,7057,10525,10631	COL6A1,LMNA,PLAT,PML,SLC2A1,TFRC,TGFB1,THBS1,HYOU1,POSTN	10/-	Specific tissue up
20_Summary	GO Biological Processes	GO:0031667	response to nutrient levels	-5.022858611	-2.756892819	790,1291,2153,2203,3309,6513,7037,7040,8428,9563,10631,51765,948,1861,3417,7837,11235,23607,8531,6789,5777,7150	CAD,COL6A1,F5,FBP1,HSPA5,SLC2A1,TFRC,TGFB1,STK24,H6PD,POSTN,STK26,CD36,TOR1A,IDH1,PXDN,PDCD10,CD2AP,YBX3,STK4,PTPN6,TOP1	22/-	Specific tissue up
1_Summary	GO Biological Processes	GO:0030029	actin filament-based process	-35.41326818	-31.41765454	60,87,226,391,395,408,817,829,830,832,1073,1627,1785,2017,2314,2316,2318,2885,3071,5048,5216,5339,5872,6093,6714,7094,7168,7169,7170,7171,7791,8394,8440,8452,9124,9168,9181,9260,9266,9322,9368,9475,9578,9938,9948,10006,10092,10093,10094,10096,10097,10109,10163,10611,10787,10788,11034,11151,11344,23002,23136,23224,23380,23396,23603,28988,29780,54874,55845,57175,63916,64423,94134,116985,345651,351,567,3326,3837,3872,3892,4134,7461,8239,9748,9793,22919,50810,57606,79875,81027	ACTB,ACTN1,ALDOA,RHOG,ARHGAP6,ARRB1,CAMK2D,CAPZA1,CAPZA2,CAPZB,CFL2,DBN1,DNM2,CTTN,FLII,FLNA,FLNC,GRB2,NCKAP1L,PAFAH1B1,PFN1,PLEC,RAB13,ROCK1,SRC,TLN1,TPM1,TPM2,TPM3,TPM4,ZYX,PIP5K1A,NCK2,CUL3,PDLIM1,TMSB10,ARHGEF2,PDLIM7,CYTH2,TRIP10,NHERF1,ROCK2,CDC42BPB,ARHGAP25,WDR1,ABI1,ARPC5,ARPC4,ARPC3,ACTR3,ACTR2,ARPC2,WASF2,PDLIM5,NCKAP1,IQGAP2,DSTN,CORO1A,TWF2,DAAM1,EPB41L3,SYNE2,SRGAP2,PIP5K1C,CORO1C,DBNL,PARVB,FNBP1L,BRK1,CORO1B,ELMO2,INF2,ARHGAP12,ARAP1,ACTBL2,APP,B2M,HSP90AB1,KPNB1,KRT17,KRT86,MAP4,CLIP2,USP9X,SLK,CKAP5,MAPRE1,HDGFL3,SLAIN2,THSD4,TUBB1	91/-	Specific serum up
2_Summary	GO Biological Processes	GO:0051493	regulation of cytoskeleton organization	-28.72016332	-25.02302119	391,395,824,829,830,832,1073,1213,1639,2011,2017,2242,2314,2316,2885,3055,3071,5048,5119,5216,5898,6093,6188,6387,7168,8440,8874,9168,9181,9353,9475,9793,9948,10092,10094,10109,10163,10787,10788,10979,10982,11034,11151,11344,22919,23191,23406,26999,28964,50810,51571,55201,55845,57175,57551,57606,83700,91624,93663,116985,136319,256364,351,2876,19,695,1981,3020,3320,5336,5515,5610,5899,6714,7415,7453,51128,55612,56681,65082,393,1173,1759,1785,2335,2664,6696,8301,9368,23108,51520,57142,57609,196527,592730,4313,120892,817,1113,2048,5578,7058,9908,10096,10097,26052,50807,54874,79837,112574,5007,5532,7203,8452,9141,10367,10694,22948,23376,51433,65992,93974	RHOG,ARHGAP6,CAPN2,CAPZA1,CAPZA2,CAPZB,CFL2,CLTC,DCTN1,MARK2,CTTN,FES,FLII,FLNA,GRB2,HCK,NCKAP1L,PAFAH1B1,CHMP1A,PFN1,RALA,ROCK1,RPS3,CXCL12,TPM1,NCK2,ARHGEF7,TMSB10,ARHGEF2,SLIT2,ROCK2,CKAP5,WDR1,ARPC5,ARPC3,ARPC2,WASF2,NCKAP1,IQGAP2,FERMT2,MAPRE2,DSTN,CORO1A,TWF2,MAPRE1,CYFIP1,COTL1,CYFIP2,GIT1,HDGFL3,CYRIB,MAP1S,BRK1,CORO1B,TAOK1,SLAIN2,JAM3,NEXN,ARHGAP18,ARAP1,MTPN,EML3,APP,GPX1,ABCA1,BTK,EIF4G1,H3-3A,HSP90AA1,PLCG2,PPP2CA,EIF2AK2,RALB,SRC,VCP,WARS1,SAR1B,FERMT1,SAR1A,VPS33A,ARHGAP4,AP2M1,DNM1,DNM2,FN1,GDI1,SPP1,PICALM,NHERF1,RAP1GAP2,LARS1,RTN4,DIP2B,ANO6,ACTA2,GCLM,MMP2,LRRK2,CAMK2D,CHGA,EPHB2,PRKCA,THBS2,G3BP2,ACTR3,ACTR2,DNM3,ASAP1,FNBP1L,PIP4K2C,SNX18,OSBP,PPP3CB,CCT3,CUL3,PDCD5,MICU1,CCT8,CCT5,UFL1,A	123/-	Specific serum up
3_Summary	GO Biological Processes	GO:0006897	endocytosis	-25.4389276	-21.96363423	19,160,163,351,408,1173,1175,1211,1212,1213,1759,1785,1991,2017,2207,2621,2885,3055,3385,3988,4067,4830,5861,5874,5898,5899,6455,6642,6714,8301,8394,8547,9260,9261,9266,9322,9938,10163,10938,11151,23184,23396,23603,26052,28988,30844,30845,50618,51411,51606,54874,63916,85021,94134,112574,115548,120892,2181,8036,9368,57181	ABCA1,AP2A1,AP2B1,APP,ARRB1,AP2M1,AP2S1,CLTA,CLTB,CLTC,DNM1,DNM2,ELANE,CTTN,FCER1G,GAS6,GRB2,HCK,ICAM3,LIPA,LYN,NME1,RAB1A,RAB27B,RALA,RALB,SH3GL1,SNX1,SRC,PICALM,PIP5K1A,FCN3,PDLIM7,MAPKAPK2,CYTH2,TRIP10,ARHGAP25,WASF2,EHD1,CORO1A,MESD,PIP5K1C,CORO1C,DNM3,DBNL,EHD4,EHD3,ITSN2,BIN2,ATP6V1H,FNBP1L,ELMO2,REPS1,ARHGAP12,SNX18,FCHO2,LRRK2,ACSL3,SHOC2,NHERF1,SL	61/-	Specific serum up
4_Summary	GO Biological Processes	GO:0051336	regulation of hydrolase activity	-20.11657786	-17.13096901	12,29,325,391,710,831,1475,1509,1893,2168,2260,2621,2876,3071,3326,3329,3336,3655,4067,5524,5657,5905,6001,6059,6091,6093,6188,6714,6774,6906,7076,7078,7415,8394,8874,9141,9475,9693,9938,10235,10979,10982,23380,23526,23603,23654,26000,26999,51035,51291,51520,55031,65992,81704,93974,94134,112574,116985,120892	SERPINA3,ABR,APCS,RHOG,SERPING1,CAST,CSTA,CTSD,ECM1,FABP1,FGFR1,GAS6,GPX1,NCKAP1L,HSP90AB1,HSPD1,HSPE1,ITGA6,LYN,PTPA,PRTN3,RANGAP1,RGS10,ABCE1,ROBO1,ROCK1,RPS3,SRC,STAT3,SERPINA7,TIMP1,TIMP3,VCP,PIP5K1A,ARHGEF7,PDCD5,ROCK2,RAPGEF2,ARHGAP25,RASGRP2,FERMT2,MAPRE2,SRGAP2,ARHGAP45,CORO1C,PLXNB2,TBC1D10B,CYFIP2,UBXN1,GMIP,LARS1,USP47,DDRGK1,DOCK8,ATP5IF1,ARHGAP12,SNX18,ARAP1,LRRK2	59/-	Specific serum up

5_Summary	GO Biological Processes	GO:0006886	intracellular protein transport	-20.1011124	-17.13096901	160,163,372,381,972,1173,1175,1211,1212,1213,1314,2017,3308,3315,3320,3837,4218,5494,5534,5861,5874,6400,6642,6643,6774,7353,7415,7514,7533,8239,8676,9181,10527,10768,10938,22931,23230,23256,23534,26060,30849,51128,51560,51762,54832,54899,56681,56850,58533,65082,81876,84313,120892,1113,3329,5898,5905,6730,7295,7531,8359,9184,10982,11021,22919,50807,55754,57142,65992,84823,114882,375056,1977,5571,6059,55308	AP2A1,AP2B1,ARCN1,ARF5,CD74,AP2M1,AP2S1,CLTA,CLTB,CLTC,COPA,CTTN,HSPA4,HSPB1,HSP90AA1,KPNB1,RAB8A,PPM1A,PPP3R1,RAB1A,RAB27B,SEL1L,SNX1,SNX2,STAT3,UFD1,VCP,XPO1,YWHAH,USP9X,STX11,ARHGEF2,IPO7,AHCYL1,EHD1,RAB18,VPS13A,SCFD1,TNPO3,APPL1,PIK3R4,SAR1B,RAB6B,RAB8B,VPS13C,PXK,SAR1A,GRIPAP1,SNX6,VPS33A,RAB1B,VPS25,LRRK2,CHGA,HSPD1,RALA,RANGAP1,SRP68,TXN,YWHAE,H4C1,BUB3,MAPRE2,RAB35,MAPRE1,ASAP1,TMEM30A,RTN4,DDRGK1,LMNB2,OSBPL8,MIA3,EIF4E,PRKAG1,ABCE1,DDX1	76/-	Specific serum up
6_Summary	GO Biological Processes	GO:0051640	organelle localization	-19.24527415	-16.34976437	372,695,1000,1113,1639,1759,1785,2011,3837,4067,4134,4218,5048,5119,5413,5537,5861,6059,7514,7534,8301,8452,8676,9184,9341,9342,9368,10096,10097,10652,10726,10768,11336,22919,23224,23241,23265,23344,26052,51128,54874,55201,55968,56288,56681,60412,64837,65082,84823,120892,256364,19,1173,2048,2621,3308,3320,5339,5578,5579,9938,11151,23136,23396,23603,28988,51411,55754,57142,84313,93974,94134,112574,114882,115548,196527,375056,5862,8874,10159,10313,22931,51560,51606,81876	ARCN1,BTK,CDH2,CHGA,DCTN1,DNM1,DNM2,MARK2,KPNB1,LYN,MAP4,RAB8A,PAFAH1B1,CHMP1A,SEPTIN5,PPP6C,RAB1A,ABCE1,XPO1,YWHAZ,PICALM,CUL3,STX11,BUB3,VAMP3,SNAP29,NHERF1,ACTR3,ACTR2,YKT6,NUDC,AHCYL1,EXOC3,MAPRE1,SYNE2,PACS2,EXOC7,ESYT1,DNM3,SAR1B,FNBP1L,MAP1S,NSFL1C,PARD3,SAR1A,EXOC4,KLC2,VPS33A,LMNB2,LRRK2,EML3,ABCA1,AP2M1,EPHB2,GAS6,HSPA4,HSP90AA1,PLEC,PRKCA,PRKCB,ARHGAP25,CORO1A,EPB41L3,PIP5K1C,CORO1C,DBNL,BIN2,TMEM30A,RTN4,VPS25,ATP5IF1,ARHGAP12,SNX18,OSBPL8,FCHO2,ANO6,MIA3,RAB2A,ARHGEF7,ATP6AP2,RTN3,RAB18,RAB6B,ATP6V1H,RAB1B	85/-	Specific serum up
7_Summary	GO Biological Processes	GO:0031346	positive regulation of cell projection organization	-18.42015912	-15.58831842	160,816,1627,2011,2048,2242,2260,2316,2335,2664,3320,3655,4067,5048,5216,5898,6091,6387,6714,8036,8874,9353,9693,10096,10097,10109,10163,10787,10938,11344,23191,23603,23654,26052,50807,51762,54874,55754,55845,57175,116985,391,393,567,832,4134,6696,7533,10611,23224,54521,57142,57609,120892,1785,2017,9368,23108,51520,136319,196527,60,695,972,1462,2621,3071,4673,4860,5196,5578,8650,9370,23376,51593,65992,102,817,1981,2273,2539,3020,3371,3872,10235,50618,55031,7058,56288,351,5515,5579,6774,8455,9518	AP2A1,CAMK2B,DBN1,MARK2,EPHB2,FES,FGFR1,FLNA,FN1,GDI1,HSP90AA1,ITGA6,LYN,PAFAH1B1,PFN1,RALA,ROBO1,CXCL12,SRC,SHOC2,ARHGEF7,SLIT2,RAPGEF2,ACTR3,ACTR2,ARPC2,WASF2,NCKAP1,EHD1,TWF2,CYFIP1,CORO1C,PLXNB2,DNM3,ASAP1,RAB8B,FNBP1L,TMEM30A,BRK1,CORO1B,ARAP1,RHOG,ARHGAP4,B2M,CAPZB,MAP4,SPP1,YWHAH,PDLIM5,SYNE2,WDR44,RTN4,DIP2B,LRRK2,DNM2,CTTN,NHERF1,RAP1GAP2,LARS1,MTPN,ANO6,ACTB,BTK,CD74,VCAN,GAS6,NCKAP1L,NAP1L1,PNP,PF4,PRKCA,NUMB,ADIPOQ,UFL1,SRRT,DDRGK1,ADAM10,CAMK2D,EIF4G1,FHL1,G6PD,H3-3A,TNC,KRT17,RASGRP2,ITSN2,USP47,THBS2,PARD3,APP,PPP2CA,PRKCB,STAT3,ATRN,GDF15	95/-	Specific serum up
8_Summary	GO Biological Processes	GO:0060627	regulation of vesicle-mediated transport	-17.03526647	-14.2589431	60,160,408,566,567,695,717,1173,1175,1445,1535,2181,2207,2212,2242,2621,3055,3071,4043,4067,4153,5336,5413,5532,5534,5579,5657,5874,5898,6093,6714,8301,8547,8650,8832,9370,10938,11151,23256,26060,28964,30844,54521,55589,56850,120892,196527,102,351,972,2260,2316,2664,3263,3315,3320,3329,3956,5515,5578,6387,7531,9261,9353,9782,57142,710,5094,5537,5610,7353,7534,11140,55914,23376,1,2885,5284	ACTB,AP2A1,ARRB1,AZU1,B2M,BTK,C2,AP2M1,AP2S1,CSK,CYBA,ACSL3,FCER1G,FCGR2A,FES,GAS6,HCK,NCKAP1L,LRPAP1,LYN,MBL2,PLCG2,SEPTIN5,PPP3CB,PPP3R1,PRKCB,PRTN3,RAB27B,RALA,ROCK1,SRC,PICALM,FCN3,NUMB,CD84,ADIPOQ,EHD1,CORO1A,SCFD1,APPL1,GIT1,EHD4,WDR44,BMP2K,GRIPAP1,LRRK2,ANO6,ADAM10,APP,CD74,FGFR1,FLNA,GDI1,HPX,HSPB1,HSP90AA1,HSPD1,LGALS1,PPP2CA,PRKCA,CXCL12,YWHAE,MAPKAPK2,SLIT2,MATR3,RTN4,SERPING1,PCBP2,PPP6C,EIF2AK2,UFD1,YWHAZ,CDC37,ERBIN,UFL1,A1BG,GRB2,PI	78/-	Specific serum up
9_Summary	GO Biological Processes	GO:0002252	immune effector process	-16.75509329	-13.99580326	351,566,567,629,695,710,717,722,727,729,730,731,732,733,735,972,1113,1511,1675,1991,2207,2212,2885,3055,3251,3329,3426,3716,3956,3988,4067,4153,5336,5339,5532,6714,6774,8547,9948,10383,11151,51279,81494,203068,325,1535,1536,3107,3263,5094,5537,5610,7791,8349,8832,9097,9181,9341,9782,9908,10096,10097,10527,23052,66005,5196,1445,2664,3071,3320,5515,5579,6188,7531,9261,51571,57142,57181,65992,2821,5657,23108,28775,28874,28877,28988,83700	APP,AZU1,B2M,CFB,BTK,SERPING1,C2,C4BPA,C5,C6,C7,C8A,C8B,C8G,C9,CD74,CHGA,CTSG,CFD,ELANE,FCER1G,FCGR2A,GRB2,HCK,HPRT1,HSPD1,CFI,JAK1,LGALS1,LIPA,LYN,MBL2,PLCG2,PLEC,PPP3CB,SRC,STAT3,FCN3,WDR1,TUBB4B,CORO1A,C1RL,CFHR5,TUBB,APCS,CYBA,CYBB,HLA-C,HPX,PCBP2,PPP6C,EIF2AK2,ZYX,H2BC21,CD84,USP14,ARHGEF2,VAMP3,MATR3,G3BP2,ACTR3,ACTR2,IPO7,ENDOD1,CHID1,PF4,CSK,GDI1,NCKAP1L,HSP90AA1,PPP2CA,PRKCB,RPS3,YWHAE,MAPKAPK2,CYRIB,RTN4,SLC39A10,DDRGK1,GPI,PRTN3,RAP1GAP2,IGLV7-46,IGKV3D-20,IGKV3D-7,DBNL,JAM3	87/-	Specific serum up

10_Summary	GO Biological Processes	GO:0000902	cell morphogenesis	-16.135534	-13.39263438	60,87,351,832,1000,1785,2017,2048,2316,2811,3071,3251,3988,4147,4218,5048,5339,5532,6091,6093,6387,6642,6643,7171,7204,7414,8239,8301,8452,8650,9181,9353,9368,9948,10006,10163,10787,23136,23181,23191,23256,23380,26999,28988,55201,56288,64072,65082,80739,81027,91624,120892,345651,2011,2260,3371,4067,4134,4313,5872,8440,9693,10938,11021,50810,51560,57142,10611,1893,2242,2876,3020,6714,51119,136319	ACTB,ACTN1,APP,CAPZB,CDH2,DNM2,CTTN,EPHB2,FLNA,GP1BA,NCKAP1L,HPRT1,LIPA,MATN2,RAB8A,PAFAH1B1,PLEC,PPP3CB,ROBO1,ROCK1,CXCL12,SNX1,SNX2,TPM4,TRIO,VCL,USP9X,PICALM,CUL3,NUMB,ARHGEF2,SLIT2,NHERF1,WDR1,ABI1,WASF2,NCKAP1,EPB41L3,DIP2A,CYFIP1,SCFD1,SRGAP2,CYFIP2,DBNL,MAP1S,PARD3,CDH23,VPS33A,MPIG6B,TUBB1,NEXN,LRRK2,ACTBL2,MARK2,FGFR1,TNC,LYN,MAP4,MMP2,RAB13,NCK2,RAPGEF2,EHD1,RAB35,HDGFL3,RAB6B,RTN4,PDLIM5,ECM1,FES,GPX1,H3-3A,SRC,SBDS,MTPN	75/-	Specific serum up
11_Summary	GO Biological Processes	GO:0034330	cell junction organization	-15.87769506	-13.15058971	60,87,102,351,395,829,831,1000,1445,1639,2017,2048,2316,2335,3371,3655,5216,5339,5578,5657,5872,6714,7094,7414,8394,8404,8650,8874,9414,9948,10163,10979,23136,23181,23191,23380,23396,23654,26052,26060,28988,51762,56288,83700,120892,345651	ACTB,ACTN1,ADAM10,APP,ARHGAP6,CAPZA1,CAST,CDH2,CSK,DCTN1,CTTN,EPHB2,FLNA,FN1,TNC,ITGA6,PFN1,PLEC,PRKCA,PRTN3,RAB13,SRC,TLN1,VCL,PIP5K1A,SPARCL1,NUMB,ARHGEF7,TJP2,WDR1,WASF2,FERMT2,EPB41L3,DIP2A,CYFIP1,SRGAP2,PIP5K1C,PLXNB2,DNM3,APPL1,DBNL,RAB8B,PARD3,JAM3,LRRK2,ACTBL2	46/-	Specific serum up
12_Summary	GO Biological Processes	GO:0044092	negative regulation of molecular function	-14.93155437	-12.28876991	12,60,325,408,567,710,817,831,1475,1845,1893,2048,2168,2316,2621,2776,2876,2950,3071,3315,4043,4067,5524,5525,5859,5899,6059,6093,6714,6906,7076,7078,7453,7532,9370,9446,9475,9908,10527,23376,23603,25927,51035,51291,54899,55031,55914,58533,81494,84313,84959,93974,120892,1981,5515,6774,7529,7531,8440,8655,9353,11137,28964,56288,2539,6188	SERPINA3,ACTB,APCS,ARRB1,B2M,SERPING1,CAMK2D,CAST,CSTA,DUSP3,ECM1,EPHB2,FABP1,FLNA,GAS6,GNAQ,GPX1,GSTP1,NCKAP1L,HSPB1,LRPAP1,LYN,PTPA,PPP2R5A,QARS1,RALB,ABCE1,ROCK1,SRC,SERPINA7,TIMP1,TIMP3,WARS1,YWHAG,ADIPOQ,GSTO1,ROCK2,G3BP2,IPO7,UFL1,CORO1C,CNRIP1,UBXN1,GMIP,PXK,USP47,ERBIN,SNX6,CFHR5,VPS25,UBASH3B,ATP5IF1,LRRK2,EIF4G1,PPP2CA,STAT3,YWHAB,YWHAE,NCK2,DYNLL1,SLIT2,PWP1,GIT1,PARD3,G6PD,RPS3	66/-	Specific serum up
13_Summary	GO Biological Processes	GO:0042060	wound healing	-14.44866609	-11.83070521	60,308,710,1511,2158,2162,2165,2316,2335,2621,2776,2811,2876,3315,5119,5196,5336,5339,6288,6714,7076,7094,7168,7414,7450,10672,57175,80739,81027,83706,84959,116844,196527,375056,2821,3371,4067,4147,4313,5905,231,1314,1535,2048,4830,5567,1000,1475,3384,3385,3655,3956,5499,6091,6093,8404,8832,9124,9414,10611,23396,23654,26999,60626,64072,79633,83700,91624	ACTB,ANXA5,SERPING1,CTSG,F9,F13A1,F13B,FLNA,FN1,GAS6,GNAQ,GP1BA,GPX1,HSPB1,CHMP1A,PF4,PLCG2,PLEC,SAA1,SRC,TIMP1,TLN1,TPM1,VCL,VWF,GNA13,CORO1B,MPIG6B,TUBB1,FERMT3,UBASH3B,LRG1,ANO6,MIA3,GPI,TNC,LYN,MATN2,MMP2,RANGAP1,AKR1B1,COPA,CYBA,EPHB2,NME1,PRKACB,CDH2,CSTA,ICAM2,ICAM3,ITGA6,LGALS1,PPP1CA,ROBO1,ROCK1,SPARCL1,CD84,PDLIM1,TJP2,PDLIM5,PIP5K1C,PLXNB2,CYFIP2,RIC8A,CDH23,FAT4,JAM3,NEXN	68/-	Specific serum up
14_Summary	GO Biological Processes	GO:0040017	positive regulation of locomotion	-14.25636601	-11.66188622	59,102,351,498,506,566,972,2048,2260,2316,2335,2621,2821,3071,3315,3655,4067,4313,4638,5216,5336,5578,5606,6387,6678,6714,6774,8650,8874,9181,9353,9475,9693,10457,10979,10982,11151,23224,55612,55749,57142,65992,81704,83700,83706,196527,284119,375056,889,23603	ACTA2,ADAM10,APP,ATP5F1A,ATP5F1B,AZU1,CD74,EPHB2,FGFR1,FLNA,FN1,GAS6,GPI,NCKAP1L,HSPB1,ITGA6,LYN,MMP2,MYLK,PFN1,PLCG2,PRKCA,MAP2K3,CXCL12,SPARC,SRC,STAT3,NUMB,ARHGEF7,ARHGEF2,SLIT2,ROCK2,RAPGEF2,GPNMB,FERMT2,MAPRE2,CORO1A,SYNE2,FERMT1,CCAR1,RTN4,DDRGK1,DOCK8,JAM3,FERMT3,ANO6,CAVIN1,MIA3,KRIT1,CORO1C	50/-	Specific serum up
15_Summary	GO Biological Processes	GO:0007264	small GTPase-mediated signal transduction	-13.76270934	-11.21169525	29,391,393,395,889,2664,2885,3988,5898,5899,6093,8036,8874,9475,9693,9908,10163,10235,10672,10787,10971,11021,22931,23191,28988,55845,79930,81704,93663,120892	ABR,RHOG,ARHGAP4,ARHGAP6,KRIT1,GDI1,GRB2,LIPA,RALA,RALB,ROCK1,SHOC2,ARHGEF7,ROCK2,RAPGEF2,G3BP2,WASF2,RASGRP2,GNA13,NCKAP1,YWHAQ,RAB35,RAB18,CYFIP1,DBNL,BRK1,DOK3,DOCK8,ARHGAP18,LRRK2	30/-	Specific serum up
16_Summary	GO Biological Processes	GO:0016197	endosomal transport	-13.67356825	-11.14275755	19,1213,1639,2316,4218,5119,5872,6642,6643,7415,8301,9341,10652,10938,11021,11151,23603,26000,30844,30845,30849,50618,51560,51762,54899,56850,58533,84313,85021,112574,160,10097,23230,54832	ABCA1,CLTC,DCTN1,FLNA,RAB8A,CHMP1A,RAB13,SNX1,SNX2,VCP,PICALM,VAMP3,YKT6,EHD1,RAB35,CORO1A,CORO1C,TBC1D10B,EHD4,EHD3,PIK3R4,ITSN2,RAB6B,RAB8B,PXK,GRIPAP1,SNX6,VPS25,REPS1,SNX18,AP2A1,ACTR2,VPS13A,VPS13C	34/-	Specific serum up
17_Summary	GO Biological Processes	GO:0022604	regulation of cell morphogenesis	-13.61054838	-11.09904283	226,391,816,832,1627,2011,2048,2242,2335,3055,5048,6678,6714,7168,8874,9368,9948,10672,10979,11151,23136,23191,23603,23654,29780,50618,57175,93663,116985	ALDOA,RHOG,CAMK2B,CAPZB,DBN1,MARK2,EPHB2,FES,FN1,HCK,PAFAH1B1,SPARC,SRC,TPM1,ARHGEF7,NHERF1,WDR1,GNA13,FERMT2,CORO1A,EPB41L3,CYFIP1,CORO1C,PLXNB2,PARVB,ITSN2,CORO1B,ARHGAP18,ARAP1	29/-	Specific serum up

18_Summary	GO Biological Processes	GO:0051129	negative regulation of cellular component organization	-13.56685276	-11.06468723	393,395,567,829,830,832,1445,1785,2048,2314,2316,2664,2876,3020,4043,4134,5048,5216,5657,6093,6696,6714,7533,8301,8874,9168,9181,9184,9353,9370,9475,9693,10163,11151,11344,22919,23256,23603,26052,26060,50810,51571,54521,57142,57175,57551,57609,83700,116985,120892,1073,9948,11034,55201,136319,2107	ARHGAP4,ARHGAP6,B2M,CAPZA1,CAPZA2,CAPZB,CSK,DNM2,EPHB2,FLII,FLNA,GDI1,GPX1,H3-3A,LRPAP1,MAP4,PAFAH1B1,PFN1,PRTN3,ROCK1,SPP1,SRC,YWHAH,PICALM,ARHGEF7,TMSB10,ARHGEF2,BUB3,SLIT2,ADIPOQ,ROCK2,RAPGEF2,WASF2,CORO1A,TWF2,MAPRE1,SCFD1,CORO1C,DNM3,APPL1,HDGFL3,CYRIB,WDR44,RTN4,CORO1B,TAOK1,DIP2B,JAM3,ARAP1,LRRK2,CFL2,WDR1,DSTN,MAP1S,MTPN,ETF1	56/-	Specific serum up
19_Summary	GO Biological Processes	GO:0072583	clathrin-dependent endocytosis	-13.51303053	-11.02000838	160,163,1173,1175,1211,1212,1213,1759,8301,23396,50618,54874,115548,408,1785,2017,2207,2885,5898,5899,6642,9260,26052,1000,4218,5413,5874,6455,8676,9342,10159,28964,51606	AP2A1,AP2B1,AP2M1,AP2S1,CLTA,CLTB,CLTC,DNM1,PICALM,PIP5K1C,ITSN2,FNBP1L,FCHO2,ARRB1,DNM2,CTTN,FCER1G,GRB2,RALA,RALB,SNX1,PDLIM7,DNM3,CDH2,RAB8A,SEPTIN5,RAB27B,SH3GL1,STX11,SNAP29,ATP6AP2,GIT1,ATP6V1H	33/-	Specific serum up
20_Summary	GO Biological Processes	GO:0045859	regulation of protein kinase activity	-13.43653956	-10.96984636	60,351,566,1116,1845,1991,2011,2048,2260,2621,2776,2950,3315,3320,4067,5515,5571,5606,5859,5899,6091,6188,6714,7295,7453,7532,9368,9370,9693,10006,10527,10979,11140,23603,58533,84313,84959,120892,219771,972,5525,408,824,1981,2181,3071,3263,3326,3655,5007,5336,5510,5524,6093,7415,8440,9181,9475,10457,30844,65992,92597,7965,8452,23181,57551,57609,59,1000,1445,2335,2885,5037,5048,5578,5610,7534,9518,10159,79930	ACTB,APP,AZU1,CHI3L1,DUSP3,ELANE,MARK2,EPHB2,FGFR1,GAS6,GNAQ,GSTP1,HSPB1,HSP90AA1,LYN,PPP2CA,PRKAG1,MAP2K3,QARS1,RALB,ROBO1,RPS3,SRC,TXN,WARS1,YWHAG,NHERF1,ADIPOQ,RAPGEF2,ABI1,IPO7,FERMT2,CDC37,CORO1C,SNX6,VPS25,UBASH3B,LRRK2,CCNY,CD74,PPP2R5A,ARRB1,CAPN2,EIF4G1,ACSL3,NCKAP1L,HPX,HSP90AB1,ITGA6,OSBP,PLCG2,PPP1R7,PTPA,ROCK1,VCP,NCK2,ARHGEF2,ROCK2,GPNMB,EHD4,DDRGK1,MOB1B,AIMP2,CUL3,DIP2A,TAOK1,DIP2B,ACTA2,CDH2,CSK,FN1,GRB2,PEBP1,PAFAH1B1,PRKCA,EIF2AK2,YWHAZ,GDF15,ATP6AP2,DOK3	80/-	Specific serum up
1_Summary	GO Biological Processes	GO:0045861	negative regulation of proteolysis	-4.040342028	-0.086246569	274,358,11345,51534	BIN1,AQP1,GABARAPL2,VTA1	4/-	Both down
2_Summary	GO Biological Processes	GO:0050769	positive regulation of neurogenesis	-3.959479954	-0.086246569	274,4131,5364,358	BIN1,MAP1B,PLXNB1,AQP1	4/-	Both down
3_Summary	GO Biological Processes	GO:0032870	cellular response to hormone stimulus	-3.022633061	0	358,4131,23558	AQP1,MAP1B,WBP2	3/-	Both down
1_Summary	GO Biological Processes	GO:0051604	protein maturation	-15.27038673	-11.09612335	165,444,811,821,2812,2815,2923,3998,4323,5034,7184,9601,10130,10954,10961,23071,51726,56886,64374,81567,5706,10956	AEBP1,ASPH,CALR,CANX,GP1BB,GP9,PDIA3,LMAN1,MMP14,P4HB,HSP90B1,PDIA4,PDIA6,PDIA5,ERP29,ERP44,DNAJB11,UGGT1,SIL1,TXNDC5,PSMC6,OS9	22/-	Both up
2_Summary	GO Biological Processes	GO:0042060	wound healing	-7.791783455	-4.322631274	977,1192,1265,1832,2812,2815,2923,3673,3998,5795,27243,1495,8673,11117,29766,4318,306,6813,8773,6788,1729,4323	CD151,CLIC1,CNN2,DSP,GP1BB,GP9,PDIA3,ITGA2,LMAN1,PTPRJ,CHMP2A,CTNNA1,VAMP8,EMILIN1,TMOD3,MMP9,ANXA3,STXBP2,SNAP23,STK3,DIAPH1,MMP14	22/-	Both up
3_Summary	GO Biological Processes	GO:0097435	supramolecular fiber organization	-7.718743405	-4.322631274	1265,1277,1278,1729,1832,3856,3860,7184,7408,8407,10095,11117,29766,144501,811,2317,29109,3728,165,27243	CNN2,COL1A1,COL1A2,DIAPH1,DSP,KRT8,KRT13,HSP90B1,VASP,TAGLN2,ARPC1B,EMILIN1,TMOD3,KRT80,CALR,FLNB,FHOD1,JUP,AEBP1,CHMP2A	20/-	Both up
4_Summary	GO Biological Processes	GO:0006491	N-glycan processing	-6.506619255	-3.235445861	2530,4124,5589,23193,5373,9761,23071,56886,821,1277,3856,4323,6788,7408	FUT8,MAN2A1,PRKCSH,GANAB,PMM2,MLEC,ERP44,UGGT1,CANX,COL1A1,KRT8,MMP14,STK3,VASP	14/-	Both up
5_Summary	GO Biological Processes	GO:0030198	extracellular matrix organization	-6.292403858	-3.133267697	1277,1278,2022,3673,4318,4323,8751,11117,22795,811,847,10095,1265,3728,967,79073,6817,10257,1495,1729,7184,1967,4969	COL1A1,COL1A2,ENG,ITGA2,MMP9,MMP14,ADAM15,EMILIN1,NID2,CALR,CAT,ARPC1B,CNN2,JUP,CD63,TMEM109,SULT1A1,ABCC4,CTNNA1,DIAPH1,HSP90B1,EIF2B1,OGN	23/-	Both up
6_Summary	GO Biological Processes	GO:0010810	regulation of cell-substrate adhesion	-5.973724612	-2.909572962	811,1277,3728,4323,5034,5795,8751,11117,1265,1495,2022,967,977,3673,6813,8673,27243,306,4318,5175,847,1967,10095,1832,6788,3106,8815	CALR,COL1A1,JUP,MMP14,P4HB,PTPRJ,ADAM15,EMILIN1,CNN2,CTNNA1,ENG,CD63,CD151,ITGA2,STXBP2,VAMP8,CHMP2A,ANXA3,MMP9,PECAM1,CAT,EIF2B1,ARPC1B,DSP,STK3,HLA-B,BANF1	27/-	Both up
7_Summary	GO Biological Processes	GO:0036503	ERAD pathway	-5.96989299	-2.909572962	811,821,5706,7184,10956,56886,4323,5700,5702,5713,27243,151636,5720,5721	CALR,CANX,PSMC6,HSP90B1,OS9,UGGT1,MMP14,PSMC1,PSMC3,PSMD7,CHMP2A,DTX3L,PSME1,PSME2	14/-	Both up
8_Summary	GO Biological Processes	GO:0045862	positive regulation of proteolysis	-5.563858221	-2.590100524	444,4323,5700,5702,5706,5720,5721,10541,55075,4318,7184	ASPH,MMP14,PSMC1,PSMC3,PSMC6,PSME1,PSME2,ANP32B,UACA,MMP9,HSP90B1	11/-	Both up
9_Summary	GO Biological Processes	GO:0032963	collagen metabolic process	-5.533914983	-2.590100524	1277,1278,4318,4323,8751,290,2022,5175,5795,10541,1495,2530,3673,3728,10095,306,5782,8673,27243,6788,7408	COL1A1,COL1A2,MMP9,MMP14,ADAM15,ANPEP,ENG,PECAM1,PTPRJ,ANP32B,CTNNA1,FUT8,ITGA2,JUP,ARPC1B,ANXA3,PTPN12,VAMP8,CHMP2A,STK3,VASP	21/-	Both up
10_Summary	GO Biological Processes	GO:0032940	secretion by cell	-4.772363967	-2.02946435	306,821,2022,6813,8773,9601,10257,10961,51150,8673	ANXA3,CANX,ENG,STXBP2,SNAP23,PDIA4,ABCC4,ERP29,SDF4,VAMP8	10/-	Both up
11_Summary	GO Biological Processes	GO:0098761	cellular response to interleukin-7	-4.660871167	-1.97796948	2923,4046,5034,1277,2317,3856,5795	PDIA3,LSP1,P4HB,COL1A1,FLNB,KRT8,PTPRJ	7/-	Both up

12_Summary	GO Biological Processes	GO:0070482	response to oxygen levels	-4.586056232	-1.934860418	847,1277,3673,4323,5034,5351,7184,8751,444,165	CAT,COL1A1,ITGA2,MMP14,P4HB,PLOD1,HSP90B1,ADAM15,ASPH,AEBP1	10/-	Both up
13_Summary	GO Biological Processes	GO:0090136	epithelial cell-cell adhesion	-4.577644882	-1.934860418	1495,1832,3728,1277,1278,2317,3673,144501,1192,2923,5175,29766,847,8751,51150,6780,151636	CTNNA1,DSP,JUP,COL1A1,COL1A2,FLNB,ITGA2,KRT80,CLIC1,PDIA3,PECAM1,TMOD3,CAT,ADAM15,SDF4,STAU1,DTX3L	17/-	Both up
14_Summary	GO Biological Processes	GO:0016032	viral process	-4.543611344	-1.913416007	290,821,2530,3673,4124,8673,27243,5702,847,4357,8801,9123	ANPEP,CANX,FUT8,ITGA2,MAN2A1,VAMP8,CHMP2A,PSMC3,CAT,MPST,SUCLG2,SLC16A3	12/-	Both up
15_Summary	GO Biological Processes	GO:0002474	antigen processing and presentation of peptide antigen via MHC class I	-3.541591486	-1.193402908	811,2923,3106,5720	CALR,PDIA3,HLA-B,PSME1	4/-	Both up
16_Summary	GO Biological Processes	GO:0032965	regulation of collagen biosynthetic process	-3.506967195	-1.165212726	2022,3673,11117,306,3728	ENG,ITGA2,EMILIN1,ANXA3,JUP	5/-	Both up
17_Summary	GO Biological Processes	GO:0097191	extrinsic apoptotic signaling pathway	-3.199522569	-0.949538474	1495,2923,3856,6788,4318,151636,8751,5034,10961,79073,11117	CTNNA1,PDIA3,KRT8,STK3,MMP9,DTX3L,ADAM15,P4HB,ERP29,TMEM109,EMILIN1	11/-	Both up
18_Summary	GO Biological Processes	GO:0006998	nuclear envelope organization	-2.987532759	-0.790992983	4001,8815,27243,6813,8673,8773,29109,55153,3998	LMNB1,BANF1,CHMP2A,STXBP2,VAMP8,SNAP23,FHOD1,SDAD1,LMAN1	9/-	Both up
19_Summary	GO Biological Processes	GO:0001889	liver development	-2.921314645	-0.729322497	3673,4124,4357,5589	ITGA2,MAN2A1,MPST,PRKCSH	4/-	Both up
20_Summary	GO Biological Processes	GO:0042058	regulation of epidermal growth factor receptor signaling pathway	-2.803107428	-0.647951284	4318,5782,5795,2022,5175,10111,10961,1265,8673,10956,5034	MMP9,PTPN12,PTPRJ,ENG,PECAM1,RAD50,ERP29,CNN2,VAMP8,OS9,P4HB	11/-	Both up

supplementary table 4 List of Specific Proteins with Identified Counts in BrM and BrM-NAT.				
**Gene Name**	**Uniprot**	**Identified count in BrM**	**Identified count in BrM-NAT**	**Group**
IGHV3-38	A0A0C4DH36	2	5	BrM-NAT-specific protein
CRPPA	A4D126	2	7	BrM-NAT-specific protein
CARNS1	A5YM72	2	7	BrM-NAT-specific protein
PLEKHD1	A6NEE1	0	5	BrM-NAT-specific protein
WIPF3	A6NGB9	2	5	BrM-NAT-specific protein
TMEM88B	A6NKF7	0	5	BrM-NAT-specific protein
GRM8	O00222	1	5	BrM-NAT-specific protein
DLGAP1	O14490	2	7	BrM-NAT-specific protein
FCHO1	O14526	2	6	BrM-NAT-specific protein
KIF3C	O14782	2	5	BrM-NAT-specific protein
FRS3	O43559	1	5	BrM-NAT-specific protein
CYTH3	O43739	2	6	BrM-NAT-specific protein
ADGRB2	O60241	2	6	BrM-NAT-specific protein
PRKN	O60260	2	7	BrM-NAT-specific protein
USP12	O75317	2	7	BrM-NAT-specific protein
DNAJB5	O75953	2	5	BrM-NAT-specific protein
DUSP14	O95147	0	5	BrM-NAT-specific protein
CSPG5	O95196	2	5	BrM-NAT-specific protein
SLC25A27	O95847	2	5	BrM-NAT-specific protein
DLGAP3	O95886	2	5	BrM-NAT-specific protein
CALCA	P01258	1	5	BrM-NAT-specific protein
IGHV1-46	P01743	1	5	BrM-NAT-specific protein
MYL3	P08590	1	5	BrM-NAT-specific protein
RLBP1	P12271	2	6	BrM-NAT-specific protein
GABRG2	P18507	2	5	BrM-NAT-specific protein
CNR1	P21554	1	5	BrM-NAT-specific protein
FGFR2	P21802	1	5	BrM-NAT-specific protein
KCNA4	P22459	1	5	BrM-NAT-specific protein
EVI2A	P22794	1	5	BrM-NAT-specific protein
GPT	P24298	2	7	BrM-NAT-specific protein
EDNRB	P24530	0	5	BrM-NAT-specific protein
PDE4A	P27815	2	5	BrM-NAT-specific protein
GABRB3	P28472	2	6	BrM-NAT-specific protein
MAPK4	P31152	1	7	BrM-NAT-specific protein
HIVEP2	P31629	2	5	BrM-NAT-specific protein
SCN1A	P35498	2	6	BrM-NAT-specific protein

BRCA1	P38398	1	5	BrM-NAT-specific protein
ADCYAP1R1	P41586	2	6	BrM-NAT-specific protein
CDKN1B	P46527	2	6	BrM-NAT-specific protein
GABRA2	P47869	2	5	BrM-NAT-specific protein
SLC6A8	P48029	0	5	BrM-NAT-specific protein
KCNJ6	P48051	2	5	BrM-NAT-specific protein
CRYBB1	P53674	2	5	BrM-NAT-specific protein
MAPK12	P53778	1	6	BrM-NAT-specific protein
MTTP	P55157	2	5	BrM-NAT-specific protein
GFRA1	P56159	1	7	BrM-NAT-specific protein
SOX8	P57073	2	7	BrM-NAT-specific protein
SLC8A3	P57103	1	5	BrM-NAT-specific protein
PCBP4	P57723	2	5	BrM-NAT-specific protein
SNX16	P57768	2	6	BrM-NAT-specific protein
PKIA	P61925	2	5	BrM-NAT-specific protein
GPC5	P78333	0	7	BrM-NAT-specific protein
KRT76	Q01546	1	6	BrM-NAT-specific protein
DMWD	Q09019	2	7	BrM-NAT-specific protein
GRIK3	Q13003	1	6	BrM-NAT-specific protein
GRIN2B	Q13224	2	5	BrM-NAT-specific protein
ART3	Q13508	1	6	BrM-NAT-specific protein
PPP1R1A	Q13522	0	5	BrM-NAT-specific protein
KCNC3	Q14003	1	5	BrM-NAT-specific protein
KCNAB1	Q14722	2	7	BrM-NAT-specific protein
JOSD1	Q15040	1	5	BrM-NAT-specific protein
RYR3	Q15413	1	5	BrM-NAT-specific protein
INPP5J	Q15735	2	6	BrM-NAT-specific protein
SOWAHA	Q2M3V2	2	5	BrM-NAT-specific protein
GRAMD1B	Q3KR37	2	6	BrM-NAT-specific protein
SLC39A12	Q504Y0	1	7	BrM-NAT-specific protein
PPM1J	Q5JR12	1	6	BrM-NAT-specific protein
GJC2	Q5T442	0	5	BrM-NAT-specific protein
KPRP	Q5T749	1	5	BrM-NAT-specific protein
PIP5KL1	Q5T9C9	1	5	BrM-NAT-specific protein
ASPHD1	Q5U4P2	1	5	BrM-NAT-specific protein
LRCH2	Q5VUJ6	2	6	BrM-NAT-specific protein
JAKMIP3	Q5VZ66	2	7	BrM-NAT-specific protein
MCTP1	Q6DN14	2	6	BrM-NAT-specific protein

TCEAL6	Q6IPX3	1	6	BrM-NAT-specific protein
RGS7BP	Q6MZT1	2	5	BrM-NAT-specific protein
SLC18B1	Q6NT16	2	5	BrM-NAT-specific protein
CHADL	Q6NUI6	1	5	BrM-NAT-specific protein
FBXO42	Q6P3S6	1	5	BrM-NAT-specific protein
LRIG3	Q6UXM1	2	6	BrM-NAT-specific protein
NSMF	Q6X4W1	2	7	BrM-NAT-specific protein
GLDN	Q6ZMI3	2	5	BrM-NAT-specific protein
AATK	Q6ZMQ8	2	7	BrM-NAT-specific protein
TRIM72	Q6ZMU5	1	6	BrM-NAT-specific protein
UNC5A	Q6ZN44	2	5	BrM-NAT-specific protein
TPGS1	Q6ZTW0	1	6	BrM-NAT-specific protein
PNPLA7	Q6ZV29	0	6	BrM-NAT-specific protein
NYAP1	Q6ZVC0	0	5	BrM-NAT-specific protein
BRINP3	Q76B58	0	5	BrM-NAT-specific protein
PDZD4	Q76G19	1	5	BrM-NAT-specific protein
HECW1	Q76N89	2	6	BrM-NAT-specific protein
VASH1	Q7L8A9	2	5	BrM-NAT-specific protein
PRICKLE2	Q7Z3G6	0	5	BrM-NAT-specific protein
LHFPL4	Q7Z7J7	2	5	BrM-NAT-specific protein
KCNMB4	Q86W47	0	5	BrM-NAT-specific protein
TMEM25	Q86YD3	2	5	BrM-NAT-specific protein
TDRP	Q86YL5	2	5	BrM-NAT-specific protein
PRAG1	Q86YV5	2	5	BrM-NAT-specific protein
TMC8	Q8IU68	0	5	BrM-NAT-specific protein
RIMKLA	Q8IXN7	0	6	BrM-NAT-specific protein
SLC35F3	Q8IY50	2	5	BrM-NAT-specific protein
EFCAB13	Q8IY85	0	5	BrM-NAT-specific protein
TMEFF1	Q8IYR6	1	6	BrM-NAT-specific protein
ZDHHC14	Q8IZN3	2	5	BrM-NAT-specific protein
ANKRD35	Q8N283	2	7	BrM-NAT-specific protein
NLGN1	Q8N2Q7	2	6	BrM-NAT-specific protein
CLIP4	Q8N3C7	1	5	BrM-NAT-specific protein
PPM1K	Q8N3J5	1	5	BrM-NAT-specific protein
GALNT15	Q8N3T1	1	6	BrM-NAT-specific protein
SFRP1	Q8N474	2	5	BrM-NAT-specific protein
PRR18	Q8N4B5	2	6	BrM-NAT-specific protein
TMEM151A	Q8N4L1	2	5	BrM-NAT-specific protein

JMY	Q8N9B5	2	6	BrM-NAT-specific protein
TMEM145	Q8NBT3	1	5	BrM-NAT-specific protein
RELL2	Q8NC24	1	6	BrM-NAT-specific protein
NETO2	Q8NC67	2	5	BrM-NAT-specific protein
KCTD6	Q8NC69	0	6	BrM-NAT-specific protein
SLAIN1	Q8ND83	1	6	BrM-NAT-specific protein
CPEB3	Q8NE35	1	5	BrM-NAT-specific protein
DENND6B	Q8NEG7	0	5	BrM-NAT-specific protein
MDGA1	Q8NFP4	2	5	BrM-NAT-specific protein
CD300LF	Q8TDQ1	2	5	BrM-NAT-specific protein
OTUD7A	Q8TE49	2	7	BrM-NAT-specific protein
SLC22A17	Q8WUG5	2	5	BrM-NAT-specific protein
CCNB3	Q8WWL7	1	6	BrM-NAT-specific protein
JPH3	Q8WXH2	0	5	BrM-NAT-specific protein
SPOCK2	Q92563	2	7	BrM-NAT-specific protein
DPF3	Q92784	0	5	BrM-NAT-specific protein
PROX1	Q92786	2	6	BrM-NAT-specific protein
SLC35B4	Q969S0	1	5	BrM-NAT-specific protein
UBE2W	Q96B02	2	5	BrM-NAT-specific protein
DACT3	Q96B18	2	5	BrM-NAT-specific protein
NACC2	Q96BF6	2	6	BrM-NAT-specific protein
NTNG2	Q96CW9	2	6	BrM-NAT-specific protein
NUDT11	Q96G61	1	5	BrM-NAT-specific protein
PHACTR3	Q96KR7	2	7	BrM-NAT-specific protein
LURAP1	Q96LR2	2	6	BrM-NAT-specific protein
TLCD4	Q96MV1	2	5	BrM-NAT-specific protein
JAKMIP1	Q96N16	2	7	BrM-NAT-specific protein
LRFN5	Q96NI6	2	5	BrM-NAT-specific protein
AIFM3	Q96NN9	2	6	BrM-NAT-specific protein
NAP1L5	Q96NT1	1	6	BrM-NAT-specific protein
CSMD1	Q96PZ7	1	5	BrM-NAT-specific protein
BBS4	Q96RK4	2	7	BrM-NAT-specific protein
GAD1	Q99259	2	6	BrM-NAT-specific protein
GAS2L1	Q99501	2	7	BrM-NAT-specific protein
SPOCK3	Q9BQ16	2	6	BrM-NAT-specific protein
SYT3	Q9BQG1	2	5	BrM-NAT-specific protein
PGAP4	Q9BRR3	1	5	BrM-NAT-specific protein
LARP6	Q9BRS8	2	7	BrM-NAT-specific protein

ZNF692	Q9BU19	2	6	BrM-NAT-specific protein
BEGAIN	Q9BUH8	2	6	BrM-NAT-specific protein
DUSP26	Q9BV47	0	5	BrM-NAT-specific protein
NT5C1A	Q9BXI3	2	6	BrM-NAT-specific protein
C1QTNF4	Q9BXJ3	2	6	BrM-NAT-specific protein
ANKRD30B	Q9BXX2	2	5	BrM-NAT-specific protein
CNTNAP3	Q9BZ76	0	6	BrM-NAT-specific protein
NMNAT2	Q9BZQ4	1	6	BrM-NAT-specific protein
KLHL4	Q9C0H6	2	5	BrM-NAT-specific protein
STMN4	Q9H169	2	6	BrM-NAT-specific protein
TLNRD1	Q9H1K6	1	5	BrM-NAT-specific protein
SYT4	Q9H2B2	1	5	BrM-NAT-specific protein
KCNN2	Q9H2S1	1	5	BrM-NAT-specific protein
CDIP1	Q9H305	2	5	BrM-NAT-specific protein
TMEM38A	Q9H6F2	0	6	BrM-NAT-specific protein
MTHFD2L	Q9H903	1	5	BrM-NAT-specific protein
HHATL	Q9HCP6	1	5	BrM-NAT-specific protein
PARD6A	Q9NPB6	1	5	BrM-NAT-specific protein
SNRK	Q9NRH2	2	5	BrM-NAT-specific protein
CNNM1	Q9NRU3	2	6	BrM-NAT-specific protein
TNFRSF19	Q9NS68	1	5	BrM-NAT-specific protein
KCND1	Q9NSA2	1	5	BrM-NAT-specific protein
NMRK1	Q9NWW6	2	7	BrM-NAT-specific protein
SYBU	Q9NX95	2	5	BrM-NAT-specific protein
ELOVL2	Q9NXB9	1	5	BrM-NAT-specific protein
WSB2	Q9NYS7	2	5	BrM-NAT-specific protein
NLGN3	Q9NZ94	2	7	BrM-NAT-specific protein
IL1RAPL1	Q9NZN1	0	5	BrM-NAT-specific protein
AKAP7	Q9P0M2	2	5	BrM-NAT-specific protein
HECA	Q9UBI9	2	5	BrM-NAT-specific protein
ARPP21	Q9UBL0	2	6	BrM-NAT-specific protein
RGS17	Q9UGC6	2	6	BrM-NAT-specific protein
KLHL3	Q9UH77	1	6	BrM-NAT-specific protein
ZDHHC2	Q9UIJ5	2	5	BrM-NAT-specific protein
KCND3	Q9UK17	2	5	BrM-NAT-specific protein
TRHDE	Q9UKU6	2	5	BrM-NAT-specific protein
CDH7	Q9ULB5	2	5	BrM-NAT-specific protein
LRFN2	Q9ULH4	2	5	BrM-NAT-specific protein

PLEKHH1	Q9ULM0	2	6	BrM-NAT-specific protein
CA14	Q9ULX7	1	7	BrM-NAT-specific protein
SUFU	Q9UMX1	2	5	BrM-NAT-specific protein
SAMD4A	Q9UPU9	2	7	BrM-NAT-specific protein
THSD7A	Q9UPZ6	0	5	BrM-NAT-specific protein
RIMS2	Q9UQ26	2	6	BrM-NAT-specific protein
SCN8A	Q9UQD0	2	6	BrM-NAT-specific protein
MAPK8IP1	Q9UQF2	1	7	BrM-NAT-specific protein
CSDC2	Q9Y534	2	6	BrM-NAT-specific protein
PCDHGB7	Q9Y5F8	1	5	BrM-NAT-specific protein
PHLDA3	Q9Y5J5	2	6	BrM-NAT-specific protein
SNX10	Q9Y5X0	1	5	BrM-NAT-specific protein
BICDL2	A1A5D9	5	0	BrM-specific protein
PXDNL	A1KZ92	5	2	BrM-specific protein
MEX3A	A1L020	5	0	BrM-specific protein
ZNF316	A6NFI3	5	2	BrM-specific protein
METTL15	A6NJ78	5	0	BrM-specific protein
LRRC37A2	A6NM11	6	0	BrM-specific protein
HIDE1	A8MVS5	5	2	BrM-specific protein
SMIM22	K7EJ46	5	2	BrM-specific protein
RFXAP	O00287	5	2	BrM-specific protein
IFRD1	O00458	6	1	BrM-specific protein
ZNF593	O00488	5	0	BrM-specific protein
LAD1	O00515	5	2	BrM-specific protein
FCN1	O00602	6	2	BrM-specific protein
CCN1	O00622	5	1	BrM-specific protein
UBE2C	O00762	6	0	BrM-specific protein
CLDN4	O14493	5	1	BrM-specific protein
RGPD8	O14715	7	1	BrM-specific protein
NDC80	O14777	5	1	BrM-specific protein
ZBTB7B	O15156	5	1	BrM-specific protein
LEPROT	O15243	5	0	BrM-specific protein
TMPRSS2	O15393	5	1	BrM-specific protein
POLR1G	O15446	7	2	BrM-specific protein
SPINT1	O43278	6	2	BrM-specific protein
BCL2L11	O43521	5	0	BrM-specific protein
CA12	O43570	5	0	BrM-specific protein
TSPAN6	O43657	5	2	BrM-specific protein

MPZL2	O60487	5	0	BrM-specific protein
B4GALT3	O60512	5	0	BrM-specific protein
B4GALT4	O60513	6	1	BrM-specific protein
SRPX2	O60687	6	2	BrM-specific protein
HUS1	O60921	5	2	BrM-specific protein
ZNF623	O75123	5	1	BrM-specific protein
DUSP11	O75319	5	0	BrM-specific protein
HMMR	O75330	5	0	BrM-specific protein
SAP30	O75446	5	1	BrM-specific protein
TADA3	O75528	5	2	BrM-specific protein
PGLYRP1	O75594	5	2	BrM-specific protein
SURF6	O75683	5	2	BrM-specific protein
RPP40	O75818	5	2	BrM-specific protein
ANXA9	O76027	5	1	BrM-specific protein
MICAL2	O94851	6	2	BrM-specific protein
TJP3	O95049	5	1	BrM-specific protein
S1PR2	O95136	5	0	BrM-specific protein
MBD4	O95243	5	2	BrM-specific protein
PAPLN	O95428	6	2	BrM-specific protein
CLIC3	O95833	6	2	BrM-specific protein
ECD	O95905	5	2	BrM-specific protein
EFEMP2	O95967	6	2	BrM-specific protein
DHFR	P00374	6	1	BrM-specific protein
PLAU	P00749	5	2	BrM-specific protein
TYMS	P04818	5	0	BrM-specific protein
SP1	P08047	5	2	BrM-specific protein
MME	P08473	6	2	BrM-specific protein
POU2F2	P09086	6	0	BrM-specific protein
MMP7	P09237	6	2	BrM-specific protein
CSF1	P09603	5	2	BrM-specific protein
EPX	P11678	5	0	BrM-specific protein
GZMA	P12544	6	1	BrM-specific protein
MT1G	P13640	5	2	BrM-specific protein
ACP5	P13686	7	2	BrM-specific protein
PRF1	P14222	5	2	BrM-specific protein
IRF2	P14316	5	0	BrM-specific protein
TRIM27	P14373	5	1	BrM-specific protein
CCNB1	P14635	6	0	BrM-specific protein

POU2F1	P14859	5	0	BrM-specific protein
ACP3	P15309	7	1	BrM-specific protein
EPCAM	P16422	6	2	BrM-specific protein
BPI	P17213	6	2	BrM-specific protein
IL1RN	P18510	6	1	BrM-specific protein
ITGB6	P18564	5	1	BrM-specific protein
LIG1	P18858	5	2	BrM-specific protein
FUT3	P21217	6	1	BrM-specific protein
C5AR1	P21730	5	2	BrM-specific protein
TGM1	P22735	5	2	BrM-specific protein
MMP8	P22894	6	2	BrM-specific protein
ITGB7	P26010	5	0	BrM-specific protein
COL8A1	P27658	5	2	BrM-specific protein
ARID4A	P29374	5	2	BrM-specific protein
TM4SF1	P30408	5	1	BrM-specific protein
FMO3	P31513	5	1	BrM-specific protein
LTBR	P36941	6	1	BrM-specific protein
FDFT1	P37268	5	1	BrM-specific protein
MMP12	P39900	7	0	BrM-specific protein
PPIC	P45877	6	2	BrM-specific protein
PRIM1	P49642	5	1	BrM-specific protein
CASP4	P49662	7	2	BrM-specific protein
CLK1	P49759	5	2	BrM-specific protein
SLC26A2	P50443	5	2	BrM-specific protein
ARSL	P51690	6	2	BrM-specific protein
LIMK2	P53671	6	2	BrM-specific protein
BLM	P54132	5	1	BrM-specific protein
INHBC	P55103	6	2	BrM-specific protein
ELL	P55199	5	2	BrM-specific protein
FOXA1	P55317	5	0	BrM-specific protein
RWDD2B	P57060	5	2	BrM-specific protein
NLRP11	P59045	6	2	BrM-specific protein
TMEM258	P61165	5	2	BrM-specific protein
ADAM8	P78325	6	1	BrM-specific protein
OLR1	P78380	5	1	BrM-specific protein
SLC35A2	P78381	5	1	BrM-specific protein
SLC35A1	P78382	5	0	BrM-specific protein
ELF3	P78545	5	0	BrM-specific protein

FAM3A	P98173	5	1	BrM-specific protein
RUNX1	Q01196	7	1	BrM-specific protein
RELB	Q01201	6	2	BrM-specific protein
TFAP4	Q01664	5	2	BrM-specific protein
ROR2	Q01974	5	1	BrM-specific protein
H1-1	Q02539	6	2	BrM-specific protein
RASSF7	Q02833	5	0	BrM-specific protein
KMT2A	Q03164	6	2	BrM-specific protein
CENPC	Q03188	7	1	BrM-specific protein
TLE3	Q04726	7	2	BrM-specific protein
DPT	Q07507	5	2	BrM-specific protein
MCL1	Q07820	6	2	BrM-specific protein
IL10RB	Q08334	7	2	BrM-specific protein
CYP4F3	Q08477	5	2	BrM-specific protein
SIGLEC14	Q08ET2	5	1	BrM-specific protein
MGAT3	Q09327	6	2	BrM-specific protein
ST3GAL1	Q11201	6	1	BrM-specific protein
TMEM115	Q12893	5	1	BrM-specific protein
PRDM2	Q13029	5	2	BrM-specific protein
GPS2	Q13227	5	1	BrM-specific protein
MAD2L1	Q13257	5	1	BrM-specific protein
SEMA3F	Q13275	5	2	BrM-specific protein
CLN3	Q13286	5	1	BrM-specific protein
TNK1	Q13470	5	2	BrM-specific protein
PKD2	Q13563	6	2	BrM-specific protein
GTF2H2	Q13888	5	1	BrM-specific protein
EBI3	Q14213	6	2	BrM-specific protein
SLBP	Q14493	5	2	BrM-specific protein
NEDD9	Q14511	5	1	BrM-specific protein
SEMA3A	Q14563	5	1	BrM-specific protein
DSC3	Q14574	6	0	BrM-specific protein
PTPRCAP	Q14761	6	2	BrM-specific protein
LTBP2	Q14767	5	1	BrM-specific protein
KIF22	Q14807	5	0	BrM-specific protein
KRT72	Q14CN4	5	0	BrM-specific protein
POLD3	Q15054	5	1	BrM-specific protein
MTFR1	Q15390	6	1	BrM-specific protein
TRIP13	Q15645	6	2	BrM-specific protein

VPS72	Q15906	5	2	BrM-specific protein
EXT1	Q16394	5	1	BrM-specific protein
SNRNP35	Q16560	5	0	BrM-specific protein
CA9	Q16790	5	2	BrM-specific protein
UPP1	Q16831	5	1	BrM-specific protein
KNOP1	Q1ED39	5	0	BrM-specific protein
KRT24	Q2M2I5	5	2	BrM-specific protein
EXOC3L2	Q2M3D2	5	2	BrM-specific protein
INAVA	Q3KP66	5	0	BrM-specific protein
ALKBH6	Q3KRA9	6	2	BrM-specific protein
MRPL51	Q4U2R6	5	2	BrM-specific protein
MPPE1	Q53F39	6	2	BrM-specific protein
FASTKD1	Q53R41	5	2	BrM-specific protein
TMEM128	Q5BJH2	5	1	BrM-specific protein
ALG10	Q5BKT4	5	1	BrM-specific protein
DDX60L	Q5H9U9	6	2	BrM-specific protein
RPS26P11	Q5JNZ5	5	2	BrM-specific protein
FGD3	Q5JSP0	6	2	BrM-specific protein
MANEA	Q5SRI9	5	2	BrM-specific protein
RARS2	Q5T160	5	1	BrM-specific protein
GPATCH4	Q5T3I0	7	1	BrM-specific protein
CERCAM	Q5T4B2	5	2	BrM-specific protein
BEND3	Q5T5X7	5	0	BrM-specific protein
ZNF648	Q5T619	5	2	BrM-specific protein
MRPL2	Q5T653	5	2	BrM-specific protein
UBAP2	Q5T6F2	5	2	BrM-specific protein
RNASEH2B	Q5TBB1	5	2	BrM-specific protein
TMEM164	Q5U3C3	5	1	BrM-specific protein
ARHGEF16	Q5VV41	5	2	BrM-specific protein
ZNF691	Q5VV52	5	0	BrM-specific protein
LARP1B	Q659C4	5	2	BrM-specific protein
OLFML2B	Q68BL8	5	1	BrM-specific protein
HAUS3	Q68CZ6	5	1	BrM-specific protein
SPTY2D1	Q68D10	6	2	BrM-specific protein
MCTP2	Q6DN12	5	0	BrM-specific protein
SIGIRR	Q6IA17	6	1	BrM-specific protein
SNRNP48	Q6IEG0	5	2	BrM-specific protein
NAA16	Q6N069	5	2	BrM-specific protein

ESRP1	Q6NXG1	5	2	BrM-specific protein
KLC3	Q6P597	5	0	BrM-specific protein
RPL22L1	Q6P5R6	6	2	BrM-specific protein
TXNDC11	Q6PKC3	6	1	BrM-specific protein
APOA5	Q6Q788	7	2	BrM-specific protein
ATRAID	Q6UW56	6	2	BrM-specific protein
POGLUT2	Q6UW63	6	2	BrM-specific protein
B3GNT9	Q6UX72	5	0	BrM-specific protein
ADAMTSL4	Q6UY14	6	2	BrM-specific protein
MACC1	Q6ZN28	5	2	BrM-specific protein
RBPMS2	Q6ZRY4	5	2	BrM-specific protein
UBN2	Q6ZU65	5	1	BrM-specific protein
NOL8	Q76FK4	5	2	BrM-specific protein
ASXL2	Q76L83	5	1	BrM-specific protein
TMC4	Q7Z404	5	0	BrM-specific protein
LRP10	Q7Z4F1	6	2	BrM-specific protein
PODN	Q7Z5L7	5	2	BrM-specific protein
FGD2	Q7Z6J4	5	2	BrM-specific protein
B3GNT8	Q7Z7M8	5	1	BrM-specific protein
ABCA12	Q86UK0	5	2	BrM-specific protein
DTX2	Q86UW9	5	1	BrM-specific protein
NAA40	Q86UY6	6	2	BrM-specific protein
IQGAP3	Q86VI3	5	2	BrM-specific protein
CHSY1	Q86X52	5	0	BrM-specific protein
ERICH1	Q86X53	6	1	BrM-specific protein
NCAPG2	Q86XI2	5	1	BrM-specific protein
PDSS2	Q86YH6	5	1	BrM-specific protein
TREML1	Q86YW5	6	2	BrM-specific protein
ZDHHC13	Q8IUH4	5	2	BrM-specific protein
APOBEC3F	Q8IUX4	5	1	BrM-specific protein
ERI1	Q8IV48	6	2	BrM-specific protein
P3H2	Q8IVL5	6	1	BrM-specific protein
ADGRA3	Q8IWK6	5	2	BrM-specific protein
DHX40	Q8IX18	5	2	BrM-specific protein
SKA3	Q8IX90	5	2	BrM-specific protein
GALNT12	Q8IXK2	6	1	BrM-specific protein
SYTL1	Q8IYJ3	6	2	BrM-specific protein
SYS1	Q8N2H4	6	0	BrM-specific protein

CLASRP	Q8N2M8	5	2	BrM-specific protein
CARNMT1	Q8N4J0	5	1	BrM-specific protein
MREG	Q8N565	5	2	BrM-specific protein
CERS5	Q8N5B7	5	2	BrM-specific protein
CALHM5	Q8N5C1	6	2	BrM-specific protein
MSL3	Q8N5Y2	6	2	BrM-specific protein
SLC15A4	Q8N697	7	2	BrM-specific protein
MYCT1	Q8N699	5	2	BrM-specific protein
CSGALNACT2	Q8N6G5	5	0	BrM-specific protein
SIRT6	Q8N6T7	6	2	BrM-specific protein
LRRC17	Q8N6Y2	5	1	BrM-specific protein
CGAS	Q8N884	5	2	BrM-specific protein
CEP112	Q8N8E3	5	1	BrM-specific protein
SLC43A3	Q8NBI5	5	2	BrM-specific protein
PCSK9	Q8NBP7	6	1	BrM-specific protein
CHST14	Q8NCH0	5	2	BrM-specific protein
SRFBP1	Q8NEF9	5	2	BrM-specific protein
SLC30A7	Q8NEW0	5	2	BrM-specific protein
KMT2C	Q8NEZ4	6	2	BrM-specific protein
GPRC5A	Q8NFJ5	5	0	BrM-specific protein
COL22A1	Q8NFW1	5	2	BrM-specific protein
HELB	Q8NG08	5	1	BrM-specific protein
DDX55	Q8NHQ9	5	2	BrM-specific protein
RIN3	Q8TB24	5	2	BrM-specific protein
SHKBP1	Q8TBC3	5	1	BrM-specific protein
EPS8L1	Q8TE68	5	2	BrM-specific protein
EOLA1	Q8TE69	5	0	BrM-specific protein
SNED1	Q8TER0	5	2	BrM-specific protein
MZB1	Q8WU39	5	2	BrM-specific protein
TSKU	Q8WUA8	7	2	BrM-specific protein
BLNK	Q8WV28	5	2	BrM-specific protein
POF1B	Q8WVV4	5	0	BrM-specific protein
GLMP	Q8WWB7	6	1	BrM-specific protein
RIN2	Q8WYP3	5	2	BrM-specific protein
SMPDL3A	Q92484	5	2	BrM-specific protein
ALG3	Q92685	6	1	BrM-specific protein
P3H4	Q92791	6	1	BrM-specific protein
CASP10	Q92851	7	2	BrM-specific protein

CD101	Q93033	5	2	BrM-specific protein
CCDC102A	Q96A19	6	1	BrM-specific protein
COL26A1	Q96A83	5	2	BrM-specific protein
PNPLA2	Q96AD5	5	2	BrM-specific protein
TMEM45B	Q96B21	5	0	BrM-specific protein
APH1A	Q96BI3	5	2	BrM-specific protein
TADA1	Q96BN2	6	2	BrM-specific protein
ZNF524	Q96C55	5	2	BrM-specific protein
CTHRC1	Q96CG8	6	1	BrM-specific protein
HAUS1	Q96CS2	5	2	BrM-specific protein
SLC7A6OS	Q96CW6	5	2	BrM-specific protein
ORAI1	Q96D31	5	1	BrM-specific protein
COQ8B	Q96D53	5	2	BrM-specific protein
MTFMT	Q96DP5	5	2	BrM-specific protein
ASB9	Q96DX5	5	0	BrM-specific protein
CCDC126	Q96EE4	6	0	BrM-specific protein
SAAL1	Q96ER3	5	2	BrM-specific protein
RRP36	Q96EU6	5	2	BrM-specific protein
TMA16	Q96EY4	6	2	BrM-specific protein
MCRS1	Q96EZ8	5	2	BrM-specific protein
IL17RA	Q96F46	6	2	BrM-specific protein
WDR89	Q96FK6	5	2	BrM-specific protein
LTV1	Q96GA3	5	2	BrM-specific protein
AURKB	Q96GD4	5	0	BrM-specific protein
ZC3HAV1L	Q96H79	5	2	BrM-specific protein
LCOR	Q96JN0	5	0	BrM-specific protein
PBK	Q96KB5	5	2	BrM-specific protein
B3GALT6	Q96L58	5	1	BrM-specific protein
NSMCE2	Q96MF7	5	1	BrM-specific protein
NSMCE3	Q96MG7	6	2	BrM-specific protein
FAM20A	Q96MK3	6	2	BrM-specific protein
HS6ST2	Q96MM7	6	1	BrM-specific protein
SFXN2	Q96NB2	5	2	BrM-specific protein
ZNF830	Q96NB3	6	2	BrM-specific protein
ZMAT2	Q96NC0	5	2	BrM-specific protein
NECTIN4	Q96NY8	5	1	BrM-specific protein
ADGRA2	Q96PE1	5	1	BrM-specific protein
USP28	Q96RU2	5	2	BrM-specific protein

UHRF1	Q96T88	6	0	BrM-specific protein
EYA3	Q99504	5	2	BrM-specific protein
MMP19	Q99542	5	0	BrM-specific protein
MPHOSPH6	Q99547	5	2	BrM-specific protein
DOK1	Q99704	6	2	BrM-specific protein
ATF6B	Q99941	5	1	BrM-specific protein
OMD	Q99983	5	0	BrM-specific protein
NCAPG	Q9BPX3	6	1	BrM-specific protein
PAIP2	Q9BPZ3	5	2	BrM-specific protein
ANTKMT	Q9BQD7	5	1	BrM-specific protein
LPIN3	Q9BQK8	6	1	BrM-specific protein
MGME1	Q9BQP7	6	2	BrM-specific protein
BUD13	Q9BRD0	5	2	BrM-specific protein
GINS4	Q9BRT9	5	0	BrM-specific protein
GINS3	Q9BRX5	6	1	BrM-specific protein
DESI2	Q9BSY9	6	2	BrM-specific protein
RAMAC	Q9BTL3	5	2	BrM-specific protein
CRB3	Q9BUF7	5	1	BrM-specific protein
KIFC1	Q9BW19	7	2	BrM-specific protein
STRA6	Q9BX79	6	2	BrM-specific protein
PLVAP	Q9BX97	6	2	BrM-specific protein
ASPN	Q9BXN1	6	2	BrM-specific protein
FANCD2	Q9BXW9	7	1	BrM-specific protein
TMPRSS13	Q9BYE2	5	2	BrM-specific protein
KRTAP4-3	Q9BYR4	5	1	BrM-specific protein
TGIF2	Q9GZN2	5	0	BrM-specific protein
TWSG1	Q9GZX9	5	1	BrM-specific protein
LAT2	Q9GZY6	5	1	BrM-specific protein
MIS12	Q9H081	6	2	BrM-specific protein
MUC3B	Q9H195	5	2	BrM-specific protein
XYLT2	Q9H1B5	6	1	BrM-specific protein
SLC35B3	Q9H1N7	6	0	BrM-specific protein
MS4A6A	Q9H2W1	7	2	BrM-specific protein
PARL	Q9H300	5	2	BrM-specific protein
FOXP1	Q9H334	5	2	BrM-specific protein
GGNBP2	Q9H3C7	5	1	BrM-specific protein
TRIT1	Q9H3H1	5	0	BrM-specific protein
ZNF768	Q9H5H4	5	1	BrM-specific protein

TFB2M	Q9H5Q4	5	1	BrM-specific protein
DENND2D	Q9H6A0	6	2	BrM-specific protein
DHX33	Q9H6R0	5	0	BrM-specific protein
ESRP2	Q9H6T0	5	2	BrM-specific protein
SH2D4A	Q9H788	5	2	BrM-specific protein
ISG20L2	Q9H9L3	5	2	BrM-specific protein
APOBEC3G	Q9HC16	5	2	BrM-specific protein
RAVER2	Q9HCJ3	5	2	BrM-specific protein
GP6	Q9HCN6	5	2	BrM-specific protein
TUFT1	Q9NNX1	5	1	BrM-specific protein
PCDH12	Q9NPG4	6	2	BrM-specific protein
DNAAF6	Q9NQM4	6	1	BrM-specific protein
SLC35C2	Q9NQQ7	5	0	BrM-specific protein
NECTIN3	Q9NQS3	5	2	BrM-specific protein
TLR8	Q9NR97	5	2	BrM-specific protein
LTBP3	Q9NS15	6	2	BrM-specific protein
TM7SF3	Q9NS93	7	1	BrM-specific protein
KRT84	Q9NSB2	5	0	BrM-specific protein
ZBTB40	Q9NUA8	5	2	BrM-specific protein
C14orf119	Q9NWQ9	5	0	BrM-specific protein
ZNF446	Q9NWS9	5	0	BrM-specific protein
RAB20	Q9NX57	6	2	BrM-specific protein
CMTM6	Q9NX76	6	2	BrM-specific protein
TMEM260	Q9NX78	5	2	BrM-specific protein
NSMCE4A	Q9NXX6	6	1	BrM-specific protein
CLEC5A	Q9NY25	5	2	BrM-specific protein
CELSR1	Q9NYQ6	5	2	BrM-specific protein
EHF	Q9NZC4	5	0	BrM-specific protein
MRPL35	Q9NZE8	5	1	BrM-specific protein
GRHL1	Q9NZI5	5	0	BrM-specific protein
EIF2AK3	Q9NZJ5	5	2	BrM-specific protein
NOP53	Q9NZM5	6	2	BrM-specific protein
HMG20B	Q9P0W2	5	0	BrM-specific protein
CHPF2	Q9P2E5	6	2	BrM-specific protein
XPR1	Q9UBH6	5	1	BrM-specific protein
METTL1	Q9UBP6	5	2	BrM-specific protein
GULP1	Q9UBP9	5	2	BrM-specific protein
CKLF	Q9UBR5	5	2	BrM-specific protein

DNAJB9	Q9UBS3	5	0	BrM-specific protein
MTFP1	Q9UDX5	5	2	BrM-specific protein
MARCO	Q9UEW3	5	2	BrM-specific protein
APOBEC3B	Q9UH17	5	2	BrM-specific protein
MLX	Q9UH92	5	1	BrM-specific protein
RSL24D1	Q9UHA3	5	1	BrM-specific protein
BAIAP2L1	Q9UHR4	5	2	BrM-specific protein
ADGRE2	Q9UHX3	6	2	BrM-specific protein
NAGPA	Q9UK23	5	2	BrM-specific protein
BRPF3	Q9ULD4	5	2	BrM-specific protein
PLEKHG1	Q9ULL1	6	2	BrM-specific protein
ABT1	Q9ULW3	5	0	BrM-specific protein
PADI4	Q9UM07	6	2	BrM-specific protein
L1RE1	Q9UN81	6	2	BrM-specific protein
FZD1	Q9UP38	5	2	BrM-specific protein
CDK11A	Q9UQ88	5	1	BrM-specific protein
LAMP3	Q9UQV4	5	0	BrM-specific protein
GINS2	Q9Y248	5	2	BrM-specific protein
SLC9A8	Q9Y2E8	5	2	BrM-specific protein
UTP11	Q9Y3A2	5	1	BrM-specific protein
RRP15	Q9Y3B9	5	1	BrM-specific protein
PKP3	Q9Y446	6	2	BrM-specific protein
LOXL2	Q9Y4K0	6	2	BrM-specific protein
RIPK3	Q9Y572	5	2	BrM-specific protein
FLVCR1	Q9Y5Y0	5	2	BrM-specific protein
TAF6L	Q9Y6J9	5	2	BrM-specific protein
CAPN6	Q9Y6Q1	7	2	BrM-specific protein
NCOA3	Q9Y6Q9	6	1	BrM-specific protein

supplementary table 5 Table of Drug Target Proteins and Their Associated Drugs

gene_claim_name	gene_concept_id	gene_name	group	interaction_source_db_name	interaction_source_db_version	interaction_type	interaction_score	drug_claim_name	drug_concept_id	drug_name	approved	immunotherapy	anti_neoplastic
CA12	hgnc:1371	CA12	BrM-specific protein	ChEMBL	33	inhibitor	0.803688845	ACETAZOLAMIDE	rxcui:167	ACETAZOLAMIDE	TRUE	FALSE	FALSE
CA12	hgnc:1371	CA12	BrM-specific protein	ChEMBL	33	inhibitor	0.937636986	ACETAZOLAMIDE SODIUM	rxcui:91406	ACETAZOLAMIDE SODIUM	TRUE	FALSE	FALSE
PCSK9	hgnc:20001	PCSK9	BrM-specific protein	ChEMBL	33	inhibitor	4.039051634	ALIROCUMAB	rxcui:1659152	ALIROCUMAB	TRUE	FALSE	FALSE
C5AR1	hgnc:1338	C5AR1	BrM-specific protein	ChEMBL	33	inhibitor	6.563458904	AVACOPAN	rxcui:2572100	AVACOPAN	TRUE	FALSE	FALSE
NCBIGENE:1805	hgnc:3011	DPT	BrM-specific protein	GuideToPharmacology	2024.1	inhibitor	0.198142156	IUPHAR.LIGAND:10368	rxcui:2272107	AVAPRITINIB	TRUE	FALSE	TRUE
IL17RA	hgnc:5985	IL17RA	BrM-specific protein	ChEMBL	33	inhibitor	35.00511416	BRODALUMAB	rxcui:1872251	BRODALUMAB	TRUE	FALSE	FALSE
TYMS	hgnc:12441	TYMS	BrM-specific protein	ChEMBL	33	inhibitor	0.062065805	CAPECITABINE	rxcui:194000	CAPECITABINE	TRUE	FALSE	TRUE
TYMS	hgnc:12441	TYMS	BrM-specific protein	ClearityFoundationClinicalTrial	41440	inhibitor	0.062065805	CAPECITABINE	rxcui:194000	CAPECITABINE	TRUE	FALSE	TRUE
PRIM1	hgnc:9369	PRIM1	BrM-specific protein	ChEMBL	33	inhibitor	0.394794521	CLOFARABINE	rxcui:44151	CLOFARABINE	TRUE	FALSE	TRUE
POLD3	hgnc:20932	POLD3	BrM-specific protein	ChEMBL	33	inhibitor	0.460593607	CLOFARABINE	rxcui:44151	CLOFARABINE	TRUE	FALSE	TRUE
POLD3	hgnc:20932	POLD3	BrM-specific protein	ChEMBL	33	inhibitor	0.108040476	CYTARABINE	rxcui:3041	CYTARABINE	TRUE	FALSE	TRUE
PRIM1	hgnc:9369	PRIM1	BrM-specific protein	ChEMBL	33	inhibitor	0.092606122	CYTARABINE	rxcui:3041	CYTARABINE	TRUE	FALSE	TRUE
CA12	hgnc:1371	CA12	BrM-specific protein	ChEMBL	33	inhibitor	0.750109589	DICHLORPHENAMIDE	rxcui:3353	DICHLORPHENAMIDE	TRUE	FALSE	FALSE
NCBIGENE:2603	hgnc:2861	DHFR	BrM-specific protein	GuideToPharmacology	2024.1	inhibitor	0.328172945	IUPHAR.LIGAND:7162	rxcui:3393	DIFLUNISAL	TRUE	FALSE	FALSE
MMP7	hgnc:7174	MMP7	BrM-specific protein	ChEMBL	33	inhibitor	0.652269208	DOXYCYCLINE HYCLATE	rxcui:1545992	DOXYCYCLINE ANHYDROUS	TRUE	FALSE	TRUE
NCBIGENE:1631	hgnc:7174	MMP7	BrM-specific protein	GuideToPharmacology	2024.1	inhibitor	0.652269208	IUPHAR.LIGAND:6464	rxcui:1545992	DOXYCYCLINE ANHYDROUS	TRUE	FALSE	TRUE
MMP8	hgnc:7175	MMP8	BrM-specific protein	ChEMBL	33	inhibitor	1.875273973	DOXYCYCLINE CALCIUM	rxcui:82012	DOXYCYCLINE CALCIUM	TRUE	FALSE	FALSE
MMP7	hgnc:7174	MMP7	BrM-specific protein	ChEMBL	33	inhibitor	1.875273973	DOXYCYCLINE CALCIUM	rxcui:82012	DOXYCYCLINE CALCIUM	TRUE	FALSE	FALSE
NCBIGENE:3024	hgnc:4715	H1-1	BrM-specific protein	GuideToPharmacology	2024.1	inhibitor	2.625383562	IUPHAR.LIGAND:6647	rxcui:60307	ENTACAPONE	TRUE	FALSE	FALSE
CA9	hgnc:1383	CA9	BrM-specific protein	ChEMBL	33	inhibitor	0.25003653	ETHOXZOLAMIDE	ncit:C65561	ETHOXZOLAMIDE	TRUE	FALSE	FALSE
CA12	hgnc:1371	CA12	BrM-specific protein	ChEMBL	33	inhibitor	0.750109589	ETHOXZOLAMIDE	ncit:C65561	ETHOXZOLAMIDE	TRUE	FALSE	FALSE
PCSK9	hgnc:20001	PCSK9	BrM-specific protein	ChEMBL	33	inhibitor	6.05857745	EVOLOCUMAB	rxcui:1665684	EVOLOCUMAB	TRUE	FALSE	FALSE
TYMS	hgnc:12441	TYMS	BrM-specific protein	ClearityFoundationClinicalTrial	41440	inhibitor	0.31919557	FLOXURIDINE	rxcui:4488	FLOXURIDINE	TRUE	FALSE	TRUE
TYMS	hgnc:12441	TYMS	BrM-specific protein	ChEMBL	33	inhibitor	0.31919557	FLOXURIDINE	rxcui:4488	FLOXURIDINE	TRUE	FALSE	TRUE
PRIM1	hgnc:9369	PRIM1	BrM-specific protein	ChEMBL	33	inhibitor	0.220620467	FLUDARABINE PHOSPHATE	rxcui:24698	FLUDARABINE	TRUE	FALSE	TRUE
POLD3	hgnc:20932	POLD3	BrM-specific protein	ChEMBL	33	inhibitor	0.257390545	FLUDARABINE PHOSPHATE	rxcui:24698	FLUDARABINE	TRUE	FALSE	TRUE
TYMS	hgnc:12441	TYMS	BrM-specific protein	ClearityFoundationClinicalTrial	41440	inhibitor	0.046940525	FLUOROURACIL	rxcui:4492	FLUOROURACIL	TRUE	FALSE	TRUE
TYMS	hgnc:12441	TYMS	BrM-specific protein	ChEMBL	33	inhibitor	0.046940525	FLUOROURACIL	rxcui:4492	FLUOROURACIL	TRUE	FALSE	TRUE
POLD3	hgnc:20932	POLD3	BrM-specific protein	ChEMBL	33	inhibitor	0.094099769	GEMCITABINE HYDROCHLORIDE	rxcui:12574	GEMCITABINE	TRUE	FALSE	TRUE
PRIM1	hgnc:9369	PRIM1	BrM-specific protein	ChEMBL	33	inhibitor	0.080656945	GEMCITABINE HYDROCHLORIDE	rxcui:12574	GEMCITABINE	TRUE	FALSE	TRUE
NCBIGENE:1805	hgnc:3011	DPT	BrM-specific protein	GuideToPharmacology	2024.1	inhibitor	0.058277105	IUPHAR.LIGAND:5656	ncit:C79910	MASITINIB	TRUE	FALSE	TRUE
DHFR	hgnc:2861	DHFR	BrM-specific protein	ChEMBL	33	inhibitor	0.190457513	METHOTREXATE	rxcui:6851	METHOTREXATE	TRUE	FALSE	TRUE
NCBIGENE:2603	hgnc:2861	DHFR	BrM-specific protein	GuideToPharmacology	2024.1	inhibitor	0.190457513	IUPHAR.LIGAND:4815	rxcui:6851	METHOTREXATE	TRUE	FALSE	TRUE
DHFR	hgnc:2861	DHFR	BrM-specific protein	ChEMBL	33	inhibitor	3.281729452	METHOTREXATE SODIUM	rxcui:287734	METHOTREXATE SODIUM	TRUE	FALSE	FALSE
ITGB7	hgnc:6162	ITGB7	BrM-specific protein	ChEMBL	33	inhibitor	2.187819635	NATALIZUMAB	rxcui:354770	NATALIZUMAB	TRUE	TRUE	FALSE
APH1A	hgnc:29509	APH1A	BrM-specific protein	ChEMBL	33	inhibitor	0.267896282	NIROGACESTAT	rxcui:2670631	NIROGACESTAT	TRUE	FALSE	TRUE
NCBIGENE:3024	hgnc:4715	H1-1	BrM-specific protein	GuideToPharmacology	2024.1	inhibitor	1.009762908	IUPHAR.LIGAND:10917	rxcui:235559	NITROFURANTOIN, MACROCRYSTALS	TRUE	FALSE	FALSE
SIRT6	hgnc:14934	SIRT6	BrM-specific protein	TALC	42502	inhibitor	1.591141553	PANOBINOSTAT	rxcui:1603350	PANOBINOSTAT	TRUE	FALSE	TRUE
NCBIGENE:1805	hgnc:3011	DPT	BrM-specific protein	GuideToPharmacology	2024.1	inhibitor	0.01708122	IUPHAR.LIGAND:5698	rxcui:714438	PAZOPANIB	TRUE	FALSE	TRUE
TYMS	hgnc:12441	TYMS	BrM-specific protein	ChEMBL	33	inhibitor	0.268124279	PEMETREXED DISODIUM	rxcui:282443	PEMETREXED DISODIUM	TRUE	FALSE	TRUE
DHFR	hgnc:2861	DHFR	BrM-specific protein	ChEMBL	33	inhibitor	0.525076712	PEMETREXED DISODIUM	rxcui:282443	PEMETREXED DISODIUM	TRUE	FALSE	TRUE
DHFR	hgnc:2861	DHFR	BrM-specific protein	ClearityFoundationClinicalTrial	41440	inhibitor	0.525076712	PEMETREXED	rxcui:282443	PEMETREXED DISODIUM	TRUE	FALSE	TRUE
TYMS	hgnc:12441	TYMS	BrM-specific protein	ClearityFoundationClinicalTrial	41440	inhibitor	0.268124279	MLS003106905	rxcui:282443	PEMETREXED DISODIUM	TRUE	FALSE	TRUE
NCBIGENE:2603	hgnc:2861	DHFR	BrM-specific protein	GuideToPharmacology	2024.1	inhibitor	0.525076712	IUPHAR.LIGAND:6837	rxcui:282443	PEMETREXED DISODIUM	TRUE	FALSE	TRUE
DHFR	hgnc:2861	DHFR	BrM-specific protein	ChEMBL	33	inhibitor	0.820432363	PEMETREXED TROMETHAMINE	ncit:C170306	PEMETREXED TROMETHAMINE	TRUE	FALSE	FALSE
NCBIGENE:1805	hgnc:3011	DPT	BrM-specific protein	GuideToPharmacology	2024.1	inhibitor	0.070765056	IUPHAR.LIGAND:8710	rxcui:2183102	PEXIDARTINIB	TRUE	FALSE	TRUE
DHFR	hgnc:2861	DHFR	BrM-specific protein	ClearityFoundationClinicalTrial	41440	inhibitor	2.734774544	PRALATREXATE	rxcui:662019	PRALATREXATE	TRUE	FALSE	TRUE
DHFR	hgnc:2861	DHFR	BrM-specific protein	ChEMBL	33	inhibitor	2.734774544	PRALATREXATE	rxcui:662019	PRALATREXATE	TRUE	FALSE	TRUE
NCBIGENE:2603	hgnc:2861	DHFR	BrM-specific protein	GuideToPharmacology	2024.1	inhibitor	2.734774544	IUPHAR.LIGAND:6840	rxcui:662019	PRALATREXATE	TRUE	FALSE	TRUE
NCBIGENE:1805	hgnc:3011	DPT	BrM-specific protein	GuideToPharmacology	2024.1	inhibitor	0.052142673	IUPHAR.LIGAND:5658	rxcui:2643048	QUIZARTINIB	TRUE	FALSE	TRUE
MME	hgnc:7154	MME	BrM-specific protein	ChEMBL	33	inhibitor	4.773424658	RACECADOTRIL	rxcui:16738	RACECADOTRIL	TRUE	FALSE	FALSE
TYMS	hgnc:12441	TYMS	BrM-specific protein	ChEMBL	33	inhibitor	1.117184494	RALTITREXED	rxcui:196239	RALTITREXED	TRUE	FALSE	TRUE
NCBIGENE:1805	hgnc:3011	DPT	BrM-specific protein	GuideToPharmacology	2024.1	inhibitor	0.198142156	IUPHAR.LIGAND:9175	rxcui:2369389	RIPRETINIB	TRUE	FALSE	TRUE
MME	hgnc:7154	MME	BrM-specific protein	ChEMBL	33	inhibitor	4.773424658	SACUBITRIL	rxcui:1656328	SACUBITRIL	TRUE	FALSE	FALSE
NCBIGENE:1805	hgnc:3011	DPT	BrM-specific protein	GuideToPharmacology	2024.1	inhibitor	0.008690445	IUPHAR.LIGAND:5711	rxcui:495881	SORAFENIB	TRUE	FALSE	TRUE
CA12	hgnc:1371	CA12	BrM-specific protein	ChEMBL	33	inhibitor	0.312545662	SULTHIAME	rxcui:10240	SULTHIAME	TRUE	FALSE	FALSE
CA9	hgnc:1383	CA9	BrM-specific protein	ChEMBL	33	inhibitor	0.312545662	SULTHIAME	rxcui:10240	SULTHIAME	TRUE	FALSE	FALSE
NCBIGENE:1805	hgnc:3011	DPT	BrM-specific protein	GuideToPharmacology	2024.1	inhibitor	0.020218587	IUPHAR.LIGAND:5713	rxcui:357977	SUNITINIB	TRUE	FALSE	TRUE
NCBIGENE:2603	hgnc:2861	DHFR	BrM-specific protein	GuideToPharmacology	2024.1	inhibitor	0.546954909	IUPHAR.LIGAND:7613	rxcui:42333	TRIMETREXATE	TRUE	FALSE	TRUE
TYMS	hgnc:12441	TYMS	BrM-specific protein	ChEMBL	33	inhibitor	0.186197416	TEGAFUR	rxcui:10995	URACIL	TRUE	FALSE	TRUE
ITGB7	hgnc:6162	ITGB7	BrM-specific protein	ChEMBL	33	inhibitor	8.751278539	VEDOLIZUMAB	rxcui:1538097	VEDOLIZUMAB	TRUE	FALSE	FALSE
GRIN2B	hgnc:4586	GRIN2B	NSCLC-specific protein	ChEMBL	33	inhibitor	0.026927011	ACAMPROSATE CALCIUM	rxcui:152761	ACAMPROSATE CALCIUM	TRUE	FALSE	FALSE
NCBIGENE:2598	hgnc:1372	CA14	NSCLC-specific protein	GuideToPharmacology	2024.1	inhibitor	0.468818493	IUPHAR.LIGAND:6792	rxcui:167	ACETAZOLAMIDE	TRUE	FALSE	FALSE
NCBIGENE:457	hgnc:4586	GRIN2B	NSCLC-specific protein	GuideToPharmacology	2024.1	blocker	0.134635054	IUPHAR.LIGAND:4128	rxcui:282415	AMANTADINE HYDROCHLORIDE	TRUE	FALSE	FALSE
GRIN2B	hgnc:4586	GRIN2B	NSCLC-specific protein	ChEMBL	33	inhibitor	0.134635054	AMANTADINE HYDROCHLORIDE	rxcui:282415	AMANTADINE HYDROCHLORIDE	TRUE	FALSE	FALSE
EDNRB	hgnc:3180	EDNRB	NSCLC-specific protein	ChEMBL	33	inhibitor	2.250328767	AMBRISENTAN	rxcui:358274	AMBRISENTAN	TRUE	FALSE	FALSE
KCND1	hgnc:6237	KCND1	NSCLC-specific protein	ChEMBL	33	blocker	0.208363775	AMIFAMPRIDINE	rxcui:2106338	AMIFAMPRIDINE	TRUE	FALSE	FALSE
KCNA4	hgnc:6222	KCNA4	NSCLC-specific protein	ChEMBL	33	blocker	0.178597521	AMIFAMPRIDINE	rxcui:2106338	AMIFAMPRIDINE	TRUE	FALSE	FALSE
KCND3	hgnc:6239	KCND3	NSCLC-specific protein	ChEMBL	33	blocker	0.156272831	AMIFAMPRIDINE	rxcui:2106338	AMIFAMPRIDINE	TRUE	FALSE	FALSE
KCNC3	hgnc:6235	KCNC3	NSCLC-specific protein	ChEMBL	33	blocker	0.208363775	AMIFAMPRIDINE	rxcui:2106338	AMIFAMPRIDINE	TRUE	FALSE	FALSE
KCNC3	hgnc:6235	KCNC3	NSCLC-specific protein	ChEMBL	33	blocker	0.213445818	AMIFAMPRIDINE PHOSPHATE	rxcui:2106337	AMIFAMPRIDINE PHOSPHATE	TRUE	FALSE	FALSE
KCND1	hgnc:6237	KCND1	NSCLC-specific protein	ChEMBL	33	blocker	0.213445818	AMIFAMPRIDINE PHOSPHATE	rxcui:2106337	AMIFAMPRIDINE PHOSPHATE	TRUE	FALSE	FALSE
KCNA4	hgnc:6222	KCNA4	NSCLC-specific protein	ChEMBL	33	blocker	0.182953558	AMIFAMPRIDINE PHOSPHATE	rxcui:2106337	AMIFAMPRIDINE PHOSPHATE	TRUE	FALSE	FALSE
KCND3	hgnc:6239	KCND3	NSCLC-specific protein	ChEMBL	33	blocker	0.160084364	AMIFAMPRIDINE PHOSPHATE	rxcui:2106337	AMIFAMPRIDINE PHOSPHATE	TRUE	FALSE	FALSE
PDE4A	hgnc:8780	PDE4A	NSCLC-specific protein	ChEMBL	33	inhibitor	0.119335616	AMINOPHYLLINE	rxcui:689	AMINOPHYLLINE	TRUE	FALSE	FALSE
PDE4A	hgnc:8780	PDE4A	NSCLC-specific protein	ChEMBL	33	inhibitor	0.091796628	AMLEXANOX	rxcui:46307	AMLEXANOX	TRUE	FALSE	FALSE
NCBIGENE:1268	hgnc:2159	CNR1	NSCLC-specific protein	GuideToPharmacology	2024.1	inhibitor	0.291709285	IUPHAR.LIGAND:6960	rxcui:17941	ANISINDIONE	TRUE	FALSE	FALSE
PDE4A	hgnc:8780	PDE4A	NSCLC-specific protein	ChEMBL	33	inhibitor	0.358006849	APREMILAST	rxcui:1492727	APREMILAST	TRUE	FALSE	FALSE
EDNRB	hgnc:3180	EDNRB	NSCLC-specific protein	ChEMBL	33	inhibitor	0.937636986	APROCITENTAN	ncit:C174728	APROCITENTAN	TRUE	FALSE	FALSE
SCN8A	hgnc:10596	SCN8A	NSCLC-specific protein	ChEMBL	33	blocker	0.088996053	ARTICAINE HYDROCHLORIDE	rxcui:2117	ARTICAINE HYDROCHLORIDE	TRUE	FALSE	FALSE
SCN1A	hgnc:10585	SCN1A	NSCLC-specific protein	ChEMBL	33	blocker	0.082043236	ARTICAINE HYDROCHLORIDE	rxcui:2117	ARTICAINE HYDROCHLORIDE	TRUE	FALSE	FALSE
PDE4A	hgnc:8780	PDE4A	NSCLC-specific protein	ChEMBL	33	inhibitor	0.025390557	DIPYRIDAMOLE	rxcui:1191	ASPIRIN	TRUE	FALSE	TRUE
SCN1A	hgnc:10585	SCN1A	NSCLC-specific protein	ChEMBL	33	blocker	0.07458476	BENOXINATE HYDROCHLORIDE	rxcui:235398	BENOXINATE HYDROCHLORIDE	TRUE	FALSE	FALSE
SCN8A	hgnc:10596	SCN8A	NSCLC-specific protein	ChEMBL	33	blocker	0.080905503	BENOXINATE HYDROCHLORIDE	rxcui:235398	BENOXINATE HYDROCHLORIDE	TRUE	FALSE	FALSE
EDNRB	hgnc:3180	EDNRB	NSCLC-specific protein	ChEMBL	33	inhibitor	3.750547945	BOSENTAN	rxcui:1468845	BOSENTAN ANHYDROUS	TRUE	FALSE	FALSE
NCBIGENE:2598	hgnc:1372	CA14	NSCLC-specific protein	GuideToPharmacology	2024.1	inhibitor	1.640864726	IUPHAR.LIGAND:6797	rxcui:194881	BRINZOLAMIDE	TRUE	FALSE	FALSE
CNR1	hgnc:2159	CNR1	NSCLC-specific protein	ChEMBL	33	negative modulator	0.194472856	CANNABIDIOL	rxcui:2045371	CANNABIDIOL	TRUE	FALSE	FALSE
SCN1A	hgnc:10585	SCN1A	NSCLC-specific protein	ChEMBL	33	blocker	0.016743518	CARBAMAZEPINE	rxcui:2002	CARBAMAZEPINE	TRUE	FALSE	FALSE

SCN8A	hgnc:10596	SCN8A	NSCLC-specific protein	ChEMBL	33	blocker	0.01816246	CARBAMAZEPINE	rxcui:2002	CARBAMAZEPINE	TRUE	FALSE	FALSE
SCN1A	hgnc:10585	SCN1A	NSCLC-specific protein	ChEMBL	33	inhibitor	0.029301156	CENOBAMATE	rxcui:2265690	CENOBAMATE	TRUE	FALSE	FALSE
SCN8A	hgnc:10596	SCN8A	NSCLC-specific protein	ChEMBL	33	inhibitor	0.031784305	CENOBAMATE	rxcui:2265690	CENOBAMATE	TRUE	FALSE	FALSE
SCN8A	hgnc:10596	SCN8A	NSCLC-specific protein	ChEMBL	33	blocker	0.068458502	CHLOROPROCAINE HYDROCHLORIDE	rxcui:20859	CHLOROPROCAINE	TRUE	FALSE	FALSE
SCN1A	hgnc:10585	SCN1A	NSCLC-specific protein	ChEMBL	33	blocker	0.063110182	CHLOROPROCAINE HYDROCHLORIDE	rxcui:20859	CHLOROPROCAINE	TRUE	FALSE	FALSE
NCBIGENE:2598	hgnc:1372	CA14	NSCLC-specific protein	GuideToPharmacology	2024.1	inhibitor	0.729273212	IUPHAR.LIGAND:7147	rxcui:2409	CHLORTHALIDONE	TRUE	FALSE	FALSE
SCN1A	hgnc:10585	SCN1A	NSCLC-specific protein	ChEMBL	33	blocker	0.082043236	COCAINE HYDROCHLORIDE	rxcui:81985	COCAINE HYDROCHLORIDE	TRUE	FALSE	FALSE
SCN8A	hgnc:10596	SCN8A	NSCLC-specific protein	ChEMBL	33	blocker	0.088996053	COCAINE HYDROCHLORIDE	rxcui:81985	COCAINE HYDROCHLORIDE	TRUE	FALSE	FALSE
PDE4A	hgnc:8780	PDE4A	NSCLC-specific protein	ChEMBL	33	inhibitor	0.511438356	CRISABOROLE	rxcui:1865953	CRISABOROLE	TRUE	FALSE	FALSE
KCNC3	hgnc:6235	KCNC3	NSCLC-specific protein	ChEMBL	33	blocker	0.182318303	DALFAMPRIDINE	rxcui:897018	DALFAMPRIDINE	TRUE	FALSE	FALSE
KCNA4	hgnc:6222	KCNA4	NSCLC-specific protein	ChEMBL	33	blocker	0.156272831	DALFAMPRIDINE	rxcui:897018	DALFAMPRIDINE	TRUE	FALSE	FALSE
KCND3	hgnc:6239	KCND3	NSCLC-specific protein	ChEMBL	33	blocker	0.136738727	DALFAMPRIDINE	rxcui:897018	DALFAMPRIDINE	TRUE	FALSE	FALSE
KCND1	hgnc:6237	KCND1	NSCLC-specific protein	ChEMBL	33	blocker	0.182318303	DALFAMPRIDINE	rxcui:897018	DALFAMPRIDINE	TRUE	FALSE	FALSE
RYR3	hgnc:10485	RYR3	NSCLC-specific protein	ChEMBL	33	inhibitor	1.458546423	DANTROLENE SODIUM	rxcui:3105	DANTROLENE	TRUE	FALSE	FALSE
NCBIGENE:749	hgnc:10485	RYR3	NSCLC-specific protein	GuideToPharmacology	2024.1	inhibitor	1.458546423	IUPHAR.LIGAND:4172	rxcui:3105	DANTROLENE	TRUE	FALSE	FALSE
SCN1A	hgnc:10585	SCN1A	NSCLC-specific protein	ChEMBL	33	blocker	0.082043236	DICHLOROBENZYL ALCOHOL	rxcui:22906	DICHLOROBENZYL ALCOHOL	TRUE	FALSE	FALSE
SCN8A	hgnc:10596	SCN8A	NSCLC-specific protein	ChEMBL	33	blocker	0.088996053	DICHLOROBENZYL ALCOHOL	rxcui:22906	DICHLOROBENZYL ALCOHOL	TRUE	FALSE	FALSE
SCN1A	hgnc:10585	SCN1A	NSCLC-specific protein	ChEMBL	33	blocker	0.07458476	DISOPYRAMIDE PHOSPHATE	rxcui:203178	DISOPYRAMIDE PHOSPHATE	TRUE	FALSE	FALSE
SCN8A	hgnc:10596	SCN8A	NSCLC-specific protein	ChEMBL	33	blocker	0.080905503	DISOPYRAMIDE PHOSPHATE	rxcui:203178	DISOPYRAMIDE PHOSPHATE	TRUE	FALSE	FALSE
NCBIGENE:2598	hgnc:1372	CA14	NSCLC-specific protein	GuideToPharmacology	2024.1	inhibitor	1.312691781	IUPHAR.LIGAND:6810	rxcui:60207	DORZOLAMIDE	TRUE	FALSE	FALSE
SCN8A	hgnc:10596	SCN8A	NSCLC-specific protein	ChEMBL	33	blocker	0.055622533	DRONEDARONE HYDROCHLORIDE	rxcui:736680	DRONEDARONE HYDROCHLORIDE	TRUE	FALSE	FALSE
SCN1A	hgnc:10585	SCN1A	NSCLC-specific protein	ChEMBL	33	blocker	0.051277023	DRONEDARONE HYDROCHLORIDE	rxcui:736680	DRONEDARONE HYDROCHLORIDE	TRUE	FALSE	FALSE
SCN8A	hgnc:10596	SCN8A	NSCLC-specific protein	ChEMBL	33	blocker	0.055622533	DYCLONINE HYDROCHLORIDE	rxcui:91188	DYCLONINE HYDROCHLORIDE	TRUE	FALSE	FALSE
SCN1A	hgnc:10585	SCN1A	NSCLC-specific protein	ChEMBL	33	blocker	0.051277023	DYCLONINE HYDROCHLORIDE	rxcui:91188	DYCLONINE HYDROCHLORIDE	TRUE	FALSE	FALSE
PDE4A	hgnc:8780	PDE4A	NSCLC-specific protein	ChEMBL	33	inhibitor	0.681917808	DYPHYLLINE	rxcui:3714	DYPHYLLINE	TRUE	FALSE	FALSE
SCN8A	hgnc:10596	SCN8A	NSCLC-specific protein	ChEMBL	33	blocker	0.088996053	ENCAINIDE HYDROCHLORIDE	rxcui:281189	ENCAINIDE HYDROCHLORIDE	TRUE	FALSE	FALSE
SCN1A	hgnc:10585	SCN1A	NSCLC-specific protein	ChEMBL	33	blocker	0.082043236	ENCAINIDE HYDROCHLORIDE	rxcui:281189	ENCAINIDE HYDROCHLORIDE	TRUE	FALSE	FALSE
CALCA	hgnc:1437	CALCA	NSCLC-specific protein	ChEMBL	33	inhibitor	0.648242855	EPTINEZUMAB	rxcui:2282660	EPTINEZUMAB	TRUE	FALSE	FALSE
FGFR2	hgnc:3689	FGFR2	NSCLC-specific protein	ChEMBL	33	inhibitor	0.864951949	ERDAFITINIB	rxcui:2123125	ERDAFITINIB	TRUE	FALSE	TRUE
GRIN2B	hgnc:4586	GRIN2B	NSCLC-specific protein	ChEMBL	33	negative modulator	0.096167896	ESKETAMINE	rxcui:2119365	ESKETAMINE	TRUE	FALSE	FALSE
SCN1A	hgnc:10585	SCN1A	NSCLC-specific protein	ChEMBL	33	blocker	0.07458476	ESLICARBAZEPINE	rxcui:1482502	ESLICARBAZEPINE	TRUE	FALSE	FALSE
SCN8A	hgnc:10596	SCN8A	NSCLC-specific protein	ChEMBL	33	blocker	0.080905503	ESLICARBAZEPINE	rxcui:1482502	ESLICARBAZEPINE	TRUE	FALSE	FALSE
SCN8A	hgnc:10596	SCN8A	NSCLC-specific protein	ChEMBL	33	blocker	0.080905503	ESLICARBAZEPINE ACETATE	rxcui:1482501	ESLICARBAZEPINE ACETATE	TRUE	FALSE	FALSE
SCN1A	hgnc:10585	SCN1A	NSCLC-specific protein	ChEMBL	33	blocker	0.07458476	ESLICARBAZEPINE ACETATE	rxcui:1482501	ESLICARBAZEPINE ACETATE	TRUE	FALSE	FALSE
SCN8A	hgnc:10596	SCN8A	NSCLC-specific protein	ChEMBL	33	blocker	0.088996053	ETHOTOIN	rxcui:4136	ETHOTOIN	TRUE	FALSE	FALSE
SCN1A	hgnc:10585	SCN1A	NSCLC-specific protein	ChEMBL	33	blocker	0.082043236	ETHOTOIN	rxcui:4136	ETHOTOIN	TRUE	FALSE	FALSE
NCBIGENE:2598	hgnc:1372	CA14	NSCLC-specific protein	GuideToPharmacology	2024.1	inhibitor	0.875127854	IUPHAR.LIGAND:6814	ncit:C65561	ETHOXZOLAMIDE	TRUE	FALSE	FALSE
CA14	hgnc:1372	CA14	NSCLC-specific protein	ChEMBL	33	inhibitor	0.875127854	ETHOXZOLAMIDE	ncit:C65561	ETHOXZOLAMIDE	TRUE	FALSE	FALSE
SCN1A	hgnc:10585	SCN1A	NSCLC-specific protein	ChEMBL	33	blocker	0.082043236	ETIDOCAINE HYDROCHLORIDE	rxcui:227221	ETIDOCAINE HYDROCHLORIDE	TRUE	FALSE	FALSE
SCN8A	hgnc:10596	SCN8A	NSCLC-specific protein	ChEMBL	33	blocker	0.088996053	ETIDOCAINE HYDROCHLORIDE	rxcui:227221	ETIDOCAINE HYDROCHLORIDE	TRUE	FALSE	FALSE
GRIN2B	hgnc:4586	GRIN2B	NSCLC-specific protein	ChEMBL	33	inhibitor	0.168293818	FELBAMATE	rxcui:24812	FELBAMATE	TRUE	FALSE	FALSE
PDE4A	hgnc:8780	PDE4A	NSCLC-specific protein	ChEMBL	33	inhibitor	0.149169521	FLAVOXATE HYDROCHLORIDE	rxcui:142437	FLAVOXATE HYDROCHLORIDE	TRUE	FALSE	FALSE
GABRG2	hgnc:4087	GABRG2	NSCLC-specific protein	ChEMBL	33	negative modulator	0.026045472	FLUMAZENIL	rxcui:4457	FLUMAZENIL	TRUE	FALSE	FALSE
GABRB3	hgnc:4083	GABRB3	NSCLC-specific protein	ChEMBL	33	negative modulator	0.027010119	FLUMAZENIL	rxcui:4457	FLUMAZENIL	TRUE	FALSE	FALSE
GABRA2	hgnc:4076	GABRA2	NSCLC-specific protein	ChEMBL	33	negative modulator	0.096167896	FLUMAZENIL	rxcui:4457	FLUMAZENIL	TRUE	FALSE	FALSE
SCN8A	hgnc:10596	SCN8A	NSCLC-specific protein	ChEMBL	33	blocker	0.068458502	FOSPHENYTOIN SODIUM	rxcui:72236	FOSPHENYTOIN	TRUE	FALSE	FALSE
SCN1A	hgnc:10585	SCN1A	NSCLC-specific protein	ChEMBL	33	blocker	0.063110182	FOSPHENYTOIN SODIUM	rxcui:72236	FOSPHENYTOIN	TRUE	FALSE	FALSE
CALCA	hgnc:1437	CALCA	NSCLC-specific protein	ChEMBL	33	inhibitor	0.486182141	FREMANEZUMAB	rxcui:2056691	FREMANEZUMAB	TRUE	FALSE	FALSE
FGFR2	hgnc:3689	FGFR2	NSCLC-specific protein	ChEMBL	33	inhibitor	0.832574068	FUTIBATINIB	rxcui:2628190	FUTIBATINIB	TRUE	FALSE	TRUE
CALCA	hgnc:1437	CALCA	NSCLC-specific protein	ChEMBL	33	inhibitor	0.648242855	GALCANEZUMAB	rxcui:2058846	GALCANEZUMAB	TRUE	FALSE	FALSE
KCND3	hgnc:6239	KCND3	NSCLC-specific protein	ChEMBL	33	blocker	0.160084364	GUANIDINE HYDROCHLORIDE	rxcui:50676	GUANIDINE HYDROCHLORIDE	TRUE	FALSE	FALSE
KCNA4	hgnc:6222	KCNA4	NSCLC-specific protein	ChEMBL	33	blocker	0.182953558	GUANIDINE HYDROCHLORIDE	rxcui:50676	GUANIDINE HYDROCHLORIDE	TRUE	FALSE	FALSE
KCND1	hgnc:6237	KCND1	NSCLC-specific protein	ChEMBL	33	blocker	0.213445818	GUANIDINE HYDROCHLORIDE	rxcui:50676	GUANIDINE HYDROCHLORIDE	TRUE	FALSE	FALSE
KCNC3	hgnc:6235	KCNC3	NSCLC-specific protein	ChEMBL	33	blocker	0.213445818	GUANIDINE HYDROCHLORIDE	rxcui:50676	GUANIDINE HYDROCHLORIDE	TRUE	FALSE	FALSE
SCN8A	hgnc:10596	SCN8A	NSCLC-specific protein	ChEMBL	33	blocker	0.088996053	HEXYLCAINE HYDROCHLORIDE	rxcui:1546032	HEXYLCAINE HYDROCHLORIDE	TRUE	FALSE	FALSE
SCN1A	hgnc:10585	SCN1A	NSCLC-specific protein	ChEMBL	33	blocker	0.082043236	HEXYLCAINE HYDROCHLORIDE	rxcui:1546032	HEXYLCAINE HYDROCHLORIDE	TRUE	FALSE	FALSE
SCN1A	hgnc:10585	SCN1A	NSCLC-specific protein	ChEMBL	33	blocker	0.082043236	INDECAINIDE HYDROCHLORIDE	ncit:C65911	INDECAINIDE HYDROCHLORIDE	TRUE	FALSE	FALSE
SCN8A	hgnc:10596	SCN8A	NSCLC-specific protein	ChEMBL	33	blocker	0.088996053	INDECAINIDE HYDROCHLORIDE	ncit:C65911	INDECAINIDE HYDROCHLORIDE	TRUE	FALSE	FALSE
FGFR2	hgnc:3689	FGFR2	NSCLC-specific protein	MyCancerGenome	42906	inhibitor	0.678393685	BGJ398	rxcui:2550729	INFIGRATINIB	TRUE	FALSE	TRUE
FGFR2	hgnc:3689	FGFR2	NSCLC-specific protein	MyCancerGenomeClinicalTrial	30-Feburary-2014	inhibitor	0.678393685	BGJ398	rxcui:2550729	INFIGRATINIB	TRUE	FALSE	TRUE
FGFR2	hgnc:3689	FGFR2	NSCLC-specific protein	ChEMBL	33	inhibitor	0.678393685	INFIGRATINIB	rxcui:2550729	INFIGRATINIB	TRUE	FALSE	TRUE
FGFR2	hgnc:3689	FGFR2	NSCLC-specific protein	TALC	42502	inhibitor	0.678393685	BGJ398	rxcui:2550729	INFIGRATINIB	TRUE	FALSE	TRUE
FGFR2	hgnc:3689	FGFR2	NSCLC-specific protein	CancerCommons	41480	inhibitor	0.678393685	BGJ398	rxcui:2550729	INFIGRATINIB	TRUE	FALSE	TRUE
FGFR2	hgnc:3689	FGFR2	NSCLC-specific protein	ChEMBL	33	inhibitor	0.152638579	INFIGRATINIB PHOSPHATE	ncit:C175088	INFIGRATINIB PHOSPHATE	TRUE	FALSE	FALSE
NCBIGENE:457	hgnc:4586	GRIN2B	NSCLC-specific protein	GuideToPharmacology	2024.1	blocker	0.079197091	IUPHAR.LIGAND:4233	rxcui:203184	KETAMINE HYDROCHLORIDE	TRUE	FALSE	FALSE
GRIN2B	hgnc:4586	GRIN2B	NSCLC-specific protein	ChEMBL	33	negative modulator	0.079197091	KETAMINE HYDROCHLORIDE	rxcui:203184	KETAMINE HYDROCHLORIDE	TRUE	FALSE	FALSE
SCN8A	hgnc:10596	SCN8A	NSCLC-specific protein	ChEMBL	33	blocker	0.068458502	ERLOSAMIDE	rxcui:623400	LACOSAMIDE	TRUE	FALSE	FALSE
SCN1A	hgnc:10585	SCN1A	NSCLC-specific protein	ChEMBL	33	blocker	0.063110182	ERLOSAMIDE	rxcui:623400	LACOSAMIDE	TRUE	FALSE	FALSE
SCN1A	hgnc:10585	SCN1A	NSCLC-specific protein	ChEMBL	33	blocker	0.261046661	LAMOTRIGINE	rxcui:28439	LAMOTRIGINE	TRUE	FALSE	FALSE
SCN8A	hgnc:10596	SCN8A	NSCLC-specific protein	ChEMBL	33	blocker	0.040452751	LAMOTRIGINE	rxcui:28439	LAMOTRIGINE	TRUE	FALSE	FALSE
FGFR2	hgnc:3689	FGFR2	NSCLC-specific protein	TALC	42502	inhibitor	0.169598421	LENVATINIB	rxcui:1603296	LENVATINIB	TRUE	FALSE	TRUE
SCN8A	hgnc:10596	SCN8A	NSCLC-specific protein	ChEMBL	33	blocker	0.052350619	LIDOCAINE	rxcui:6387	LIDOCAINE	TRUE	FALSE	FALSE
SCN1A	hgnc:10585	SCN1A	NSCLC-specific protein	ChEMBL	33	blocker	0.048260727	LIDOCAINE	rxcui:6387	LIDOCAINE	TRUE	FALSE	FALSE
SCN8A	hgnc:10596	SCN8A	NSCLC-specific protein	ChEMBL	33	blocker	0.088996053	LIDOCAINE HYDROCHLORIDE	rxcui:142440	LIDOCAINE HYDROCHLORIDE	TRUE	FALSE	FALSE
SCN1A	hgnc:10585	SCN1A	NSCLC-specific protein	ChEMBL	33	blocker	0.082043236	LIDOCAINE HYDROCHLORIDE	rxcui:142440	LIDOCAINE HYDROCHLORIDE	TRUE	FALSE	FALSE
MTTP	hgnc:7467	MTTP	NSCLC-specific protein	ChEMBL	33	inhibitor	5.250767124	LOMITAPIDE MESYLATE	rxcui:1364478	LOMITAPIDE MESYLATE	TRUE	FALSE	FALSE
EDNRB	hgnc:3180	EDNRB	NSCLC-specific protein	ChEMBL	33	inhibitor	2.812910959	MACITENTAN	rxcui:1442132	MACITENTAN	TRUE	FALSE	FALSE
FGFR2	hgnc:3689	FGFR2	NSCLC-specific protein	MyCancerGenome	42906	inhibitor	0.03591496	MASITINIB	ncit:C79910	MASITINIB	TRUE	FALSE	TRUE
MYL3	hgnc:7584	MYL3	NSCLC-specific protein	ChEMBL	33	inhibitor	1.944728564	MAVACAMTEN	rxcui:2600867	MAVACAMTEN	TRUE	FALSE	FALSE
GRIN2B	hgnc:4586	GRIN2B	NSCLC-specific protein	ChEMBL	33	negative modulator	0.224391757	MEMANTINE HYDROCHLORIDE	rxcui:236685	MEMANTINE HYDROCHLORIDE	TRUE	FALSE	FALSE
SCN1A	hgnc:10585	SCN1A	NSCLC-specific protein	ChEMBL	33	blocker	0.068369364	MEPHENYTOIN	rxcui:6757	MEPHENYTOIN	TRUE	FALSE	FALSE
SCN8A	hgnc:10596	SCN8A	NSCLC-specific protein	ChEMBL	33	blocker	0.074163377	MEPHENYTOIN	rxcui:6757	MEPHENYTOIN	TRUE	FALSE	FALSE
SCN8A	hgnc:10596	SCN8A	NSCLC-specific protein	ChEMBL	33	blocker	0.055622533	MEPIVACAINE HYDROCHLORIDE	rxcui:203185	MEPIVACAINE HYDROCHLORIDE	TRUE	FALSE	FALSE
SCN1A	hgnc:10585	SCN1A	NSCLC-specific protein	ChEMBL	33	blocker	0.051277023	MEPIVACAINE HYDROCHLORIDE	rxcui:203185	MEPIVACAINE HYDROCHLORIDE	TRUE	FALSE	FALSE
SCN8A	hgnc:10596	SCN8A	NSCLC-specific protein	ChEMBL	33	blocker	0.088996053	MERETHOXYLLINE PROCAINE	chembl:CHEMBL1200443	MERETHOXYLLINE PROCAINE	TRUE	FALSE	FALSE
SCN1A	hgnc:10585	SCN1A	NSCLC-specific protein	ChEMBL	33	blocker	0.082043236	MERETHOXYLLINE PROCAINE	chembl:CHEMBL1200443	MERETHOXYLLINE PROCAINE	TRUE	FALSE	FALSE
NCBIGENE:2598	hgnc:1372	CA14	NSCLC-specific protein	GuideToPharmacology	2024.1	inhibitor	0.65634589	IUPHAR.LIGAND:6828	rxcui:6826	METHAZOLAMIDE	TRUE	FALSE	FALSE
SCN8A	hgnc:10596	SCN8A	NSCLC-specific protein	ChEMBL	33	blocker	0.088996053	MEXILETINE HYDROCHLORIDE	rxcui:142138	MEXILETINE HYDROCHLORIDE	TRUE	FALSE	FALSE
SCN1A	hgnc:10585	SCN1A	NSCLC-specific protein	ChEMBL	33	blocker	0.082043236	MEXILETINE HYDROCHLORIDE	rxcui:142138	MEXILETINE HYDROCHLORIDE	TRUE	FALSE	FALSE
SCN1A	hgnc:10585	SCN1A	NSCLC-specific protein	ChEMBL	33	blocker	0.068369364	MORICIZINE HYDROCHLORIDE	rxcui:221126	MORICIZINE HYDROCHLORIDE	TRUE	FALSE	FALSE

SCN8A	hgnc:10596	SCN8A	NSCLC-specific protein	ChEMBL	33	blocker	0.074163377	MORICIZINE HYDROCHLORIDE	rxcui:221126	MORICIZINE HYDROCHLORIDE	TRUE	FALSE	FALSE
FGFR2	hgnc:3689	FGFR2	NSCLC-specific protein	ClearityFoundationClinicalTrial	41440	inhibitor	0.146533036	NINTEDANIB	rxcui:1592736	NINTEDANIB ESYLATE	TRUE	FALSE	TRUE
FGFR2	hgnc:3689	FGFR2	NSCLC-specific protein	ChEMBL	33	inhibitor	0.146533036	NINTEDANIB ESYLATE	rxcui:1592736	NINTEDANIB ESYLATE	TRUE	FALSE	TRUE
FGFR2	hgnc:3689	FGFR2	NSCLC-specific protein	MyCancerGenome	42906	inhibitor	0.146533036	NINTEDANIB	rxcui:1592736	NINTEDANIB ESYLATE	TRUE	FALSE	TRUE
GRIN2B	hgnc:4586	GRIN2B	NSCLC-specific protein	ChEMBL	33	inhibitor	0.032055965	ORPHENADRINE CITRATE	rxcui:7716	ORPHENADRINE CITRATE	TRUE	FALSE	FALSE
SCN8A	hgnc:10596	SCN8A	NSCLC-specific protein	ChEMBL	33	blocker	0.042379073	ORPHENADRINE CITRATE	rxcui:7716	ORPHENADRINE CITRATE	TRUE	FALSE	FALSE
SCN1A	hgnc:10585	SCN1A	NSCLC-specific protein	ChEMBL	33	blocker	0.039068208	ORPHENADRINE CITRATE	rxcui:7716	ORPHENADRINE CITRATE	TRUE	FALSE	FALSE
SCN1A	hgnc:10585	SCN1A	NSCLC-specific protein	ChEMBL	33	blocker	0.043180651	ORPHENADRINE HYDROCHLORIDE	rxcui:202723	ORPHENADRINE HYDROCHLORIDE	TRUE	FALSE	FALSE
GRIN2B	hgnc:4586	GRIN2B	NSCLC-specific protein	ChEMBL	33	inhibitor	0.035430277	ORPHENADRINE HYDROCHLORIDE	rxcui:202723	ORPHENADRINE HYDROCHLORIDE	TRUE	FALSE	FALSE
SCN8A	hgnc:10596	SCN8A	NSCLC-specific protein	ChEMBL	33	blocker	0.046840028	ORPHENADRINE HYDROCHLORIDE	rxcui:202723	ORPHENADRINE HYDROCHLORIDE	TRUE	FALSE	FALSE
SCN1A	hgnc:10585	SCN1A	NSCLC-specific protein	ChEMBL	33	blocker	0.045579576	OXCARBAZEPINE	rxcui:32624	OXCARBAZEPINE	TRUE	FALSE	FALSE
SCN8A	hgnc:10596	SCN8A	NSCLC-specific protein	ChEMBL	33	blocker	0.049442252	OXCARBAZEPINE	rxcui:32624	OXCARBAZEPINE	TRUE	FALSE	FALSE
PDE4A	hgnc:8780	PDE4A	NSCLC-specific protein	ChEMBL	33	inhibitor	0.238671233	OXTRIPHYLLINE	rxcui:20976	OXTRIPHYLLINE	TRUE	FALSE	FALSE
FGFR2	hgnc:3689	FGFR2	NSCLC-specific protein	ChEMBL	33	inhibitor	1.628144845	PEMIGATINIB	rxcui:2359268	PEMIGATINIB	TRUE	FALSE	TRUE
PDE4A	hgnc:8780	PDE4A	NSCLC-specific protein	ChEMBL	33	inhibitor	0.102287671	PENTOXIFYLLINE	rxcui:8013	PENTOXIFYLLINE	TRUE	FALSE	FALSE
SCN1A	hgnc:10585	SCN1A	NSCLC-specific protein	ChEMBL	33	blocker	0.223754281	PHENACEMIDE	rxcui:33253	PHENACEMIDE	TRUE	FALSE	FALSE
SCN8A	hgnc:10596	SCN8A	NSCLC-specific protein	ChEMBL	33	blocker	0.080905503	PHENACEMIDE	rxcui:33253	PHENACEMIDE	TRUE	FALSE	FALSE
SCN1A	hgnc:10585	SCN1A	NSCLC-specific protein	ChEMBL	33	blocker	0.051277023	PHENAZOPYRIDINE HYDROCHLORIDE	rxcui:203197	PHENAZOPYRIDINE HYDROCHLORIDE	TRUE	FALSE	FALSE
SCN8A	hgnc:10596	SCN8A	NSCLC-specific protein	ChEMBL	33	blocker	0.055622533	PHENAZOPYRIDINE HYDROCHLORIDE	rxcui:203197	PHENAZOPYRIDINE HYDROCHLORIDE	TRUE	FALSE	FALSE
SCN1A	hgnc:10585	SCN1A	NSCLC-specific protein	ChEMBL	33	blocker	0.058602312	PHENYTOIN SODIUM	rxcui:71227	PHENYTOIN SODIUM	TRUE	FALSE	FALSE
FGFR2	hgnc:3689	FGFR2	NSCLC-specific protein	TALC	42502	inhibitor	0.317488245	PONATINIB	rxcui:1364347	PONATINIB	TRUE	FALSE	TRUE
FGFR2	hgnc:3689	FGFR2	NSCLC-specific protein	MyCancerGenome	42906	inhibitor	0.317488245	PONATINIB	rxcui:1364347	PONATINIB	TRUE	FALSE	TRUE
SCN8A	hgnc:10596	SCN8A	NSCLC-specific protein	ChEMBL	33	blocker	0.080905503	PRILOCAINE	rxcui:8686	PRILOCAINE	TRUE	FALSE	FALSE
SCN1A	hgnc:10585	SCN1A	NSCLC-specific protein	ChEMBL	33	blocker	0.07458476	PRILOCAINE	rxcui:8686	PRILOCAINE	TRUE	FALSE	FALSE
SCN1A	hgnc:10585	SCN1A	NSCLC-specific protein	ChEMBL	33	blocker	0.07458476	PRILOCAINE HYDROCHLORIDE	rxcui:2557	PRILOCAINE HYDROCHLORIDE	TRUE	FALSE	FALSE
SCN8A	hgnc:10596	SCN8A	NSCLC-specific protein	ChEMBL	33	blocker	0.080905503	PRILOCAINE HYDROCHLORIDE	rxcui:2557	PRILOCAINE HYDROCHLORIDE	TRUE	FALSE	FALSE
SCN1A	hgnc:10585	SCN1A	NSCLC-specific protein	ChEMBL	33	blocker	0.023440925	PRIMIDONE	rxcui:8691	PRIMIDONE	TRUE	FALSE	FALSE
SCN8A	hgnc:10596	SCN8A	NSCLC-specific protein	ChEMBL	33	blocker	0.025427444	PRIMIDONE	rxcui:8691	PRIMIDONE	TRUE	FALSE	FALSE
SCN8A	hgnc:10596	SCN8A	NSCLC-specific protein	ChEMBL	33	blocker	0.088996053	PROCAINAMIDE HYDROCHLORIDE	rxcui:155056	PROCAINAMIDE HYDROCHLORIDE	TRUE	FALSE	FALSE
SCN1A	hgnc:10585	SCN1A	NSCLC-specific protein	ChEMBL	33	blocker	0.082043236	PROCAINAMIDE HYDROCHLORIDE	rxcui:155056	PROCAINAMIDE HYDROCHLORIDE	TRUE	FALSE	FALSE
SCN1A	hgnc:10585	SCN1A	NSCLC-specific protein	ChEMBL	33	blocker	0.082043236	PROCAINE HYDROCHLORIDE	rxcui:106558	PROCAINE HYDROCHLORIDE	TRUE	FALSE	FALSE
SCN8A	hgnc:10596	SCN8A	NSCLC-specific protein	ChEMBL	33	blocker	0.088996053	PROCAINE HYDROCHLORIDE	rxcui:106558	PROCAINE HYDROCHLORIDE	TRUE	FALSE	FALSE
SCN8A	hgnc:10596	SCN8A	NSCLC-specific protein	ChEMBL	33	blocker	0.063568609	PROPAFENONE HYDROCHLORIDE	rxcui:203135	PROPAFENONE HYDROCHLORIDE	TRUE	FALSE	FALSE
SCN1A	hgnc:10585	SCN1A	NSCLC-specific protein	ChEMBL	33	blocker	0.058602312	PROPAFENONE HYDROCHLORIDE	rxcui:203135	PROPAFENONE HYDROCHLORIDE	TRUE	FALSE	FALSE
SCN1A	hgnc:10585	SCN1A	NSCLC-specific protein	ChEMBL	33	blocker	0.082043236	PROPARACAINE HYDROCHLORIDE	rxcui:227778	PROPARACAINE HYDROCHLORIDE	TRUE	FALSE	FALSE
SCN8A	hgnc:10596	SCN8A	NSCLC-specific protein	ChEMBL	33	blocker	0.088996053	PROPARACAINE HYDROCHLORIDE	rxcui:227778	PROPARACAINE HYDROCHLORIDE	TRUE	FALSE	FALSE
SCN8A	hgnc:10596	SCN8A	NSCLC-specific protein	ChEMBL	33	blocker	0.088996053	PROPOXYCAINE HYDROCHLORIDE	ncit:C66493	PROPOXYCAINE HYDROCHLORIDE	TRUE	FALSE	FALSE
SCN1A	hgnc:10585	SCN1A	NSCLC-specific protein	ChEMBL	33	blocker	0.082043236	PROPOXYCAINE HYDROCHLORIDE	ncit:C66493	PROPOXYCAINE HYDROCHLORIDE	TRUE	FALSE	FALSE
SCN1A	hgnc:10585	SCN1A	NSCLC-specific protein	ChEMBL	33	blocker	0.082043236	QUINIDINE GLUCONATE	rxcui:35220	QUINIDINE GLUCONATE	TRUE	FALSE	FALSE
SCN8A	hgnc:10596	SCN8A	NSCLC-specific protein	ChEMBL	33	blocker	0.088996053	QUINIDINE GLUCONATE	rxcui:35220	QUINIDINE GLUCONATE	TRUE	FALSE	FALSE
SCN1A	hgnc:10585	SCN1A	NSCLC-specific protein	ChEMBL	33	blocker	0.082043236	QUINIDINE POLYGALACTURONATE	rxcui:41875	QUINIDINE POLYGALACTURONATE	TRUE	FALSE	FALSE
SCN8A	hgnc:10596	SCN8A	NSCLC-specific protein	ChEMBL	33	blocker	0.088996053	QUINIDINE POLYGALACTURONATE	rxcui:41875	QUINIDINE POLYGALACTURONATE	TRUE	FALSE	FALSE
SCN1A	hgnc:10585	SCN1A	NSCLC-specific protein	ChEMBL	33	blocker	0.082043236	QUINIDINE SULFATE	rxcui:9069	QUINIDINE SULFATE	TRUE	FALSE	FALSE
SCN8A	hgnc:10596	SCN8A	NSCLC-specific protein	ChEMBL	33	blocker	0.088996053	QUINIDINE SULFATE	rxcui:9069	QUINIDINE SULFATE	TRUE	FALSE	FALSE
FGFR2	hgnc:3689	FGFR2	NSCLC-specific protein	MyCancerGenome	42906	inhibitor	0.046965717	E-3810	rxcui:114979	RABEPRAZOLE	TRUE	FALSE	TRUE
FGFR2	hgnc:3689	FGFR2	NSCLC-specific protein	MyCancerGenomeClinicalTrial	30-Feburary-2014	inhibitor	0.111009876	REGORAFENIB	rxcui:1312397	REGORAFENIB	TRUE	FALSE	TRUE
FGFR2	hgnc:3689	FGFR2	NSCLC-specific protein	ChEMBL	33	inhibitor	0.111009876	REGORAFENIB	rxcui:1312397	REGORAFENIB	TRUE	FALSE	TRUE
FGFR2	hgnc:3689	FGFR2	NSCLC-specific protein	TALC	42502	inhibitor	0.111009876	REGORAFENIB	rxcui:1312397	REGORAFENIB	TRUE	FALSE	TRUE
SCN1A	hgnc:10585	SCN1A	NSCLC-specific protein	ChEMBL	33	blocker	0.035670972	RILUZOLE	rxcui:35623	RILUZOLE	TRUE	FALSE	FALSE
SCN8A	hgnc:10596	SCN8A	NSCLC-specific protein	ChEMBL	33	blocker	0.038693936	RILUZOLE	rxcui:35623	RILUZOLE	TRUE	FALSE	FALSE
CNR1	hgnc:2159	CNR1	NSCLC-specific protein	ChEMBL	33	inhibitor	1.750255708	RIMONABANT	ncit:C73244	RIMONABANT	TRUE	FALSE	FALSE
PDE4A	hgnc:8780	PDE4A	NSCLC-specific protein	ChEMBL	33	inhibitor	0.596678082	ROFLUMILAST	rxcui:1091836	ROFLUMILAST	TRUE	FALSE	FALSE
SCN1A	hgnc:10585	SCN1A	NSCLC-specific protein	ChEMBL	33	blocker	0.082043236	ROPIVACAINE HYDROCHLORIDE	rxcui:236539	ROPIVACAINE HYDROCHLORIDE	TRUE	FALSE	FALSE
SCN8A	hgnc:10596	SCN8A	NSCLC-specific protein	ChEMBL	33	blocker	0.088996053	ROPIVACAINE HYDROCHLORIDE	rxcui:236539	ROPIVACAINE HYDROCHLORIDE	TRUE	FALSE	FALSE
SCN8A	hgnc:10596	SCN8A	NSCLC-specific protein	ChEMBL	33	blocker	0.074163377	RUFINAMIDE	rxcui:69036	RUFINAMIDE	TRUE	FALSE	FALSE
SCN1A	hgnc:10585	SCN1A	NSCLC-specific protein	ChEMBL	33	blocker	0.068369364	RUFINAMIDE	rxcui:69036	RUFINAMIDE	TRUE	FALSE	FALSE
EDNRB	hgnc:3180	EDNRB	NSCLC-specific protein	ChEMBL	33	inhibitor	1.250182648	SITAXENTAN	ncit:C73038	SITAXENTAN	TRUE	FALSE	FALSE
EDNRB	hgnc:3180	EDNRB	NSCLC-specific protein	ChEMBL	33	inhibitor	1.875273973	SITAXENTAN SODIUM	ncit:C152373	SITAXENTAN SODIUM	TRUE	FALSE	FALSE
CA14	hgnc:1372	CA14	NSCLC-specific protein	ChEMBL	33	inhibitor	0.546954909	SULTHIAME	rxcui:10240	SULTHIAME	TRUE	FALSE	FALSE
SCN8A	hgnc:10596	SCN8A	NSCLC-specific protein	ChEMBL	33	blocker	0.080905503	TETRACAINE	rxcui:10391	TETRACAINE	TRUE	FALSE	FALSE
PDE4A	hgnc:8780	PDE4A	NSCLC-specific protein	ChEMBL	33	inhibitor	0.132595129	THEOPHYLLINE	rxcui:10438	THEOPHYLLINE	TRUE	FALSE	FALSE
PDE4A	hgnc:8780	PDE4A	NSCLC-specific protein	ChEMBL	33	inhibitor	0.119335616	THEOPHYLLINE SODIUM GLYCINATE	rxcui:155075	THEOPHYLLINE SODIUM GLYCINATE	TRUE	FALSE	FALSE
SCN1A	hgnc:10585	SCN1A	NSCLC-specific protein	ChEMBL	33	blocker	0.082043236	TOCAINIDE HYDROCHLORIDE	ncit:C47761	TOCAINIDE HYDROCHLORIDE	TRUE	FALSE	FALSE
SCN8A	hgnc:10596	SCN8A	NSCLC-specific protein	ChEMBL	33	blocker	0.088996053	TOCAINIDE HYDROCHLORIDE	ncit:C47761	TOCAINIDE HYDROCHLORIDE	TRUE	FALSE	FALSE
GRIK3	hgnc:4581	GRIK3	NSCLC-specific protein	ChEMBL	33	inhibitor	0.170479452	TOPIRAMATE	rxcui:38404	TOPIRAMATE	TRUE	FALSE	FALSE
SCN1A	hgnc:10585	SCN1A	NSCLC-specific protein	ChEMBL	33	blocker	0.05593857	TOPIRAMATE	rxcui:38404	TOPIRAMATE	TRUE	FALSE	FALSE
SCN8A	hgnc:10596	SCN8A	NSCLC-specific protein	ChEMBL	33	blocker	0.020226376	TOPIRAMATE	rxcui:38404	TOPIRAMATE	TRUE	FALSE	FALSE
SCN8A	hgnc:10596	SCN8A	NSCLC-specific protein	ChEMBL	33	blocker	0.035598421	ZONISAMIDE	rxcui:39998	ZONISAMIDE	TRUE	FALSE	FALSE
SCN1A	hgnc:10585	SCN1A	NSCLC-specific protein	ChEMBL	33	blocker	0.098451884	ZONISAMIDE	rxcui:39998	ZONISAMIDE	TRUE	FALSE	FALSE
PLAT	hgnc:9051	PLAT	Up in BrM	ChEMBL	33	inhibitor	3.182283105	AMINOCAPROIC ACID	rxcui:99	6-AMINOCAPROIC ACID	TRUE	FALSE	FALSE
NCBIGENE:1942	hgnc:3221	EFNA1	Up in BrM	GuideToPharmacology	2024.1	inhibitor	0.280789686	IUPHAR.LIGAND:8912	rxcui:1986808	ACALABRUTINIB	TRUE	FALSE	FALSE
EML4	hgnc:1316	EML4	Up in BrM	ChEMBL	33	inhibitor	0.380490371	ALECTINIB HYDROCHLORIDE	rxcui:1727454	ALECTINIB HYDROCHLORIDE	TRUE	FALSE	FALSE
DPP4	hgnc:3009	DPP4	Up in BrM	ChEMBL	33	inhibitor	1.050153425	ALOGLIPTIN BENZOATE	rxcui:1368000	ALOGLIPTIN BENZOATE	TRUE	FALSE	FALSE
NCBIGENE:317	hgnc:576	APAF1	Up in BrM	GuideToPharmacology	2024.1	inhibitor	0.092933931	IUPHAR.LIGAND:7471	rxcui:1516803	ALVIMOPAN ANHYDROUS	TRUE	FALSE	FALSE
NCBIGENE:1362	hgnc:2301	CPD	Up in BrM	GuideToPharmacology	2024.1	inhibitor	0.596678082	IUPHAR.LIGAND:7054	rxcui:677	AMINOGLUTETHIMIDE	TRUE	FALSE	TRUE
NCBIGENE:1362	hgnc:2301	CPD	Up in BrM	GuideToPharmacology	2024.1	inhibitor	0.367186512	IUPHAR.LIGAND:5137	rxcui:84857	ANASTROZOLE	TRUE	FALSE	TRUE
GGCX	hgnc:4247	GGCX	Up in BrM	ChEMBL	33	inhibitor	5.250767124	ANISINDIONE	rxcui:17941	ANISINDIONE	TRUE	FALSE	FALSE
NFKB1	hgnc:7794	NFKB1	Up in BrM	ClearityFoundationClinicalTrial	41440	inhibitor	0.006426888	BORTEZOMIB	rxcui:358258	BORTEZOMIB	TRUE	FALSE	TRUE
NCBIGENE:1611	hgnc:2672	DAP	Up in BrM	GuideToPharmacology	2024.1	inhibitor	1.591141553	IUPHAR.LIGAND:6492	ncit:C171870	CANDOXATRIL	TRUE	FALSE	FALSE
EML4	hgnc:1316	EML4	Up in BrM	ChEMBL	33	inhibitor	2.092697042	CERITINIB	rxcui:1535457	CERITINIB	TRUE	FALSE	TRUE
COL3A1	hgnc:2201	COL3A1	Up in BrM	ChEMBL	33	cleavage	2.812910959	COLLAGENASE CLOSTRIDIUM HISTOLYTICUM	rxcui:2475861	LAGENASE CLOSTRIDIUM HISTOLYTICUM-A	TRUE	FALSE	FALSE
COL1A2	hgnc:2198	COL1A2	Up in BrM	ChEMBL	33	cleavage	0.625091324	COLLAGENASE CLOSTRIDIUM HISTOLYTICUM	rxcui:2475861	LAGENASE CLOSTRIDIUM HISTOLYTICUM-A	TRUE	FALSE	FALSE
COL5A2	hgnc:2210	COL5A2	Up in BrM	ChEMBL	33	cleavage	0.937636986	COLLAGENASE CLOSTRIDIUM HISTOLYTICUM	rxcui:2475861	LAGENASE CLOSTRIDIUM HISTOLYTICUM-A	TRUE	FALSE	FALSE
EML4	hgnc:1316	EML4	Up in BrM	ChEMBL	33	inhibitor	2.634164109	CRIZOTINIB	rxcui:1148495	CRIZOTINIB	TRUE	FALSE	TRUE
NCBIGENE:1362	hgnc:2301	CPD	Up in BrM	GuideToPharmacology	2024.1	inhibitor	0.280789686	IUPHAR.LIGAND:7073	rxcui:258494	EXEMESTANE	TRUE	FALSE	TRUE
NCBIGENE:1942	hgnc:3221	EFNA1	Up in BrM	GuideToPharmacology	2024.1	inhibitor	0.144649232	IUPHAR.LIGAND:6912	rxcui:1442981	IBRUTINIB	TRUE	FALSE	TRUE
CYP51	hgnc:2649	CYP51A1	Up in BrM	ChEMBL	33	inhibitor	17.50255708	ISAVUCONAZONIUM SULFATE	rxcui:1608321	ISAVUCONAZONIUM SULFATE	TRUE	FALSE	FALSE
NCBIGENE:1362	hgnc:2301	CPD	Up in BrM	GuideToPharmacology	2024.1	inhibitor	0.265190259	IUPHAR.LIGAND:5209	rxcui:72965	LETROZOLE	TRUE	FALSE	TRUE
DPP4	hgnc:3009	DPP4	Up in BrM	ChEMBL	33	inhibitor	0.350051142	LINAGLIPTIN	rxcui:1100699	LINAGLIPTIN	TRUE	FALSE	FALSE
P4HB	hgnc:8548	P4HB	Up in BrM	ChEMBL	33	inhibitor	0.954684932	LOMITAPIDE MESYLATE	rxcui:1364478	LOMITAPIDE MESYLATE	TRUE	FALSE	FALSE

EML4	hgnc:1316	EML4	Up in BrM	ChEMBL	33	inhibitor	3.424413341	LORLATINIB	rxcui:2103164	LORLATINIB	TRUE	FALSE	TRUE
TGFB1	hgnc:11766	TGFB1	Up in BrM	ChEMBL	33	inhibitor	0.222490132	LUSPATERCEPT	rxcui:2262543	LUSPATERCEPT-AAMT	TRUE	FALSE	FALSE
NCBIGENE:1803	hgnc:3009	DPP4	Up in BrM	GuideToPharmacology	2024.1	inhibitor	0.061773731	IUPHAR.LIGAND:5656	ncit:C79910	MASITINIB	TRUE	FALSE	TRUE
NDUFA4L2	hgnc:29836	NDUFA4L2	Up in BrM	ChEMBL	33	inhibitor	0.312545662	METFORMIN HYDROCHLORIDE	rxcui:235743	METFORMIN HYDROCHLORIDE	TRUE	FALSE	TRUE
NCBIGENE:317	hgnc:576	APAF1	Up in BrM	GuideToPharmacology	2024.1	inhibitor	0.077444943	IUPHAR.LIGAND:7563	rxcui:29899	METHYLNALTREXONE	TRUE	FALSE	FALSE
NCBIGENE:317	hgnc:576	APAF1	Up in BrM	GuideToPharmacology	2024.1	inhibitor	0.116167414	IUPHAR.LIGAND:9150	rxcui:1876597	NALDEMEDINE	TRUE	FALSE	FALSE
NCBIGENE:317	hgnc:576	APAF1	Up in BrM	GuideToPharmacology	2024.1	inhibitor	0.027333509	IUPHAR.LIGAND:1638	rxcui:203192	NALOXONE HYDROCHLORIDE	TRUE	FALSE	FALSE
NCBIGENE:317	hgnc:576	APAF1	Up in BrM	GuideToPharmacology	2024.1	inhibitor	0.027333509	IUPHAR.LIGAND:1639	rxcui:7243	NALTREXONE	TRUE	FALSE	FALSE
NCBIGENE:1803	hgnc:3009	DPP4	Up in BrM	GuideToPharmacology	2024.1	inhibitor	0.042006137	IUPHAR.LIGAND:5936	rxcui:1592736	NINTEDANIB ESYLATE	TRUE	FALSE	TRUE
COL1A2	hgnc:2198	COL1A2	Up in BrM	ChEMBL	33	cleavage	0.153531202	OCRIPLASMIN	rxcui:29998	OCRIPLASMIN	TRUE	FALSE	FALSE
COL5A2	hgnc:2210	COL5A2	Up in BrM	ChEMBL	33	cleavage	0.690890411	OCRIPLASMIN	rxcui:29998	OCRIPLASMIN	TRUE	FALSE	FALSE
COL3A1	hgnc:2201	COL3A1	Up in BrM	ChEMBL	33	cleavage	0.690890411	OCRIPLASMIN	rxcui:29998	OCRIPLASMIN	TRUE	FALSE	FALSE
PRKD2	hgnc:17293	PRKD2	Up in BrM	MyCancerGenome	42906	inhibitor	0.019548649	SOPHORETIN	rxcui:9060	QUERCETIN	TRUE	FALSE	TRUE
NCBIGENE:1803	hgnc:3009	DPP4	Up in BrM	GuideToPharmacology	2024.1	inhibitor	0.055271233	IUPHAR.LIGAND:5658	rxcui:2643048	QUIZARTINIB	TRUE	FALSE	TRUE
NCBIGENE:317	hgnc:576	APAF1	Up in BrM	GuideToPharmacology	2024.1	inhibitor	0.077444943	IUPHAR.LIGAND:10651	rxcui:2559612	SAMIDORPHAN	TRUE	FALSE	FALSE
DPP4	hgnc:3009	DPP4	Up in BrM	ChEMBL	33	inhibitor	1.050153425	SAXAGLIPTIN HYDROCHLORIDE	rxcui:1043560	SAXAGLIPTIN HYDROCHLORIDE	TRUE	FALSE	FALSE
DPP4	hgnc:3009	DPP4	Up in BrM	ChEMBL	33	inhibitor	0.630092055	SITAGLIPTIN PHOSPHATE	rxcui:593411	SITAGLIPTIN	TRUE	FALSE	FALSE
NCBIGENE:1362	hgnc:2301	CPD	Up in BrM	GuideToPharmacology	2024.1	inhibitor	1.193356164	IUPHAR.LIGAND:7303	rxcui:10378	TESTOLACTONE	TRUE	FALSE	TRUE
TOP1MT	hgnc:29787	TOP1MT	Up in BrM	ChEMBL	33	inhibitor	0.84012274	TOPOTECAN HYDROCHLORIDE	rxcui:266573	TOPOTECAN HYDROCHLORIDE	TRUE	FALSE	TRUE
DPP4	hgnc:3009	DPP4	Up in BrM	ChEMBL	33	inhibitor	0.300043836	VILDAGLIPTIN	rxcui:596554	VILDAGLIPTIN	TRUE	FALSE	FALSE
NCBIGENE:2026	hgnc:3353	ENO2	Up in NSCLC	GuideToPharmacology	2024.1	inhibitor	0.16256245	IUPHAR.LIGAND:8912	rxcui:1986808	ACALABRUTINIB	TRUE	FALSE	FALSE
ATP1B1	hgnc:804	ATP1B1	Up in NSCLC	ChEMBL	33	inhibitor	0.307062405	ACETYLDIGITOXIN	rxcui:132871	ACETYLDIGITOXIN	TRUE	FALSE	FALSE
ATP1A3	hgnc:801	ATP1A3	Up in NSCLC	ChEMBL	33	inhibitor	0.364636606	ACETYLDIGITOXIN	rxcui:132871	ACETYLDIGITOXIN	TRUE	FALSE	FALSE
ATP1A2	hgnc:800	ATP1A2	Up in NSCLC	ChEMBL	33	inhibitor	0.833455099	ACETYLDIGITOXIN	rxcui:132871	ACETYLDIGITOXIN	TRUE	FALSE	FALSE
ATP1B2	hgnc:805	ATP1B2	Up in NSCLC	ChEMBL	33	inhibitor	0.972364282	ACETYLDIGITOXIN	rxcui:132871	ACETYLDIGITOXIN	TRUE	FALSE	FALSE
TUBB2A	hgnc:12412	TUBB2A	Up in NSCLC	ChEMBL	33	inhibitor	0.034727296	TRASTUZUMAB EMTANSINE	rxcui:1371041	ADO-TRASTUZUMAB EMTANSINE	TRUE	FALSE	TRUE
TUBB3	hgnc:20772	TUBB3	Up in NSCLC	ChEMBL	33	inhibitor	0.035574303	TRASTUZUMAB EMTANSINE	rxcui:1371041	ADO-TRASTUZUMAB EMTANSINE	TRUE	FALSE	TRUE
TUBB6	hgnc:20776	TUBB6	Up in NSCLC	ChEMBL	33	inhibitor	0.035574303	TRASTUZUMAB EMTANSINE	rxcui:1371041	ADO-TRASTUZUMAB EMTANSINE	TRUE	FALSE	TRUE
TUBB2B	hgnc:30829	TUBB2B	Up in NSCLC	ChEMBL	33	inhibitor	0.035574303	TRASTUZUMAB EMTANSINE	rxcui:1371041	ADO-TRASTUZUMAB EMTANSINE	TRUE	FALSE	TRUE
TUBB4B	hgnc:20771	TUBB4B	Up in NSCLC	ChEMBL	33	inhibitor	0.035145697	TRASTUZUMAB EMTANSINE	rxcui:1371041	ADO-TRASTUZUMAB EMTANSINE	TRUE	FALSE	TRUE
TUBA1B	hgnc:18809	TUBA1B	Up in NSCLC	ChEMBL	33	inhibitor	0.035574303	TRASTUZUMAB EMTANSINE	rxcui:1371041	ADO-TRASTUZUMAB EMTANSINE	TRUE	FALSE	TRUE
KCNA1	hgnc:6218	KCNA1	Up in NSCLC	ChEMBL	33	blocker	0.089298761	AMIFAMPRIDINE	rxcui:2106338	AMIFAMPRIDINE	TRUE	FALSE	FALSE
KCNA1	hgnc:6218	KCNA1	Up in NSCLC	ChEMBL	33	blocker	0.091476779	AMIFAMPRIDINE PHOSPHATE	rxcui:2106337	AMIFAMPRIDINE PHOSPHATE	TRUE	FALSE	FALSE
NCBIGENE:1921	hgnc:19679	AAK1	Up in NSCLC	GuideToPharmacology	2024.1	inhibitor	0.795570776	IUPHAR.LIGAND:7792	rxcui:2047232	BARICITINIB	TRUE	FALSE	FALSE
TUBA1B	hgnc:18809	TUBA1B	Up in NSCLC	ChEMBL	33	inhibitor	0.037666909	BELANTAMAB MAFODOTIN	rxcui:2387833	BELANTAMAB MAFODOTIN-BLMF	TRUE	FALSE	FALSE
TUBB4B	hgnc:20771	TUBB4B	Up in NSCLC	ChEMBL	33	inhibitor	0.037213091	BELANTAMAB MAFODOTIN	rxcui:2387833	BELANTAMAB MAFODOTIN-BLMF	TRUE	FALSE	FALSE
TUBB3	hgnc:20772	TUBB3	Up in NSCLC	ChEMBL	33	inhibitor	0.037666909	BELANTAMAB MAFODOTIN	rxcui:2387833	BELANTAMAB MAFODOTIN-BLMF	TRUE	FALSE	FALSE
TUBB2B	hgnc:30829	TUBB2B	Up in NSCLC	ChEMBL	33	inhibitor	0.037666909	BELANTAMAB MAFODOTIN	rxcui:2387833	BELANTAMAB MAFODOTIN-BLMF	TRUE	FALSE	FALSE
TUBB2A	hgnc:12412	TUBB2A	Up in NSCLC	ChEMBL	33	inhibitor	0.036770078	BELANTAMAB MAFODOTIN	rxcui:2387833	BELANTAMAB MAFODOTIN-BLMF	TRUE	FALSE	FALSE
TUBB6	hgnc:20776	TUBB6	Up in NSCLC	ChEMBL	33	inhibitor	0.037666909	BELANTAMAB MAFODOTIN	rxcui:2387833	BELANTAMAB MAFODOTIN-BLMF	TRUE	FALSE	FALSE
NCBIGENE:2026	hgnc:3353	ENO2	Up in NSCLC	GuideToPharmacology	2024.1	inhibitor	0.120154854	IUPHAR.LIGAND:5710	rxcui:1307619	BOSUTINIB	TRUE	FALSE	TRUE
NCBIGENE:1953	hgnc:3232	MEGF6	Up in NSCLC	GuideToPharmacology	2024.1	inhibitor	2.282942228	IUPHAR.LIGAND:5710	rxcui:1307619	BOSUTINIB	TRUE	FALSE	TRUE
TUBB4B	hgnc:20771	TUBB4B	Up in NSCLC	ChEMBL	33	inhibitor	0.035145697	BRENTUXIMAB VEDOTIN	rxcui:1147320	BRENTUXIMAB VEDOTIN	TRUE	FALSE	FALSE
TUBB2A	hgnc:12412	TUBB2A	Up in NSCLC	ChEMBL	33	inhibitor	0.034727296	BRENTUXIMAB VEDOTIN	rxcui:1147320	BRENTUXIMAB VEDOTIN	TRUE	FALSE	FALSE
TUBB3	hgnc:20772	TUBB3	Up in NSCLC	ChEMBL	33	inhibitor	0.035574303	BRENTUXIMAB VEDOTIN	rxcui:1147320	BRENTUXIMAB VEDOTIN	TRUE	FALSE	FALSE
TUBA1B	hgnc:18809	TUBA1B	Up in NSCLC	ChEMBL	33	inhibitor	0.035574303	BRENTUXIMAB VEDOTIN	rxcui:1147320	BRENTUXIMAB VEDOTIN	TRUE	FALSE	FALSE
TUBB2B	hgnc:30829	TUBB2B	Up in NSCLC	ChEMBL	33	inhibitor	0.035574303	BRENTUXIMAB VEDOTIN	rxcui:1147320	BRENTUXIMAB VEDOTIN	TRUE	FALSE	FALSE
TUBB6	hgnc:20776	TUBB6	Up in NSCLC	ChEMBL	33	inhibitor	0.035574303	BRENTUXIMAB VEDOTIN	rxcui:1147320	BRENTUXIMAB VEDOTIN	TRUE	FALSE	FALSE
TUBB2B	hgnc:30829	TUBB2B	Up in NSCLC	ChEMBL	33	inhibitor	0.029106248	CABAZITAXEL	rxcui:996051	CABAZITAXEL	TRUE	FALSE	TRUE
TUBB2A	hgnc:12412	TUBB2A	Up in NSCLC	ChEMBL	33	inhibitor	0.028413242	CABAZITAXEL	rxcui:996051	CABAZITAXEL	TRUE	FALSE	TRUE
TUBA1B	hgnc:18809	TUBA1B	Up in NSCLC	ChEMBL	33	inhibitor	0.029106248	CABAZITAXEL	rxcui:996051	CABAZITAXEL	TRUE	FALSE	TRUE
TUBB6	hgnc:20776	TUBB6	Up in NSCLC	ChEMBL	33	inhibitor	0.029106248	CABAZITAXEL	rxcui:996051	CABAZITAXEL	TRUE	FALSE	TRUE
TUBB4B	hgnc:20771	TUBB4B	Up in NSCLC	ChEMBL	33	inhibitor	0.02875557	CABAZITAXEL	rxcui:996051	CABAZITAXEL	TRUE	FALSE	TRUE
TUBB3	hgnc:20772	TUBB3	Up in NSCLC	ChEMBL	33	inhibitor	0.029106248	CABAZITAXEL	rxcui:996051	CABAZITAXEL	TRUE	FALSE	TRUE
NCBIGENE:18	hgnc:23	ABAT	Up in NSCLC	GuideToPharmacology	2024.1	inhibitor	0.016311796	IUPHAR.LIGAND:407	rxcui:1886	CAFFEINE	TRUE	FALSE	FALSE
NCBIGENE:526	hgnc:854	ATP6V1B2	Up in NSCLC	GuideToPharmacology	2024.1	blocker	4.37563927	IUPHAR.LIGAND:2503	rxcui:1362698	CHLOROFORM	TRUE	FALSE	FALSE
TUBB4B	hgnc:20771	TUBB4B	Up in NSCLC	ChEMBL	33	inhibitor	0.109492364	COLCHICINE	rxcui:2683	COLCHICINE	TRUE	FALSE	FALSE
TUBB2A	hgnc:12412	TUBB2A	Up in NSCLC	ChEMBL	33	inhibitor	0.108188883	COLCHICINE	rxcui:2683	COLCHICINE	TRUE	FALSE	FALSE
TUBA1B	hgnc:18809	TUBA1B	Up in NSCLC	ChEMBL	33	inhibitor	0.110827636	COLCHICINE	rxcui:2683	COLCHICINE	TRUE	FALSE	FALSE
TUBB6	hgnc:20776	TUBB6	Up in NSCLC	ChEMBL	33	inhibitor	0.110827636	COLCHICINE	rxcui:2683	COLCHICINE	TRUE	FALSE	FALSE
TUBB3	hgnc:20772	TUBB3	Up in NSCLC	ChEMBL	33	inhibitor	0.110827636	COLCHICINE	rxcui:2683	COLCHICINE	TRUE	FALSE	FALSE
TUBB2B	hgnc:30829	TUBB2B	Up in NSCLC	ChEMBL	33	inhibitor	0.110827636	COLCHICINE	rxcui:2683	COLCHICINE	TRUE	FALSE	FALSE
KCNA1	hgnc:6218	KCNA1	Up in NSCLC	ChEMBL	33	blocker	0.234409247	DALFAMPRIDINE	rxcui:897018	DALFAMPRIDINE	TRUE	FALSE	FALSE
FYN	hgnc:4037	FYN	Up in NSCLC	ChEMBL	33	inhibitor	0.075768645	DASATINIB	rxcui:1546019	DASATINIB ANHYDROUS	TRUE	FALSE	TRUE
ATP1A2	hgnc:800	ATP1A2	Up in NSCLC	ChEMBL	33	inhibitor	0.750109589	DESLANOSIDE	rxcui:3248	DESLANOSIDE	TRUE	FALSE	FALSE
ATP1A3	hgnc:801	ATP1A3	Up in NSCLC	ChEMBL	33	inhibitor	0.328172945	DESLANOSIDE	rxcui:3248	DESLANOSIDE	TRUE	FALSE	FALSE
ATP1B2	hgnc:805	ATP1B2	Up in NSCLC	ChEMBL	33	inhibitor	0.875127854	DESLANOSIDE	rxcui:3248	DESLANOSIDE	TRUE	FALSE	FALSE
ATP1B1	hgnc:804	ATP1B1	Up in NSCLC	ChEMBL	33	inhibitor	0.276356164	DESLANOSIDE	rxcui:3248	DESLANOSIDE	TRUE	FALSE	FALSE
ATP1A2	hgnc:800	ATP1A2	Up in NSCLC	ChEMBL	33	inhibitor	0.394794521	DIGITOXIN	rxcui:3403	DIGITOXIN	TRUE	FALSE	FALSE
ATP1A3	hgnc:801	ATP1A3	Up in NSCLC	ChEMBL	33	inhibitor	0.172722603	DIGITOXIN	rxcui:3403	DIGITOXIN	TRUE	FALSE	FALSE
ATP1B1	hgnc:804	ATP1B1	Up in NSCLC	ChEMBL	33	inhibitor	0.145450613	DIGITOXIN	rxcui:3403	DIGITOXIN	TRUE	FALSE	FALSE
ATP1B2	hgnc:805	ATP1B2	Up in NSCLC	ChEMBL	33	inhibitor	0.460593607	DIGITOXIN	rxcui:3403	DIGITOXIN	TRUE	FALSE	FALSE
ATP1B1	hgnc:804	ATP1B1	Up in NSCLC	ChEMBL	33	inhibitor	0.081281225	DIGOXIN	rxcui:3407	DIGOXIN	TRUE	FALSE	FALSE
ATP1B2	hgnc:805	ATP1B2	Up in NSCLC	ChEMBL	33	inhibitor	0.257390545	DIGOXIN	rxcui:3407	DIGOXIN	TRUE	FALSE	FALSE
ATP1A2	hgnc:800	ATP1A2	Up in NSCLC	ChEMBL	33	inhibitor	0.220620467	DIGOXIN	rxcui:3407	DIGOXIN	TRUE	FALSE	FALSE
ATP1A3	hgnc:801	ATP1A3	Up in NSCLC	ChEMBL	33	inhibitor	0.096521454	DIGOXIN	rxcui:3407	DIGOXIN	TRUE	FALSE	FALSE
TUBB4B	hgnc:20771	TUBB4B	Up in NSCLC	ChEMBL	33	inhibitor	0.007621958	DOCETAXEL	rxcui:1299922	DOCETAXEL ANHYDROUS	TRUE	FALSE	TRUE
TUBB6	hgnc:20776	TUBB6	Up in NSCLC	ChEMBL	33	inhibitor	0.007714909	DOCETAXEL	rxcui:1299922	DOCETAXEL ANHYDROUS	TRUE	FALSE	TRUE
TUBB2B	hgnc:30829	TUBB2B	Up in NSCLC	ChEMBL	33	inhibitor	0.007714909	DOCETAXEL	rxcui:1299922	DOCETAXEL ANHYDROUS	TRUE	FALSE	TRUE
TUBB2A	hgnc:12412	TUBB2A	Up in NSCLC	ChEMBL	33	inhibitor	0.007531221	DOCETAXEL	rxcui:1299922	DOCETAXEL ANHYDROUS	TRUE	FALSE	TRUE
TUBA1B	hgnc:18809	TUBA1B	Up in NSCLC	ChEMBL	33	inhibitor	0.007714909	DOCETAXEL	rxcui:1299922	DOCETAXEL ANHYDROUS	TRUE	FALSE	TRUE
TUBB3	hgnc:20772	TUBB3	Up in NSCLC	ChEMBL	33	inhibitor	0.015429818	DOCETAXEL	rxcui:1299922	DOCETAXEL ANHYDROUS	TRUE	FALSE	TRUE
GI	hgnc:4384	GNAI1	Up in NSCLC	ChEMBL	33	inhibitor	6.563458904	DOCOSANOL	rxcui:594680	DOCOSANOL	TRUE	FALSE	FALSE
TUBB6	hgnc:20776	TUBB6	Up in NSCLC	ChEMBL	33	inhibitor	0.037666909	ENFORTUMAB VEDOTIN	rxcui:2268306	ENFORTUMAB VEDOTIN-EJFV	TRUE	FALSE	TRUE
TUBB2A	hgnc:12412	TUBB2A	Up in NSCLC	ChEMBL	33	inhibitor	0.036770078	ENFORTUMAB VEDOTIN	rxcui:2268306	ENFORTUMAB VEDOTIN-EJFV	TRUE	FALSE	TRUE
TUBB2B	hgnc:30829	TUBB2B	Up in NSCLC	ChEMBL	33	inhibitor	0.037666909	ENFORTUMAB VEDOTIN	rxcui:2268306	ENFORTUMAB VEDOTIN-EJFV	TRUE	FALSE	TRUE
TUBB3	hgnc:20772	TUBB3	Up in NSCLC	ChEMBL	33	inhibitor	0.037666909	ENFORTUMAB VEDOTIN	rxcui:2268306	ENFORTUMAB VEDOTIN-EJFV	TRUE	FALSE	TRUE
TUBB4B	hgnc:20771	TUBB4B	Up in NSCLC	ChEMBL	33	inhibitor	0.037213091	ENFORTUMAB VEDOTIN	rxcui:2268306	ENFORTUMAB VEDOTIN-EJFV	TRUE	FALSE	TRUE
TUBA1B	hgnc:18809	TUBA1B	Up in NSCLC	ChEMBL	33	inhibitor	0.037666909	ENFORTUMAB VEDOTIN	rxcui:2268306	ENFORTUMAB VEDOTIN-EJFV	TRUE	FALSE	TRUE
NCBIGENE:812	hgnc:854	ATP6V1B2	Up in NSCLC	GuideToPharmacology	2024.1	inhibitor	5.834185693	IUPHAR.LIGAND:8882	rxcui:3925	ENOXACIN	TRUE	FALSE	FALSE

NCBIGENE:1808	hgnc:3014	DPYSL2	Up in NSCLC	GuideToPharmacology	2024.1	inhibitor	0.115148402	IUPHAR.LIGAND:9039	rxcui:2123125	ERDAFITINIB	TRUE	FALSE	TRUE
TUBB2A	hgnc:12412	TUBB2A	Up in NSCLC	ChEMBL	33	inhibitor	0.025003653	ERIBULIN MESYLATE	rxcui:1045452	ERIBULIN MESYLATE	TRUE	FALSE	TRUE
TUBB4B	hgnc:20771	TUBB4B	Up in NSCLC	ChEMBL	33	inhibitor	0.025304902	ERIBULIN MESYLATE	rxcui:1045452	ERIBULIN MESYLATE	TRUE	FALSE	TRUE
TUBB2B	hgnc:30829	TUBB2B	Up in NSCLC	ChEMBL	33	inhibitor	0.025613498	ERIBULIN MESYLATE	rxcui:1045452	ERIBULIN MESYLATE	TRUE	FALSE	TRUE
TUBB6	hgnc:20776	TUBB6	Up in NSCLC	ChEMBL	33	inhibitor	0.025613498	ERIBULIN MESYLATE	rxcui:1045452	ERIBULIN MESYLATE	TRUE	FALSE	TRUE
TUBA1B	hgnc:18809	TUBA1B	Up in NSCLC	ChEMBL	33	inhibitor	0.025613498	ERIBULIN MESYLATE	rxcui:1045452	ERIBULIN MESYLATE	TRUE	FALSE	TRUE
TUBB3	hgnc:20772	TUBB3	Up in NSCLC	ChEMBL	33	inhibitor	0.025613498	ERIBULIN MESYLATE	rxcui:1045452	ERIBULIN MESYLATE	TRUE	FALSE	TRUE
GABRA1	hgnc:4075	GABRA1	Up in NSCLC	ChEMBL	33	negative modulator	0.067839369	FLUMAZENIL	rxcui:4457	FLUMAZENIL	TRUE	FALSE	FALSE
GABRB2	hgnc:4082	GABRB2	Up in NSCLC	ChEMBL	33	negative modulator	0.025439763	FLUMAZENIL	rxcui:4457	FLUMAZENIL	TRUE	FALSE	FALSE
NCBIGENE:1808	hgnc:3014	DPYSL2	Up in NSCLC	GuideToPharmacology	2024.1	inhibitor	0.125616438	IUPHAR.LIGAND:9786	rxcui:2628190	FUTIBATINIB	TRUE	FALSE	TRUE
NCBIGENE:1837	hgnc:3057	DTNA	Up in NSCLC	GuideToPharmacology	2024.1	inhibitor	0.184237443	IUPHAR.LIGAND:8708	rxcui:2105806	GILTERITINIB	TRUE	FALSE	TRUE
KCNA1	hgnc:6218	KCNA1	Up in NSCLC	ChEMBL	33	blocker	0.091476779	GUANIDINE HYDROCHLORIDE	rxcui:50676	GUANIDINE HYDROCHLORIDE	TRUE	FALSE	FALSE
NCBIGENE:523	hgnc:851	ATP6V1A	Up in NSCLC	GuideToPharmacology	2024.1	inhibitor	1.458546423	IUPHAR.LIGAND:2401	rxcui:5095	HALOTHANE	TRUE	FALSE	FALSE
NCBIGENE:1759	hgnc:2972	DNM1	Up in NSCLC	GuideToPharmacology	2024.1	inhibitor	1.458546423	IUPHAR.LIGAND:7198	rxcui:5521	HYDROXYCHLOROQUINE	TRUE	FALSE	FALSE
NCBIGENE:2026	hgnc:3353	ENO2	Up in NSCLC	GuideToPharmacology	2024.1	inhibitor	0.083744292	IUPHAR.LIGAND:6912	rxcui:1442981	IBRUTINIB	TRUE	FALSE	TRUE
NCBIGENE:1808	hgnc:3014	DPYSL2	Up in NSCLC	GuideToPharmacology	2024.1	inhibitor	0.051177067	IUPHAR.LIGAND:7877	rxcui:2550729	INFIGRATINIB	TRUE	FALSE	TRUE
NCBIGENE:529	hgnc:857	ATP6V1E1	Up in NSCLC	GuideToPharmacology	2024.1	inhibitor	0.486182141	IUPHAR.LIGAND:2733	rxcui:33910	ISRADIPINE	TRUE	FALSE	FALSE
NCBIGENE:528	hgnc:856	ATP6V1C1	Up in NSCLC	GuideToPharmacology	2024.1	inhibitor	0.875127854	IUPHAR.LIGAND:2733	rxcui:33910	ISRADIPINE	TRUE	FALSE	FALSE
NCBIGENE:18	hgnc:23	ABAT	Up in NSCLC	GuideToPharmacology	2024.1	inhibitor	0.12070729	IUPHAR.LIGAND:5608	rxcui:2199015	ISTRADEFYLLINE	TRUE	FALSE	FALSE
TUBB2B	hgnc:30829	TUBB2B	Up in NSCLC	ChEMBL	33	inhibitor	0.029106248	IXABEPILONE	rxcui:337523	IXABEPILONE	TRUE	FALSE	FALSE
TUBB4B	hgnc:20771	TUBB4B	Up in NSCLC	ChEMBL	33	inhibitor	0.02875557	IXABEPILONE	rxcui:337523	IXABEPILONE	TRUE	FALSE	FALSE
TUBB6	hgnc:20776	TUBB6	Up in NSCLC	ChEMBL	33	inhibitor	0.029106248	IXABEPILONE	rxcui:337523	IXABEPILONE	TRUE	FALSE	FALSE
TUBB2A	hgnc:12412	TUBB2A	Up in NSCLC	ChEMBL	33	inhibitor	0.028413242	IXABEPILONE	rxcui:337523	IXABEPILONE	TRUE	FALSE	FALSE
TUBB3	hgnc:20772	TUBB3	Up in NSCLC	ChEMBL	33	inhibitor	0.058212496	IXABEPILONE	rxcui:337523	IXABEPILONE	TRUE	FALSE	FALSE
TUBA1B	hgnc:18809	TUBA1B	Up in NSCLC	ChEMBL	33	inhibitor	0.029106248	IXABEPILONE	rxcui:337523	IXABEPILONE	TRUE	FALSE	FALSE
ATP1A3	hgnc:801	ATP1A3	Up in NSCLC	ChEMBL	33	inhibitor	0.218781963	LANATOSIDE C	rxcui:27929	LANATOSIDE C	TRUE	FALSE	FALSE
ATP1B1	hgnc:804	ATP1B1	Up in NSCLC	ChEMBL	33	inhibitor	0.184237443	LANATOSIDE C	rxcui:27929	LANATOSIDE C	TRUE	FALSE	FALSE
ATP1B2	hgnc:805	ATP1B2	Up in NSCLC	ChEMBL	33	inhibitor	0.583418569	LANATOSIDE C	rxcui:27929	LANATOSIDE C	TRUE	FALSE	FALSE
ATP1A2	hgnc:800	ATP1A2	Up in NSCLC	ChEMBL	33	inhibitor	0.500073059	LANATOSIDE C	rxcui:27929	LANATOSIDE C	TRUE	FALSE	FALSE
IMPA1	hgnc:6050	IMPA1	Up in NSCLC	ChEMBL	33	inhibitor	5.250767124	LITHIUM CARBONATE	rxcui:42351	LITHIUM CARBONATE	TRUE	FALSE	FALSE
IMPA1	hgnc:6050	IMPA1	Up in NSCLC	ChEMBL	33	inhibitor	8.751278539	LITHIUM CITRATE	rxcui:52105	LITHIUM CITRATE	TRUE	FALSE	FALSE
NCBIGENE:18	hgnc:23	ABAT	Up in NSCLC	GuideToPharmacology	2024.1	inhibitor	0.201178817	IUPHAR.LIGAND:4252	rxcui:6694	MEFLOQUINE	TRUE	FALSE	FALSE
TUBB2B	hgnc:30829	TUBB2B	Up in NSCLC	ChEMBL	33	inhibitor	0.040021091	MIRVETUXIMAB SORAVTANSINE	rxcui:2621548	MIRVETUXIMAB SORAVTANSINE	TRUE	FALSE	FALSE
TUBB6	hgnc:20776	TUBB6	Up in NSCLC	ChEMBL	33	inhibitor	0.040021091	MIRVETUXIMAB SORAVTANSINE	rxcui:2621548	MIRVETUXIMAB SORAVTANSINE	TRUE	FALSE	FALSE
TUBB4B	hgnc:20771	TUBB4B	Up in NSCLC	ChEMBL	33	inhibitor	0.039538909	MIRVETUXIMAB SORAVTANSINE	rxcui:2621548	MIRVETUXIMAB SORAVTANSINE	TRUE	FALSE	FALSE
TUBB3	hgnc:20772	TUBB3	Up in NSCLC	ChEMBL	33	inhibitor	0.040021091	MIRVETUXIMAB SORAVTANSINE	rxcui:2621548	MIRVETUXIMAB SORAVTANSINE	TRUE	FALSE	FALSE
TUBA1B	hgnc:18809	TUBA1B	Up in NSCLC	ChEMBL	33	inhibitor	0.040021091	MIRVETUXIMAB SORAVTANSINE	rxcui:2621548	MIRVETUXIMAB SORAVTANSINE	TRUE	FALSE	FALSE
TUBB2A	hgnc:12412	TUBB2A	Up in NSCLC	ChEMBL	33	inhibitor	0.039068208	MIRVETUXIMAB SORAVTANSINE	rxcui:2621548	MIRVETUXIMAB SORAVTANSINE	TRUE	FALSE	FALSE
NCBIGENE:528	hgnc:856	ATP6V1C1	Up in NSCLC	GuideToPharmacology	2024.1	inhibitor	0.552712329	IUPHAR.LIGAND:2523	rxcui:7426	NIMODIPINE	TRUE	FALSE	FALSE
NCBIGENE:1808	hgnc:3014	DPYSL2	Up in NSCLC	GuideToPharmacology	2024.1	inhibitor	0.055271233	IUPHAR.LIGAND:5936	rxcui:1592736	NINTEDANIB ESYLATE	TRUE	FALSE	TRUE
NCBIGENE:529	hgnc:857	ATP6V1E1	Up in NSCLC	GuideToPharmacology	2024.1	inhibitor	0.343187394	IUPHAR.LIGAND:2524	rxcui:7435	NISOLDIPINE	TRUE	FALSE	FALSE
NCBIGENE:491	hgnc:815	ATP2B2	Up in NSCLC	GuideToPharmacology	2024.1	blocker	0.308868654	IUPHAR.LIGAND:7091	rxcui:31994	NORGESTIMATE	TRUE	FALSE	FALSE
TUBA1B	hgnc:18809	TUBA1B	Up in NSCLC	ChEMBL	33	inhibitor	0.077077657	PACLITAXEL	rxcui:56946	PACLITAXEL	TRUE	FALSE	TRUE
TUBB3	hgnc:20772	TUBB3	Up in NSCLC	ChEMBL	33	inhibitor	0.088935758	PACLITAXEL	rxcui:56946	PACLITAXEL	TRUE	FALSE	TRUE
TUBB2B	hgnc:30829	TUBB2B	Up in NSCLC	ChEMBL	33	inhibitor	0.077077657	PACLITAXEL	rxcui:56946	PACLITAXEL	TRUE	FALSE	TRUE
TUBB4B	hgnc:20771	TUBB4B	Up in NSCLC	ChEMBL	33	inhibitor	0.07614901	PACLITAXEL	rxcui:56946	PACLITAXEL	TRUE	FALSE	TRUE
TUBB2A	hgnc:12412	TUBB2A	Up in NSCLC	ChEMBL	33	inhibitor	0.086818239	PACLITAXEL	rxcui:56946	PACLITAXEL	TRUE	FALSE	TRUE
TUBB6	hgnc:20776	TUBB6	Up in NSCLC	ChEMBL	33	inhibitor	0.077077657	PACLITAXEL	rxcui:56946	PACLITAXEL	TRUE	FALSE	TRUE
SIRT2	hgnc:10886	SIRT2	Up in NSCLC	TALC	42502	inhibitor	0.132595129	PANOBINOSTAT	rxcui:1603350	PANOBINOSTAT	TRUE	FALSE	TRUE
NCBIGENE:481	hgnc:804	ATP1B1	Up in NSCLC	GuideToPharmacology	2024.1	inhibitor	0.110542466	IUPHAR.LIGAND:4790	rxcui:1298842	AROXETINE HYDROCHLORIDE, HEMIHYDRA	TRUE	FALSE	FALSE
NCBIGENE:1808	hgnc:3014	DPYSL2	Up in NSCLC	GuideToPharmacology	2024.1	inhibitor	0.023823807	IUPHAR.LIGAND:5698	rxcui:714438	PAZOPANIB	TRUE	FALSE	TRUE
NCBIGENE:1808	hgnc:3014	DPYSL2	Up in NSCLC	GuideToPharmacology	2024.1	inhibitor	0.230296804	IUPHAR.LIGAND:9767	rxcui:2359268	PEMIGATINIB	TRUE	FALSE	TRUE
TUBB4B	hgnc:20771	TUBB4B	Up in NSCLC	ChEMBL	33	inhibitor	0.033295923	POLATUZUMAB VEDOTIN	rxcui:2174090	POLATUZUMAB VEDOTIN	TRUE	FALSE	FALSE
TUBB3	hgnc:20772	TUBB3	Up in NSCLC	ChEMBL	33	inhibitor	0.033701971	POLATUZUMAB VEDOTIN	rxcui:2174090	POLATUZUMAB VEDOTIN	TRUE	FALSE	FALSE
TUBB2B	hgnc:30829	TUBB2B	Up in NSCLC	ChEMBL	33	inhibitor	0.033701971	POLATUZUMAB VEDOTIN	rxcui:2174090	POLATUZUMAB VEDOTIN	TRUE	FALSE	FALSE
TUBA1B	hgnc:18809	TUBA1B	Up in NSCLC	ChEMBL	33	inhibitor	0.033701971	POLATUZUMAB VEDOTIN	rxcui:2174090	POLATUZUMAB VEDOTIN	TRUE	FALSE	FALSE
TUBB2A	hgnc:12412	TUBB2A	Up in NSCLC	ChEMBL	33	inhibitor	0.032899543	POLATUZUMAB VEDOTIN	rxcui:2174090	POLATUZUMAB VEDOTIN	TRUE	FALSE	FALSE
TUBB6	hgnc:20776	TUBB6	Up in NSCLC	ChEMBL	33	inhibitor	0.033701971	POLATUZUMAB VEDOTIN	rxcui:2174090	POLATUZUMAB VEDOTIN	TRUE	FALSE	FALSE
BCHE	hgnc:983	BCHE	Up in NSCLC	ChEMBL	33	inhibitor	0.65634589	PROPANIDID	rxcui:8758	PROPANIDID	TRUE	FALSE	FALSE
BCHE	hgnc:983	BCHE	Up in NSCLC	ChEMBL	33	inhibitor	0.562582192	RIVASTIGMINE	rxcui:183379	RIVASTIGMINE	TRUE	FALSE	FALSE
BCHE	hgnc:983	BCHE	Up in NSCLC	ChEMBL	33	inhibitor	0.65634589	RIVASTIGMINE TARTRATE	rxcui:994808	RIVASTIGMINE TARTRATE	TRUE	FALSE	FALSE
NCBIGENE:1808	hgnc:3014	DPYSL2	Up in NSCLC	GuideToPharmacology	2024.1	inhibitor	0.012120884	IUPHAR.LIGAND:5711	rxcui:495881	SORAFENIB	TRUE	FALSE	TRUE
NCBIGENE:610	hgnc:4846	HCN2	Up in NSCLC	GuideToPharmacology	2024.1	inhibitor	0.192335792	IUPHAR.LIGAND:5425	rxcui:10237	SULINDAC	TRUE	FALSE	TRUE
NCBIGENE:1808	hgnc:3014	DPYSL2	Up in NSCLC	GuideToPharmacology	2024.1	inhibitor	0.028199609	IUPHAR.LIGAND:5713	rxcui:357977	SUNITINIB	TRUE	FALSE	TRUE
BCHE	hgnc:983	BCHE	Up in NSCLC	ChEMBL	33	inhibitor	0.65634589	TACRINE HYDROCHLORIDE	rxcui:235972	TACRINE HYDROCHLORIDE	TRUE	FALSE	FALSE
NCBIGENE:18	hgnc:23	ABAT	Up in NSCLC	GuideToPharmacology	2024.1	inhibitor	0.022353202	IUPHAR.LIGAND:413	rxcui:10438	THEOPHYLLINE	TRUE	FALSE	FALSE
TUBB2B	hgnc:30829	TUBB2B	Up in NSCLC	ChEMBL	33	inhibitor	0.040021091	TISOTUMAB VEDOTIN	rxcui:2571093	TISOTUMAB VEDOTIN-TFTV	TRUE	FALSE	FALSE
TUBB2A	hgnc:12412	TUBB2A	Up in NSCLC	ChEMBL	33	inhibitor	0.039068208	TISOTUMAB VEDOTIN	rxcui:2571093	TISOTUMAB VEDOTIN-TFTV	TRUE	FALSE	FALSE
TUBB6	hgnc:20776	TUBB6	Up in NSCLC	ChEMBL	33	inhibitor	0.040021091	TISOTUMAB VEDOTIN	rxcui:2571093	TISOTUMAB VEDOTIN-TFTV	TRUE	FALSE	FALSE
TUBB4B	hgnc:20771	TUBB4B	Up in NSCLC	ChEMBL	33	inhibitor	0.039538909	TISOTUMAB VEDOTIN	rxcui:2571093	TISOTUMAB VEDOTIN-TFTV	TRUE	FALSE	FALSE
TUBA1B	hgnc:18809	TUBA1B	Up in NSCLC	ChEMBL	33	inhibitor	0.040021091	TISOTUMAB VEDOTIN	rxcui:2571093	TISOTUMAB VEDOTIN-TFTV	TRUE	FALSE	FALSE
TUBB3	hgnc:20772	TUBB3	Up in NSCLC	ChEMBL	33	inhibitor	0.040021091	TISOTUMAB VEDOTIN	rxcui:2571093	TISOTUMAB VEDOTIN-TFTV	TRUE	FALSE	FALSE
ABAT	hgnc:23	ABAT	Up in NSCLC	ChEMBL	33	inhibitor	0.603536451	VIGABATRIN	rxcui:14851	VIGABATRIN	TRUE	FALSE	FALSE
TUBB4B	hgnc:20771	TUBB4B	Up in NSCLC	ChEMBL	33	inhibitor	0.031631127	VINBLASTINE SULFATE	rxcui:11199	VINBLASTINE SULFATE	TRUE	FALSE	FALSE
TUBB6	hgnc:20776	TUBB6	Up in NSCLC	ChEMBL	33	inhibitor	0.032016873	VINBLASTINE SULFATE	rxcui:11199	VINBLASTINE SULFATE	TRUE	FALSE	FALSE
TUBB3	hgnc:20772	TUBB3	Up in NSCLC	ChEMBL	33	inhibitor	0.032016873	VINBLASTINE SULFATE	rxcui:11199	VINBLASTINE SULFATE	TRUE	FALSE	FALSE
TUBB2B	hgnc:30829	TUBB2B	Up in NSCLC	ChEMBL	33	inhibitor	0.032016873	VINBLASTINE SULFATE	rxcui:11199	VINBLASTINE SULFATE	TRUE	FALSE	FALSE
TUBA1B	hgnc:18809	TUBA1B	Up in NSCLC	ChEMBL	33	inhibitor	0.032016873	VINBLASTINE SULFATE	rxcui:11199	VINBLASTINE SULFATE	TRUE	FALSE	FALSE
TUBB2A	hgnc:12412	TUBB2A	Up in NSCLC	ChEMBL	33	inhibitor	0.031254566	VINBLASTINE SULFATE	rxcui:11199	VINBLASTINE SULFATE	TRUE	FALSE	FALSE
TUBB4B	hgnc:20771	TUBB4B	Up in NSCLC	ChEMBL	33	inhibitor	0.033295923	VINCRISTINE SULFATE	rxcui:11203	VINCRISTINE SULFATE	TRUE	FALSE	FALSE
TUBB2B	hgnc:30829	TUBB2B	Up in NSCLC	ChEMBL	33	inhibitor	0.033701971	VINCRISTINE SULFATE	rxcui:11203	VINCRISTINE SULFATE	TRUE	FALSE	FALSE
TUBA1B	hgnc:18809	TUBA1B	Up in NSCLC	ChEMBL	33	inhibitor	0.033701971	VINCRISTINE SULFATE	rxcui:11203	VINCRISTINE SULFATE	TRUE	FALSE	FALSE
TUBB2A	hgnc:12412	TUBB2A	Up in NSCLC	ChEMBL	33	inhibitor	0.032899543	VINCRISTINE SULFATE	rxcui:11203	VINCRISTINE SULFATE	TRUE	FALSE	FALSE
TUBB6	hgnc:20776	TUBB6	Up in NSCLC	ChEMBL	33	inhibitor	0.033701971	VINCRISTINE SULFATE	rxcui:11203	VINCRISTINE SULFATE	TRUE	FALSE	FALSE
TUBB3	hgnc:20772	TUBB3	Up in NSCLC	ChEMBL	33	inhibitor	0.033701971	VINCRISTINE SULFATE	rxcui:11203	VINCRISTINE SULFATE	TRUE	FALSE	FALSE
TUBB2B	hgnc:30829	TUBB2B	Up in NSCLC	ChEMBL	33	inhibitor	0.042689164	VINFLUNINE	ncit:C61564	VINFLUNINE	TRUE	FALSE	FALSE
TUBB6	hgnc:20776	TUBB6	Up in NSCLC	ChEMBL	33	inhibitor	0.042689164	VINFLUNINE	ncit:C61564	VINFLUNINE	TRUE	FALSE	FALSE
TUBB3	hgnc:20772	TUBB3	Up in NSCLC	ChEMBL	33	inhibitor	0.042689164	VINFLUNINE	ncit:C61564	VINFLUNINE	TRUE	FALSE	FALSE
TUBB2A	hgnc:12412	TUBB2A	Up in NSCLC	ChEMBL	33	inhibitor	0.041672755	VINFLUNINE	ncit:C61564	VINFLUNINE	TRUE	FALSE	FALSE
TUBA1B	hgnc:18809	TUBA1B	Up in NSCLC	ChEMBL	33	inhibitor	0.042689164	VINFLUNINE	ncit:C61564	VINFLUNINE	TRUE	FALSE	FALSE

TUBB4B	hgnc:20771	TUBB4B	Up in NSCLC	ChEMBL	33	inhibitor	0.042174836	VINFLUNINE	ncit:C61564	VINFLUNINE	TRUE	FALSE	FALSE
TUBB2B	hgnc:30829	TUBB2B	Up in NSCLC	ChEMBL	33	inhibitor	0.042689164	VINORELBINE TARTRATE	rxcui:114527	VINORELBINE TARTRATE	TRUE	FALSE	FALSE
TUBA1B	hgnc:18809	TUBA1B	Up in NSCLC	ChEMBL	33	inhibitor	0.042689164	VINORELBINE TARTRATE	rxcui:114527	VINORELBINE TARTRATE	TRUE	FALSE	FALSE
TUBB4B	hgnc:20771	TUBB4B	Up in NSCLC	ChEMBL	33	inhibitor	0.042174836	VINORELBINE TARTRATE	rxcui:114527	VINORELBINE TARTRATE	TRUE	FALSE	FALSE
TUBB6	hgnc:20776	TUBB6	Up in NSCLC	ChEMBL	33	inhibitor	0.042689164	VINORELBINE TARTRATE	rxcui:114527	VINORELBINE TARTRATE	TRUE	FALSE	FALSE
TUBB3	hgnc:20772	TUBB3	Up in NSCLC	ChEMBL	33	inhibitor	0.042689164	VINORELBINE TARTRATE	rxcui:114527	VINORELBINE TARTRATE	TRUE	FALSE	FALSE
TUBB2A	hgnc:12412	TUBB2A	Up in NSCLC	ChEMBL	33	inhibitor	0.041672755	VINORELBINE TARTRATE	rxcui:114527	VINORELBINE TARTRATE	TRUE	FALSE	FALSE

plementary table 6 Paired serum-tissue proteomic data and correlation coefficients of protein express

Uniprot	Gene Name	rho	*p* -value	Group	FDR
Q9Y6W5	WASF2	-0.117635547	0.850567932	stable	0.990207998
Q9Y6W3	CAPN7	-0.898804814	0.038051266	negative	0.988954333
Q9Y6R7	FCGBP	-0.047473738	0.939577272	stable	0.990577889
Q9Y6R4	MAP3K4	0.364659282	0.546207189	stable	0.988954333
Q9Y6N7	ROBO1	0.17184122	0.782286599	stable	0.988954333
Q9Y6M4	CSNK1G3	0.698833244	0.189182155	stable	0.988954333
Q9Y6M1	IGF2BP2	0.502123033	0.3886629	stable	0.988954333
Q9Y6E2	BZW2	0.226892628	0.713609513	stable	0.988954333
Q9Y6E0	STK24	-0.197488448	0.750194092	stable	0.988954333
Q9Y6C2	EMILIN1	-0.713145302	0.176276881	stable	0.988954333
Q9Y6B6	SAR1B	0.982907647	0.002675593	positive	0.988954333
Q9Y696	CLIC4	-0.020434417	0.973983903	stable	0.994727102
Q9Y646	CPQ	-0.597063667	0.28775201	stable	0.988954333
Q9Y639	NPTN	0.492827137	0.398930223	stable	0.988954333
Q9Y624	F11R	0.595280053	0.289575272	stable	0.988954333
Q9Y613	FHOD1	0.181325697	0.770400402	stable	0.988954333
Q9Y608	LRRFIP2	-0.638243565	0.246524569	stable	0.988954333
Q9Y5X3	SNX5	-0.329490372	0.588199295	stable	0.988954333
Q9Y5S9	RBM8A	-0.231260566	0.708195973	stable	0.988954333
Q9Y5S2	CDC42BPB	0.226102421	0.714589488	stable	0.988954333
Q9Y5L0	TNPO3	0.74997516	0.144314533	stable	0.988954333
Q9Y5K8	ATP6V1D	0.297338222	0.627072056	stable	0.988954333
Q9Y5K6	CD2AP	-0.169125986	0.78569313	stable	0.988954333
Q9Y5A7	NUB1	0.142470088	0.819216995	stable	0.990207998
Q9Y520	PRRC2C	0.902447539	0.03603562	positive	0.988954333
Q9Y4Z0	LSM4	-0.116808037	0.851614286	stable	0.990207998
Q9Y4X5	ARIH1	0.738029151	0.154477292	stable	0.988954333
Q9Y4L1	HYOU1	0.354703157	0.558035283	stable	0.988954333
Q9Y4I1	MYO5A	0.576815607	0.30862397	stable	0.988954333
Q9Y4G8	RAPGEF2	0.107166941	0.863812445	stable	0.990207998
Q9Y4G6	TLN2	0.104053284	0.867754705	stable	0.990207998
Q9Y4E8	USP15	-0.803013845	0.101793368	stable	0.988954333
Q9Y4D8	HECTD4	0.465302375	0.429690912	stable	0.988954333
Q9Y4D1	DAAM1	0.412467357	0.490126599	stable	0.988954333
Q9Y490	TLN1	0.197615553	0.750035447	stable	0.988954333
Q9Y487	ATP6V0A2	3.59E-05	0.999954289	stable	0.999973019
Q9Y450	HBS1L	-0.587748253	0.297307421	stable	0.988954333
Q9Y3Z3	SAMHD1	-0.47183991	0.422337432	stable	0.988954333
Q9Y3X0	CCDC9	-0.273949147	0.655610427	stable	0.988954333
Q9Y3L3	SH3BP1	0.553210197	0.333420851	stable	0.988954333
Q9Y3I1	FBXO7	-0.321524146	0.59778979	stable	0.988954333
Q9Y3I0	RTCB	-0.147949959	0.812313768	stable	0.988954333
Q9Y3E7	CHMP3	0.586797943	0.298286759	stable	0.988954333
Q9Y3E1	HDGFL3	0.11543995	0.853344404	stable	0.990207998
Q9Y3C8	UFC1	-0.068074043	0.913392425	stable	0.990207998
Q9Y3C5	RNF11	0.250038601	0.684990055	stable	0.988954333
Q9Y3A5	SBDS	0.17012945	0.784433995	stable	0.988954333
Q9Y383	LUC7L2	-0.492424662	0.399376175	stable	0.988954333
Q9Y376	CAB39	-0.172171491	0.781872352	stable	0.988954333
Q9Y333	LSM2	-0.130556974	0.834243149	stable	0.990207998
Q9Y315	DERA	0.339514736	0.576171182	stable	0.988954333
Q9Y2X7	GIT1	0.406422868	0.497147988	stable	0.988954333
Q9Y2W1	THRAP3	-0.099701407	0.873266852	stable	0.990207998
Q9Y2V2	CARHSP1	0.323682642	0.595188447	stable	0.988954333
Q9Y2T2	AP3M1	-0.200184289	0.74683018	stable	0.988954333
Q9Y2Q5	LAMTOR2	0.429992681	0.469889272	stable	0.988954333

Q9Y2J2	EPB41L3	0.616776527	0.267804914	stable	0.988954333
Q9Y2I8	WDR37	0.085870243	0.890801125	stable	0.990207998
Q9Y2A7	NCKAP1	0.300752127	0.62292425	stable	0.988954333
Q9Y295	DRG1	0.955174337	0.011315717	positive	0.988954333
Q9Y285	FARSA	-0.211118133	0.733206416	stable	0.988954333
Q9Y281	CFL2	-0.353136153	0.559901319	stable	0.988954333
Q9Y274	ST3GAL6	-0.359244116	0.552634493	stable	0.988954333
Q9Y266	NUDC	-0.63409464	0.25060033	stable	0.988954333
Q9Y262	EIF3L	0.262934059	0.669120424	stable	0.988954333
Q9Y241	HIGD1A	-0.456074848	0.440118685	stable	0.988954333
Q9Y237	PIN4	-0.358913772	0.553027048	stable	0.988954333
Q9Y224	RTRAF	-0.454479763	0.441926919	stable	0.988954333
Q9UQN3	CHMP2B	0.518945112	0.370246407	stable	0.988954333
Q9UQE7	SMC3	-0.115552272	0.853202348	stable	0.990207998
Q9UQ80	PA2G4	0.264287266	0.667458411	stable	0.988954333
Q9UQ35	SRRM2	-0.159822189	0.797377723	stable	0.988954333
Q9UQ16	DNM3	-0.542938024	0.344360366	stable	0.988954333
Q9UQ13	SHOC2	-0.459861476	0.435832719	stable	0.988954333
Q9UPT5	EXOC7	-0.165392616	0.790379639	stable	0.988954333
Q9UPN3	MACF1	-0.132746872	0.831479153	stable	0.990207998
Q9UNZ2	NSFL1C	-0.760172319	0.13580228	stable	0.988954333
Q9UNW1	MINPP1	-0.141092399	0.820953403	stable	0.990207998
Q9UNM6	PSMD13	0.525504415	0.363124152	stable	0.988954333
Q9UNH7	SNX6	0.419283982	0.482233574	stable	0.988954333
Q9UNF0	PACSIN2	-0.200395969	0.746566121	stable	0.988954333
Q9UN86	G3BP2	-0.758271655	0.137377292	stable	0.988954333
Q9UN37	VPS4A	-0.027776373	0.964638572	stable	0.99196576
Q9UMZ2	SYNRG	-0.290994836	0.634791333	stable	0.988954333
Q9ULV4	CORO1C	0.39909737	0.50568512	stable	0.988954333
Q9ULH1	ASAP1	-0.429502534	0.470452781	stable	0.988954333
Q9ULF5	SLC39A10	-0.412916052	0.489606222	stable	0.988954333
Q9ULC4	MCTS1	0.565429763	0.320523828	stable	0.988954333
Q9UL46	PSME2	0.313748186	0.607177692	stable	0.988954333
Q9UL25	RAB21	-0.517819554	0.371471926	stable	0.988954333
Q9UL18	AGO1	0.409336203	0.493761199	stable	0.988954333
Q9UKY7	CDV3	0.004769539	0.993927258	stable	0.998177081
Q9UKV8	AGO2	-0.364244733	0.546698707	stable	0.988954333
Q9UKV3	ACIN1	-0.561240142	0.324931248	stable	0.988954333
Q9UKU9	ANGPTL2	0.199357341	0.747861859	stable	0.988954333
Q9UKM9	RALY	-0.221675272	0.720083212	stable	0.988954333
Q9UKG1	APPL1	0.409071349	0.494068895	stable	0.988954333
Q9UKE5	TNIK	0.114684971	0.854299289	stable	0.990207998
Q9UK55	SERPINA10	-0.895353677	0.039992871	negative	0.988954333
Q9UJX4	ANAPC5	0.717295412	0.172583851	stable	0.988954333
Q9UJU6	DBNL	0.126000196	0.83999708	stable	0.990207998
Q9UJJ9	GNPTG	-0.683354179	0.203426293	stable	0.988954333
Q9UIQ6	LNPEP	0.347529838	0.566587108	stable	0.988954333
Q9UII2	ATP5IF1	0.585223433	0.299911196	stable	0.988954333
Q9UIB8	CD84	0.639549807	0.245245113	stable	0.988954333
Q9UI15	TAGLN3	-0.452066451	0.444665857	stable	0.988954333
Q9UI12	ATP6V1H	-0.195069867	0.753213624	stable	0.988954333
Q9UI10	EIF2B4	-0.130322035	0.834539726	stable	0.990207998
Q9UHG3	PCYOX1	0.676529642	0.209797796	stable	0.988954333
Q9UHD8	SEPTIN9	0.041146905	0.947624921	stable	0.990577889
Q9UHB9	SRP68	-0.179086701	0.773204509	stable	0.988954333
Q9UHA4	LAMTOR3	-0.416159453	0.485848133	stable	0.988954333
Q9UH65	SWAP70	0.240395018	0.696893708	stable	0.988954333
Q9UGM5	FETUB	-0.600274565	0.284477346	stable	0.988954333

Q9UEY8	ADD3	0.183120358	0.768153626	stable	0.988954333
Q9UEU0	VTI1B	-0.088244876	0.887789129	stable	0.990207998
Q9UDY4	DNAJB4	0.261837231	0.670468019	stable	0.988954333
Q9UDY2	TJP2	-0.795748946	0.107350869	stable	0.988954333
Q9UDT6	CLIP2	0.055039759	0.929956601	stable	0.990207998
Q9UBX5	FBLN5	0.84073178	0.074450932	stable	0.988954333
Q9UBX1	CTSF	0.807231574	0.098608289	stable	0.988954333
Q9UBW5	BIN2	-0.401584524	0.502783225	stable	0.988954333
Q9UBV8	PEF1	-0.700397226	0.187759315	stable	0.988954333
Q9UBV2	SEL1L	-0.28451135	0.642697196	stable	0.988954333
Q9UBS4	DNAJB11	0.16966954	0.785011058	stable	0.988954333
Q9UBQ7	GRHPR	-0.269310406	0.661294587	stable	0.988954333
Q9UBQ5	EIF3K	-0.560764341	0.325432745	stable	0.988954333
Q9UBI6	GNG12	-0.062229802	0.920817725	stable	0.990207998
Q9P2X0	DPM3	-0.62850768	0.256117127	stable	0.988954333
Q9P2T1	GMPR2	0.323942646	0.594875237	stable	0.988954333
Q9P2J5	LARS1	-0.076548055	0.902631257	stable	0.990207998
Q9P2E9	RRBP1	-0.436121635	0.462855363	stable	0.988954333
Q9P289	STK26	-0.464248915	0.430878543	stable	0.988954333
Q9P270	SLAIN2	-0.204001188	0.742070632	stable	0.988954333
Q9P265	DIP2B	0.677880982	0.208531804	stable	0.988954333
Q9P258	RCC2	-0.77216223	0.125992046	stable	0.988954333
Q9P219	CCDC88C	-0.652863564	0.232308569	stable	0.988954333
Q9P107	GMIP	-0.119598579	0.848086168	stable	0.990207998
Q9NZP8	C1RL	-0.337567643	0.578503909	stable	0.988954333
Q9NZN5	ARHGEF12	-0.263261551	0.66871814	stable	0.988954333
Q9NZN4	EHD2	-0.200717895	0.746164561	stable	0.988954333
Q9NZN3	EHD3	0.080259326	0.897920468	stable	0.990207998
Q9NZM3	ITSN2	0.674062235	0.212114871	stable	0.988954333
Q9NZK5	ADA2	0.052195403	0.933572936	stable	0.990504544
Q9NZD4	AHSP	0.643056726	0.241819066	stable	0.988954333
Q9NZB2	FAM120A	0.726048037	0.164869556	stable	0.988954333
Q9NYU2	UGGT1	0.048447334	0.938339077	stable	0.990577889
Q9NYL9	TMOD3	-0.503932337	0.3866719	stable	0.988954333
Q9NYF8	BCLAF1	-0.228218447	0.711965715	stable	0.988954333
Q9NVU7	SDAD1	-0.655325532	0.229937462	stable	0.988954333
Q9NVN3	RIC8B	0.952438453	0.012362177	positive	0.988954333
Q9NVM6	DNAJC17	0.046285331	0.941088734	stable	0.990577889
Q9NVI7	ATAD3A	-0.4256392	0.474899417	stable	0.988954333
Q9NVA2	SEPTIN11	0.25976036	0.673020861	stable	0.988954333
Q9NV96	TMEM30A	-0.192934488	0.755880817	stable	0.988954333
Q9NV70	EXOC1	-0.178078798	0.774467181	stable	0.988954333
Q9NUV9	GIMAP4	0.439011599	0.459546704	stable	0.988954333
Q9NUU7	DDX19A	-0.829453871	0.082349756	stable	0.988954333
Q9NUQ9	CYRIB	0.179759348	0.772361965	stable	0.988954333
Q9NUB1	ACSS1	-0.111885293	0.857841001	stable	0.990207998
Q9NTK5	OLA1	0.67813739	0.208291832	stable	0.988954333
Q9NTJ5	SACM1L	-0.086810815	0.889608024	stable	0.990207998
Q9NT62	ATG3	0.417391699	0.484421947	stable	0.988954333
Q9NSY1	BMP2K	-0.337475192	0.578614714	stable	0.988954333
Q9NSB8	HOMER2	0.128034911	0.837427383	stable	0.990207998
Q9NS86	LANCL2	-0.203450733	0.742756794	stable	0.988954333
Q9NRY5	FAM114A2	0.386217979	0.520766469	stable	0.988954333
Q9NRW7	VPS45	-0.345276456	0.569278565	stable	0.988954333
Q9NRW1	RAB6B	0.439959857	0.458462194	stable	0.988954333
Q9NRV9	HEBP1	0.195502037	0.752673963	stable	0.988954333
Q9NRN5	OLFML3	-0.025254735	0.967848091	stable	0.992762798
Q9NRL3	STRN4	0.671612987	0.21442189	stable	0.988954333

Q9NRF8	CTPS2	0.382747082	0.52484605	stable	0.988954333
Q9NR99	MXRA5	0.455921754	0.440292164	stable	0.988954333
Q9NR50	EIF2B3	-0.621148107	0.263433217	stable	0.988954333
Q9NR31	SAR1A	0.235139348	0.703393522	stable	0.988954333
Q9NR30	DDX21	0.750170099	0.144150381	stable	0.988954333
Q9NR12	PDLIM7	0.549413693	0.337453731	stable	0.988954333
Q9NQG5	RPRD1B	0.071765905	0.908703308	stable	0.990207998
Q9NQC3	RTN4	-0.104654115	0.866993879	stable	0.990207998
Q9NQ79	CRTAC1	0.685674403	0.201272684	stable	0.988954333
Q9NQ29	LUC7L	-0.721342863	0.169003916	stable	0.988954333
Q9NPQ8	RIC8A	0.111376219	0.858485122	stable	0.990207998
Q9NPH3	IL1RAP	0.548662371	0.338253266	stable	0.988954333
Q9NP79	VTA1	-0.046030119	0.941413335	stable	0.990577889
Q9NP72	RAB18	0.359369153	0.552485922	stable	0.988954333
Q9NP58	ABCB6	0.460696247	0.434889135	stable	0.988954333
Q9NNX6	CD209	0.370805981	0.538929355	stable	0.988954333
Q9HDC9	APMAP	0.487576749	0.404756918	stable	0.988954333
Q9HD42	CHMP1A	-0.641830071	0.243015944	stable	0.988954333
Q9HCB6	SPON1	0.117636766	0.850566391	stable	0.990207998
Q9HC35	EML4	-0.241984751	0.694929352	stable	0.988954333
Q9HBL0	TNS1	0.412384025	0.490223257	stable	0.988954333
Q9HBI1	PARVB	-0.185591267	0.765061492	stable	0.988954333
Q9HB71	CACYBP	-0.391126065	0.515008642	stable	0.988954333
Q9HAU5	UPF2	0.944299784	0.015647933	positive	0.988954333
Q9H939	PSTPIP2	-0.766227891	0.130820288	stable	0.988954333
Q9H8W4	PLEKHF2	0.452314819	0.444383803	stable	0.988954333
Q9H8L6	MMRN2	0.226354132	0.714277308	stable	0.988954333
Q9H832	UBE2Z	0.76650113	0.130596796	stable	0.988954333
Q9H7D0	DOCK5	0.289258214	0.636907363	stable	0.988954333
Q9H7C9	AAMDC	0.524727669	0.363965808	stable	0.988954333
Q9H6X2	ANTXR1	0.313992065	0.606882867	stable	0.988954333
Q9H4M9	EHD1	0.00786641	0.989984279	stable	0.997512333
Q9H4G4	GLIPR2	-0.813434283	0.093980636	stable	0.988954333
Q9H4E7	DEF6	-0.893575017	0.041005486	negative	0.988954333
Q9H4B7	TUBB1	0.04653661	0.940769141	stable	0.990577889
Q9H4A4	RNPEP	0.172179	0.781862933	stable	0.988954333
Q9H4A3	WNK1	0.035134333	0.955274783	stable	0.991691336
Q9H479	FN3K	-0.155383777	0.802958254	stable	0.988954333
Q9H444	CHMP4B	-0.080440486	0.897690554	stable	0.990207998
Q9H3U1	UNC45A	-0.93981891	0.017561604	negative	0.988954333
Q9H3K6	BOLA2	-0.159484657	0.797801969	stable	0.988954333
Q9H2K8	TAOK3	0.065615145	0.916516189	stable	0.990207998
Q9H2G2	SLK	0.064606899	0.917797203	stable	0.990207998
Q9H299	SH3BGRL3	-0.520500802	0.368554171	stable	0.988954333
Q9H269	VPS16	0.064757209	0.917606223	stable	0.990207998
Q9H257	CARD9	-0.223916593	0.717301202	stable	0.988954333
Q9H251	CDH23	0.966328009	0.007379574	positive	0.988954333
Q9H223	EHD4	0.776560699	0.122448507	stable	0.988954333
Q9H1E3	NUCKS1	-0.056058313	0.928661741	stable	0.990207998
Q9H173	SIL1	-0.65556509	0.229707102	stable	0.988954333
Q9H0U4	RAB1B	0.412368769	0.490240953	stable	0.988954333
Q9H0B6	KLC2	0.744827643	0.148668822	stable	0.988954333
Q9H098	FAM107B	0.422353736	0.478687972	stable	0.988954333
Q9GZZ9	UBA5	0.033738738	0.957050656	stable	0.99196576
Q9GZP4	PITHD1	-0.191061381	0.75822136	stable	0.988954333
Q9GZM7	TINAGL1	0.837046792	0.077004712	stable	0.988954333
Q9C0C9	UBE2O	-0.104689927	0.866948533	stable	0.990207998
Q9BZZ5	API5	0.091573135	0.883568623	stable	0.990207998

Q9BZL4	PPP1R12C	-0.813206206	0.094149593	stable	0.988954333
Q9BZL1	UBL5	-0.389947412	0.516390174	stable	0.988954333
Q9BZF9	UACA	0.761545189	0.134667978	stable	0.988954333
Q9BZF1	OSBPL8	0.846724695	0.070355224	stable	0.988954333
Q9BYC5	FUT8	0.855365818	0.064578279	stable	0.988954333
Q9BY44	EIF2A	-0.247441057	0.688193402	stable	0.988954333
Q9BY43	CHMP4A	0.632248802	0.252419405	stable	0.988954333
Q9BXS5	AP1M1	0.229695961	0.710134457	stable	0.988954333
Q9BXR6	CFHR5	-0.13515931	0.828435225	stable	0.990207998
Q9BXP5	SRRT	0.308114923	0.61399465	stable	0.988954333
Q9BXJ9	NAA15	-0.040123324	0.948927108	stable	0.990577889
Q9BXD5	NPL	0.26435275	0.667377999	stable	0.988954333
Q9BX67	JAM3	0.179492922	0.772695672	stable	0.988954333
Q9BWS9	CHID1	0.56410706	0.321913645	stable	0.988954333
Q9BVC6	TMEM109	-0.039006194	0.950348369	stable	0.990577889
Q9BV40	VAMP8	-0.075706695	0.9036994	stable	0.990207998
Q9BUQ8	DDX23	0.811942701	0.095087261	stable	0.988954333
Q9BUL8	PDCD10	-0.046665481	0.940605236	stable	0.990577889
Q9BTU6	PI4K2A	0.369726663	0.540205913	stable	0.988954333
Q9BTT0	ANP32E	0.083426134	0.893901892	stable	0.990207998
Q9BSW2	CRACR2A	-0.049852389	0.936552269	stable	0.990577889
Q9BSJ8	ESYT1	0.872050057	0.053873872	stable	0.988954333
Q9BS26	ERP44	0.056962386	0.927512478	stable	0.990207998
Q9BRP8	PYM1	-0.647505433	0.237491884	stable	0.988954333
Q9BRK5	SDF4	0.295516673	0.629287085	stable	0.988954333
Q9BRG1	VPS25	-0.099406182	0.873640877	stable	0.990207998
Q9BR76	CORO1B	0.69058107	0.196739679	stable	0.988954333
Q9BQS8	FYCO1	0.275784832	0.653363187	stable	0.988954333
Q9BQL6	FERMT1	-0.641748422	0.243095668	stable	0.988954333
Q9BQE5	APOL2	-0.683929523	0.202891664	stable	0.988954333
Q9BQE3	TUBA1C	0.474078506	0.419826105	stable	0.988954333
Q9BPX7	C7orf25	0.371670338	0.537907469	stable	0.988954333
Q9BPX6	MICU1	-0.832594794	0.080125295	stable	0.988954333
Q99988	GDF15	0.508182562	0.382004443	stable	0.988954333
Q99985	SEMA3C	0.431891041	0.467708162	stable	0.988954333
Q99969	RARRES2	0.266513034	0.664726105	stable	0.988954333
Q99961	SH3GL1	0.397284303	0.507802684	stable	0.988954333
Q99933	BAG1	0.243008669	0.693664572	stable	0.988954333
Q99832	CCT7	-0.126488928	0.839379785	stable	0.990207998
Q99816	TSG101	-0.213871546	0.729780727	stable	0.988954333
Q99808	SLC29A1	0.286023131	0.64085232	stable	0.988954333
Q99755	PIP5K1A	0.731835714	0.159824883	stable	0.988954333
Q99747	NAPG	0.51019187	0.379802601	stable	0.988954333
Q99733	NAP1L4	0.744779365	0.148709839	stable	0.988954333
Q99729	HNRNPAB	-0.35212749	0.561103089	stable	0.988954333
Q99719	SEPTIN5	0.558093655	0.328251279	stable	0.988954333
Q99715	COL12A1	-0.141004455	0.821064257	stable	0.990207998
Q99685	MGLL	-0.897656549	0.038693857	negative	0.988954333
Q99683	MAP3K5	-0.181271656	0.770468068	stable	0.988954333
Q99615	DNAJC7	-0.691429075	0.195959209	stable	0.988954333
Q99613	EIF3C	-0.092713152	0.882123283	stable	0.990207998
Q99570	PIK3R4	0.651187747	0.233926345	stable	0.988954333
Q99543	DNAJC2	0.02869839	0.963465091	stable	0.99196576
Q99536	VAT1	0.175665708	0.777491181	stable	0.988954333
Q99497	PARK7	0.458268148	0.437635011	stable	0.988954333
Q99490	AGAP2	-0.292702147	0.632712155	stable	0.988954333
Q99460	PSMD1	-0.393827605	0.511844931	stable	0.988954333
Q99459	CDC5L	-0.667111407	0.218680084	stable	0.988954333

Q99439	CNN2	0.03700794	0.952890785	stable	0.990577889
Q99418	CYTH2	0.546310757	0.340758827	stable	0.988954333
Q96T51	RUFY1	-0.808968103	0.097305901	stable	0.988954333
Q96S97	MYADM	0.158977145	0.798439908	stable	0.988954333
Q96RU3	FNBP1	0.25828099	0.67484017	stable	0.988954333
Q96RT1	ERBIN	0.250158325	0.684842462	stable	0.988954333
Q96RL7	VPS13A	0.365138118	0.545639555	stable	0.988954333
Q96RF0	SNX18	-0.463530295	0.431689113	stable	0.988954333
Q96QK1	VPS35	0.591673962	0.293270674	stable	0.988954333
Q96PD5	PGLYRP2	-0.35514335	0.557511301	stable	0.988954333
Q96P48	ARAP1	0.318515806	0.601418692	stable	0.988954333
Q96MW1	CCDC43	-0.034708701	0.955816384	stable	0.991705679
Q96M27	PRRC1	0.119158499	0.848642489	stable	0.990207998
Q96KR1	ZFR	0.147976721	0.812280068	stable	0.988954333
Q96KP4	CNDP2	-0.372828919	0.536538333	stable	0.988954333
Q96KP1	EXOC2	0.213573541	0.730151392	stable	0.988954333
Q96KN2	CNDP1	-0.288018355	0.63841881	stable	0.988954333
Q96K76	USP47	-0.49785394	0.393370279	stable	0.988954333
Q96K17	BTF3L4	0.872683646	0.05347951	stable	0.988954333
Q96JM2	ZNF462	0.896783687	0.039184616	positive	0.988954333
Q96JJ3	ELMO2	-0.231681455	0.707674634	stable	0.988954333
Q96IY4	CPB2	-0.028957962	0.963134731	stable	0.99196576
Q96I99	SUCLG2	0.39228602	0.51364976	stable	0.988954333
Q96HY6	DDRGK1	0.013202209	0.983190914	stable	0.997512333
Q96HC4	PDLIM5	0.099683714	0.873289267	stable	0.990207998
Q96GG9	DCUN1D1	-0.597167607	0.287645853	stable	0.988954333
Q96FZ7	CHMP6	0.373887158	0.535288375	stable	0.988954333
Q96F85	CNRIP1	-0.497639355	0.393607246	stable	0.988954333
Q96F07	CYFIP2	0.51789135	0.371393724	stable	0.988954333
Q96EP5	DAZAP1	0.476958316	0.4166005	stable	0.988954333
Q96DV4	MRPL38	0.068689351	0.912610825	stable	0.990207998
Q96D71	REPS1	0.360949457	0.550608848	stable	0.988954333
Q96CW1	AP2M1	0.531635941	0.356496975	stable	0.988954333
Q96CT7	CCDC124	-0.335397131	0.581106345	stable	0.988954333
Q96C24	SYTL4	-0.52174675	0.367200217	stable	0.988954333
Q96C19	EFHD2	-0.95582725	0.011070489	negative	0.988954333
Q96BY6	DOCK10	0.210882221	0.733500026	stable	0.988954333
Q96BM9	ARL8A	-0.151800947	0.807465923	stable	0.988954333
Q96BJ3	AIDA	0.646448617	0.238517901	stable	0.988954333
Q96B97	SH3KBP1	0.751715624	0.142850897	stable	0.988954333
Q96AX1	VPS33A	-0.014039026	0.982125545	stable	0.997333758
Q96AT9	RPE	-0.566689548	0.319201541	stable	0.988954333
Q96AQ6	PBXIP1	-0.117602944	0.850609155	stable	0.990207998
Q96AP7	ESAM	-0.574862827	0.310656723	stable	0.988954333
Q96AG4	LRRC59	0.361046928	0.550493113	stable	0.988954333
Q96AC1	FERMT2	0.579593876	0.305737836	stable	0.988954333
Q96A65	EXOC4	-0.144017062	0.817267639	stable	0.990207998
Q96A00	PPP1R14A	-0.197614139	0.750037212	stable	0.988954333
Q969X1	TMBIM1	0.578875653	0.306483275	stable	0.988954333
Q969T9	WBP2	-0.605562058	0.279106357	stable	0.988954333
Q93091	RNASE6	-0.688679723	0.198492779	stable	0.988954333
Q93084	ATP2A3	0.320456949	0.599076683	stable	0.988954333
Q93050	ATP6V0A1	-0.428404297	0.471715919	stable	0.988954333
Q93034	CUL5	0.26825905	0.662583953	stable	0.988954333
Q93008	USP9X	-0.332363475	0.584747262	stable	0.988954333
Q92974	ARHGEF2	-0.351993763	0.561262455	stable	0.988954333
Q92954	PRG4	0.647501471	0.237495729	stable	0.988954333
Q92930	RAB8B	-0.462963299	0.432328901	stable	0.988954333

Q92896	GLG1	0.779141305	0.120383599	stable	0.988954333
Q92890	UFD1	0.707584714	0.181259969	stable	0.988954333
Q92888	ARHGEF1	0.367469054	0.542877994	stable	0.988954333
Q92882	OSTF1	-0.08573353	0.890974551	stable	0.990207998
Q92878	RAD50	-0.775173532	0.123562793	stable	0.988954333
Q92841	DDX17	-0.019572829	0.975080692	stable	0.994834663
Q92835	INPP5D	-0.305935187	0.616635931	stable	0.988954333
Q92820	GGH	0.212724513	0.731207565	stable	0.988954333
Q92804	TAF15	-0.581691334	0.303563572	stable	0.988954333
Q92743	HTRA1	0.856131026	0.064074187	stable	0.988954333
Q92734	TFG	0.116704661	0.851745008	stable	0.990207998
Q92688	ANP32B	0.673046511	0.213070768	stable	0.988954333
Q92626	PXDN	-0.368972343	0.541098432	stable	0.988954333
Q92621	NUP205	0.322919617	0.596107787	stable	0.988954333
Q92620	DHX38	0.980410668	0.00328158	positive	0.988954333
Q92619	ARHGAP45	-0.623452253	0.261136758	stable	0.988954333
Q92614	MYO18A	-0.504180863	0.386398606	stable	0.988954333
Q92608	DOCK2	-0.68241333	0.20430141	stable	0.988954333
Q92599	SEPTIN8	-0.027459613	0.965041729	stable	0.99196576
Q92598	HSPH1	-0.637057266	0.247688101	stable	0.988954333
Q92572	AP3S1	0.058508727	0.9255469	stable	0.990207998
Q92556	ELMO1	0.296577505	0.627996939	stable	0.988954333
Q92542	NCSTN	0.102107775	0.870218608	stable	0.990207998
Q92539	LPIN2	0.62162335	0.262959121	stable	0.988954333
Q92522	H1-10	0.300937045	0.622699712	stable	0.988954333
Q92520	FAM3C	-0.108190085	0.862517313	stable	0.990207998
Q92499	DDX1	0.688612885	0.198554485	stable	0.988954333
Q8WZA0	LZIC	0.954811208	0.011452863	positive	0.988954333
Q8WZ42	TTN	-0.030360086	0.961350277	stable	0.99196576
Q8WXX5	DNAJC9	-0.108842751	0.861691219	stable	0.990207998
Q8WXH0	SYNE2	0.406522163	0.497032477	stable	0.988954333
Q8WXG6	MADD	0.790926277	0.111089016	stable	0.988954333
Q8WWI5	SLC44A1	-0.018341662	0.97664798	stable	0.995725206
Q8WW22	DNAJA4	-0.798574049	0.105179108	stable	0.988954333
Q8WW12	PCNP	-0.203489107	0.742708956	stable	0.988954333
Q8WVM8	SCFD1	-0.26615409	0.66516662	stable	0.988954333
Q8WUW1	BRK1	0.056650806	0.927908553	stable	0.990207998
Q8WUM4	PDCD6IP	0.241613612	0.695387879	stable	0.988954333
Q8WUJ3	CEMIP	0.017202562	0.978098098	stable	0.996030767
Q8TF42	UBASH3B	-0.717723297	0.172204372	stable	0.988954333
Q8TEW0	PARD3	0.889354376	0.043440298	positive	0.988954333
Q8TEA8	DTD1	0.248021344	0.687477589	stable	0.988954333
Q8TDX7	NEK7	-0.232419055	0.706761129	stable	0.988954333
Q8TDR0	TRAF3IP1	-0.509715693	0.38032413	stable	0.988954333
Q8TDB6	DTX3L	-0.768447947	0.129007713	stable	0.988954333
Q8TD55	PLEKHO2	-0.435544003	0.4635173	stable	0.988954333
Q8TD06	AGR3	0.249488119	0.685668737	stable	0.988954333
Q8TBX8	PIP4K2C	-0.642028603	0.242822123	stable	0.988954333
Q8NHP6	MOSPD2	0.413847949	0.488525821	stable	0.988954333
Q8NHG7	SVIP	0.705035068	0.183558038	stable	0.988954333
Q8NFW8	CMAS	-0.405316795	0.498435073	stable	0.988954333
Q8NFH8	REPS2	-0.79356252	0.109040828	stable	0.988954333
Q8NF50	DOCK8	0.380504113	0.527485747	stable	0.988954333
Q8NE71	ABCF1	-0.611460439	0.273146813	stable	0.988954333
Q8NE00	TMEM104	-0.344436379	0.570282569	stable	0.988954333
Q8ND76	CCNY	0.517061501	0.372297852	stable	0.988954333
Q8NCA5	FAM98A	-0.811526834	0.095396502	stable	0.988954333
Q8NC51	SERBP1	0.234779642	0.703838693	stable	0.988954333

Q8NBZ7	UXS1	-0.177720386	0.774916246	stable	0.988954333
Q8NBS9	TXNDC5	0.634184537	0.250511826	stable	0.988954333
Q8NBJ7	SUMF2	0.863259818	0.05943791	stable	0.988954333
Q8NB16	MLKL	-0.315054992	0.605598191	stable	0.988954333
Q8N9N7	LRRC57	0.025418345	0.967639843	stable	0.992762798
Q8N6M0	OTUD6B	-0.443546506	0.454365276	stable	0.988954333
Q8N4C8	MINK1	0.397803145	0.507196518	stable	0.988954333
Q8N428	GALNT16	0.54395049	0.343278227	stable	0.988954333
Q8N392	ARHGAP18	0.065664131	0.916453953	stable	0.990207998
Q8N1N4	KRT78	0.840384198	0.074690675	stable	0.988954333
Q8N1G4	LRRC47	-0.335474031	0.581014105	stable	0.988954333
Q8N122	RPTOR	0.74196524	0.151106462	stable	0.988954333
Q8IZS8	CACNA2D3	-0.078808932	0.899761317	stable	0.990207998
Q8IZP0	ABI1	-0.402610623	0.501587025	stable	0.988954333
Q8IZH2	XRN1	0.374594198	0.534453562	stable	0.988954333
Q8IZA0	KIAA0319L	-0.693854008	0.193732237	stable	0.988954333
Q8IYJ1	CPNE9	0.084255414	0.892849737	stable	0.990207998
Q8IYD1	GSPT2	-0.683950688	0.202872004	stable	0.988954333
Q8IYB3	SRRM1	-0.227863249	0.71240605	stable	0.988954333
Q8IX12	CCAR1	0.730762206	0.160757141	stable	0.988954333
Q8IWW6	ARHGAP12	0.848211975	0.069349981	stable	0.988954333
Q8IWA5	SLC44A2	0.635428457	0.249288064	stable	0.988954333
Q8IW45	NAXD	0.704239983	0.184276357	stable	0.988954333
Q8IUX7	AEBP1	-0.496797684	0.394537036	stable	0.988954333
Q8IUR7	ARMC8	0.3982326	0.506694897	stable	0.988954333
Q8IUE6	H2AC21	-0.194014609	0.75453155	stable	0.988954333
Q86YT6	MIB1	-0.260571109	0.672024131	stable	0.988954333
Q86Y82	STX12	-0.816623673	0.091627702	stable	0.988954333
Q86VP6	CAND1	-0.768811339	0.128711736	stable	0.988954333
Q86VP3	PACS2	0.352092546	0.561144732	stable	0.988954333
Q86VN1	VPS36	-0.492428016	0.399372459	stable	0.988954333
Q86V81	ALYREF	0.18640452	0.764044095	stable	0.988954333
Q86UX7	FERMT3	-0.540134918	0.347360762	stable	0.988954333
Q86US8	SMG6	0.759486946	0.136369606	stable	0.988954333
Q86UP2	KTN1	0.78357346	0.11686179	stable	0.988954333
Q86UE4	MTDH	0.437480862	0.461298568	stable	0.988954333
Q7Z7G0	ABI3BP	0.191963403	0.757094129	stable	0.988954333
Q7Z7A4	PXK	-0.242948843	0.693738463	stable	0.988954333
Q7Z6Z7	HUWE1	0.473055083	0.420973788	stable	0.988954333
Q7Z4W1	DCXR	0.759708526	0.136186114	stable	0.988954333
Q7Z4V5	HDGFL2	0.923226337	0.025239844	positive	0.988954333
Q7Z478	DHX29	0.053647865	0.931726197	stable	0.990207998
Q7Z460	CLASP1	0.128657083	0.836641761	stable	0.990207998
Q7Z406	MYH14	0.987773868	0.00161983	positive	0.988954333
Q7Z3V4	UBE3B	0.31134431	0.610085067	stable	0.988954333
Q7Z2W4	ZC3HAV1	0.255212603	0.678615999	stable	0.988954333
Q7RTP6	MICAL3	0.006972537	0.991122363	stable	0.997512333
Q7LDG7	RASGRP2	-0.313505808	0.607470727	stable	0.988954333
Q7LBR1	CHMP1B	0.23009443	0.7096407	stable	0.988954333
Q7L9L4	MOB1B	-0.080436331	0.897695828	stable	0.990207998
Q7L8L6	FASTKD5	0.446329253	0.451192212	stable	0.988954333
Q7L7X3	TAOK1	-0.429973495	0.469911328	stable	0.988954333
Q7L591	DOK3	-0.74334711	0.149928205	stable	0.988954333
Q7L576	CYFIP1	0.007168178	0.99087327	stable	0.997512333
Q7L2H7	EIF3M	-0.152568031	0.806500618	stable	0.988954333
Q7L2E3	DHX30	0.317475462	0.602674541	stable	0.988954333
Q7L1Q6	BZW1	0.067237293	0.914455368	stable	0.990207998
Q7KZI7	MARK2	0.853473336	0.065830256	stable	0.988954333

Q7KZF4	SND1	0.430186438	0.469666556	stable	0.988954333
Q70J99	UNC13D	0.499679411	0.391355757	stable	0.988954333
Q709C8	VPS13C	0.079729222	0.898593254	stable	0.990207998
Q6ZVX7	NCCRP1	-0.748861261	0.145253559	stable	0.988954333
Q6ZN30	BNC2	-0.024774884	0.968458866	stable	0.992762798
Q6ZMP0	THSD4	-0.07309801	0.907011674	stable	0.990207998
Q6YHK3	CD109	0.866641965	0.05727679	stable	0.988954333
Q6VY07	PACS1	0.080107314	0.898113393	stable	0.990207998
Q6V0I7	FAT4	0.765648425	0.13129463	stable	0.988954333
Q6UXI9	NPNT	-0.198221878	0.749278721	stable	0.988954333
Q6UX71	PLXDC2	-0.724216672	0.166475232	stable	0.988954333
Q6UWY5	OLFML1	0.420346946	0.481005209	stable	0.988954333
Q6PL24	TMED8	-0.491732769	0.400143083	stable	0.988954333
Q6PKG0	LARP1	0.087147023	0.889181573	stable	0.990207998
Q6P2Q9	PRPF8	0.88547819	0.045715293	positive	0.988954333
Q6P2E9	EDC4	0.713754449	0.175733421	stable	0.988954333
Q6P1N0	CC2D1A	-0.290028663	0.635968446	stable	0.988954333
Q6P1M3	LLGL2	0.779808444	0.119851488	stable	0.988954333
Q6NZI2	CAVIN1	0.288631311	0.637671514	stable	0.988954333
Q6NYC8	PPP1R18	0.299018294	0.625030231	stable	0.988954333
Q6NYC1	JMJD6	0.695582195	0.192149533	stable	0.988954333
Q6KB66	KRT80	0.337220529	0.578919953	stable	0.988954333
Q6IBS0	TWF2	0.624569291	0.260025384	stable	0.988954333
Q6IAA8	LAMTOR1	0.193870239	0.754711877	stable	0.988954333
Q684P5	RAP1GAP2	0.827499262	0.083743476	stable	0.988954333
Q66K74	MAP1S	-0.553288901	0.333337376	stable	0.988954333
Q658P3	STEAP3	-0.18914946	0.7606113	stable	0.988954333
Q641Q3	METRNL	0.742298839	0.15082177	stable	0.988954333
Q5VZK9	CARMIL1	-0.245188842	0.690972659	stable	0.988954333
Q5VW36	FOCAD	0.577125006	0.308302215	stable	0.988954333
Q5VW32	BROX	0.416301672	0.485683485	stable	0.988954333
Q5VVQ6	YOD1	-0.937947466	0.018381839	negative	0.988954333
Q5TCZ1	SH3PXD2A	0.972642864	0.005409406	positive	0.988954333
Q5T8D3	ACBD5	-0.47723683	0.416288846	stable	0.988954333
Q5T4S7	UBR4	-0.680143939	0.206416577	stable	0.988954333
Q5T3F8	TMEM63B	0.055579761	0.929270101	stable	0.990207998
Q5T2T1	MPP7	0.416256775	0.485735461	stable	0.988954333
Q5T0N5	FNBP1L	-0.880288326	0.048818261	negative	0.988954333
Q5SSJ5	HP1BP3	-0.305208588	0.617516815	stable	0.988954333
Q5S007	LRRK2	0.572097402	0.313541233	stable	0.988954333
Q5K651	SAMD9	0.29988465	0.62397777	stable	0.988954333
Q5JWF2	GNAS	0.017753784	0.977396368	stable	0.995947817
Q5JSL3	DOCK11	0.037122059	0.952745585	stable	0.990577889
Q5JSH3	WDR44	-0.245725742	0.69030997	stable	0.988954333
Q5JRA6	MIA3	-0.574987507	0.310526835	stable	0.988954333
Q5H9R7	PPP6R3	-0.507198132	0.383084309	stable	0.988954333
Q567U6	CCDC93	0.57138141	0.314289165	stable	0.988954333
Q562R1	ACTBL2	-0.773135024	0.125205741	stable	0.988954333
Q53TN4	CYBRD1	-0.518776268	0.370430184	stable	0.988954333
Q53F19	NCBP3	-0.138260946	0.824523179	stable	0.990207998
Q52LJ0	FAM98B	0.086725157	0.889716675	stable	0.990207998
Q4V328	GRIPAP1	-0.729405892	0.161937239	stable	0.988954333
Q4LDE5	SVEP1	-0.732129013	0.15957045	stable	0.988954333
Q4KMQ2	ANO6	0.4170223	0.484849392	stable	0.988954333
Q4KMP7	TBC1D10B	0.418593661	0.483031669	stable	0.988954333
Q3YEC7	RABL6	-0.590621087	0.294351922	stable	0.988954333
Q3LXA3	TKFC	0.501591578	0.389248185	stable	0.988954333
Q32P44	EML3	0.400974435	0.503494729	stable	0.988954333

Q32MZ4	LRRFIP1	-0.468705458	0.42585949	stable	0.988954333
Q30154	HLA-DRB5	-0.368018184	0.542227814	stable	0.988954333
Q2LD37	BLTP1	0.142105091	0.819676995	stable	0.990207998
Q27J81	INF2	-0.561868563	0.324269188	stable	0.988954333
Q16851	UGP2	0.163195478	0.793139107	stable	0.988954333
Q16778	H2BC21	0.168403873	0.786599365	stable	0.988954333
Q16775	HAGH	-0.025363464	0.967709698	stable	0.992762798
Q16769	QPCT	-0.43298359	0.466453877	stable	0.988954333
Q16706	MAN2A1	0.056902523	0.927588574	stable	0.990207998
Q16643	DBN1	0.35011314	0.563504542	stable	0.988954333
Q16630	CPSF6	0.056310445	0.928341223	stable	0.990207998
Q16629	SRSF7	-0.789393397	0.112285228	stable	0.988954333
Q16610	ECM1	-0.581916239	0.303330669	stable	0.988954333
Q16563	SYPL1	-0.643831028	0.241064388	stable	0.988954333
Q16543	CDC37	0.210208382	0.734338752	stable	0.988954333
Q16537	PPP2R5E	0.543685264	0.343561622	stable	0.988954333
Q16531	DDB1	0.772183668	0.125974702	stable	0.988954333
Q16401	PSMD5	0.040906625	0.947930597	stable	0.990577889
Q16270	IGFBP7	-0.63285039	0.25182615	stable	0.988954333
Q16204	CCDC6	-0.425252609	0.475344871	stable	0.988954333
Q16181	SEPTIN7	-0.417256809	0.484578024	stable	0.988954333
Q15942	ZYX	0.096297321	0.877580203	stable	0.990207998
Q15904	ATP6AP1	-0.053333141	0.932126342	stable	0.990207998
Q15848	ADIPOQ	-0.78873471	0.112800429	stable	0.988954333
Q15836	VAMP3	0.127255189	0.838412036	stable	0.990207998
Q15833	STXBP2	0.296347459	0.628276675	stable	0.988954333
Q15758	SLC1A5	-0.062114814	0.920963848	stable	0.990207998
Q15746	MYLK	-0.685257828	0.20165887	stable	0.988954333
Q15691	MAPRE1	0.848842426	0.068925223	stable	0.988954333
Q15642	TRIP10	-0.695932058	0.191829569	stable	0.988954333
Q15582	TGFBI	0.368609876	0.541527408	stable	0.988954333
Q15555	MAPRE2	-0.096841491	0.876890583	stable	0.990207998
Q15485	FCN2	-0.054812582	0.930245415	stable	0.990207998
Q15477	SKIC2	0.306450624	0.616011178	stable	0.988954333
Q15435	PPP1R7	0.393514696	0.512211168	stable	0.988954333
Q15424	SAFB	-0.121338374	0.845887126	stable	0.990207998
Q15404	RSU1	-0.271234622	0.658935778	stable	0.988954333
Q15366	PCBP2	0.142153299	0.819616238	stable	0.990207998
Q15365	PCBP1	0.772075272	0.126062405	stable	0.988954333
Q15293	RCN1	-0.201575775	0.745094599	stable	0.988954333
Q15286	RAB35	-0.168850904	0.786038338	stable	0.988954333
Q15257	PTPA	-0.256375893	0.677184127	stable	0.988954333
Q15185	PTGES3	-0.114767845	0.854194468	stable	0.990207998
Q15172	PPP2R5A	0.051647656	0.93426941	stable	0.990577889
Q15149	PLEC	0.601422002	0.283309507	stable	0.988954333
Q15147	PLCB4	0.648666529	0.236366019	stable	0.988954333
Q15113	PCOLCE	0.503068797	0.387621857	stable	0.988954333
Q15102	PAFAH1B3	-0.491006494	0.40094847	stable	0.988954333
Q15084	PDIA6	-0.181751101	0.76986776	stable	0.988954333
Q15063	POSTN	0.027514466	0.964971914	stable	0.99196576
Q15056	EIF4H	0.732840206	0.158953987	stable	0.988954333
Q15046	KARS1	0.324525426	0.594173305	stable	0.988954333
Q15029	EFTUD2	-0.477823779	0.415632235	stable	0.988954333
Q15027	ACAP1	0.490299953	0.401732338	stable	0.988954333
Q15019	SEPTIN2	-0.784166735	0.116392756	stable	0.988954333
Q14CX7	NAA25	0.723208688	0.167360912	stable	0.988954333
Q14C86	GAPVD1	-0.291426806	0.634265169	stable	0.988954333
Q14997	PSME4	0.623361273	0.261227332	stable	0.988954333

Q14978	NOLC1	0.422126923	0.478949753	stable	0.988954333
Q14974	KPNB1	-0.293422567	0.631835161	stable	0.988954333
Q14956	GPNMB	-0.935792738	0.019341162	negative	0.988954333
Q14919	DRAP1	0.057866029	0.926363822	stable	0.990207998
Q14847	LASP1	-0.854837269	0.064927187	stable	0.988954333
Q14789	GOLGB1	-0.311671523	0.609689177	stable	0.988954333
Q14766	LTBP1	-0.68337391	0.203407951	stable	0.988954333
Q14739	LBR	-0.502179526	0.388600697	stable	0.988954333
Q14738	PPP2R5D	-0.750323179	0.144021517	stable	0.988954333
Q14697	GANAB	0.774460198	0.124136973	stable	0.988954333
Q14696	MESD	0.533609275	0.354370491	stable	0.988954333
Q14689	DIP2A	0.229965705	0.709800202	stable	0.988954333
Q14677	CLINT1	-0.303976538	0.619010971	stable	0.988954333
Q14644	RASA3	-0.260835089	0.671699644	stable	0.988954333
Q14624	ITIH4	0.298699234	0.625417905	stable	0.988954333
Q14558	PRPSAP1	-0.392947838	0.51287477	stable	0.988954333
Q14554	PDIA5	0.22937654	0.710530297	stable	0.988954333
Q14520	HABP2	0.440539261	0.457799814	stable	0.988954333
Q14517	FAT1	0.279824462	0.648422231	stable	0.988954333
Q14515	SPARCL1	-0.02035828	0.974080824	stable	0.994727102
Q14393	GAS6	-0.7804212	0.119363377	stable	0.988954333
Q14344	GNA13	0.268658105	0.662094512	stable	0.988954333
Q14315	FLNC	0.447428513	0.449940118	stable	0.988954333
Q14314	FGL2	0.437040377	0.461802954	stable	0.988954333
Q14258	TRIM25	0.448107299	0.449167343	stable	0.988954333
Q14254	FLOT2	-0.453628484	0.442892629	stable	0.988954333
Q14247	CTTN	0.782917818	0.11738079	stable	0.988954333
Q14242	SELPLG	0.346827112	0.567426194	stable	0.988954333
Q14232	EIF2B1	-0.302594263	0.620688042	stable	0.988954333
Q14206	RCAN2	0.207471223	0.737746964	stable	0.988954333
Q14204	DYNC1H1	0.794078031	0.108641656	stable	0.988954333
Q14203	DCTN1	0.565798521	0.320136631	stable	0.988954333
Q14185	DOCK1	0.71918984	0.170905558	stable	0.988954333
Q14165	MLEC	0.318931602	0.600916893	stable	0.988954333
Q14155	ARHGEF7	0.729951346	0.161462351	stable	0.988954333
Q14152	EIF3A	0.227035491	0.713432361	stable	0.988954333
Q14141	SEPTIN6	-0.107852875	0.862944149	stable	0.990207998
Q14126	DSG2	0.370019346	0.539859685	stable	0.988954333
Q14118	DAG1	0.238240942	0.699556664	stable	0.988954333
Q14112	NID2	0.512164806	0.377643584	stable	0.988954333
Q14103	HNRNPD	-0.552790374	0.333866216	stable	0.988954333
Q14019	COTL1	0.363165426	0.54797881	stable	0.988954333
Q14011	CIRBP	-0.810308406	0.096304291	stable	0.988954333
Q14008	CKAP5	-0.19895528	0.748363523	stable	0.988954333
Q13885	TUBB2A	-0.11278707	0.85670009	stable	0.990207998
Q13867	BLMH	-0.041002999	0.947807993	stable	0.990577889
Q13838	DDX39B	0.059847423	0.923845407	stable	0.990207998
Q13813	SPTAN1	0.177715839	0.774921943	stable	0.988954333
Q13790	APOF	-0.630466625	0.254179103	stable	0.988954333
Q13642	FHL1	0.052764422	0.932849438	stable	0.990296099
Q13619	CUL4A	0.647269488	0.237720849	stable	0.988954333
Q13618	CUL3	0.556898545	0.329514532	stable	0.988954333
Q13610	PWP1	-0.879152522	0.049505892	negative	0.988954333
Q13596	SNX1	-0.88845576	0.043964425	negative	0.988954333
Q13595	TRA2A	-0.090197833	0.885312464	stable	0.990207998
Q13576	IQGAP2	-0.64738571	0.237608058	stable	0.988954333
Q13557	CAMK2D	0.627250128	0.257363332	stable	0.988954333
Q13554	CAMK2B	-0.410659663	0.492224259	stable	0.988954333

Q13547	HDAC1	-0.804406466	0.100738313	stable	0.988954333
Q13526	PIN1	-0.177778022	0.77484403	stable	0.988954333
Q13510	ASAH1	-0.159794504	0.797412519	stable	0.988954333
Q13496	MTM1	-0.766363518	0.13070934	stable	0.988954333
Q13492	PICALM	0.858139976	0.062756652	stable	0.988954333
Q13464	ROCK1	-0.692507525	0.194967911	stable	0.988954333
Q13459	MYO9B	0.572982506	0.312617273	stable	0.988954333
Q13451	FKBP5	0.669084929	0.216810403	stable	0.988954333
Q13444	ADAM15	-0.150452022	0.809163701	stable	0.988954333
Q13442	PDAP1	0.611685964	0.272919625	stable	0.988954333
Q13438	OS9	-0.280394554	0.647725426	stable	0.988954333
Q13435	SF3B2	0.849775098	0.068298338	stable	0.988954333
Q13428	TCOF1	0.606257875	0.27840156	stable	0.988954333
Q13423	NNT	0.476285241	0.417353884	stable	0.988954333
Q13421	MSLN	0.256301408	0.677275796	stable	0.988954333
Q13418	ILK	-0.302510704	0.620789447	stable	0.988954333
Q13409	DYNC1I2	0.201952168	0.744625216	stable	0.988954333
Q13405	MRPL49	-0.558509385	0.327812132	stable	0.988954333
Q13404	UBE2V1	-0.233694213	0.705182255	stable	0.988954333
Q13347	EIF3I	-0.541578086	0.345815215	stable	0.988954333
Q13283	G3BP1	0.290689491	0.635163303	stable	0.988954333
Q13247	SRSF6	-0.558292282	0.328041446	stable	0.988954333
Q13243	SRSF5	-0.600952012	0.283787702	stable	0.988954333
Q13242	SRSF9	-0.001838088	0.997659675	stable	0.999264082
Q13228	SELENBP1	0.460301964	0.435334756	stable	0.988954333
Q13217	DNAJC3	0.656457414	0.228849598	stable	0.988954333
Q13214	SEMA3B	0.340215597	0.575331936	stable	0.988954333
Q13201	MMRN1	0.040885483	0.947957493	stable	0.990577889
Q13200	PSMD2	-0.043717303	0.944355137	stable	0.990577889
Q13188	STK3	-0.529474148	0.358830115	stable	0.988954333
Q13185	CBX3	-0.273270552	0.656441471	stable	0.988954333
Q13177	PAK2	-0.384239206	0.523091472	stable	0.988954333
Q13155	AIMP2	0.856082431	0.064106163	stable	0.988954333
Q13153	PAK1	-0.166078432	0.789518508	stable	0.988954333
Q13144	EIF2B5	-0.173794831	0.77983661	stable	0.988954333
Q13126	MTAP	0.441427801	0.456784433	stable	0.988954333
Q13098	GPS1	-0.728309143	0.162893322	stable	0.988954333
Q13094	LCP2	-0.149170058	0.810777529	stable	0.988954333
Q13045	FLII	0.19219315	0.756807053	stable	0.988954333
Q13043	STK4	-0.324462152	0.594249508	stable	0.988954333
Q12979	ABR	-0.134516484	0.829246222	stable	0.990207998
Q12974	PTP4A2	-0.030530928	0.961132855	stable	0.99196576
Q12965	MYO1E	0.426349312	0.474081416	stable	0.988954333
Q12913	PTPRJ	0.119085213	0.848735134	stable	0.990207998
Q12907	LMAN2	0.802208367	0.102405121	stable	0.988954333
Q12906	ILF3	0.00150574	0.998082833	stable	0.999264082
Q12905	ILF2	0.470867101	0.423429824	stable	0.988954333
Q12904	AIMP1	-0.968737561	0.006604213	negative	0.988954333
Q12846	STX4	-0.895225314	0.04006568	negative	0.988954333
Q12805	EFEMP1	0.668679108	0.217194508	stable	0.988954333
Q12797	ASPH	0.100600132	0.872128313	stable	0.990207998
Q12768	WASHC5	0.334676416	0.581970953	stable	0.988954333
Q10588	BST1	-0.358519066	0.553496157	stable	0.988954333
Q10567	AP1B1	0.183750599	0.767364795	stable	0.988954333
Q10471	GALNT2	0.967260803	0.007076061	positive	0.988954333
Q0ZGT2	NEXN	0.296644531	0.627915439	stable	0.988954333
Q0JRZ9	FCHO2	0.011466416	0.985400826	stable	0.997512333
Q09666	AHNAK	-0.274826402	0.654536341	stable	0.988954333

Q09028	RBBP4	0.006172302	0.992141231	stable	0.997698745
Q08722	CD47	-0.215870824	0.727294625	stable	0.988954333
Q08495	DMTN	0.696252471	0.191536671	stable	0.988954333
Q08431	MFGE8	0.643467001	0.241419108	stable	0.988954333
Q08380	LGALS3BP	0.621997257	0.262586275	stable	0.988954333
Q08378	GOLGA3	-0.307458814	0.61478948	stable	0.988954333
Q08257	CRYZ	-0.198554504	0.748863626	stable	0.988954333
Q08211	DHX9	-0.21295539	0.730920339	stable	0.988954333
Q08170	SRSF4	-0.03819658	0.951378432	stable	0.990577889
Q07960	ARHGAP1	0.323408033	0.595519284	stable	0.988954333
Q07955	SRSF1	-0.043730461	0.9443384	stable	0.990577889
Q07954	LRP1	-0.200709606	0.7461749	stable	0.988954333
Q07866	KLC1	-0.365086153	0.545701152	stable	0.988954333
Q07666	KHDRBS1	0.401848173	0.502475813	stable	0.988954333
Q07020	RPL18	-0.531709849	0.356417275	stable	0.988954333
Q06830	PRDX1	0.281953895	0.645820109	stable	0.988954333
Q06481	APLP2	-0.110051506	0.860161431	stable	0.990207998
Q06323	PSME1	0.41889593	0.482682175	stable	0.988954333
Q06187	BTK	-0.860149052	0.061447624	stable	0.988954333
Q06033	ITIH3	-0.254815187	0.679105275	stable	0.988954333
Q05707	COL14A1	0.355506311	0.557079323	stable	0.988954333
Q05682	CALD1	0.483358225	0.409452702	stable	0.988954333
Q05655	PRKCD	-0.830421805	0.081662247	stable	0.988954333
Q05397	PTK2	-0.097153235	0.87649553	stable	0.990207998
Q05209	PTPN12	-0.958857936	0.009955533	negative	0.988954333
Q05193	DNM1	-0.082961263	0.894491732	stable	0.990207998
Q04941	PLP2	0.516800349	0.372582489	stable	0.988954333
Q04917	YWHAH	0.153929074	0.804788154	stable	0.988954333
Q04771	ACVR1	0.429132183	0.470878658	stable	0.988954333
Q04756	HGFAC	-0.288114758	0.63830127	stable	0.988954333
Q04695	KRT17	0.861528361	0.060553939	stable	0.988954333
Q04637	EIF4G1	-0.320908569	0.598532032	stable	0.988954333
Q04323	UBXN1	-0.103660878	0.868251629	stable	0.990207998
Q03591	CFHR1	-0.475234161	0.418530999	stable	0.988954333
Q03252	LMNB2	0.903703386	0.035348883	positive	0.988954333
Q03169	TNFAIP2	-0.159079877	0.798310771	stable	0.988954333
Q03154	ACY1	-0.426359523	0.474069656	stable	0.988954333
Q02952	AKAP12	0.308225806	0.613860341	stable	0.988954333
Q02878	RPL6	-0.525793805	0.3628107	stable	0.988954333
Q02818	NUCB1	-0.715502736	0.174176333	stable	0.988954333
Q02809	PLOD1	-0.328610492	0.589257206	stable	0.988954333
Q02750	MAP2K1	-0.094154577	0.880296032	stable	0.990207998
Q02218	OGDH	-0.700713294	0.187472142	stable	0.988954333
Q02094	RHAG	-0.379366555	0.528825521	stable	0.988954333
Q01995	TAGLN	0.015898047	0.979758831	stable	0.996733537
Q01970	PLCB3	0.159107714	0.798275779	stable	0.988954333
Q01844	EWSR1	-0.668885015	0.216999595	stable	0.988954333
Q01813	PFKP	0.711413454	0.177824602	stable	0.988954333
Q01658	DR1	0.480619152	0.412508365	stable	0.988954333
Q01628	IFITM3	0.696831593	0.191007604	stable	0.988954333
Q01518	CAP1	-0.020760704	0.97356855	stable	0.994727102
Q01469	FABP5	-0.911345653	0.031262428	negative	0.988954333
Q01433	AMPD2	-0.746219355	0.147487832	stable	0.988954333
Q01130	SRSF2	-0.864897499	0.058388333	stable	0.988954333
Q01105	SET	0.328577661	0.589296687	stable	0.988954333
Q01082	SPTBN1	0.351421744	0.561944241	stable	0.988954333
Q00839	HNRNPU	-0.882518175	0.047477161	negative	0.988954333
Q00796	SORD	-0.680615257	0.205976786	stable	0.988954333

Q00722	PLCB2	-0.67521397	0.211032425	stable	0.988954333
Q00688	FKBP3	0.242290084	0.694552159	stable	0.988954333
Q00610	CLTC	0.512869798	0.376872815	stable	0.988954333
Q00577	PURA	0.858719289	0.062378312	stable	0.988954333
Q00341	HDLBP	0.189727196	0.759889023	stable	0.988954333
Q00013	MPP1	-0.615736197	0.268848088	stable	0.988954333
P98179	RBM3	-0.535109159	0.352756293	stable	0.988954333
P98172	EFNB1	0.049092978	0.937517997	stable	0.990577889
P98171	ARHGAP4	-0.308044462	0.614080001	stable	0.988954333
P98160	HSPG2	0.856108642	0.064088916	stable	0.988954333
P84243	H3-3A	-0.483290395	0.409528308	stable	0.988954333
P84103	SRSF3	-0.373111448	0.536204563	stable	0.988954333
P84098	RPL19	-0.106920037	0.864125007	stable	0.990207998
P84095	RHOG	0.384659793	0.522597121	stable	0.988954333
P84085	ARF5	0.601105485	0.283631526	stable	0.988954333
P82979	SARNP	0.682360644	0.204350446	stable	0.988954333
P80748	IGLV3-21	-0.572144933	0.313491597	stable	0.988954333
P80723	BASP1	-0.459549108	0.436185922	stable	0.988954333
P80511	S100A12	-0.076151897	0.903134188	stable	0.990207998
P80303	NUCB2	-0.580168973	0.305141282	stable	0.988954333
P80188	LCN2	0.524317674	0.364410255	stable	0.988954333
P80108	GPLD1	-0.509414755	0.380653819	stable	0.988954333
P78559	MAP1A	0.041035472	0.947766682	stable	0.990577889
P78527	PRKDC	-0.616584677	0.267997207	stable	0.988954333
P78509	RELN	-0.307002522	0.615342351	stable	0.988954333
P78417	GSTO1	-0.315520341	0.605035909	stable	0.988954333
P78371	CCT2	0.193369641	0.755337197	stable	0.988954333
P78356	PIP4K2B	0.389152201	0.517322687	stable	0.988954333
P78344	EIF4G2	-0.004107341	0.994770386	stable	0.998490147
P69905	HBA1	-0.594988335	0.28987376	stable	0.988954333
P69891	HBG1	-0.140176305	0.822108217	stable	0.990207998
P68871	HBB	-0.230240503	0.709459707	stable	0.988954333
P68402	PAFAH1B2	-0.977864734	0.003940159	negative	0.988954333
P68371	TUBB4B	-0.125509425	0.840616988	stable	0.990207998
P68366	TUBA4A	0.504183998	0.386395159	stable	0.988954333
P68104	EEF1A1	0.281136528	0.646818714	stable	0.988954333
P68036	UBE2L3	-0.421842504	0.479278065	stable	0.988954333
P67936	TPM4	0.410434842	0.492485274	stable	0.988954333
P67809	YBX1	0.495094199	0.396420446	stable	0.988954333
P67775	PPP2CA	0.793315797	0.109232028	stable	0.988954333
P63313	TMSB10	0.330835702	0.586582423	stable	0.988954333
P63241	EIF5A	-0.251669198	0.682980299	stable	0.988954333
P63218	GNG5	-0.423533344	0.477326995	stable	0.988954333
P63208	SKP1	0.876390132	0.051190894	stable	0.988954333
P63167	DYNLL1	0.681245049	0.205389534	stable	0.988954333
P63104	YWHAZ	0.059900337	0.923778155	stable	0.990207998
P63098	PPP3R1	0.327653761	0.590407908	stable	0.988954333
P63010	AP2B1	0.651364164	0.233755894	stable	0.988954333
P63000	RAC1	0.160218122	0.796880101	stable	0.988954333
P62995	TRA2B	-0.331464117	0.585827445	stable	0.988954333
P62993	GRB2	-0.524368871	0.364354749	stable	0.988954333
P62942	FKBP1A	-0.541886553	0.345485086	stable	0.988954333
P62937	PPIA	-0.274006638	0.655540028	stable	0.988954333
P62917	RPL8	0.045518007	0.942064693	stable	0.990577889
P62913	RPL11	-0.569740237	0.316005265	stable	0.988954333
P62906	RPL10A	0.786612535	0.114465133	stable	0.988954333
P62899	RPL31	-0.275012565	0.654308444	stable	0.988954333
P62879	GNB2	-0.250741902	0.68412311	stable	0.988954333

P62873	GNB1	0.510062199	0.379944605	stable	0.988954333
P62854	RPS26	0.781773991	0.118287878	stable	0.988954333
P62851	RPS25	0.569552199	0.31620204	stable	0.988954333
P62847	RPS24	-0.360474985	0.551172293	stable	0.988954333
P62841	RPS15	0.468060025	0.426585563	stable	0.988954333
P62834	RAP1A	-0.373048366	0.536279082	stable	0.988954333
P62826	RAN	0.248584118	0.686783483	stable	0.988954333
P62820	RAB1A	0.918125735	0.027774625	positive	0.988954333
P62805	H4C1	0.147698773	0.812630075	stable	0.988954333
P62753	RPS6	-0.708921319	0.180058556	stable	0.988954333
P62750	RPL23A	-0.183366108	0.767846025	stable	0.988954333
P62736	ACTA2	-0.392598042	0.513284353	stable	0.988954333
P62701	RPS4X	-0.639887615	0.244914526	stable	0.988954333
P62495	ETF1	0.605852958	0.278811646	stable	0.988954333
P62491	RAB11A	-0.433211959	0.466191795	stable	0.988954333
P62424	RPL7A	0.091664805	0.883452395	stable	0.990207998
P62333	PSMC6	-0.956553281	0.010799872	negative	0.988954333
P62330	ARF6	-0.096169616	0.877742049	stable	0.990207998
P62328	TMSB4X	-0.209070903	0.735754852	stable	0.988954333
P62318	SNRPD3	-0.845015098	0.071516264	stable	0.988954333
P62314	SNRPD1	-0.602317778	0.28239868	stable	0.988954333
P62312	LSM6	-0.703848706	0.184630149	stable	0.988954333
P62310	LSM3	-0.499722818	0.391307884	stable	0.988954333
P62304	SNRPE	-0.734868873	0.157199354	stable	0.988954333
P62277	RPS13	0.068715801	0.912577228	stable	0.990207998
P62269	RPS18	0.096388617	0.877464503	stable	0.990207998
P62263	RPS14	0.430839833	0.468915671	stable	0.988954333
P62258	YWHAE	-0.236913343	0.701198618	stable	0.988954333
P62256	UBE2H	-0.588794977	0.296229691	stable	0.988954333
P62249	RPS16	-0.247692076	0.687883745	stable	0.988954333
P62241	RPS8	-0.407965763	0.495353752	stable	0.988954333
P62191	PSMC1	0.875415056	0.051789905	stable	0.988954333
P62140	PPP1CB	0.745820959	0.147825624	stable	0.988954333
P62136	PPP1CA	-0.083862923	0.893347704	stable	0.990207998
P62081	RPS7	-0.219036755	0.723360098	stable	0.988954333
P61981	YWHAG	0.525056951	0.363608951	stable	0.988954333
P61978	HNRNPK	-0.507512505	0.382739379	stable	0.988954333
P61960	UFM1	0.151296523	0.808100757	stable	0.988954333
P61769	B2M	-0.884274536	0.046429189	negative	0.988954333
P61626	LYZ	-0.458270379	0.437632486	stable	0.988954333
P61604	HSPE1	0.374796485	0.534214765	stable	0.988954333
P61586	RHOA	0.237779681	0.700127082	stable	0.988954333
P61421	ATP6V0D1	-0.234275562	0.704462607	stable	0.988954333
P61353	RPL27	-0.597200176	0.287612591	stable	0.988954333
P61313	RPL15	-0.63172983	0.252931492	stable	0.988954333
P61254	RPL26	-0.7491376	0.145020436	stable	0.988954333
P61247	RPS3A	-0.106444398	0.864727154	stable	0.990207998
P61225	RAP2B	0.445591409	0.452033071	stable	0.988954333
P61224	RAP1B	-0.509245628	0.380839133	stable	0.988954333
P61221	ABCE1	0.248887415	0.686409451	stable	0.988954333
P61204	ARF3	-0.748255432	0.145765027	stable	0.988954333
P61201	COPS2	-0.782358397	0.117824165	stable	0.988954333
P61160	ACTR2	0.380653693	0.527309628	stable	0.988954333
P61158	ACTR3	0.815367268	0.092552423	stable	0.988954333
P61106	RAB14	0.184631311	0.766262623	stable	0.988954333
P61088	UBE2N	-0.592658298	0.292260748	stable	0.988954333
P61081	UBE2M	-0.049581428	0.93689684	stable	0.990577889
P61077	UBE2D3	0.476329412	0.417304433	stable	0.988954333

P61026	RAB10	0.833775647	0.079293864	stable	0.988954333
P61020	RAB5B	0.268179657	0.662681335	stable	0.988954333
P61019	RAB2A	0.659613644	0.22582362	stable	0.988954333
P61006	RAB8A	0.073530545	0.906462436	stable	0.990207998
P60983	GMFB	0.851855613	0.066906389	stable	0.988954333
P60981	DSTN	-0.215194698	0.728135262	stable	0.988954333
P60953	CDC42	-0.573673247	0.311896692	stable	0.988954333
P60900	PSMA6	0.764942315	0.131873328	stable	0.988954333
P60891	PRPS1	0.154478685	0.804096738	stable	0.988954333
P60866	RPS20	-0.583522406	0.301668723	stable	0.988954333
P60842	EIF4A1	0.271187116	0.658993997	stable	0.988954333
P60709	ACTB	-0.706934983	0.181844805	stable	0.988954333
P60660	MYL6	-0.422194046	0.478872279	stable	0.988954333
P60520	GABARAPL2	0.662726908	0.222849741	stable	0.988954333
P60228	EIF3E	0.171781009	0.782362121	stable	0.988954333
P60174	TPI1	-0.642682629	0.242183912	stable	0.988954333
P60033	CD81	-0.789810386	0.11195944	stable	0.988954333
P59998	ARPC4	0.326327401	0.592003846	stable	0.988954333
P59768	GNG2	0.221451408	0.72036116	stable	0.988954333
P58546	MTPN	0.488425755	0.403813386	stable	0.988954333
P58335	ANTXR2	-0.122941702	0.843860989	stable	0.990207998
P58107	EPPK1	0.036805898	0.953147857	stable	0.990577889
P57772	EEFSEC	-0.081056003	0.896909412	stable	0.990207998
P55884	EIF3B	0.425698402	0.474831209	stable	0.988954333
P55854	SUMO3	0.803301366	0.101575268	stable	0.988954333
P55786	NPEPPS	0.309031913	0.612884078	stable	0.988954333
P55290	CDH13	-0.395783204	0.509557257	stable	0.988954333
P55209	NAP1L1	0.314802538	0.605903269	stable	0.988954333
P55160	NCKAP1L	-0.582110522	0.303129513	stable	0.988954333
P55145	MANF	0.001413349	0.998200469	stable	0.999264082
P55084	HADHB	0.602325905	0.282390421	stable	0.988954333
P55072	VCP	-0.514583323	0.375000994	stable	0.988954333
P55060	CSE1L	-0.456292045	0.439872592	stable	0.988954333
P55058	PLTP	0.201961493	0.744613588	stable	0.988954333
P55056	APOC4	0.596662523	0.288161809	stable	0.988954333
P55039	DRG2	-0.766612084	0.130506076	stable	0.988954333
P55036	PSMD4	0.557697188	0.328670217	stable	0.988954333
P55010	EIF5	0.263279182	0.668696483	stable	0.988954333
P54920	NAPA	-0.894623326	0.040407694	negative	0.988954333
P54709	ATP1B3	-0.29154739	0.634118303	stable	0.988954333
P54652	HSPA2	-0.069473092	0.91161532	stable	0.990207998
P54619	PRKAG1	-0.636243573	0.248487035	stable	0.988954333
P54578	USP14	0.210566904	0.733892485	stable	0.988954333
P54577	YARS1	0.191155958	0.75810316	stable	0.988954333
P54136	RARS1	-0.388003876	0.518669882	stable	0.988954333
P53999	SUB1	-0.292272613	0.633235138	stable	0.988954333
P53990	IST1	0.30449556	0.618381457	stable	0.988954333
P53680	AP2S1	0.165478068	0.790272338	stable	0.988954333
P53634	CTSC	0.665664117	0.220054046	stable	0.988954333
P53621	COPA	-0.304121383	0.618835279	stable	0.988954333
P53618	COPB1	0.623660372	0.260929598	stable	0.988954333
P53041	PPP5C	0.166747252	0.788678813	stable	0.988954333
P53004	BLVRA	0.401873211	0.502446621	stable	0.988954333
P52943	CRIP2	-0.706027826	0.182662255	stable	0.988954333
P52907	CAPZA1	-0.874985869	0.05205426	stable	0.988954333
P52888	THOP1	0.717531964	0.17237403	stable	0.988954333
P52788	SMS	0.414056149	0.488284511	stable	0.988954333
P52566	ARHGDIB	-0.723558839	0.167053093	stable	0.988954333

P52565	ARHGDIA	-0.417604248	0.484176036	stable	0.988954333
P52272	HNRNPM	0.855451784	0.064521586	stable	0.988954333
P52209	PGD	-0.261212154	0.671236194	stable	0.988954333
P51991	HNRNPA3	0.060425741	0.923110402	stable	0.990207998
P51911	CNN1	0.028158988	0.964151601	stable	0.99196576
P51888	PRELP	0.619553993	0.265025152	stable	0.988954333
P51884	LUM	0.564525539	0.321473768	stable	0.988954333
P51858	HDGF	0.570397982	0.315317205	stable	0.988954333
P51812	RPS6KA3	0.48528178	0.407309966	stable	0.988954333
P51665	PSMD7	-0.645946718	0.239005596	stable	0.988954333
P51659	HSD17B4	0.739081815	0.153573677	stable	0.988954333
P51511	MMP15	0.290485558	0.635411755	stable	0.988954333
P51452	DUSP3	0.23296769	0.706081759	stable	0.988954333
P51153	RAB13	0.110450191	0.859656903	stable	0.990207998
P51149	RAB7A	0.495458558	0.396017425	stable	0.988954333
P51148	RAB5C	0.352468734	0.56069646	stable	0.988954333
P50995	ANXA11	-0.232908434	0.706155131	stable	0.988954333
P50991	CCT4	0.332042844	0.585132317	stable	0.988954333
P50990	CCT8	0.50521682	0.385259901	stable	0.988954333
P50914	RPL14	-0.73939274	0.153307072	stable	0.988954333
P50895	BCAM	0.357322206	0.554919091	stable	0.988954333
P50579	METAP2	-0.661768239	0.22376433	stable	0.988954333
P50570	DNM2	0.028500592	0.963716833	stable	0.99196576
P50552	VASP	-0.741179949	0.151777243	stable	0.988954333
P50502	ST13	-0.114224598	0.854881602	stable	0.990207998
P50454	SERPINH1	0.364260228	0.546680334	stable	0.988954333
P50416	CPT1A	-0.388510706	0.51807519	stable	0.988954333
P50395	GDI2	0.292178469	0.633349774	stable	0.988954333
P50281	MMP14	-0.703995535	0.184497364	stable	0.988954333
P50238	CRIP1	-0.741284868	0.151687572	stable	0.988954333
P50225	SULT1A1	-0.265758677	0.665651943	stable	0.988954333
P50151	GNG10	-0.553264994	0.333362732	stable	0.988954333
P50148	GNAQ	0.60013285	0.284621669	stable	0.988954333
P49959	MRE11	-0.943637993	0.015926026	negative	0.988954333
P49915	GMPS	-0.357608037	0.554579207	stable	0.988954333
P49913	CAMP	0.664023688	0.221614237	stable	0.988954333
P49908	SELENOP	0.511173306	0.378728231	stable	0.988954333
P49821	NDUFV1	0.107267415	0.863685255	stable	0.990207998
P49773	HINT1	-0.043773256	0.944283963	stable	0.990577889
P49770	EIF2B2	-0.379043123	0.529206571	stable	0.988954333
P49757	NUMB	0.508465073	0.381694678	stable	0.988954333
P49721	PSMB2	0.590971542	0.293991908	stable	0.988954333
P49619	DGKG	-0.8362914	0.077531495	stable	0.988954333
P49591	SARS1	0.344611124	0.570073698	stable	0.988954333
P49590	HARS2	0.614081921	0.270509104	stable	0.988954333
P49589	CARS1	-0.545874842	0.341223784	stable	0.988954333
P49588	AARS1	-0.005560659	0.992919985	stable	0.997698745
P49585	PCYT1A	0.49336761	0.398331549	stable	0.988954333
P49458	SRP9	-0.384788249	0.522446154	stable	0.988954333
P49411	TUFM	-0.212918633	0.730966066	stable	0.988954333
P49407	ARRB1	0.116993253	0.851380079	stable	0.990207998
P49368	CCT3	0.607541461	0.277102644	stable	0.988954333
P49327	FASN	-0.803091674	0.101734317	stable	0.988954333
P49257	LMAN1	0.408712292	0.494486097	stable	0.988954333
P49189	ALDH9A1	0.139034172	0.823548186	stable	0.990207998
P49137	MAPKAPK2	-0.401820354	0.502508248	stable	0.988954333
P49006	MARCKSL1	0.151887054	0.80735756	stable	0.988954333
P48960	ADGRE5	0.319955213	0.599681878	stable	0.988954333

P48740	MASP1	-0.290002913	0.635999824	stable	0.988954333
P48735	IDH2	0.611174089	0.273435345	stable	0.988954333
P48729	CSNK1A1	0.79161922	0.110549532	stable	0.988954333
P48643	CCT5	0.02511724	0.968023099	stable	0.992762798
P48509	CD151	0.849451368	0.068515728	stable	0.988954333
P48507	GCLM	0.24724374	0.688436828	stable	0.988954333
P48444	ARCN1	0.196008175	0.752041996	stable	0.988954333
P48426	PIP4K2A	0.313437822	0.607552927	stable	0.988954333
P48061	CXCL12	-0.560518093	0.325692368	stable	0.988954333
P48059	LIMS1	0.505515532	0.38493171	stable	0.988954333
P47914	RPL29	0.433085611	0.466336792	stable	0.988954333
P47897	QARS1	-0.766785308	0.130364477	stable	0.988954333
P47756	CAPZB	0.523412409	0.365392057	stable	0.988954333
P47755	CAPZA2	0.277378442	0.651413292	stable	0.988954333
P46976	GYG1	0.80598097	0.099549491	stable	0.988954333
P46940	IQGAP1	-0.978585544	0.003749682	negative	0.988954333
P46939	UTRN	0.640048953	0.24475668	stable	0.988954333
P46821	MAP1B	-0.11311988	0.856279056	stable	0.990207998
P46783	RPS10	-0.508041303	0.382159351	stable	0.988954333
P46782	RPS5	0.324854776	0.593776682	stable	0.988954333
P46781	RPS9	0.585054941	0.300085166	stable	0.988954333
P46777	RPL5	-0.423520591	0.477341705	stable	0.988954333
P46776	RPL27A	0.450405002	0.44655367	stable	0.988954333
P46734	MAP2K3	-0.727017154	0.164021692	stable	0.988954333
P46459	NSF	0.816197031	0.091941395	stable	0.988954333
P46379	BAG6	0.554019456	0.332562779	stable	0.988954333
P46060	RANGAP1	-0.359380716	0.552472183	stable	0.988954333
P43652	AFM	0.054672469	0.930423545	stable	0.990207998
P43490	NAMPT	-0.031635627	0.959726989	stable	0.99196576
P43487	RANBP1	0.593540705	0.291356174	stable	0.988954333
P43405	SYK	-0.51996908	0.369132353	stable	0.988954333
P43251	BTD	0.383874405	0.523520331	stable	0.988954333
P43250	GRK6	-0.270314532	0.66006351	stable	0.988954333
P43243	MATR3	-0.700083616	0.188044379	stable	0.988954333
P43034	PAFAH1B1	0.172440454	0.781535019	stable	0.988954333
P42892	ECE1	0.920894382	0.026389025	positive	0.988954333
P42768	WAS	-0.702400579	0.185941222	stable	0.988954333
P42766	RPL35	-0.167040484	0.788310696	stable	0.988954333
P42356	PI4KA	0.674279689	0.211910382	stable	0.988954333
P42331	ARHGAP25	-0.30386096	0.619151168	stable	0.988954333
P42330	AKR1C3	0.9250337	0.024360609	positive	0.988954333
P42166	TMPO	0.060527668	0.922980862	stable	0.990207998
P41252	IARS1	0.231123179	0.70836616	stable	0.988954333
P41250	GARS1	-0.260602433	0.671985626	stable	0.988954333
P41240	CSK	-0.108941097	0.861566746	stable	0.990207998
P41222	PTGDS	-0.566001591	0.319923458	stable	0.988954333
P41218	MNDA	0.184424885	0.76652094	stable	0.988954333
P41091	EIF2S3	0.344335166	0.570403555	stable	0.988954333
P40939	HADHA	0.20057131	0.746347403	stable	0.988954333
P40926	MDH2	0.862896044	0.059671845	stable	0.988954333
P40925	MDH1	0.109769045	0.860518892	stable	0.990207998
P40818	USP8	-0.407794011	0.495553415	stable	0.988954333
P40763	STAT3	0.264382082	0.667341982	stable	0.988954333
P40227	CCT6A	0.660421942	0.225050466	stable	0.988954333
P40121	CAPG	-0.066809239	0.914999158	stable	0.990207998
P39748	FEN1	-0.20877053	0.73612886	stable	0.988954333
P39687	ANP32A	0.502333685	0.388430969	stable	0.988954333
P39060	COL18A1	-0.03932711	0.949940078	stable	0.990577889

P39059	COL15A1	0.169992595	0.784605706	stable	0.988954333
P39023	RPL3	-0.669867576	0.216070171	stable	0.988954333
P39019	RPS19	0.404778769	0.499061398	stable	0.988954333
P38646	HSPA9	-0.03579987	0.954427929	stable	0.99135991
P38606	ATP6V1A	-0.123511916	0.843140503	stable	0.990207998
P38571	LIPA	0.58379891	0.301382854	stable	0.988954333
P38159	RBMX	0.671552703	0.214478761	stable	0.988954333
P38117	ETFB	-0.246459851	0.68940402	stable	0.988954333
P37840	SNCA	0.085036339	0.891859	stable	0.990207998
P37837	TALDO1	-0.716676247	0.173133397	stable	0.988954333
P37802	TAGLN2	0.044056141	0.943924131	stable	0.990577889
P37235	HPCAL1	0.711493968	0.177752562	stable	0.988954333
P37108	SRP14	-0.028074293	0.964259396	stable	0.99196576
P36969	GPX4	-0.374178352	0.534944527	stable	0.988954333
P36959	GMPR	0.298834147	0.625253974	stable	0.988954333
P36955	SERPINF1	0.348361397	0.565594493	stable	0.988954333
P36915	GNL1	-0.165206317	0.790613579	stable	0.988954333
P36871	PGM1	-0.191912386	0.757157877	stable	0.988954333
P36578	RPL4	-0.567947856	0.317882192	stable	0.988954333
P36543	ATP6V1E1	0.388072079	0.518589848	stable	0.988954333
P36222	CHI3L1	0.380473025	0.527522353	stable	0.988954333
P35998	PSMC2	-0.412880031	0.489647994	stable	0.988954333
P35908	KRT2	-0.309199712	0.612680893	stable	0.988954333
P35858	IGFALS	-0.488504639	0.403725745	stable	0.988954333
P35813	PPM1A	-0.283729341	0.643651848	stable	0.988954333
P35749	MYH11	-0.25983013	0.672935077	stable	0.988954333
P35659	DEK	-0.534482725	0.353430251	stable	0.988954333
P35658	NUP214	-0.109027971	0.861456794	stable	0.990207998
P35637	FUS	0.129158795	0.836008292	stable	0.990207998
P35625	TIMP3	-0.198220623	0.749280287	stable	0.988954333
P35613	BSG	-0.860668435	0.061110623	stable	0.988954333
P35612	ADD2	0.153931362	0.804785276	stable	0.988954333
P35611	ADD1	0.228498852	0.711618125	stable	0.988954333
P35580	MYH10	-0.430868294	0.46888297	stable	0.988954333
P35579	MYH9	-0.668310641	0.217543419	stable	0.988954333
P35555	FBN1	-0.075931015	0.90341461	stable	0.990207998
P35542	SAA4	0.282958699	0.644592849	stable	0.988954333
P35527	KRT9	-0.315333827	0.605261263	stable	0.988954333
P35443	THBS4	-0.015363146	0.980439804	stable	0.996886576
P35442	THBS2	-0.407671959	0.495695313	stable	0.988954333
P35268	RPL22	-0.143888389	0.817429764	stable	0.990207998
P35241	RDX	0.367343735	0.543026396	stable	0.988954333
P35221	CTNNA1	-0.517938391	0.371342489	stable	0.988954333
P35052	GPC1	0.062436834	0.920554636	stable	0.990207998
P35030	PRSS3	0.480714449	0.412401966	stable	0.988954333
P34947	GRK5	-0.653663485	0.231537438	stable	0.988954333
P34932	HSPA4	0.603568576	0.281128158	stable	0.988954333
P33908	MAN1A1	0.855502401	0.064488212	stable	0.988954333
P33241	LSP1	-0.136555424	0.826674119	stable	0.990207998
P33176	KIF5B	-0.549752419	0.337093424	stable	0.988954333
P33151	CDH5	-0.476998874	0.416555113	stable	0.988954333
P32969	RPL9	-0.185118761	0.765652681	stable	0.988954333
P32942	ICAM3	0.618934228	0.265644758	stable	0.988954333
P32121	ARRB2	0.081578128	0.896246825	stable	0.990207998
P32119	PRDX2	0.393209405	0.512568541	stable	0.988954333
P31949	S100A11	-0.54067522	0.346781931	stable	0.988954333
P31948	STIP1	-0.073902776	0.905989786	stable	0.990207998
P31946	YWHAB	-0.14918539	0.810758225	stable	0.988954333

P31943	HNRNPH1	0.19460366	0.753795841	stable	0.988954333
P31942	HNRNPH3	0.180588597	0.771323413	stable	0.988954333
P31689	DNAJA1	-0.301575302	0.621924811	stable	0.988954333
P31327	CPS1	-0.25635239	0.677213052	stable	0.988954333
P31323	PRKAR2B	0.773339532	0.125040625	stable	0.988954333
P31150	GDI1	0.209996864	0.734602054	stable	0.988954333
P31146	CORO1A	-0.434319205	0.464921538	stable	0.988954333
P30740	SERPINB1	-0.304006296	0.618974874	stable	0.988954333
P30626	SRI	0.20822263	0.736811136	stable	0.988954333
P30622	CLIP1	-0.17520675	0.778066481	stable	0.988954333
P30613	PKLR	-0.585205851	0.299929349	stable	0.988954333
P30566	ADSL	-0.19762949	0.750018052	stable	0.988954333
P30533	LRPAP1	-0.447656377	0.449680669	stable	0.988954333
P30419	NMT1	0.603403672	0.281295576	stable	0.988954333
P30273	FCER1G	0.214185243	0.72939057	stable	0.988954333
P30153	PPP2R1A	-0.401049211	0.503407512	stable	0.988954333
P30101	PDIA3	-0.029764354	0.962108443	stable	0.99196576
P30086	PEBP1	0.092166369	0.882816488	stable	0.990207998
P30085	CMPK1	-0.234912248	0.703674576	stable	0.988954333
P30084	ECHS1	-0.042376189	0.946061113	stable	0.990577889
P30050	RPL12	-0.544955194	0.342205221	stable	0.988954333
P30044	PRDX5	0.282343499	0.645344205	stable	0.988954333
P30043	BLVRB	0.789265598	0.112385132	stable	0.988954333
P30041	PRDX6	0.388539203	0.518041758	stable	0.988954333
P30040	ERP29	0.088133487	0.8879304	stable	0.990207998
P29972	AQP1	-0.611891737	0.272712379	stable	0.988954333
P29966	MARCKS	-0.493607524	0.398065866	stable	0.988954333
P29692	EEF1D	0.689664045	0.197584657	stable	0.988954333
P29622	SERPINA4	-0.292261386	0.633248809	stable	0.988954333
P29590	PML	-0.673593692	0.212555668	stable	0.988954333
P29401	TKT	0.15023073	0.809442257	stable	0.988954333
P29350	PTPN6	-0.690523264	0.196792914	stable	0.988954333
P29323	EPHB2	-0.884349952	0.046384356	negative	0.988954333
P29279	CCN2	-0.268206576	0.662648316	stable	0.988954333
P29218	IMPA1	0.107161389	0.863819473	stable	0.990207998
P29144	TPP2	0.082272603	0.895365564	stable	0.990207998
P28838	LAP3	0.931618828	0.021243847	positive	0.988954333
P28676	GCA	0.262530808	0.669615822	stable	0.988954333
P28289	TMOD1	0.811076098	0.095732017	stable	0.988954333
P28070	PSMB4	0.295341662	0.62949997	stable	0.988954333
P28066	PSMA5	0.078902988	0.899641935	stable	0.990207998
P27986	PIK3R1	-0.145166885	0.815819024	stable	0.990207998
P27824	CANX	0.062272837	0.920763037	stable	0.990207998
P27816	MAP4	0.155993083	0.802191921	stable	0.988954333
P27797	CALR	0.057671481	0.926611115	stable	0.990207998
P27708	CAD	-0.162291689	0.794274505	stable	0.988954333
P27701	CD82	0.351413797	0.561953714	stable	0.988954333
P27695	APEX1	0.600516064	0.284231449	stable	0.988954333
P27448	MARK3	-0.459944037	0.435739375	stable	0.988954333
P27348	YWHAQ	0.598856059	0.285922813	stable	0.988954333
P27338	MAOB	-0.199985731	0.747077879	stable	0.988954333
P27169	PON1	0.174322637	0.779174845	stable	0.988954333
P27105	STOM	-0.499602935	0.391440104	stable	0.988954333
P26927	MST1	-0.127851552	0.837658925	stable	0.990207998
P26641	EEF1G	0.256495417	0.677037034	stable	0.988954333
P26640	VARS1	0.3322921	0.584832975	stable	0.988954333
P26639	TARS1	0.137774217	0.825136972	stable	0.990207998
P26599	PTBP1	0.182904577	0.768423726	stable	0.988954333

P26583	HMGB2	0.183658313	0.767480296	stable	0.988954333
P26572	MGAT1	0.770218571	0.127567478	stable	0.988954333
P26447	S100A4	-0.501417121	0.389440359	stable	0.988954333
P26373	RPL13	0.12894583	0.83627718	stable	0.990207998
P26368	U2AF2	-0.426148394	0.474312829	stable	0.988954333
P26196	DDX6	0.404037455	0.499924642	stable	0.988954333
P26038	MSN	0.47047845	0.42386643	stable	0.988954333
P25940	COL5A3	-0.727315373	0.16376104	stable	0.988954333
P25815	S100P	0.175205886	0.778067565	stable	0.988954333
P25789	PSMA4	0.930875484	0.021588724	positive	0.988954333
P25788	PSMA3	0.942629657	0.016352794	positive	0.988954333
P25787	PSMA2	-0.670794405	0.215194482	stable	0.988954333
P25786	PSMA1	-0.238438489	0.699312387	stable	0.988954333
P25774	CTSS	0.24193476	0.694991112	stable	0.988954333
P25705	ATP5F1A	-0.398491586	0.50639244	stable	0.988954333
P25686	DNAJB2	0.580590683	0.304704029	stable	0.988954333
P25685	DNAJB1	-0.089871638	0.885726102	stable	0.990207998
P25325	MPST	0.029958172	0.961861777	stable	0.99196576
P25311	AZGP1	-0.235588251	0.702838017	stable	0.988954333
P25098	GRK2	-0.160449337	0.796589518	stable	0.988954333
P24844	MYL9	0.501129932	0.38975676	stable	0.988954333
P24821	TNC	-0.275555049	0.653644419	stable	0.988954333
P24666	ACP1	0.878845944	0.049692018	positive	0.988954333
P24592	IGFBP6	-0.011920553	0.98482264	stable	0.997512333
P24534	EEF1B2	0.292032463	0.633527566	stable	0.988954333
P24158	PRTN3	0.932020534	0.021058225	positive	0.988954333
P23919	DTYMK	-0.029306806	0.962690758	stable	0.99196576
P23634	ATP2B4	0.832141706	0.08044502	stable	0.988954333
P23588	EIF4B	0.561987993	0.324143404	stable	0.988954333
P23528	CFL1	-0.849340292	0.068590366	stable	0.988954333
P23526	AHCY	0.375573354	0.533297882	stable	0.988954333
P23458	JAK1	-0.294546842	0.630466947	stable	0.988954333
P23396	RPS3	-0.31394233	0.60694299	stable	0.988954333
P23381	WARS1	0.481207375	0.411851711	stable	0.988954333
P23284	PPIB	0.29043016	0.635479248	stable	0.988954333
P23276	KEL	0.871332725	0.054321457	stable	0.988954333
P23246	SFPQ	-0.416305251	0.485679341	stable	0.988954333
P23229	ITGA6	-0.629420413	0.25521365	stable	0.988954333
P23193	TCEA1	0.289687896	0.636383698	stable	0.988954333
P23142	FBLN1	0.230404825	0.709256112	stable	0.988954333
P23141	CES1	-0.224148681	0.717013209	stable	0.988954333
P22891	PROZ	-0.378927503	0.529342801	stable	0.988954333
P22792	CPN2	-0.554303295	0.33226195	stable	0.988954333
P22748	CA4	-0.013766937	0.982471945	stable	0.997333758
P22694	PRKACB	0.23172606	0.707619388	stable	0.988954333
P22681	CBL	-0.099545545	0.873464315	stable	0.990207998
P22626	HNRNPA2B1	0.287526127	0.639019023	stable	0.988954333
P22392	NME2	0.939248602	0.017810274	positive	0.988954333
P22352	GPX3	-0.567519116	0.318331575	stable	0.988954333
P22314	UBA1	0.042427468	0.945995882	stable	0.990577889
P22303	ACHE	-0.314255114	0.606564896	stable	0.988954333
P22105	TNXB	0.891720691	0.042069715	positive	0.988954333
P22087	FBL	0.113417422	0.855902652	stable	0.990207998
P22061	PCMT1	-0.545777268	0.341327882	stable	0.988954333
P22059	OSBP	0.754593853	0.140440118	stable	0.988954333
P21926	CD9	-0.383310824	0.524183011	stable	0.988954333
P21810	BGN	0.387027245	0.519816205	stable	0.988954333
P21589	NT5E	-0.427059928	0.473263137	stable	0.988954333

P21333	FLNA	0.083841799	0.893374505	stable	0.990207998
P21291	CSRP1	0.334066272	0.582703097	stable	0.988954333
P21283	ATP6V1C1	0.627461897	0.25715336	stable	0.988954333
P21281	ATP6V1B2	0.14717742	0.813286627	stable	0.98910393
P20962	PTMS	0.137234336	0.82581784	stable	0.990207998
P20851	C4BPB	-0.130013004	0.834929849	stable	0.990207998
P20810	CAST	0.314045481	0.606818296	stable	0.988954333
P20774	OGN	-0.103369503	0.868620627	stable	0.990207998
P20742	PZP	0.007388439	0.990592832	stable	0.997512333
P20702	ITGAX	0.449392903	0.447704532	stable	0.988954333
P20701	ITGAL	0.033048025	0.957929609	stable	0.99196576
P20700	LMNB1	0.008087801	0.989702404	stable	0.997512333
P20618	PSMB1	0.233271289	0.705705855	stable	0.988954333
P20339	RAB5A	-0.301147407	0.622444296	stable	0.988954333
P20338	RAB4A	-0.869727049	0.055327541	stable	0.988954333
P20160	AZU1	0.008688704	0.988937338	stable	0.997512333
P20073	ANXA7	-0.132112805	0.832279362	stable	0.990207998
P20042	EIF2S2	0.522485159	0.366398369	stable	0.988954333
P19971	TYMP	0.553792688	0.332803169	stable	0.988954333
P19878	NCF2	-0.103706914	0.86819333	stable	0.990207998
P19827	ITIH1	-0.283906691	0.643435325	stable	0.988954333
P19823	ITIH2	-0.147693033	0.812637302	stable	0.988954333
P19652	ORM2	0.256159386	0.677450587	stable	0.988954333
P19634	SLC9A1	-0.479404526	0.41386506	stable	0.988954333
P19525	EIF2AK2	-0.385381279	0.521749314	stable	0.988954333
P19440	GGT1	0.117357915	0.850918975	stable	0.990207998
P19397	CD53	-0.060983824	0.922401139	stable	0.990207998
P19338	NCL	-0.133641281	0.830350504	stable	0.990207998
P19320	VCAM1	0.148252668	0.811932597	stable	0.988954333
P19256	CD58	0.183913229	0.767161256	stable	0.988954333
P19086	GNAZ	0.466600698	0.428228253	stable	0.988954333
P19022	CDH2	-0.628727968	0.255898994	stable	0.988954333
P19021	PAM	-0.524825976	0.363859259	stable	0.988954333
P19013	KRT4	-0.308494944	0.613534362	stable	0.988954333
P18669	PGAM1	-0.298929767	0.625137791	stable	0.988954333
P18577	RHCE	-0.781598283	0.118427405	stable	0.988954333
P18433	PTPRA	0.205491587	0.740213198	stable	0.988954333
P18428	LBP	-0.640522015	0.244294014	stable	0.988954333
P18206	VCL	-0.17746584	0.775235192	stable	0.988954333
P18124	RPL7	-0.435741415	0.463291052	stable	0.988954333
P18085	ARF4	-0.065084649	0.917190193	stable	0.990207998
P17987	TCP1	0.303416094	0.619690846	stable	0.988954333
P17980	PSMC3	0.793270559	0.109267096	stable	0.988954333
P17948	FLT1	0.007239119	0.990782948	stable	0.997512333
P17936	IGFBP3	-0.786623526	0.114456492	stable	0.988954333
P17931	LGALS3	-0.005589819	0.992882858	stable	0.997698745
P17927	CR1	0.426800478	0.47356186	stable	0.988954333
P17858	PFKL	0.651020103	0.234088352	stable	0.988954333
P17844	DDX5	0.284055898	0.64325317	stable	0.988954333
P17813	ENG	0.069017514	0.912193987	stable	0.990207998
P17812	CTPS1	0.215410761	0.727866614	stable	0.988954333
P17655	CAPN2	0.79518241	0.107787998	stable	0.988954333
P17612	PRKACA	0.685713752	0.201236216	stable	0.988954333
P17301	ITGA2	-0.606495058	0.278161424	stable	0.988954333
P17252	PRKCA	0.600646864	0.28409829	stable	0.988954333
P17096	HMGA1	0.276362444	0.65265633	stable	0.988954333
P16989	YBX3	-0.542090452	0.345266912	stable	0.988954333
P16949	STMN1	-0.080199539	0.897996346	stable	0.990207998

P16885	PLCG2	-0.729625633	0.161745878	stable	0.988954333
P16671	CD36	-0.586476937	0.298617759	stable	0.988954333
P16615	ATP2A2	0.969242602	0.006445319	positive	0.988954333
P16452	EPB42	-0.092645223	0.882209401	stable	0.990207998
P16403	H1-2	0.103662281	0.868249853	stable	0.990207998
P16401	H1-5	0.45756497	0.438430941	stable	0.988954333
P16298	PPP3CB	-0.18438721	0.766568085	stable	0.988954333
P16284	PECAM1	-0.727834246	0.163307816	stable	0.988954333
P16157	ANK1	0.084061746	0.893095448	stable	0.990207998
P16152	CBR1	-0.612493187	0.272106862	stable	0.988954333
P16150	SPN	-0.236029562	0.702291969	stable	0.988954333
P16070	CD44	0.054912123	0.930118867	stable	0.990207998
P16035	TIMP2	0.562706684	0.323386734	stable	0.988954333
P15924	DSP	0.601885152	0.282838479	stable	0.988954333
P15880	RPS2	-0.361874218	0.549510994	stable	0.988954333
P15814	IGLL1	0.032483166	0.958648423	stable	0.99196576
P15531	NME1	0.476134853	0.417522258	stable	0.988954333
P15498	VAV1	0.282719089	0.644885472	stable	0.988954333
P15311	EZR	0.913855691	0.029955798	positive	0.988954333
P15291	B4GALT1	-0.418521445	0.483115174	stable	0.988954333
P15170	GSPT1	0.136150006	0.827185492	stable	0.990207998
P15169	CPN1	0.30253149	0.620764221	stable	0.988954333
P15153	RAC2	0.016857058	0.978537944	stable	0.996030767
P15144	ANPEP	0.482900511	0.409962953	stable	0.988954333
P15121	AKR1B1	0.289247236	0.636920744	stable	0.988954333
P14923	JUP	-0.009711077	0.987635668	stable	0.997512333
P14868	DARS1	0.235883919	0.702472171	stable	0.988954333
P14866	HNRNPL	-0.395585464	0.509788479	stable	0.988954333
P14780	MMP9	-0.564798761	0.321186657	stable	0.988954333
P14770	GP9	-0.326635405	0.591633172	stable	0.988954333
P14649	MYL6B	0.489675284	0.402425671	stable	0.988954333
P14625	HSP90B1	0.21493617	0.728456727	stable	0.988954333
P14618	PKM	-0.150181691	0.809503987	stable	0.988954333
P14598	NCF1	0.960138244	0.009496303	positive	0.988954333
P14543	NID1	-0.05324417	0.932239462	stable	0.990207998
P14324	FDPS	0.339384096	0.576327642	stable	0.988954333
P14317	HCLS1	-0.932125295	0.021009903	negative	0.988954333
P14314	PRKCSH	0.319572823	0.600143189	stable	0.988954333
P14209	CD99	0.976448587	0.004323337	positive	0.988954333
P13987	CD59	-0.008813986	0.988777829	stable	0.997512333
P13929	ENO3	-0.599708336	0.285054106	stable	0.988954333
P13807	GYS1	-0.201936645	0.744644574	stable	0.988954333
P13797	PLS3	0.81666026	0.091600816	stable	0.988954333
P13796	LCP1	-0.494903606	0.396631301	stable	0.988954333
P13747	HLA-E	0.01453461	0.981494612	stable	0.997333758
P13716	ALAD	0.024208098	0.969180303	stable	0.992960627
P13693	TPT1	-0.436813011	0.46206335	stable	0.988954333
P13671	C6	-0.421032667	0.48021314	stable	0.988954333
P13667	PDIA4	0.622020053	0.262563548	stable	0.988954333
P13647	KRT5	-0.47402433	0.419886841	stable	0.988954333
P13646	KRT13	-0.412738234	0.489812434	stable	0.988954333
P13645	KRT10	0.098892895	0.874291194	stable	0.990207998
P13639	EEF2	-0.69972129	0.188373877	stable	0.988954333
P13611	VCAN	0.326295755	0.592041933	stable	0.988954333
P13598	ICAM2	0.011948235	0.984787397	stable	0.997512333
P13498	CYBA	0.670019813	0.215926267	stable	0.988954333
P13489	RNH1	0.445533614	0.452098949	stable	0.988954333
P13473	LAMP2	-0.776157284	0.122772253	stable	0.988954333

P13224	GP1BB	-0.486177237	0.406313367	stable	0.988954333
P12956	XRCC6	0.305156508	0.617579962	stable	0.988954333
P12931	SRC	0.493354672	0.398345877	stable	0.988954333
P12882	MYH1	0.563429399	0.322626281	stable	0.988954333
P12830	CDH1	-0.790627146	0.111322145	stable	0.988954333
P12829	MYL4	0.20414341	0.74189336	stable	0.988954333
P12814	ACTN1	0.188683186	0.761194288	stable	0.988954333
P12724	RNASE3	-0.868642143	0.056010599	stable	0.988954333
P12429	ANXA3	-0.060606626	0.922880514	stable	0.990207998
P12318	FCGR2A	0.442591559	0.455455294	stable	0.988954333
P12259	F5	0.50652815	0.383819659	stable	0.988954333
P12111	COL6A3	0.330808379	0.586615252	stable	0.988954333
P12110	COL6A2	0.127519155	0.838078682	stable	0.990207998
P12109	COL6A1	-0.06241065	0.920587909	stable	0.990207998
P12081	HARS1	0.44907159	0.448070035	stable	0.988954333
P11940	PABPC1	0.505329551	0.385136038	stable	0.988954333
P11766	ADH5	-0.090008563	0.885552469	stable	0.990207998
P11597	CETP	-0.566989574	0.318886835	stable	0.988954333
P11586	MTHFD1	0.399437149	0.505288479	stable	0.988954333
P11413	G6PD	-0.617744052	0.266835712	stable	0.988954333
P11387	TOP1	-0.161066236	0.795814273	stable	0.988954333
P11362	FGFR1	0.470309334	0.424056446	stable	0.988954333
P11279	LAMP1	0.474751639	0.419071634	stable	0.988954333
P11277	SPTB	0.865581558	0.057951664	stable	0.988954333
P11234	RALB	0.88691468	0.044867903	positive	0.988954333
P11233	RALA	0.55736127	0.329025279	stable	0.988954333
P11226	MBL2	0.419476895	0.482010593	stable	0.988954333
P11217	PYGM	-0.370338467	0.539482233	stable	0.988954333
P11216	PYGB	-0.304342912	0.61856659	stable	0.988954333
P11215	ITGAM	0.830530721	0.081584997	stable	0.988954333
P11171	EPB41	0.539619366	0.3479133	stable	0.988954333
P11169	SLC2A3	-0.404623578	0.499242089	stable	0.988954333
P11166	SLC2A1	0.684856656	0.202030973	stable	0.988954333
P11142	HSPA8	-0.109846844	0.860420435	stable	0.990207998
P11021	HSPA5	0.055415905	0.929478408	stable	0.990207998
P10909	CLU	-0.340978618	0.574418515	stable	0.988954333
P10809	HSPD1	0.584325858	0.300838254	stable	0.988954333
P10768	ESD	0.932699006	0.020745913	positive	0.988954333
P10645	CHGA	0.581370838	0.303895546	stable	0.988954333
P10644	PRKAR1A	-0.713838604	0.175658378	stable	0.988954333
P10643	C7	-0.595629738	0.289217577	stable	0.988954333
P10599	TXN	-0.227134459	0.713309643	stable	0.988954333
P10451	SPP1	0.301629067	0.621859543	stable	0.988954333
P10412	H1-4	0.511763202	0.378082827	stable	0.988954333
P10321	HLA-C	-0.114765659	0.854197232	stable	0.990207998
P10301	RRAS	0.695970303	0.191794602	stable	0.988954333
P10155	RO60	0.667493649	0.218317607	stable	0.988954333
P10124	SRGN	0.602744616	0.281964942	stable	0.988954333
P10114	RAP2A	0.229940632	0.709831271	stable	0.988954333
P0DPH7	TUBA3C	0.254176478	0.679891727	stable	0.988954333
P0DP23	CALM1	-0.485672697	0.406874825	stable	0.988954333
P0DOY3	IGLC3	0.2346271	0.704027489	stable	0.988954333
P0DMV8	HSPA1A	-0.056602365	0.927970131	stable	0.990207998
P0DJI8	SAA1	-0.701632761	0.186637442	stable	0.988954333
P0C0L5	C4B	-0.819829802	0.089280889	stable	0.988954333
P0C0L4	C4A	-0.834972867	0.078453647	stable	0.988954333
P09972	ALDOC	0.551048108	0.335716068	stable	0.988954333
P09960	LTA4H	0.466387614	0.428468231	stable	0.988954333

P09871	C1S	-0.333891671	0.582912642	stable	0.988954333
P09668	CTSH	0.48033871	0.412821518	stable	0.988954333
P09651	HNRNPA1	0.31530853	0.605291829	stable	0.988954333
P09543	CNP	-0.318752822	0.601132642	stable	0.988954333
P09525	ANXA4	-0.148097513	0.812127966	stable	0.988954333
P09497	CLTB	0.726672956	0.164322681	stable	0.988954333
P09496	CLTA	0.737842847	0.154637379	stable	0.988954333
P09493	TPM1	-0.094285494	0.880130084	stable	0.990207998
P09486	SPARC	-0.337410418	0.578692349	stable	0.988954333
P09467	FBP1	0.67663714	0.20969701	stable	0.988954333
P09429	HMGB1	0.237832408	0.700061874	stable	0.988954333
P09382	LGALS1	-0.660698428	0.224786169	stable	0.988954333
P09326	CD48	-0.273332599	0.656365477	stable	0.988954333
P09211	GSTP1	-0.301535482	0.621973152	stable	0.988954333
P09110	ACAA1	0.412843963	0.48968982	stable	0.988954333
P09104	ENO2	0.174060598	0.779503383	stable	0.988954333
P09012	SNRPA	0.50105982	0.389834013	stable	0.988954333
P08962	CD63	-0.923106492	0.0252985	negative	0.988954333
P08779	KRT16	0.713996173	0.175517896	stable	0.988954333
P08758	ANXA5	-0.623070812	0.261516555	stable	0.988954333
P08754	GNAI3	0.076606196	0.902557447	stable	0.990207998
P08709	F7	0.247971191	0.687539451	stable	0.988954333
P08708	RPS17	-0.507572762	0.382673274	stable	0.988954333
P08697	SERPINF2	-0.696965497	0.190885332	stable	0.988954333
P08670	VIM	0.951013359	0.012919127	positive	0.988954333
P08648	ITGA5	-0.092407009	0.882511402	stable	0.990207998
P08637	FCGR3A	0.465564115	0.429395951	stable	0.988954333
P08631	HCK	-0.257335236	0.676003635	stable	0.988954333
P08603	CFH	-0.636571198	0.24816527	stable	0.988954333
P08575	PTPRC	0.112177288	0.857471562	stable	0.990207998
P08572	COL4A2	0.804741341	0.100485108	stable	0.988954333
P08571	CD14	-0.469659516	0.424786748	stable	0.988954333
P08567	PLEK	-0.947379001	0.014375221	negative	0.988954333
P08519	LPA	-0.416975301	0.484903782	stable	0.988954333
P08514	ITGA2B	-0.425775899	0.474741925	stable	0.988954333
P08493	MGP	-0.044830639	0.942938983	stable	0.990577889
P08397	HMBS	-0.155581068	0.802710111	stable	0.988954333
P08311	CTSG	-0.053969366	0.931317442	stable	0.990207998
P08294	SOD3	-0.394817506	0.510686677	stable	0.988954333
P08253	MMP2	0.815804087	0.092230601	stable	0.988954333
P08246	ELANE	-0.853530751	0.065792163	stable	0.988954333
P08240	SRPRA	0.132978981	0.831186243	stable	0.990207998
P08238	HSP90AB1	-0.357939369	0.554185266	stable	0.988954333
P08237	PFKM	-0.656720897	0.228596564	stable	0.988954333
P08195	SLC3A2	0.209496694	0.735224724	stable	0.988954333
P08185	SERPINA6	-0.396488535	0.50873267	stable	0.988954333
P08134	RHOC	0.416560218	0.485384196	stable	0.988954333
P08133	ANXA6	-0.639762184	0.245037261	stable	0.988954333
P08123	COL1A2	0.360411493	0.551247701	stable	0.988954333
P07996	THBS1	0.464325302	0.430792402	stable	0.988954333
P07951	TPM2	0.135815629	0.827607279	stable	0.990207998
P07948	LYN	0.591785976	0.293155702	stable	0.988954333
P07947	YES1	0.157834195	0.799876785	stable	0.988954333
P07910	HNRNPC	0.05706444	0.927382751	stable	0.990207998
P07900	HSP90AA1	-0.6977907	0.190132305	stable	0.988954333
P07858	CTSB	-0.404017771	0.499947568	stable	0.988954333
P07814	EPRS1	0.118233472	0.849811942	stable	0.990207998
P07741	APRT	-0.178193084	0.774323995	stable	0.988954333

P07737	PFN1	-0.723565537	0.167047206	stable	0.988954333
P07711	CTSL	0.734472585	0.157541666	stable	0.988954333
P07585	DCN	0.590538038	0.294437253	stable	0.988954333
P07451	CA3	0.085474727	0.89130286	stable	0.990207998
P07437	TUBB	-0.205677632	0.739981378	stable	0.988954333
P07384	CAPN1	-0.159869966	0.797317673	stable	0.988954333
P07360	C8G	-0.663539207	0.222075603	stable	0.988954333
P07359	GP1BA	-0.185191872	0.765561203	stable	0.988954333
P07358	C8B	-0.899493819	0.037667336	negative	0.988954333
P07357	C8A	-0.825367099	0.085271974	stable	0.988954333
P07355	ANXA2	0.599871198	0.284888185	stable	0.988954333
P07339	CTSD	0.447116957	0.450294912	stable	0.988954333
P07332	FES	-0.4921996	0.3996256	stable	0.988954333
P07305	H1-0	-0.244684636	0.69159508	stable	0.988954333
P07237	P4HB	0.306030288	0.616520653	stable	0.988954333
P07225	PROS1	-0.140421066	0.821799661	stable	0.990207998
P07205	PGK2	0.357626857	0.554556829	stable	0.988954333
P07203	GPX1	0.481539479	0.411481078	stable	0.988954333
P07195	LDHB	-0.088634077	0.887295524	stable	0.990207998
P07148	FABP1	-0.463816772	0.431365939	stable	0.988954333
P07108	DBI	0.614392316	0.270197237	stable	0.988954333
P07093	SERPINE2	-0.300412211	0.62333703	stable	0.988954333
P06753	TPM3	-0.363027513	0.548142421	stable	0.988954333
P06748	NPM1	0.442804867	0.455211766	stable	0.988954333
P06744	GPI	0.608611055	0.276021502	stable	0.988954333
P06737	PYGL	0.038352494	0.951180061	stable	0.990577889
P06733	ENO1	-0.131793983	0.832681749	stable	0.990207998
P06732	CKM	0.06876306	0.912517198	stable	0.990207998
P06730	EIF4E	0.054136657	0.931104752	stable	0.990207998
P06727	APOA4	0.691933478	0.195495391	stable	0.988954333
P06703	S100A6	0.204393365	0.741581818	stable	0.988954333
P06702	S100A9	-0.445009068	0.45269696	stable	0.988954333
P06681	C2	0.019443717	0.97524505	stable	0.994834663
P06576	ATP5F1B	-0.403790671	0.500212085	stable	0.988954333
P06454	PTMA	-0.650326705	0.23475876	stable	0.988954333
P06396	GSN	-0.75808941	0.137528593	stable	0.988954333
P06312	IGKV4-1	-0.514020844	0.375615188	stable	0.988954333
P06307	CCK	0.787193862	0.114008392	stable	0.988954333
P06276	BCHE	-0.590881815	0.294084071	stable	0.988954333
P05787	KRT8	0.14107924	0.82096999	stable	0.990207998
P05783	KRT18	-0.197850144	0.749742654	stable	0.988954333
P05771	PRKCB	0.379984671	0.528097444	stable	0.988954333
P05556	ITGB1	0.22583193	0.71492498	stable	0.988954333
P05546	SERPIND1	-0.359643313	0.552160189	stable	0.988954333
P05543	SERPINA7	-0.689488374	0.197746643	stable	0.988954333
P05455	SSB	0.022733309	0.971057545	stable	0.994341759
P05452	CLEC3B	-0.344691873	0.569977185	stable	0.988954333
P05388	RPLP0	-0.240548341	0.696704219	stable	0.988954333
P05387	RPLP2	0.552693126	0.333969402	stable	0.988954333
P05386	RPLP1	0.338963065	0.576831938	stable	0.988954333
P05362	ICAM1	0.663954054	0.221680532	stable	0.988954333
P05204	HMGN2	-0.327172212	0.590987241	stable	0.988954333
P05198	EIF2S1	0.2502489	0.684730807	stable	0.988954333
P05165	PCCA	-0.188081788	0.761946303	stable	0.988954333
P05164	MPO	0.391665819	0.514376232	stable	0.988954333
P05160	F13B	-0.6245239	0.26007052	stable	0.988954333
P05156	CFI	0.578893051	0.306465212	stable	0.988954333
P05155	SERPING1	-0.63298616	0.251692312	stable	0.988954333

P05154	SERPINA5	-0.043060688	0.945190377	stable	0.990577889
P05114	HMGN1	-0.533096574	0.354922682	stable	0.988954333
P05109	S100A8	-0.393615345	0.51209336	stable	0.988954333
P05107	ITGB2	0.671579583	0.214453403	stable	0.988954333
P05106	ITGB3	-0.52277199	0.366087009	stable	0.988954333
P05091	ALDH2	0.049585154	0.936892101	stable	0.990577889
P05090	APOD	-0.34995459	0.563693643	stable	0.988954333
P05089	ARG1	-0.388067102	0.518595687	stable	0.988954333
P05067	APP	-0.2244074	0.71669219	stable	0.988954333
P05060	CHGB	-0.154856709	0.803621215	stable	0.988954333
P05023	ATP1A1	0.122013714	0.845033645	stable	0.990207998
P04899	GNAI2	0.418340625	0.483324278	stable	0.988954333
P04839	CYBB	-0.160179572	0.79692855	stable	0.988954333
P04792	HSPB1	0.870278056	0.054981637	stable	0.988954333
P04632	CAPNS1	0.487190003	0.405186894	stable	0.988954333
P04439	HLA-A	0.352710394	0.56040853	stable	0.988954333
P04430	IGKV1-16	-0.519124009	0.370051712	stable	0.988954333
P04424	ASL	-0.399988489	0.504645008	stable	0.988954333
P04406	GAPDH	-0.420432976	0.480905821	stable	0.988954333
P04278	SHBG	-0.793488763	0.109097976	stable	0.988954333
P04275	VWF	0.569661228	0.316087941	stable	0.988954333
P04264	KRT1	-0.072402092	0.907895397	stable	0.990207998
P04233	CD74	-0.348988814	0.564845773	stable	0.988954333
P04217	A1BG	-0.307279563	0.615006662	stable	0.988954333
P04196	HRG	0.551118603	0.335641171	stable	0.988954333
P04180	LCAT	-0.447129867	0.450280209	stable	0.988954333
P04156	PRNP	0.592941483	0.29197037	stable	0.988954333
P04114	APOB	-0.764474635	0.132257034	stable	0.988954333
P04083	ANXA1	-0.171966411	0.782129573	stable	0.988954333
P04080	CSTB	-0.187939433	0.762124322	stable	0.988954333
P04075	ALDOA	0.441447266	0.456762195	stable	0.988954333
P04070	PROC	-0.611829807	0.272774748	stable	0.988954333
P04040	CAT	-0.154735223	0.803774031	stable	0.988954333
P04004	VTN	0.484460719	0.408224264	stable	0.988954333
P04003	C4BPA	0.154465087	0.804113843	stable	0.988954333
P03952	KLKB1	-0.36597598	0.54464659	stable	0.988954333
P03951	F11	0.459881539	0.435810035	stable	0.988954333
P03950	ANG	0.58549198	0.299633973	stable	0.988954333
P02792	FTL	-0.145655725	0.815203229	stable	0.990207998
P02790	HPX	-0.217834126	0.724854356	stable	0.988954333
P02788	LTF	0.707092485	0.181702985	stable	0.988954333
P02787	TF	0.133279507	0.830807007	stable	0.990207998
P02786	TFRC	0.374885346	0.534109874	stable	0.988954333
P02776	PF4	0.63434456	0.250354306	stable	0.988954333
P02775	PPBP	0.402534979	0.501675189	stable	0.988954333
P02774	GC	-0.121475172	0.845714237	stable	0.990207998
P02768	ALB	-0.532099322	0.35599735	stable	0.988954333
P02766	TTR	0.219098224	0.723283734	stable	0.988954333
P02765	AHSG	-0.444808083	0.452926139	stable	0.988954333
P02763	ORM1	-0.064123457	0.918411465	stable	0.990207998
P02760	AMBP	-0.413098229	0.489394975	stable	0.988954333
P02753	RBP4	-0.38303846	0.524503328	stable	0.988954333
P02751	FN1	-0.695595151	0.192137681	stable	0.988954333
P02750	LRG1	-0.84953915	0.068456759	stable	0.988954333
P02749	APOH	-0.365304279	0.545442608	stable	0.988954333
P02748	C9	-0.691550086	0.195847905	stable	0.988954333
P02747	C1QC	0.370521088	0.539266254	stable	0.988954333
P02746	C1QB	-0.451429871	0.445388957	stable	0.988954333

P02745	C1QA	-0.543629019	0.343621728	stable	0.988954333
P02743	APCS	-0.096843266	0.876888335	stable	0.990207998
P02741	CRP	0.809008408	0.097275735	stable	0.988954333
P02730	SLC4A1	0.424141798	0.476625313	stable	0.988954333
P02686	MBP	0.322760599	0.596299414	stable	0.988954333
P02679	FGG	0.519053609	0.370128326	stable	0.988954333
P02675	FGB	0.271957835	0.658049568	stable	0.988954333
P02671	FGA	0.387910063	0.518779973	stable	0.988954333
P02656	APOC3	0.309968953	0.611749582	stable	0.988954333
P02655	APOC2	-0.423725011	0.477105936	stable	0.988954333
P02654	APOC1	0.456240861	0.439930583	stable	0.988954333
P02652	APOA2	0.192437673	0.756501527	stable	0.988954333
P02649	APOE	0.249049037	0.686210146	stable	0.988954333
P02647	APOA1	0.497553984	0.393701531	stable	0.988954333
P02549	SPTA1	-0.279521727	0.648792304	stable	0.988954333
P02545	LMNA	0.138562563	0.824142845	stable	0.990207998
P02538	KRT6A	0.442378151	0.455698966	stable	0.988954333
P02533	KRT14	0.055020185	0.929981485	stable	0.990207998
P02452	COL1A1	0.563650616	0.322393602	stable	0.988954333
P02042	HBD	-0.421147191	0.480080882	stable	0.988954333
P01911	HLA-DRB1	0.380269085	0.527762499	stable	0.988954333
P01903	HLA-DRA	-0.008262928	0.989479433	stable	0.997512333
P01889	HLA-B	-0.513268878	0.376436668	stable	0.988954333
P01880	IGHD	0.683764592	0.203044884	stable	0.988954333
P01877	IGHA2	0.614813422	0.269774287	stable	0.988954333
P01876	IGHA1	0.38863839	0.517925393	stable	0.988954333
P01871	IGHM	0.43683306	0.462040387	stable	0.988954333
P01861	IGHG4	-0.542174129	0.345177386	stable	0.988954333
P01860	IGHG3	0.154081453	0.804596454	stable	0.988954333
P01859	IGHG2	-0.160332504	0.796736348	stable	0.988954333
P01857	IGHG1	0.218445975	0.724094085	stable	0.988954333
P01834	IGKC	0.554005502	0.332577569	stable	0.988954333
P01833	PIGR	-0.647387031	0.237606775	stable	0.988954333
P01780	IGHV3-7	0.500996447	0.389903844	stable	0.988954333
P01742	IGHV1-69	-0.314047158	0.606816269	stable	0.988954333
P01721	IGLV6-57	0.372196953	0.537285064	stable	0.988954333
P01717	IGLV3-25	0.496491968	0.394874887	stable	0.988954333
P01715	IGLV3-1	0.781246858	0.118706614	stable	0.988954333
P01714	IGLV3-19	-0.517484843	0.37183655	stable	0.988954333
P01709	IGLV2-8	-0.89205664	0.041876266	negative	0.988954333
P01706	IGLV2-11	0.463554938	0.431661311	stable	0.988954333
P01701	IGLV1-51	-0.98663037	0.001851996	negative	0.988954333
P01700	IGLV1-47	-0.710389971	0.178741088	stable	0.988954333
P01619	IGKV3-20	-0.613761207	0.270831438	stable	0.988954333
P01602	IGKV1-5	0.255471992	0.678296683	stable	0.988954333
P01601	IGKV1D-16	0.718582352	0.171443224	stable	0.988954333
P01597	IGKV1-39	-0.623599645	0.26099004	stable	0.988954333
P01593	IGKV1D-33	-0.506109363	0.384279476	stable	0.988954333
P01591	JCHAIN	-0.347909466	0.566133913	stable	0.988954333
P01303	NPY	-0.259142707	0.673780351	stable	0.988954333
P01137	TGFB1	-0.428445977	0.471667968	stable	0.988954333
P01116	KRAS	-0.502528606	0.388216388	stable	0.988954333
P01111	NRAS	0.411283525	0.491500113	stable	0.988954333
P01042	KNG1	0.322149467	0.597035967	stable	0.988954333
P01040	CSTA	0.267033986	0.664086847	stable	0.988954333
P01034	CST3	0.403551874	0.500490257	stable	0.988954333
P01033	TIMP1	-0.669005805	0.216885278	stable	0.988954333
P01031	C5	-0.623869703	0.260721276	stable	0.988954333

P01024	C3	-0.341610384	0.573662423	stable	0.988954333
P01023	A2M	-0.279180764	0.649209149	stable	0.988954333
P01019	AGT	-0.536023413	0.351773249	stable	0.988954333
P01011	SERPINA3	-0.492732414	0.399035168	stable	0.988954333
P01009	SERPINA1	0.064400951	0.918058878	stable	0.990207998
P01008	SERPINC1	-0.865267479	0.058152028	stable	0.988954333
P00966	ASS1	-0.631721185	0.252940024	stable	0.988954333
P00918	CA2	-0.902964264	0.035752547	negative	0.988954333
P00915	CA1	-0.359381806	0.552470888	stable	0.988954333
P00751	CFB	-0.612446155	0.2721542	stable	0.988954333
P00750	PLAT	0.119991186	0.847589885	stable	0.990207998
P00748	F12	0.532874924	0.355161471	stable	0.988954333
P00747	PLG	0.101984237	0.87037508	stable	0.990207998
P00746	CFD	0.351585415	0.561749146	stable	0.988954333
P00742	F10	-0.231119802	0.708370345	stable	0.988954333
P00740	F9	-0.128299428	0.837093367	stable	0.990207998
P00739	HPR	0.668425044	0.217435072	stable	0.988954333
P00738	HP	0.215062209	0.728300003	stable	0.988954333
P00736	C1R	-0.318042848	0.601989565	stable	0.988954333
P00734	F2	-0.683960871	0.202862546	stable	0.988954333
P00568	AK1	0.84316663	0.072778232	stable	0.988954333
P00558	PGK1	-0.531212136	0.356954077	stable	0.988954333
P00505	GOT2	0.187101189	0.763172675	stable	0.988954333
P00492	HPRT1	-0.899284056	0.03778409	negative	0.988954333
P00491	PNP	-0.652060978	0.233082975	stable	0.988954333
P00488	F13A1	0.586216741	0.298886125	stable	0.988954333
P00450	CP	-0.426565576	0.473832354	stable	0.988954333
P00441	SOD1	-0.730451639	0.161027138	stable	0.988954333
P00390	GSR	0.258643102	0.674394781	stable	0.988954333
P00387	CYB5R3	0.637205201	0.247542924	stable	0.988954333
P00352	ALDH1A1	-0.006957235	0.991141845	stable	0.997512333
P00338	LDHA	0.337174476	0.578975155	stable	0.988954333
P00326	ADH1C	-0.473496538	0.420478645	stable	0.988954333
P00167	CYB5A	0.203258518	0.742996416	stable	0.988954333
O96000	NDUFB10	-0.872259735	0.053743262	stable	0.988954333
O95980	RECK	-0.132763344	0.831458367	stable	0.990207998
O95881	TXNDC12	0.647740497	0.237263835	stable	0.988954333
O95866	MPIG6B	-0.515826117	0.373644793	stable	0.988954333
O95865	DDAH2	0.391531246	0.514533891	stable	0.988954333
O95825	CRYZL1	0.976871161	0.004207771	positive	0.988954333
O95819	MAP4K4	-0.7931228	0.109381662	stable	0.988954333
O95810	CAVIN2	0.190064534	0.759467326	stable	0.988954333
O95793	STAU1	-0.084629695	0.89237489	stable	0.990207998
O95786	RIGI	0.483373114	0.409436106	stable	0.988954333
O95782	AP2A1	0.42086213	0.4804101	stable	0.988954333
O95777	LSM8	-0.104262747	0.867489458	stable	0.990207998
O95747	OXSR1	0.521441932	0.367531347	stable	0.988954333
O95721	SNAP29	-0.642999403	0.241874961	stable	0.988954333
O95573	ACSL3	0.240582925	0.696661478	stable	0.988954333
O95498	VNN2	-0.737434431	0.154988488	stable	0.988954333
O95479	H6PD	0.457914972	0.438034731	stable	0.988954333
O95477	ABCA1	0.492676801	0.399096785	stable	0.988954333
O95466	FMNL1	0.452107158	0.444619626	stable	0.988954333
O95445	APOM	-0.67137121	0.214650004	stable	0.988954333
O95433	AHSA1	0.677018684	0.209339394	stable	0.988954333
O95394	PGM3	0.294815304	0.630140309	stable	0.988954333
O95379	TNFAIP8	-0.677779955	0.208626377	stable	0.988954333
O95373	IPO7	-0.666196076	0.219548762	stable	0.988954333

O95347	SMC2	-0.574051645	0.311502129	stable	0.988954333
O95260	ATE1	-0.938250896	0.018248024	negative	0.988954333
O95236	APOL3	0.003403229	0.995666882	stable	0.998856419
O95232	LUC7L3	-0.201682199	0.744961878	stable	0.988954333
O95218	ZRANB2	-0.713348316	0.176095706	stable	0.988954333
O95197	RTN3	0.413018571	0.489487341	stable	0.988954333
O95084	PRSS23	-0.160543094	0.79647169	stable	0.988954333
O94921	CDK14	0.953122424	0.012097733	positive	0.988954333
O94919	ENDOD1	0.60873219	0.275899129	stable	0.988954333
O94874	UFL1	0.18879443	0.761055194	stable	0.988954333
O94813	SLIT2	-0.660584444	0.224895119	stable	0.988954333
O94804	STK10	-0.950765498	0.013016811	negative	0.988954333
O76094	SRP72	-0.372977061	0.536363318	stable	0.988954333
O75995	SASH3	0.440009773	0.458405121	stable	0.988954333
O75962	TRIO	0.571446079	0.314221593	stable	0.988954333
O75955	FLOT1	-0.036954733	0.952958484	stable	0.990577889
O75937	DNAJC8	-0.078409374	0.900268476	stable	0.990207998
O75888	TNFSF13	-0.673726815	0.212430402	stable	0.988954333
O75882	ATRN	0.168533908	0.786436165	stable	0.988954333
O75874	IDH1	0.731027226	0.160526845	stable	0.988954333
O75822	EIF3J	-0.447907174	0.449395148	stable	0.988954333
O75821	EIF3G	0.708800559	0.180167008	stable	0.988954333
O75787	ATP6AP2	0.148711405	0.81135499	stable	0.988954333
O75695	RP2	-0.139708351	0.822698172	stable	0.990207998
O75636	FCN3	-0.524838725	0.363845442	stable	0.988954333
O75563	SKAP2	-0.60077728	0.283965537	stable	0.988954333
O75558	STX11	-0.159534866	0.797738859	stable	0.988954333
O75531	BANF1	0.689978092	0.197295168	stable	0.988954333
O75475	PSIP1	-0.614700252	0.269887936	stable	0.988954333
O75390	CS	0.917025189	0.028331711	positive	0.988954333
O75369	FLNB	0.421666037	0.479481788	stable	0.988954333
O75368	SH3BGRL	-0.700377705	0.187777055	stable	0.988954333
O75367	MACROH2A1	-0.372281016	0.537185723	stable	0.988954333
O75351	VPS4B	-0.566643882	0.319249448	stable	0.988954333
O75348	ATP6V1G1	-0.219177307	0.723185489	stable	0.988954333
O75347	TBCA	0.814619036	0.093104473	stable	0.988954333
O75340	PDCD6	0.409895579	0.49311147	stable	0.988954333
O75251	NDUFS7	-0.725687479	0.165185326	stable	0.988954333
O75165	DNAJC13	-0.908371503	0.032833624	negative	0.988954333
O75131	CPNE3	0.170857206	0.783520954	stable	0.988954333
O75122	CLASP2	0.231620686	0.707749903	stable	0.988954333
O75116	ROCK2	-0.224973218	0.71599019	stable	0.988954333
O75083	WDR1	0.123371981	0.843317312	stable	0.990207998
O75044	SRGAP2	0.87830363	0.050021802	stable	0.988954333
O60927	PPP1R11	0.508708614	0.38142769	stable	0.988954333
O60888	CUTA	-0.367957673	0.542299452	stable	0.988954333
O60884	DNAJA2	0.580926237	0.304356223	stable	0.988954333
O60841	EIF5B	-0.507335457	0.382933628	stable	0.988954333
O60826	CCDC22	0.457412353	0.438603731	stable	0.988954333
O60763	USO1	-0.478299863	0.41509982	stable	0.988954333
O60749	SNX2	0.046034591	0.941407647	stable	0.990577889
O60664	PLIN3	0.198872053	0.748467373	stable	0.988954333
O60645	EXOC3	0.505967085	0.384435723	stable	0.988954333
O60610	DIAPH1	0.464921391	0.430120333	stable	0.988954333
O60506	SYNCRIP	0.009733527	0.987607085	stable	0.997512333
O60493	SNX3	0.433257581	0.466139441	stable	0.988954333
O60488	ACSL4	0.854514846	0.065140314	stable	0.988954333
O60476	MAN1A2	-0.265215474	0.666318751	stable	0.988954333

O60331	PIP5K1C	0.554392491	0.332167429	stable	0.988954333
O60268	KIAA0513	0.558623548	0.327691565	stable	0.988954333
O60262	GNG7	-0.072563506	0.907690419	stable	0.990207998
O60256	PRPSAP2	-0.074362897	0.905405554	stable	0.990207998
O60234	GMFG	-0.556582554	0.329848744	stable	0.988954333
O60229	KALRN	-0.183164488	0.768098388	stable	0.988954333
O43866	CD5L	-0.002670512	0.996599802	stable	0.99925882
O43865	AHCYL1	-0.713110308	0.176308117	stable	0.988954333
O43852	CALU	0.222951676	0.718498714	stable	0.988954333
O43813	LANCL1	0.089199098	0.886578967	stable	0.990207998
O43790	KRT86	-0.227326225	0.713071865	stable	0.988954333
O43776	NARS1	-0.361495726	0.54996028	stable	0.988954333
O43768	ENSA	0.401478219	0.502907186	stable	0.988954333
O43760	SYNGR2	0.497525295	0.393733218	stable	0.988954333
O43707	ACTN4	-0.434106682	0.46516529	stable	0.988954333
O43684	BUB3	0.074545179	0.90517411	stable	0.990207998
O43670	ZNF207	0.371630649	0.537954383	stable	0.988954333
O43665	RGS10	-0.345799204	0.568653978	stable	0.988954333
O43639	NCK2	-0.910236841	0.031845306	negative	0.988954333
O43633	CHMP2A	-0.218061934	0.724571273	stable	0.988954333
O43583	DENR	-0.41930747	0.482206424	stable	0.988954333
O43566	RGS14	0.567970624	0.317858333	stable	0.988954333
O43516	WIPF1	-0.543615446	0.343636233	stable	0.988954333
O43491	EPB41L2	0.149730833	0.810071546	stable	0.988954333
O43488	AKR7A2	0.854953539	0.064850384	stable	0.988954333
O43432	EIF4G3	0.197738651	0.749881807	stable	0.988954333
O43396	TXNL1	-0.037356102	0.952447798	stable	0.990577889
O43390	HNRNPR	-0.774112132	0.124417428	stable	0.988954333
O43324	EEF1E1	0.291271159	0.634454748	stable	0.988954333
O43314	PPIP5K2	0.188342649	0.761620099	stable	0.988954333
O43312	MTSS1	-0.285369556	0.641649793	stable	0.988954333
O43242	PSMD3	-0.069027308	0.912181547	stable	0.990207998
O43236	SEPTIN4	0.085936751	0.890716758	stable	0.990207998
O43182	ARHGAP6	-0.248287308	0.687149545	stable	0.988954333
O43157	PLXNB1	0.682771344	0.203968285	stable	0.988954333
O43150	ASAP2	-0.529498227	0.358804108	stable	0.988954333
O43143	DHX15	-0.688240652	0.198898238	stable	0.988954333
O15530	PDPK1	0.748447904	0.145602476	stable	0.988954333
O15523	DDX3Y	-0.098292175	0.875052328	stable	0.990207998
O15511	ARPC5	-0.51713873	0.372213687	stable	0.988954333
O15498	YKT6	-0.70355861	0.184892578	stable	0.988954333
O15439	ABCC4	0.130165331	0.834737549	stable	0.990207998
O15438	ABCC3	2.12E-05	0.999973019	stable	0.999973019
O15427	SLC16A3	0.432255793	0.467289332	stable	0.988954333
O15400	STX7	0.912995083	0.030401779	positive	0.988954333
O15372	EIF3H	-0.515455801	0.374048777	stable	0.988954333
O15371	EIF3D	-0.810230792	0.096362206	stable	0.988954333
O15347	HMGB3	0.386778809	0.520107889	stable	0.988954333
O15305	PMM2	-0.775881523	0.1229937	stable	0.988954333
O15240	VGF	0.871338701	0.054317723	stable	0.988954333
O15230	LAMA5	-0.366039375	0.544571475	stable	0.988954333
O15162	PLSCR1	-0.269055779	0.661606821	stable	0.988954333
O15145	ARPC3	-0.23136684	0.70806433	stable	0.988954333
O15144	ARPC2	-0.484284383	0.408420687	stable	0.988954333
O15143	ARPC1B	-0.095132381	0.879056641	stable	0.990207998
O15127	SCAMP2	-0.492186394	0.399640237	stable	0.988954333
O15117	FYB1	-0.200215559	0.746791171	stable	0.988954333
O15050	TRANK1	0.732357391	0.159372416	stable	0.988954333

O15031	PLXNB2	0.416673397	0.485253193	stable	0.988954333
O15021	MAST4	0.330847377	0.586568394	stable	0.988954333
O14980	XPO1	-0.485760698	0.406776884	stable	0.988954333
O14979	HNRNPDL	0.357800121	0.55435082	stable	0.988954333
O14974	PPP1R12A	-0.203735581	0.74240171	stable	0.988954333
O14950	MYL12B	0.39630476	0.508947491	stable	0.988954333
O14818	PSMA7	0.337697113	0.578348743	stable	0.988954333
O14791	APOL1	-0.227825181	0.712453245	stable	0.988954333
O14786	NRP1	-0.150030365	0.809694478	stable	0.988954333
O14745	NHERF1	0.354297869	0.558517799	stable	0.988954333
O14737	PDCD5	0.47381139	0.420125584	stable	0.988954333
O14672	ADAM10	-0.286777972	0.639931487	stable	0.988954333
O14656	TOR1A	0.627755188	0.256862634	stable	0.988954333
O14617	AP3D1	0.260157493	0.6725326	stable	0.988954333
O14602	EIF1AY	-0.783914126	0.116592396	stable	0.988954333
O14530	TXNDC9	-0.65294822	0.232226926	stable	0.988954333
O00743	PPP6C	0.709712804	0.179348204	stable	0.988954333
O00571	DDX3X	0.504653669	0.385878808	stable	0.988954333
O00560	SDCBP	0.344713499	0.569951337	stable	0.988954333
O00522	KRIT1	-0.327199746	0.590954113	stable	0.988954333
O00506	STK25	-0.240586066	0.696657597	stable	0.988954333
O00499	BIN1	0.09009929	0.885437422	stable	0.990207998
O00478	BTN3A3	0.603599002	0.281097271	stable	0.988954333
O00471	EXOC5	-0.939625309	0.017645892	negative	0.988954333
O00461	GOLIM4	-0.705950886	0.182731634	stable	0.988954333
O00429	DNM1L	0.78386386	0.116632134	stable	0.988954333
O00410	IPO5	0.338417005	0.577486112	stable	0.988954333
O00391	QSOX1	0.775140637	0.123589254	stable	0.988954333
O00339	MATN2	-0.185650078	0.764987913	stable	0.988954333
O00303	EIF3F	-0.24548148	0.690611449	stable	0.988954333
O00299	CLIC1	0.953468718	0.01196456	positive	0.988954333
O00267	SUPT5H	-0.865236497	0.058171805	stable	0.988954333
O00264	PGRMC1	0.35238778	0.560792921	stable	0.988954333
O00233	PSMD9	0.26538473	0.66611097	stable	0.988954333
O00231	PSMD11	0.759067884	0.136716833	stable	0.988954333
O00203	AP3B1	0.037340329	0.952467867	stable	0.990577889
O00194	RAB27B	0.564284576	0.321727033	stable	0.988954333
O00187	MASP2	-0.65065328	0.234442948	stable	0.988954333
O00186	STXBP3	0.798086653	0.105552828	stable	0.988954333
O00178	GTPBP1	0.319569622	0.600147052	stable	0.988954333
O00161	SNAP23	-0.196415408	0.751533569	stable	0.988954333
O00160	MYO1F	0.704610909	0.183941145	stable	0.988954333
O00159	MYO1C	-0.635206734	0.249506075	stable	0.988954333
O00151	PDLIM1	-0.158699273	0.798789215	stable	0.988954333
O00139	KIF2A	-0.289727162	0.636335847	stable	0.988954333
G9CGD6	CNK3/IPCEF1	-0.155047741	0.803380923	stable	0.988954333
E9PAV3	NACA	-0.021627323	0.972465384	stable	0.994727102
C4AMC7	WASH3P	0.42155288	0.479612433	stable	0.988954333
B9A064	IGLL5	0.28356844	0.643848299	stable	0.988954333
B2RUZ4	SMIM1	-0.274691262	0.654701784	stable	0.988954333
B0I1T2	MYO1G	-0.54478588	0.342385986	stable	0.988954333
A8MWD9	SNRPGP15	0.133824615	0.830119172	stable	0.990207998
A6NDG6	PGP	-0.432752092	0.466719585	stable	0.988954333
A6NCV1	OR6C74	0.249670375	0.685444025	stable	0.988954333
A2RTX5	TARS3	0.740705907	0.152182581	stable	0.988954333
A2NJV5	IGKV2-29	-0.48453863	0.408137485	stable	0.988954333
A0M8Q6	IGLC7	0.702490651	0.185859598	stable	0.988954333
A0A0J9YX35	IGHV3-64D	0.124331009	0.842105641	stable	0.990207998

A0A0G2JRQ6	A0A0G2JRQ6	0.83885383	0.075749065	stable	0.988954333
A0A0C4DH67	IGKV1-8	-0.93839492	0.01818462	negative	0.988954333
A0A0C4DH55	IGKV3D-7	-0.247638217	0.687950184	stable	0.988954333
A0A0C4DH38	IGHV5-51	-0.292701225	0.632713277	stable	0.988954333
A0A0C4DH31	IGHV1-18	-0.467824596	0.426850476	stable	0.988954333
A0A0C4DH29	IGHV1-3	0.084889826	0.892044872	stable	0.990207998
A0A0C4DH25	IGKV3D-20	0.625624421	0.258976771	stable	0.988954333
A0A0B4J2B5	IGHV3OR16-9	0.685608604	0.201333668	stable	0.988954333
A0A0B4J1Y9	IGHV3-72	0.131607304	0.832917366	stable	0.990207998
A0A0B4J1Y8	IGLV9-49	0.154524659	0.804038905	stable	0.988954333
A0A0B4J1V6	IGHV3-73	0.021015597	0.973244081	stable	0.994727102
A0A0B4J1V0	IGHV3-15	-0.533768626	0.354198909	stable	0.988954333
A0A0B4J1U7	IGHV6-1	-0.119918308	0.847682006	stable	0.990207998
A0A0A0MT36	IGKV6D-21	-0.448971611	0.448183778	stable	0.988954333
A0A0A0MS15	IGHV3-49	0.675378501	0.210877918	stable	0.988954333
A0A0A0MRZ8	IGKV3D-11	0.678722264	0.207744738	stable	0.988954333
A0A087WSY6	IGKV3D-15	-0.775798525	0.123060375	stable	0.988954333
A0A087WSX0	IGLV5-45	-0.234693461	0.703945356	stable	0.988954333
A0A075B6S2	IGKV2D-29	-0.401347512	0.503059611	stable	0.988954333
A0A075B6P5	IGKV2-28	-0.432212626	0.467338894	stable	0.988954333
A0A075B6K5	IGLV3-9	-0.615569706	0.269015134	stable	0.988954333
A0A075B6K4	IGLV3-10	-0.607787524	0.276853826	stable	0.988954333
A0A075B6J9	IGLV2-18	-0.136691192	0.826502875	stable	0.990207998
A0A075B6I9	IGLV7-46	0.436991936	0.461858429	stable	0.988954333
A0A075B6I0	IGLV8-61	-0.501843618	0.388970591	stable	0.988954333

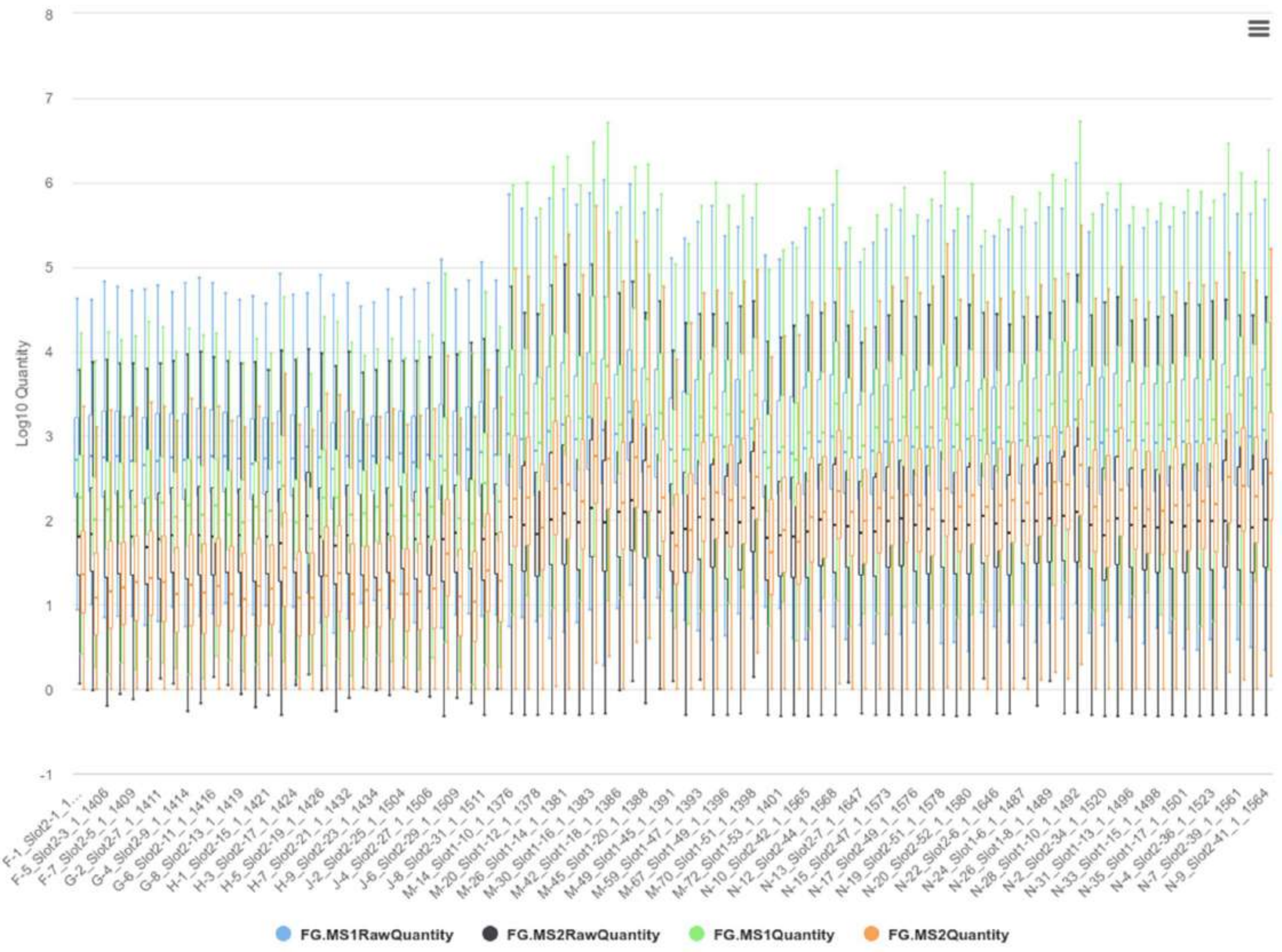

Supplementary Figure 1. Normalized intensity distributions demonstrating data quality. Box plots showing the normalized MS1 and MS2 intensity distributions (log10scale) across samples.

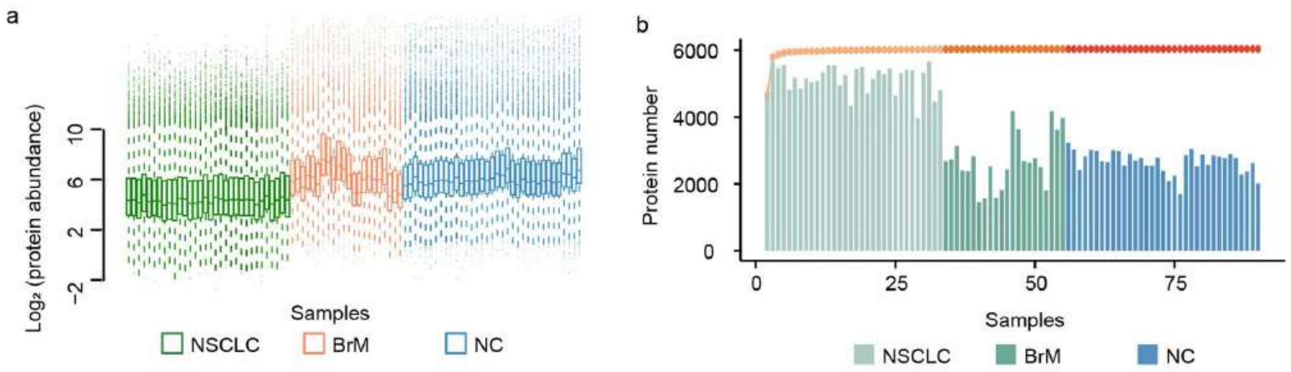

Supplementary Figure 2. Overview of plasma proteome profiling in the study cohorts. a) Box plots showing quantified protein intensities and median values in NSCLC (n = 32), BrM (n = 22), and normal control (NC) groups. b) Bar plot showing the number of proteins identified in each sample, with a line plot showing the cumulative number of identified proteins.

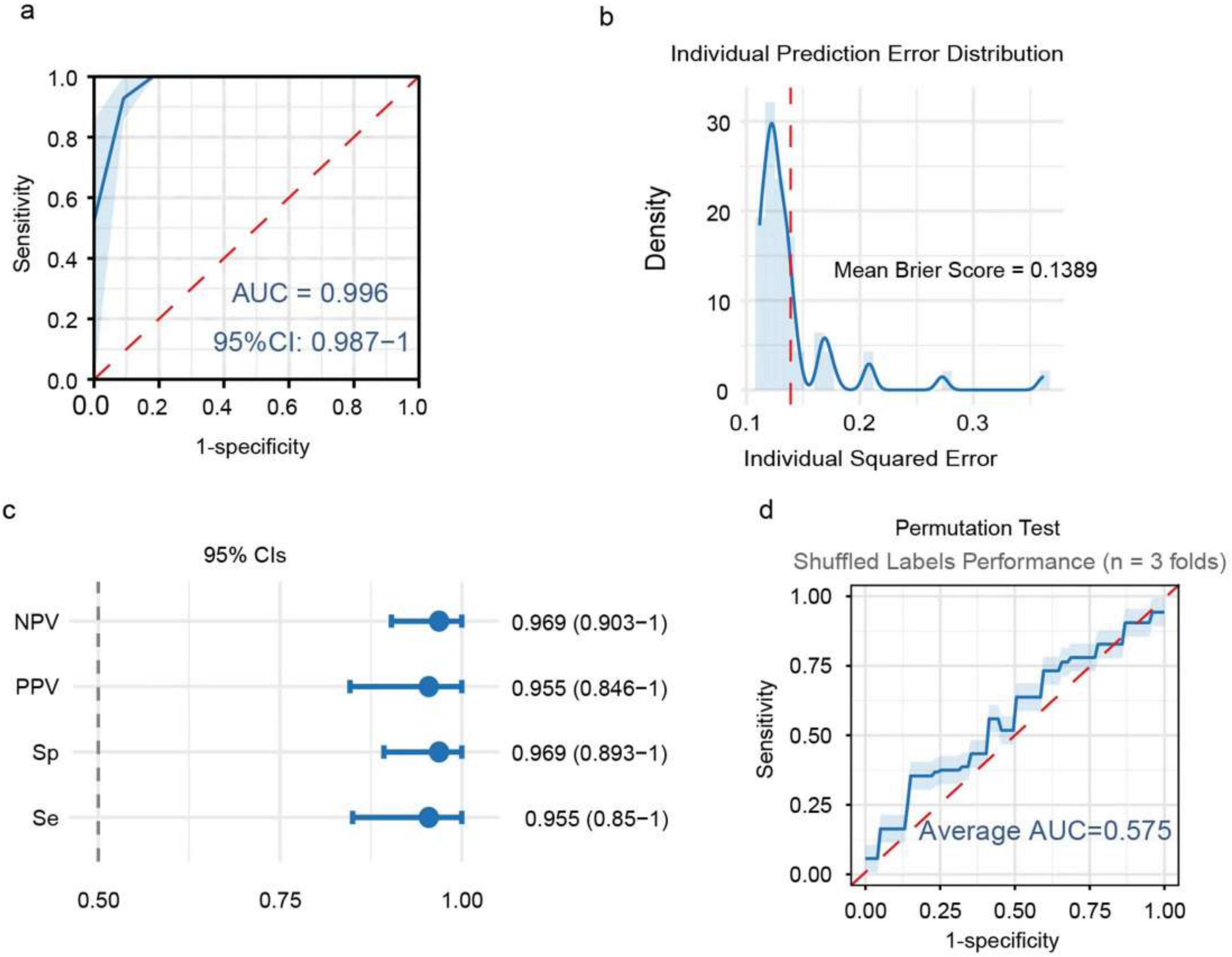

Supplementary Figure 3. Comprehensive evaluation of the machine learning model using nested cross-validation and permutation testing. a) ROC curve from the primary analysis (BrM vs. NSCLC) in outer-fold validation, showing AUC with 95% confidence intervals. b) Calibration plot showing agreement between predicted probabilities and observed outcomes across risk strata. c) Classification performance metrics with 95% confidence intervals, including sensitivity, specificity, positive predictive value (PPV), and negative predictive value (NPV). d) ROC curves from permutation testing using shuffled labels, showing the average AUC across three folds.

Supplemental Methods

Serum sample preparation used RSP-MOSF

Serum sample preparation was conducted using our previously developed method known as RSP-MOSF. Specifically, 100 ng of a trypsin and Lys-C mixture (maintaining a fixed ratio of trypsin to Lys-C at 2:1) was immobilized in 500 μL of a 25 mM ammonium bicarbonate solution containing 0.5 mg of MOSF. The mixtures were then agitated at 25 °C for one hour to achieve adsorption equilibrium. Subsequently, the enzyme-loaded materials were washed three times with 1 mL of the same ammonium bicarbonate solution. The enzyme-loaded materials were either stored at −20 °C or resuspended in 100 μL of the 25 mM ammonium bicarbonate solution for protein digestion.

This method has now been adapted into a commercial kit (LABP magnetic bead, OSFP0002, Shanghai Omicsolution Co., Ltd., China). Therefore, in this study, we utilized the commercial kit directly for serum sample preparation.
